# Evaluation of the pain intensity differences among hospitalized cancer patients based on a nursing information system

**DOI:** 10.1371/journal.pone.0222516

**Published:** 2019-09-25

**Authors:** Wei-Yun Wang, Chi-Ming Chu, Yi-Syuan Wu, Chun-Sung Sung, Shung-Tai Ho, Hsueh-Hsing Pan, Kwua-Yun Wang

**Affiliations:** 1 Department of Nursing, Tri-Service General Hospital, Taipei, Taiwan; 2 School of Nursing, National Defense Medical Center, Taipei, Taiwan; 3 School of Public Health, National Defense Medical Center, Taipei, Taiwan; 4 Graduate Institute of Life Science, National Defense Medical Center, Taipei, Taiwan; 5 Department of Anesthesiology, Taipei Veterans General Hospital, Taipei, Taiwan; 6 School of Medicine, National Yang-Ming University, Taipei, Taiwan; 7 Department of Anesthesiology, Taipei Veterans General Hospital, Taipei, Taiwan; 8 School of Nursing, National Defense Medical Center, Taipei, Taiwan; 9 Department of Nursing, Taipei Veterans General Hospital, Taipei, Taiwan; 10 School of Nursing, National Defense Medical Center, Taipei, Taiwan; University Magna Graecia of Catanzaro, ITALY

## Abstract

Evaluating the absolute difference in pain intensity and the percentage difference in pain intensity could facilitate an understanding of pain reduction among cancer patients during repeated hospitalizations. Examinations of the absolute differences in pain intensity and the percentage differences in pain intensity according to the worst pain intensity and last evaluated pain intensity before discharge are lacking. The aim of this study was to evaluate the absolute and percentage difference in pain intensities among cancer patients with moderate or severe pain from their 1st to 18th hospitalizations from 2011–2013. A population-based retrospective cohort study was conducted. Pain intensity was assessed using scales and was recorded in a nursing information system. The absolute and percentage difference in pain intensities were examined via the one-sample Kolmogorov-Smirnov test, and group differences in moderate or severe pain were evaluated with the Mann-Whitney U test. For moderate pain patients, the mean absolute difference in pain intensity was 1.52, and the percentage difference in pain intensity was 29.0%; both these values were significant. More significant changes in the absolute and percentage difference in pain intensities were associated with severe pain patients. Both the average absolute difference in pain intensity (3.09) and the percentage difference in pain intensity (38.5%) in patients with severe pain were significantly higher than the average absolute difference in pain intensity (1.52) and the percentage difference in pain intensity (29.0%) in patients with moderate pain. Cancer patients with moderate and severe pain experienced pain reductions of approximately 30% and 40%, respectively. Early pain management intervention in patients with severe pain is necessary to achieve an obvious analgesic effect, and the formula of the percentage difference in pain intensity should be incorporated into the nursing information system to alert clinicians for early detection of the effectiveness of cancer pain management.

## Introduction

The absolute pain intensity difference (PID) and the percentage difference in pain intensity (%PID) are common methods for evaluating the magnitude of pain reduction [1]. The raw PID data is easy to calculate but may lead to inconsistent results, especially when variations in baseline pain intensity occur. Therefore, the %PID was calculated as a percentage difference to adjust for the baseline pain intensity to provide a more consistent and clinically relevant measure of pain intensity and has the potential to increase comparability between studies [2]. A reduction of more than 2 points in the PID or a 30% reduction in the %PID indicates a significant decrease in pain [3].

Patient-reported pain intensity is commonly measured using a numerical rating scale (NRS), the Faces Pain Scale (FPS), or the Faces, Legs, Activity, Cry, and Consolability (FLACC) Behavioral Tool, which are important pain measurement instruments that quantify a patient’s perception of pain on a scale of 0 to 10 [4,5]. When pain intensity is greater than 7 points, pain is considered severe. Pain intensities between 4 and 6 points are defined as moderate pain [6]. More than one-third of cancer patients experience moderate or severe pain [7]. Despite clear recommendations from the World Health Organization (WHO), pain is still a major problem experienced by cancer patients during repeated hospitalizations [8–10].

Due to cancer treatment regimens, cancer patients may require repeated hospitalizations to receive inpatient care [11–12]. The use of traditional manual chart records to obtain pain intensities during repeated hospitalizations increases time and cost. This information gap presents a challenge, and it may be difficult to integrate the collection of valid outcome measures into a demanding clinical practice in which time and cost-containment pressures already exist. Real-time availability of data essentially requires electronic data capture, followed by automatic reporting and minimization to make data collection as efficient as possible to produce meaningful results [13]. Therefore, a nursing information system (NIS) should be established to routinely document patient-reported pain intensity. Previous studies have suggested that a patient-reported outcome data collection system should be developed for many pain programs [6,14]. The pain intensity data accumulated in a NIS could be used to establish a hospital-wide pain scoring protocol. Therefore, implementation of a NIS to collect patient-reported pain intensity data is a critical step for implementing a hospital-wide pain score database [13].

Repeated hospitalizations are also a potential indicator for examining the level of aggressiveness of cancer care [12,15] and for identifying cancer pain trends [10]. Limited information is currently available regarding pain reduction measures, such as the PID and %PID, in cancer patients who experience moderate or severe pain during repeated hospitalizations. These values can be examined over a long period according to the worst pain intensity (WPI) and the last evaluated pain intensity (LPI) before discharge. Thus, the aim of this study was to evaluate the PID and %PID among hospitalized cancer patients with moderate or severe pain during each hospitalization based on a NIS.

## Materials and methods

### Study design

This study, which was a single-center, hospital-based, retrospective cohort study conducted at a national academic medical center in Taiwan, was approved by the Institutional Review Board of our institution. All data were fully anonymized before assessment. The research was supported by the chief executive officer, medical director and nursing officer of the study hospital.

Since cancer patients repeatedly require hospitalization to receive inpatient clinical care, we examined their pain scores over multiple hospitalizations. For each patient during each hospitalization, we chose only one pain score, which included two measures: the WPI and the LPI before discharge. A pain intensity greater than 7 points was considered indicative of severe pain, and a pain intensity rated between 4 and 6 points was defined as moderate pain [6,16–18]. Based on the cut-off points of 4 and 7, the PIDs of moderate or severe pain were examined based on the WPI among cancer patients during each hospitalization. The PIDs were calculated according to (a) the absolute pain intensity difference (PID) (PID = WPI—LPI) for each hospitalization (e.g., if a patient with one hospitalization had recorded pain intensities of 4, 6, 9, 8, 7, 5, and 3, then the WPI was 9 points, the LPI was 3 points, and the PID was 6 points) and (b) the percentage difference in pain intensity (%PID) [%PID = (PID / WPI) × 100] for each hospitalization [1–3] (using the above example, [%PID = (6 / 9) × 100] = 66.7%).

### Setting

Our academic center manages approximately 97,000 hospitalizations per year and has approximately 2,800 inpatient beds and about 2,700 nursing staff to provide healthcare services. An electronic NIS was introduced at the medical center in 2011 and has been used for all hospitalized patients to routinely document and chart vital signs, including patient-reported pain intensity scores. Before 2011, all nurses participated in an educational program that was developed by the research team. The training program taught proper pain assessment and pain documentation techniques using a NIS to meet the policy of pain management at the center. The training program provided a cornerstone for quality pain assessments and pain documentation for all nursing staff. The integrity analysis of pain assessment and documentation was 97.3%-99.9% [19], indicating that the nursing staff were consistent. In this manner, systematic pain assessments and pain documentation in the NIS were implemented in clinical settings.

All patients were systematically assessed at least once per day by the nurses when they reported no pain at all or when their pain score was < 4 and tolerable, whereas they were assessed three times per day when the pain score was ≥ 4 or < 4 and intolerable; the patients were assessed as needed when pain medication was indicated. The tools used by the nurses to assess pain included the NRS, the FPS and the FLACC Behavioral Tool. The NRS and the FPS demonstrate good reliability and validity when used to evaluate pain intensities experienced by conscious, alert and cooperative patients [4,20–23]. The FLACC Behavioral Tool was used for nonalert or uncooperative patients to quantify the clinician’s perception of the patients’ pain. The intraclass correlation coefficient of the FLACC Behavioral Tool was 0.96, and Cronbach’s α was 0.88 [5]. These three pain scales are all 11-point pain scales, with 0 points indicating no pain at all and 10 points indicating the worst possible pain [5,20].

After each pain assessment, the pain intensity score was recorded by nurses using handheld devices at the patient’s bedside in the academic center. The nurses immediately input the patient-reported pain intensity into the NIS; immediate data entry ensured the accuracy of the recorded pain intensities. After the pain intensity was entered and stored in the NIS, all clinicians could retrieve the pain intensity scores. Due to the real-time availability of the data, clinicians could manage pain immediately.

### Data retrieval

**Fig 1:**
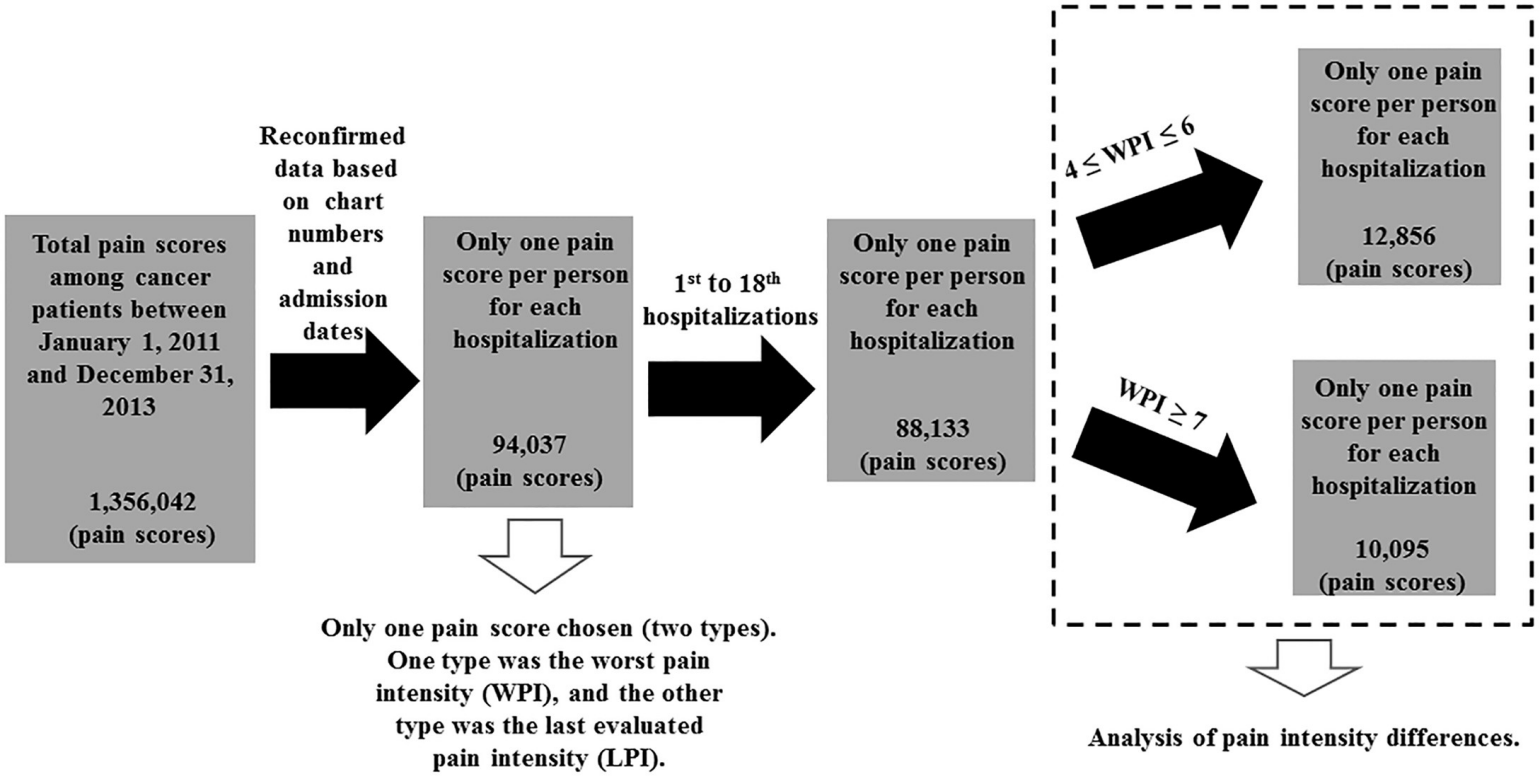
Retrieval of moderate and severe pain scores from the nursing information system database. In all, 1,356,042 pain scores were collected over the three-year study period. After we reconfirmed the data according to chart numbers and admission dates, the number of pain scores was reduced to 94,037. The number of hospitalizations per patient at this academic center ranged from one to 18, and 88,133 pain scores were assessed. To evaluate the PID in cancer patients with a WPI that indicated moderate or severe pain, the WPI scores were classified into the following two categories: moderate pain (4 ≤ WPI ≤ 6 points) and severe pain (WPI ≥ 7 points).

The pain intensity scores of all hospitalized patients were stored in the NIS as of 2011. All the cancer patients admitted between January 1, 2011, and December 31, 2013, were included in the analysis. According to the International Classification of Diseases, 9th Revision, the codes 140–209 and 230–239 were used to identify cancer patients. Through information technology management, we retrieved the pain scores of cancer patients recorded between January 1, 2011, and December 31, 2013. In all, 1,356,042 pain scores were collected, which means that the number of pain assessments were collected per patient for each hospitalization over the three-year period. According to the chart numbers and admission dates, we reconfirmed the pain scores and selected one pain score per patient for each hospitalization. The number of pain scores was then reduced to 94,037. After the 19th hospitalization, the number of pain assessments suddenly dropped. Therefore, the number of hospitalizations per patient at this medical center ranged from one to 18, and 88,133 pain scores were assessed. To evaluate the PID in cancer patients with moderate or severe pain at the time of the WPI, the WPI scores were classified into the following two categories: moderate pain (4 ≤ WPI ≤ 6 points) and severe pain (WPI ≥ 7 points). Fig 1 shows the process of retrieving moderate and severe pain data from the NIS database.

### Data analysis

Categorical data are expressed as person-times and percentages, and continuous data (WPI, LPI, PID and %PID values) are reported as the means and standard deviations. Since the PID and %PID data did not fit a normal distribution, we used nonparametric tests to determine significance. The PID and %PID were evaluated using the one-sample Kolmogorov-Smirnov test. The differences between groups in the moderate or severe pain categories based on the number of hospitalizations were evaluated using the Mann-Whitney U test. A *P*-value less than 0.05 was considered significant. All the data manipulations and analyses were performed using the Statistical Package for the Social Sciences (SPSS) version 21.0 software package for Windows (SPSS Inc., Chicago, IL, USA).

## Results

### Demographic characteristics of the cancer patients

In all, 24,430 patients with an average age of 59.3 ± 17.1 (range 0.3–101.6) years and corresponding 88,133 pain scores were analyzed in this study (Table 1). The moderate pain subset included 5,700 patients and 12,856 pain scores, and the mean age of these patients was 60.0 ± 16.8 (range 0.6–101.5) years. The severe pain subset included 4,446 patients and 10,095 pain scores, and the mean age of this group was 59.0 ± 17.3 (range 0.9–101.5) years. A greater number of males than females were included in this study.

**Table 1:** Pain intensity values for patients with moderate pain or severe pain during each hospitalization.

Number of hospitalizations	All person-times	Average period before the previous hospitalization (days)	Moderate pain							Severe pain						
			Person-times n (%)	WPI ^a^		LPI ^b^		PID ^c^	%PID ^d^	Person-times n (%)	WPI ^a^		LPI ^b^		PID ^c^	%PID ^d^
				Mean	Sd ^e^	Mean	Sd ^e^				Mean	Sd ^e^	Mean	Sd ^e^		
1	24,430	-	5,700 (23.3)	5.06	0.80	3.55	1.30	1.51***	28.6***	4,446 (18.3)	8.02	1.04	4.94	2.14	3.09***	38.4***
2	13,469	87.3	2,167 (16.1)	5.02	0.79	3.39	1.39	1.63***	31.3***	1,697 (12.6)	8.06	1.07	4.91	2.19	3.15***	39.0***
3	9,385	64.3	1,171 (12.5)	5.00	0.79	3.54	1.43	1.46***	28.2***	904 (9.7)	8.06	1.04	4.91	2.21	3.15***	39.2***
4	7,366	50.3	763 (10.4)	4.98	0.79	3.44	1.43	1.54***	29.8***	642 (8.7)	8.12	1.07	4.94	2.16	3.18***	39.1***
5	6,092	44.7	610 (10.0)	5.02	0.78	3.54	1.40	1.48***	28.3***	458 (7.5)	8.12	1.06	5.07	2.12	3.05***	37.5***
6	5,040	43.5	490 (9.7)	4.99	0.77	3.49	1.38	1.50***	28.8***	384(7.6)	8.09	1.07	5.02	2.18	3.07***	37.9***
7	4,156	42.6	394 (9.5)	5.00	0.80	3.45	1.46	1.56***	30.0***	318 (7.7)	8.08	1.03	5.18	2.28	2.90***	36.3***
8	3,365	40.8	298 (8.9)	4.99	0.83	3.57	1.31	1.42***	27.1***	267 (7.9)	8.04	1.01	5.04	2.23	3.00***	37.3***
9	2,764	36.8	263 (9.5)	4.94	0.83	3.42	1.37	1.52***	28.7***	199 (7.2)	8.15	1.10	5.11	2.31	3.05**	37.4*
10	2,277	35.2	198 (8.7)	4.95	0.82	3.49	1.42	1.46***	28.3***	168 (7.4)	7.99	0.97	4.93	2.27	3.06**	38.7**
11	1,995	33.3	156 (7.8)	5.00	0.82	3.56	1.44	1.44***	27.5***	136 (6.8)	8.05	0.98	5.21	2.21	2.84	35.5
12	1,726	29.4	141 (8.2)	4.94	0.83	3.43	1.39	1.52***	28.9***	112 (6.5)	8.08	1.10	4.89	2.23	3.19*	39.6
13	1,492	31.6	132 (8.8)	4.91	0.81	3.64	1.40	1.27***	24.4***	92 (6.2)	7.95	0.97	4.91	1.91	3.03	37.9
14	1,228	31.8	101 (8.2)	4.85	0.81	3.32	1.33	1.53***	29.5***	81 (6.6)	7.94	0.94	4.58	2.01	3.36**	42.5
15	1,021	32.1	73 (7.1)	5.04	0.84	3.71	1.37	1.33***	25.4***	70 (6.9)	7.94	0.90	4.80	1.95	3.14	39.2
16	881	28.7	76 (8.6)	4.92	0.83	3.79	1.28	1.13***	21.8***	49 (5.6)	7.98	0.88	5.04	2.06	2.94	36.1
17	770	29.9	73 (9.5)	4.88	0.80	3.14	1.47	1.74**	33.9**	37 (4.8)	8.00	1.03	4.81	2.21	3.19	40.1
18	676	25.2	50 (7.4)	4.98	0.80	3.28	1.43	1.70**	32.6**	35 (5.2)	8.00	1.11	5.14	2.00	2.86	35.8
	88,133^f^	40.4^g^	12,856^f^ (14.6)	5.02^g^	0.79^g^	3.50^g^	1.36^g^	1.52^g^***	29.0^g^***	10,095^f^ (11.5)	8.05^g^	1.04^g^	4.96^g^	2.17^g^	3.09^g^***	38.5^g^

^a^ WPI: worst pain intensity;

^b^ LPI: last evaluated pain intensity;

^c^ PID: absolute difference in pain intensity;

^d^ %PID: percentage difference in pain intensity;

^e^ Sd: standard deviation;

^f^ total number of person-times;

^g^ average of the 1st to the 18th hospitalizations;

***P < 0.001;

**P < 0.01;

*P < 0.05.

### Pain intensity differences during each hospitalization

The average interval between two successive hospitalizations was 40.4 days (Table 1). The average interval between two hospitalizations was the longest between the 1st and 2nd hospitalizations (87.3 days) and the shortest between the 17th and 18th hospitalizations (25.2 days). Among the moderate pain group, the mean PID was 1.52 (range 1.13–1.74), which was statistically significant (one-sample Kolmogorov-Smirnov test, *P* < 0.001). The average %PID from the 1st to the 18th hospitalizations was 29.0% (range 21.8%-33.9%), which was also statistically significant (one-sample Kolmogorov-Smirnov test, *P* < 0.001). Significant changes were also observed in the PID and %PID values associated with the severe pain group; for this category, the mean PID was 3.09 (range 2.84–3.36), and the average %PID was 38.5% (range 35.5%-42.5%) (Table 1).

### Group differences in pain intensity during each hospitalization

**Fig 2:**
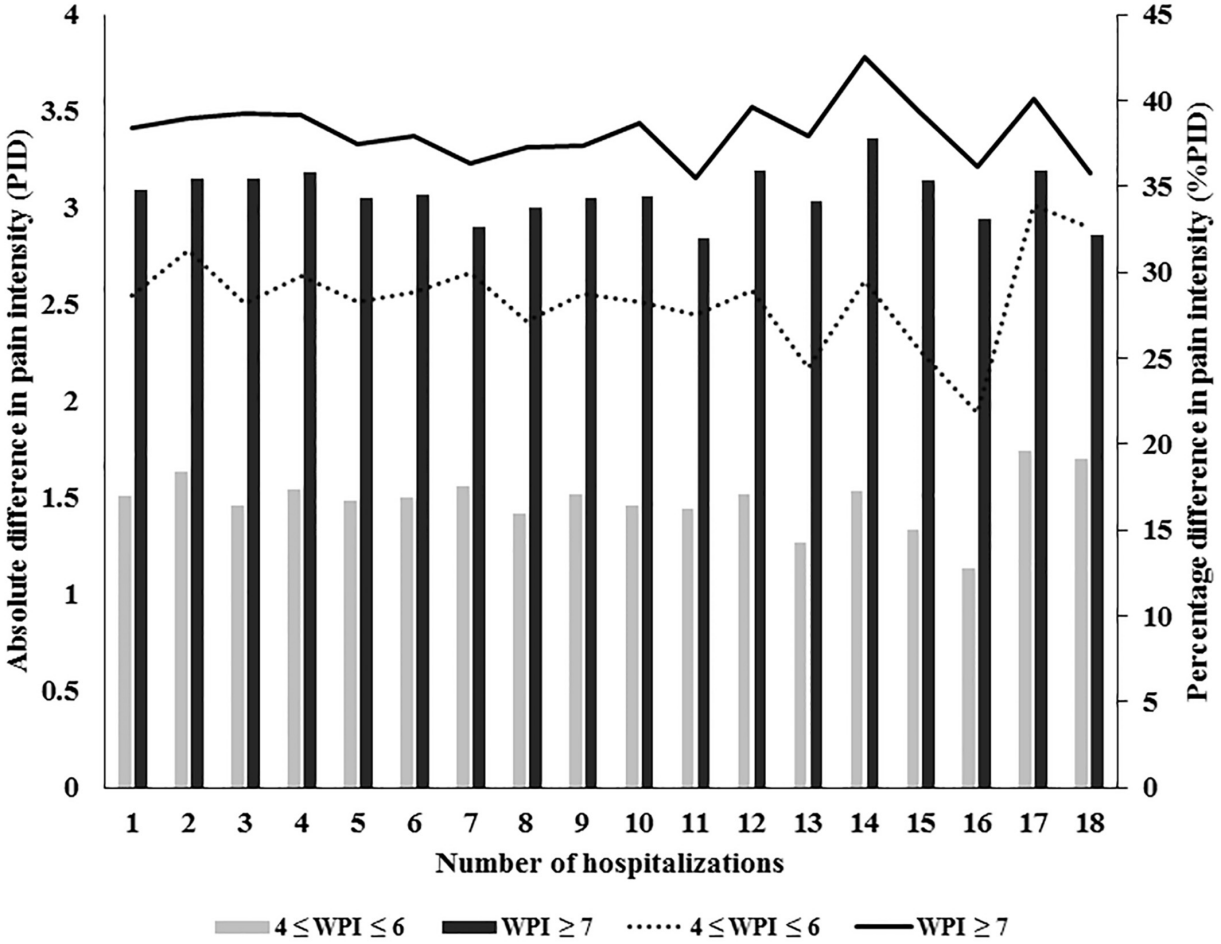
Differences in the PID and %PID between the moderate and severe pain group. The combined figure includes the left axis, right axis, and horizontal axis. The left axis indicates the PID of the moderate pain (gray bar) and severe pain (black bar) groups. The right axis indicates the %PID of the moderate pain (dotted line) and severe pain (solid line) groups. The horizontal axis indicates the 1st to 18th hospitalizations. The PID of the black bar was approximately 2.84–3.36 from the 1st to 18th hospitalizations in the severe pain group. The PID of the gray bar was approximately 1.13–1.74 from the 1st to 18th hospitalizations in the moderate pain group. The PID in the WPI ≥ 7 group was higher than the PID in the 4 ≤ WPI ≤ 6 group during each hospitalization. The %PID of the solid line was approximately 35.5–42.5% from the 1st to 18th hospitalizations in the severe pain group. The %PID of the dotted line was approximately 21.8–33.9% from the 1st to 18th hospitalizations in the moderate pain group. The %PID in the WPI ≥ 7 group was also higher than the %PID in the 4 ≤ WPI ≤ 6 group during each hospitalization.

The average PID in the severe pain group (3.09) was significantly higher than the average PID in the moderate pain group (1.52) (Mann-Whitney U test, *P* < 0.001). The average %PID in the severe pain group (38.5%) was also significantly higher than the average %PID in the moderate pain group (29.0%) (Mann-Whitney U test, *P* < 0.001). The PID values of the WPI ≥ 7 group (black bar) were higher than those of the 4 ≤ WPI ≤ 6 group (gray bar) from the 1st to the 18th hospitalizations. The %PID values in the WPI ≥ 7 group (solid line) were also higher than those in the 4 ≤ WPI ≤ 6 group (line) at each hospitalization. The results shown in Fig 2 reveal the group variations in the PID and %PID at each hospitalization.

## Discussion

This hospital-based study used 3-year data from 2011–2013 from the NIS database installed at a medical center in Taiwan to examine the PID and %PID values among cancer patients with moderate and severe pain during each hospitalization. Importantly, the results showed the magnitude of the decline in PID and %PID among cancer patients for each hospitalization. For moderate pain patients, the range of the PID was 1.13–1.74, and the mean %PID was 29.0%. For severe pain patients, the range of the PID was 2.84–3.36, and the mean %PID was 38.5%. Previously, the PID and %PID were considered the most common indicators for evaluating pain reduction [24–27]. Marinsek et al. [28] suggested that a decrease in pain intensity of ≥ 2 points between the initial and subsequent NRS measurements predicts satisfactory pain relief. A reduction of 2.0–2.7 points in the PID or 30%-36% in the %PID appears to reflect at least moderately significant differences representing “much better,” “much improved,” or “meaningful” decreases in pain [24–27]. Therefore, the range of the PID was 1.13–1.74, and the average %PID of 29.0% observed in the moderate pain group in this study may indicate that the pain relief provided to patients with moderate pain was insufficient. Similar undesirable findings have been reported at other hospitals [29,30].

Additionally, more significant changes in the mean PID (3.09) and mean %PID (38.5%) were observed among patients with severe pain. Although the results did not comply with the Initiative on Methods, Measurement, and Pain Assessment in Clinical Trials (IMMPACT) recommendations [2], according to which a decrease in the PID of ≥ 4 points or in the %PID of ≥ 50% appears to represent a substantial improvement, the severe pain group showed a meaningful decrease in pain and satisfied the criteria more closely than the moderate pain group. A prominent effect on pain reduction was found among the patients with severe pain in this study.

All cancer patients experience pain during repeated hospitalizations; however, continuous and effective pain management strategies can reduce the severity of their pain [31]. In Fig 2, we showed that the PID and %PID values of the WPI ≥ 7 group were higher than those of the 4 ≤ WPI ≤ 6 group from the 1st to the 18th hospitalizations. Our data revealed that pain reduction among patients with severe pain was better than that in patients with moderate pain during each hospitalization, possibly because clinicians always applied aggressive interventions to treat severe pain, but not moderate pain. Therefore, more aggressive and immediate pain management is necessary for patients with moderate pain. We will continue to promote pain management techniques for moderate pain patients to achieve significant improvements that are consistent with IMMPACT recommendations.

In this study, 12,856 pain scores were included in the moderate pain group and 10,095 pain scores were included in the severe pain group from the 1st to the 18th hospitalizations. An individual’s pain score was measured using the NRS, FPS or FLACC Behavioral Tool; this approach is also known as a patient-reported outcome. Patient-reported pain intensities can be immediately scored and entered into the NIS using electronic devices at each patient’s bedside. This electronic data recording system can improve documentation by eliminating transcription errors, thereby facilitating the subsequent retrieval and analysis of the data, permitting complex analyses of the relationships between the scores and their timing and allowing tracking by clinicians over time to guide patient care [3,13,30]. Using the NIS, collected pain intensity data can be integrated into the ongoing process of pain measurement, and immediate comprehensive information can be provided for pain assessments [13].

Based on the patient-reported pain score at different time points, this study showed that the ranges of the PID and %PID were 1.13–1.74 and 21.8%-33.9%, respectively, in the moderate pain group; the ranges of the PID and %PID were 2.84–3.36 and 35.5%-42.5%, respectively, in the severe pain group from the 1st to the 18th hospitalizations. We examined the PIDs during each hospitalization from a time-series perspective among cancer patients with moderate or severe pain who were repeatedly hospitalized. Previous studies have evaluated pain phenomena during a single hospitalization [17,20]. Since cancer patients often require repeated hospitalizations and pain is associated with cancer progression and survival [10,32], a long-term time-series analysis of the hospitalizations of each patient should be performed. More severe pain is independently associated with shorter overall survival [32]. Measurements of pain using the same scoring method at different time points provide data that can be used by clinicians to observe variations in pain reduction over time [31]. When clinicians observe more severe pain in a patient compared with their previous hospitalizations, the clinicians should be alerted that the patient’s condition is worsening. Thus, we can share this information with the patients early to allow them enough time to say goodbye peacefully and to express their love, apologies, and gratitude to important people in their lives.

In addition, our study evaluated the entire population of cancer patients during each hospitalization and collected data over three years at an academic medical center, thereby addressing the weaknesses of previous studies [20,33], which included evaluations from only a single medical oncology unit and evaluations performed over only six months. Although a study of hospital-wide pain scores has been previously published [30], that study examined pain scores over the span of only one year. Thus, the collection of pain scores over a three-year period in our study provides more comprehensive information than the aforementioned studies.

Another important aspect of this study was the implementation of the NIS. Most pain-related studies rely on manual documentation of pain assessments and retrospective chart review [33–35], and thus, the literature on pain assessment and management among hospitalized cancer patients remains limited [36]. The electronic NIS is increasingly viewed as an essential tool for quality assurance and improvement in a variety of care settings. Through this electronic system, clinicians can maintain patient continuity with a primary care team, document and compile patient information, use this information to manage and coordinate care delivered in a primary care practice, and share patient information across practices and settings to provide support or exchange information during transitions [31,37]. Therefore, in this study, we used a NIS to collect pain scores to link pain assessments over time and then evaluated the PID and %PID during the 3-year period among hospitalized cancer patients.

This study has several limitations. One limitation is that this study was conducted using a cancer patient cohort that was heterogeneous with respect to the clinical disease stage, age and cancer type. Additionally, the generalizability of the findings may be limited because this study was conducted at a single academic medical center. However, this study design can be extended to other patient groups and replicated at other institutions to validate the results.

## Conclusions

In conclusion, this investigation represents the first hospital-based study that used an electronic database to evaluate the differences in pain intensity among cancer patients in Taiwan. All cancer patients in our medical center were included in the study. Using an NIS, we effectively collected pain intensity scores to conduct a hospital-wide, long-term study. During each hospitalization, the cancer patients with moderate and severe pain at this medical center exhibited pain reductions of approximately 30% and 40%, respectively, and a prominent effect on pain reduction was observed in patients with severe pain. Based on these primary and positive findings, we can provide feedback and empower clinicians to be more committed and aggressive with regard to pain management in patients with severe pain to achieve a marked analgesic effect. The %PID outcomes can be considered available indicators for evaluating pain reduction, and in the future, we can implement the %PID formula in the NIS to alert clinicians for early detection of the effectiveness and appropriateness of cancer pain management.

## Supporting information

S1 File Pain score. (PDF)
