# Supplementary material for: Evaluation of the pain intensity differences among hospitalized cancer patients based on a nursing information system
Source: PLoS One. 2019 Sep 25;14(9):e0222516. doi: 10.1371/journal.pone.0222516 (PMC6760775; doi:10.1371/journal.pone.0222516)
Supplement: S1 File — (PDF) [file pone.0222516.s001.pdf]

idpadmd	SER_NUM_first	PBIRTHDT_first	PSEX_first	PADMDT_first	DEGREE_max_r
493320131007	17985431	19340903	1	20131007	6
81630 20121207	17050346	19290916	1	20121207	4
98044 20120819	16711639	19290722	1	20120819	6
111024 20120227	16185561	19260212	1	20120227	5
116392 20110329	15216320	19211201	1	20110329	5
116949 20110223	15117672	19250710	1	20110223	5
121722 20110608	15423685	19250408	1	20110608	5
13698120131219	18221937	19251028	1	20131219	5
141924 20120523	16446145	19140825	1	20120523	4
146598 20101227	14958220	19330318	1	20101227	5
147091 20120714	16606261	19220822	1	20120714	4
16764620130130	17208351	19300101	1	20130130	5
178563 20120910	16777032	19451103	2	20120910	5
178961 20110518	15366656	19310505	1	20110518	6
18290120130226	17281593	19280815	1	20130226	5
183584 20110516	15358286	19261020	1	20110516	5
18762220131007	17987429	19310303	1	20131007	4
204308 20120603	16474661	19330721	2	20120603	5
22925420130327	17376141	19290906	1	20130327	5
23912320131017	18016812	19300118	1	20131017	4
24228420130702	17675686	19241010	1	20130702	6
261198 20121210	17053551	19381002	1	20121210	5
281958 20120405	16302660	19200913	1	20120405	5
29213720130328	17378562	19411228	2	20130328	4
297938 20110108	14993698	19320127	1	20110108	5
29865720130104	17132689	19331125	1	20130104	6
312538 20110712	15526589	19260406	1	20110712	5
313779 20110815	15624930	19320108	1	20110815	4
32646520130425	17467098	19350218	2	20130425	5
343511 20110507	15334399	19270110	1	20110507	5
34405820130712	17714608	19540413	1	20130712	4
411530 20120721	16626222	19230120	1	20120721	6
429152 20111213	15974281	19301224	1	20111213	6
458639 20120526	16454302	19281020	1	20120526	5
47711220130704	17683188	19240310	1	20130704	4
47718920130527	17562695	19320529	1	20130527	5
485085 20120625	16538560	19190808	1	20120625	5
491883 20110509	15338941	19271020	1	20110509	4
509375 20121005	16854008	19280807	1	20121005	4
53433820130912	17909197	19250414	1	20130912	6
53913920131127	18147717	19280525	1	20131127	4
550992 20111118	15899992	19140802	1	20111118	5
56850320130601	17577216	19261203	1	20130601	4
569813 20110322	15199271	19260920	1	20110322	5
584996 20110618	15455249	19310714	1	20110618	5
597331 20110504	15324646	19261220	1	20110504	5
63143620130306	17312059	19310319	1	20130306	4
636248 20111205	15946243	19250708	1	20111205	6
67413520131202	18159692	19221230	1	20131202	6
67958320130714	17716104	19470101	2	20130714	6
704358 20110109	14994390	19260525	2	20110109	6
742121 20121014	16878260	19600911	1	20121014	5
74896920131124	18137814	19250815	2	20131124	5
771584 20111201	15935848	19620302	2	20111201	5

780267	20110220	15106173	19400422	2	20110220	6
783733	20130212	17243726	19270705	1	20130212	6
819909	20120504	16391974	19250623	1	20120504	4
856622	20120204	16119137	19290801	1	20120204	5
862044	20110516	15359497	19600130	2	20110516	5
889758	20110907	15692414	19270622	1	20110907	5
909160	20120611	16502593	19230311	1	20120611	5
931919	20111118	15900855	19281113	1	20111118	5
941355	20121110	16961191	19330219	2	20121110	4
941753	20130702	17677153	19520131	1	20130702	4
945153	20120219	16164386	19310919	1	20120219	5
962129	20120627	16548118	19260421	1	20120627	6
964374	20120504	16392139	19210905	1	20120504	4
985999	20130803	17780237	19490908	1	20130803	6
992256	20130616	17625683	19631112	2	20130616	5
992267	20130102	17123043	19301215	1	20130102	6
993511	20110423	15291134	19260110	1	20110423	6
1003469	20120201	16107549	19300506	1	20120201	6
1024482	20110121	15030871	19350929	2	20110121	5
1028166	20120929	16831236	19261020	1	20120929	5
1028586	20130504	17495451	19311215	1	20130504	6
1048108	20120405	16303710	19330515	1	20120405	6
1049849	20130209	17242127	19250909	1	20130209	5
1055807	20110209	15069832	19331210	1	20110209	6
1064933	20111116	15894340	19390817	2	20111116	5
1075576	20111102	15851532	19361102	1	20111102	6
1137800	20120430	16372311	19550701	2	20120430	6
1155017	20120526	16453478	19240109	1	20120526	6
1157477	20110415	15270430	19310815	1	20110415	5
1171342	20111004	15765230	19230820	1	20111004	4
1178887	20130102	17121691	19670525	2	20130102	6
1195682	20110613	15437388	19670603	1	20110613	5
1228440	20120306	16214572	19310908	1	20120306	5
1272613	20101229	14963978	19290205	1	20101229	4
1290206	20130916	17921826	19200508	1	20130916	5
1298653	20110608	15422805	19501124	2	20110608	6
1298993	20110825	15654335	19281014	1	20110825	4
1324012	20130404	17402740	19241001	1	20130404	4
1340701	20120527	16454760	19351205	1	20120527	4
1342796	20120217	16161313	19281221	1	20120217	4
1384798	20130621	17644264	19240506	2	20130621	6
1387902	20120411	16320201	19470624	2	20120411	6
1388518	20110528	15391417	19250801	2	20110528	5
1400346	20110227	15126045	19490410	2	20110227	5
1408828	20120623	16537453	19250224	1	20120623	5
1424846	20130226	17281957	19470628	2	20130226	6
1443614	20110424	15292514	19210613	1	20110424	5
1445665	20111024	15822349	19510223	2	20111024	6
1455283	20120620	16530951	19280622	1	20120620	6
1475292	20110502	15314131	19501018	2	20110502	6
1484215	20120807	16674316	19470520	2	20120807	4
1488535	20111002	15756559	19320202	1	20111002	5
1510890	20131122	18135848	19250610	2	20131122	6
1515157	20101226	14954770	19541212	2	20101226	4
1533171	20111102	15848953	19290505	2	20111102	6

1542467	20120423	16355030	19250926	1	20120423	6
1556861	20111004	15763893	19330614	1	20111004	4
1590085	20130124	17193532	19660723	2	20130124	6
1597213	20120811	16690119	19410717	1	20120811	5
1609376	20120829	16738337	19490430	2	20120829	6
1615981	20120310	16227435	19420105	2	20120310	4
1635832	20101221	14943802	19210203	1	20101221	5
1652046	20120131	16101347	19260613	1	20120131	6
1677550	20111110	15877356	19280511	2	20111110	6
1685649	20120716	16608714	19200806	1	20120716	5
1687327	20130127	17200250	19290318	2	20130127	6
1689538	20111107	15864934	19221226	1	20111107	5
1689583	20130701	17667648	19370316	1	20130701	5
1689845	20130825	17847512	19290424	1	20130825	4
1696577	20130906	17891441	19310919	2	20130906	5
1735553	20131110	18095052	19220627	1	20131110	5
1748454	20131001	17962189	19240703	1	20131001	5
1769693	20110417	15272707	19430830	2	20110417	6
1788585	20110118	15021772	19270219	1	20110118	5
1789555	20131001	17962383	19640513	2	20131001	4
1791931	20131203	18166334	19360103	2	20131203	5
1804508	20120401	16285182	19290117	1	20120401	4
1822351	20130512	17519049	19330919	1	20130512	5
1822602	20110918	15719099	19360408	1	20110918	5
1828848	20110823	15647667	19401015	2	20110823	6
1835854	20110816	15629113	19281114	1	20110816	5
1849429	20120630	16556326	19410721	2	20120630	5
1864193	20101229	14964113	19261014	1	20101229	5
1865129	20131209	18190044	19300308	1	20131209	4
1876353	20110713	15530745	19251010	1	20110713	6
1880279	20121225	17104436	19270116	1	20121225	6
1888262	20121025	16912419	19260812	1	20121025	5
1889016	20120319	16252925	19450809	1	20120319	4
1891550	20121019	16896837	19301011	1	20121019	4
1894742	20120717	16614436	19510225	1	20120717	4
1904796	20131202	18161077	19300103	1	20131202	4
1914289	20130512	17519125	19440305	2	20130512	6
1947144	20110219	15105834	19220729	1	20110219	4
1948749	20110420	15284063	19511203	1	20110420	5
1954616	20130719	17735640	19280805	1	20130719	6
1967528	20110531	15397929	19550123	1	20110531	5
1981437	20121116	16983412	19280525	1	20121116	6
1987560	20110504	15323505	19300205	2	20110504	4
1988074	20130923	17936755	19201122	1	20130923	4
2005847	20130729	17760672	19290802	2	20130729	5
2006486	20120210	16139168	19640528	1	20120210	5
2018271	20110815	15626136	19250415	1	20110815	5
2037209	20131202	18162843	19271106	1	20131202	5
2039909	20130228	17287803	19300801	1	20130228	5
2044679	20130823	17844271	19260624	1	20130823	4
2046493	20121011	16872316	19280714	1	20121011	6
2059054	20120604	16479865	19260819	1	20120604	6
2076564	20110718	15542357	19540708	1	20110718	4
2095058	20111224	16004101	19301011	1	20111224	6
2095923	20131006	17981962	19700430	1	20131006	6

2106801	20120812	16690627	19431225	1	20120812	6
2111719	20110211	15080310	19311215	1	20110211	6
2130101	20120613	16510478	19300501	1	20120613	4
2141380	20110817	15632557	19380627	2	20110817	4
2148063	20111109	15873258	19160802	1	20111109	6
2184136	20130407	17407203	19300520	1	20130407	6
2212626	20120430	16371953	19321027	1	20120430	4
2214600	20110424	15292553	19500104	2	20110424	6
2215896	20130227	17285237	19300528	1	20130227	6
2224411	20130927	17951490	19301010	1	20130927	5
2230899	20110315	15180473	19251229	1	20110315	6
2251947	20110418	15275204	19491107	2	20110418	4
2260017	20120923	16814427	19701204	2	20120923	6
2288020	20130716	17723433	19260902	1	20130716	5
2307484	20110428	15304159	19390612	2	20110428	5
2351839	20110810	15610911	19271029	1	20110810	5
2382481	20130805	17785928	19230511	1	20130805	6
2386472	20110914	15710174	19340120	1	20110914	6
2386950	20110102	14970035	19580201	1	20110102	6
2391051	20111214	15977645	19391130	2	20111214	5
2394050	20110417	15272645	19490702	2	20110417	6
2414520	20110224	15121199	19290216	1	20110224	5
2418293	20110718	15542963	19330223	1	20110718	4
2439089	20120201	16104517	19700820	1	20120201	4
2452439	20110122	15033179	19320312	2	20110122	4
2453976	20130120	17180529	19301127	1	20130120	6
2470475	20110802	15585831	19440608	1	20110802	5
2481632	20131029	18051032	19310405	1	20131029	5
2489692	20130403	17400496	19300923	1	20130403	5
2494715	20120313	16237085	19250205	1	20120313	5
2497576	20120219	16164499	19290629	1	20120219	6
2511344	20110415	15268820	19320310	1	20110415	5
2520652	20111116	15894496	19290408	1	20111116	4
2528769	20110201	15056162	19370103	2	20110201	4
2537680	20101224	14952240	19280906	1	20101224	5
2540230	20130118	17176707	19300524	1	20130118	4
2552536	20121108	16954909	19400324	2	20121108	4
2587782	20120803	16661840	19500928	2	20120803	5
2623087	20110610	15432392	19290228	2	20110610	5
2624353	20120401	16285323	19300222	2	20120401	4
2646062	20110803	15589984	19340222	2	20110803	5
2693869	20121202	17024987	19400201	2	20121202	6
2694624	20131018	18021057	19430201	2	20131018	6
2710841	20121003	16847076	19220729	1	20121003	5
2727971	20130710	17706870	19330410	1	20130710	5
2728098	20111101	15844790	19271019	1	20111101	4
2732403	20130920	17931706	19290920	1	20130920	6
2753506	20110330	15218863	19251123	1	20110330	6
2825743	20110210	15078024	19340815	1	20110210	5
2863652	20110317	15184349	19350105	1	20110317	4
2904816	20130317	17345603	19560318	2	20130317	6
2960067	20110516	15358438	19311229	1	20110516	4
2963124	20130415	17434974	19290524	2	20130415	5
2985184	20120829	16738594	19470203	2	20120829	6
2987044	20130521	17546772	19400802	2	20130521	4

3015676	20111129	15925993	19310705	1	20111129	6
3031309	20130611	17613216	19500314	2	20130611	6
3080179	20120307	16218119	19590820	1	20120307	6
3096886	20110731	15574944	19470815	2	20110731	4
3126156	20130609	17605348	19240928	1	20130609	5
3178265	20110209	15072368	19451120	2	20110209	4
3182294	20130102	17121407	19241201	1	20130102	4
3250848	20110715	15536424	19321001	2	20110715	4
3254408	20130123	17191483	19340908	2	20130123	6
3254793	20110328	15213231	19300420	1	20110328	4
3266873	20120720	16623511	19490209	1	20120720	6
3270517	20110823	15646799	19280813	1	20110823	4
3273210	20130502	17488582	19371229	2	20130502	6
3290628	20110830	15663279	19320123	1	20110830	4
3296682	20130602	17578464	19480515	1	20130602	6
3306363	20120827	16732055	19710317	2	20120827	4
3316469	20120305	16208302	19520827	2	20120305	4
3321800	20110419	15280134	19410926	2	20110419	5
3334427	20111104	15860114	19490323	2	20111104	6
3334756	20110808	15603496	19600320	1	20110808	6
3339091	20120525	16452053	19260222	1	20120525	5
3385544	20131202	18162738	19520410	1	20131202	5
3403143	20130902	17870375	19490103	2	20130902	4
3406517	20111008	15781910	19300820	1	20111008	4
3412213	20110811	15616867	19250111	1	20110811	5
3454066	20111009	15782374	19691003	1	20111009	5
3461538	20120417	16340692	19310920	1	20120417	4
3514792	20110225	15123495	19620307	2	20110225	5
3543553	20120202	16110944	19261230	1	20120202	6
3559146	20110214	15087104	19491013	1	20110214	5
3562514	20130811	17806594	19551128	1	20130811	5
3570669	20120823	16723291	19710809	1	20120823	4
3633065	20120924	16817328	19320810	1	20120924	5
3634182	20131123	18137322	19230108	1	20131123	4
3658013	20130923	17934781	19380221	2	20130923	6
3660320	20121224	17100172	19380729	2	20121224	5
3678168	20111129	15927952	19510926	1	20111129	4
3684239	20120218	16163800	19290102	1	20120218	5
3717666	20110425	15295366	19291124	2	20110425	4
3726178	20110323	15202127	19311205	1	20110323	5
3743928	20120511	16413814	19481009	2	20120511	6
3752485	20120508	16402306	19570416	2	20120508	5
3754903	20110731	15574993	19290815	1	20110731	5
3760154	20111107	15866372	19291229	1	20111107	5
3763722	20110925	15738363	19230207	1	20110925	6
3765126	20110623	15469397	19240303	1	20110623	5
3788792	20130223	17273457	19290104	1	20130223	6
3812326	20120925	16821194	19430708	2	20120925	6
3834795	20120207	16129078	19261102	1	20120207	6
3839187	20130623	17647341	19240805	2	20130623	4
3853869	20110810	15612942	19210505	1	20110810	6
3867490	20130829	17860870	19381005	2	20130829	4
3877676	20110325	15208166	19280319	1	20110325	4
3884422	20120202	16109992	19460720	2	20120202	4
3899385	20110525	15384331	19570318	2	20110525	5

391273020121127		17013460	19300203	2	20121127	5
392713720131012		18002243	19240616	1	20131012	5
3962718	20110509	15339562	19320105	2	20110509	5
3968705	20110621	15462602	19421115	2	20110621	6
397551720130523		17554712	19150607	1	20130523	6
397719320130219		17258362	19250112	1	20130219	4
3980823	20110420	15283646	19430109	2	20110420	5
3982932	20120904	16757115	19261004	1	20120904	6
3994590	20120129	16094662	19220610	1	20120129	4
4011761	20111028	15835476	19291228	1	20111028	6
4039310	20110923	15735651	19340910	2	20110923	6
4048242	20121121	16996922	19390425	1	20121121	5
4050366	20121219	17088511	19490324	2	20121219	6
4050479	20110614	15443125	19511203	2	20110614	5
4061896	20121101	16931353	19331217	2	20121101	6
4072075	20120528	16457906	19291001	2	20120528	4
4088975	20110822	15643475	19500314	2	20110822	4
4096860	20120308	16223067	19431231	2	20120308	4
412259220130718		17732291	19311101	1	20130718	4
4131606	20111110	15876503	19670228	2	20111110	4
4145942	20110813	15621679	19501209	1	20110813	5
415504820130623		17646944	19290519	2	20130623	6
4181231	20120808	16678618	19240515	1	20120808	4
419900220131120		18127048	19541117	2	20131120	5
4202997	20110926	15741704	19550806	2	20110926	6
4218762	20121111	16963652	19540217	2	20121111	4
424117220130829		17859328	19420802	2	20130829	5
424778320130521		17548444	19410202	2	20130521	4
4274388	20110407	15240385	19740223	2	20110407	5
427798920130505		17495994	19291203	1	20130505	4
430212920130302		17296336	19260404	2	20130302	4
4319000	20110328	15213197	19390512	1	20110328	6
431981720130304		17302313	19350321	1	20130304	6
4360489	20120108	16047437	19270508	1	20120108	5
4371544	20110228	15128176	19530221	1	20110228	4
4400639	20111230	16020804	19470115	2	20111230	4
4411750	20120517	16431657	19420715	2	20120517	5
4422984	20120710	16592980	19240505	1	20120710	5
4460086	20110906	15687029	19240715	2	20110906	5
4460962	20110324	15205403	19380920	2	20110324	5
446651720130528		17567044	19520902	2	20130528	5
446722520130308		17320439	19260406	1	20130308	5
448537420131130		18156001	19280527	1	20131130	5
4509164	20111021	15818275	19480615	2	20111021	5
4514209	20120218	16163902	19311029	1	20120218	5
452217220130808		17799027	19280123	1	20130808	5
4571293	20111002	15756390	19250811	1	20111002	6
458105920130716		17724607	19320103	1	20130716	6
460286220130501		17479095	19500225	1	20130501	4
461193220130813		17815444	19461003	1	20130813	5
4627310	20111104	15858923	19250212	1	20111104	6
463419920130723		17745134	19410111	1	20130723	4
4638613	20110220	15106355	19570215	2	20110220	5
464215320130905		17884436	19370401	2	20130905	5
466492020131210		18192896	19220904	1	20131210	5

4707395	20120730	16648432	19311008	1	20120730	5
4727860	20111205	15945379	19400125	2	20111205	5
4729742	20130310	17324200	19501218	2	20130310	6
4763739	20130314	17339692	19161009	1	20130314	6
4764210	20121121	16997059	19430428	1	20121121	6
4767786	20110518	15364306	19491214	1	20110518	4
4782245	20110315	15180496	19451204	2	20110315	4
4785948	20110922	15732598	19280102	2	20110922	6
4788516	20130715	17719488	19490402	1	20130715	6
4791202	20111130	15930926	19341017	2	20111130	5
4800593	20131207	18184906	19270104	1	20131207	4
4807630	20121001	16837408	19470901	1	20121001	4
4814793	20130412	17428119	19490912	1	20130412	5
4818171	20110813	15621791	19270914	1	20110813	5
4818568	20111019	15812068	19640730	2	20111019	6
4865592	20110920	15724721	19281127	2	20110920	6
4869016	20131119	18125826	19260614	1	20131119	5
4883469	20130406	17406674	19260611	1	20130406	6
4899176	20120418	16342582	19490301	2	20120418	5
4907306	20130420	17452647	19200625	1	20130420	5
4908774	20111101	15846091	19550910	2	20111101	4
4915257	20120514	16417834	19390715	2	20120514	6
4924076	20120617	16520860	19400513	1	20120617	6
4930512	20120911	16781564	19430829	1	20120911	5
4952903	20131020	18025808	19540522	2	20131020	6
4953440	20120718	16618020	19591114	1	20120718	6
4956052	20110105	14981762	19390723	1	20110105	6
4962827	20120310	16229010	19410507	1	20120310	6
4984741	20110410	15249996	19300816	1	20110410	5
4991906	20121009	16867239	19401224	1	20121009	4
5003361	20111011	15786420	19481108	1	20111011	6
5007749	20120925	16821492	19300604	1	20120925	4
5024453	20111225	16005718	19510407	1	20111225	4
5037343	20111118	15900372	19220928	2	20111118	5
5037672	20130429	17474887	19251023	2	20130429	5
5052346	20120312	16233948	19460324	1	20120312	6
5057216	20120912	16784452	19370916	2	20120912	6
5075069	20110806	15600252	19390916	1	20110806	5
5106161	20130124	17193873	19250219	2	20130124	5
5112776	20131028	18049286	19261221	2	20131028	5
5120478	20121009	16866009	19551120	2	20121009	6
5153877	20120527	16454582	19271224	1	20120527	4
5166461	20110910	15701501	19301123	1	20110910	4
5170365	20120923	16814283	19471116	1	20120923	4
5192063	20121031	16925198	19280505	1	20121031	6
5225229	20111003	15760025	19211009	1	20111003	6
5236395	20110223	15116534	19290821	1	20110223	6
5251923	20130217	17250494	19220201	1	20130217	5
5259712	20111127	15921808	19280515	2	20111127	5
5267323	20130515	17531846	19450801	2	20130515	6
5286420	20111017	15802922	19520902	1	20111017	4
5298033	20120325	16268393	19360511	2	20120325	5
5302841	20120125	16089988	19230707	1	20120125	6
5303311	20130728	17757719	19321130	1	20130728	6
5319744	20130501	17482216	19520725	2	20130501	5

5333857	20110212	15083890	19700627	1	20110212	5
5336345	20120503	16388888	19520825	1	20120503	4
5337199	20110224	15119809	19311228	1	20110224	4
540215320130209		17242063	19351101	2	20130209	5
5407625	20110625	15473354	19260122	1	20110625	4
5434899	20110320	15192410	19480312	2	20110320	4
544886220131117		18118029	19300822	1	20131117	4
5484004	20120507	16398582	19241115	1	20120507	6
5497916	20111220	15994126	19501013	2	20111220	5
5505862	20120209	16135797	19570215	2	20120209	4
5512094	20110608	15423000	19281020	1	20110608	6
5542381	20120422	16351717	19260506	2	20120422	6
5570058	20110624	15472069	19490925	2	20110624	6
5631754	20121215	17076533	19571015	2	20121215	6
5631925	20120814	16699000	19250110	2	20120814	6
5641736	20101214	14924570	19341213	2	20101214	4
5641883	20120609	16498881	19441203	2	20120609	5
566404020130825		17847473	19270415	1	20130825	6
5673438	20121209	17053199	19700601	2	20121209	6
5684684	20120919	16804632	19560408	2	20120919	5
5742632	20120131	16100519	19430620	2	20120131	6
579151520130923		17937071	19290806	1	20130923	4
5793555	20111031	15840047	19480320	2	20111031	5
5796349	20120717	16613416	19430827	2	20120717	5
5815714	20120804	16664534	19240425	1	20120804	6
5825558	20120305	16207458	19310629	1	20120305	4
5841350	20111211	15966452	19330919	1	20111211	6
586364920130920		17930956	19191217	1	20130920	4
587919620131120		18129124	19270816	2	20131120	6
587974320130724		17749332	19540608	2	20130724	6
588983820130305		17307915	19521215	2	20130305	4
5912072	20111017	15804828	19560705	1	20111017	5
5930983	20121023	16905676	19520815	2	20121023	6
5938454	20120402	16291726	19240813	1	20120402	5
5947079	20111208	15958398	19580809	2	20111208	4
596917520130430		17477817	19600929	2	20130430	6
597761720131021		18028657	19490723	2	20131021	4
5979624	20120713	16603520	19220515	1	20120713	4
5982332	20110211	15082083	19240921	1	20110211	5
6022851	20121014	16878147	19490627	1	20121014	4
603049520130604		17585585	19490707	1	20130604	4
6056571	20120323	16266660	19321208	1	20120323	5
6064375	20120315	16244034	19540926	2	20120315	4
607553220130124		17193938	19301018	1	20130124	5
6099372	20110527	15389605	19200105	1	20110527	5
6120372	20111227	16012405	19290905	2	20111227	6
6134992	20111029	15837483	19500115	2	20111029	6
613569920130726		17754419	19550304	1	20130726	5
614337920130220		17261497	19260929	1	20130220	5
6150294	20120311	16229602	19400130	2	20120311	5
6153555	20110208	15067169	19530210	1	20110208	4
6206515	20111023	15820389	19290606	1	20111023	4
621701220130522		17551868	19560207	2	20130522	4
6231067	20110616	15450385	19550318	2	20110616	6
6243476	20121104	16938855	19300502	1	20121104	4

626983620130618		17633628	19320101	2	20130618	4
6288897	20110423	15290781	19530320	1	20110423	4
632526420130328		17379154	19310228	1	20130328	4
6382907	20110608	15423906	19440507	2	20110608	4
638384220131130		18156050	19201010	1	20131130	6
6397279	20121125	17006506	19180115	2	20121125	5
6399877	20120207	16127901	19300906	1	20120207	6
6400415	20110510	15343688	19541130	2	20110510	5
6409558	20120213	16147720	19330211	2	20120213	4
6409785	20111029	15837428	19280314	2	20111029	5
6430075	20120224	16178997	19560726	1	20120224	4
6432979	20120717	16614690	19740517	2	20120717	6
6437656	20121202	17025074	19431028	2	20121202	4
6464240	20110914	15708700	19260323	1	20110914	5
6484679	20120406	16307276	19260227	1	20120406	4
6510696	20111027	15832939	19310328	2	20111027	5
6536998	20120722	16626901	19510514	1	20120722	6
6556714	20110216	15096240	19451002	2	20110216	5
6576303	20121112	16965687	19570228	2	20121112	5
658488920130504		17493845	19220510	2	20130504	4
6600379	20110731	15575062	19311015	1	20110731	4
6620899	20111018	15807724	19291029	1	20111018	6
6627323	20110607	15417952	19300806	1	20110607	5
6630804	20120701	16556979	19500218	2	20120701	5
666627920130526		17560275	19500907	1	20130526	4
6674642	20121119	16989985	19240117	1	20121119	4
6705620	20110918	15719375	19430105	1	20110918	4
6725800	20120329	16280721	19490710	2	20120329	6
672681420130714		17716154	19591002	1	20130714	6
6735995	20110331	15220643	19270824	1	20110331	4
6755415	20110521	15374440	19471219	1	20110521	5
6766967	20101225	14954246	19560208	2	20101225	6
6772641	20120629	16554130	19311125	1	20120629	4
6776201	20110624	15469911	19400407	1	20110624	5
6777511	20110522	15375532	19310112	1	20110522	4
6787504	20120810	16688209	19670124	2	20120810	5
6802159	20110214	15086260	19530504	2	20110214	6
680896420130527		17564047	19560620	2	20130527	6
6828780	20110217	15100300	19240126	1	20110217	5
6866519	20120709	16586249	19290223	1	20120709	6
6875407	20110302	15139512	19560810	2	20110302	6
694256920131113		18108379	19270115	1	20131113	6
6977635	20121011	16871184	19750723	1	20121011	6
6986238	20120223	16177346	19510315	2	20120223	4
6987913	20110916	15716613	19430124	1	20110916	6
6998330	20110621	15461790	19290330	1	20110621	6
7012580	20111026	15830250	19390827	1	20111026	5
7019387	20110223	15117201	19590201	2	20110223	4
7029336	20120920	16808085	19230715	2	20120920	6
7032011	20120826	16729915	19250123	1	20120826	4
705013720130226		17280852	19460123	2	20130226	5
7078684	20111101	15844807	19361108	1	20111101	4
7081972	20120626	16544541	19250715	1	20120626	4
7090144	20120222	16174255	19250418	1	20120222	6
7096788	20110212	15084075	19480816	1	20110212	4

7108738	20110508	15335096	19261225	1	20110508	6
7140116	20111021	15816078	19371205	2	20111021	5
714276920130325		17368927	19441117	2	20130325	4
7143046	20120912	16785474	19260908	1	20120912	5
7162427	20111227	16011436	19230219	1	20111227	4
718527520130117		17174286	19320117	2	20130117	5
719388620130511		17518835	19550415	2	20130511	6
7226688	20120503	16386041	19490913	2	20120503	5
7255290	20110506	15332413	19380113	2	20110506	5
7282351	20120926	16824707	19310828	1	20120926	6
7284879	20110419	15278939	19521015	1	20110419	4
7289216	20111101	15846598	19401117	2	20111101	4
7309742	20110831	15666593	19301124	1	20110831	5
731083020130806		17791319	19411113	2	20130806	6
7363204	20110906	15688230	19600301	2	20110906	5
7366134	20110404	15231748	19500102	2	20110404	6
7368118	20110522	15375467	19600121	1	20110522	4
737175720130726		17753978	19250701	1	20130726	6
7389404	20120305	16209341	19240815	2	20120305	6
7399000	20120923	16814149	19530818	2	20120923	4
7404446	20110701	15489890	19270514	2	20110701	6
740557420130417		17443861	19500101	2	20130417	5
7444760	20120426	16364036	19550101	1	20120426	6
7497623	20121113	16971719	19270302	1	20121113	5
750248920130423		17459335	19460214	2	20130423	5
7519893	20110510	15342308	19540813	1	20110510	5
7534512	20110923	15735862	19680315	2	20110923	5
754164220130528		17565518	19520228	1	20130528	6
7570618	20121018	16892975	19251007	1	20121018	5
7589493	20120527	16454691	19471205	1	20120527	5
7590081	20120918	16801918	19481011	2	20120918	4
7641227	20110125	15040869	19521101	2	20110125	6
7657149	20121122	16999903	19191018	1	20121122	5
766252420130428		17471579	19251115	1	20130428	4
7676735	20120302	16198596	19260606	1	20120302	6
767761420130623		17647082	19560808	1	20130623	6
7681110	20120624	16537963	19451212	2	20120624	6
7681609	20111107	15866245	19470901	1	20111107	5
7695525	20110501	15309391	19510818	1	20110501	5
7715733	20110729	15572588	19410306	2	20110729	6
7717773	20110222	15113960	19451201	2	20110222	6
7730612	20111219	15989497	19250917	1	20111219	5
7778021	20120401	16285358	19601101	1	20120401	5
780800620130705		17689287	19470501	1	20130705	6
7826406	20111012	15789143	19251213	1	20111012	5
7836319	20120318	16250578	19720518	1	20120318	6
7855358	20121001	16834795	19560609	1	20121001	5
786966120130520		17543168	19251106	2	20130520	6
7880093	20110924	15737877	19501028	1	20110924	4
790752020130202		17219879	19350706	2	20130202	6
7910330	20110220	15106357	19401229	2	20110220	6
7918118	20120301	16196622	19510301	2	20120301	6
7951555	20110111	15001717	19180418	1	20110111	6
7966383	20120423	16354356	19500423	1	20120423	5
798985720130710		17705552	19300517	1	20130710	4

8036768	20110312	15172218	19541129	1	20110312	5
8053267	20130416	17438412	19521114	2	20130416	6
8053438	20101230	14966634	19431121	2	20101230	4
8068277	20120718	16617863	19171005	2	20120718	5
8082551	20130913	17915154	19350601	1	20130913	5
8137904	20110808	15604573	19300814	2	20110808	5
8140612	20121031	16924647	19270412	1	20121031	6
8149982	20121107	16952713	19291015	1	20121107	4
8158803	20121015	16881326	19460703	2	20121015	6
8166981	20120501	16377007	19301221	1	20120501	6
8174478	20120419	16347068	19250309	1	20120419	6
8178152	20110302	15136103	19250823	1	20110302	5
8183560	20111028	15835412	19270201	1	20111028	5
8220211	20121017	16889419	19410805	2	20121017	6
8234682	20120303	16203360	19340324	1	20120303	4
8244119	20110526	15386982	19230212	1	20110526	6
8261754	20111121	15907171	19390128	2	20111121	4
8297787	20110201	15054849	19371111	2	20110201	4
8308654	20120724	16633648	19500415	1	20120724	4
8313539	20101119	14850779	19490105	1	20101119	4
8321195	20130324	17365848	19530820	2	20130324	4
8331677	20130108	17145371	19281003	1	20130108	4
8354129	20120420	16349205	19390830	2	20120420	6
8368067	20130830	17863151	19300113	1	20130830	5
8368498	20130907	17893304	19560312	1	20130907	5
8401885	20120101	16022447	19450101	1	20120101	5
8419930	20130901	17864974	19240101	1	20130901	5
8427154	20110520	15373432	19251004	1	20110520	5
8438468	20110518	15366915	19480301	1	20110518	5
8467263	20130719	17735621	19310707	1	20130719	6
8494891	20121114	16973840	19460118	1	20121114	4
8498746	20130923	17938273	19420108	2	20130923	5
8509248	20130322	17362702	19340808	1	20130322	6
8515784	20120426	16364949	19440728	2	20120426	5
8526269	20130302	17296263	19590428	2	20130302	5
8539035	20130114	17162859	19651031	2	20130114	6
8616124	20131017	18018620	19540125	1	20131017	6
8617376	20110107	14991975	19411020	1	20110107	5
8620368	20110914	15708760	19230622	2	20110914	4
8653483	20120629	16554855	19301001	1	20120629	4
8683509	20110509	15339877	19391122	2	20110509	6
8721673	20110221	15109827	19450122	2	20110221	4
8722745	20130619	17636990	19320419	1	20130619	6
8738543	20110903	15678193	19390823	1	20110903	5
8755304	20120814	16699073	19530730	1	20120814	5
8758074	20110424	15292290	19300624	1	20110424	4
8787357	20110315	15178507	19221123	1	20110315	5
8791002	20120622	16536896	19280201	1	20120622	5
8799017	20110105	14983529	19280723	2	20110105	5
8808433	20131013	18003367	19551220	2	20131013	5
8818175	20130601	17577785	19231105	1	20130601	4
8832846	20130127	17200177	19790324	1	20130127	6
8848440	20120517	16431216	19681127	1	20120517	5
8854191	20111003	15756996	19690303	2	20111003	4
8867707	20120114	16068996	19460715	1	20120114	5

8895861	20110219	15105648	19581207	2	20110219	4
8908369	20110918	15718989	19280206	1	20110918	6
8908654	20111121	15906982	19370402	1	20111121	4
8909602	20131027	18045895	19541212	2	20131027	6
8911920	20110512	15351403	19271006	1	20110512	4
8914532	20110613	15438101	19470210	1	20110613	4
8921059	20130527	17564004	19590116	2	20130527	5
8928163	20130511	17517217	19270203	1	20130511	4
8958392	20130106	17135343	19490601	1	20130106	4
8967166	20120327	16274791	19450222	1	20120327	4
8969071	20130828	17856308	19300126	2	20130828	5
8972427	20130512	17519062	19480901	2	20130512	5
8981655	20120730	16648392	19270705	1	20120730	6
8990054	20130810	17806225	19500208	1	20130810	5
9026180	20110303	15142773	19420625	2	20110303	5
9059647	20110624	15470253	19581202	2	20110624	4
9063085	20130218	17254161	19241210	1	20130218	6
9066868	20120911	16780368	19420603	1	20120911	5
9073147	20110330	15218996	19500514	1	20110330	6
9080028	20110814	15622335	19501120	2	20110814	5
9080982	20121229	17114687	19310824	1	20121229	5
9081907	20130705	17690909	19301105	1	20130705	6
9082035	20120617	16520702	19610401	1	20120617	4
9089150	20110321	15195769	19290205	1	20110321	6
9092584	20110418	15276738	19470701	1	20110418	5
9124010	20131107	18088516	19471118	2	20131107	5
9132347	20110324	15203486	19480618	1	20110324	6
9140505	20110424	15292513	19271201	1	20110424	5
9143888	20120818	16711278	19730626	1	20120818	5
9150372	20111102	15850650	19400725	2	20111102	4
9168610	20121028	16917006	19430911	2	20121028	4
9169431	20130427	17471287	19300320	2	20130427	6
9170654	20130421	17453202	19220622	1	20130421	6
9199580	20120429	16369629	19541002	2	20120429	5
9219152	20120213	16147456	19570328	1	20120213	5
9228993	20110920	15726163	19560517	2	20110920	5
9286651	20110719	15546658	19511029	2	20110719	4
9323379	20120104	16036896	19560701	1	20120104	4
9335028	20130707	17693894	19310513	1	20130707	4
9349137	20130104	17132785	19510205	2	20130104	6
9389086	20110502	15314341	19300305	2	20110502	4
9408837	20110302	15136972	19320917	2	20110302	4
9410439	20120127	16092281	19281025	2	20120127	6
9451096	20130827	17853239	19550301	1	20130827	5
9460791	20121030	16923258	19290224	1	20121030	4
9469141	20110615	15446318	19310626	2	20110615	4
9486899	20110901	15671012	19421017	2	20110901	5
9497432	20111005	15770311	19540307	1	20111005	5
9506267	20131118	18120046	19300409	1	20131118	4
9515677	20120612	16508090	19380614	2	20120612	5
9557226	20120320	16257598	19600906	2	20120320	6
9584161	20110613	15436949	19320928	2	20110613	6
9593219	20130923	17938243	19530918	1	20130923	5
9594712	20110504	15323084	19291119	2	20110504	5
9626975	20110327	15210305	19610323	2	20110327	4

9682988	20121111	16963201	19350201	2	20121111	5
9714878	20110914	15709273	19280202	1	20110914	5
9739053	20130506	17500915	19521027	2	20130506	6
9753859	20120223	16176824	19311128	1	20120223	4
9757646	20120629	16552686	19200806	1	20120629	6
9790141	20111216	15983915	19320913	2	20111216	5
9816620	20121211	17061805	19480619	2	20121211	6
9843510	20111010	15782842	19331228	1	20111010	4
9860655	20131225	18237649	19481101	1	20131225	4
9863109	20130625	17653842	19170916	1	20130625	6
9865547	20120320	16256854	19520416	2	20120320	4
9910365	20130604	17588616	19590903	2	20130604	6
9913273	20130519	17540905	19330715	2	20130519	5
9930761	20110210	15078224	19500220	1	20110210	5
9948225	20130621	17643544	19281201	1	20130621	6
9962792	20131009	17995170	19300323	1	20131009	4
9991746	20111010	15782668	19570914	1	20111010	6
10018469	20120513	16417474	19571220	1	20120513	5
10067411	20121006	16857700	19511005	1	20121006	6
10073468	20120507	16398880	19280803	1	20120507	5
10091051	20120105	16041094	19600121	2	20120105	5
10094505	20111220	15994044	19281010	1	20111220	4
10118044	20110803	15589993	19531020	2	20110803	6
10118715	20131007	17985937	19290826	1	20131007	5
10175276	20120625	16541964	19321201	1	20120625	4
10189534	20121113	16970577	19580112	2	20121113	5
10193938	20120301	16193155	19291005	1	20120301	4
10197770	20121217	17080767	19660127	2	20121217	6
10201066	20110425	15295805	19630621	2	20110425	6
10201511	20121001	16837085	19550320	1	20121001	4
10219291	20131203	18165860	19191210	1	20131203	5
10227619	20131219	18223374	19490616	1	20131219	5
10234261	20110428	15304266	19511011	1	20110428	4
10267433	20101224	14952644	19470613	2	20101224	5
10288047	20130730	17762754	19370104	2	20130730	4
10334028	20120622	16537122	19290320	1	20120622	4
10353910	20130719	17735916	19500713	2	20130719	6
10376600	20110904	15678723	19570405	2	20110904	5
10393121	20121118	16985782	19540427	1	20121118	5
10428543	20130118	17178155	19330613	1	20130118	5
10434727	20120617	16520964	19441231	1	20120617	5
10488730	20130903	17877068	19340328	1	20130903	5
10491448	20130802	17775206	19541101	2	20130802	6
10544293	20120705	16573784	19610117	2	20120705	4
10580377	20121113	16971025	19350315	2	20121113	5
10608465	20110405	15233351	19440724	2	20110405	5
10617091	20130103	17124300	19610225	2	20130103	6
10641017	20110907	15691043	19560320	2	20110907	6
10653620	20111113	15883262	19240705	1	20111113	4
10654781	20120930	16831684	19771221	1	20120930	6
10675748	20131230	18247729	19510520	1	20131230	6
10684272	20110207	15062363	19471102	1	20110207	5
10685231	20120311	16229542	19670422	2	20120311	6
10706806	20120417	16340575	19330601	1	20120417	5
10707058	20120823	16724668	19250202	1	20120823	5

10727090	20121026	16914212	19491124	1	20121026	4
10740042	20110919	15720442	19270502	1	20110919	4
10740393	20110901	15671766	19311019	1	20110901	4
10757729	20120528	16457425	19280916	1	20120528	6
10775527	20120507	16399535	19800603	2	20120507	6
10781358	20120619	16526516	19481117	2	20120619	6
10855397	20110415	15270695	19271015	1	20110415	6
10865540	20130116	17170845	19511110	2	20130116	6
10866634	20120723	16629967	19250101	1	20120723	6
10895291	20110607	15419881	19310814	1	20110607	4
10914086	20120223	16177158	19271209	1	20120223	6
10924115	20120525	16452264	19150116	1	20120525	4
10935690	20110326	15209270	19291115	1	20110326	6
11007979	20121106	16948492	19380703	2	20121106	5
11020272	20120917	16797643	19240205	2	20120917	5
11045117	20110922	15733483	19430211	1	20110922	4
11108717	20111210	15965968	19550816	2	20111210	6
11116691	20120421	16351442	19730731	1	20120421	5
11121667	20121028	16917317	19361013	1	20121028	4
11134999	20110909	15699380	19340621	1	20110909	4
11159969	20111114	15887357	19280408	1	20111114	4
11192102	20110408	15247456	19590920	2	20110408	4
11210074	20130920	17931926	19361130	2	20130920	6
11217746	20120315	16245224	19630720	2	20120315	4
11220841	20120321	16260545	19640115	1	20120321	5
11226327	20131113	18108787	19251015	1	20131113	6
11237813	20120108	16047587	19561225	1	20120108	4
11255713	20120523	16447080	19461226	1	20120523	4
11256294	20101130	14877207	19590711	2	20101130	5
11274241	20130531	17573292	19250726	1	20130531	5
11277160	20120706	16581935	19530302	1	20120706	6
11284416	20110414	15266937	19420528	1	20110414	5
11298729	20110311	15170360	19570702	2	20110311	6
11344062	20121030	16922802	19470201	2	20121030	5
11359130	20130104	17131860	19411215	2	20130104	6
11360513	20111124	15914258	19390915	1	20111124	5
11411271	20120224	16180746	19321111	1	20120224	6
11424525	20130627	17659780	19591210	2	20130627	4
11471015	20110329	15216272	19321010	2	20110329	4
11487460	20120703	16565541	19280429	2	20120703	5
11495059	20121014	16878134	19540204	1	20121014	4
11502048	20131103	18066627	19460915	2	20131103	5
11503143	20120131	16100797	19370125	1	20120131	4
11504077	20121221	17095623	19300712	2	20121221	6
11575523	20110421	15286615	19420710	1	20110421	4
11620249	20131120	18128451	19250815	1	20131120	4
11669491	20130214	17246061	19300706	1	20130214	6
11673179	20131208	18185485	19530712	1	20131208	6
11673613	20110207	15063256	19271003	1	20110207	4
11752435	20110504	15324594	19410405	2	20110504	4
11766226	20130904	17882089	19430812	1	20130904	6
11776253	20110421	15287617	19370813	2	20110421	4
11777723	20120914	16792138	19290511	1	20120914	6
11819684	20130625	17652189	19621027	1	20130625	5
11826930	20120505	16394567	19281225	1	20120505	6

11842221	20110414	15266768	19530720	2	20110414	6
11850047	20111026	15830188	19321115	1	20111026	4
1186153320130515		17531113	19360910	2	20130515	5
11871639	20120502	16384286	19261204	2	20120502	5
11890349	20120717	16613955	19260829	1	20120717	5
1189099620121205		17040681	19590314	2	20121205	5
1193070420121222		17097038	19580527	1	20121222	4
1194868820131003		17973829	19720619	2	20131003	5
11958331	20120604	16478649	19511217	2	20120604	6
11964537	20111026	15829471	19301004	1	20111026	5
11972966	20120105	16040762	19380103	2	20120105	5
11993650	20110305	15149544	19610502	1	20110305	6
1199907820130407		17407292	19301005	2	20130407	4
11999909	20110801	15580856	19480608	2	20110801	5
12020132	20110516	15358666	19291210	1	20110516	4
1202074520130115		17167487	19491010	1	20130115	6
12021102	20120701	16556989	19260603	1	20120701	6
12026367	20121008	16862081	19520819	2	20121008	4
12068143	20110526	15386667	19491015	2	20110526	4
12071588	20110301	15134500	19230928	2	20110301	5
12075886	20110406	15238512	19501110	2	20110406	5
12084967	20111011	15785883	19430804	1	20111011	6
12087126	20110925	15738219	19520728	2	20110925	6
12110655	20120401	16285405	19521017	2	20120401	6
12121470	20120319	16253635	19321024	1	20120319	5
12134019	20110103	14974326	19580603	1	20110103	4
12154471	20110210	15075064	19511028	1	20110210	6
1221023020131014		18007799	19600827	2	20131014	4
12217060	20110310	15167044	19250128	1	20110310	5
12219953	20110531	15397065	19630917	2	20110531	4
1222083620130304		17302176	19360429	2	20130304	5
1223836720130313		17335471	19520412	1	20130313	4
12260445	20110921	15729672	19420507	2	20110921	6
12293468	20110728	15570220	19320727	1	20110728	4
1231224220130623		17647283	19630112	1	20130623	5
12350537	20111225	16005952	19291103	1	20111225	5
12360280	20111101	15846196	19600911	2	20111101	6
12360757	20110628	15479963	19240927	1	20110628	6
1236651720130107		17139986	19581208	1	20130107	5
1237685120130518		17540644	19320104	1	20130518	4
1238956920131117		18117961	19351022	2	20131117	4
12408774	20120923	16814390	19561101	1	20120923	4
12425433	20120715	16606924	19410217	1	20120715	4
12438049	20111123	15913342	19620418	2	20111123	4
12455037	20121116	16983227	19280614	1	20121116	4
12458934	20110416	15271847	19570807	1	20110416	4
12483873	20110417	15272926	19570105	2	20110417	6
1248489820130708		17697572	19601210	2	20130708	6
12501063	20111009	15782362	19290501	2	20111009	5
12511498	20120523	16445918	19501127	2	20120523	4
12517543	20120219	16164319	19431126	1	20120219	5
1252570120130326		17372516	19540103	1	20130326	5
12548413	20111208	15960772	19570319	1	20111208	6
1256257120130728		17757723	19541128	2	20130728	4
12579214	20120813	16694288	19511211	1	20120813	5

1258793820130116		17171888	19580121	1	20130116	4
12589809	20120422	16351855	19250104	1	20120422	5
1259095320130317		17345520	19311202	2	20130317	6
12600758	20120131	16101321	19561105	2	20120131	6
12607704	20110220	15106391	19360822	2	20110220	6
12614947	20120815	16702807	19291205	1	20120815	6
1263714820130929		17954921	19430208	2	20130929	6
12638367	20110119	15025345	19570310	2	20110119	4
1263984820130607		17601485	19551028	2	20130607	6
12640458	20120915	16793818	19320720	1	20120915	5
12646116	20120717	16614560	19350702	1	20120717	6
12677644	20110320	15192400	19240704	1	20110320	4
12709818	20110801	15580247	19530610	1	20110801	5
12723238	20111115	15889674	19361202	2	20111115	6
1278016420131024		18040419	19281005	1	20131024	6
12789536	20120610	16500182	19221021	1	20120610	5
12795492	20120921	16811179	19310125	1	20120921	5
1281606720130306		17310373	19260404	1	20130306	5
12819760	20111119	15902833	19540212	1	20111119	6
12850463	20121208	17052419	19271204	1	20121208	6
12853359	20120802	16657780	19600103	2	20120802	4
12862054	20121115	16978577	19241019	2	20121115	4
12877337	20120226	16182759	19271208	1	20120226	5
12902782	20120719	16621355	19340303	1	20120719	5
12927003	20120903	16752631	19470310	2	20120903	5
12940997	20111228	16014609	19821109	1	20111228	5
12953570	20120618	16523109	19390119	1	20120618	5
12985578	20121118	16985838	19211212	1	20121118	5
1298614020130811		17806716	19611011	2	20130811	4
12999314	20120724	16632908	19280727	1	20120724	4
12999596	20110906	15688049	19271225	1	20110906	4
13011068	20110928	15746925	19501015	2	20110928	6
13019506	20110515	15356519	19580324	1	20110515	5
13020003	20120506	16394997	19540520	2	20120506	5
1302685220131010		17997074	19490103	1	20131010	4
13027424	20121009	16866653	19590913	1	20121009	5
1303537520130331		17383738	19590924	2	20130331	6
13037713	20110104	14975105	19421104	2	20110104	4
1305009420130215		17247907	19210507	1	20130215	5
13061784	20120830	16741633	19280308	1	20120830	4
1306729320131129		18154473	19640729	2	20131129	5
13082172	20111117	15897587	19421102	2	20111117	4
13099995	20120303	16203475	19280410	1	20120303	5
13158684	20111124	15916810	19340605	1	20111124	4
13162179	20110116	15014685	19320505	2	20110116	5
1316262420130827		17854527	19300701	1	20130827	5
1316344520130611		17613937	19290910	1	20130611	6
13188084	20120211	16141437	19311025	1	20120211	5
13193754	20121113	16970994	19300722	1	20121113	6
13201144	20111013	15795102	19280729	1	20111013	5
1320430320130313		17335204	19590102	2	20130313	6
13232967	20110922	15733499	19390524	2	20110922	6
13251848	20120312	16232545	19701029	1	20120312	4
13277731	20120617	16521020	19651017	2	20120617	5
13287440	20110806	15600326	19271224	2	20110806	4

13302631	20111229	16018123	19420528	2	20111229	5
13305221	20110801	15580852	19480804	1	20110801	5
13331776	20110315	15180529	19290619	2	20110315	5
13342091	20130130	17208743	19611207	2	20130130	6
13347778	20110309	15162210	19501212	2	20110309	5
13348737	20121217	17081492	19410216	1	20121217	6
13376119	20130925	17943582	19480725	1	20130925	6
13404518	20111220	15994583	19740612	2	20111220	5
13442701	20130106	17135314	19410407	1	20130106	4
13459900	20130121	17184403	19290807	1	20130121	5
13484043	20131028	18048653	19580812	1	20131028	6
13491413	20121029	16919746	19350715	2	20121029	5
13501423	20131130	18155920	19260505	1	20131130	4
13519158	20110509	15338651	19510905	2	20110509	5
13522935	20121011	16870945	19471101	2	20121011	5
13536464	20120322	16263090	19500326	2	20120322	6
13537172	20110623	15467340	19520319	2	20110623	5
13537558	20120703	16567121	19381025	2	20120703	6
13544111	20110629	15482759	19320212	2	20110629	4
13544859	20120624	16537839	19570418	1	20120624	4
13555390	20120206	16123731	19280317	1	20120206	5
13562599	20120611	16503724	19580209	1	20120611	6
13571749	20111115	15891017	19511112	2	20111115	4
13579356	20120925	16821566	19400127	2	20120925	4
13584753	20110904	15678855	19260625	1	20110904	4
13604223	20110401	15225446	19380806	1	20110401	4
13622554	20131202	18160521	19550222	1	20131202	4
13627468	20110815	15626003	19421123	1	20110815	6
13635999	20120224	16180916	19181223	1	20120224	5
13649348	20110511	15346966	19241020	1	20110511	4
13649600	20110210	15074810	19490719	1	20110210	4
13653946	20111129	15927062	19330502	1	20111129	4
13687871	20130103	17125874	19350701	2	20130103	6
13767583	20110502	15314441	19451030	1	20110502	6
13776448	20111120	15903282	19560510	2	20111120	6
13781049	20111126	15921338	19320305	1	20111126	5
13802498	20110828	15658841	19330114	1	20110828	6
13826852	20110814	15621972	19450901	1	20110814	6
13833039	20111105	15861738	19530807	1	20111105	6
13835159	20120130	16097627	19520318	1	20120130	6
13838409	20120804	16664445	19350228	2	20120804	6
13854085	20111215	15981696	19231220	1	20111215	6
13864374	20110407	15240784	19321223	1	20110407	4
13883573	20130520	17543108	19590201	1	20130520	5
13898674	20110124	15036806	19271222	1	20110124	5
13918859	20131113	18105593	19211001	1	20131113	5
13981550	20130403	17400154	19300316	1	20130403	5
13991850	20110630	15484842	19510510	1	20110630	5
14008389	20120725	16636406	19490319	2	20120725	4
14009019	20120911	16778031	19620624	2	20120911	4
14019002	20131204	18172225	19490415	2	20131204	6
14021579	20130701	17670297	19490901	1	20130701	4
14028172	20120819	16711690	19361129	2	20120819	4
14028821	20130416	17439361	19560622	2	20130416	5
14043993	20110218	15103241	19230717	2	20110218	4

14070372	20120913	16788481	19331227	1	20120913	4
14084481	20130616	17625593	19531025	1	20130616	5
14097280	20130611	17611902	19580112	2	20130611	6
14101929	20111031	15839276	19561223	2	20111031	5
14199689	20111019	15811998	19401226	2	20111019	5
14209440	20110627	15475149	19350530	1	20110627	4
14235122	20110531	15397461	19521031	1	20110531	6
14238063	20110629	15482320	19571214	1	20110629	5
14255982	20111017	15802109	19301229	1	20111017	5
14296356	20110918	15719310	19610127	2	20110918	5
14300324	20121228	17113304	19330215	2	20121228	5
14301065	20130523	17554562	19541210	1	20130523	6
14321029	20130426	17468512	19360201	2	20130426	5
14340568	20130430	17478146	19510704	2	20130430	5
14345698	20130423	17459553	19480618	2	20130423	6
14359989	20110725	15560424	19491003	2	20110725	6
14406667	20110210	15078072	19291222	1	20110210	5
14419477	20110924	15737533	19270905	1	20110924	4
14424238	20120505	16394436	19401207	1	20120505	6
14435688	20120806	16668844	19270928	1	20120806	6
14451855	20131201	18156656	19401231	2	20131201	6
14452563	20110316	15184056	19431120	1	20110316	5
14457386	20121216	17077417	19310512	1	20121216	6
14461440	20130812	17810247	19300722	1	20130812	4
14463560	20111029	15837432	19461205	2	20111029	6
14475184	20120805	16665085	19720108	2	20120805	6
14476621	20121020	16898327	19410305	1	20121020	5
14482178	20110614	15442143	19530310	2	20110614	6
14491771	20131228	18244779	19230506	1	20131228	5
14498658	20131015	18010976	19630103	2	20131015	6
14503787	20121107	16951110	19350503	1	20121107	6
14516586	20130417	17443294	19241113	2	20130417	6
14520786	20111207	15955410	19420803	1	20111207	6
14527129	20130429	17473938	19280921	1	20130429	5
14538659	20110810	15609040	19281227	1	20110810	6
14546919	20111007	15779992	19440121	1	20111007	4
14560384	20131025	18042333	19270404	1	20131025	5
14600954	20111228	16014692	19500605	1	20111228	5
14603088	20130901	17865281	19251010	1	20130901	4
14605186	20120229	16189840	19531008	1	20120229	4
14645295	20120424	16358564	19190913	1	20120424	5
14697177	20131225	18237439	19380712	1	20131225	5
14702091	20110608	15424424	19290826	1	20110608	6
14708624	20131107	18086668	19410227	1	20131107	4
14713667	20110522	15375625	19461201	2	20110522	5
14732753	20130113	17159463	19420801	2	20130113	4
14737872	20110124	15037084	19501230	1	20110124	4
14747003	20130718	17732708	19381231	2	20130718	5
14750664	20130815	17821473	19601231	2	20130815	6
14750697	20131001	17963241	19191215	1	20131001	5
14751167	20120921	16812283	19360718	2	20120921	4
14761741	20120804	16664357	19630115	2	20120804	5
14768082	20110518	15366418	19280322	1	20110518	4
14770093	20101210	14912808	19220218	1	20101210	6
14779807	20131227	18243438	19490404	1	20131227	5

14785343	20130504	17493803	19550228	1	20130504	5
14812874	20110507	15334542	19330208	2	20110507	5
14820805	20120215	16154442	19280208	1	20120215	5
14828230	20130505	17496051	19600408	1	20130505	6
14835428	20120314	16241619	19550415	1	20120314	6
14870690	20110516	15360268	19300614	1	20110516	5
14900744	20120930	16831704	19640501	2	20120930	6
14910511	20130222	17269577	19251106	1	20130222	5
14913236	20120830	16741411	19600301	2	20120830	4
14913429	20111215	15980757	19581215	2	20111215	6
14923343	20120611	16500355	19420223	2	20120611	6
14947525	20130629	17665368	19381113	2	20130629	4
14959172	20131220	18225251	19501128	1	20131220	4
14959321	20110726	15561977	19311020	1	20110726	6
14995290	20131116	18117713	19260206	1	20131116	5
15008681	20130219	17259406	19490222	2	20130219	4
15025646	20121129	17019239	19590220	2	20121129	5
15037226	20120709	16588420	19510108	1	20120709	6
15039891	20120829	16739165	19590508	2	20120829	5
15043820	20120726	16640274	19300705	1	20120726	4
15055284	20130506	17499331	19400825	1	20130506	4
15059106	20120506	16394697	19251216	1	20120506	4
15064570	20120522	16443243	19521014	2	20120522	5
15075204	20111218	15987145	19670714	1	20111218	4
15079773	20120605	16483590	19740505	2	20120605	6
15113378	20110908	15692539	19460506	2	20110908	6
15119536	20130729	17760333	19300618	2	20130729	4
15122266	20120706	16580824	19600630	2	20120706	6
15122879	20130322	17363532	19320608	1	20130322	5
15179665	20130620	17641854	19131010	2	20130620	4
15184459	20110809	15608932	19580810	1	20110809	6
15185576	20120430	16372047	19350316	1	20120430	4
15207008	20120929	16831192	19310620	1	20120929	6
15240558	20110225	15124328	19360830	2	20110225	5
15248905	20120427	16367639	19290516	1	20120427	4
15262494	20130613	17619364	19470505	1	20130613	5
15310040	20110801	15581027	19530820	2	20110801	5
15323269	20120419	16345662	19590731	2	20120419	5
15328617	20121025	16912367	19380314	1	20121025	5
15352020	20130423	17460380	19300126	2	20130423	6
15370033	20120423	16355264	19270512	1	20120423	5
15370919	20120104	16036938	19320901	2	20120104	6
15379096	20120404	16299665	19350830	2	20120404	5
15391089	20130412	17428539	19330525	2	20130412	4
15411435	20111220	15994532	19500621	2	20111220	5
15414730	20110316	15184141	19470410	2	20110316	6
15432867	20110902	15673481	19260324	1	20110902	6
15456594	20111114	15886540	19320914	1	20111114	5
15468016	20130306	17308901	19590930	2	20130306	6
15492009	20110416	15272116	19440206	2	20110416	5
15513798	20131111	18097608	19470401	1	20131111	4
15531665	20130802	17775993	19581010	2	20130802	6
15545138	20110801	15580715	19270614	1	20110801	4
15547805	20111204	15943383	19530225	2	20111204	5
15549845	20111109	15874610	19341115	1	20111109	4

15551516	20110428	15304228	19310407	2	20110428	5
15570599	20130804	17781027	19430313	2	20130804	6
15573010	20110327	15210107	19270715	1	20110327	5
15583718	20130423	17460343	19360922	2	20130423	6
15587050	20110517	15362710	19530506	1	20110517	5
15602490	20131114	18112508	19251019	1	20131114	5
15607199	20130716	17720958	19310222	1	20130716	6
15614605	20110412	15259030	19360410	2	20110412	4
15627788	20111029	15837442	19180120	1	20111029	5
15637044	20110409	15249383	19510117	1	20110409	5
15644970	20110509	15339883	19250101	1	20110509	4
15657326	20120718	16617423	19340416	2	20120718	5
15679206	20110625	15472970	19521015	1	20110625	5
15688365	20110316	15183935	19810627	2	20110316	6
15694652	20130415	17434987	19580625	1	20130415	5
15718566	20110402	15228336	19340406	1	20110402	5
15731949	20120307	16217849	19280207	1	20120307	5
15736320	20111016	15801006	19540929	1	20111016	4
15752417	20111003	15761961	19371207	2	20111003	5
15752746	20110107	14991666	19521028	1	20110107	5
15760131	20130501	17482937	19670808	2	20130501	4
15761587	20110602	15406997	19640602	2	20110602	5
15786104	20131116	18117758	19500520	2	20131116	5
15792139	20110116	15014621	19390601	1	20110116	4
15801759	20131025	18042263	19300616	2	20131025	6
15808216	20131225	18238195	19290614	2	20131225	4
15808863	20110818	15636074	19290205	2	20110818	6
15827471	20110105	14984197	19600201	2	20110105	4
15837146	20111223	16002996	19750411	1	20111223	6
15840456	20110423	15291806	19440125	1	20110423	5
15845371	20110920	15725969	19241212	1	20110920	4
15845882	20121024	16907534	19260228	2	20121024	4
15849033	20110824	15650421	19530614	2	20110824	6
15858772	20130518	17540692	19291230	1	20130518	5
15868265	20110603	15412369	19470428	1	20110603	4
15871575	20120117	16077447	19180514	2	20120117	5
15893320	20110112	15005790	19200901	1	20110112	6
15898927	20120613	16511727	19230803	2	20120613	6
15911667	20120515	16424778	19430728	1	20120515	4
15927716	20110507	15334675	19270914	2	20110507	5
15940688	20130627	17659772	19591115	2	20130627	6
15947021	20120127	16092432	19290719	1	20120127	6
15947043	20130513	17522125	19580820	2	20130513	5
15951210	20110121	15031958	19260807	1	20110121	5
15959781	20121216	17077617	19691031	1	20121216	4
15967530	20120321	16259957	19631015	2	20120321	6
15993825	20131013	18003075	19661226	1	20131013	4
15994306	20121211	17061790	19640730	2	20121211	6
16002736	20110927	15744701	19490310	1	20110927	6
16014963	20110606	15415244	19490120	2	20110606	4
16024401	20110505	15327864	19790805	1	20110505	6
16040576	20130814	17818813	19260302	1	20130814	5
16049119	20121217	17080875	19690628	1	20121217	5
16060916	20110926	15740886	19350112	1	20110926	6
16061613	20130313	17336320	19460701	2	20130313	5

16079406	20111018	15808356	19610216	2	20111018	4
16080378	20130824	17846472	19420910	1	20130824	5
16098821	20130715	17720341	19511116	1	20130715	5
16099711	20120501	16377863	19280330	2	20120501	5
16117854	20130217	17250421	19381225	2	20130217	6
16132164	20111116	15892757	19260606	1	20111116	5
16140811	20121105	16940964	19541024	2	20121105	6
16156653	20110808	15604895	19280309	1	20110808	5
16163841	20130812	17807751	19541128	1	20130812	5
16170233	20130719	17735939	19651225	2	20130719	5
16184308	20111013	15794917	19560410	1	20111013	5
16191836	20131017	18020295	19650228	2	20131017	6
16202996	20120104	16036419	19540826	1	20120104	6
16217406	20130407	17407407	19680824	1	20130407	5
16231326	20110407	15242638	19530628	2	20110407	4
16244034	20130430	17477163	19361006	1	20130430	6
16246574	20130505	17496198	19371010	1	20130505	4
16249493	20110810	15612757	19410213	1	20110810	6
16250785	20130703	17680224	19250315	1	20130703	5
16257344	20120928	16829410	19250620	1	20120928	5
16258007	20110427	15301976	19341216	1	20110427	5
16258325	20130919	17929984	19301110	1	20130919	4
16259179	20130105	17134509	19490430	1	20130105	5
16272881	20110624	15472142	19460707	2	20110624	6
16279917	20110727	15567614	19301120	2	20110727	6
16281882	20120409	16315667	19500216	1	20120409	5
16284972	20110501	15309173	19310812	1	20110501	6
16296198	20120912	16784713	19530104	1	20120912	4
16303938	20120302	16201022	19470901	1	20120302	4
16304022	20130505	17496265	19420707	2	20130505	6
16309005	20121002	16841444	19560626	2	20121002	5
16309903	20120206	16124610	19600408	2	20120206	4
16316668	20131209	18189210	19251220	1	20131209	6
16326526	20130407	17407404	19250310	1	20130407	4
16327369	20120117	16075328	19351006	1	20120117	4
16335607	20130429	17475059	19350227	1	20130429	6
16336440	20130702	17675574	19480205	2	20130702	6
16349170	20110313	15172654	19490811	1	20110313	6
16352184	20121107	16950753	19290822	1	20121107	5
16356744	20131013	18003320	19300603	1	20131013	6
16360897	20130401	17389479	19551201	1	20130401	5
16368017	20110623	15469474	19560804	1	20110623	5
16383907	20120628	16550795	19330125	1	20120628	6
16413517	20120718	16617942	19531202	2	20120718	4
16413915	20110407	15243414	19330103	1	20110407	4
16417315	20130614	17623143	19230927	1	20130614	5
16441875	20120509	16408272	19300417	1	20120509	4
16446665	20130401	17389123	19520702	2	20130401	6
16449266	20110620	15458346	19601030	1	20110620	6
16472154	20110213	15084511	19270304	2	20110213	6
16475062	20120308	16221391	19300903	2	20120308	4
16480710	20130121	17184617	19600402	2	20130121	4
16485964	20130829	17858798	19291001	1	20130829	4
16493473	20120617	16520826	19620815	2	20120617	4
16539336	20130206	17234252	19280415	1	20130206	5

16553201	20110419	15279403	19310208	1	20110419	5
16553814	20110301	15133810	19370101	1	20110301	4
16557827	20110304	15147216	19310202	2	20110304	6
16566226	20121228	17112310	19280228	1	20121228	4
16612183	20120922	16813751	19430119	1	20120922	4
16621731	20111217	15986731	19500325	1	20111217	4
16631655	20131007	17986947	19380127	2	20131007	5
16642527	20120612	16507829	19451013	2	20120612	6
16651573	20131013	18003224	19360122	2	20131013	6
16657582	20131124	18137888	19401117	2	20131124	6
16697113	20110530	15394153	19680524	2	20110530	5
16708320	20130615	17625078	19260515	1	20130615	5
16710068	20110409	15249247	19330120	1	20110409	5
16715847	20130923	17935862	19410802	2	20130923	4
16727767	20131022	18032673	19561102	1	20131022	6
16737590	20110818	15635368	19291011	1	20110818	4
16747141	20120115	16069622	19400420	2	20120115	6
16752015	20121205	17040909	19261125	1	20121205	6
16752242	20120117	16076746	19600106	2	20120117	6
16759878	20111024	15823390	19530824	1	20111024	4
16766500	20110124	15036939	19390510	1	20110124	5
16798064	20101226	14954811	19500701	1	20101226	5
16803080	20130206	17232853	19521101	2	20130206	4
16811099	20121017	16890077	19860817	2	20121017	6
16818578	20120826	16729680	19610104	2	20120826	4
16822541	20111109	15874064	19331005	2	20111109	5
16825506	20130403	17399691	19560605	2	20130403	5
16829984	20110918	15719109	19271214	1	20110918	5
16835840	20130630	17665958	19220930	1	20130630	6
16883402	20120208	16131451	19340702	1	20120208	4
16911787	20120922	16813476	19310919	1	20120922	6
16915905	20110418	15275478	19521006	1	20110418	4
16926300	20131211	18198048	19490101	1	20131211	5
16932084	20121115	16979687	19640907	1	20121115	5
16938946	20130523	17553308	19560424	2	20130523	6
16957496	20121107	16952321	19381015	2	20121107	4
16963170	20110513	15354043	19490526	1	20110513	4
16978180	20110919	15722618	19361015	2	20110919	5
16984604	20130810	17806094	19550310	2	20130810	6
17015246	20130606	17597198	19570215	2	20130606	5
17031617	20120829	16738293	19410718	2	20120829	6
17031991	20110308	15159088	19250923	1	20110308	5
17032621	20120204	16119136	19320517	1	20120204	6
17049682	20110526	15388155	19740813	1	20110526	6
17049955	20110323	15202750	19591008	2	20110323	4
17054330	20110427	15302151	19340306	2	20110427	6
17094814	20120229	16189444	19550910	2	20120229	5
17097722	20110531	15397011	19320329	1	20110531	5
17130266	20120421	16351020	19471115	2	20120421	6
17147750	20110506	15332463	19320817	1	20110506	4
17147943	20131021	18029432	19400528	1	20131021	6
17165070	20120517	16431304	19351210	2	20120517	4
17182375	20130328	17378915	19260914	1	20130328	4
17204523	20120926	16823423	19290501	2	20120926	6
17205786	20130714	17715926	19500401	2	20130714	6

17227393	20130304	17300882	19371205	2	20130304	5
17236703	20120417	16340133	19281224	1	20120417	5
17237897	20111116	15893710	19351020	2	20111116	5
17241111	20130626	17657874	19520327	2	20130626	5
17241235	20111113	15883148	19291122	1	20111113	4
17244143	20110612	15434795	19400101	2	20110612	5
17273119	20110414	15266103	19541017	2	20110414	4
17279720	20111023	15820438	19300312	1	20111023	6
17279775	20120207	16125844	19290109	1	20120207	5
17291688	20121230	17115062	19231010	1	20121230	6
17312300	20130426	17468925	19351120	2	20130426	5
17340135	20110731	15574864	19320910	1	20110731	5
17350026	20131002	17967047	19301020	2	20131002	6
17350151	20110320	15192421	19380307	2	20110320	6
17392788	20130730	17763441	19270605	2	20130730	4
17406212	20120716	16610765	19470808	2	20120716	6
17415326	20120917	16797226	19260903	2	20120917	4
17462576	20121206	17044516	19630520	2	20121206	6
17464389	20130128	17202684	19350927	1	20130128	4
17464390	20110412	15259146	19470115	2	20110412	6
17476618	20110521	15374923	19290406	1	20110521	4
17481355	20111210	15966034	19330305	1	20111210	4
17482109	20120403	16295955	19651201	1	20120403	4
17489633	20111228	16015268	19560215	2	20111228	6
17494507	20120313	16237235	19320927	2	20120313	5
17509863	20120319	16253622	19610102	2	20120319	6
17523501	20111203	15942718	19781129	2	20111203	4
17538102	20131024	18039361	19690918	1	20131024	4
17544659	20130814	17817746	19610218	2	20130814	5
17546440	20120226	16182726	19650801	2	20120226	6
17549223	20120214	16150767	19530701	1	20120214	6
17554744	20110615	15446209	19390102	2	20110615	6
17570988	20130727	17756946	19331230	2	20130727	4
17595801	20110325	15207882	19321127	2	20110325	5
17601682	20120321	16260632	19470110	2	20120321	6
17614890	20130625	17654370	19270809	1	20130625	5
17628443	20130805	17783168	19480301	2	20130805	6
17641735	20130815	17821334	19390510	1	20130815	6
17652083	20121201	17024740	19460514	2	20121201	6
17660581	20131009	17992862	19250813	2	20131009	4
17683146	20130224	17274205	19590222	2	20130224	5
17688685	20130321	17360528	19540112	1	20130321	6
17717714	20121217	17081668	19521022	1	20121217	4
17722100	20120619	16528377	19601010	2	20120619	4
17725643	20110531	15396850	19320306	2	20110531	5
17738420	20110111	15001748	19251113	1	20110111	6
17750742	20101223	14950405	19490322	2	20101223	4
17759885	20110210	15077031	19610822	1	20110210	5
17767576	20120815	16702241	19601202	2	20120815	6
17770193	20120724	16634018	19310225	2	20120724	6
17791003	20120530	16463449	19340208	2	20120530	5
17811448	20130925	17945113	19270515	1	20130925	5
17827268	20110105	14983713	19450717	2	20110105	5
17827315	20111226	16009263	19550306	1	20111226	5
17841428	20120131	16101010	19701028	1	20120131	6

17849262	20131009	17994411	19500810	1	20131009	6
17852787	20131208	18185246	19400601	2	20131208	4
17853473	20120724	16633174	19300920	2	20120724	6
17866238	20120820	16714230	19491214	2	20120820	6
17871373	20120527	16454564	19620315	2	20120527	6
17880432	20130114	17163517	19240901	2	20130114	4
17899820	20110604	15414019	19280919	1	20110604	5
17900242	20111105	15862054	19330627	1	20111105	5
17905872	20120403	16294068	19271016	1	20120403	4
17919243	20111104	15860044	19520526	2	20111104	4
17923169	20120924	16817980	19590120	2	20120924	5
17926340	20130228	17287859	19290302	1	20130228	5
17955929	20121205	17041344	19340606	1	20121205	5
17979952	20111101	15846884	19560911	1	20111101	6
17989365	20110611	15434223	19521205	2	20110611	6
17991570	20120925	16821612	19221202	1	20120925	4
17999472	20121224	17100898	19270113	1	20121224	5
18012889	20110928	15747986	19570328	1	20110928	4
18015822	20110308	15157935	19300216	1	20110308	5
18027731	20130111	17156097	19260506	1	20130111	6
18031544	20110924	15737883	19631111	1	20110924	5
18032763	20131121	18130726	19620610	2	20131121	6
18038614	20120115	16069721	19550103	2	20120115	6
18042347	20111024	15823627	19521127	1	20111024	4
18044729	20130430	17477825	19500203	1	20130430	6
18046907	20131027	18045730	19670228	1	20131027	4
18051053	20120510	16412124	19350613	2	20120510	6
18061364	20120205	16119791	19630624	2	20120205	6
18079077	20130608	17604857	19361120	2	20130608	5
18093306	20120502	16384180	19501213	2	20120502	6
18106597	20130115	17166929	19361222	2	20130115	6
18132484	20111227	16011226	19640109	2	20111227	4
18138017	20110920	15726214	19240812	1	20110920	4
18151105	20130828	17856423	19590710	1	20130828	5
18155801	20130331	17383719	19601001	2	20130331	6
18158424	20110623	15469624	19631226	2	20110623	5
18254358	20131028	18048036	19330505	1	20131028	5
18280767	20111017	15804149	19430221	2	20111017	5
18298152	20130422	17454080	19490905	1	20130422	5
18309109	20110210	15074673	19460804	1	20110210	5
18324920	20120920	16808363	19270915	1	20120920	6
18346617	20110406	15236862	19350515	1	20110406	4
18347165	20101228	14961263	19310925	1	20101228	4
18402001	20110207	15061337	19311209	2	20110207	5
18410827	20120208	16133179	19230228	1	20120208	6
18414772	20131009	17996529	19561118	1	20131009	6
18414818	20120312	16232493	19440222	1	20120312	4
18436721	20120403	16295571	19441030	1	20120403	5
18438852	20110815	15625682	19610524	2	20110815	5
18441479	20120801	16654922	19670804	2	20120801	6
18446996	20120426	16364960	19620723	2	20120426	4
18448276	20110406	15235333	19281012	1	20110406	5
18448414	20120201	16107704	19330127	1	20120201	6
18475144	20120725	16637407	19330604	1	20120725	4
18476329	20110302	15139334	19470827	1	20110302	4

18491162	20110105	14983355	19440222	1	20110105	6
1852461320130414		17431669	19390124	1	20130414	5
18525241	20120202	16110940	19250819	2	20120202	6
1852580920130905		17884608	19340801	1	20130905	5
18538039	20110103	14974245	19411127	2	20110103	4
1854094820131206		18181999	19390923	1	20131206	6
18551025	20120713	16603627	19300406	1	20120713	6
1856582720130421		17453464	19580617	2	20130421	5
18568495	20111004	15765602	19440309	2	20111004	4
18568677	20110311	15169719	19480824	1	20110311	5
18573701	20111120	15903344	19560714	1	20111120	5
1860804320130224		17274111	19651118	2	20130224	5
18625337	20120622	16536811	19410113	2	20120622	6
18641720	20110815	15625283	19171214	1	20110815	5
18649633	20101231	14968623	19300106	1	20101231	6
1867547320130105		17133471	19330115	2	20130105	5
18679395	20121007	16858304	19410116	2	20121007	4
18682763	20120322	16263478	19481101	2	20120322	6
18685228	20120820	16715476	19511118	2	20120820	6
18687746	20120105	16040962	19301212	1	20120105	5
18696736	20110207	15062397	19431115	1	20110207	6
18698287	20110306	15150184	19471220	1	20110306	4
18700942	20120528	16457393	19260330	1	20120528	4
1872200420130528		17564077	19450320	2	20130528	4
18725898	20110914	15709528	19351118	1	20110914	4
18732291	20120423	16355438	19281126	1	20120423	5
18741190	20121220	17092176	19880311	1	20121220	5
18749956	20110106	14987294	19341220	1	20110106	6
18757034	20121008	16860441	19470402	1	20121008	6
18761438	20111231	16022017	19280115	1	20111231	5
18761767	20111112	15882725	19461001	2	20111112	4
18762782	20110307	15152976	19280309	1	20110307	5
1878515420130904		17880874	19590309	2	20130904	4
18785427	20120911	16779276	19310826	1	20120911	5
18788528	20121126	17008810	19180830	1	20121126	5
18792206	20111124	15915126	19540515	2	20111124	5
18793743	20110419	15280649	19330629	1	20110419	6
18805975	20120225	16182347	19351020	1	20120225	4
1880669420130609		17605433	19260524	1	20130609	4
18819620	20120318	16250570	19360901	2	20120318	4
18827673	20111202	15939839	19321208	1	20111202	6
18831419	20111115	15889447	19540628	1	20111115	4
18839344	20110825	15652992	19291229	1	20110825	5
18844274	20121112	16965528	19480214	1	20121112	5
1888643620130609		17605481	19360827	1	20130609	5
1890115020130416		17439768	19560123	1	20130416	5
1893172120131118		18120679	19420624	1	20131118	6
18939509	20110309	15163161	19561021	1	20110309	5
18950259	20120327	16274377	19271028	1	20120327	4
1895584520130114		17163697	19401225	2	20130114	6
18979470	20101203	14891168	19370116	2	20101203	5
18982597	20120527	16454699	19610825	2	20120527	5
19001344	20120319	16253066	19290105	2	20120319	6
1901083420131030		18055662	19620501	2	20131030	6
19014518	20120307	16218941	19540626	2	20120307	5

19018952	20121128	17015015	19280910	1	20121128	6
19043960	20120507	16399054	19461215	2	20120507	6
19045488	20130407	17407227	19530916	1	20130407	5
19049413	20120727	16642694	19310416	1	20120727	6
19050909	20131003	17973042	19330523	1	20131003	6
19092843	20120422	16351894	19560728	2	20120422	4
19107290	20120709	16587694	19520112	2	20120709	6
19108088	20130325	17369821	19460725	2	20130325	6
19120435	20110407	15242343	19611111	2	20110407	5
19131965	20130801	17768907	19231115	1	20130801	5
19152659	20120616	16520367	19621201	2	20120616	5
19153174	20110221	15110313	19480215	2	20110221	4
19155465	20110501	15309202	19430605	2	20110501	6
19184533	20121017	16889328	19241011	1	20121017	6
19196577	20110425	15295898	19431124	2	20110425	6
19200578	20110621	15462480	19421202	2	20110621	6
19204558	20110127	15046497	19550924	1	20110127	5
19207159	20130701	17669352	19360610	1	20130701	4
19218634	20120321	16260740	19450618	2	20120321	6
19223246	20131031	18057940	19361223	2	20131031	5
19224669	20110320	15192199	19371225	1	20110320	5
19238392	20130507	17505269	19631016	1	20130507	5
19239351	20110912	15702351	19510801	2	20110912	6
19253964	20130920	17932341	19530617	2	20130920	6
19287139	20110410	15249894	19431115	2	20110410	4
19288609	20120924	16817330	19390406	2	20120924	5
19300573	20110320	15192638	19580702	1	20110320	6
19307405	20130912	17910845	19311015	1	20130912	4
19310953	20130123	17191592	19600609	1	20130123	5
19316600	20111106	15862458	19320624	2	20111106	4
19349714	20111023	15820541	19681022	2	20111023	4
19377787	20120705	16577750	19501224	1	20120705	4
19381205	20110102	14969659	19400515	2	20110102	6
19417653	20130925	17944863	19550102	1	20130925	4
19453760	20121011	16868657	19580216	1	20121011	4
19470612	20110420	15284337	19230708	1	20110420	4
19474147	20111205	15947953	19241021	1	20111205	4
19476654	20130124	17192289	19300215	1	20130124	6
19508908	20130213	17244310	19670202	2	20130213	5
19509092	20110909	15699849	19250524	1	20110909	6
19511945	20120720	16623504	19280312	1	20120720	5
19517658	20130221	17267724	19270219	1	20130221	5
19528940	20111107	15864828	19641111	2	20111107	5
19535661	20110412	15257444	19600524	2	20110412	6
19540546	20110629	15483155	19341104	1	20110629	4
19541129	20120223	16176031	19330331	1	20120223	6
19588875	20120406	16307941	19710707	1	20120406	4
19592304	20120326	16270984	19310605	1	20120326	6
19599576	20130529	17570260	19551120	1	20130529	4
19608447	20110309	15160037	19250223	2	20110309	4
19623144	20111102	15850287	19160306	1	20111102	6
19633773	20130222	17271012	19490316	1	20130222	5
19634925	20110802	15583833	19240103	1	20110802	4
19657251	20130421	17453223	19480901	2	20130421	4
19669773	20120408	16310861	19350405	1	20120408	5

19719927	20120202	16112574	19421111	2	20120202	6
19721778	20110107	14991938	19550707	2	20110107	4
19728586	20121216	17077331	19630207	2	20121216	6
19736255	20120215	16152344	19251201	1	20120215	5
19745052	20110815	15625440	19620421	2	20110815	6
19755761	20110321	15196096	19670528	2	20110321	6
1976817320131105		18078501	19351115	1	20131105	4
19770139	20120720	16623908	19530120	1	20120720	4
19781410	20111225	16005747	19361002	2	20111225	6
1978422620130729		17759810	19461110	1	20130729	5
19806725	20110416	15272374	19490314	1	20110416	6
19808630	20111224	16005283	19470829	2	20111224	4
19819308	20120930	16831833	19190624	1	20120930	5
19858470	20120528	16458099	19620910	1	20120528	6
1987126220130707		17693615	19450605	1	20130707	5
19893802	20110915	15713902	19641201	1	20110915	4
1990386720130303		17296883	19380708	2	20130303	6
19911901	20120430	16371711	19671127	1	20120430	6
19914864	20111223	16002637	19450825	2	20111223	4
19915969	20120706	16580202	19250615	2	20120706	6
1992087720131223		18230818	19560921	1	20131223	6
19924448	20120524	16450183	19241211	1	20120524	6
19950175	20120314	16241634	19160618	1	20120314	6
19960384	20120909	16773023	19240727	1	20120909	6
19976397	20111221	15997273	19540110	2	20111221	5
1998884220130430		17477041	19411228	2	20130430	6
20000628	20101229	14963753	19320702	2	20101229	6
2000834820131119		18125920	19430723	2	20131119	6
2001262820131211		18198027	19350815	1	20131211	5
20032375	20110412	15258905	19530329	2	20110412	4
20043021	20120521	16439940	19541212	2	20120521	6
20050026	20110423	15292050	19630102	2	20110423	4
20060382	20110626	15473770	19330518	2	20110626	5
2006786120130908		17893990	19450511	1	20130908	5
2008338920131119		18123914	19290720	1	20131119	6
2008569220130315		17340937	19540227	1	20130315	5
2009245920130410		17419675	19400109	1	20130410	4
20121044	20110423	15291818	19281222	1	20110423	5
2012549920130331		17383736	19530909	1	20130331	5
20135108	20111202	15939567	19491210	1	20111202	5
20136736	20111128	15924178	19280817	2	20111128	4
20173813	20110116	15014682	19570415	2	20110116	4
2025730120131222		18228351	19680115	2	20131222	5
20271129	20110220	15106134	19430401	2	20110220	5
20274640	20120610	16500183	19240318	1	20120610	6
20278664	20110313	15172511	19890502	1	20110313	5
2030061220130818		17829225	19310203	2	20130818	5
20317935	20120523	16446985	19491226	1	20120523	4
2032133920130228		17288173	19700215	2	20130228	6
20333760	20110413	15262018	19290112	1	20110413	6
2033540420130707		17693612	19271026	1	20130707	6
2034303720130703		17680700	19290601	1	20130703	4
20349966	20120517	16431610	19790609	2	20120517	6
20361562	20111115	15890292	19480326	2	20111115	6
20365257	20111021	15817994	19640704	2	20111021	5

2038369120131127		18148280	19370418	2	20131127	4
20391746	20110125	15040543	19391021	2	20110125	5
20403730	20120620	16530916	19251128	1	20120620	6
20430459	20110307	15152716	19270519	2	20110307	5
20434928	20110215	15092983	19370203	2	20110215	5
20443781	20110313	15172372	19370201	1	20110313	4
20443792	20110327	15210284	19250424	1	20110327	4
2044824220130619		17638185	19410905	2	20130619	6
2046304120131030		18055588	19381013	1	20131030	4
2047343220130815		17821748	19411102	2	20130815	5
20481485	20120618	16524709	19271005	2	20120618	4
20482671	20120526	16454293	19261214	1	20120526	4
20486297	20110106	14988421	19590227	1	20110106	4
2048860220121226		17106595	19520919	1	20121226	5
20489138	20110221	15108086	19420214	2	20110221	6
20490248	20120308	16220141	19231015	1	20120308	6
2050100220130725		17751569	19331115	2	20130725	4
20504443	20110530	15394204	19310614	1	20110530	6
20506609	20110308	15157568	19361206	1	20110308	5
20518778	20110422	15290032	19410125	2	20110422	6
2052005220130917		17925898	19310913	1	20130917	5
20532303	20111122	15910492	19280603	2	20111122	5
20537739	20120730	16646927	19300226	1	20120730	5
20556303	20110425	15294231	19600506	2	20110425	6
20556529	20110329	15216252	19550520	2	20110329	4
20564890	20120625	16540554	19360830	1	20120625	6
2058237020130402		17393790	19521105	1	20130402	6
2058872120130902		17871642	19410319	2	20130902	6
2059177920130428		17471876	19320329	1	20130428	5
20596207	20110613	15439145	19390302	1	20110613	5
20596445	20120405	16302616	19400613	2	20120405	6
20615672	20120413	16331109	19270902	2	20120413	5
20617189	20120401	16285422	19591215	1	20120401	6
20621049	20120224	16180200	19270315	1	20120224	6
2063816820130201		17216799	19300722	1	20130201	5
20697830	20120702	16562429	19521001	2	20120702	6
2071786620130209		17242074	19530704	2	20130209	4
20718767	20120423	16354473	19280324	2	20120423	5
2071926020131029		18051466	19520427	1	20131029	6
20750792	20110108	14993729	19600924	1	20110108	5
2075502620130610		17606836	19261016	1	20130610	4
2075894520131207		18184558	19430207	2	20131207	4
20764550	20110219	15105143	19180426	1	20110219	6
20775193	20110903	15678276	19600701	2	20110903	4
20801609	20120101	16022351	19521005	1	20120101	6
2080228220131018		18023693	19231112	1	20131018	4
20850800	20110811	15616804	19370908	1	20110811	5
20856966	20110510	15343552	19261105	2	20110510	6
20865605	20111111	15880967	19241211	1	20111111	5
2087864220121215		17076657	19371010	1	20121215	4
20904147	20111031	15839934	19530220	2	20111031	5
20915417	20121220	17092349	19641011	2	20121220	4
2092582020131111		18098249	19391122	2	20131111	4
20938210	20121024	16907444	19600408	2	20121024	5
20941428	20110411	15254678	19300319	1	20110411	5

20951717	20111215	15980250	19270606	2	20111215	6
20957033	20120708	16584333	19370615	1	20120708	6
2095993720131014		18007724	19330331	2	20131014	4
20963046	20110815	15623553	19521015	1	20110815	5
20967526	20110410	15249918	19581124	1	20110410	5
2097626520131202		18160897	19630707	2	20131202	6
20978170	20120711	16595291	19410628	1	20120711	4
2098182220130306		17311213	19630816	2	20130306	4
20990345	20110119	15025111	19301203	1	20110119	5
20998032	20110624	15471493	19351013	2	20110624	4
2100022620131024		18039826	19690430	2	20131024	6
21003623	20111031	15840641	19610128	2	20111031	5
21004988	20110801	15581072	19691003	2	20110801	5
21017312	20110414	15267453	19410420	2	20110414	6
21053634	20121024	16909353	19531031	2	20121024	6
21062953	20110815	15624831	19420829	1	20110815	4
21084764	20120606	16489470	19240906	1	20120606	5
2108938320130107		17140591	19461010	2	20130107	6
21097063	20120403	16295301	19340514	1	20120403	6
21106081	20110324	15205813	19510114	1	20110324	4
2110781320131110		18094908	19210810	1	20131110	4
21111193	20120824	16727274	19481226	1	20120824	5
21120638	20110322	15198707	19470822	1	20110322	4
21152885	20121012	16875837	19680531	1	20121012	5
21163393	20110309	15162796	19400713	1	20110309	6
21184985	20120218	16163948	19510223	1	20120218	5
21188829	20110504	15324804	19280302	2	20110504	4
2119862920130730		17763406	19320625	1	20130730	6
21207341	20110216	15096260	19380806	1	20110216	4
2124902520130602		17578431	19500906	2	20130602	4
21264675	20110523	15377044	19210505	1	20110523	5
2127572920131030		18055652	19291017	1	20131030	4
2128969020130505		17496095	19750812	2	20130505	6
2129242220130426		17469719	19230605	1	20130426	6
21297905	20110504	15323870	19240922	1	20110504	4
2130207820130729		17760608	19270902	2	20130729	4
21306627	20111213	15973355	19480405	1	20111213	6
21342881	20110112	15005527	19370718	2	20110112	6
21354983	20120507	16398682	19820321	2	20120507	6
21357697	20110130	15051026	19480824	1	20110130	4
21374981	20110429	15307448	19260901	2	20110429	5
21376829	20120724	16634095	19541205	1	20120724	5
21390965	20120724	16633871	19531106	1	20120724	6
2139714820131223		18231132	19380220	2	20131223	4
21399542	20120504	16388910	19530815	1	20120504	5
21402415	20110727	15567353	19411201	1	20110727	5
21412317	20110810	15611394	19491203	2	20110810	6
21417196	20110116	15014706	19350109	1	20110116	5
21423018	20121207	17049958	19240927	1	20121207	4
21430557	20120523	16446899	19440827	1	20120523	6
21431561	20110307	15153598	19670906	2	20110307	5
21437398	20110408	15244524	19310606	1	20110408	5
21466433	20110419	15279329	19330608	1	20110419	4
21467141	20120706	16580954	19260805	1	20120706	6
2147449720130417		17443541	19431221	2	20130417	4

21475456	20111003	15761090	19560623	1	20111003	4
21489394	20130607	17599579	19470913	1	20130607	4
21491714	20121128	17015591	19340124	2	20121128	4
21500481	20130507	17502554	19260313	1	20130507	6
21506092	20120423	16355498	19170629	1	20120423	6
21511535	20111013	15794802	19351016	1	20111013	5
21520694	20121004	16851104	19400321	1	20121004	5
21524174	20131215	18208494	19560825	2	20131215	5
21536243	20120426	16364731	19480402	1	20120426	5
21555419	20120617	16520607	19220716	1	20120617	5
21566518	20120125	16089994	19401216	1	20120125	6
21591833	20130311	17327006	19640524	1	20130311	5
21599042	20120902	16746949	19340920	1	20120902	5
21611132	20111211	15966354	19720612	1	20111211	6
21619750	20121113	16971637	19680719	2	20121113	6
21626766	20120315	16244898	19400616	1	20120315	6
21629209	20110104	14978401	19510628	2	20110104	6
21634753	20111228	16014744	19470830	2	20111228	6
21671341	20120730	16648676	19720405	2	20120730	6
21673018	20110922	15733316	19661004	2	20110922	4
21674577	20121201	17024321	19390510	2	20121201	6
21674624	20120128	16093985	19510420	2	20120128	6
21686384	20120828	16736255	19480526	1	20120828	5
21693630	20110209	15073509	19310723	1	20110209	4
21712083	20120501	16378301	19590507	1	20120501	4
21712129	20110531	15397810	19570621	1	20110531	6
21715220	20120630	16556293	19281010	2	20120630	4
21741980	20110320	15192598	19550216	1	20110320	5
21769335	20120310	16229331	19251218	1	20120310	6
21774754	20110329	15215332	19600409	2	20110329	6
21774958	20111110	15877935	19551025	2	20111110	6
21775837	20110124	15036048	19270101	2	20110124	6
21791037	20111011	15783915	19261009	1	20111011	5
21791888	20120506	16394902	19501230	2	20120506	6
21803792	20110408	15247478	19451121	1	20110408	6
21808979	20130924	17940731	19650131	2	20130924	6
21826857	20131109	18094410	19491110	1	20131109	6
21837149	20121209	17053096	19570115	2	20121209	6
21843469	20131203	18163196	19470814	2	20131203	6
21846093	20111123	15912830	19560120	1	20111123	5
21846300	20120422	16351955	19670305	2	20120422	5
21849230	20130724	17749293	19390303	2	20130724	6
21873063	20120424	16358036	19720210	2	20120424	5
21873949	20110824	15649947	19690706	2	20110824	6
21875809	20120101	16022532	19651227	2	20120101	6
21905419	20120415	16333472	19841129	2	20120415	4
21931099	20111110	15878023	19470914	1	20111110	4
21951815	20120417	16340610	19250924	1	20120417	6
21954529	20121105	16943585	19590324	2	20121105	4
21961604	20121001	16836650	19340103	2	20121001	6
21965117	20130409	17415550	19630326	1	20130409	6
21980881	20130119	17179623	19580801	1	20130119	5
21982274	20121219	17088965	19360313	2	20121219	5
21990409	20131027	18045998	19620623	2	20131027	6
21997422	20120612	16508016	19510427	1	20120612	5

22004053	20120621	16534268	19260720	1	20120621	5
22005147	20120227	16184982	19540615	2	20120227	6
22010668	20110623	15468758	19710227	2	20110623	5
22012244	20110206	15060184	19441201	1	20110206	6
22019938	20110921	15730420	19210707	1	20110921	4
22020139	20121004	16847861	19471123	2	20121004	6
22021756	20110306	15149963	19440403	2	20110306	5
22023172	20110823	15647375	19371017	2	20110823	5
22025021	20110824	15651233	19281118	1	20110824	4
22034102	20110527	15390041	19260113	1	20110527	6
22057609	20110815	15624614	19460513	2	20110815	4
22076897	20110507	15334124	19640118	1	20110507	5
22082355	20120524	16449744	19110824	2	20120524	6
22106010	20110420	15283512	19631102	1	20110420	6
22108470	20110103	14974307	19510811	1	20110103	6
22121557	20131124	18137769	19581219	1	20131124	6
22161042	20130816	17825991	19490625	1	20130816	5
22178785	20130729	17758941	19370118	2	20130729	5
22181266	20120206	16122372	19241009	1	20120206	4
22183137	20120225	16182250	19511126	2	20120225	6
22198341	20110513	15352853	19290909	2	20110513	5
22222588	20111204	15943371	19521118	2	20111204	4
22246351	20110411	15253243	19651205	2	20110411	6
22288206	20130527	17563360	19540318	2	20130527	5
22294582	20120628	16551394	19350805	2	20120628	5
22300307	20110311	15168151	19731001	2	20110311	5
22304332	20130626	17657244	19290315	1	20130626	5
22326085	20120128	16093954	19471020	2	20120128	5
22328252	20110207	15062690	19310601	1	20110207	6
22332850	20120712	16601115	19490615	1	20120712	5
22354230	20120321	16258825	19240203	1	20120321	5
22354412	20120301	16194099	19740906	1	20120301	6
22374318	20120817	16708705	19341101	1	20120817	6
22382145	20131206	18181109	19290915	1	20131206	5
22388994	20120426	16364752	19320404	1	20120426	4
22396210	20111225	16005587	19340115	1	20111225	4
22396221	20130619	17638130	19300830	1	20130619	6
22397202	20121107	16949062	19260316	1	20121107	4
22404020	20130804	17781026	19540920	2	20130804	6
22405910	20120320	16255524	19590922	1	20120320	6
22434237	20130722	17741445	19370307	1	20130722	4
22451963	20110214	15087333	19560103	1	20110214	4
22452115	20111228	16014812	19670526	2	20111228	6
22462551	20130726	17754625	19660917	2	20130726	6
22505051	20120902	16746903	19570712	2	20120902	4
22517119	20101214	14923491	19420719	2	20101214	4
22517766	20120915	16793945	19601125	2	20120915	5
22520054	20120201	16106938	19540620	2	20120201	5
22528718	20120716	16609732	19240420	1	20120716	6
22554514	20110524	15381280	19500428	1	20110524	5
22567200	20120920	16807989	19911101	1	20120920	5
22568907	20110613	15439029	19361005	2	20110613	5
22578274	20120227	16185227	19250110	1	20120227	4
22593766	20130619	17637215	19540520	2	20130619	5
22593915	20120910	16776683	19480928	2	20120910	5

22594974	20111123	15913761	19700622	2 20111123	4
22597906	20110404	15231408	19530220	2 20110404	5
22601816	20110909	15699757	19280613	2 20110909	5
22609489	20120519	16436404	19540315	2 20120519	6
22611150	20111212	15970723	19440822	1 20111212	5
22611650	20120815	16703029	19231201	1 20120815	5
22624948	20121202	17024804	19920318	2 20121202	6
22640933	20121122	17001125	19481218	2 20121122	5
2264628220131129		18154006	19580213	1 20131129	6
22648891	20110725	15561117	19470428	1 20110725	5
22649394	20120508	16403608	19511025	2 20120508	6
22669289	20110804	15595095	19270225	1 20110804	5
22691765	20110729	15573168	19481017	2 20110729	6
2269375020130121		17183219	19701231	2 20130121	5
22695187	20120908	16772569	19261004	2 20120908	5
2270471820130312		17331469	19621126	1 20130312	4
22727839	20111121	15905954	19370513	2 20111121	4
2273249620130628		17661750	19510717	2 20130628	4
22742514	20111128	15925142	19440324	1 20111128	4
22744601	20101205	14893868	19260106	1 20101205	4
22788734	20120828	16734967	19250119	1 20120828	5
2278942020131123		18137466	19421002	1 20131123	5
22792730	20110315	15180227	19270310	2 20110315	6
22795091	20110504	15320218	19540202	2 20110504	6
22819745	20121114	16975177	19590509	2 20121114	6
2282193820130902		17871429	19490419	2 20130902	6
2282878020130529		17570107	19610411	2 20130529	6
22835036	20120104	16037088	19440623	1 20120104	6
22842519	20120221	16171881	19240706	1 20120221	5
2284438820130103		17127915	19460723	2 20130103	4
2284581420130701		17671498	19410420	1 20130701	5
22855910	20110629	15481509	19400523	1 20110629	6
2286536720130120		17180484	19311026	2 20130120	6
22875929	20111010	15782902	19340914	2 20111010	6
22902698	20110109	14994432	19411004	1 20110109	5
2291091420130708		17698637	19421013	2 20130708	6
22915260	20111019	15812073	19690318	1 20111019	5
22918894	20110112	15004918	19310420	1 20110112	4
22932645	20120116	16072543	19630120	2 20120116	6
22932747	20110430	15308423	19310212	1 20110430	5
2293389720130617		17629214	19590501	2 20130617	4
22936318	20110209	15071325	19580827	1 20110209	4
22951219	20120810	16684573	19330226	1 20120810	5
22955880	20121202	17025083	19600428	2 20121202	6
22964358	20110726	15563776	19611107	1 20110726	4
2296546420130430		17477000	19530730	1 20130430	6
2298183520130422		17455707	19681229	2 20130422	6
22990723	20120129	16094666	19240302	2 20120129	6
2299729120130226		17281238	19600301	1 20130226	6
23002848	20110126	15043472	19450515	2 20110126	5
2300423120130913		17915152	19390901	1 20130913	6
23009929	20110414	15266306	19551217	2 20110414	6
2306607120130320		17356840	19590228	1 20130320	4
2308786720130715		17719489	19260506	1 20130715	4
23092866	20111127	15921805	19471110	2 20111127	5

2310344820130407		17407215	19410320	1	20130407	6
23103799	20110821	15641219	19430606	1	20110821	4
23103948	20110521	15374965	19400512	1	20110521	4
2310481620131013		18003286	19410305	2	20131013	5
23104918	20110410	15250088	19521014	1	20110410	6
23106050	20110323	15202852	19421119	2	20110323	6
23110681	20120911	16780420	19410528	2	20120911	5
23133995	20110413	15260027	19481120	1	20110413	5
2313738420130212		17243433	19270831	1	20130212	6
23154134	20110125	15040508	19570121	2	20110125	4
23156561	20120313	16236038	19620607	2	20120313	5
2317899620131219		18223241	19281224	1	20131219	5
23188354	20110412	15256737	19531001	2	20110412	5
23192770	20120503	16387896	19510505	2	20120503	4
23215034	20120613	16510512	19580121	1	20120613	5
23253283	20110906	15687790	19530415	1	20110906	4
23253385	20120514	16420294	19540329	1	20120514	4
23255278	20110219	15105804	19420824	2	20110219	6
2327271120130909		17897074	19670820	2	20130909	6
23273361	20110502	15315263	19450515	2	20110502	6
2328173420130622		17646558	19391014	2	20130622	6
2331003420130813		17814431	19690102	1	20130813	4
23335106	20110410	15249755	19640601	1	20110410	4
23344130	20110425	15296006	19481206	2	20110425	4
2334610320131226		18240671	19680918	2	20131226	5
23348461	20120401	16285478	19441023	2	20120401	4
2337499420130503		17493721	19401210	2	20130503	6
23383428	20120210	16139944	19300519	1	20120210	6
23387077	20110512	15350377	19490630	1	20110512	5
23410620	20120526	16454135	19680102	1	20120526	5
23415829	20120213	16146365	19380504	1	20120213	6
23420328	20110805	15598555	19270826	2	20110805	5
23431143	20110601	15403649	19401127	1	20110601	5
23431198	20120308	16223022	19280727	1	20120308	4
2344998120130522		17550903	19500920	2	20130522	5
23466695	20110415	15269574	19330920	1	20110415	6
23467790	20120209	16136737	19650907	2	20120209	4
23479303	20110316	15184179	19280818	1	20110316	5
23490837	20120110	16055675	19581211	2	20120110	5
23493289	20110204	15058717	19291111	2	20110204	4
23497725	20120615	16518624	19640507	1	20120615	5
23512494	20121118	16985770	19481101	2	20121118	6
23523991	20110525	15384656	19560810	1	20110525	5
23525522	20111218	15987050	19280826	1	20111218	5
23543148	20110120	15028857	19531201	2	20110120	5
23555988	20110206	15060236	19480229	2	20110206	6
23557724	20111013	15791581	19210409	1	20111013	6
2356280320130930		17956603	19540206	1	20130930	5
2357374220131223		18230751	19640825	1	20131223	5
23576332	20111010	15782994	19630326	1	20111010	4
23577084	20120926	16824317	19361025	2	20120926	5
2357714220130426		17468802	19290610	1	20130426	4
23580338	20120914	16791870	19301120	2	20120914	5
23592010	20110407	15241775	19401214	1	20110407	6
23593126	20120731	16650954	19611210	2	20120731	6

23607514	20130311	17328658	19310420	2	20130311	5
23607901	20111127	15921758	19291220	1	20111127	5
23615852	20130220	17263858	19461015	2	20130220	6
23650104	20110322	15198392	19260505	1	20110322	4
23665852	20111113	15883266	19290709	1	20111113	5
23667643	20120703	16567485	19650312	2	20120703	6
23703880	20110106	14988259	19690725	2	20110106	6
23706732	20110329	15215853	19521114	1	20110329	6
23714092	20130115	17166233	19450307	2	20130115	5
23740376	20110104	14979048	19560229	1	20110104	4
23760294	20130906	17887640	19470104	1	20130906	6
23767977	20110329	15216288	19330424	1	20110329	6
23789324	20110221	15109175	19291202	1	20110221	4
23798803	20131128	18152129	19480519	1	20131128	5
23801174	20131105	18076950	19530106	2	20131105	4
23809441	20121119	16988806	19691018	2	20121119	6
23811816	20110518	15365775	19460827	2	20110518	4
23817314	20110908	15695653	19620621	2	20110908	6
23831994	20110803	15590595	19680530	2	20110803	6
23838917	20130522	17551705	19551205	1	20130522	6
23839501	20110207	15062101	19610722	2	20110207	4
23848966	20110803	15589474	19550515	2	20110803	4
23858084	20130607	17602090	19550625	2	20130607	6
23873054	20120621	16533957	19260125	1	20120621	6
23889590	20101214	14921013	19391107	2	20101214	6
23896186	20120522	16442673	19940204	1	20120522	4
23945177	20131021	18029185	19580906	2	20131021	5
23955319	20120606	16489597	19840427	1	20120606	6
23957020	20121017	16890035	19620910	2	20121017	5
23964923	20110919	15722670	19340816	1	20110919	6
23996567	20130409	17416189	19360730	1	20130409	6
24007678	20120408	16310794	19290201	1	20120408	5
24009378	20120224	16180497	19400210	2	20120224	6
24010433	20131215	18208587	19610310	1	20131215	6
24011823	20120327	16274305	19500720	2	20120327	6
24024586	20120721	16626366	19560203	2	20120721	6
24029127	20121018	16893653	19820608	1	20121018	4
24035516	20101231	14968485	19460617	1	20101231	5
24047436	20120417	16339861	19390506	2	20120417	4
24051590	20110207	15063337	19650422	2	20110207	6
24074246	20110529	15391932	19330421	1	20110529	4
24076617	20110727	15567127	19490404	2	20110727	6
24090377	20130826	17848651	19340210	1	20130826	5
24095496	20120519	16436498	19431003	1	20120519	5
24113039	20130116	17171208	19511104	2	20130116	6
24139524	20110214	15089161	19690811	2	20110214	5
24140894	20111026	15830493	19311019	1	20111026	4
24162445	20110114	15011141	19280615	1	20110114	5
24210035	20120719	16621732	19331031	1	20120719	5
24212655	20120205	16119798	19700417	2	20120205	5
24213136	20110824	15649515	19291122	1	20110824	5
24213158	20110628	15480291	19551030	2	20110628	6
24214026	20120203	16115857	19270214	1	20120203	6
24216237	20131126	18142882	19331212	1	20131126	6
24219349	20111215	15980780	19331202	1	20111215	4

24224804	20120416	16336895	19240108	2	20120416	4
24239234	20130925	17944866	19430929	1	20130925	4
24250837	20110726	15563615	19320725	2	20110726	5
24268722	20110220	15106151	19580710	1	20110220	4
24269929	20120405	16303964	19520701	1	20120405	5
24274440	20111130	15930425	19240123	1	20111130	5
24295554	20111128	15924585	19610620	1	20111128	5
24301551	20120513	16417801	19571031	1	20120513	4
24305224	20110310	15166978	19381013	2	20110310	6
24340112	20121112	16967671	19550412	2	20121112	4
24355940	20121125	17006445	19280404	2	20121125	5
24356125	20131029	18050326	19310604	1	20131029	5
24364656	20121115	16979343	19620210	2	20121115	6
24367440	20120106	16044701	19120819	1	20120106	4
24380118	20110223	15116351	19360109	2	20110223	6
24397624	20110227	15125991	19290308	1	20110227	6
24402606	20110321	15195762	19310320	1	20110321	6
24418573	20110309	15161445	19510404	1	20110309	5
24424848	20111031	15840148	19260302	1	20111031	5
24443423	20120405	16303945	19350601	2	20120405	4
24453494	20111222	16000049	19300105	1	20111222	4
24482255	20120524	16449713	19380915	2	20120524	6
24483849	20131103	18066717	19591003	1	20131103	6
24489665	20120701	16556828	19310520	1	20120701	4
24507184	20110123	15033819	19460905	1	20110123	4
24532647	20100914	14657774	19410402	1	20100914	4
24534267	20120522	16442931	19410722	1	20120522	4
24536990	20120807	16674052	19520922	1	20120807	5
24552032	20120903	16751787	19561111	1	20120903	4
24558187	20110415	15270676	19430821	1	20110415	4
24564703	20130623	17647126	19430328	1	20130623	5
24566072	20111031	15840108	19361024	1	20111031	5
24576054	20110614	15443158	19241002	1	20110614	5
24625272	20111108	15870583	19251013	2	20111108	6
24626559	20110331	15221690	19390210	1	20110331	5
24634013	20130304	17301481	19380124	2	20130304	4
24641132	20101129	14872691	19440811	2	20101129	5
24641303	20110916	15716849	19331114	2	20110916	4
24642884	20130312	17332711	19371129	1	20130312	4
24654748	20111225	16005751	19940414	1	20111225	4
24657350	20131016	18015086	19570215	2	20131016	6
24683543	20110914	15710601	19611015	1	20110914	6
24694802	20120214	16151950	19551213	2	20120214	4
24711420	20120322	16263657	19300922	1	20120322	5
24733811	20120605	16484414	19351220	1	20120605	5
24752350	20110523	15379023	19631031	1	20110523	4
24761500	20110428	15304811	19300312	1	20110428	4
24761919	20110720	15549317	19350205	1	20110720	4
24783093	20111019	15811368	19471113	2	20111019	4
24794089	20130723	17744723	19410927	1	20130723	6
24797497	20110328	15212272	19521203	2	20110328	6
24822579	20121114	16974937	19490303	1	20121114	6
24844664	20130611	17610548	19451125	1	20130611	5
24856631	20131101	18061697	19310910	1	20131101	6
24857190	20110831	15665839	19511222	1	20110831	6

24880920	20111206	15951573	19511003	1	20111206	4
24883496	20130324	17365809	19251023	2	20130324	5
24891563	20110206	15059972	19201204	1	20110206	5
24892511	20120810	16688122	19280906	1	20120810	6
24895565	20120604	16478900	19461121	1	20120604	4
24904594	20110503	15319522	19380102	2	20110503	4
24906249	20130703	17677381	19690219	1	20130703	5
24906647	20110718	15541405	19450317	1	20110718	6
24921026	20120110	16055751	19700521	1	20120110	6
24934574	20111114	15886800	19530704	1	20111114	6
24937368	20120802	16657517	19330103	2	20120802	4
24944556	20121126	17009535	19621231	2	20121126	6
24947259	20110906	15687729	19620526	2	20110906	5
24949211	20130225	17276885	19291012	1	20130225	6
24979382	20130805	17783480	19521007	1	20130805	4
24985146	20130415	17436032	19480604	1	20130415	6
25011341	20110906	15687490	19690713	1	20110906	6
25020206	20130616	17625307	19350909	1	20130616	6
25020897	20120513	16417443	19250101	1	20120513	5
25023352	20120123	16088586	19401026	1	20120123	4
25023636	20120524	16450125	19470525	2	20120524	6
25031327	20110222	15112748	19391101	2	20110222	4
25033118	20120317	16250164	19651110	2	20120317	6
25034520	20120827	16732647	19490201	2	20120827	5
25045425	20110619	15455797	19431225	2	20110619	6
25046199	20121215	17076907	19641221	2	20121215	5
25048311	20131021	18028953	19441120	2	20131021	5
25049461	20130731	17767213	19650916	1	20130731	5
25067054	20130507	17504179	19681029	1	20130507	5
25067656	20120816	16705769	19490412	2	20120816	5
25078062	20121207	17050147	19210701	1	20121207	5
25081178	20111120	15903476	19470320	2	20111120	5
25081930	20131122	18135243	19630213	2	20131122	6
25108978	20120703	16568101	19481130	1	20120703	4
25109619	20110517	15363580	19390217	2	20110517	4
25112372	20110608	15422585	19280414	1	20110608	4
25114185	20120310	16229162	19370310	2	20120310	6
25129468	20111201	15936392	19521105	1	20111201	5
25136861	20130113	17159768	19310625	2	20130113	4
25141655	20131219	18222995	19621230	2	20131219	4
25142410	20120813	16694061	19700617	2	20120813	4
25157873	20130901	17865088	19570620	1	20130901	5
25162021	20130530	17573230	19750528	1	20130530	4
25167548	20110918	15719452	19560110	2	20110918	4
25177199	20131121	18132951	19370115	2	20131121	5
25208859	20120318	16250702	19291116	2	20120318	5
25214077	20121120	16992645	19260529	2	20121120	6
25215263	20131017	18019164	19420527	1	20131017	4
25230517	20120707	16583815	19261127	2	20120707	6
25238964	20101223	14947038	19281226	1	20101223	4
25273476	20110304	15147783	19550427	1	20110304	6
25293270	20120822	16722561	19591208	2	20120822	5
25309039	20120916	16794334	19270424	2	20120916	4
25311857	20130825	17847503	19400625	1	20130825	6
25321180	20130312	17331347	19271113	1	20130312	6

25333204	20121106	16948157	19581220	2	20121106	6
25346003	20111102	15850810	19220828	1	20111102	4
25366045	20120410	16320018	19510526	2	20120410	4
25377779	20120306	16210092	19660605	2	20120306	4
25381048	20130106	17135300	19411127	2	20130106	5
25385802	20120219	16164350	19480102	1	20120219	4
25394358	20110528	15390835	19431018	1	20110528	5
25401314	20101213	14918604	19470702	1	20101213	5
25410622	20131027	18045870	19490410	2	20131027	5
25421732	20110522	15375696	19390817	1	20110522	6
25440806	20130301	17289078	19280502	1	20130301	5
25445583	20110216	15096534	19620116	1	20110216	5
25448877	20111231	16022038	19540913	1	20111231	5
25451007	20121119	16986912	19270916	2	20121119	5
25467805	20120416	16337086	19680915	2	20120416	5
25472837	20120911	16780198	19910620	2	20120911	6
25477285	20111231	16022007	19640529	1	20111231	4
25487552	20110304	15147692	19320620	1	20110304	4
25487927	20110303	15143924	19550803	1	20110303	5
25489003	20121129	17019425	19430505	2	20121129	5
25495801	20120604	16477226	19501214	1	20120604	5
25517344	20130319	17352255	19510924	2	20130319	4
25533759	20110326	15209666	19470517	1	20110326	5
25546569	20111224	16005258	19360604	1	20111224	5
25558898	20120425	16361132	19740324	2	20120425	4
25563535	20121027	16916585	19390216	2	20121027	6
25572025	20110719	15545056	19430618	2	20110719	5
25578454	20101116	14842239	19280926	1	20101116	5
25586509	20120229	16189398	19630221	2	20120229	6
25601347	20121009	16863889	19341010	1	20121009	4
25604880	20110919	15721926	19670701	2	20110919	5
25608075	20101222	14946217	19190723	1	20101222	4
25617338	20111122	15910710	19360201	2	20111122	4
25631510	20120117	16076900	19451014	1	20120117	4
25637187	20121202	17025235	19301001	2	20121202	6
25645356	20110113	15009368	19440923	1	20110113	5
25701784	20120723	16630639	19460915	2	20120723	6
25704692	20121027	16916567	19520111	2	20121027	6
25718698	20130419	17451141	19661022	1	20130419	4
25741428	20110208	15063699	19701113	2	20110208	6
25772741	20120417	16340674	19920511	1	20120417	5
25774305	20120521	16440617	19371002	1	20120521	5
25777100	20120603	16474650	19680302	2	20120603	4
25780603	20120425	16361847	19770122	2	20120425	4
25780852	20130910	17902158	19630216	2	20130910	4
25781720	20130930	17956241	19510211	1	20130930	4
25785697	20120527	16454631	19611105	1	20120527	5
25789973	20110314	15174261	19290927	2	20110314	5
25794314	20130218	17253289	19430901	2	20130218	5
25799955	20130218	17251610	19561019	2	20130218	4
25802000	20120706	16581551	19581031	1	20120706	6
25810597	20110627	15477145	19631121	2	20110627	6
25828540	20131102	18066077	19390121	2	20131102	4
25837541	20110507	15334565	19560130	2	20110507	4
25837574	20131108	18089144	19681216	2	20131108	5

25853092	20120413	16331546	19301222	2	20120413	5
2585641120130624		17650409	19660613	2	20130624	6
2586279720130213		17244480	19500808	1	20130213	4
25869629	20111003	15760484	19310502	1	20111003	5
2588607120130330		17382976	19390830	1	20130330	5
25907475	20120416	16336169	19360809	1	20120416	6
25913386	20110905	15683395	19410115	2	20110905	4
25931162	20111106	15862353	19600906	2	20111106	6
2598730820130223		17273530	19480315	2	20130223	6
25987820	20111009	15782237	19570224	2	20111009	5
25999795	20110614	15442565	19440802	2	20110614	6
26003112	20110123	15033660	19350220	2	20110123	5
26007567	20110619	15455633	19700514	1	20110619	6
2602056420130429		17474871	19360702	2	20130429	6
26026415	20111004	15766893	19590128	1	20111004	4
26054206	20111119	15902899	19551011	1	20111119	6
2606159620130514		17527053	19530128	1	20130514	4
26062748	20110501	15309241	19680517	1	20110501	4
26063229	20110819	15636408	19560820	1	20110819	4
2608501820130512		17519340	19680415	2	20130512	5
26092682	20120311	16229628	19530401	1	20120311	5
26124550	20121017	16889075	19721010	2	20121017	6
26130643	20111114	15887366	19420630	2	20111114	4
26135739	20120214	16151757	19170516	1	20120214	6
2613707520130314		17338644	19520212	2	20130314	5
2615775520131208		18185436	19350603	2	20131208	6
26164114	20111208	15960947	19820405	2	20111208	5
26200293	20110201	15055713	19440426	2	20110201	5
26205403	20120727	16643272	19260126	1	20120727	5
26212282	20110419	15279565	19481013	2	20110419	6
26255867	20111026	15830520	19640403	1	20111026	4
26256224	20111112	15882631	19720727	1	20111112	4
26262511	20110831	15666418	19440920	1	20110831	5
2627539820130110		17153244	19400107	1	20130110	5
26281378	20121125	17006427	19370220	2	20121125	6
2628270220130626		17656621	19260708	2	20130626	5
26314863	20121018	16893402	19341010	1	20121018	6
26342347	20110417	15272626	19221008	1	20110417	6
26370138	20120809	16682952	19390603	1	20120809	6
2637855220130411		17425363	19560331	2	20130411	5
26412680	20111101	15844191	19420816	2	20111101	5
26422797	20110329	15215302	19630326	2	20110329	4
26425616	20121003	16847390	19630324	2	20121003	4
26431516	20111120	15903417	19481115	1	20111120	4
26438573	20121111	16963519	19320104	2	20121111	6
26439281	20120821	16718295	19751228	2	20120821	6
2644233120131020		18025543	19520605	1	20131020	5
26460491	20111026	15829546	19640328	1	20111026	4
26474737	20110920	15724537	19321010	1	20110920	4
26482337	20110607	15417253	19560607	2	20110607	4
2649673120130710		17707603	19521201	1	20130710	6
26498828	20110522	15375618	19600727	2	20110522	4
2652223620130102		17123135	19570717	1	20130102	5
2655557720130726		17754493	19530520	1	20130726	4
2656409020130107		17139365	19531011	1	20130107	6

2661003720130308		17320631	19570930	2	20130308	4
26633294	20110902	15675166	19340808	1	20110902	5
26665281	20121007	16858427	19710718	1	20121007	6
26690755	20110121	15031984	19281114	1	20110121	5
26696082	20110621	15462189	19540606	1	20110621	4
26697983	20120229	16189862	19550719	2	20120229	6
26701735	20120513	16417574	19470614	1	20120513	5
26711320	20120228	16187484	19541016	1	20120228	6
2671679020130813		17814168	19680104	2	20130813	6
26737520	20120803	16657844	19470120	2	20120803	5
26742869	20120305	16208430	19720710	2	20120305	6
26750027	20120404	16299599	19260424	1	20120404	6
26779715	20110911	15701955	19480613	1	20110911	5
26802891	20101219	14936612	19630102	2	20101219	6
26809416	20110608	15423820	19390604	1	20110608	5
2681382120130815		17823579	19500422	1	20130815	6
26815849	20120401	16285391	19311223	1	20120401	4
26818473	20120203	16115938	19570718	2	20120203	6
26827576	20110827	15658398	19460429	1	20110827	4
2682915220130908		17893801	19420609	2	20130908	6
26840960	20110629	15482165	19550925	1	20110629	4
26845498	20121001	16836137	19440520	1	20121001	5
2685559420130108		17144601	19360717	2	20130108	4
26859245	20120119	16084254	19700905	2	20120119	6
2688817520130708		17697666	19290611	2	20130708	6
26891623	20120222	16173984	19440216	2	20120222	4
26892784	20120718	16616830	19300520	1	20120718	6
26902307	20120507	16396436	19440924	1	20120507	5
26905691	20120613	16511515	19400112	1	20120613	5
26915684	20110831	15666960	19640605	1	20110831	4
26939684	20110913	15702667	19551026	1	20110913	6
2695676320131126		18144555	19590312	2	20131126	5
2697011620130830		17862421	19410226	1	20130830	5
26970467	20110102	14969885	19760614	2	20110102	5
26980574	20110313	15172716	19370615	2	20110313	5
26993793	20111224	16005305	19550707	1	20111224	6
27003367	20111006	15775268	19750421	2	20111006	5
2702223720131126		18144530	19650809	2	20131126	6
27024528	20120828	16735788	19700925	2	20120828	4
27041607	20110307	15153969	19540504	2	20110307	6
27043498	20120102	16027602	19240924	2	20120102	4
27048379	20120702	16562590	19250823	1	20120702	4
27081443	20120306	16209983	19480503	2	20120306	6
27110210	20111227	16011913	19370325	1	20111227	4
27120065	20110116	15014417	19320706	1	20110116	6
27121353	20121218	17085577	19510822	1	20121218	6
27122005	20121202	17025011	19590813	1	20121202	6
27122492	20120820	16714323	19560730	1	20120820	5
27125140	20110414	15266360	19360222	2	20110414	4
2713219020131223		18231122	19610517	1	20131223	5
27133648	20110728	15569215	19410806	2	20110728	4
27134958	20120909	16773002	19540109	1	20120909	5
27138381	20121121	16996705	19440706	1	20121121	5
2715622520130304		17300901	19410803	1	20130304	5
27158141	20120101	16022584	19310120	2	20120101	5

27163979	20120530	16463904	19601123	1	20120530	4
27203685	20110426	15298786	19540527	1	20110426	4
27204677	20121013	16877530	19580725	1	20121013	5
27224415	20130520	17543533	19550415	2	20130520	6
27245950	20130319	17353067	19410101	2	20130319	4
27251554	20120819	16711615	19321205	2	20120819	6
27256264	20110830	15662309	19440403	1	20110830	4
27265561	20130725	17752278	19570919	2	20130725	4
27268662	20130115	17167081	19570304	2	20130115	6
27277798	20131118	18122159	19631227	2	20131118	6
27323702	20130415	17434415	19610729	2	20130415	4
27324738	20110305	15148488	19500922	1	20110305	5
27336910	20120304	16204045	19450808	2	20120304	5
27354081	20120319	16254209	19390913	1	20120319	5
27363946	20121114	16974856	19600328	1	20121114	4
27367244	20120124	16089315	19441203	2	20120124	5
27381631	20110228	15127517	19291111	2	20110228	5
27399742	20121223	17097236	19550119	1	20121223	4
27402171	20110405	15233443	19340626	1	20110405	4
27404235	20130928	17954323	19460906	1	20130928	5
27414819	20120828	16736238	19560423	2	20120828	5
27415561	20130410	17421094	19541001	2	20130410	4
27431658	20110426	15299111	19420507	1	20110426	5
27432435	20110815	15624539	19710819	2	20110815	5
27482004	20110627	15476876	19470103	2	20110627	6
27495847	20111209	15963811	19800927	1	20111209	6
27509019	20110807	15600880	19381026	1	20110807	5
27513093	20110114	15011076	19330912	1	20110114	4
27513195	20120205	16119672	19670210	2	20120205	6
27520985	20121024	16907215	19471215	2	20121024	4
27558970	20110607	15418757	19321011	2	20110607	4
27565362	20120131	16098415	19470221	1	20120131	5
27566763	20110804	15593045	19271105	2	20110804	5
27575593	20110308	15159051	19621120	1	20110308	6
27593926	20130830	17862457	19420715	2	20130830	6
27597575	20130102	17121038	19630528	1	20130102	4
27606275	20131102	18066183	19641119	2	20131102	4
27615107	20110828	15659051	19401208	1	20110828	4
27634226	20130222	17270704	19590120	1	20130222	6
27637996	20120721	16626414	19570326	2	20120721	5
27643476	20130513	17522509	19300127	1	20130513	5
27669143	20130708	17697270	19550919	2	20130708	6
27699952	20121208	17052256	19540520	2	20121208	4
27704730	20110330	15219155	19541215	2	20110330	6
27712965	20121112	16967065	19511226	2	20121112	6
27716683	20130803	17780218	19620406	1	20130803	4
27723757	20130816	17825964	19470307	2	20130816	5
27736807	20110625	15473311	19370118	2	20110625	5
27738154	20110906	15688291	19480108	1	20110906	4
27745911	20120607	16492691	19460105	1	20120607	5
27753908	20110306	15150064	19390722	2	20110306	6
27763684	20120507	16398365	19481127	2	20120507	6
27771455	20110413	15263205	19660831	2	20110413	5
27773257	20121128	17014755	19550529	1	20121128	4
27776154	20120402	16290331	19570409	1	20120402	6

27777759	20110401	15226529	19620124	2	20110401	6
27780730	20120806	16667801	19560224	1	20120806	4
27783159	20131016	18014765	19490314	1	20131016	5
27799468	20131008	17990868	19650925	2	20131008	6
27801281	20131127	18148964	19400411	2	20131127	5
27853436	20130923	17937010	19670225	1	20130923	4
27859821	20110307	15154214	19590226	2	20110307	5
27865163	20121120	16994221	19540315	2	20121120	5
27877527	20110220	15106219	19580211	2	20110220	5
27878440	20110310	15166785	19361115	2	20110310	6
27879114	20110419	15280305	19650305	2	20110419	5
27884282	20130123	17191693	19160508	1	20130123	6
27913402	20121104	16938777	19550328	2	20121104	5
27914938	20120424	16358454	19720702	2	20120424	6
27915599	20121203	17031453	19620215	1	20121203	4
27929551	20120415	16333633	19330822	2	20120415	6
27931971	20130512	17519233	19960215	2	20130512	5
27941055	20131207	18184937	19500821	2	20131207	4
27946629	20130202	17219964	19410906	1	20130202	4
27977135	20131003	17973567	19500724	2	20131003	6
27978718	20110918	15719454	19380307	2	20110918	5
27989657	20130311	17327055	19511103	1	20130311	6
28010244	20110629	15483184	19490311	1	20110629	4
28016355	20120828	16735359	19930129	1	20120828	4
28024091	20110913	15705294	19671108	2	20110913	6
28024728	20110328	15212482	19670225	1	20110328	4
28025174	20110628	15480167	19600622	2	20110628	6
28029063	20120417	16340200	19590116	2	20120417	4
28064815	20120217	16161578	19450302	2	20120217	6
28065965	20131211	18198524	19430911	1	20131211	6
28077227	20110919	15720845	19470809	1	20110919	6
28086013	20120426	16364740	19350419	2	20120426	6
28096879	20110824	15649964	19460601	2	20110824	4
28102150	20120212	16143237	19570810	1	20120212	4
28119439	20131012	18002705	19531130	1	20131012	5
28121111	20121018	16893734	19330726	2	20121018	6
28121359	20130415	17435986	19251123	2	20130415	6
28121600	20130114	17163302	19350501	2	20130114	6
28123877	20110626	15473872	19750314	1	20110626	4
28127437	20130113	17159820	19310305	2	20130113	4
28127471	20120910	16776147	19550828	1	20120910	5
28127595	20120801	16654826	19750805	2	20120801	4
28153404	20121101	16928479	19490501	2	20121101	6
28158103	20120817	16709610	19651124	1	20120817	6
28169815	20130514	17525948	19721006	2	20130514	6
28181295	20110101	14969305	19460211	2	20110101	4
28182118	20130824	17846966	19270704	1	20130824	4
28187215	20130721	17738279	19560822	1	20130721	4
28220077	20110601	15402712	19560704	2	20110601	4
28256691	20120430	16371080	19480505	1	20120430	5
28258551	20130813	17815309	19550208	1	20130813	5
28271161	20121222	17096285	19520403	1	20121222	6
28325657	20111013	15795321	19590328	2	20111013	6
28326150	20120603	16474534	19900604	1	20120603	6
28332185	20131225	18237244	19561205	2	20131225	6

2833590220130915		17917588	19450105	2	20130915	5
28373742	20120614	16515369	19370730	2	20120614	6
28375522	20111011	15783390	19561216	1	20111011	5
28377119	20111028	15835917	19320210	2	20111028	5
28383746	20120509	16408280	19350226	2	20120509	4
28410788	20110217	15100661	19830530	2	20110217	4
28419229	20111221	15997440	19620825	2	20111221	5
28429916	20120709	16588186	19630512	2	20120709	5
2844670020130611		17613003	19330526	1	20130611	6
28458653	20110721	15553319	19420609	1	20110721	4
2846394720130502		17489345	19611117	2	20130502	4
28465487	20121108	16956644	19270301	1	20121108	6
28466264	20120918	16801438	19440823	2	20120918	4
28466388	20120731	16650478	19360622	2	20120731	6
28505820	20110318	15190225	19320409	2	20110318	6
28546896	20120919	16805670	19520622	2	20120919	5
28547684	20120416	16337152	19360120	2	20120416	5
2857345720131123		18137670	19361025	2	20131123	5
2857802120130527		17561325	19390111	1	20130527	6
28588627	20120220	16167368	19400107	2	20120220	5
2862411520130702		17677222	19630220	1	20130702	4
2862479520130303		17296854	19490730	1	20130303	4
28643369	20110503	15319343	19640409	2	20110503	4
28643687	20110327	15210141	19750327	1	20110327	4
2865541620130502		17489222	19520103	1	20130502	4
2867816220131003		17974692	19591226	2	20131003	4
28682000	20120614	16515217	19471123	2	20120614	5
2868570320131221		18226969	19681123	2	20131221	6
28730361	20110713	15529307	19241020	1	20110713	4
28738854	20111225	16005760	19641204	2	20111225	6
28761560	20110317	15186874	19630510	1	20110317	4
28763362	20110622	15465025	19690601	2	20110622	4
2876829820130930		17957265	19550518	2	20130930	6
28797797	20110917	15718183	19270414	2	20110917	4
28798858	20110621	15462924	19350201	1	20110621	5
28802359	20111118	15901164	19690512	1	20111118	6
2880261120130925		17945408	19330912	1	20130925	5
28820000	20111222	16000053	19400715	2	20111222	4
28821989	20120424	16357419	19390909	1	20120424	4
28845581	20120721	16626418	19580125	2	20120721	4
28847032	20111123	15912882	19341008	1	20111123	5
28861225	20120723	16629455	19430528	1	20120723	4
28865216	20120112	16063232	19720908	2	20120112	4
2889198720130301		17293268	19771117	2	20130301	5
28918754	20110421	15287077	19590722	2	20110421	6
28943773	20120619	16528077	19350610	1	20120619	5
28946590	20120614	16514961	19790226	2	20120614	5
2894674920130409		17417034	19780130	1	20130409	6
2895063220131125		18141674	19620912	1	20131125	6
28952536	20111011	15786530	19450122	2	20111011	6
28955717	20120923	16814185	19311110	1	20120923	6
2897090320130703		17682179	19510410	1	20130703	6
2899066120131017		18019896	19591225	1	20131017	6
29007623	20110106	14986805	19510723	1	20110106	6
29010159	20110601	15403559	19160903	1	20110601	6

29015176	20120308	16223370	19640716	2	20120308	6
29074257	20120823	16725286	19230715	1	20120823	6
29080817	20120728	16645012	19621112	2	20120728	4
29080975	20130925	17944819	19580802	2	20130925	5
29082824	20111004	15766266	19510812	2	20111004	5
29100194	20130127	17200017	19391214	2	20130127	5
29105417	20110609	15424636	19560125	2	20110609	4
29108245	20120415	16333617	19440119	1	20120415	6
29111942	20111127	15921619	19610715	1	20111127	5
29113222	20111201	15935774	19781003	2	20111201	4
29120078	20130923	17938352	19400817	2	20130923	4
29120841	20110326	15209896	19400901	1	20110326	4
29127400	20130806	17789520	19560223	2	20130806	6
29131597	20101224	14953161	19340527	1	20101224	4
29150343	20130724	17748103	19340606	2	20130724	6
29169004	20130205	17230078	19580504	2	20130205	5
29176587	20110329	15215892	19660406	2	20110329	4
29205058	20110626	15473792	19500216	1	20110626	5
29212111	20111003	15761285	19510525	2	20111003	5
29235356	20111022	15819830	19501003	1	20111022	4
29235674	20130317	17345733	19500124	2	20130317	4
29237556	20110218	15103596	19320818	2	20110218	4
29255070	20120607	16493476	19511216	2	20120607	5
29258524	20120611	16502996	19281008	1	20120611	4
29288253	20131114	18112145	19741203	2	20131114	4
29289290	20110519	15369433	19511124	1	20110519	5
29292442	20130102	17122082	19630906	2	20130102	4
29338598	20120808	16679041	19500610	2	20120808	6
29346892	20110929	15750198	19520101	2	20110929	6
29347373	20130425	17463983	19391105	2	20130425	4
29355337	20110217	15099882	19620830	2	20110217	6
29371059	20110520	15373616	19611026	2	20110520	5
29372256	20111017	15804514	19280129	1	20111017	5
29378505	20120724	16634048	19430514	2	20120724	5
29388816	20111005	15770673	19860429	1	20111005	5
29395537	20130307	17313695	19470425	1	20130307	5
29409367	20120811	16690014	19450826	2	20120811	5
29412586	20130306	17312484	19450102	2	20130306	4
29419929	20110614	15442994	19200725	1	20110614	5
29437852	20131027	18045932	19551202	2	20131027	6
29448553	20120311	16229692	19650301	1	20120311	5
29473287	20121107	16950872	19261115	2	20121107	5
29479752	20130305	17306102	19280201	1	20130305	5
29510916	20131206	18179191	19520715	1	20131206	4
29512967	20130414	17431792	19310605	2	20130414	6
29526690	20110815	15625057	19610922	2	20110815	5
29553444	20110221	15107553	19360618	1	20110221	5
29615687	20131002	17968525	19641221	2	20131002	5
29630259	20120603	16474464	19600622	1	20120603	6
29632653	20110802	15581343	19521226	2	20110802	4
29656335	20121106	16947087	19500801	2	20121106	6
29670426	20110418	15276958	19640530	1	20110418	5
29670460	20130403	17399177	19630525	2	20130403	6
29677916	20110408	15246645	19300124	1	20110408	6
29680260	20110131	15053193	19510701	1	20110131	4

29683521	20131013	18003096	19760906	2	20131013	6
29694277	20111107	15864898	19421106	1	20111107	6
29700616	20120508	16403322	19461001	2	20120508	4
29733231	20110327	15210346	19350216	1	20110327	5
29735533	20131013	18003313	19390411	2	20131013	6
29738463	20110517	15363150	19360511	2	20110517	4
29747215	20120510	16410825	19530718	1	20120510	4
29753273	20110227	15126027	19420414	1	20110227	6
29758303	20110528	15391645	19490120	1	20110528	6
29764543	20111014	15798594	19410221	1	20111014	5
29764816	20120109	16050787	19770710	2	20120109	5
29768216	20131026	18045502	19560412	2	20131026	5
29771548	20121003	16845467	19490224	2	20121003	5
29776214	20110529	15392116	19300101	2	20110529	5
29791897	20111218	15986816	19481215	2	20111218	4
29793086	20120806	16668108	19500415	1	20120806	4
29795571	20130528	17567331	19530330	2	20130528	4
29797022	20111207	15953372	19481214	1	20111207	6
29806346	20110814	15621976	19780625	1	20110814	4
29810739	20111029	15837423	19700420	1	20111029	5
29812804	20120812	16690447	19390201	1	20120812	5
29816679	20130321	17359366	19640109	1	20130321	5
29835652	20130728	17757765	19281119	2	20130728	5
29850224	20131021	18029640	19590530	1	20131021	6
29897970	20130617	17627826	19610216	1	20130617	5
29903900	20131013	18003273	19461109	2	20131013	6
29911624	20130611	17614101	19701208	2	20130611	6
29913040	20120129	16094446	19500125	1	20120129	5
29923555	20111115	15891154	19320913	1	20111115	4
29936945	20121104	16938611	19351025	2	20121104	4
29939364	20130811	17806636	19210205	1	20130811	6
29945435	20130820	17835575	19561130	2	20130820	5
29951813	20110314	15176808	19630923	2	20110314	6
29955575	20110419	15280252	19530116	2	20110419	5
29963664	20131215	18208742	19440803	2	20131215	6
29964703	20111218	15987054	19611202	1	20111218	5
29968874	20111203	15942565	19570609	1	20111203	4
29977091	20131210	18193856	19570210	1	20131210	5
29977308	20110429	15306046	19420609	2	20110429	6
29981213	20110114	15011794	19540810	2	20110114	6
29991239	20110807	15600984	19430102	1	20110807	5
29993122	20130607	17602856	19550416	1	20130607	6
30002007	20120418	16343667	19670116	2	20120418	6
30008072	20110925	15738277	19610405	2	20110925	6
30019706	20121219	17087673	19790507	2	20121219	6
30032394	20110221	15109117	19400504	1	20110221	5
30032543	20131007	17986706	19430226	1	20131007	4
30064303	20110523	15378498	19370210	1	20110523	6
30065486	20131226	18240255	19590208	2	20131226	5
30068383	20121009	16866244	19530705	2	20121009	4
30072038	20120703	16567088	19441126	2	20120703	6
30077828	20120429	16369694	19360524	1	20120429	6
30079233	20110616	15450630	19610801	2	20110616	4
30097508	20120605	16483871	19751113	2	20120605	5
30106435	20111128	15923115	19951019	1	20111128	5

30108555	20121126	17009159	19800718	2	20121126	5
30109047	20130514	17528106	19840722	2	20130514	6
30114422	20111030	15837823	19361130	1	20111030	4
30124788	20120319	16253554	19600916	2	20120319	6
30132924	20120207	16127991	19350810	2	20120207	6
30158555	20120618	16524561	19341216	2	20120618	6
30209779	20120410	16320161	19310513	1	20120410	5
30210914	20110321	15195656	19580305	2	20110321	4
30213479	20110821	15641387	19411018	1	20110821	5
30218452	20131015	18007912	19540523	2	20131015	6
30244087	20121201	17024527	19501007	2	20121201	6
30252574	20110929	15750895	19490815	2	20110929	4
30264007	20111212	15970489	19510604	2	20111212	6
30264574	20111026	15830048	19451110	1	20111026	4
30281197	20110720	15549113	19420311	1	20110720	4
30283706	20110123	15034076	19440221	1	20110123	4
30290723	20101220	14938954	19660524	1	20101220	4
30291806	20110808	15603728	19741209	2	20110808	4
30306822	20131211	18199093	19590317	1	20131211	6
30308259	20111013	15794373	19440205	2	20111013	4
30322420	20111020	15813912	19581104	2	20111020	4
30339323	20130420	17452809	19290214	1	20130420	4
30340773	20130905	17886524	19560613	1	20130905	6
30352488	20131014	18007504	19261203	1	20131014	6
30356548	20120115	16069307	19430201	2	20120115	4
30366064	20120913	16788862	19750126	1	20120913	5
30372420	20110208	15065552	19330716	1	20110208	5
30401162	20120816	16705930	19520610	2	20120816	4
30411768	20121206	17045314	19270704	1	20121206	5
30415748	20110105	14983212	19491110	2	20110105	5
30423371	20120406	16306552	19280207	1	20120406	6
30425899	20120405	16304175	19591206	1	20120405	5
30427919	20101212	14916413	19540516	1	20101212	5
30433591	20110912	15702380	19470424	1	20110912	6
30435031	20110801	15578962	19671115	2	20110801	5
30435611	20120508	16404179	19321210	1	20120508	5
30440483	20131204	18172050	19541023	1	20131204	4
30445295	20130203	17220591	19440608	1	20130203	4
30454547	20120311	16229525	19511126	2	20120311	6
30454796	20121106	16947699	19740222	2	20121106	5
30457217	20110726	15564671	19371004	1	20110726	5
30466183	20120911	16781337	19461102	2	20120911	6
30479120	20110326	15209700	19360517	1	20110326	6
30486716	20120813	16694557	19521224	2	20120813	4
30504042	20120228	16187521	19730901	2	20120228	4
30515389	20111210	15966052	19430712	2	20111210	5
30515663	20120308	16223088	19490414	2	20120308	4
30522997	20110520	15373650	19360319	1	20110520	6
30546840	20130513	17523560	19391228	1	20130513	5
30548573	20131009	17996515	19690125	1	20131009	4
30559047	20120628	16551113	19620223	1	20120628	6
30563907	20131113	18107323	19431008	1	20131113	6
30572577	20130528	17566598	19570313	2	20130528	5
30599338	20120605	16483203	19420920	2	20120605	6
30601106	20110510	15341807	19490110	1	20110510	6

30607706	20121116	16983160	19490428	1	20121116	4
30609337	20121103	16937870	19340411	1	20121103	4
30640632	20101215	14928312	19360405	1	20101215	6
30643482	20120711	16596763	19511225	2	20120711	6
30649220	20110405	15233780	19270515	2	20110405	5
30666741	20121230	17115258	19411007	2	20121230	6
30667164	20110613	15437015	19340117	2	20110613	5
30685597	20110106	14988136	19600310	1	20110106	6
30690610	20110628	15479752	19290626	1	20110628	6
30692887	20110418	15276490	19380729	1	20110418	5
30701929	20110926	15739955	19570622	1	20110926	4
30721358	20120503	16388487	19410405	1	20120503	6
30740488	20111004	15766959	19600206	2	20111004	4
30769994	20110619	15455691	19370228	2	20110619	6
30791356	20110825	15653686	19321115	2	20110825	5
30798324	20111226	16008340	19631016	2	20111226	6
30813286	20111124	15916461	19381122	1	20111124	6
30838018	20130905	17886143	20011022	1	20130905	4
30844429	20121119	16990101	19680927	1	20121119	6
30878456	20131026	18045459	19511016	1	20131026	5
30895933	20130304	17302312	19531005	2	20130304	5
30926999	20120220	16167994	19690216	2	20120220	4
30937941	20120620	16531095	19550128	2	20120620	4
30943329	20120427	16367487	19660510	2	20120427	4
30944800	20101220	14940095	19321216	2	20101220	5
30954451	20111117	15897180	19810808	2	20111117	6
30966100	20120930	16831749	19410911	1	20120930	5
30967034	20130624	17650676	19530518	2	20130624	5
30970515	20120922	16813914	19211016	2	20120922	6
30974528	20111123	15914026	19530509	2	20111123	5
30980291	20120814	16699089	19330218	2	20120814	4
31009082	20121205	17041492	19480403	1	20121205	5
31012369	20110214	15088433	19440709	2	20110214	4
31018970	20130712	17714406	19731122	1	20130712	5
31026194	20120919	16805780	19551002	1	20120919	5
31026218	20120827	16732655	19300806	1	20120827	5
31042178	20111219	15988299	19550516	1	20111219	6
31052729	20110611	15434116	19650915	2	20110611	6
31052730	20110804	15592798	19671215	2	20110804	4
31054510	20130303	17296934	19530716	2	20130303	6
31054941	20120525	16451575	19430920	1	20120525	6
31061571	20110124	15035836	19500617	1	20110124	6
31062314	20111124	15915674	19270924	2	20111124	5
31062358	20110919	15720860	19340724	2	20110919	4
31074596	20121024	16908812	19770729	1	20121024	5
31079308	20111009	15782283	19550222	2	20111009	4
31085424	20110414	15265302	19360620	1	20110414	6
31091119	20120802	16657770	19360207	1	20120802	6
31095359	20111230	16020916	19340702	1	20111230	4
31098110	20111103	15853216	19320708	2	20111103	6
31098529	20111012	15791431	19331218	1	20111012	4
31105018	20110712	15526948	19291003	2	20110712	4
31107025	20130722	17741747	19570804	1	20130722	4
31126677	20120514	16419878	19590510	2	20120514	6
31136455	20130428	17471752	19441220	1	20130428	6

31137914	20110502	15314876	19650529	1	20110502	4
31152542	20120924	16817288	19530320	2	20120924	5
31159269	20120821	16717683	19370408	2	20120821	6
31164575	20110310	15166470	19320327	2	20110310	6
31188439	20131125	18139581	19520806	1	20131125	5
31192822	20121204	17034443	19441210	2	20121204	6
31193518	20120207	16129138	19621225	1	20120207	6
31196835	20130519	17541060	19350118	1	20130519	4
31225215	20130505	17495962	19460516	1	20130505	5
31231193	20110610	15431030	19540902	1	20110610	5
31254758	20120709	16588223	19590218	1	20120709	6
31259866	20120618	16524722	19411114	1	20120618	5
31264718	20110219	15105805	19330902	1	20110219	6
31281080	20131118	18120162	19591014	2	20131118	5
31284318	20130512	17519299	19360708	2	20130512	4
31294696	20110203	15057883	19990704	1	20110203	5
31318782	20120208	16132267	19760930	2	20120208	6
31329745	20130701	17671317	19641113	1	20130701	6
31332873	20130721	17738176	19430108	2	20130721	4
31341272	20110225	15123879	19440202	1	20110225	4
31357434	20130820	17836831	19341210	2	20130820	5
31363141	20110223	15117693	19510507	2	20110223	4
31367063	20130308	17320927	19450505	1	20130308	4
31371365	20130930	17957627	19370831	2	20130930	5
31380208	20131024	18039967	19591101	2	20131024	5
31394464	20120607	16490720	19460625	1	20120607	6
31402796	20111227	16010993	19520506	2	20111227	6
31413771	20110406	15238854	19260806	1	20110406	6
31428338	20110302	15139471	19580605	1	20110302	6
31430418	20110104	14979611	19461125	1	20110104	4
31433644	20130407	17407277	19540210	2	20130407	5
31450507	20120414	16333052	19420527	1	20120414	6
31456527	20120128	16093931	19351020	1	20120128	4
31466598	20130605	17592738	19300828	2	20130605	6
31470550	20120628	16550688	19821001	2	20120628	4
31523805	20131020	18025764	19430527	1	20131020	5
31544475	20131007	17985912	19540102	1	20131007	6
31548762	20110327	15210149	19641118	1	20110327	4
31548922	20120624	16538168	19310407	1	20120624	5
31555201	20110617	15452848	19250203	1	20110617	6
31560711	20130919	17929869	19360809	2	20130919	5
31584211	20110103	14975037	19410516	2	20110103	6
31601384	20111011	15787006	19530801	2	20111011	4
31603197	20110219	15105873	19360107	1	20110219	5
31661606	20110723	15557492	19280315	2	20110723	6
31676398	20110830	15663702	19520610	2	20110830	6
31688321	20120715	16606490	19350830	2	20120715	5
31688343	20120820	16713562	19550405	1	20120820	4
31695246	20111129	15928072	19651113	2	20111129	4
31698336	20120409	16313862	19331030	2	20120409	4
31698836	20110518	15366156	19640213	2	20110518	6
31704742	20120408	16310760	19400325	1	20120408	5
31710299	20110311	15170447	19391206	1	20110311	4
31722584	20121125	17006664	19490321	2	20121125	6
31723349	20120919	16805721	19400911	1	20120919	4

31725607	20130411	17422455	19270222	1	20130411	6
31733934	20110616	15450286	19190724	2	20110616	5
31757990	20120315	16245093	19411112	1	20120315	4
31759305	20120202	16112627	19271015	1	20120202	4
31760302	20110418	15275909	19341016	2	20110418	6
31760982	20121004	16851626	19471026	2	20121004	5
31791750	20120416	16336387	19770717	2	20120416	6
31811866	20121223	17097373	19580801	2	20121223	4
31819348	20110106	14988360	19451212	1	20110106	5
31820878	20130924	17941994	19481214	1	20130924	5
31823128	20110106	14986788	19530623	1	20110106	5
31824018	20120710	16592355	19581130	1	20120710	4
31870867	20120610	16499964	19520622	1	20120610	4
31883166	20120808	16678189	19541110	1	20120808	4
31890810	20110107	14991927	19470730	1	20110107	6
31901436	20130106	17135059	19510814	1	20130106	6
31910346	20110728	15570911	19430831	1	20110728	5
31917176	20120731	16651547	19440417	1	20120731	4
31939045	20130425	17466995	19440210	1	20130425	4
31941705	20130527	17563362	19380810	2	20130527	4
31944851	20110115	15014144	19450605	2	20110115	6
31950148	20130122	17186427	19710721	2	20130122	4
31950955	20110217	15099884	19201226	1	20110217	6
31972915	20110323	15202546	19621112	2	20110323	5
31980048	20120716	16610829	19511115	1	20120716	4
31982646	20121130	17021319	19501106	1	20121130	4
32000741	20120221	16171791	19520413	2	20120221	6
32018589	20120326	16272050	19540406	2	20120326	6
32044556	20120303	16203432	19450527	1	20120303	6
32049482	20121107	16951818	19420207	2	20121107	5
32052678	20120325	16268637	19551125	2	20120325	5
32053079	20130901	17865018	19540824	1	20130901	5
32059373	20121016	16885189	19640221	1	20121016	4
32061044	20130430	17478418	19360322	1	20130430	6
32068089	20130107	17138614	19450305	1	20130107	5
32070443	20120907	16769678	19601102	2	20120907	4
32092925	20111003	15761849	19311001	1	20111003	4
32093371	20130308	17320625	19440114	2	20130308	4
32105047	20110720	15550187	19550510	1	20110720	4
32139790	20110806	15600260	19431106	1	20110806	4
32142635	20110525	15385243	19390322	1	20110525	5
32155503	20130623	17646918	19620221	2	20130623	4
32156960	20111102	15850469	19790213	2	20111102	4
32157203	20110401	15223110	19580117	1	20110401	6
32185743	20121015	16881642	19691125	2	20121015	4
32188628	20110511	15347139	19460226	1	20110511	4
32198064	20110730	15574564	19370427	2	20110730	4
32204549	20121215	17076926	19910919	1	20121215	5
32210643	20131224	18233914	19361012	1	20131224	6
32245855	20110103	14974767	19540501	1	20110103	5
32247828	20130131	17211868	19200820	1	20130131	5
32257957	20120305	16207584	19350319	2	20120305	5
32261215	20121019	16896125	19231214	2	20121019	4
32263073	20130723	17744390	19740916	1	20130723	5
32263879	20111018	15806871	19500715	2	20111018	5

3229733920130805		17786345	19291010	1	20130805	5
32307010	20110418	15276166	19270819	1	20110418	4
32310499	20120513	16417763	19480928	1	20120513	5
3231509620130927		17951582	19670619	1	20130927	4
32320051	20111124	15916074	19490205	2	20111124	4
32330635	20111108	15869770	19490504	2	20111108	5
3233152520130207		17238501	19521201	1	20130207	5
3233967620130913		17914666	19300806	2	20130913	5
32348224	20110329	15216321	19830922	2	20110329	4
3235564920130323		17365436	19360525	1	20130323	6
32363341	20110618	15455186	19300329	1	20110618	4
32384524	20110520	15373130	19460405	2	20110520	6
32400672	20120603	16474411	19330910	1	20120603	4
32407208	20111120	15903481	19781105	2	20111120	6
32408096	20110503	15319179	19590718	2	20110503	4
32410234	20121111	16963420	19380215	1	20121111	6
3242691820130410		17419397	19550129	1	20130410	4
32427864	20110415	15269578	19610215	2	20110415	4
3243650320131031		18057299	19300401	2	20131031	5
32450183	20120523	16446116	19420222	1	20120523	4
3246067620130620		17640942	19530810	1	20130620	6
3246243420131023		18037169	19630820	1	20131023	6
32464974	20110408	15246598	19630520	2	20110408	5
32466538	20110426	15298087	19301108	1	20110426	5
32471060	20110714	15533349	19430802	1	20110714	4
3247538020130519		17540901	19380901	1	20130519	5
32475437	20110311	15170420	19581020	1	20110311	6
32484858	20120514	16420498	19451225	2	20120514	5
32501227	20110304	15147800	19391212	2	20110304	5
32509481	20110205	15059454	19670627	1	20110205	6
32511414	20120402	16289881	19600302	1	20120402	6
32519758	20110417	15272830	19580221	2	20110417	5
32537421	20120802	16656984	19591104	1	20120802	4
3253763620130417		17442842	19481102	1	20130417	5
32548155	20110919	15722849	19490205	2	20110919	5
32551272	20120917	16797945	19320211	2	20120917	5
32565518	20110904	15678805	19401219	1	20110904	4
32572057	20110409	15249411	19580212	1	20110409	6
3258211920130106		17135248	19551214	1	20130106	5
32585481	20101218	14936022	19870831	2	20101218	4
32600365	20110918	15719445	19500414	1	20110918	5
32608518	20110323	15202445	19480223	1	20110323	4
32629724	20121117	16985160	19350622	1	20121117	6
32630641	20110313	15172729	19660120	1	20110313	5
32641999	20121025	16912245	19470614	2	20121025	4
3264865020130530		17571367	19500220	1	20130530	6
3265630720130711		17711258	19540525	2	20130711	4
32665422	20110306	15150103	19510521	1	20110306	6
32681257	20110115	15014185	19250808	2	20110115	5
32705274	20110309	15161325	19461105	2	20110309	6
32713410	20110501	15309257	19411112	2	20110501	5
32718573	20110928	15747072	19561116	2	20110928	4
32719076	20120410	16320073	19370311	2	20120410	5
32731854	20110811	15615702	19950318	1	20110811	6
32733689	20110503	15320078	19430610	2	20110503	6

32763647	20130707	17693734	19251216	1	20130707	6
32763896	20120507	16399773	19420718	2	20120507	5
32800025	20110303	15143446	19931102	2	20110303	5
32832556	20130304	17299360	19590516	2	20130304	4
32846449	20120314	16240819	19530222	2	20120314	4
32848387	20110214	15088400	19341208	2	20110214	6
32853557	20120313	16237673	19431221	1	20120313	6
32900597	20120603	16474408	19541202	2	20120603	5
32912337	20130405	17403866	19661031	1	20130405	6
32912837	20121017	16889977	19660212	1	20121017	6
32921623	20120403	16297022	19730729	2	20120403	6
32934784	20130122	17187297	19680906	2	20130122	6
32936304	20111001	15755929	19410324	1	20111001	5
32948097	20120523	16446918	19630310	1	20120523	6
32951363	20130111	17157025	19550916	2	20130111	6
32956584	20121129	17018649	19471005	1	20121129	5
32960568	20110623	15468477	19650220	2	20110623	5
32965438	20110410	15250049	19291120	1	20110410	6
32974439	20110302	15138579	19300211	1	20110302	6
32975556	20130613	17618415	19411121	1	20130613	5
32991278	20120520	16437005	19390413	1	20120520	4
32996035	20110818	15636298	19310909	1	20110818	6
32996831	20130327	17375888	19540403	2	20130327	6
32998735	20120409	16315763	19470924	1	20120409	4
33021646	20110601	15401165	19521205	2	20110601	5
33022047	20110124	15037597	19550209	1	20110124	6
33023142	20130128	17203274	19630125	2	20130128	6
33023415	20131022	18032575	19521110	1	20131022	6
33024076	20130805	17784544	20021215	2	20130805	5
33032314	20120113	16066718	19630214	2	20120113	4
33032950	20110321	15193504	19320510	1	20110321	5
33034912	20110406	15238432	19601108	1	20110406	4
33043877	20120819	16711746	19360424	2	20120819	6
33054385	20121205	17041240	19610216	2	20121205	6
33058490	20110117	15017430	19380801	1	20110117	6
33069102	20121201	17024610	19410310	1	20121201	4
33106864	20110514	15355409	19570812	2	20110514	4
33110268	20111211	15966597	19590123	2	20111211	5
33112968	20120613	16511703	19450603	2	20120613	6
33113416	20111011	15786701	19410202	1	20111011	5
33118535	20130611	17613279	19620426	1	20130611	4
33120717	20120821	16717895	19541126	2	20120821	4
33122473	20120409	16315408	19531012	2	20120409	6
33130426	20130425	17466933	19180120	1	20130425	5
33131849	20110612	15434562	19591111	1	20110612	6
33141127	20131021	18029641	19240406	1	20131021	5
33147181	20130807	17795843	19590530	2	20130807	6
33153694	20120810	16687510	19350219	1	20120810	6
33158257	20110415	15270798	19600529	2	20110415	6
33162264	20111031	15840019	19650104	1	20111031	5
33180835	20130529	17569078	19590101	1	20130529	5
33183118	20110925	15738459	19600327	2	20110925	6
33187461	20120701	16557042	19670110	1	20120701	6
33188635	20110916	15715761	19331217	1	20110916	6
33192233	20110802	15584402	19350910	1	20110802	4

33194193	20110109	14994272	19531107	1	20110109	4
33196666	20111029	15836969	19871127	1	20111029	4
33198242	20121212	17066792	19630918	2	20121212	6
33201819	20120827	16730988	19531001	2	20120827	4
33220063	20120502	16382688	19500223	2	20120502	6
3322258120130718		17731985	19480820	2	20130718	4
3322713320130402		17395793	19350429	2	20130402	5
33228272	20110208	15065705	19540529	1	20110208	6
3324076520130709		17701735	19320504	2	20130709	6
3325201620130729		17760156	19590625	1	20130729	5
33271862	20120327	16274620	19501020	1	20120327	5
33274532	20121120	16993475	19480202	1	20121120	6
33288652	20121217	17080819	19411104	2	20121217	6
33294574	20120627	16548508	19300817	2	20120627	4
3330258820130925		17945749	19421029	1	20130925	6
33302646	20110308	15158005	19461113	2	20110308	4
33321447	20121129	17017605	19480203	1	20121129	6
33323103	20120827	16733020	19610227	2	20120827	6
33325949	20111212	15969804	19391005	1	20111212	4
33353901	20120108	16047530	19381122	2	20120108	5
33356988	20111114	15886201	19560221	1	20111114	4
33357674	20110305	15149358	19610315	1	20110305	5
33358859	20120926	16824667	19350404	2	20120926	6
33368933	20111004	15765179	19280610	2	20111004	4
33371878	20110808	15602185	19390822	2	20110808	6
33373374	20110314	15176000	19521022	1	20110314	4
33377127	20110328	15212306	19500728	2	20110328	6
33377296	20120619	16527882	19791215	2	20120619	6
3339647320130913		17915107	19380614	1	20130913	6
33428454	20120213	16147784	19460924	2	20120213	5
33440436	20110309	15163044	19770929	2	20110309	4
33444143	20110905	15682972	19650921	2	20110905	5
33459982	20120820	16715000	19361211	1	20120820	5
33473686	20111221	15997272	19380108	2	20111221	6
33474623	20111204	15943380	19480223	2	20111204	5
33479877	20110504	15324681	19670111	1	20110504	5
33483237	20110416	15272357	19650212	2	20110416	4
33490389	20111012	15790599	19590320	2	20111012	4
33497539	20110914	15707707	19370113	1	20110914	5
33498565	20110103	14973662	19260504	1	20110103	4
33499604	20120703	16567196	19680530	2	20120703	4
3351092820130226		17281037	19630704	2	20130226	6
33516926	20110210	15078212	19440814	2	20110210	4
33517929	20121128	17014728	19511017	1	20121128	5
33523772	20120822	16721981	19371223	2	20120822	4
33524139	20110127	15046438	19450707	2	20110127	4
3352588120130408		17412022	19950622	2	20130408	6
33531190	20110419	15279872	19380927	2	20110419	6
33534291	20111215	15981588	19500715	2	20111215	5
33538464	20121213	17068989	19690425	2	20121213	4
33541116	20110120	15028523	19410727	1	20110120	4
33541321	20120519	16436506	19521216	1	20120519	6
3354983620130321		17358724	19390606	2	20130321	4
33555623	20120620	16530087	19450402	1	20120620	4
33557301	20111211	15966342	19600106	2	20111211	6

33560031	20121113	16971171	19340415	2	20121113	5
33567645	20120729	16645496	19530901	1	20120729	6
33570591	20131209	18190048	19440419	1	20131209	5
33572144	20110615	15447009	19550720	2	20110615	4
33589730	20121002	16841851	19700409	1	20121002	6
33596519	20120830	16741238	19311118	2	20120830	5
33600430	20110328	15212282	19410424	1	20110328	4
33601466	20131210	18193046	19500810	2	20131210	5
33611302	20110303	15142310	19570815	2	20110303	6
33614652	20111222	16000630	19520501	1	20111222	5
33615440	20130304	17301806	19491015	2	20130304	6
33616670	20120329	16279987	19620918	2	20120329	6
33624189	20110622	15466440	19581116	1	20110622	5
33632041	20130104	17131755	19590606	1	20130104	4
33634116	20110113	15009428	19481001	2	20110113	4
33636485	20120413	16328366	19840508	1	20120413	5
33648667	20120418	16342436	19560315	1	20120418	5
33666410	20130709	17703163	19570805	1	20130709	6
33680921	20110308	15157316	19330215	1	20110308	5
33684978	20110905	15683609	19530602	2	20110905	4
33688298	20130115	17167014	19411030	1	20130115	5
33717714	20130429	17475075	19381203	2	20130429	4
33728119	20120910	16775509	19641207	1	20120910	5
33740431	20101224	14953008	19630329	1	20101224	6
33742744	20110814	15622292	19330511	2	20110814	4
33743338	20110104	14977877	19530913	1	20110104	6
33766197	20110425	15294821	19361106	1	20110425	5
33771016	20120203	16115627	19641010	2	20120203	5
33771390	20120506	16395005	19510110	1	20120506	6
33798708	20131209	18189089	19410702	2	20131209	4
33803939	20120219	16164369	19490817	2	20120219	6
33804501	20110525	15385239	19400731	1	20110525	5
33808592	20120517	16430415	19380425	1	20120517	6
33812554	20120207	16126365	19550420	1	20120207	6
33846547	20110315	15178653	19491016	1	20110315	5
33874714	20110207	15062775	19530116	1	20110207	4
33878341	20110828	15658868	19520206	1	20110828	5
33879764	20131119	18125740	19451118	1	20131119	6
33890469	20110214	15088368	19510701	2	20110214	5
33911910	20110425	15295585	19730728	2	20110425	6
33925096	20110801	15580751	19380815	2	20110801	5
33931145	20130812	17809787	19690726	2	20130812	6
33934962	20120723	16630450	19330520	2	20120723	4
33949701	20131112	18102231	19680117	2	20131112	6
33958531	20110512	15350586	19380425	2	20110512	5
33973329	20130428	17471873	19361026	1	20130428	6
33979496	20130701	17668461	19541116	1	20130701	6
33993930	20121212	17066625	19461101	2	20121212	5
33999767	20130224	17273987	19311205	1	20130224	5
34009320	20110611	15434109	19791127	1	20110611	6
34030029	20110307	15153328	19610502	2	20110307	4
34032092	20110626	15473759	19600208	1	20110626	5
34037542	20121119	16988855	19280821	1	20121119	6
34039184	20111115	15890607	19441013	2	20111115	6
34047875	20110613	15438628	19531118	1	20110613	6

34049644	20120713	16604409	19390812	1	20120713	6
34055602	20120708	16584416	19540205	2	20120708	6
34057120	20120915	16793993	19281003	2	20120915	4
34058292	20110812	15619542	19591218	2	20110812	6
34062958	20111102	15851290	19390815	1	20111102	4
34070683	20111102	15851707	19380928	2	20111102	5
3408412320130707		17693966	19500328	1	20130707	4
34085842	20110326	15209817	19541209	1	20110326	4
3408589720130406		17406577	19370608	1	20130406	5
3408996820130327		17375463	19700910	1	20130327	6
3411188120131009		17992314	19301122	2	20131009	5
34116444	20120708	16584272	19430505	2	20120708	5
34135267	20121112	16967501	19310818	1	20121112	5
3416121220131111		18099309	19511209	2	20131111	5
34162408	20121021	16899239	19580810	1	20121021	6
34164119	20110106	14987846	19761227	2	20110106	6
3417708720130803		17779991	19790501	2	20130803	5
34181743	20110308	15159108	19480124	1	20110308	5
34182768	20121023	16906255	19340716	1	20121023	5
34198599	20121031	16925385	19470401	1	20121031	6
34198851	20110623	15469534	19490321	1	20110623	6
34199649	20110407	15241673	20010528	2	20110407	4
34201531	20110907	15691476	19520718	2	20110907	5
34203957	20121214	17073643	19381226	1	20121214	5
34205851	20120113	16066241	19411208	1	20120113	6
34206081	20121219	17088030	19650226	2	20121219	6
34214067	20120403	16295260	19380221	2	20120403	5
34217668	20120218	16163957	19291124	1	20120218	4
34222985	20120416	16335908	19231121	1	20120416	4
34231942	20110801	15578968	19421029	2	20110801	5
34233540	20110228	15127154	20020710	1	20110228	6
34237699	20111015	15800294	19320520	1	20111015	5
34243599	20120702	16559260	19470226	2	20120702	5
3424382820130526		17560158	19590803	1	20130526	5
34247682	20110216	15096964	19560421	1	20110216	5
34254541	20120923	16814311	19530705	1	20120923	5
34254701	20110607	15419134	19520517	2	20110607	4
34257584	20110208	15067803	19440625	1	20110208	5
3426251620130303		17296882	19650110	1	20130303	4
34264341	20110512	15350167	19510502	1	20110512	4
3426468120130124		17193954	19461207	2	20130124	5
34278847	20110214	15088304	19600120	2	20110214	4
34282252	20110112	15005244	19391104	1	20110112	5
34283517	20110523	15377600	19520801	1	20110523	4
34287804	20120222	16174053	19400211	1	20120222	4
34291333	20110112	15005646	19490920	2	20110112	5
34298152	20110213	15084437	19450323	1	20110213	5
34305481	20111124	15916907	19410708	2	20111124	5
34307863	20120625	16541702	19560113	1	20120625	5
34317663	20110222	15111311	19781216	1	20110222	5
34337036	20110427	15301792	19510415	2	20110427	4
34347154	20120914	16791297	19510509	1	20120914	4
34369658	20110624	15471362	19610822	1	20110624	5
34381641	20120603	16474570	19610329	1	20120603	6
34384651	20110214	15087379	19400722	2	20110214	6

34403231	20131006	17982020	19380722	1	20131006	6
34403899	20110209	15068840	19460525	2	20110209	6
34410407	20111129	15927182	19380731	1	20111129	6
34413315	20110120	15028336	19550102	2	20110120	5
34425075	20130412	17429097	19670806	2	20130412	4
34426852	20121212	17067243	19380712	2	20121212	4
34430665	20121007	16858130	19671130	2	20121007	6
34431691	20111109	15873429	19441220	1	20111109	4
34436732	20121014	16878337	19300618	2	20121014	4
34438136	20111026	15829280	19481015	1	20111026	5
34462538	20110612	15434430	19410911	2	20110612	4
34462992	20110325	15207924	19411120	2	20110325	6
34463713	20130912	17909779	19570310	1	20130912	6
34495982	20110629	15483220	19530725	1	20110629	5
34499451	20131026	18045434	19470111	1	20131026	4
34504057	20110427	15301201	19620522	1	20110427	5
34508435	20111219	15989868	19381225	2	20111219	6
34511621	20110206	15059821	19500302	1	20110206	4
34513285	20110101	14969299	19481215	1	20110101	5
34520973	20110120	15028417	19680728	1	20110120	4
34538620	20120607	16492206	19640308	2	20120607	4
34553281	20131222	18228236	19271014	2	20131222	4
34559085	20111115	15891111	19350725	1	20111115	5
34564539	20130606	17597871	19410403	2	20130606	5
34582100	20121013	16877761	19290203	1	20121013	4
34605113	20110119	15025637	19571109	2	20110119	4
34606230	20110503	15319089	19470601	1	20110503	4
34613780	20120213	16146330	19590120	2	20120213	6
34616063	20131118	18120987	19680219	2	20131118	6
34619766	20110610	15432247	19400613	2	20110610	6
34628927	20120323	16265265	19500319	1	20120323	4
34629964	20120517	16431057	19430610	1	20120517	5
34672429	20111101	15846844	19401216	1	20111101	4
34681511	20110406	15238785	19490510	1	20110406	6
34693339	20130905	17887299	19430304	2	20130905	5
34708253	20131112	18103939	19390216	1	20131112	4
34709621	20130628	17662924	19370326	1	20130628	5
34721636	20130320	17353429	19551012	2	20130320	6
34731754	20111128	15924576	19550328	2	20111128	4
34734015	20110625	15473306	19460821	2	20110625	6
34740142	20110728	15568978	19410402	1	20110728	5
34745432	20120708	16584410	19590520	1	20120708	4
34752404	20130422	17456122	19381016	1	20130422	5
34752595	20120619	16528222	19280528	1	20120619	5
34755470	20120311	16229596	19530811	2	20120311	6
34757807	20120109	16051950	19390410	2	20120109	4
34762760	20110918	15719236	19370415	1	20110918	6
34762920	20120522	16443047	19630819	1	20120522	6
34764686	20121202	17025125	19571130	1	20121202	6
34766955	20131201	18156674	19210307	2	20131201	5
34767254	20120412	16326647	19520414	1	20120412	4
34767969	20130724	17749087	19510101	1	20130724	5
34770600	20110214	15088799	19901021	1	20110214	6
34774351	20110114	15012684	19700530	2	20110114	4
34777509	20110927	15744900	19260606	1	20110927	5

34780831	20131004	17977381	19690403	2	20131004	5
34803333	20110109	14994414	19600119	2	20110109	4
34804165	20131210	18193148	19460926	1	20131210	6
34815264	20120109	16051922	19670801	1	20120109	4
34827300	20120317	16249535	19320102	1	20120317	6
34833006	20110523	15378925	19550202	2	20110523	6
34846350	20131218	18218873	19401029	1	20131218	4
34855862	20120926	16823927	19500402	1	20120926	6
34858225	20120531	16466138	19480817	1	20120531	5
34865866	20130126	17199459	19710917	2	20130126	6
34881419	20111006	15775547	19510226	2	20111006	6
34884689	20120828	16735818	19541115	1	20120828	5
34904046	20110315	15180503	19461221	1	20110315	5
34904875	20110117	15017520	19491216	1	20110117	6
34905721	20110206	15059940	19490125	1	20110206	6
34941134	20110920	15726125	19421005	1	20110920	6
34948908	20131008	17991859	19490125	2	20131008	4
34964835	20111011	15786341	19480520	2	20111011	6
34967594	20130813	17815284	19451125	2	20130813	4
34974497	20131112	18102358	19640301	1	20131112	4
34978422	20130109	17149593	19350101	2	20130109	5
34988608	20110308	15158013	19590522	2	20110308	4
34990222	20111003	15760693	19590220	1	20111003	6
34994735	20121112	16966244	19540102	2	20121112	6
34995794	20110208	15068003	19390102	1	20110208	5
35003533	20111010	15782786	19450326	1	20111010	5
35015691	20110420	15284370	19580528	1	20110420	4
35020134	20130219	17259261	19631224	2	20130219	5
35033580	20120122	16088277	19270216	1	20120122	4
35035360	20120228	16187240	19690308	2	20120228	4
35037899	20110224	15121156	19720629	1	20110224	6
35038676	20110802	15584955	19701220	1	20110802	5
35039908	20110107	14991505	19501125	2	20110107	4
35041793	20110526	15387280	19500626	1	20110526	4
35043493	20110729	15572305	20020130	2	20110729	5
35045217	20110327	15210115	19740216	1	20110327	4
35059177	20110106	14988370	19340510	2	20110106	4
35061804	20130316	17345132	19350203	1	20130316	6
35075071	20111116	15894750	19620702	1	20111116	4
35076789	20121216	17077432	19511118	2	20121216	6
35097531	20110127	15047171	19610221	2	20110127	4
35099628	20120610	16499912	19631219	1	20120610	6
35117385	20111026	15827541	19430318	1	20111026	4
35121289	20120104	16036243	19520819	1	20120104	6
35125043	20120925	16821507	19430624	1	20120925	5
35127334	20111004	15764460	19570913	1	20111004	6
35130122	20111210	15965952	19200906	2	20111210	4
35132548	20110323	15202801	19420903	1	20110323	5
35145530	20110104	14977740	19550207	2	20110104	5
35145621	20121107	16952042	19571124	1	20121107	6
35150835	20120221	16171188	19561213	1	20120221	5
35167841	20110218	15104130	19430115	1	20110218	5
35174437	20110315	15179000	19600228	2	20110315	5
35185901	20120110	16055851	19201231	1	20120110	5
35188842	20110301	15134610	19270306	2	20110301	5

35189356	20110508	15335274	19360428	1	20110508	5
35189630	20110311	15170400	19380512	1	20110311	6
3519883520130709		17702812	19730702	2	20130709	6
35205379	20120712	16600844	19671216	1	20120712	5
35210710	20110921	15729752	19580717	2	20110921	5
35213015	20110228	15128620	19300208	2	20110228	6
35216821	20110821	15641261	19671128	1	20110821	6
3521818920130623		17646912	20041226	1	20130623	5
35223188	20111220	15994190	19410624	2	20111220	5
35226972	20120207	16127889	19620709	2	20120207	5
35238529	20110119	15022528	19491025	1	20110119	4
35248409	20120115	16069630	20031213	1	20120115	6
35253862	20110729	15571567	19441101	1	20110729	5
35256043	20110504	15324376	19670702	2	20110504	6
35265839	20110822	15642781	19430812	1	20110822	5
3528895020130823		17845112	19421118	2	20130823	5
3530148520121108		16956698	19580405	1	20121108	5
35308997	20120523	16446766	19370609	2	20120523	5
35312868	20110902	15676039	19460425	1	20110902	6
3531856020131120		18128820	19661113	2	20131120	4
35339765	20120216	16159028	19430913	1	20120216	4
35341685	20110223	15117666	19691212	1	20110223	6
35343363	20110518	15367179	19460301	1	20110518	5
35352808	20110513	15353576	19361213	1	20110513	5
35353652	20110302	15139494	19371119	1	20110302	5
3536130920130724		17748073	19750820	1	20130724	4
35376228	20110506	15331840	19600820	1	20110506	4
35401242	20111201	15935130	19450713	1	20111201	4
35402609	20120201	16106379	19560825	2	20120201	6
35406996	20110216	15095538	19441111	1	20110216	6
35409735	20101230	14966667	19661108	1	20101230	6
35417244	20110220	15106249	19560729	1	20110220	5
35421728	20110110	14998847	19520128	1	20110110	5
35423428	20110406	15238306	19400512	2	20110406	5
3542643820130701		17670976	19340104	2	20130701	4
35429391	20110601	15401441	19320923	2	20110601	5
3543356820130805		17786334	19520916	2	20130805	6
35448670	20110221	15109640	19401223	2	20110221	6
35449548	20110309	15163139	19530324	2	20110309	5
35451402	20110512	15350692	19740329	2	20110512	5
35455108	20110105	14984248	19481120	2	20110105	5
35456850	20120210	16140176	19260908	1	20120210	6
35465964	20101217	14933214	19441130	1	20101217	5
35466536	20120815	16701156	19470917	1	20120815	5
35470792	20120509	16407643	19641020	1	20120509	5
35471660	20120430	16372018	19551025	1	20120430	5
35478569	20120803	16660833	19541009	1	20120803	4
35480069	20110812	15619509	19510906	2	20110812	4
3548502020130623		17646884	19390201	1	20130623	4
35487913	20110904	15678737	19490828	2	20110904	5
35496845	20111023	15820381	19611010	1	20111023	5
3550105420130120		17180455	19300519	1	20130120	4
3550241120130914		17916910	19561111	1	20130914	5
3550760920130628		17663188	19350119	1	20130628	5
35509092	20110830	15663983	19780716	2	20110830	6

35511365	20121104	16938619	19661005	2	20121104	4
35511934	20120924	16817242	19770610	2	20120924	5
35514831	20130130	17209280	19600315	2	20130130	5
35515685	20110903	15677052	19470501	2	20110903	5
35517067	20130222	17271920	19770126	1	20130222	5
35518639	20130501	17484220	19470610	1	20130501	6
35521438	20111120	15903668	19761225	2	20111120	4
35524620	20120502	16382732	19651009	2	20120502	6
35535901	20121031	16925932	19361021	1	20121031	5
35539685	20111028	15836101	19260919	2	20111028	5
35545916	20110210	15077152	19960907	1	20110210	4
35549043	20130525	17559687	19600115	2	20130525	5
35562368	20120213	16145768	19300501	1	20120213	6
35562686	20110918	15719497	19640915	2	20110918	5
35577174	20111129	15927410	19450918	2	20111129	4
35579181	20120303	16203060	19680902	2	20120303	5
35582355	20120216	16158421	19540102	2	20120216	4
35586904	20110103	14973058	19540810	1	20110103	6
35588886	20130502	17486551	19731210	2	20130502	6
35591356	20110825	15653473	20030311	1	20110825	4
35593261	20110918	15719336	19850814	2	20110918	6
35602483	20130902	17868216	19450304	2	20130902	5
35620598	20131014	18006811	19550104	1	20131014	4
35631039	20110805	15596198	19950418	1	20110805	4
35640369	20111212	15969504	19421016	2	20111212	4
35648965	20121110	16963066	19590130	1	20121110	5
35652676	20110107	14990924	19441117	2	20110107	4
35664358	20130409	17414671	19610317	2	20130409	5
35682032	20120501	16378358	19530610	1	20120501	4
35687491	20130115	17167933	19521027	1	20130115	6
35695035	20111004	15766045	19670724	2	20111004	5
35702988	20130626	17657404	19340129	2	20130626	6
35711150	20130624	17648683	19510901	2	20130624	6
35724095	20110411	15254584	19651013	2	20110411	4
35737098	20120527	16454700	19421118	1	20120527	6
35745223	20110424	15292285	19270202	1	20110424	4
35749689	20110212	15082327	19420311	1	20110212	4
35751894	20110904	15678904	19880103	1	20110904	5
35767498	20110303	15142328	19670615	1	20110303	5
35771574	20121206	17046201	19361018	1	20121206	4
35776693	20130728	17757764	19671130	2	20130728	4
35780133	20110426	15296275	19480830	2	20110426	4
35785570	20110404	15229616	19521122	1	20110404	4
35797967	20111220	15994638	19540208	2	20111220	5
35816898	20120703	16566530	19600303	2	20120703	5
35824556	20110807	15600793	19540415	1	20110807	4
35843835	20110214	15088702	19681031	2	20110214	4
35872630	20120402	16291766	19590701	1	20120402	5
35875264	20110207	15061237	19590730	1	20110207	6
35876983	20110302	15139500	19521101	2	20110302	4
35899560	20110502	15309759	19971218	1	20110502	5
35900471	20120812	16690525	19760627	2	20120812	6
35906560	20130628	17662837	19241025	2	20130628	5
35908180	20131121	18132682	19300116	1	20131121	5
35912197	20130911	17905296	19270925	1	20130911	6

35913918	20110424	15292516	19521223	2	20110424	4
35926400	20111018	15808286	19681008	2	20111018	6
35930199	20110331	15221440	19270707	1	20110331	4
35952820	20120504	16391909	19491206	2	20120504	6
35956457	20130520	17543878	19810912	2	20130520	6
35957701	20111227	16011632	19591202	1	20111227	5
35965094	20110430	15308508	19691001	1	20110430	5
35967647	20110616	15449442	19540901	2	20110616	6
35973945	20110108	14993942	19701206	2	20110108	6
35977550	20130311	17327534	19540628	2	20130311	6
35988671	20111130	15930786	19621028	2	20111130	4
35990944	20110408	15245606	19390618	1	20110408	5
35994184	20110226	15125520	19570607	1	20110226	6
36000552	20110330	15218734	19560810	2	20110330	6
36003960	20120719	16621650	19550220	2	20120719	5
36004021	20131209	18190165	19440904	2	20131209	5
36016598	20130114	17163054	19600501	1	20130114	4
36030725	20110610	15432232	19630905	1	20110610	4
36032323	20130902	17871542	19431110	1	20130902	5
36035855	20110530	15393760	19821129	2	20110530	6
36036201	20110805	15598612	19370927	2	20110805	4
36037464	20110114	15011750	19800714	1	20110114	5
36042054	20120403	16295610	19520708	1	20120403	4
36047640	20121022	16902276	19601107	1	20121022	6
36059195	20110206	15060211	19590105	1	20110206	6
36072249	20110216	15097296	19650903	2	20110216	5
36072692	20110504	15323315	19540609	1	20110504	5
36073866	20130519	17541194	19760227	2	20130519	5
36074007	20120331	16284850	19310922	1	20120331	5
36077164	20110426	15298567	19660303	2	20110426	5
36079251	20110904	15678914	19580824	1	20110904	5
36079682	20120226	16182748	19340806	2	20120226	4
36093784	20110418	15275825	19420115	1	20110418	5
36093944	20121216	17077374	19510929	1	20121216	4
36095177	20120511	16415231	19640827	1	20120511	5
36099419	20120109	16051398	19370101	2	20120109	4
36099873	20110223	15116366	19330929	2	20110223	5
36100944	20110317	15187383	19550618	1	20110317	6
36103976	20130306	17312887	19591002	1	20130306	6
36117052	20120416	16337221	19540422	1	20120416	5
36117609	20130530	17572097	19561027	2	20130530	5
36117905	20130407	17407557	19410720	2	20130407	5
36119649	20121116	16982964	19361104	2	20121116	5
36122335	20131205	18176734	19520120	2	20131205	6
36123510	20110317	15187282	19500401	2	20110317	5
36124626	20120520	16436946	19441208	2	20120520	6
36133898	20110724	15558025	19560930	1	20110724	5
36134028	20111113	15883335	19580120	2	20111113	6
36154957	20121022	16902585	19550120	2	20121022	4
36160186	20110510	15342644	19430120	1	20110510	5
36165783	20130221	17268344	19500628	1	20130221	4
36169309	20120208	16133283	19530930	2	20120208	4
36169989	20120523	16447071	19220117	1	20120523	5
36171832	20110304	15145498	20021203	2	20110304	5
36173587	20130911	17906219	19530912	1	20130911	5

3619103420130410		17419308	19500921	2	20130410	5
36193949	20120515	16424064	19320102	1	20120515	5
36195401	20110421	15287007	19501025	2	20110421	5
36209231	20110523	15379120	19520706	1	20110523	4
36211286	20101231	14967674	19311005	2	20101231	5
36226387	20121204	17035555	19600212	2	20121204	5
36226923	20110227	15125938	19381014	2	20110227	4
36238310	20110102	14969888	19550101	1	20110102	4
36238854	20120520	16436965	19600614	1	20120520	6
36245995	20120416	16336586	19480116	1	20120416	6
36254338	20121027	16916657	19501105	1	20121027	6
36262665	20120328	16277645	19520309	1	20120328	5
36274949	20120105	16041063	19321009	1	20120105	6
36275555	20110817	15633282	19400430	1	20110817	5
3628278720130327		17374148	19460115	2	20130327	5
36296943	20110413	15263444	19640314	1	20110413	5
3629896120121111		16963596	19820703	1	20121111	5
36303318	20111104	15859839	19510522	2	20111104	5
3630906520130511		17518614	19470328	1	20130511	4
3631072220130528		17566685	19630201	2	20130528	6
36312308	20110324	15205327	19500415	2	20110324	6
36321003	20110310	15167015	19710625	2	20110310	5
36321274	20110816	15629313	19391025	1	20110816	5
36321956	20120228	16186984	19860411	2	20120228	6
36323758	20120724	16633792	19341201	2	20120724	5
36342559	20120108	16047600	19450310	2	20120108	4
36343314	20120205	16119734	19320520	1	20120205	4
36345810	20110522	15375407	19560510	1	20110522	5
36350466	20121009	16867519	19460403	1	20121009	4
36352984	20121204	17035335	19341109	1	20121204	4
36353578	20120313	16236988	19420401	1	20120313	4
36355154	20111030	15837973	19981215	1	20111030	4
36356624	20120318	16250727	19660205	2	20120318	6
36357569	20110209	15072955	19501220	2	20110209	5
36366742	20120229	16189028	19330929	1	20120229	5
36371376	20120210	16140851	19510120	1	20120210	4
36374240	20110805	15598713	19660119	1	20110805	4
36378242	20111201	15932979	19320817	1	20111201	5
36380537	20110525	15384594	19440731	1	20110525	4
36381358	20111228	16015654	19510522	1	20111228	6
36381610	20110408	15246105	19670923	2	20110408	6
36382124	20110406	15234200	19520210	1	20110406	6
3638271520131224		18234178	19650401	1	20131224	6
36387118	20111107	15865990	19781020	2	20111107	6
36387209	20110419	15280153	19570501	1	20110419	4
36406389	20120516	16427687	19520609	2	20120516	4
36409366	20120904	16757632	19640116	2	20120904	5
36414241	20110426	15299112	19440124	2	20110426	5
3641549320130812		17809685	19550801	1	20130812	6
3641603220130715		17720262	19651011	1	20130715	5
36422841	20110316	15183408	19580415	1	20110316	6
36430214	20120827	16732388	19621018	2	20120827	6
36433735	20110322	15198828	19250429	1	20110322	5
36438854	20120212	16143372	19411012	1	20120212	5
36443591	20111005	15768766	19340616	1	20111005	4

36457360	20130120	17180576	19660228	2	20130120	4
36463920	20120313	16237166	19361113	1	20120313	5
36464423	20120804	16664306	19330129	2	20120804	6
36467795	20111220	15994049	19470822	1	20111220	5
36473355	20121008	16863072	19650527	1	20121008	4
36481217	20111207	15957014	19430125	2	20111207	4
36496738	20130627	17660841	19530220	2	20130627	4
36513903	20110211	15082277	19961118	1	20110211	6
36518511	20120822	16722487	19441212	1	20120822	5
36520113	20131228	18245015	19620322	1	20131228	4
36524455	20110423	15292039	19830603	2	20110423	4
36530082	20110117	15018110	19500818	2	20110117	4
36533343	20131112	18103310	19990213	1	20131112	4
36545116	20110301	15133295	19411018	1	20110301	4
36547087	20120918	16798995	19470316	1	20120918	5
36558799	20120524	16449990	19350820	2	20120524	4
36564702	20130501	17484197	19480301	1	20130501	4
36578264	20131027	18045857	19470720	1	20131027	5
36585112	20120708	16584297	19260907	2	20120708	5
36600369	20110419	15279716	19730330	1	20110419	5
36603028	20130131	17211164	19620520	2	20130131	6
36606754	20130602	17578496	19561220	2	20130602	6
36607246	20120420	16349941	19490513	2	20120420	5
36611800	20130825	17847140	19671225	2	20130825	6
36616203	20131212	18203234	19500329	2	20131212	5
36616849	20110123	15034071	19530421	1	20110123	6
36622498	20120625	16541132	19360825	1	20120625	4
36625828	20110224	15120915	19440810	1	20110224	5
36629499	20130714	17715888	19510220	1	20130714	6
36631819	20130305	17307191	19630715	1	20130305	4
36634705	20130321	17359941	19470104	2	20130321	5
36636892	20130510	17516933	19540626	1	20130510	6
36643944	20120723	16629098	19700630	2	20120723	4
36654247	20130929	17954850	19511211	1	20130929	5
36654361	20110807	15600659	19521015	1	20110807	6
36659957	20110823	15647082	19511202	1	20110823	5
36668630	20101209	14911126	19521022	1	20101209	5
36669644	20111130	15930270	19680425	1	20111130	4
36676445	20120723	16629553	19420215	2	20120723	6
36677346	20110318	15189915	19400113	2	20110318	5
36682981	20130120	17180396	19250406	2	20130120	6
36689846	20110426	15298210	19421230	1	20110426	6
36690172	20101210	14914568	19430705	2	20101210	4
36694685	20120528	16458000	19600323	1	20120528	5
36703987	20120417	16338989	19640529	1	20120417	6
36706113	20130613	17618796	19510810	2	20130613	6
36707274	20130903	17875823	19540210	1	20130903	6
36716355	20131029	18051688	19511014	1	20131029	4
36724615	20110915	15712032	19701027	2	20110915	4
36732373	20110118	15020596	19900417	2	20110118	4
36745445	20130621	17644085	19350416	1	20130621	5
36746506	20110220	15106047	19670102	1	20110220	6
36766515	20130515	17531274	19640412	1	20130515	5
36770431	20110420	15283326	19321102	1	20110420	4
36773838	20110121	15031890	19270625	1	20110121	6

36775083	20110801	15581096	19420107	1	20110801	4
36781110	20111004	15766872	19500816	2	20111004	5
36781745	20101228	14961291	19541103	2	20101228	6
36786057	20121111	16963377	19480303	1	20121111	4
36792480	20110107	14991482	19620418	1	20110107	6
36794920	20111019	15811833	19340318	2	20111019	5
36796335	20111126	15920933	19610701	2	20111126	5
36797758	20120814	16697833	19401105	1	20120814	6
36797974	20110425	15295444	19510718	1	20110425	4
36798513	20121110	16963003	19290909	1	20121110	5
36802843	20110116	15014784	19590418	1	20110116	4
36810261	20110427	15302049	19630225	2	20110427	5
36811479	20110620	15459751	19530107	2	20110620	4
36814230	20120429	16369497	19400317	2	20120429	5
36820050	20111111	15879371	19710504	1	20111111	4
36830236	20110413	15262494	19561002	2	20110413	5
36834636	20110608	15419966	19210115	1	20110608	6
3683882120130710		17707485	19471002	2	20130710	6
36842838	20101219	14936506	19960403	2	20101219	4
36848325	20120305	16209276	19330814	1	20120305	6
3685407620130315		17342328	19660428	1	20130315	6
36858318	20101225	14954329	19391026	2	20101225	4
36864945	20110819	15638989	19550810	1	20110819	5
36885468	20120213	16147203	19710414	1	20120213	4
36886972	20110120	15028908	19560806	2	20110120	5
36892725	20120617	16520903	19531004	1	20120617	5
36896534	20121022	16902830	19291115	1	20121022	4
3689883620130315		17343424	19510923	2	20130315	5
36906602	20110214	15089056	19391201	2	20110214	4
36916924	20120116	16073594	19730429	2	20120116	4
36918408	20110609	15428681	19260330	2	20110609	4
36920237	20110227	15126044	19410318	2	20110227	5
36921694	20110921	15730354	19460202	1	20110921	5
36923769	20110108	14993906	19580902	2	20110108	4
36931687	20110306	15150150	19620205	2	20110306	5
36935270	20110918	15719282	19930412	2	20110918	6
36936626	20110412	15257764	19550905	1	20110412	6
36940644	20110320	15192313	19500816	1	20110320	6
36941647	20110316	15184035	19560201	1	20110316	6
36947178	20121024	16908366	19520906	2	20121024	6
3695516520121227		17109282	19630312	2	20121227	4
36955347	20110103	14973457	19671130	1	20110103	4
36958028	20111219	15989920	19230108	2	20111219	4
3696246620130102		17123241	19600517	2	20130102	5
36967007	20121121	16997920	19670912	1	20121121	5
36969865	20120717	16613034	19370328	1	20120717	5
36973587	20110725	15559754	19570221	1	20110725	5
36974466	20110508	15335084	19641110	1	20110508	6
36977192	20110427	15300650	19460816	1	20110427	5
36979154	20110307	15154724	19460501	1	20110307	4
36980720	20110323	15202195	19280206	1	20110323	6
36990815	20121205	17040080	19560730	1	20121205	6
36994420	20101229	14963772	19551201	1	20101229	4
36999890	20110801	15580966	19570523	2	20110801	6
36999969	20110117	15018634	19620112	1	20110117	6

37008676	20110515	15356617	19480429	1	20110515	6
37009840	20120409	16315019	19541219	2	20120409	6
37011022	20110110	14997843	19570220	2	20110110	4
37012627	20110610	15429830	19641104	2	20110610	4
3701286520130707		17693744	19580404	1	20130707	4
37014872	20120729	16645373	19560422	1	20120729	4
37018352	20121127	17013409	19520827	1	20121127	5
37021355	20110824	15650406	19411207	1	20110824	5
3702145720131223		18230087	19530515	2	20131223	5
37021617	20110804	15594099	19630519	2	20110804	4
37021968	20110826	15656101	19501001	1	20110826	5
37021991	20110908	15695340	19570524	2	20110908	4
3703248720130121		17183082	19620901	2	20130121	5
37033399	20111013	15792604	19551001	2	20111013	5
37035760	20111124	15916649	19570115	1	20111124	5
37036752	20110828	15658741	19570524	2	20110828	4
37037415	20110613	15437579	19600224	2	20110613	5
37039751	20111207	15956494	19550420	2	20111207	5
37041284	20110607	15419217	19570926	1	20110607	4
37051539	20120922	16813905	19530101	2	20120922	6
37052236	20111117	15898288	19640331	2	20111117	5
37058267	20110324	15205835	19660626	2	20110324	5
37061226	20111107	15864392	19390213	1	20111107	6
37063380	20110326	15209545	19670221	2	20110326	4
37063675	20110209	15071489	19560715	2	20110209	4
37079199	20120521	16439948	19631031	2	20120521	6
37080947	20110603	15412160	19340601	1	20110603	6
37081473	20121015	16882472	19430819	2	20121015	4
37082307	20110912	15702478	19560728	1	20110912	4
37088565	20120410	16319716	19471231	1	20120410	5
37099288	20110613	15438283	19510725	2	20110613	5
37101818	20111020	15815251	19390929	1	20111020	5
37103278	20110410	15249770	19360606	2	20110410	4
37106460	20110404	15229618	19531212	2	20110404	5
37107792	20110505	15329030	19360513	2	20110505	6
37109845	20121003	16846922	19241003	1	20121003	4
37118040	20110804	15594798	19670406	2	20110804	5
37118744	20110626	15473886	19671205	2	20110626	5
37120675	20110228	15128267	19461025	2	20110228	6
37129229	20111224	16004023	19360101	2	20111224	6
37131707	20110428	15305072	19281228	1	20110428	5
37138399	20110503	15318375	19680105	2	20110503	4
3714756120130320		17357089	19550905	2	20130320	4
3714887120130625		17653890	19690621	1	20130625	5
37155207	20110111	15002544	19580413	2	20110111	5
3715857920130130		17206913	19590123	1	20130130	4
37163443	20110417	15272821	19610212	2	20110417	4
37172773	20120118	16080768	19430317	2	20120118	5
37179581	20110808	15604235	19531205	1	20110808	5
37181569	20111226	16008325	19441114	1	20111226	4
37181821	20111109	15874262	19580101	2	20111109	6
37188446	20120407	16310422	19450213	2	20120407	5
37193514	20120926	16822290	19390420	1	20120926	6
37198702	20111227	16011426	19580812	2	20111227	5
37201482	20121114	16975970	19530325	1	20121114	5

37202645	20110619	15455927	19470426	1	20110619	5
37207811	20111216	15983873	19721216	1	20111216	5
37221662	20111130	15929723	19490819	1	20111130	4
37221935	20110210	15076679	19590310	1	20110210	5
37222972	20131013	18003253	19830810	1	20131013	4
37223748	20130421	17453231	19510720	2	20130421	5
37225040	20120315	16244743	19790811	2	20120315	5
37232943	20130808	17799369	19720323	2	20130808	5
37240087	20130923	17937065	19311015	1	20130923	5
37250876	20110305	15149638	19570309	1	20110305	4
37253513	20110210	15076467	19550325	1	20110210	4
37258870	20120105	16040684	19650225	2	20120105	6
37266210	20130515	17531321	19390505	1	20130515	6
37267940	20120808	16678074	19771020	1	20120808	4
37272585	20120911	16781556	19810411	1	20120911	4
37272949	20111031	15840706	19520217	2	20111031	6
37273168	20121226	17106580	19261103	1	20121226	5
37276429	20110928	15748035	19520408	1	20110928	4
37276703	20120206	16123413	19391027	1	20120206	4
37277864	20111019	15812135	19621025	1	20111019	4
37278798	20110107	14991915	19690508	1	20110107	4
37280287	20130303	17296737	19430503	1	20130303	5
37285248	20120807	16675030	19460112	2	20120807	4
37285668	20101217	14934512	19521010	1	20101217	6
37291728	20101224	14950426	19331220	1	20101224	5
37292969	20110325	15208029	19480621	2	20110325	6
37304657	20130904	17880890	19700918	2	20130904	6
37305547	20101225	14954173	19570510	1	20101225	5
37305785	20120524	16449331	19230116	1	20120524	4
37309094	20110731	15574866	19641123	2	20110731	6
37312520	20110225	15124295	19390822	2	20110225	6
37312871	20120809	16683061	19600810	2	20120809	6
37313512	20110109	14994519	19550128	2	20110109	5
37314253	20121007	16858497	19750228	2	20121007	6
37317854	20110926	15738770	19350927	1	20110926	6
37323356	20120214	16151135	19680918	1	20120214	6
37323765	20120724	16631841	19301122	2	20120724	5
37324917	20110106	14988373	19510613	2	20110106	4
37325625	20111107	15866373	19490822	2	20111107	4
37328942	20110430	15308293	19800807	2	20110430	4
37330555	20110517	15363834	19710711	2	20110517	5
37337090	20120611	16504259	19740605	2	20120611	5
37343649	20111119	15903032	19380803	1	20111119	4
37347221	20111118	15901380	19381120	2	20111118	6
37349681	20110506	15331191	19410512	1	20110506	6
37352593	20130402	17394775	19491215	2	20130402	6
37355672	20110413	15263498	19680720	1	20110413	4
37360591	20130225	17277960	19600701	2	20130225	5
37362757	20120727	16643694	19310301	1	20120727	4
37362940	20110816	15628371	19510227	2	20110816	6
37364651	20120410	16319195	19550411	1	20120410	6
37366248	20110102	14969994	19390905	1	20110102	5
37370459	20110110	14998832	19760221	1	20110110	4
37370904	20110626	15473823	19530103	1	20110626	5
37371021	20130811	17806602	19820404	2	20130811	4

37371032	20120301	16195347	19610528	1	20120301	5
37371098	20110522	15375599	19501011	2	20110522	4
37372422	20110520	15373574	19561210	2	20110520	5
37373458	20110220	15106042	19780212	2	20110220	6
37373696	20101225	14954504	19510815	1	20101225	5
37374111	20110109	14994618	19360515	2	20110109	5
37374188	20120506	16395032	19610124	1	20120506	5
37374382	20120328	16278441	19540905	2	20120328	5
37376515	20110612	15434651	19610917	1	20110612	4
37385094	20110610	15431695	19510404	1	20110610	4
37388015	20111020	15814409	19380802	1	20111020	4
3739039920130617		17628916	19401010	1	20130617	6
37392022	20111211	15966562	19560216	1	20111211	4
37396524	20120503	16388813	19440103	1	20120503	5
3739757220130317		17345697	19470820	1	20130317	4
37403375	20120521	16439610	19540630	1	20120521	6
37407786	20110120	15028742	19440301	2	20110120	6
37419173	20101231	14968705	19410516	1	20101231	6
37419286	20121129	17018781	19571222	1	20121129	6
37421026	20110211	15079766	19570820	2	20110211	4
37423226	20110105	14980439	19330906	2	20110105	4
37424376	20120210	16139926	19680213	2	20120210	5
37429531	20101215	14928419	19430322	1	20101215	5
37431677	20110421	15287671	19410905	2	20110421	4
37432783	20110717	15539199	19460505	1	20110717	4
37435704	20120527	16454870	19500504	1	20120527	5
37439251	20110902	15675763	19740325	1	20110902	5
37450321	20110921	15729207	19570706	1	20110921	5
37453897	20110524	15381099	19521104	1	20110524	4
37456103	20121006	16857836	19460502	2	20121006	6
37460916	20120812	16690452	19530108	2	20120812	6
37465751	20120423	16354351	19481220	2	20120423	5
37474843	20110817	15633304	19570303	2	20110817	6
37475255	20110305	15149599	19421111	2	20110305	4
37477922	20110518	15364427	19671126	1	20110518	4
37478196	20111022	15819377	19510312	2	20111022	5
37483333	20120327	16274862	19680930	1	20120327	6
3748741520130129		17205302	19300409	2	20130129	6
37487551	20110110	14997830	19811025	2	20110110	5
37488985	20110228	15127225	19561004	1	20110228	5
37489284	20110222	15114030	19600226	2	20110222	6
3749232320131209		18188140	19450213	1	20131209	4
37493860	20121125	17006671	19160522	2	20121125	5
37496610	20110626	15473909	19391210	1	20110626	5
37500860	20110825	15654066	19360806	1	20110825	5
37505241	20120530	16462623	19531108	2	20120530	6
37508466	20110303	15143160	19510926	1	20110303	6
3751132320131124		18137795	19490612	1	20131124	5
37521189	20110221	15110129	19460204	1	20110221	5
37527529	20110128	15049015	19780626	1	20110128	6
37528715	20110331	15221819	19521001	1	20110331	4
3753607520131110		18094902	19651230	2	20131110	5
37537216	20120404	16299666	19560821	2	20120404	6
37537307	20110303	15143901	19501102	1	20110303	5
37538082	20111104	15860188	19590320	2	20111104	6

37539289	20130513	17523387	19560907	1	20130513	6
37547538	20110614	15442335	19661115	2	20110614	6
37553530	20110127	15046481	19420715	1	20110127	6
37559630	20110301	15134524	19591206	1	20110301	5
37559787	20110602	15407265	19721206	2	20110602	5
37560922	20110102	14969859	19541011	1	20110102	4
37562495	20131102	18066135	19350402	1	20131102	6
37562633	20131014	18005422	19761107	2	20131014	5
37564117	20130502	17489231	19701009	1	20130502	5
37569349	20120820	16715483	19610318	1	20120820	5
37571258	20110816	15629281	19411023	2	20110816	4
37574542	20120429	16369590	19431118	2	20120429	5
37576888	20110107	14989437	19421020	1	20110107	6
37578680	20110410	15249785	19491113	2	20110410	6
37578920	20120710	16592125	19710403	2	20120710	6
37578986	20110622	15466403	19391003	1	20110622	5
37583225	20111216	15984221	19200115	1	20111216	4
37583689	20110107	14991459	19370901	2	20110107	5
37584002	20110805	15597996	19360209	2	20110805	5
37588822	20110531	15396581	19511101	2	20110531	4
37590708	20130424	17463782	19440314	1	20130424	4
37591201	20101224	14953204	19820927	2	20101224	5
37592544	20110324	15205630	19830709	1	20110324	4
37592704	20101126	14869165	19340715	1	20101126	4
37595043	20120513	16417627	19430802	1	20120513	6
37597312	20130708	17697521	19511115	1	20130708	4
37598020	20120704	16572473	19491019	1	20120704	4
37598177	20121223	17097490	19690727	1	20121223	5
37598257	20120408	16310970	19620210	1	20120408	6
37605202	20121205	17041562	19870316	1	20121205	4
37612354	20130314	17340012	19620308	1	20130314	6
37613971	20110409	15249373	19581209	1	20110409	6
37623908	20110323	15202815	19501123	2	20110323	5
37624923	20130908	17893966	19310924	1	20130908	4
37626394	20121208	17052492	19630119	1	20121208	6
37633617	20100919	14671053	19640311	1	20100919	6
37634665	20110611	15434050	19490429	2	20110611	6
37636503	20120227	16184881	19550514	1	20120227	5
37636683	20101213	14920407	19550825	1	20101213	6
37640269	20121111	16963637	19610811	1	20121111	6
37652509	20110307	15154422	19381110	2	20110307	5
37655633	20110412	15258502	19301009	1	20110412	5
37660336	20120206	16123867	19441208	2	20120206	6
37661453	20111020	15815409	19510330	1	20111020	4
37679371	20110524	15379990	19310420	2	20110524	4
37692710	20110212	15084156	19571010	2	20110212	4
37692981	20110915	15713575	19671219	1	20110915	4
37693600	20110627	15476364	19510618	2	20110627	6
37696212	20110712	15526166	19470714	1	20110712	5
37698478	20130825	17847152	19640918	1	20130825	5
37702242	20110406	15237396	19700114	2	20110406	4
37704522	20120707	16583870	19370928	2	20120707	4
37707565	20110220	15106271	19490330	2	20110220	6
37713216	20110207	15062196	19620615	1	20110207	6
37720459	20110411	15253589	19560122	1	20110411	6

3772058420130220		17264359	19660509	2	20130220	6
37720777	20110210	15078172	19430403	1	20110210	4
37726684	20110529	15392158	19361117	2	20110529	5
37726764	20110314	15175472	19810722	2	20110314	4
37727278	20111026	15830331	19450701	1	20111026	5
3772896420130305		17305985	19360516	1	20130305	6
37736235	20110523	15378138	19740205	1	20110523	5
3773635920130217		17250461	19800113	2	20130217	6
37737078	20110306	15150089	19461124	1	20110306	5
37742511	20120326	16270948	19411027	2	20120326	4
3774655720130128		17203029	19570825	1	20130128	4
37749238	20101227	14956326	19450521	1	20101227	4
37749352	20110515	15356592	19640707	1	20110515	5
37749738	20110418	15274312	19751215	1	20110418	4
37754066	20111226	16008331	19680229	1	20111226	6
3775535420130905		17886858	19430420	1	20130905	4
37758648	20110523	15378556	19590602	1	20110523	5
37762906	20110110	14998508	19430618	1	20110110	6
37764333	20121114	16974551	19621226	1	20121114	5
3776946320121212		17066398	19520220	1	20121212	6
37770288	20120603	16474569	19410125	1	20120603	6
37773958	20110115	15014233	19410515	2	20110115	5
37774360	20110418	15276388	19491012	1	20110418	5
37780726	20110420	15284241	19571230	1	20110420	6
37788786	20110326	15209808	19610119	1	20110326	4
37791007	20110102	14969819	19271016	2	20110102	6
37792431	20110308	15159116	19360923	1	20110308	4
37793638	20110103	14974537	19580818	1	20110103	5
37794799	20110111	15001859	19470103	1	20110111	6
3779748120130117		17174811	19530901	2	20130117	6
3779898420130717		17724632	19431008	1	20130717	4
37799976	20110501	15309247	19390204	2	20110501	4
37800296	20110116	15014560	19590112	2	20110116	6
37800741	20110122	15033287	19500227	2	20110122	4
37803886	20110206	15060165	19710205	1	20110206	5
37804470	20110901	15671331	19791007	1	20110901	5
3780858520130512		17519256	19461202	1	20130512	5
37814485	20101228	14961376	19730415	2	20101228	5
3781635620130806		17790976	19650511	2	20130806	5
37816696	20120109	16050321	19601113	2	20120109	6
37817122	20110612	15434500	19271018	1	20110612	4
37818567	20110510	15343906	19510222	2	20110510	5
37818647	20110314	15175822	19391118	2	20110314	5
37820136	20120901	16745545	19291216	1	20120901	6
37821695	20121101	16929089	19560225	2	20121101	6
37823919	20110224	15119833	19860919	1	20110224	5
37825197	20110109	14994464	19411214	2	20110109	6
37825471	20110125	15040493	19660901	2	20110125	6
37826690	20101228	14961365	19521110	1	20101228	6
37831791	20110612	15434629	19441119	1	20110612	4
3783338820131210		18193768	19580510	2	20131210	5
37835748	20120521	16439985	19910713	1	20120521	5
3784105720131031		18058648	19290520	2	20131031	5
37842061	20111018	15807906	19380717	2	20111018	6
37843508	20120423	16354698	19310720	2	20120423	6

37850934	20110102	14969531	19570406	1	20110102	5
37851051	20110424	15292339	19520116	1	20110424	5
37853364	20110207	15060692	19630712	1	20110207	6
37854107	20110207	15062679	19550413	1	20110207	4
37857300	20110627	15476748	19590705	1	20110627	4
37858596	20110404	15231093	19591010	1	20110404	5
37859806	20110405	15232155	19431209	1	20110405	4
3786539720121221		17094029	19390825	2	20121221	6
37866890	20110208	15066195	20090129	1	20110208	5
37869004	20111008	15781631	19250504	1	20111008	4
37873475	20110630	15485936	19400409	1	20110630	5
37874070	20100912	14650601	19591228	1	20100912	6
37876292	20101228	14959255	19480607	1	20101228	5
37877579	20110103	14972743	19480929	1	20110103	5
37879042	20110123	15034045	19510605	1	20110123	4
37880969	20111210	15965949	19710707	1	20111210	5
37883242	20120524	16449422	19370708	2	20120524	6
37885271	20101211	14915853	19680426	1	20101211	5
37889013	20110315	15179659	19560823	1	20110315	5
37890383	20110303	15142352	19370418	1	20110303	5
37891115	20110106	14987506	20050331	2	20110106	6
37891637	20110326	15209794	19420222	1	20110326	5
37893508	20110314	15174975	19491204	1	20110314	4
37896063	20101117	14844634	19570816	1	20101117	5
37902060	20110530	15394652	19740618	2	20110530	6
37902899	20110530	15394594	19560108	2	20110530	6
37904066	20110218	15102749	19511004	2	20110218	4
37909425	20110124	15037243	19620627	2	20110124	4
37910342	20110101	14969094	19550504	1	20110101	6
37911798	20110301	15133402	19770116	2	20110301	5
37912713	20110119	15025587	19740717	1	20110119	6
37912962	20120920	16808178	19360201	1	20120920	5
37913341	20101125	14866410	19550130	1	20101125	5
37917127	20111230	16020668	19360105	1	20111230	4
37917627	20110909	15697324	19320129	1	20110909	6
37923118	20111106	15862669	19360327	2	20111106	4
37924166	20110307	15154919	19550206	2	20110307	5
37925590	20110305	15147931	19670801	2	20110305	6
37929707	20110814	15621925	19440401	1	20110814	5
37931376	20110113	15009110	19450512	1	20110113	6
37931569	20120716	16609621	19571120	1	20120716	5
37932993	20110204	15058684	19860320	1	20110204	6
37936406	20120612	16505437	19730323	2	20120612	5
37937954	20110103	14974972	19510713	1	20110103	6
37938684	20110104	14977922	19560901	1	20110104	6
3794063920131024		18040476	19841001	2	20131024	6
3794129020130421		17453225	19501020	1	20130421	4
37943536	20110214	15088327	19630212	1	20110214	4
37943694	20121107	16951957	19410217	1	20121107	6
37946240	20110427	15302179	19300304	1	20110427	6
3794674020130826		17850977	19510218	2	20130826	6
37948315	20101224	14950594	19500607	2	20101224	5
37949501	20101228	14961018	19541020	1	20101228	5
37950882	20101224	14952869	19300116	2	20101224	6
37951125	20101209	14910931	19390228	1	20101209	5

3795116920130705		17688173	19880616	1	20130705	5
37953790	20110324	15205596	19740112	2	20110324	5
37954362	20111209	15963461	19511222	1	20111209	5
37954862	20110810	15611499	19731011	2	20110810	6
37955683	20111005	15770209	19590112	2	20111005	6
37955796	20121031	16924269	19680606	2	20121031	6
37963534	20110626	15473891	19910211	1	20110626	4
37964355	20110820	15640622	19530501	1	20110820	4
37965201	20110307	15154384	19510330	1	20110307	4
3796699720130122		17188320	19620212	1	20130122	5
37967581	20110316	15184016	19620302	2	20110316	6
37968700	20110213	15084794	19461011	1	20110213	5
37970733	20110414	15267044	19631124	1	20110414	6
37974268	20120410	16318582	19511202	2	20120410	6
37977041	20110320	15192214	19751210	1	20110320	6
3797724520130415		17435461	19321101	1	20130415	4
37979741	20110425	15295963	19390828	1	20110425	4
3798086220131017		18016233	19450725	1	20131017	5
37983292	20110306	15150113	19570405	1	20110306	5
37984148	20110222	15113409	19631004	2	20110222	5
37986962	20110303	15139837	19471219	2	20110303	6
37991405	20110411	15254301	19440715	1	20110411	6
37994813	20110413	15263625	19310123	1	20110413	5
37994915	20110410	15249945	19281201	2	20110410	5
37995112	20101212	14916253	19720903	1	20101212	6
37999125	20110129	15050638	19361017	1	20110129	4
37999603	20110127	15046641	19621008	2	20110127	5
38001353	20110312	15172094	19560310	1	20110312	6
38006701	20110324	15204933	19690302	2	20110324	4
38008116	20110322	15199642	19340703	1	20110322	5
38008569	20110411	15254995	19450502	2	20110411	5
38008810	20110328	15213073	19480404	1	20110328	6
38015677	20121203	17030282	19290522	1	20121203	4
38022490	20120410	16319370	19490401	2	20120410	4
38023846	20110409	15249458	19530117	1	20110409	6
38024203	20110403	15228931	19211021	1	20110403	6
38025977	20101230	14964622	19590208	1	20101230	6
38026561	20101231	14967736	19481201	1	20101231	5
38026867	20110305	15149370	19490101	1	20110305	4
38027735	20101230	14965826	19590701	2	20101230	6
38027928	20110116	15014739	19640516	1	20110116	4
38029322	20110102	14969674	19760421	2	20110102	6
3803120820131222		18228140	19461209	2	20131222	6
38032698	20110102	14969766	19681010	2	20110102	4
38034478	20110117	15018529	19540530	1	20110117	5
38034581	20110128	15049044	19900329	2	20110128	5
38034650	20110103	14973783	19521013	2	20110103	4
38035062	20101224	14952680	19580913	1	20101224	5
38035084	20110301	15132589	19520819	1	20110301	4
38036736	20101229	14964117	19360620	2	20101229	4
3803955320130221		17268152	19360511	1	20130221	4
38044109	20110110	14997667	19750616	2	20110110	5
38044370	20111022	15819966	19391028	1	20111022	4
38045431	20110119	15024655	19600112	2	20110119	4
38048032	20110106	14988050	19740323	2	20110106	4

38048098	20120104	16032928	19960314	2	20120104	6
38048134	20110214	15087936	19940417	2	20110214	5
38050587	20110213	15084466	19740210	1	20110213	5
38050736	20110308	15158471	19340708	2	20110308	4
38052061	20110223	15115076	19571028	2	20110223	5
38054205	20110530	15395098	19300104	2	20110530	5
38055491	20120531	16465547	19870917	2	20120531	5
38055957	20110522	15375479	19760924	2	20110522	4
38056121	20110314	15174790	19410505	1	20110314	4
38056676	20110322	15199402	19560906	2	20110322	6
38057839	20110321	15195543	19700529	1	20110321	4
38058150	20110404	15231319	19490105	1	20110404	4
38058763	20120919	16805970	19820318	2	20120919	4
38063080	20110123	15033888	19630615	2	20110123	6
38063342	20110119	15024082	19390814	2	20110119	5
38064834	20110125	15040395	19690810	2	20110125	4
38071271	20110212	15083869	19580711	1	20110212	4
38072263	20111004	15765194	19521103	1	20111004	5
38074623	20110821	15641210	19450125	1	20110821	5
38074907	20110216	15095085	19690808	2	20110216	5
38076334	20111011	15787271	19860424	2	20111011	5
38076378	20110530	15393994	19700114	2	20110530	5
38077519	20110226	15125676	19691014	2	20110226	6
38078216	20110215	15090262	19350709	2	20110215	4
38078523	20111023	15820509	19610228	2	20111023	4
38084376	20110208	15066489	19540522	2	20110208	5
38087353	20110208	15065681	19600512	2	20110208	4
38088663	20110327	15210371	19541225	1	20110327	5
38093719	20101207	14902307	19261226	1	20101207	6
38096558	20101216	14931228	19360225	1	20101216	5
38097346	20110220	15106308	20100420	1	20110220	5
38099706	20110513	15354726	19480515	1	20110513	5
38113063	20110201	15056456	20001105	1	20110201	5
38113290	20120510	16411726	19600206	2	20120510	4
38113972	20110324	15204822	19300923	2	20110324	4
38114000	20110220	15106189	19471201	1	20110220	4
38115263	20110608	15424240	19420901	1	20110608	5
38116879	20110220	15106212	19401214	2	20110220	6
38118079	20110324	15204453	19571023	2	20110324	6
38120217	20110301	15130919	19481202	2	20110301	6
38120751	20110214	15086580	19751230	1	20110214	5
38121378	20110214	15088915	19590501	1	20110214	6
38122746	20110510	15343918	19581227	2	20110510	6
38123307	20120201	16107099	19500110	1	20120201	6
38123545	20110223	15117614	19570607	2	20110223	5
38124571	20110214	15087925	19480304	1	20110214	5
38125869	20110807	15600830	19670815	2	20110807	6
38126384	20110217	15099856	19470815	1	20110217	5
38126475	20110215	15093116	19970226	2	20110215	5
38128108	20110529	15392146	19350711	2	20110529	4
38128379	20110220	15106030	19410822	1	20110220	6
38129372	20110314	15176233	19441110	2	20110314	5
38129883	20110222	15111285	19441127	1	20110222	6
3813042620130225		17277346	19700625	2	20130225	6
38131725	20110314	15176783	19520305	1	20110314	5

38131918	20120317	16250104	19400701	2	20120317	4
38132002	20110418	15276973	19431118	2	20110418	6
38132422	20110508	15335156	19550328	2	20110508	4
38132557	20110509	15337226	19340828	1	20110509	4
38132900	20110520	15373419	19700803	1	20110520	6
38132977	20130101	17117822	19300521	1	20130101	5
38133038	20110323	15201500	19491101	1	20110323	4
38133072	20110320	15192420	19570212	1	20110320	5
38133334	20110621	15461863	20070416	1	20110621	5
38133572	20110414	15266032	19520515	2	20110414	4
38133652	20110620	15459457	19580407	1	20110620	4
38133812	20110420	15281970	19490124	2	20110420	6
38133925	20110425	15295743	19571004	1	20110425	4
38134053	20110612	15434634	19690727	2	20110612	6
38134155	20110523	15379015	19570205	1	20110523	5
38134235	20110529	15391918	19481213	2	20110529	5
38134371	20110601	15403719	19611201	2	20110601	6
38134531	20110531	15397156	19390701	2	20110531	5
38135589	20110228	15128216	19540319	2	20110228	5
38138953	20111124	15915218	19440214	1	20111124	6
38139489	20110307	15153431	19510826	1	20110307	5
38139592	20111111	15880177	19550807	1	20111111	4
38139741	20110405	15233955	19470221	2	20110405	6
38143509	20110426	15299236	19530528	1	20110426	5
38143963	20110327	15210324	19511017	2	20110327	6
38145196	20110228	15127943	19680614	1	20110228	5
38146188	20110509	15339814	19471115	2	20110509	6
38148297	20110302	15136189	19760122	1	20110302	5
38148593	20110306	15150201	19490415	2	20110306	5
38149529	20110307	15154940	19640704	1	20110307	5
38152986	20110108	14993802	19311212	1	20110108	4
38158382	20110504	15324310	19790312	1	20110504	6
38162060	20110414	15266650	19850228	1	20110414	6
38164362	20110518	15363897	19560803	1	20110518	6
38168455	20110505	15328575	19670121	2	20110505	5
38171505	20110301	15133886	19650910	1	20110301	5
38172520	20110311	15169483	19520123	1	20110311	4
38172882	20110320	15192336	19540326	1	20110320	5
38176271	20110308	15158336	19630202	1	20110308	6
38177514	20110323	15202377	19350929	1	20110323	5
38178697	20110313	15172422	19560326	1	20110313	5
38178960	20131011	17999986	19380308	2	20131011	5
38179601	20110413	15263572	19481001	1	20110413	5
38180233	20130521	17548423	19520908	2	20130521	4
38181167	20110310	15163230	19641104	2	20110310	4
38181805	20111023	15820584	19410610	1	20111023	5
38182364	20110507	15334417	19460309	1	20110507	6
38182466	20110620	15458723	19610928	1	20110620	5
38183969	20120109	16050723	19830129	2	20120109	5
38184597	20110317	15186675	19560818	2	20110317	6
38185318	20110405	15233914	19810328	1	20110405	5
38187450	20111002	15756478	19511228	2	20111002	6
38187596	20110310	15166332	19470428	2	20110310	5
38187701	20110310	15165991	19410418	2	20110310	4
38189310	20110328	15213654	19480410	1	20110328	5

38189627	20110310	15164682	19360604	2	20110310	6
38193510	20110414	15267012	19480917	1	20110414	5
3819394120130605		17591911	19390128	1	20130605	6
38195663	20110405	15233499	19780315	2	20110405	5
38199018	20110417	15272656	19980227	2	20110417	6
38205491	20110405	15233888	19810916	2	20110405	6
38207260	20110502	15313266	20010525	2	20110502	5
38207793	20120101	16022735	19560501	1	20120101	5
38208069	20110412	15258223	19801004	2	20110412	4
38208536	20110418	15275778	19580623	1	20110418	5
38208741	20110413	15263587	19631011	2	20110413	4
38209391	20110515	15356468	19590818	1	20110515	6
38210901	20110412	15259139	19471209	2	20110412	5
38211266	20110412	15258238	19450114	2	20110412	4
38212667	20110411	15252320	19590713	2	20110411	5
38213568	20110405	15233430	19941201	2	20110405	5
38214992	20110417	15272586	19560102	2	20110417	6
38215155	20110407	15242453	19700531	2	20110407	6
38217504	20110419	15279464	19790927	1	20110419	5
38217537	20110823	15646820	19600605	1	20110823	5
38218881	20110418	15276938	19940317	1	20110418	5
38219613	20110502	15315298	19661201	1	20110502	5
3821963520131109		18094451	19570709	2	20131109	6
38219997	20110501	15309101	19330915	2	20110501	4
38224338	20110416	15272276	19580410	2	20110416	4
38224963	20110419	15280136	19711108	2	20110419	5
3822642520130813		17813172	19400525	2	20130813	6
38231515	20110430	15308673	19610607	1	20110430	4
38232063	20110426	15299235	19480131	2	20110426	5
38232778	20111221	15994965	19511021	2	20111221	4
38234376	20121030	16922194	19380517	1	20121030	6
38235506	20110504	15324853	19290901	1	20110504	5
38238629	20110428	15304675	19541113	1	20110428	5
38243651	20110425	15296037	19790218	1	20110425	4
38245715	20110428	15304601	19551213	1	20110428	5
38246218	20110430	15308576	19290906	1	20110430	5
38247686	20110621	15462647	19901107	2	20110621	5
38247926	20110506	15331662	19650819	1	20110506	6
38248985	20110507	15334598	19320330	1	20110507	5
38250565	20110209	15071883	19510104	2	20110209	4
38250576	20110209	15073302	19361001	2	20110209	5
38256198	20110426	15298128	19840727	1	20110426	6
38256994	20110516	15359181	19721021	1	20110516	6
38257146	20110425	15295636	19620418	1	20110425	6
38258070	20110427	15302428	19620721	2	20110427	6
38261288	20110502	15315291	19580501	1	20110502	4
38261664	20110516	15359504	19620312	1	20110516	5
38271135	20111018	15807613	19560315	1	20111018	6
38271475	20120108	16047446	19520616	1	20120108	6
38272898	20110517	15362165	19291027	1	20110517	4
38273153	20110602	15405353	19860721	2	20110602	4
38273460	20110523	15379103	19430712	1	20110523	4
38282358	20110531	15396328	19330428	1	20110531	5
38284525	20110724	15558018	19121210	1	20110724	5
38285302	20110531	15397001	19530428	1	20110531	5

38287353	20110607	15418739	19580905	2	20110607	6
38287546	20110512	15351504	19440703	2	20110512	5
38287808	20110516	15358704	19390407	1	20110516	5
38289713	20110526	15387206	19571102	2	20110526	4
38291279	20110319	15191938	19671001	1	20110319	6
38291951	20110302	15138822	19580918	1	20110302	6
38292545	20110304	15146772	19970326	1	20110304	5
38295497	20110319	15191995	19511220	1	20110319	5
38296401	20110316	15184213	19600704	2	20110316	4
38296592	20110317	15186565	19340214	2	20110317	5
38298350	20110325	15208446	19580110	2	20110325	6
38298429	20110324	15205864	19380722	1	20110324	4
38298781	20110328	15212588	19460106	2	20110328	5
38300162	20110612	15434635	19510728	2	20110612	6
38300311	20110505	15326118	19560810	1	20110505	4
38300979	20111212	15970555	19611122	2	20111212	6
38302680	20111215	15981107	19811030	2	20111215	5
38304233	20110510	15343182	19521026	2	20110510	5
38308257	20110511	15346410	19420316	2	20110511	5
38308280	20110529	15392139	19320211	1	20110529	5
38309261	20110515	15356690	19520805	2	20110515	4
38310111	20110530	15394412	19581006	2	20110530	6
38310291	20110512	15351064	19281201	1	20110512	6
38310406	20110521	15375082	19550308	2	20110521	6
38310531	20110518	15367094	19640417	1	20110518	4
38311965	20110525	15384704	19481124	1	20110525	4
38312833	20110523	15378589	19710926	1	20110523	6
38313472	20110522	15375346	19240103	2	20110522	5
38314862	20110516	15359722	19360903	1	20110516	4
38315569	20110617	15453239	19641007	2	20110617	4
38316506	20110621	15462388	19841019	2	20110621	5
38317656	20120207	16128380	19551012	2	20120207	4
38318422	20110520	15373503	19571010	2	20110520	6
38320068	20110518	15367352	19510707	1	20110518	5
38320648	20110628	15479157	19430502	2	20110628	4
38324117	20110524	15381944	19590106	2	20110524	5
38325303	20110608	15423905	19590502	2	20110608	5
38326248	20110809	15607758	19580516	1	20110809	5
38328971	20110612	15434722	19430306	2	20110612	5
38335114	20120627	16547938	19541125	2	20120627	5
38335658	20121022	16902959	19900723	2	20121022	6
38337778	20110808	15604044	19260102	2	20110808	4
38340088	20110331	15221451	19510727	2	20110331	6
38341865	20110404	15230981	19290712	2	20110404	5
38342971	20110413	15263604	19460918	2	20110413	5
38345072	20110411	15253588	19720217	1	20110411	5
38345389	20110411	15254743	19411008	1	20110411	5
38346951	20110424	15292234	19430203	2	20110424	5
38348253	20110422	15289605	19481210	1	20110422	4
38350571	20110529	15391998	19620325	1	20110529	6
38350593	20110523	15376642	19430701	1	20110523	4
38350811	20120306	16212816	19881120	2	20120306	4
38351427	20110629	15482017	19500801	1	20110629	4
38353503	20120815	16702089	19560130	1	20120815	6
38357254	20111128	15923625	19350925	1	20111128	5

38359738	20110605	15414514	19641117	1	20110605	4
38361045	20110801	15580970	19530406	2	20110801	6
38362651	20110619	15455951	19620530	2	20110619	4
38365467	20110616	15450658	19451230	1	20110616	4
38367009	20110828	15658825	19670320	1	20110828	6
38368386	20110904	15678836	19390930	1	20110904	6
38375245	20110609	15428483	19630409	2	20110609	6
38375643	20110616	15447824	19730224	1	20110616	6
38376124	20110603	15410954	19540324	2	20110603	5
38376588	20110621	15462836	19311106	1	20110621	5
38377478	20110607	15419116	19620502	1	20110607	5
38379101	20110621	15463108	19411115	2	20110621	5
38379598	20111019	15811210	19580915	2	20111019	4
38380891	20110504	15323404	19411231	2	20110504	6
38382693	20110612	15434526	19940620	1	20110612	5
38385227	20110609	15426804	19420212	1	20110609	5
38387723	20110622	15466458	19491120	1	20110622	4
38388964	20110616	15449729	19721104	2	20110616	6
38392471	20110615	15446227	19400624	1	20110615	5
38395243	20110627	15476651	19420828	2	20110627	5
38396177	20111116	15894734	19710406	1	20111116	5
38397830	20110817	15631781	19511227	1	20110817	4
38398322	20110803	15590449	19580925	2	20110803	5
38401693	20110623	15469570	19680606	2	20110623	4
38401762	20111122	15910455	19371129	1	20111122	6
38402152	20110626	15473697	19591030	1	20110626	4
38402878	20110630	15485022	19780302	1	20110630	5
38405173	20110507	15334617	19351028	2	20110507	4
38406063	20110629	15481371	19520810	1	20110629	6
38406325	20120120	16086759	19370505	1	20120120	6
38408956	20110429	15307171	19530525	2	20110429	5
38412781	20110517	15360368	19430110	2	20110517	4
38416067	20110627	15476086	19541010	1	20110627	4
38416590	20110626	15473966	19460301	1	20110626	5
38416727	20110629	15482788	19421012	1	20110629	6
38416761	20110630	15484691	19230323	2	20110630	6
38417219	20110618	15455211	19681106	2	20110618	6
38417899	20110628	15479320	19661010	2	20110628	4
38418358	20110618	15455227	19410216	2	20110618	6
38421839	20111001	15756147	19460118	2	20111001	4
38422707	20110614	15443168	19790206	1	20110614	5
3842363120130511		17518708	19510420	1	20130511	4
38423880	20110718	15541774	19930214	1	20110718	4
38426038	20110617	15453262	19450412	2	20110617	6
38427611	20110626	15473738	19400102	1	20110626	4
38427655	20110624	15472277	19340716	2	20110624	5
38428772	20110715	15536037	19460927	1	20110715	5
38428783	20120821	16717945	19720703	2	20120821	5
38429275	20110703	15493799	19351001	1	20110703	5
38432881	20110829	15661677	19420714	1	20110829	6
38433099	20110629	15482126	19370305	1	20110629	4
38435277	20110626	15473985	19440125	2	20110626	5
38435551	20110622	15465672	19580128	1	20110622	5
38435755	20110717	15539091	19221112	2	20110717	4
38436930	20111011	15786716	19500713	2	20111011	5

38451659	20110713	15528737	19570413	1	20110713	5
38452129	20120916	16794339	19500103	2	20120916	6
38455606	20111101	15845273	19540228	2	20111101	4
38457226	20110721	15553070	19540627	1	20110721	5
38459299	20111112	15882762	19360125	1	20111112	5
38461335	20110825	15654488	19460906	2	20110825	5
38470370	20110623	15467557	19650501	1	20110623	5
38470585	20110626	15473708	19440327	2	20110626	6
38471226	20110802	15583849	19511106	1	20110802	5
38471588	20110725	15560609	19531124	2	20110725	5
38473619	20110919	15721036	19370713	1	20110919	4
38473631	20110815	15624685	19600709	2	20110815	6
38476992	20110629	15482508	19991102	2	20110629	6
38477291	20131225	18237594	19580112	1	20131225	4
38478169	20110628	15479606	19610510	2	20110628	6
38479037	20110628	15480234	19710713	1	20110628	6
38480078	20110813	15621837	19860622	2	20110813	5
38483602	20120603	16474605	19610215	2	20120603	4
38485095	20110522	15375399	19691203	1	20110522	5
38485722	20111228	16015590	19470720	2	20111228	5
38486816	20110527	15390424	19830218	1	20110527	4
38487115	20110608	15423356	19500616	2	20110608	6
38487239	20110529	15391875	19641208	1	20110529	4
38492023	20130715	17720233	19641028	2	20130715	6
38493377	20110615	15445173	19580906	2	20110615	5
38495577	20110630	15486015	19711204	1	20110630	6
38497619	20120502	16384249	19500926	1	20120502	6
38498827	20110905	15683207	19311231	1	20110905	4
38498963	20120226	16182755	19640411	1	20120226	6
38500128	20111120	15903552	19561108	2	20111120	6
38502328	20110727	15567869	19771125	1	20110727	6
38503467	20110728	15570183	19400204	1	20110728	4
38504595	20110926	15741907	19300515	1	20110926	5
38505394	20110825	15654379	19630110	2	20110825	6
38506411	20110801	15579539	19501025	2	20110801	6
38506795	20110812	15619757	19470525	1	20110812	5
38507572	20110921	15729051	19611010	2	20110921	6
38508133	20111111	15880866	19450314	1	20111111	5
38508622	20121125	17006497	19320917	2	20121125	5
38508951	20110811	15616186	19661031	1	20110811	5
38509136	20111229	16018320	19500302	2	20111229	6
38510666	20130306	17312083	19481117	1	20130306	6
38516517	20110701	15490032	19570208	1	20110701	5
38516631	20120205	16119833	19301006	1	20120205	4
38519845	20110714	15531583	20090825	2	20110714	5
38521356	20110621	15461850	19881209	1	20110621	5
38522779	20111202	15940812	19470922	1	20111202	5
38523103	20110716	15538694	19560702	1	20110716	6
38524128	20130602	17578449	19870810	2	20130602	6
38524515	20120318	16250359	19370116	2	20120318	6
38524695	20110721	15552240	19661216	1	20110721	5
38525110	20110807	15600818	19830423	2	20110807	4
38527343	20110711	15521826	19441223	2	20110711	4
38528506	20110819	15638106	19510802	2	20110819	4
38528686	20130808	17798467	19250128	1	20130808	5

38530335	20110724	15557982	19710731	2	20110724	6
38530437	20110720	15550005	19280116	2	20110720	6
38533390	20110907	15691681	19550707	2	20110907	6
38534917	20110715	15537101	19510812	2	20110715	5
38543601	20110720	15549316	19951012	1	20110720	6
38545710	20110814	15622117	19520909	2	20110814	4
38545798	20110831	15666439	19660430	1	20110831	5
38547590	20110808	15605022	19550320	2	20110808	5
38547669	20110821	15641119	19910814	2	20110821	4
38547932	20110918	15719254	19460504	2	20110918	4
38549756	20110818	15635691	19510228	2	20110818	6
38551336	20110809	15607311	19680424	2	20110809	5
38555349	20110828	15658957	19691105	2	20110828	6
38555543	20110815	15625406	19920129	2	20110815	6
38556455	20110903	15678196	19811114	2	20110903	6
38557312	20110818	15635002	19530126	2	20110818	5
38557594	20110923	15735786	19550211	2	20110923	5
38557856	20110911	15701936	19390226	2	20110911	6
38558326	20110922	15733134	19670714	2	20110922	4
38558940	20110917	15718720	19560102	1	20110917	6
38559330	20110918	15719388	19510502	2	20110918	5
38559512	20110911	15701929	19550519	1	20110911	5
38561170	20110731	15575058	19290915	1	20110731	4
38563267	20110807	15600778	19611126	1	20110807	6
38563938	20110802	15585422	19391020	2	20110802	6
38566095	20110727	15566449	19700320	2	20110727	6
38568579	20120316	16247901	19400902	1	20120316	5
38568740	20110728	15570094	19501106	1	20110728	6
38571016	20110802	15585412	19560316	2	20110802	5
38571458	20110825	15654156	19300525	1	20110825	6
38572053	20110731	15575114	19280113	1	20110731	5
38572473	20110823	15648075	19810728	2	20110823	5
38572906	20110814	15622077	19331202	1	20110814	6
38575278	20110808	15603935	19570805	2	20110808	6
38575712	20120423	16355162	19960630	1	20120423	6
38576248	20110808	15603554	19570318	1	20110808	4
38577741	20110805	15598520	19301214	2	20110805	6
38578095	20110804	15591753	19671126	1	20110804	4
38579394	20110808	15603936	19641218	1	20110808	6
38579929	20110820	15640684	19560707	1	20110820	6
38580982	20110818	15635180	19770318	2	20110818	5
38582193	20110830	15664123	19530225	2	20110830	4
38583878	20110927	15744093	19320409	2	20110927	6
38584699	20111116	15894833	19330301	1	20111116	6
38586673	20111017	15803454	19340714	1	20111017	4
38586731	20111213	15973724	19800628	1	20111213	6
38586811	20111020	15815217	19540905	2	20111020	6
38588577	20111125	15919846	19401001	1	20111125	6
38588828	20111023	15820437	19690530	1	20111023	5
38595061	20110823	15648012	19640605	1	20110823	6
38595732	20110829	15661681	19541222	2	20110829	5
38596008	20110925	15738291	19570309	2	20110925	4
38601897	20110928	15747110	19510119	2	20110928	5
38602458	20110919	15722785	19390102	1	20110919	5
38602594	20111024	15823404	19470626	1	20111024	5

38603713	20111020	15812563	19560920	1	20111020	6
38605242	20111011	15786509	19541002	2	20111011	5
38606085	20120604	16479167	19380803	1	20120604	4
38607408	20120321	16259380	19461022	1	20120321	4
38607613	20120410	16318976	19621111	2	20120410	5
38607668	20120426	16364161	19820220	2	20120426	4
3860790820131105		18075609	19650428	2	20131105	5
38608627	20120920	16809102	19370929	2	20120920	5
3860969720130221		17268092	19280208	2	20130221	4
3860998220130416		17439219	19681230	2	20130416	4
38612827	20111115	15890394	19540725	1	20111115	4
38615100	20110723	15557395	19270828	2	20110723	6
38618132	20110815	15622466	19270405	1	20110815	6
3862052920130308		17321593	19480301	1	20130308	4
38620814	20111101	15845865	19460109	1	20111101	5
38620949	20110815	15624875	19341108	1	20110815	6
38621022	20110831	15666291	19361228	2	20110831	5
38622070	20110819	15638437	19680116	1	20110819	6
38624918	20110907	15691540	19420412	2	20110907	5
38625046	20110824	15650039	19350312	1	20110824	5
38625240	20120426	16364232	19450610	1	20120426	5
38625579	20110821	15641319	19401219	1	20110821	5
38625671	20110817	15631924	20010328	2	20110817	4
38625728	20110819	15638261	19651007	1	20110819	5
38625842	20110824	15650970	19330125	2	20110824	5
38625911	20110826	15656013	19391129	1	20110826	4
38626061	20120104	16036723	19601102	1	20120104	5
38626118	20120105	16041127	19560328	2	20120105	6
38626721	20120209	16135673	19240502	1	20120209	4
38627508	20120226	16182775	19320512	1	20120226	5
38628987	20110913	15705269	19710412	1	20110913	5
38629071	20110829	15660992	19400211	1	20110829	5
38630363	20110905	15680454	19330812	2	20110905	5
38631300	20110724	15557871	19531110	1	20110724	4
38631504	20111031	15840025	19420224	1	20111031	5
38632405	20110920	15725617	19550123	1	20110920	5
38633293	20110908	15695385	19381022	1	20110908	5
3863351120130926		17949266	19390912	2	20130926	5
38633986	20110927	15744735	19410105	2	20110927	5
38634581	20110921	15730319	19561022	2	20110921	6
38635540	20110820	15640856	19520911	2	20110820	6
38635813	20110823	15647066	19541011	1	20110823	4
38636032	20110904	15678702	19750322	2	20110904	6
38637171	20110907	15692380	19351230	1	20110907	6
38637331	20110828	15659097	19681026	1	20110828	5
38637466	20110819	15639344	19690216	2	20110819	5
38638958	20110903	15678327	19360216	1	20110903	5
38639520	20110823	15646881	19580905	2	20110823	5
38641677	20110830	15664244	19421006	1	20110830	5
38642181	20110912	15702447	19510823	2	20110912	5
38646070	20110903	15678202	19520511	1	20110903	5
38650214	20110915	15713661	19630613	2	20110915	6
38651400	20110928	15747453	19420725	1	20110928	4
38651604	20120208	16133295	19650611	1	20120208	6
38656289	20111029	15837261	19160908	1	20111029	4

38656325	20111031	15839398	19370114	1	20111031	5
38656552	20111108	15869531	19640101	1	20111108	5
38656643	20111107	15865619	19650211	2	20111107	6
38656803	20111113	15883192	19430220	2	20111113	5
38656870	20111114	15886082	19660808	2	20111114	5
38657317	20110925	15738398	19690308	2	20110925	6
38658365	20110826	15655793	19530901	1	20110826	5
38659006	20110831	15666836	19740124	2	20110831	4
38659119	20110906	15687596	20020619	2	20110906	5
38659164	20110908	15695900	19451118	2	20110908	4
38659620	20110921	15728483	19851021	1	20110921	6
38659631	20110927	15742275	19510203	1	20110927	4
38660025	20120314	16240808	19360101	1	20120314	6
38660581	20120425	16361054	19540211	1	20120425	5
38660605	20120403	16296858	19360810	2	20120403	5
38660821	20120418	16343379	19600901	1	20120418	5
38661142	20111228	16013584	19511027	2	20111228	6
38661266	20120101	16022394	19630212	2	20120101	6
38661380	20120110	16055124	19490325	1	20120110	5
38661824	20120119	16083041	19491004	1	20120119	6
38661915	20120129	16094723	19530731	2	20120129	5
38662112	20111004	15764354	19570902	1	20111004	6
38662816	20111014	15797805	19331006	1	20111014	4
38662861	20111030	15838027	19440123	1	20111030	6
38663466	20111129	15926601	19340522	2	20111129	6
38663502	20111201	15936015	19750720	1	20111201	4
38663682	20111227	16011568	19420828	2	20111227	4
38663977	20120308	16222207	20080917	1	20120308	6
3866477620130112		17159173	19750221	2	20130112	5
38665702	20110906	15687725	19500101	1	20110906	5
38665951	20120426	16364109	20100108	1	20120426	6
38669011	20110915	15713506	19320730	1	20110915	4
38673119	20121207	17049832	19560511	1	20121207	6
38673120	20110915	15713938	19960524	1	20110915	4
38673539	20120211	16142862	19460309	1	20120211	6
38673891	20111127	15921670	19610210	2	20111127	4
3867411220130521		17547079	19980421	1	20130521	5
38674178	20110928	15745654	19610220	1	20110928	6
38674190	20110926	15741594	19500310	2	20110926	6
38677597	20110913	15705649	19721115	1	20110913	4
38678772	20111106	15862471	19730124	1	20111106	4
38679833	20110913	15705940	19730628	2	20110913	6
38679980	20110910	15701633	19371018	1	20110910	5
38683566	20110914	15709139	19650514	1	20110914	6
38683657	20110918	15719421	19570826	2	20110918	4
38683817	20120724	16632945	19290226	1	20120724	6
38684569	20110915	15713951	19511212	2	20110915	6
3868731920130807		17792548	19760717	1	20130807	5
38687819	20111011	15787214	19530202	2	20111011	5
38688356	20120819	16711755	19521128	2	20120819	4
38688516	20120313	16237219	19440630	1	20120313	6
38691779	20111022	15819913	19640616	2	20111022	4
38692669	20111102	15851043	19570224	1	20111102	4
38693060	20111013	15794023	19600429	2	20111013	5
38693399	20111027	15831964	19530220	2	20111027	4

38693822	20111123	15913682	19421114	1	20111123	5
38695033	20110924	15737735	19641115	1	20110924	4
38695737	20111026	15828796	19391216	1	20111026	6
38695748	20111012	15790027	19400526	1	20111012	6
38697299	20110918	15718957	19440129	1	20110918	5
38698612	20120101	16022582	19681204	2	20120101	4
38701596	20110920	15725769	19800409	2	20110920	5
38701610	20110920	15726099	19620917	2	20110920	4
38703274	20111011	15785849	19951212	1	20111011	5
38703718	20110922	15731080	19591012	1	20110922	4
38704357	20110926	15741670	19500914	2	20110926	5
38704595	20111004	15766533	19590804	2	20111004	5
38705963	20111006	15775093	19490409	1	20111006	6
38706513	20111003	15761698	19590720	1	20111003	4
38708815	20120725	16637457	19260608	1	20120725	4
38710417	20110929	15750745	19550309	2	20110929	5
38710666	20111027	15833396	19490926	1	20111027	6
38710962	20111025	15826273	19620815	1	20111025	4
38711738	20111011	15785810	19640101	2	20111011	4
38713132	20110930	15753303	19801004	2	20110930	5
38713518	20111204	15943210	19560416	2	20111204	6
38713687	20111010	15782804	19770502	2	20111010	6
38714113	20111005	15769434	19550623	2	20111005	5
38717247	20111107	15866803	19611110	2	20111107	6
38718295	20111120	15903439	19690120	1	20111120	5
38720411	20111020	15814497	19520504	1	20111020	4
38721823	20111115	15890824	19611125	2	20111115	4
3872199220130829		17859906	19960430	1	20130829	4
38722735	20111030	15837834	19700901	2	20111030	5
38725278	20111021	15818324	19520825	2	20111021	6
38725289	20111024	15823033	19801106	2	20111024	4
38727650	20121002	16842633	19590929	1	20121002	5
38729156	20111109	15874401	19750213	2	20111109	6
38729974	20111120	15903584	19750202	1	20111120	6
38730062	20111011	15784941	19680108	1	20111011	6
38731758	20111018	15807338	19631006	2	20111018	5
38732433	20111023	15820230	20060110	2	20111023	6
38732728	20111017	15803049	19540830	1	20111017	6
38733061	20111005	15771464	19740428	1	20111005	5
38733867	20111201	15935660	19651011	2	20111201	4
3873529420121225		17103977	19520711	1	20121225	4
38749687	20111031	15840156	19560715	1	20111031	6
38750866	20111022	15819950	19401010	1	20111022	5
38751007	20111020	15814729	19391109	2	20111020	4
38751085	20111103	15855545	19540701	1	20111103	5
38751289	20111107	15865475	19520815	1	20111107	5
38752248	20111018	15808445	19430419	2	20111018	4
38752704	20111114	15886915	19691210	2	20111114	6
38754368	20111030	15837911	19380102	1	20111030	5
38756717	20111008	15781987	19571203	2	20111008	6
38757801	20111013	15795099	19610126	2	20111013	4
38759716	20111020	15815369	19211213	1	20111020	4
38760713	20111023	15820656	19640529	2	20111023	4
38761512	20111026	15830466	19140801	1	20111026	5
38761727	20111027	15833295	20091102	1	20111027	6

38762297	20111029	15837464	19620315	1	20111029	5
38763223	20111118	15901211	19211225	1	20111118	5
38765423	20111107	15864307	19540826	2	20111107	5
38765956	20111024	15821501	19620401	1	20111024	5
38767838	20111029	15837329	19610520	1	20111029	5
38768740	20111107	15865627	19520612	1	20111107	5
38769107	20120223	16178028	19491206	1	20120223	5
38769221	20111120	15903307	19471120	2	20111120	4
38770126	20111123	15913630	19610208	2	20111123	6
38770240	20111115	15890375	19520102	2	20111115	5
38770659	20111230	16020407	19390615	1	20111230	4
38771481	20121225	17103217	19580809	2	20121225	6
38771607	20111220	15991465	19280201	1	20111220	4
38771970	20111207	15955533	19750505	1	20111207	5
38772779	20111117	15897893	19820627	2	20111117	4
38774628	20111127	15921826	19490620	2	20111127	6
38775096	20120423	16354419	19331205	2	20120423	6
38775552	20111217	15986656	19690414	2	20111217	5
38775596	20111231	16021802	19680502	2	20111231	4
38776271	20111114	15886651	19610430	1	20111114	4
38776599	20111121	15906191	19570831	2	20111121	6
38778313	20111205	15947954	19441206	2	20111205	4
38780153	20111217	15986606	19501111	2	20111217	4
38780175	20111023	15820332	19481122	2	20111023	6
38782035	20111027	15833151	19660501	2	20111027	4
38783469	20121204	17035349	19510307	1	20121204	5
38783618	20120221	16170938	19420110	1	20120221	5
38783878	20111027	15832771	19490320	1	20111027	6
38784097	20111031	15840420	19560320	2	20111031	4
38787041	20111031	15839252	19750510	1	20111031	6
38789649	20111105	15861838	19641103	2	20111105	6
38791627	20120208	16132992	19430210	1	20120208	4
38794319	20120106	16043971	19321124	1	20120106	4
38794615	20111120	15903511	19711120	1	20111120	5
38800134	20111201	15935204	19480401	1	20111201	6
38801615	20120117	16076767	19540227	2	20120117	4
38801671	20111223	16002041	19530408	2	20111223	5
38801717	20111211	15966576	19631003	1	20111211	6
38802732	20111128	15923308	19410924	1	20111128	6
38803882	20111220	15993389	19570720	2	20111220	4
38806369	20111217	15986705	19250823	1	20111217	6
38808229	20111210	15966005	19330317	1	20111210	4
38808354	20111212	15969912	19401212	2	20111212	5
38809197	20111219	15990223	19550613	2	20111219	6
38809835	20111217	15986362	19410521	2	20111217	6
38810047	20111227	16012002	19731202	2	20111227	6
38810116	20111226	16007806	19340210	1	20111226	4
38811039	20111121	15906435	19471101	1	20111121	5
38811277	20111206	15952276	19621114	1	20111206	5
38813513	20111109	15874067	19491216	2	20111109	6
38814301	20111108	15869623	19771113	2	20111108	4
38814823	20111126	15921236	19710407	1	20111126	4
38818734	20111113	15883407	19790214	2	20111113	5
38819771	20120131	16100923	20001222	1	20120131	5
38821351	20111124	15917007	19671125	2	20111124	4

38821748	20111128	15924699	19660730	1	20111128	5
38823017	20120605	16483813	19590710	1	20120605	6
38825137	20111129	15927535	19650612	2	20111129	5
38825193	20111128	15924030	19680728	2	20111128	5
38825240	20111115	15890934	19630116	1	20111115	4
3882579520130702		17675286	19570106	1	20130702	4
38828432	20120315	16243836	19740722	2	20120315	4
38829877	20120102	16025678	19901220	1	20120102	4
38829979	20111220	15993487	19371118	2	20111220	4
38831402	20111117	15895765	19641115	1	20111117	6
38831526	20111124	15916351	19500728	1	20111124	5
38831719	20111125	15919282	19590430	1	20111125	4
38832472	20111126	15921130	19490929	1	20111126	5
38832790	20111121	15906287	19711114	1	20111121	4
38835904	20120101	16022629	19660731	2	20120101	4
38836305	20111231	16021943	19560420	1	20111231	4
38837206	20120224	16180889	19740202	1	20120224	5
38839531	20120228	16187451	19500810	1	20120228	6
38839917	20120229	16189748	19490910	2	20120229	6
38841371	20120104	16037031	19440301	2	20120104	6
38841939	20120322	16263114	19710425	2	20120322	6
38845099	20111123	15913834	19521026	1	20111123	5
38846070	20111204	15943225	19840920	2	20111204	5
38848576	20120325	16268540	19500713	1	20120325	6
38851397	20111114	15886490	19990701	2	20111114	4
38853315	20111121	15903729	19971217	1	20111121	6
38857555	20111205	15947819	19370113	2	20111205	6
38857975	20111206	15952703	19590817	1	20111206	5
38858252	20111208	15960956	19540126	1	20111208	6
3885838720130807		17794930	19291214	1	20130807	6
38859368	20111215	15981831	19280727	1	20111215	5
38859755	20111215	15981319	19340721	1	20111215	5
3886051420131029		18052004	19540810	2	20131029	4
38861095	20111125	15919027	19430930	1	20111125	4
38861904	20111209	15964216	19490928	1	20111209	5
38862394	20111206	15950492	19720911	1	20111206	5
38862850	20120108	16047413	19740301	2	20120108	4
38863831	20111128	15923307	19500428	1	20111128	4
3886554220130801		17772495	19660702	2	20130801	4
38866636	20111212	15969658	19540912	1	20111212	6
38867037	20111130	15930877	19660724	2	20111130	6
38867333	20120419	16345933	19920719	1	20120419	4
38867695	20111210	15965580	19640525	1	20111210	6
38868063	20120421	16351463	19570408	2	20120421	5
38868972	20111222	16000548	19411108	2	20111222	5
3886923720130619		17637172	19571029	2	20130619	6
38870789	20120107	16045596	19361016	2	20120107	6
38871168	20120101	16022690	19650621	1	20120101	6
38871248	20111221	15996571	19270116	2	20111221	6
38872774	20111226	16008934	19450223	2	20111226	5
38876094	20111207	15956670	19450820	1	20111207	4
38877111	20111211	15966392	19460201	2	20111211	6
38878794	20120513	16417726	19420803	1	20120513	5
38879468	20111214	15978018	19410419	1	20111214	4
38879811	20111217	15986641	19340205	2	20111217	4

38881297	20111211	15966358	19460305	2	20111211	5
38883691	20120130	16097694	19510730	2	20120130	5
38883806	20120418	16342801	19580329	2	20120418	6
38884967	20130911	17907479	19711015	1	20130911	5
38885891	20120101	16022465	19790620	2	20120101	6
38886327	20120409	16315563	19620312	2	20120409	4
38886918	20120103	16031612	19330215	2	20120103	4
38887568	20111230	16019625	19520321	1	20111230	6
38888027	20120216	16158186	19360128	2	20120216	5
38889086	20120128	16093857	19331019	1	20120128	4
38889622	20120924	16817367	19560902	2	20120924	5
38890550	20120217	16162401	19370408	1	20120217	5
38891600	20111229	16018226	19310807	1	20111229	6
38891940	20111229	16017679	19520720	2	20111229	4
38892852	20111229	16017862	19620211	1	20111229	5
38893026	20130320	17355521	19551101	2	20130320	6
38893479	20120722	16626991	19510510	1	20120722	4
38895373	20111215	15980629	19500905	1	20111215	4
38897357	20120508	16402966	19650330	2	20120508	6
38897653	20120610	16499975	19701122	1	20120610	4
38900355	20111211	15966599	19530728	1	20111211	4
38905225	20111222	16000652	19640415	2	20111222	4
38906513	20120113	16066341	19531005	2	20120113	6
38908100	20111225	16005905	19801122	2	20111225	4
38908973	20121005	16854374	19540325	1	20121005	4
38909114	20130909	17897764	19821127	1	20130909	5
38909670	20120101	16022678	19620624	2	20120101	5
38910326	20111229	16017767	19730626	2	20111229	4
38910804	20111226	16009069	19600226	1	20111226	6
38911307	20130314	17339645	19620505	1	20130314	5
38911761	20120118	16078002	19580221	2	20120118	4
38913336	20120117	16076854	19570210	1	20120117	4
38914362	20120428	16368980	19470331	1	20120428	6
38916733	20120129	16094516	19550923	2	20120129	6
38918773	20120213	16147198	19510105	2	20120213	5
38919390	20120201	16106070	19550720	1	20120201	6
38921196	20120103	16029262	19621021	2	20120103	5
38922735	20111229	16017349	19430615	2	20111229	4
38925234	20120103	16029962	19570228	1	20120103	5
38929532	20120117	16076612	19360912	1	20120117	6
38929918	20120131	16101749	19530425	1	20120131	6
38935409	20120102	16025867	19320313	1	20120102	4
38935738	20120107	16046930	19621130	2	20120107	4
38935863	20120211	16142874	19740809	1	20120211	6
38937518	20120102	16026486	19440813	1	20120102	4
38938419	20120102	16027685	19320804	1	20120102	4
38940635	20120105	16041081	19950803	1	20120105	6
38941570	20120109	16051085	19530510	2	20120109	6
38943327	20120111	16058997	19481120	1	20120111	5
38943338	20120403	16296122	19360306	2	20120403	5
38943725	20120116	16071849	19550219	1	20120116	5
38945936	20120201	16106260	19411020	2	20120201	5
38946633	20120116	16073514	19560520	2	20120116	5
38948708	20120709	16586630	19540610	2	20120709	5
38952113	20120307	16219062	19370626	2	20120307	6

38954517	20120312	16231819	19540303	2	20120312	5
38958360	20120207	16129182	19571228	2	20120207	4
38966324	20120408	16310951	19730609	2	20120408	6
38966506	20120203	16115336	19381018	1	20120203	6
38966642	20120201	16106661	19500810	1	20120201	6
38967510	20120207	16129036	19480519	2	20120207	5
38967805	20120205	16119858	19480329	2	20120205	4
38969243	20120429	16369788	19610513	1	20120429	5
38970864	20120327	16275047	19510309	2	20120327	5
38971561	20120208	16132625	19461220	2	20120208	4
38972837	20120205	16119606	19580726	1	20120205	5
38972962	20120429	16369725	19400118	2	20120429	4
38973987	20120208	16132588	19531124	2	20120208	6
38975370	20120214	16151867	19451206	2	20120214	6
38976339	20120904	16757520	19380227	2	20120904	4
38979134	20120611	16504184	19440210	2	20120611	5
38979394	20120320	16256137	19730827	1	20120320	6
38983890	20120227	16185216	19540216	2	20120227	5
38986548	20120416	16336710	19530313	2	20120416	6
38986731	20120229	16189214	19671117	2	20120229	5
38986775	20120213	16147830	19740519	1	20120213	4
38987018	20120216	16158858	19580304	1	20120216	4
38987427	20120214	16151891	19631026	2	20120214	6
38987438	20120226	16182701	19361125	1	20120226	5
38991490	20120211	16141841	19400128	2	20120211	5
38991774	20120212	16143300	19590714	2	20120212	5
38992017	20120210	16140619	19520208	2	20120210	5
38992346	20120617	16520693	19700303	1	20120617	4
38994013	20120221	16171542	19701001	1	20120221	6
38995094	20120214	16151074	19550515	1	20120214	5
38995561	20120217	16160579	19380423	1	20120217	5
38996940	20130930	17957382	19460106	2	20130930	6
38997352	20120227	16185334	19610801	2	20120227	4
38997681	20120215	16154350	19360810	1	20120215	6
38999563	20120321	16260439	19610512	1	20120321	6
39000210	20120304	16203907	19800731	1	20120304	6
39004325	20120227	16184625	19390806	1	20120227	6
39010429	20120131	16101320	19390716	2	20120131	6
39010703	20120131	16100892	19721217	2	20120131	5
39011035	20120201	16107737	19660405	2	20120201	5
39013053	20120208	16132025	19530727	1	20120208	6
39013871	20120513	16417534	19450501	1	20120513	5
39014125	20120211	16142882	19431029	1	20120211	4
39017102	20120228	16187444	19630202	2	20120228	4
39020150	20120226	16182839	19380620	1	20120226	4
39021528	20120223	16176669	20030927	2	20120223	6
39021880	20120823	16723608	19630606	1	20120823	5
39022407	20120603	16474632	19330825	2	20120603	4
39023615	20120801	16655579	19290404	1	20120801	4
39023717	20120309	16226532	19420104	2	20120309	5
39024061	20130813	17814358	19901207	2	20130813	4
39024196	20120229	16190096	19441129	1	20120229	4
39024801	20120302	16197994	19630302	1	20120302	5
39024823	20120515	16425375	19460205	2	20120515	5
39025655	20120305	16209138	19510802	1	20120305	5

39026205	20130626	17655287	19410125	2	20130626	6
39027219	20120323	16266165	19450808	2	20120323	6
39027388	20120428	16369032	19451202	1	20120428	6
39029771	20120301	16193738	19820208	2	20120301	4
39029862	20120301	16194989	19501017	1	20120301	6
39030790	20120318	16250528	19550920	2	20120318	6
39030983	20130720	17737782	19560803	2	20130720	6
39033517	20120318	16250476	19491116	1	20120318	5
39035319	20120307	16218155	19530320	1	20120307	4
39041877	20120306	16214545	19331205	1	20120306	6
39045197	20120214	16150557	19370114	1	20120214	4
39046225	20120216	16155879	19980508	2	20120216	6
39046781	20120217	16162357	19280401	1	20120217	5
39048630	20120222	16174273	19730911	1	20120222	6
39048709	20120401	16285443	19301208	1	20120401	6
39048992	20120224	16178110	19460505	1	20120224	4
39049008	20120224	16180300	19530405	1	20120224	6
39049213	20120225	16182300	19300626	1	20120225	5
39051542	20120302	16201270	19601012	1	20120302	4
39051871	20120318	16250508	19630120	1	20120318	6
39053662	20120309	16226974	19790331	2	20120309	5
39053888	20120324	16267888	19350628	1	20120324	6
39060463	20120322	16263337	19630725	1	20120322	6
39061557	20120423	16355359	19550109	1	20120423	6
39061911	20120328	16278379	19640103	1	20120328	5
39064578	20120904	16757803	19501207	1	20120904	4
39065537	20120615	16518198	19511230	1	20120615	6
39065593	20121203	17031122	19490202	1	20121203	5
39065720	20120717	16613748	19581222	1	20120717	4
39065957	20120309	16225908	19520210	2	20120309	4
39066018	20120318	16250408	19450701	2	20120318	6
39066698	20120319	16254245	19630114	2	20120319	4
39067839	20120324	16267940	19650426	2	20120324	5
39068150	20120318	16250603	19581228	1	20120318	5
39069335	20120406	16307928	19470814	1	20120406	4
39072407	20120321	16259977	19620924	1	20120321	5
39072601	20120323	16265565	19711206	1	20120323	6
39073148	20120530	16462947	19440420	2	20120530	5
39074061	20120327	16274262	19630402	1	20120327	6
39075575	20120405	16300960	19360729	2	20120405	5
39075951	20120401	16285435	19390316	1	20120401	6
39076998	20120405	16303044	19380114	1	20120405	6
39077004	20120409	16315746	19640902	1	20120409	5
39083904	20120423	16355432	19421218	2	20120423	5
39085977	20120327	16274718	19700715	2	20120327	5
39086890	20120320	16255876	19550815	1	20120320	5
39088090	20120326	16271938	19551104	1	20120326	5
39088498	20120516	16428909	19440715	1	20120516	6
39089139	20120404	16297839	19460117	1	20120404	4
39090023	20120314	16240701	19550313	2	20120314	5
39090476	20120315	16245249	19390921	1	20120315	4
39093077	20120324	16268318	19850529	1	20120324	4
39094309	20120328	16278231	19390825	2	20120328	5
39094321	20120328	16278318	19430107	1	20120328	5
39094525	20120328	16278148	20090626	1	20120328	4

39096372	20120403	16296847	19550114	1	20120403	6
39096418	20120415	16333812	19630924	2	20120415	5
39097159	20120428	16367824	19411125	1	20120428	6
39098210	20120410	16320126	19510205	1	20120410	5
39098378	20120410	16319985	20070101	2	20120410	6
3909853820130908		17893842	19840508	1	20130908	4
39099746	20120423	16354819	19520520	1	20120423	6
39100624	20120319	16252210	19471029	2	20120319	6
39101796	20120329	16280411	19501126	1	20120329	6
39102959	20120409	16314364	19580626	2	20120409	6
39105107	20120927	16826516	19730530	2	20120927	6
39106053	20120322	16263500	19490523	2	20120322	4
39106473	20120408	16310939	19450212	2	20120408	6
39107385	20120327	16274958	19611112	2	20120327	6
39109610	20120504	16392544	19390225	1	20120504	4
39110026	20120401	16285302	19660201	1	20120401	5
3911020820130106		17135330	19780829	2	20130106	6
39111132	20120504	16391071	19530220	2	20120504	6
39113592	20120528	16457933	19700801	2	20120528	4
39114620	20120426	16364745	19480411	1	20120426	4
39114722	20120415	16333443	19620827	2	20120415	6
39117561	20120409	16315203	19651013	2	20120409	5
39119783	20120501	16377753	19810723	2	20120501	5
39126755	20120501	16376445	19491113	1	20120501	4
39127612	20120408	16310918	19561201	2	20120408	6
39127930	20120423	16354208	19440730	2	20120423	5
3913013720130730		17762980	19521023	2	20130730	6
39130397	20120725	16635160	19470403	2	20120725	5
39130637	20120420	16349314	19360618	2	20120420	5
39131798	20120411	16323060	19590221	2	20120411	5
39133669	20120413	16331640	19770828	1	20120413	6
39139394	20120422	16351881	19550515	2	20120422	5
39141383	20120504	16389359	19750108	1	20120504	5
39141930	20120701	16557040	19641013	2	20120701	4
39142115	20120411	16323627	19550728	2	20120411	6
39146162	20120614	16515154	19840907	2	20120614	6
39148771	20120420	16348627	19670525	2	20120420	6
39151570	20120424	16358008	19710809	1	20120424	4
39153758	20120527	16454662	19491226	1	20120527	5
39154671	20120526	16454180	19750401	2	20120526	6
39155072	20120416	16336070	19220709	2	20120416	4
39155243	20120422	16351841	19860319	1	20120422	4
39155583	20120509	16407940	19410115	2	20120509	4
39155823	20120508	16403136	19480809	1	20120508	5
39155856	20120508	16404212	19400511	1	20120508	4
39156019	20120614	16515266	19490128	1	20120614	5
39156155	20120614	16514931	19400718	1	20120614	4
39156315	20120810	16688303	19990130	2	20120810	5
39156666	20120702	16559125	19650307	2	20120702	5
39157045	20120513	16417657	19651210	1	20120513	5
39157294	20120524	16448907	19360501	2	20120524	6
39157307	20120607	16493145	19590105	2	20120607	6
39157501	20120530	16462083	19681030	2	20120530	6
3915754520130106		17135268	19560504	2	20130106	4
3915839920131104		18070800	19650108	2	20131104	4

39160719	20120519	16436219	19530303	2	20120519	4
39161370	20121207	17049525	19401125	1	20121207	4
39165849	20120503	16386272	19630911	2	20120503	6
39166375	20120504	16392721	19331211	2	20120504	5
39167936	20120419	16347220	19581103	1	20120419	4
39173063	20120515	16421654	19310629	2	20120515	6
39173552	20120516	16428249	19540927	2	20120516	5
39174806	20120522	16441563	19341125	1	20120522	5
39175503	20120417	16338947	19550522	1	20120417	4
39176697	20120425	16361487	19440926	2	20120425	5
39177190	20120428	16369055	19580420	1	20120428	6
39177850	20120528	16457407	19520919	1	20120528	6
39178626	20120422	16351730	19740812	2	20120422	5
39178659	20120423	16355417	19520515	2	20120423	5
39179209	20120419	16346202	20030416	2	20120419	5
39181276	20120420	16349782	19361129	1	20120420	6
39181516	20120503	16388335	19660321	1	20120503	6
39181594	20120425	16360969	19610707	2	20120425	6
39181890	20120429	16369655	19371118	1	20120429	4
39183158	20120423	16352964	19420401	1	20120423	6
39185676	20121031	16925727	19970904	1	20121031	5
39186000	20120430	16370397	19490210	1	20120430	4
39187514	20120719	16621704	19611205	1	20120719	5
39187627	20120502	16383042	19541205	1	20120502	4
39191612	20120429	16369731	19521001	1	20120429	4
39192499	20120515	16423981	19560825	1	20120515	5
39192615	20120426	16364124	19681018	2	20120426	6
39193925	20120611	16503377	19500730	2	20120611	6
39197278	20120501	16378536	19460123	1	20120501	5
39197518	20120501	16378044	19420806	1	20120501	4
39203957	20120522	16443980	19540808	2	20120522	4
3920486920130326		17373213	19470520	1	20130326	5
39204961	20120605	16484667	19880813	2	20120605	5
39207233	20120529	16461001	19610813	2	20120529	6
3920902420130618		17633281	19730102	2	20130618	4
39210418	20120519	16436520	19700723	2	20120519	6
39214089	20120525	16452925	19610503	1	20120525	4
39214567	20120524	16450146	19860811	1	20120524	5
39215946	20120606	16487776	19680205	2	20120606	6
39216369	20120604	16479772	19430905	1	20120604	4
39216803	20120511	16414128	19520226	1	20120511	4
39216938	20120527	16454814	19590731	1	20120527	5
39217179	20120506	16395146	19311005	2	20120506	6
39217760	20120514	16421588	19480522	2	20120514	5
39217953	20120508	16402525	19571017	1	20120508	5
39218172	20121006	16856752	19990220	1	20121006	6
39219868	20120510	16411742	19420915	1	20120510	4
39221722	20120515	16424430	19881108	2	20120515	6
39222338	20120511	16413636	19571220	2	20120511	4
39223171	20120517	16430179	19730429	2	20120517	4
39227888	20120521	16439647	19991013	1	20120521	6
39228018	20120703	16567293	19450319	2	20120703	4
39228483	20120622	16536120	19520925	1	20120622	6
39230198	20120603	16474517	19700305	2	20120603	6
39231179	20120614	16515442	19591011	1	20120614	4

39232570	20120927	16827201	19481105	2	20120927	5
39232627	20120916	16794385	19570918	1	20120916	4
39236016	20120520	16437031	19400115	1	20120520	4
39236130	20120520	16436929	19581010	2	20120520	6
39236301	20120617	16520697	19680414	2	20120617	5
39238205	20120602	16474224	19520320	2	20120602	6
39245631	20120721	16626367	19401101	2	20120721	5
39249337	20121216	17077256	19660922	2	20121216	6
39250389	20120718	16618311	19570228	1	20120718	5
39253457	20120624	16538083	19521204	1	20120624	6
39254790	20130416	17440402	20040630	2	20130416	4
39255351	20120612	16507885	19640220	2	20120612	4
39256149	20120618	16524109	19600901	1	20120618	6
39257299	20120622	16536969	19690510	1	20120622	5
39257302	20120617	16520782	19310404	2	20120617	6
39258689	20120709	16586724	19431110	2	20120709	6
39259148	20120706	16580093	19520416	1	20120706	5
39262630	20120614	16514548	19680822	1	20120614	5
39266416	20120529	16460474	19551010	1	20120529	6
39266790	20120613	16511731	19820408	2	20120613	6
39268525	20120602	16474059	19681203	1	20120602	4
39269197	20120603	16474489	19730920	2	20120603	5
39276976	20120617	16520814	19640327	2	20120617	6
39278950	20130127	17200074	19370703	2	20130127	6
39279599	20130227	17284704	19410103	1	20130227	5
39279635	20120701	16557117	19680517	1	20120701	5
39284703	20120806	16668959	19500226	1	20120806	4
39284907	20120708	16584435	19360813	1	20120708	6
39285751	20120618	16523292	19821221	2	20120618	4
39285819	20120619	16527769	19450112	2	20120619	6
39286389	20120615	16516624	19590726	2	20120615	6
39290421	20120727	16642667	19391122	2	20120727	6
39291468	20120625	16541440	19501201	2	20120625	6
39292234	20120703	16567234	19570101	1	20120703	5
39293533	20120626	16544898	19601101	1	20120626	6
39293602	20120625	16541674	19360216	1	20120625	5
39294387	20120627	16547659	19560902	2	20120627	6
39296043	20120827	16731909	19680920	2	20120827	6
39299100	20120616	16520323	19560519	1	20120616	6
39299360	20130327	17375260	19911114	2	20130327	6
39300088	20120613	16510648	19581101	2	20120613	6
39301581	20120611	16502763	19590525	2	20120611	4
39301796	20120613	16510540	19761204	2	20120613	6
39303236	20120624	16537740	19450730	1	20120624	5
39305732	20120616	16520340	19580222	2	20120616	4
39306359	20120618	16523867	19751208	2	20120618	6
39307330	20120723	16630325	19471028	1	20120723	4
39307409	20120624	16538044	19500105	1	20120624	4
39308571	20120916	16794447	19461125	2	20120916	4
39308582	20120619	16526499	19500503	1	20120619	5
39309756	20120705	16577283	19511001	1	20120705	4
39309803	20120628	16552139	19760331	2	20120628	5
39309972	20120622	16536929	19490110	1	20120622	6
39310560	20120629	16552205	20020808	1	20120629	6
39312602	20120706	16581159	19580902	1	20120706	5

39313638	20120709	16588560	19700104	1	20120709	6
39315418	20120630	16556133	19500826	1	20120630	5
39315509	20120731	16651167	19500220	1	20120731	5
39315634	20121030	16922902	19360516	1	20121030	4
39317163	20120630	16556323	19450405	1	20120630	6
39318020	20120803	16660712	19970724	2	20120803	4
39318713	20120701	16556733	19381011	1	20120701	4
39322491	20120710	16591763	19740718	2	20120710	5
39322946	20120704	16571873	19361017	1	20120704	5
39323518	20120723	16630653	19730307	1	20120723	4
39325376	20120713	16603572	19540128	2	20120713	5
39326120	20120705	16577305	19560210	2	20120705	4
39326277	20120709	16585860	19790208	1	20120709	4
39326697	20120708	16584306	19680810	2	20120708	6
39328513	20120710	16592660	19600210	1	20120710	4
39329210	20120718	16616985	19400315	1	20120718	6
39329243	20120717	16614375	19261225	1	20120717	5
39330024	20120624	16537992	19430408	1	20120624	4
39331550	20120625	16541527	19570228	1	20120625	6
39334173	20120820	16715530	19560305	1	20120820	6
39335665	20120715	16606984	19660404	1	20120715	6
39336226	20120704	16571345	19541203	1	20120704	6
39336420	20120709	16588302	19520601	1	20120709	6
39337616	20120710	16592498	19671103	1	20120710	6
39339418	20120807	16675417	19460720	1	20120807	6
39339907	20120705	16576338	19320907	2	20120705	4
39340186	20121207	17048113	19401029	2	20121207	5
3934138320130420		17452682	19950712	2	20130420	5
39341532	20120715	16606885	19560226	1	20120715	4
39342568	20120723	16629797	19850816	2	20120723	6
39342682	20120731	16651348	19380622	2	20120731	6
39343856	20120721	16626420	19571230	1	20120721	5
39348259	20120820	16712871	19440327	1	20120820	6
3935040820130408		17410445	19641206	1	20130408	5
39350793	20120731	16651570	19890830	1	20120731	6
39355094	20120821	16717954	19510314	2	20120821	6
39357636	20120805	16664869	19601129	1	20120805	5
39357772	20120719	16621517	19650131	2	20120719	5
39360300	20120724	16634161	19521023	1	20120724	4
39362011	20120722	16626736	19440216	2	20120722	5
39362577	20120807	16674893	19590902	1	20120807	5
39363989	20120817	16709653	19410524	1	20120817	6
39364379	20120724	16633953	19340129	2	20120724	6
39365032	20120712	16600873	19570329	1	20120712	4
39366206	20120719	16621759	19560914	1	20120719	6
39368564	20120724	16633762	19381203	2	20120724	5
39369829	20120729	16645600	19311021	2	20120729	6
39370542	20120731	16651493	19680819	2	20120731	5
39372060	20120806	16666563	19261127	1	20120806	6
39372571	20120810	16687377	19470827	1	20120810	5
39374873	20120816	16705019	19430916	1	20120816	5
39374986	20120816	16706555	19500212	1	20120816	5
3937953820121231		17117192	19551023	1	20121231	5
39379743	20120813	16691927	19900326	1	20120813	6
39379947	20120812	16690379	19681110	1	20120812	4

39380262	20120717	16613715	19510712	2	20120717	6
39383750	20120815	16702251	19280311	1	20120815	5
39385029	20120711	16595436	19541024	2	20120711	6
39385132	20120712	16599575	20031128	2	20120712	5
39385176	20120712	16600488	19500321	1	20120712	6
3938617920131003		17973963	19340519	2	20131003	5
39386215	20120811	16689925	19660610	2	20120811	5
39386522	20121015	16881504	19571031	1	20121015	4
39386691	20120825	16728930	19510107	2	20120825	6
39386737	20120828	16735360	19570205	1	20120828	5
39386748	20120828	16735155	19510106	1	20120828	5
39388391	20120812	16690326	19630225	2	20120812	6
39389827	20120902	16746749	19540603	2	20120902	4
39391601	20120905	16761363	19430215	1	20120905	5
39391792	20120929	16831188	19490610	2	20120929	5
39391805	20120914	16791982	19571126	2	20120914	4
39392057	20120918	16801373	19260503	2	20120918	5
39395078	20120808	16679774	19591127	1	20120808	5
39396297	20120725	16637208	19511209	1	20120725	4
39399525	20120916	16794282	19700210	2	20120916	5
39400992	20120915	16793780	19760405	2	20120915	5
39401086	20120730	16648420	19560410	2	20120730	6
39402090	20120730	16648113	19510311	2	20120730	5
39402238	20120812	16690336	19670831	2	20120812	4
39402818	20120807	16673438	19500424	1	20120807	5
39405168	20120819	16711666	19701012	1	20120819	4
39407200	20120804	16664528	19510829	1	20120804	6
39407379	20120814	16698117	19551127	2	20120814	6
39409911	20120911	16781000	19360120	2	20120911	5
39409966	20120904	16756878	19560210	1	20120904	4
39410792	20120916	16794314	19720925	2	20120916	6
39413268	20120813	16694532	19510102	2	20120813	6
39413393	20120814	16699144	19880912	1	20120814	5
39419619	20120919	16805100	19370519	2	20120919	4
39420661	20120821	16718186	19561224	2	20120821	6
39422725	20120817	16709523	19381230	1	20120817	6
39427775	20121006	16857622	19741119	1	20121006	6
39428085	20120807	16671473	19940212	2	20120807	4
39429000	20120730	16648673	19510619	2	20120730	6
39429986	20120814	16697149	19240918	1	20120814	5
39430110	20120808	16677458	19501012	1	20120808	5
39430176	20120803	16662323	19320311	2	20120803	4
39430198	20120801	16654145	19520112	1	20120801	5
39431613	20120814	16698831	19681016	2	20120814	6
39437677	20120809	16684205	19720626	1	20120809	6
39439377	20120813	16694349	19551127	1	20120813	5
39450312	20120809	16682640	19690808	2	20120809	5
39450618	20120815	16703042	19481021	1	20120815	4
39451280	20121003	16847429	19610915	2	20121003	5
39451939	20120813	16694650	19740729	1	20120813	5
3945216920131005		17981492	19480804	1	20131005	6
39456876	20120816	16705279	19560510	1	20120816	4
3945762020131116		18117616	19590725	1	20131116	4
39465957	20120901	16746272	19590602	2	20120901	6
39468003	20120820	16714838	19751024	1	20120820	6

39468581	20120821	16717511	19900329	1	20120821	4
39473615	20120917	16797542	19620202	2	20120917	5
39476909	20120821	16719158	19780917	1	20120821	5
39477275	20120822	16721537	19240325	1	20120822	6
39477833	20120824	16726968	19591218	2	20120824	6
39477935	20120824	16727325	19410810	2	20120824	5
39480370	20120830	16741990	19311014	2	20120830	6
39480392	20121002	16842530	19830831	2	20121002	4
39480983	20120901	16746496	19300123	1	20120901	4
39481828	20120913	16789025	19610425	1	20120913	6
39481997	20120904	16757823	19530810	1	20120904	6
39485013	20120827	16731055	19580302	1	20120827	5
39486083	20120918	16801051	19540812	2	20120918	6
39486129	20120903	16752509	19350904	2	20120903	6
39487031	20121105	16943028	19271024	1	20121105	6
39487133	20120912	16784168	19570510	2	20120912	6
39488250	20120922	16813757	19560710	1	20120922	4
39488261	20121010	16867885	19571012	1	20121010	5
39490658	20130301	17294109	19281123	2	20130301	5
39492223	20120825	16728556	19570919	2	20120825	6
39492609	20120817	16709346	19440315	1	20120817	5
39493500	20120822	16720766	19670615	2	20120822	6
39496565	20120904	16757335	19620323	2	20120904	5
39501332	20120824	16725370	19680320	2	20120824	5
39501627	20120905	16762174	19341023	1	20120905	5
39503383	20120912	16785466	19411204	2	20120912	5
39510731	20130804	17780924	19581004	1	20130804	5
39514711	20121109	16961075	19680923	2	20121109	6
39515338	20120905	16760681	19481204	2	20120905	6
39517038	20120903	16752559	19620311	2	20120903	6
39517403	20120906	16766212	19700802	2	20120906	5
39517516	20120906	16766060	19791204	1	20120906	6
39517776	20120910	16776024	19580419	2	20120910	6
39522526	20120913	16788795	19570810	2	20120913	4
39523198	20120912	16785368	19520614	1	20120912	5
39523632	20120925	16821389	19550808	1	20120925	5
39525047	20121107	16952954	19630726	1	20121107	6
39525332	20120916	16794443	19780315	1	20120916	5
39526517	20121002	16842474	19290317	2	20121002	4
39526846	20121130	17022047	19521001	2	20121130	6
39527258	20120924	16816469	19500602	1	20120924	5
39530615	20130321	17357951	19380303	2	20130321	4
39531458	20120919	16804566	19770206	2	20120919	6
39532928	20121113	16971738	19470401	1	20121113	5
39533625	20130611	17612253	19270303	1	20130611	5
39534220	20120925	16820401	19431210	1	20120925	6
39534902	20120916	16794325	19750528	1	20120916	5
39535198	20120909	16772889	19610815	2	20120909	6
39536306	20120924	16816700	19650124	2	20120924	6
39536317	20121119	16988347	19770919	1	20121119	6
39541305	20120915	16793353	19970728	1	20120915	4
39542397	20121022	16902132	19440703	1	20121022	4
39545567	20120925	16818187	19570317	1	20120925	5
39546582	20120928	16829091	19581010	1	20120928	6
39547596	20120930	16831489	19440517	1	20120930	6

39550522	20120928	16829109	19420527	1	20120928	4
39552335	20120928	16828153	19390127	2	20120928	6
39556020	20120927	16826647	19780603	2	20120927	6
3955676820131112		18103398	19641210	2	20131112	4
39556973	20121002	16841870	19520411	2	20121002	6
3955713620130112		17159197	19450115	2	20130112	4
3955721620130109		17147907	19250913	2	20130109	6
39559847	20121031	16925667	19590219	1	20121031	6
39560059	20121015	16880772	19640325	2	20121015	6
39563376	20121009	16865999	19570217	2	20121009	6
39564891	20121014	16878161	19310922	1	20121014	5
3956585020131017		18020112	19800323	1	20131017	6
39566502	20121001	16836627	19451025	1	20121001	4
39567141	20121008	16862480	19570125	2	20121008	6
39571352	20120925	16819735	19511225	1	20120925	5
39571432	20121005	16855589	19380601	2	20121005	6
39572242	20121107	16952812	19761226	2	20121107	5
39574179	20121017	16890343	19590621	1	20121017	6
39574873	20121015	16882453	19540317	1	20121015	4
3957523020130102		17123134	19431222	1	20130102	6
39575912	20121022	16902707	19740222	1	20121022	5
39576131	20121011	16870492	20010329	2	20121011	4
39576164	20121111	16963416	19540708	1	20121111	4
39576891	20121024	16908838	19530402	2	20121024	4
39578159	20121015	16881518	19650816	1	20121015	4
39578999	20121103	16938101	19451001	2	20121103	6
39581425	20121012	16875723	19540310	2	20121012	5
39581890	20121014	16878321	19521025	2	20121014	6
39582304	20121015	16882471	19570622	2	20121015	5
39583103	20121018	16893443	19341027	2	20121018	4
39587127	20121011	16869175	19301223	1	20121011	6
39588755	20121022	16901528	19380910	1	20121022	6
39590062	20120927	16827263	19730114	2	20120927	6
3959077720130617		17628623	19490514	1	20130617	4
39592148	20121108	16957069	19601014	1	20121108	4
39592922	20121002	16840983	19640320	2	20121002	6
39593878	20121014	16878169	19560225	1	20121014	6
39595169	20121026	16914248	19500826	1	20121026	4
3959622020121029		16920420	19191112	2	20121029	5
39596413	20121031	16923398	19491015	1	20121031	6
39596537	20121031	16923387	19961013	1	20121031	4
39597278	20121103	16938178	19190517	2	20121103	5
39598099	20121121	16996981	19520912	1	20121121	6
39599898	20121112	16967185	19660716	1	20121112	6
39602227	20121102	16934806	19600601	2	20121102	6
39603924	20121210	17058057	19410508	1	20121210	4
3960858720130103		17127330	19540313	1	20130103	5
3960892920130806		17790830	19430415	2	20130806	5
39610270	20121019	16894235	19490114	2	20121019	6
39612209	20121015	16882317	19510316	1	20121015	6
39617191	20121124	17005706	19620118	1	20121124	5
39618161	20121106	16947028	19531102	1	20121106	4
39619471	20121025	16911228	19481028	1	20121025	4
39622430	20121225	17104141	19901011	2	20121225	5
39622714	20121022	16902940	19510706	1	20121022	4

39624527	20121026	16914500	19410219	1 20121026	5
39627902	20121029	16918867	19520117	2 20121029	6
39629168	20121106	16948477	19610331	2 20121106	6
39629931	20121203	17031323	19590115	2 20121203	6
39629997	20121205	17040512	19300512	2 20121205	5
39630676	20121107	16952621	19660112	2 20121107	5
3963132820130612		17614927	19820818	1 20130612	6
39631919	20121028	16917293	19560728	2 20121028	5
39632047	20121024	16908315	19790808	2 20121024	6
39632661	20121106	16945281	19370621	2 20121106	5
39635193	20121112	16967072	19460708	1 20121112	6
39636425	20121022	16902464	19540901	1 20121022	5
39636970	20121031	16925502	19600605	1 20121031	6
39637133	20121026	16915054	19480613	1 20121026	6
39637268	20121117	16985408	19620110	1 20121117	4
39638476	20121216	17077612	19450228	2 20121216	4
39639208	20121105	16939823	19620818	1 20121105	5
39640181	20121110	16963048	19460606	2 20121110	5
39641286	20121126	17009994	19660620	1 20121126	5
39642176	20121126	17008853	19650407	2 20121126	5
39642198	20121129	17018156	19580607	1 20121129	6
39645391	20121119	16986844	19701214	1 20121119	5
39645517	20121108	16955296	19590905	2 20121108	6
39645539	20121109	16958306	19640921	2 20121109	6
39645620	20121112	16966987	19531025	2 20121112	6
39646167	20121118	16985997	19530904	2 20121118	5
39647080	20121127	17013071	19320323	2 20121127	4
3964755720121129		17019447	19470301	1 20121129	6
39647591	20121202	17024999	19961219	1 20121202	5
3964868520121206		17044676	19730321	1 20121206	6
39648798	20121206	17044715	19751225	2 20121206	5
3964890320130124		17195072	19681222	2 20130124	5
3964892520121231		17116714	19620529	1 20121231	4
39649520	20121205	17039053	19390427	1 20121205	5
3965061820130102		17122239	19511205	1 20130102	4
3965163320130218		17255466	19660206	1 20130218	6
39654563	20121106	16947497	19730520	2 20121106	4
39655157	20121105	16943068	19621215	1 20121105	4
39655248	20121112	16967480	19670629	1 20121112	6
39655657	20121102	16934111	19670410	1 20121102	6
39657095	20121204	17035702	19310828	1 20121204	6
39657233	20121108	16954485	19250508	1 20121108	5
39657266	20121205	17041624	19621021	1 20121205	4
39657891	20121210	17057234	19611207	2 20121210	6
39661568	20121212	17066067	19351016	2 20121212	6
39662674	20121129	17019695	19540101	1 20121129	5
39662947	20121128	17016033	19710224	2 20121128	6
3966295820121127		17012368	20060224	2 20121127	4
39663519	20121124	17006029	19430510	1 20121124	4
3966386020130102		17122675	19611220	2 20130102	4
39668047	20121124	17006007	19531020	1 20121124	4
39670116	20121201	17024770	19391013	2 20121201	6
3967235020121211		17063014	19700514	2 20121211	6
3967259820121213		17071365	19600425	1 20121213	5
39682514	20121104	16938507	19450303	2 20121104	4

39682967	20121109	16960759	19650911	2	20121109	5
39683120	20130108	17143589	19550221	1	20130108	4
39684236	20121119	16989177	19550728	2	20121119	6
39684850	20121111	16963390	19570301	2	20121111	6
39686890	20130130	17208976	19641113	2	20130130	6
39688852	20121112	16967543	19580530	1	20121112	6
39689195	20121113	16971268	19531205	2	20121113	5
39692029	20121225	17103594	19960914	1	20121225	6
39692132	20130102	17121699	19670219	2	20130102	6
39692198	20130103	17126551	19301013	1	20130103	6
39692596	20130115	17167886	19581124	2	20130115	5
39692905	20130406	17406761	19380606	2	20130406	4
39692916	20130503	17493406	19991111	2	20130503	6
39693168	20121122	16999830	20001031	1	20121122	5
39693680	20121217	17079886	19550110	2	20121217	6
39695266	20121219	17088529	19461028	1	20121219	4
39696747	20121125	17006510	19590510	1	20121125	5
39698107	20121125	17006581	19470401	1	20121125	6
39698174	20121202	17025317	19731010	1	20121202	5
39708742	20130512	17519146	19600111	2	20130512	4
39711961	20121214	17073677	19551206	2	20121214	6
39712691	20121125	17006554	19560506	2	20121125	4
39713707	20130401	17387813	19731030	2	20130401	6
39713809	20130425	17464831	19420806	2	20130425	4
39713923	20130425	17466070	19630521	2	20130425	4
39715452	20130128	17202116	19330201	1	20130128	4
39715509	20121119	16988137	19470922	1	20121119	6
39716228	20121210	17056514	19630906	2	20121210	6
39716386	20121203	17031215	19671010	2	20121203	6
39717254	20121212	17066800	19820824	1	20121212	4
39722786	20130109	17147767	19860320	1	20130109	6
39723234	20121220	17091845	19350125	2	20121220	5
39725536	20121121	16996573	19540130	2	20121121	5
39725887	20121208	17052696	19730810	2	20121208	4
39727509	20130328	17378547	19470305	2	20130328	4
39727770	20121202	17024985	19560502	1	20121202	5
39728057	20121126	17009556	19481215	1	20121126	6
39729630	20121217	17081476	19460317	2	20121217	5
39732359	20130228	17287785	19451029	1	20130228	4
39732564	20130411	17424332	19680225	1	20130411	5
39732779	20121212	17066115	19601225	1	20121212	5
39732848	20130710	17705388	19660125	1	20130710	6
39732917	20121215	17076969	19800203	1	20121215	5
39733432	20121209	17053234	19430423	2	20121209	5
39733487	20130127	17200038	19610212	1	20130127	5
39735972	20121223	17097344	19490201	2	20121223	6
39737296	20130702	17674545	19480316	2	20130702	6
39738197	20121226	17107577	19581202	2	20121226	6
39738482	20130120	17180417	19680920	2	20130120	6
39738971	20121231	17115733	19590625	1	20121231	4
39749070	20121224	17100824	19540106	1	20121224	5
39765112	20130331	17383868	19990819	1	20130331	6
39765861	20130107	17139145	19550801	2	20130107	6
39767981	20121217	17080625	19750410	2	20121217	6
39768326	20121216	17077316	19800223	2	20121216	6

39768791	20121206	17044673	19660203	2	20121206	5
39768837	20121216	17077631	19710919	2	20121216	5
39770279	20121208	17052788	19381218	1	20121208	5
39770702	20130114	17162458	19601113	1	20130114	6
39772128	20121211	17062970	19340627	1	20121211	6
39772526	20121211	17061715	19471205	1	20121211	5
39774602	20130106	17135078	20050726	2	20130106	5
39774657	20121212	17066559	19840314	2	20121212	4
39790595	20130115	17164663	19511020	1	20130115	4
39792433	20130104	17130798	19410304	2	20130104	4
39794257	20130807	17795285	19440804	1	20130807	5
39795976	20121231	17116826	19530120	1	20121231	6
39796151	20130112	17159022	19400222	1	20130112	5
39796311	20130224	17274062	19740127	2	20130224	6
39796979	20121230	17115117	19490323	2	20121230	4
39797154	20130104	17131751	19621120	1	20130104	4
39797847	20130103	17127141	19590703	2	20130103	5
39798215	20130109	17148454	19520327	2	20130109	5
39798260	20130121	17182311	19460626	2	20130121	6
39799467	20130115	17166796	19410701	2	20130115	5
39799558	20130111	17155446	19640605	2	20130111	6
39799729	20130107	17138328	19470514	1	20130107	4
39802987	20130113	17159738	19420617	1	20130113	5
39803968	20130102	17122037	19660801	1	20130102	4
39807948	20130117	17175030	19411124	1	20130117	6
39816461	20121226	17107496	19620916	1	20121226	6
39817408	20121231	17117177	19781024	1	20121231	6
39818683	20130104	17132426	19350823	1	20130104	5
39820536	20130112	17159001	19580823	1	20130112	5
39820627	20130113	17159500	19761208	1	20130113	6
39820650	20130111	17157215	19320617	1	20130111	5
39820672	20130111	17157371	19240709	1	20130111	6
39822338	20130309	17323772	19430215	1	20130309	4
39823319	20130122	17188384	19250429	2	20130122	6
39823671	20130123	17191467	19840121	1	20130123	5
39824130	20130217	17250707	19631020	1	20130217	6
39840307	20130110	17152782	19471101	1	20130110	6
39841082	20130203	17220293	20010902	1	20130203	5
39842370	20130101	17117593	19571123	1	20130101	5
39846598	20130101	17117569	19400121	1	20130101	5
39846725	20130316	17345003	19410120	1	20130316	4
39847171	20130128	17202480	19740701	1	20130128	6
39847262	20130106	17135251	19431025	1	20130106	4
39848083	20130411	17424062	19350321	2	20130411	6
39848765	20130105	17134814	19380403	2	20130105	6
39865480	20130528	17566648	19650903	2	20130528	4
39865491	20130620	17641020	19540606	2	20130620	5
39865800	20130722	17740336	19391101	2	20130722	5
39865877	20130730	17763230	19530120	2	20130730	5
39866041	20130923	17937186	19500102	2	20130923	6
39867613	20130114	17164058	19841112	1	20130114	4
39870489	20130105	17134571	19320305	1	20130105	4
39872316	20130129	17205450	19460825	2	20130129	5
39872747	20130109	17148659	19440218	2	20130109	6
39873591	20130308	17321043	19340603	2	20130308	4

3987367120121205	17040811	19571025	2	20121205	6
3987559720130121	17184435	19370220	2	20130121	5
3987633020130205	17230746	19730905	1	20130205	5
3987992020130201	17217414	19670715	2	20130201	6
3988148620130125	17197112	19491225	1	20130125	5
3988168020130203	17220586	19860325	2	20130203	6
3988266120130201	17216781	19721212	1	20130201	6
3988281020130130	17209133	19560620	1	20130130	6
3988508020130113	17159706	19820822	2	20130113	4
3988600520130128	17202379	19430810	2	20130128	4
3988691420130109	17149303	19491023	1	20130109	4
3988781520130120	17180509	19411004	2	20130120	5
3988968420130110	17151984	19921025	2	20130110	6
3989126420130115	17166742	19550921	2	20130115	6
3989348620130121	17183081	19741001	1	20130121	5
3989530220130131	17211359	19720515	1	20130131	6
3989586820130126	17199576	19490102	2	20130126	6
3989759120130220	17262829	19340202	2	20130220	6
3990029320130126	17199621	19361015	2	20130126	4
3990085120130129	17205797	19860327	2	20130129	5
3990215320130122	17188352	19241002	2	20130122	5
3990537820130129	17206295	19880611	1	20130129	6
3990788520130208	17241865	19870404	1	20130208	5
3991210220131012	18002565	19401116	1	20131012	5
3991264620130223	17273663	19460626	1	20130223	4
3991325220130702	17675340	19601030	2	20130702	6
3991531620130218	17254605	19541117	2	20130218	6
3991841720130130	17209358	19950322	2	20130130	6
3991995420130129	17205492	19440510	1	20130129	5
3992106720130217	17250595	19391206	2	20130217	6
3992228620130217	17250457	19570926	2	20130217	6
3992279720130319	17352718	19680819	2	20130319	4
3992387020130218	17253958	19530412	1	20130218	5
3992615320130220	17262025	19450829	2	20130220	6
3992647120130214	17245107	19421008	1	20130214	6
3992824020130216	17249116	19630525	2	20130216	6
3992919620130219	17259487	19400629	2	20130219	6
3992957220130526	17560383	19711121	1	20130526	6
3993025120130325	17369023	19580726	2	20130325	5
3993103820130307	17317076	19641201	1	20130307	6
3993148120130611	17611749	19560402	1	20130611	6
3993175420130226	17281720	19470404	1	20130226	5
3993201920130306	17311155	19630729	2	20130306	5
3993284820130324	17365837	19740711	1	20130324	5
3993338520130303	17296801	19650530	2	20130303	6
3993480020130326	17372370	19640329	1	20130326	5
3993558520130205	17229406	19701007	2	20130205	6
3993735420130411	17424409	19910215	1	20130411	5
3993797820130223	17273618	19640116	1	20130223	5
3993815320130223	17273469	19540606	1	20130223	6
3993857320130214	17245897	19591206	2	20130214	5
3993867520130909	17897299	19700723	2	20130909	6
3993952120130203	17220224	19511121	2	20130203	4
3994055120130715	17717294	19321002	2	20130715	6
3994059520130206	17234074	19920410	2	20130206	6

3994107620130321	17360244	19670413	2	20130321	5
3994108720130308	17318243	19620120	1	20130308	5
3994131620130310	17324055	19350320	1	20130310	5
3994132720130307	17317710	19811008	1	20130307	5
3994181620130408	17412136	19460720	1	20130408	4
3994191820130329	17381192	19691113	1	20130329	6
3994196320130401	17388642	19570610	1	20130401	4
3994755220130317	17345491	19601016	1	20130317	5
3994837320130310	17324272	19480911	2	20130310	5
3995225520130304	17302427	19490611	1	20130304	4
3995396320130219	17260025	19620719	2	20130219	4
3996318320130307	17317401	19500923	1	20130307	6
3996554320130522	17550620	19580115	2	20130522	5
3996890620130311	17327938	19650829	2	20130311	6
3996963620130722	17740025	19580820	2	20130722	4
3997397220130319	17352188	19540825	2	20130319	6
3997460220130401	17389710	19591228	1	20130401	5
3997476020130313	17336344	19340113	1	20130313	4
3997529620130228	17288228	19410904	2	20130228	6
3997681320130307	17317191	19341112	1	20130307	5
3997682420130227	17285662	19520416	1	20130227	5
3997718920130325	17369893	19640728	1	20130325	6
3997829520130225	17276810	19321010	2	20130225	5
3997889720130303	17296699	19990104	2	20130303	6
3998052420130227	17285342	19300806	1	20130227	4
3998073920130313	17335059	19861026	1	20130313	4
3998143620130311	17328002	19380918	1	20130311	4
3998162920130306	17311294	19570725	1	20130306	5
3998258620130305	17307433	19720927	2	20130305	6
3998288220130305	17306663	19761021	2	20130305	6
3998330720130305	17307686	19730809	1	20130305	4
3998425320130523	17553522	19481124	1	20130523	6
3998493520130310	17324222	19500625	1	20130310	4
3998889120130317	17345481	19520401	1	20130317	4
3998993020130325	17369201	19601117	1	20130325	6
3999665120130907	17893294	19311022	1	20130907	5
3999739220130313	17336508	19520915	2	20130313	5
3999811320130321	17359310	19540330	2	20130321	5
3999821520130316	17345190	19740930	2	20130316	5
4000084820130417	17443791	19651013	2	20130417	4
4000195420130408	17412039	19590614	1	20130408	4
4000311020130328	17378363	19470315	1	20130328	5
4000398320130620	17641040	19361125	2	20130620	6
4000717820130309	17323842	19410209	1	20130309	5
4000745220130314	17339752	19401001	2	20130314	4
4000946920130315	17342904	19520701	1	20130315	5
4000972120130325	17367289	19431204	2	20130325	5
4001098820130313	17334457	19630711	1	20130313	5
4001166320130312	17332666	19530314	2	20130312	6
4001193620130314	17340198	19880207	2	20130314	6
4001227920130312	17331603	19990523	1	20130312	5
4001247320130325	17368333	19830720	2	20130325	5
4001554120130307	17317260	19660820	2	20130307	6
4001639520130310	17324372	19800829	2	20130310	6
4001685120130313	17335031	19360609	1	20130313	4

4001749020130315	17343257	19421126	1	20130315	6
4001779620130316	17345191	19521108	1	20130316	6
4001835720130318	17349566	19411108	1	20130318	6
4001880220130320	17357151	19580620	2	20130320	6
4001886820130321	17359383	19590302	2	20130321	4
4001985020130903	17877106	19501101	2	20130903	5
4002064220130328	17377700	19260605	1	20130328	6
4002374320130408	17412415	19560218	2	20130408	5
4002394720130409	17415274	19470902	1	20130409	6
4002398120130410	17420015	19711119	2	20130410	4
4002836220130320	17356916	19571115	1	20130320	6
4002862420130323	17365399	19971209	1	20130323	5
4002900320130325	17369394	19560505	1	20130325	6
4002953620130325	17367761	19440915	1	20130325	5
4003020420130327	17376234	19520729	2	20130327	5
4003033920130314	17339500	19531020	2	20130314	4
4003051120130324	17365794	19921024	1	20130324	5
4003056620130316	17345256	19590702	2	20130316	5
4003078220130319	17351950	19581228	1	20130319	4
4003157020130313	17336117	19401027	1	20130313	6
4003164920130314	17340156	19300513	1	20130314	4
4003213120130408	17410638	19500124	2	20130408	4
4003255120130508	17509504	19540301	1	20130508	4
4003429520131008	17992180	19620725	1	20131008	5
4003468220130511	17518629	19830129	2	20130511	4
4003495520130323	17364750	19660626	1	20130323	4
4003654220130326	17373103	19770215	2	20130326	5
4003880020130422	17456800	19701219	2	20130422	6
4003911020130401	17388016	19671103	1	20130401	5
4004653520130619	17635911	19700425	2	20130619	6
4004972720130402	17395422	19680919	2	20130402	4
4004975020130327	17375037	19471201	1	20130327	5
4004986320130327	17376263	19471022	1	20130327	4
4005243520130329	17381208	19560526	1	20130329	5
4005451120130420	17452951	19470816	2	20130420	5
4005567220130502	17486957	19820923	2	20130502	4
4005943620130411	17425662	19620928	1	20130411	5
4005962920130408	17411618	19580110	2	20130408	5
4005985620130407	17407061	19630907	2	20130407	5
4006101620130421	17453258	19370521	1	20130421	5
4006174320130528	17566690	19720301	1	20130528	4
4006231520130405	17404426	19580725	1	20130405	5
4006278020130424	17463809	19401229	1	20130424	4
4006280420130404	17401919	19511122	2	20130404	6
4006314720130409	17416820	19531001	2	20130409	6
4006402620130411	17425343	19530719	2	20130411	6
4006499120130420	17452657	19370226	1	20130420	5
4006662420130519	17540938	19411020	1	20130519	6
4006858420130516	17535643	20101029	1	20130516	4
4006907620130416	17440355	19590612	1	20130416	6
4006925820130429	17473491	19671117	2	20130429	6
4007007320130418	17447989	19550529	1	20130418	4
4007222820130430	17477188	19810809	2	20130430	6
4007308320130429	17473213	20010131	1	20130429	6
4007321020130510	17515773	19320119	1	20130510	6

4007613920130406	17406806	19561125	1	20130406	4
4007645720130411	17425462	19501211	1	20130411	5
4007700720130407	17407439	19650505	1	20130407	5
4007821520130407	17407180	19421111	2	20130407	5
4008045320130517	17538963	19820503	1	20130517	4
4008504920130413	17431209	19340113	2	20130413	6
4008624620130419	17448027	19501018	2	20130419	6
4008722720130422	17455567	19550102	2	20130422	6
4008735220130504	17495543	19590730	1	20130504	4
4009095720130509	17513565	19370302	1	20130509	4
4013247620130513	17522437	19700605	2	20130513	6
4013275020130603	17583907	19600405	1	20130603	6
4013351520130527	17563342	19730701	1	20130527	5
4013483620130519	17541061	19481120	2	20130519	6
4013575920130505	17496237	19490819	2	20130505	4
4013628520130421	17453488	19311123	2	20130421	5
4013713120130922	17933863	19401220	1	20130922	5
4013765320131124	18137936	19780510	1	20131124	5
4013813420130425	17465489	19680616	2	20130425	6
4013835020130502	17487987	19750627	2	20130502	4
4013856520130526	17560216	19660320	2	20130526	4
4013883820130522	17550786	19391226	2	20130522	6
4014516220130427	17471448	19620525	2	20130427	6
4014697220130423	17459442	19340521	1	20130423	6
4014893420130513	17523782	19580916	1	20130513	4
4014911920130428	17471937	19450302	2	20130428	4
4014958420130430	17478340	19800410	2	20130430	5
4015370620130430	17478278	19631124	1	20130430	5
4015390020130513	17523646	19450322	1	20130513	5
4015636320130506	17501057	19560507	2	20130506	5
4015861020130521	17547948	19390803	1	20130521	6
4015933920130429	17473391	19620320	1	20130429	5
4016854520130805	17784420	19360901	1	20130805	6
4016973120130904	17880772	19640913	1	20130904	6
4017139920130501	17483043	19480304	1	20130501	6
4017195720131217	18216063	19760923	2	20131217	6
4017290520130523	17553796	19540106	2	20130523	6
4017391920130510	17515652	19480617	1	20130510	5
4017538020130522	17551784	19420407	1	20130522	6
4017551720130614	17621883	19400110	2	20130614	5
4017650920130612	17615091	19591120	1	20130612	5
4017955320130602	17578423	19631102	2	20130602	5
4017975720130526	17560168	19480520	2	20130526	5
4018003820130618	17632235	19620906	2	20130618	6
4018270520130520	17544829	19381010	1	20130520	5
4018292120130522	17550606	19530102	2	20130522	6
4018731320130519	17541011	19641023	2	20130519	5
4018766420130507	17504779	19670426	1	20130507	6
4019162620130527	17563930	19490526	1	20130527	6
4019165920130527	17562668	19570220	2	20130527	5
4019183120130528	17567347	19571229	2	20130528	6
4019335920130609	17605491	19720507	2	20130609	4
4019365520130617	17628594	19481220	2	20130617	4
4019383720131008	17989872	19291015	2	20131008	6
4019691620130617	17627585	19521220	1	20130617	5

4019731720130617	17629293	19661018	2	20130617	5
4019787320130711	17711360	19430312	2	20130711	6
4019842320130621	17644942	19810716	2	20130621	5
4020046220130507	17504169	19830922	2	20130507	4
4020112520130513	17523467	19650620	1	20130513	4
4020141020130514	17526458	19530911	1	20130514	4
4020153420130513	17521841	19511222	2	20130513	6
4020308520130521	17547435	19990914	1	20130521	4
4020538720130519	17540835	19590807	2	20130519	5
4020556920130516	17535386	19461020	2	20130516	6
4020670020130923	17936823	19660308	1	20130923	4
4020688020130520	17543350	19720205	1	20130520	4
4020909420130519	17541064	19390126	1	20130519	6
4020940320130519	17541033	19340605	1	20130519	5
4021581420130513	17523551	19400426	2	20130513	6
4021772920130613	17617975	19701104	2	20130613	4
4021816420130516	17535147	19930730	2	20130516	5
4021918920130602	17578475	19650421	2	20130602	6
4022088020130519	17541285	19541209	1	20130519	4
4022133820130524	17557568	19480610	1	20130524	6
4022325420130525	17559441	19310605	1	20130525	5
4022362920130527	17562792	19700507	2	20130527	6
4022488220130527	17563697	19420102	1	20130527	6
4022497320130617	17629434	19560205	1	20130617	5
4022614020130607	17603221	19540201	1	20130607	6
4022655920130619	17635663	19611113	1	20130619	6
4022689920130621	17643494	19360830	1	20130621	5
4023029320130618	17631703	19450915	1	20130618	5
4023033920130624	17650274	19400714	2	20130624	6
4023238020130630	17665748	19750824	2	20130630	6
4023401320130611	17614410	19520214	1	20130611	5
4023557220130603	17579543	19611031	1	20130603	6
4023636020130602	17578374	19471115	1	20130602	6
4023737420130529	17568718	19591231	1	20130529	4
4023862820130530	17572463	19600616	1	20130530	5
4024139220130523	17554672	19460430	1	20130523	5
4024154120130524	17557523	19561031	1	20130524	5
4024555420130606	17595680	19361215	1	20130606	4
4024652420130622	17646614	19431015	1	20130622	6
4024711820130721	17738268	19560604	1	20130721	5
4024783420131029	18051775	19361202	1	20131029	6
4024801920131118	18121446	19251215	2	20131118	6
4024802020130922	17933915	19611129	1	20130922	5
4024804220131124	18137986	19531129	2	20131124	6
4024811120130825	17847266	19540131	2	20130825	6
4024820220130827	17854338	19480825	1	20130827	6
4024821320130826	17849460	19540208	1	20130826	5
4024822420130826	17849907	19550623	2	20130826	6
4024854220131009	17996412	19611210	1	20131009	5
4024856420130917	17923234	19440408	1	20130917	6
4024869920130909	17896394	19540604	1	20130909	6
4024874620130911	17907395	19550401	2	20130911	5
4024928320131019	18025303	19470111	1	20131019	4
4024930720131031	18056628	19480110	2	20131031	6
4024962520131112	18103919	19920603	2	20131112	6

4024989620131125	18141731	19450519	1	20131125	4
4025172720130530	17571975	19520913	1	20130530	5
4025538720130620	17640688	19300829	2	20130620	4
4025640420130614	17619781	19450614	1	20130614	6
4026254220130708	17696683	19640111	2	20130708	4
4026355620130607	17601929	19581107	2	20130607	4
4026678220130626	17657873	19441221	1	20130626	4
4026725220130626	17656582	19521108	2	20130626	4
4026938320130621	17644551	19570210	1	20130621	6
4027363020130701	17671686	19901205	1	20130701	4
4027364120130627	17660987	19560226	1	20130627	5
4027679720130725	17751146	19710125	2	20130725	5
4027825920130726	17754901	19531010	2	20130726	6
4028160520130714	17715881	19750103	2	20130714	6
4028294820130629	17665406	19580228	2	20130629	5
4028308720130619	17636511	19341215	2	20130619	5
4028420620130703	17679762	19421003	1	20130703	4
4028435320130619	17636598	19171111	2	20130619	4
4028499920130628	17663753	19541231	2	20130628	6
4028632620130702	17674362	19410913	1	20130702	5
4029027520130730	17763053	19641120	2	20130730	4
4029052620130710	17706823	19670707	1	20130710	5
4029082220130726	17753675	19520801	1	20130726	5
4029090220130815	17823449	19690125	1	20130815	4
4029094620130821	17838405	19530321	2	20130821	4
4029163220130709	17702610	19461205	2	20130709	4
4029240820130716	17723414	19470703	2	20130716	5
4029521420130628	17663876	19340725	1	20130628	5
4029522520130629	17665383	19450519	2	20130629	5
4029560120131031	18057378	19341118	1	20131031	4
4029652420130704	17685183	19590109	2	20130704	6
4029802020130709	17701553	19310723	2	20130709	6
4029909020130712	17714150	19300110	2	20130712	5
4029920520130712	17714921	20011013	2	20130712	5
4030274720130726	17755655	19370209	1	20130726	6
4030424320130801	17771971	19420114	1	20130801	5
4030475420130803	17780498	19500828	1	20130803	5
4030507520130715	17719997	19480901	1	20130715	5
4030511120130630	17665713	19430129	2	20130630	5
4030754820130624	17648968	19500205	1	20130624	5
4030782220130722	17742026	19370706	2	20130722	5
4030794620130626	17656864	19601204	1	20130626	5
4030903320130624	17649762	19630312	2	20130624	6
4031251420130626	17657844	19830120	1	20130626	4
4031253620130630	17665642	19450701	2	20130630	4
4031417820130629	17664762	19580708	1	20130629	5
4031425820130715	17719041	19801129	2	20130715	6
4038033420130721	17738309	19800426	2	20130721	6
4038111120130722	17741319	19451127	2	20130722	6
4038147320130811	17806874	19500918	2	20130811	4
4038279420130816	17826888	19610320	1	20130816	5
4038569120130916	17920633	19571020	2	20130916	6
4038814520130806	17791426	19660207	1	20130806	5
4039225420130710	17707228	19640118	2	20130710	4
4039428320130728	17757703	19351023	2	20130728	5

4039672320130813	17814635	19580505	2	20130813	4
4039689220130805	17786285	19510416	1	20130805	5
4039823020130812	17811248	19751025	1	20130812	4
4040092820130707	17693464	19290514	2	20130707	4
4040528520130722	17740826	19570420	2	20130722	6
4040638020130711	17710577	19571205	2	20130711	5
4040771420130725	17752673	19580409	1	20130725	5
4040999220130724	17748160	19760710	2	20130724	4
4041596120130707	17693525	19561111	1	20130707	4
4041698620130721	17738470	19790219	2	20130721	6
4041726320130712	17714555	19540914	2	20130712	5
4041737620130715	17717513	19431128	1	20130715	5
4042245520130721	17738079	19620105	1	20130721	5
4042349220130721	17738326	19541011	2	20130721	6
4042390320130722	17741758	19490115	1	20130722	5
4042599820130804	17781063	19380505	2	20130804	4
4042612820130805	17785846	19520221	2	20130805	5
4042669520130731	17766400	19380109	2	20130731	5
4042841920130819	17833268	19930917	2	20130819	5
4042957020130728	17757651	19411218	1	20130728	4
4043017920130727	17756703	19640810	1	20130727	6
4043052220130729	17758913	19490214	1	20130729	6
4043111620130808	17799644	19780222	2	20130808	5
4043297120130729	17760366	19610111	2	20130729	6
4043320320130728	17757796	19381026	2	20130728	6
4043628020130811	17806715	19600128	2	20130811	5
4043689320131004	17979555	19600907	2	20131004	5
4043784120130809	17801646	19760913	1	20130809	6
4044168720130804	17780908	19770225	2	20130804	6
4044373020130805	17781329	19431113	1	20130805	5
4044571420130818	17829273	19591110	1	20130818	5
4045081520130725	17752586	19450220	1	20130725	5
4045142120130729	17759857	19670830	1	20130729	6
4045263920130729	17759667	19490110	1	20130729	5
4045288820130813	17815137	19521101	2	20130813	4
4045660820130902	17871596	19520104	1	20130902	5
4045683520130924	17941239	19260818	1	20130924	4
4045737220130904	17880449	19500419	2	20130904	5
4045898820130920	17932044	19550824	1	20130920	5
4046018220130805	17785995	19490223	1	20130805	6
4046041120130803	17780357	19401028	2	20130803	4
4046099920130806	17789398	19551002	2	20130806	6
4046182320130912	17910763	19731222	2	20130912	5
4046280420130805	17781859	19520324	2	20130805	5
4046349820130802	17775732	19530125	2	20130802	5
4046353420130813	17814644	19680513	2	20130813	5
4046476420130813	17815398	19641128	1	20130813	5
4046486620131023	18036024	19640203	2	20131023	6
4046758120130807	17794780	19350809	2	20130807	5
4046795620130811	17806494	19671010	1	20130811	4
4046859520130904	17879013	19410808	1	20130904	5
4046903220131130	18156051	20070312	2	20131130	5
4047016420130908	17894042	19830309	1	20130908	5
4047073320130810	17806219	19400326	1	20130810	6
4047141820130828	17858077	19541105	2	20130828	5

4047851120131009	17994686	19681205	2	20131009	5
4048159220130929	17954672	19621022	2	20130929	6
4048274420130904	17877710	19690415	1	20130904	6
4048298220130909	17894733	19560417	1	20130909	5
4048352120130915	17917448	19370905	2	20130915	4
4048564120130822	17840809	19420615	1	20130822	5
4048591420130816	17826678	19511230	1	20130816	5
4048698420131114	18111113	19750313	2	20131114	6
4048717020130916	17920512	19550625	1	20130916	5
4048791020130923	17937017	19530212	1	20130923	4
4048998320130923	17936391	19620910	1	20130923	6
4049019520130826	17850472	19510311	1	20130826	5
4049104120130817	17828732	19510108	1	20130817	5
4049157420130819	17832451	19470205	2	20130819	4
4049371820130903	17873580	19580221	1	20130903	6
4049388720130829	17859449	19501013	1	20130829	5
4049670620130815	17820143	19620917	2	20130815	6
4049690020130818	17829064	19570313	2	20130818	6
4049729820131008	17991684	19630919	2	20131008	5
4049816620131018	18022718	19511005	2	20131018	5
4049996520130901	17865155	19651109	2	20130901	6
4050115320130901	17865081	19671020	1	20130901	4
4050181320130822	17840667	19691010	2	20130822	6
4050269020130826	17850996	19360816	1	20130826	5
4050339720130830	17861437	19360214	1	20130830	4
4050369320130831	17864746	19750815	1	20130831	4
4050713920130820	17836388	19701107	1	20130820	6
4050865420131222	18228247	19440308	1	20131222	5
4050940820130825	17847478	19530818	1	20130825	4
4051084720130827	17851997	19891118	2	20130827	6
4051315320130928	17954125	19330722	2	20130928	6
4051430520130908	17893881	19901004	1	20130908	6
4051564820130829	17859863	19710522	2	20130829	6
4051664120130903	17877273	19561110	2	20130903	4
4052016920130731	17766575	19351116	2	20130731	5
4052061420130802	17777566	19580103	1	20130802	6
4052310220131030	18052698	19740831	2	20131030	6
4052507320130906	17890554	19450815	1	20130906	5
4052644120130909	17897779	19450413	1	20130909	5
4052854920131006	17982121	19590501	1	20131006	6
4053106420130918	17928936	19250101	1	20130918	4
4053203420130924	17939502	19530115	1	20130924	6
4053259020130929	17954646	19570902	1	20130929	6
4053334420131001	17963402	19601119	1	20131001	5
4053518020130916	17919308	19321025	2	20130916	6
4053532820130922	17934084	19591007	2	20130922	4
4053557720130927	17952671	19661023	1	20130927	4
4053626320130923	17937559	19670811	2	20130923	6
4053737920130927	17952748	19770225	1	20130927	6
4053902420130907	17893362	19381130	1	20130907	6
4053931920130917	17925372	19541020	1	20130917	5
4054466920131121	18133115	19661010	1	20131121	4
4060566120131005	17981556	19540305	1	20131005	4
4060714520130913	17912727	19560401	1	20130913	6
4060742920130913	17914958	19660308	2	20130913	4

4060894420131103	18066511	19620724	1	20131103	5
4060967420131007	17986923	19460915	2	20131007	5
4061062620131001	17962807	19660118	2	20131001	6
4061183420131003	17974609	19500622	2	20131003	6
4061215520131014	18006914	19370925	2	20131014	5
4061328320131008	17992197	19631129	2	20131008	4
4061329420131103	18066693	19550402	1	20131103	6
4061420820131016	18013552	19291009	1	20131016	6
4061422020131109	18094351	19671114	2	20131109	6
4061423120131014	18006593	19591125	1	20131014	6
4061428620131014	18007327	19420901	2	20131014	6
4061475320131008	17989789	19831231	1	20131008	5
4062734720131012	18002808	19270122	2	20131012	5
4062835120131006	17982172	19620518	2	20131006	6
4062954720131002	17969005	19570525	2	20131002	5
4063374720131022	18030490	19411206	1	20131022	4
4063459120130930	17958121	19560521	2	20130930	4
4063576520130910	17902838	19421001	2	20130910	4
4063726120130927	17952662	19501006	2	20130927	4
4063836620131002	17969752	19490920	2	20131002	6
4063902920131004	17979796	19721105	1	20131004	4
4064238420131007	17984512	19791009	2	20131007	5
4064408420131022	18031419	19810628	2	20131022	6
4064552120131102	18065559	19501112	1	20131102	4
4064666020131015	18012095	19441120	2	20131015	5
4064674020131016	18014402	19580126	1	20131016	5
4064703820131004	17978669	19390210	2	20131004	5
4064931820131015	18010403	19390828	1	20131015	6
4065056420131022	18033006	19590530	1	20131022	6
4065386920131106	18081066	19550308	1	20131106	6
4065390520131103	18066488	19580125	1	20131103	5
4065425920131019	18025268	19470115	2	20131019	4
4065874020131020	18025581	19501203	2	20131020	5
4065876220131105	18078324	19440909	1	20131105	5
4065911820131104	18070410	19570808	1	20131104	5
4066038620131031	18058777	19510301	1	20131031	6
4066350120131031	18057670	19470101	1	20131031	5
4066449120131122	18134554	19701111	1	20131122	6
4066842620131028	18048756	19490216	1	20131028	4
4066870020131203	18168187	19400522	1	20131203	6
4066930520131002	17968453	19630403	2	20131002	6
4067089120131020	18025987	19620815	1	20131020	5
4067133820131028	18049212	19720526	1	20131028	5
4067218220131108	18091964	19510825	1	20131108	4
4067327620131016	18014081	19310626	1	20131016	6
4067502320131104	18071923	19451015	1	20131104	5
4067784720131022	18031970	19450715	1	20131022	6
4067813520131028	18049360	19460915	1	20131028	5
4067874820131117	18118245	19541220	2	20131117	6
4068038420131021	18027299	19530108	2	20131021	6
4068325820131123	18137449	19510918	2	20131123	6
4068376920131201	18156501	19360217	1	20131201	6
4068709020131109	18094274	19380824	2	20131109	6
4068826420131119	18125659	19610826	2	20131119	5
4068892420131210	18192705	19520215	2	20131210	4

4069065120131027	18046062	19620527	1	20131027	4
4069223720131126	18143903	19410408	2	20131126	4
4069281720131108	18090366	19440928	1	20131108	6
4069404020131112	18104481	19501120	1	20131112	4
4069618220130914	17916821	19590427	1	20130914	5
4070428720131105	18075124	19430102	1	20131105	5
4070494720131119	18124956	19391209	2	20131119	6
4070515520131113	18107025	19710825	1	20131113	4
4070581520131103	18066685	19500201	2	20131103	6
4070588220131025	18041551	19291230	1	20131025	4
4070772020131230	18247781	19610107	2	20131230	5
4070926020131209	18189477	19560126	1	20131209	5
4071433820131126	18143864	19750224	2	20131126	4
4071474720131119	18125478	19571001	1	20131119	5
4071490720131127	18147933	19540304	2	20131127	5
4071789320131215	18208789	19490507	1	20131215	5
4072032920131125	18139989	19491123	2	20131125	6
4072053420131126	18143305	19550920	1	20131126	5
4072083020131211	18198926	19400320	1	20131211	5
4072150420131211	18198032	19731205	2	20131211	6
4072526620131125	18140741	19671010	2	20131125	6
4072617820131203	18166204	19580502	2	20131203	5
4072780820131203	18166399	19481128	2	20131203	4
4072805020131125	18141578	19520801	2	20131125	6
4072837820131121	18132395	19730702	2	20131121	5
4073067420131105	18076007	19830104	2	20131105	4
4073087820131109	18094466	19611020	2	20131109	6
4073127920131124	18137872	19330201	2	20131124	6
4073267020131106	18083743	19820226	2	20131106	6
4073343520131126	18143780	19530301	2	20131126	4
4073385520131118	18120463	19690308	1	20131118	5
4073458520131126	18144567	19680213	2	20131126	6
4073549720131119	18125409	19680130	2	20131119	4
4073660520131123	18137283	19580220	1	20131123	4
4073775520131127	18148587	19561116	2	20131127	4
4074122820131222	18228324	19550227	2	20131222	6
4074270920131209	18189081	19511020	2	20131209	4
4074512820131009	17994662	19671108	1	20131009	6
4074514020131009	17996634	19401226	1	20131009	6
4074566220131011	18000354	19401102	2	20131011	6
4074669820131015	18012206	19630802	2	20131015	4
4074739520131018	18023375	19611208	1	20131018	4
4074743120131018	18023574	19370417	2	20131018	5
4074855820131023	18035952	19690428	1	20131023	4
4075118620131215	18208487	19590320	1	20131215	6
4075217820131222	18228154	19490921	1	20131222	4
4075235020131217	18216261	19570129	1	20131217	6
4075350220131216	18211062	19201027	1	20131216	6
4075698720131126	18143842	19571019	1	20131126	5
4075794620131208	18185321	19520530	1	20131208	6
4076049420131206	18181014	19450818	1	20131206	5
4076059620131118	18120967	19830211	2	20131118	6
4076110220131121	18132673	19620722	1	20131121	6
4076424720131225	18235455	19370627	1	20131225	6
4076545520131127	18148148	19460811	1	20131127	5

4076563720131202	18161976	19700811	1	20131202	6
4076623220131128	18151420	19701013	2	20131128	4
4076813620131209	18188550	19431122	2	20131209	5
4076822720131213	18205295	19530920	2	20131213	6
4076829420131220	18225291	19460602	2	20131220	5
4076857820131222	18228163	19470818	2	20131222	6
4077005620131118	18122105	19630202	1	20131118	5
4077228920131126	18142961	19401226	2	20131126	5
4077264320131204	18170495	19630709	1	20131204	4
4077290520131201	18156737	19751001	2	20131201	4
4077324820131208	18185250	19630326	1	20131208	4
4077482120131208	18185090	19590103	2	20131208	6
4077499020131203	18166554	20060601	1	20131203	4
4077593720131208	18185200	19591205	2	20131208	6
4077746620131202	18162014	19820210	1	20131202	6
4077770620131208	18185225	19580606	2	20131208	4
4077866320131209	18190077	19710902	1	20131209	5
4079008720131205	18177513	19861007	2	20131205	6
4079434120131219	18222989	19421214	2	20131219	5
4079595720131216	18211400	19790627	1	20131216	5
4079851420131218	18218394	19490202	1	20131218	6
4079953920131219	18221108	19650306	2	20131219	5
4081687320131224	18234090	19620217	2	20131224	6
4085667520131216	18210578	19270820	2	20131216	5
4086058020131030	18054450	19560317	2	20131030	6
4086063720131030	18054951	19801224	1	20131030	5
4086345420131111	18099443	19491206	1	20131111	5
4086347620131111	18099957	19410303	1	20131111	6
4086352320131116	18117493	19481021	1	20131116	6
4089645520131125	18141627	19640320	1	20131125	6
4089648820131128	18151021	19601210	2	20131128	5
4089681920131127	18148281	19480317	1	20131127	5
4089705020131129	18154265	19410728	1	20131129	6
4089927220131209	18189509	19390912	1	20131209	5
4094746420131025	18043832	19490328	2	20131025	4
4101031520131214	18208262	19490213	2	20131214	4
50259 20110409	15249353	19280125	1	20110409	6
5910920131208	18185102	19320715	1	20131208	6
108703 20120902	16746709	19291003	1	20120902	6
111024 20120503	16388588	19260212	1	20120503	4
177026 20120627	16547323	19330420	1	20120627	6
178563 20120918	16802127	19451103	2	20120918	4
18290120130329	17381478	19280815	1	20130329	5
204308 20120804	16664547	19330721	2	20120804	5
23213320121222	17097039	19211218	1	20121222	4
275547 20111112	15882758	19230906	1	20111112	5
297938 20110126	15043744	19320127	1	20110126	4
29865720130410	17421229	19331125	1	20130410	6
39844720130902	17871066	19260905	1	20130902	6
41153020121106	16946638	19230120	1	20121106	6
428284 20121125	17006627	19241025	1	20121125	4
434106 20121107	16952766	19321111	1	20121107	6
47718920130712	17713178	19320529	1	20130712	5
482393 20111124	15916421	19400527	1	20111124	6
485938 20120319	16254064	19320601	1	20120319	6

486215	20130310	17324398	19300924	1	20130310	6
490459	20130520	17544619	19300121	1	20130520	4
536221	20110930	15753018	19260201	1	20110930	4
586867	20121031	16926107	19221027	1	20121031	4
631436	20130426	17468834	19310319	1	20130426	5
738738	20110501	15309136	19310303	1	20110501	6
763086	20111009	15782353	19390302	2	20111009	5
781782	20131016	18015502	19450415	2	20131016	5
845921	20131215	18208673	19450225	2	20131215	5
847256	20130617	17629298	19420817	1	20130617	6
868462	20130619	17635954	19181014	1	20130619	5
920898	20110814	15622329	19320920	1	20110814	4
962129	20121022	16902261	19260421	1	20121022	6
973568	20120321	16260814	19630604	1	20120321	6
1040693	20110903	15678316	19530818	1	20110903	6
1060408	20120701	16556588	19210804	2	20120701	4
1223003	20120628	16550539	19281017	2	20120628	4
1242882	20130120	17180671	19421015	2	20130120	4
1272613	20121201	17024664	19290205	1	20121201	4
1290206	20131013	18003033	19200508	1	20131013	6
1408828	20130219	17256356	19250224	1	20130219	6
1597213	20120917	16798302	19410717	1	20120917	5
1635832	20110114	15011298	19210203	1	20110114	5
1684613	20120625	16541860	19260920	2	20120625	6
1687327	20130312	17331737	19290318	2	20130312	6
1689538	20111208	15958837	19221226	1	20111208	4
1693965	20120104	16037289	19331128	1	20120104	5
1750534	20130716	17723968	19201012	1	20130716	5
1777237	20130830	17862313	19240504	2	20130830	5
1822351	20130626	17656250	19330919	1	20130626	6
1904796	20131211	18196889	19300103	1	20131211	5
1916376	20110929	15750781	19201002	1	20110929	6
1929733	20111207	15956883	19420902	2	20111207	6
1948589	20131023	18037058	19260420	1	20131023	4
1981437	20130116	17168231	19280525	1	20130116	6
1984301	20111102	15850376	19280711	1	20111102	5
1984925	20120307	16217878	19190712	1	20120307	5
2075152	20130711	17711453	19350325	2	20130711	5
2090757	20111002	15756710	19320920	1	20111002	6
2106936	20110927	15744866	19400816	1	20110927	5
2131206	20130305	17306075	19411009	2	20130305	4
2153915	20130427	17470930	19471106	2	20130427	5
2171553	20120518	16434705	19321208	1	20120518	6
2181171	20120202	16112009	19450509	2	20120202	6
2207467	20110517	15362743	19190906	1	20110517	4
2212626	20120522	16442460	19321027	1	20120522	5
2251947	20110615	15445476	19491107	2	20110615	5
2364365	20120103	16032782	19300614	2	20120103	6
2371622	20130429	17475159	19260726	1	20130429	5
2394050	20110503	15318141	19490702	2	20110503	6
2414359	20130708	17695385	19540321	1	20130708	5
2488699	20131112	18102907	19260208	1	20131112	5
2511344	20110628	15479100	19320310	1	20110628	6
2518992	20131110	18094937	19421129	2	20131110	4
2546330	20121107	16949850	19400325	1	20121107	4

2557973	20130310	17324118	19280919	2	20130310	4
2583713	20120909	16772882	19271016	2	20120909	6
2617007	20110601	15402392	19180607	1	20110601	4
2624353	20120502	16384073	19300222	2	20120502	5
2655381	20130129	17204996	19280905	1	20130129	6
2739051	20120412	16326698	19670326	2	20120412	6
2955819	20120613	16510097	19701017	1	20120613	6
2968583	20121220	17090674	19351022	2	20121220	6
3043934	20121111	16963415	19270219	1	20121111	5
3065063	20111205	15946224	19250215	1	20111205	5
3093978	20120811	16689969	19430517	1	20120811	6
3127773	20120207	16128128	19310429	2	20120207	6
3232006	20121008	16862831	19381228	2	20121008	6
3273210	20131008	17991321	19371229	2	20131008	6
3314792	20111012	15789911	19200310	2	20111012	6
3316469	20120324	16268125	19520827	2	20120324	4
3325824	20110220	15106300	19190813	1	20110220	5
3370603	20110601	15402171	19280706	1	20110601	6
3406517	20111112	15882873	19300820	1	20111112	5
3460160	20130709	17698995	19541029	2	20130709	4
3562230	20110510	15341993	19300108	1	20110510	5
3589648	20120521	16439850	19461010	2	20120521	6
3617230	20121020	16898432	19170529	1	20121020	4
3683747	20120324	16268133	19590923	2	20120324	5
3696911	20111229	16018283	19370220	1	20111229	5
3792583	20120530	16462539	19291026	2	20120530	4
3839187	20130705	17690272	19240805	2	20130705	4
3959088	20130325	17369805	19500103	2	20130325	4
4050479	20111018	15807269	19511203	2	20111018	5
4091025	20120630	16555943	19190619	1	20120630	5
4128136	20120325	16268515	19551001	2	20120325	4
4149273	20120905	16760555	19440517	2	20120905	5
4214340	20120527	16454608	19180322	1	20120527	6
4305388	20120924	16816689	19210302	2	20120924	5
4307737	20120228	16187248	19401012	1	20120228	5
4309277	20130715	17719239	19240719	1	20130715	4
4457425	20130512	17519319	19290118	1	20130512	6
4529402	20110323	15202233	19220908	1	20110323	4
4575886	20131129	18154896	19510810	1	20131129	4
4611932	20131003	17975257	19461003	1	20131003	5
4648059	20110421	15286483	19360209	1	20110421	5
4661067	20121214	17072880	19400327	1	20121214	6
4702027	20120601	16470427	19350710	1	20120601	6
4780158	20131203	18166274	19590201	1	20131203	6
4785948	20120309	16225511	19280102	2	20120309	5
4816448	20131002	17969910	19660509	1	20131002	5
4822575	20110405	15233912	19540801	1	20110405	5
4826260	20110806	15600231	19251217	1	20110806	4
4868999	20120603	16474473	19480514	2	20120603	6
4891172	20130907	17893293	19460403	1	20130907	4
4899176	20120608	16495746	19490301	2	20120608	4
4900087	20110926	15741849	19480320	1	20110926	4
4972194	20110913	15706076	19320427	1	20110913	4
5065441	20110613	15439058	19550103	2	20110613	6
5110167	20111209	15964403	19230901	2	20111209	5

5272559	20121025	16912534	19260505	1	20121025	6
5293538	20121222	17096678	19290128	2	20121222	4
5363337	20111111	15880795	19530627	1	20111111	5
5487285	20120729	16645385	19561126	2	20120729	4
5497916	20120103	16031997	19501013	2	20120103	4
5515208	20130720	17737756	19580307	1	20130720	4
5542381	20121018	16893510	19260506	2	20121018	4
5557460	20130222	17271876	19440709	2	20130222	4
5631754	20130114	17162456	19571015	2	20130114	6
5705180	20110418	15275824	19370220	2	20110418	6
5707415	20110523	15379161	19421218	1	20110523	4
5872208	20130528	17566916	19281110	1	20130528	5
5912743	20130905	17885777	19270522	1	20130905	6
6016575	20120520	16436878	19250123	1	20120520	6
6040751	20120412	16324464	19250107	1	20120412	5
6066031	20110824	15651296	19460220	2	20110824	4
6293501	20120111	16058806	19300303	1	20120111	5
6315453	20121001	16837227	19531211	2	20121001	6
6343437	20110925	15738406	19670304	2	20110925	6
6349822	20110924	15737045	19241006	1	20110924	4
6352121	20111227	16011517	19250515	1	20111227	4
6355926	20131031	18058820	19240405	2	20131031	6
6416462	20110206	15060148	19290806	1	20110206	5
6430495	20130609	17605611	19310506	1	20130609	5
6437101	20130514	17527796	19451225	2	20130514	5
6437656	20130317	17345567	19431028	2	20130317	6
6447809	20120528	16458113	19490910	1	20120528	4
6535291	20111219	15990061	19330128	1	20111219	6
6541588	20130417	17442080	19501214	1	20130417	5
6600379	20120108	16047544	19311015	1	20120108	6
6607814	20110525	15384429	19540221	1	20110525	4
6658259	20130820	17836963	19250908	1	20130820	5
6866519	20121210	17054854	19290223	1	20121210	4
6947848	20130316	17345167	19310811	1	20130316	6
6969671	20120624	16537796	19540610	2	20120624	5
6987913	20111120	15903398	19430124	1	20111120	5
7019387	20110304	15147380	19590201	2	20110304	6
7020646	20120308	16223405	19581021	2	20120308	5
7291261	20110912	15702584	19310801	1	20110912	6
7532378	20110819	15639434	19210812	1	20110819	5
7570618	20121206	17043299	19251007	1	20121206	5
7641227	20110131	15053667	19521101	2	20110131	6
7643041	20130122	17188180	19460328	2	20130122	5
7703744	20120512	16417058	19371008	2	20120512	4
7731217	20130910	17902247	19781116	1	20130910	5
7742769	20121002	16842560	19330729	1	20121002	5
7869661	20130628	17663407	19251106	2	20130628	5
7918118	20120330	16283809	19510301	2	20120330	6
7930418	20110607	15419483	19560709	2	20110607	4
7981557	20130128	17201667	19340713	1	20130128	6
8003063	20110530	15392513	19500401	1	20110530	6
8012859	20130925	17945635	19281003	1	20130925	5
8081649	20130513	17523235	19341229	2	20130513	6
8097903	20130623	17647157	19460810	2	20130623	4
8136707	20121212	17066377	19280624	1	20121212	4

8180232	20120806	16670090	19401203	1	20120806	5
8211049	20110325	15208560	19590723	2	20110325	6
8331677	20130206	17232898	19281003	1	20130206	5
8372187	20110825	15654406	19360312	2	20110825	5
8377400	20110921	15729849	19290608	1	20110921	5
8395173	20120719	16621777	19471128	2	20120719	5
8498746	20130930	17957590	19420108	2	20130930	6
8515784	20120610	16500193	19440728	2	20120610	5
8604340	20130626	17657198	19490910	1	20130626	6
8630215	20120412	16326455	19790415	1	20120412	6
8663636	20130312	17332028	19290426	1	20130312	5
8683509	20110801	15580000	19391122	2	20110801	5
8916323	20120330	16282698	19470715	1	20120330	5
8967166	20130813	17814277	19450222	1	20130813	4
9026180	20110411	15253223	19420625	2	20110411	6
9066868	20121107	16953026	19420603	1	20121107	5
9073147	20111017	15804867	19500514	1	20111017	5
9133179	20131101	18062202	19290216	1	20131101	5
9135346	20131220	18225723	19560513	1	20131220	6
9219152	20120605	16484329	19570328	1	20120605	4
9294444	20130715	17720392	19501007	2	20130715	4
9330841	20130402	17395253	19520923	1	20130402	6
9337364	20130617	17628575	19431229	1	20130617	4
9369497	20130213	17244394	19590626	2	20130213	4
9408837	20120313	16236554	19320917	2	20120313	5
9410439	20120404	16298645	19281025	2	20120404	5
9416119	20130830	17862743	19270527	1	20130830	6
9518358	20110129	15050726	19320206	1	20110129	5
9561131	20131205	18178218	19740924	2	20131205	6
9575853	20131230	18248904	19591012	2	20131230	6
9599897	20120708	16584434	19400712	1	20120708	6
9674446	20120402	16289634	19590608	1	20120402	6
9683209	20120505	16394430	19330825	1	20120505	6
9913273	20130528	17566603	19330715	2	20130528	5
9922252	20110503	15319937	19611027	1	20110503	4
9961584	20120609	16499696	19560308	1	20120609	5
9984092	20120401	16285564	19540926	2	20120401	5
10100648	20110914	15708708	19250413	1	20110914	5
10126064	20120607	16493330	19550813	1	20120607	5
10131029	20110420	15283532	19370412	1	20110420	6
10182306	20121109	16957369	19350503	1	20121109	4
10225793	20120423	16355070	19540815	1	20120423	4
10234261	20110503	15319318	19511011	1	20110503	4
10271495	20120408	16311015	19430820	1	20120408	6
10280101	20131107	18086252	19391219	1	20131107	4
10311098	20131126	18145370	19470507	1	20131126	4
10311587	20130406	17406854	19570127	2	20130406	5
10339841	20110207	15060617	19310607	1	20110207	5
10537527	20120131	16100788	19490923	1	20120131	4
10544920	20130118	17178142	19440214	2	20130118	5
10681819	20130125	17195798	19480118	2	20130125	6
10684272	20110510	15340001	19471102	1	20110510	4
10869166	20121116	16983511	19300824	1	20121116	6
10924115	20120618	16523650	19150116	1	20120618	6
10939818	20120307	16218389	19431225	1	20120307	4

11006272	20130808	17798577	19611121	1	20130808	6
11018998	20110515	15356670	19490610	2	20110515	5
11020272	20120928	16829030	19240205	2	20120928	6
11024672	20110620	15459224	19590417	2	20110620	4
11032545	20120414	16331686	19351207	2	20120414	6
11050092	20131015	18008619	19300205	2	20131015	5
11102844	20130119	17179983	19270911	1	20130119	6
11118788	20131022	18033294	19561116	1	20131022	6
11130862	20130502	17487693	19321001	1	20130502	4
11140822	20110613	15438646	19290505	1	20110613	5
11169792	20110908	15696103	19480915	1	20110908	4
11255713	20120731	16650688	19461226	1	20120731	4
11278812	20130311	17328600	19260825	1	20130311	4
11298729	20110401	15226702	19570702	2	20110401	6
11330839	20111126	15921413	19310912	2	20111126	6
11347981	20131114	18112642	19530716	1	20131114	4
11487460	20120807	16671515	19280429	2	20120807	5
11495899	20120205	16119695	19521029	2	20120205	6
11515483	20111208	15960819	19510304	2	20111208	5
11515881	20111210	15965124	19300622	1	20111210	6
11601379	20131127	18149006	19280716	1	20131127	5
11656750	20110817	15632665	19641118	2	20110817	6
11672825	20121217	17080610	19300504	1	20121217	5
11718368	20130717	17726706	19260127	1	20130717	6
11722499	20110421	15285938	19370817	2	20110421	5
11752435	20120425	16361353	19410405	2	20120425	5
11779387	20130526	17560477	19810226	2	20130526	4
11826872	20130913	17914798	19250319	1	20130913	4
11842221	20110503	15320085	19530720	2	20110503	5
11860450	20130513	17523787	19230113	2	20130513	6
11972115	20110531	15396869	19530610	1	20110531	5
11972966	20120130	16095132	19380103	2	20120130	5
12075886	20110503	15320119	19501110	2	20110503	4
12089382	20120207	16127677	19520503	2	20120207	4
12110097	20110914	15710549	19510623	2	20110914	4
12116459	20120428	16369054	19230809	2	20120428	4
12154471	20110324	15203700	19511028	1	20110324	4
12207511	20120220	16168369	19470608	2	20120220	4
12210230	20131107	18088510	19600827	2	20131107	4
12219953	20110809	15609021	19630917	2	20110809	6
12227031	20130930	17957583	19350209	2	20130930	6
12241122	20121111	16963493	19560425	2	20121111	6
12245635	20110630	15484912	19400716	2	20110630	6
12370160	20110808	15604953	19420126	2	20110808	4
12441348	20110810	15612981	19680417	1	20110810	4
12444676	20130518	17540710	19511220	1	20130518	4
12456609	20111218	15987026	19620218	1	20111218	5
12475159	20110324	15205468	19600712	2	20110324	6
12494596	20110823	15648047	19490720	2	20110823	5
12501461	20131116	18117285	19411020	2	20131116	6
12506068	20110610	15432201	19511210	1	20110610	5
12538055	20131103	18066549	19341204	1	20131103	5
12548515	20121106	16947701	19290309	1	20121106	6
12614947	20120926	16824143	19291205	1	20120926	6
12632574	20130516	17535337	19520310	2	20130516	4

12640276	20120823	16725233	19460310	2	20120823	5
12693037	20131214	18207062	19550505	1	20131214	6
12714668	20111019	15811899	19451117	2	20111019	5
12723238	20120307	16219066	19361202	2	20120307	5
12727025	20121123	17004288	19250919	1	20121123	5
12777081	20131007	17986887	19590702	2	20131007	4
12819806	20131122	18135970	19271215	1	20131122	5
12849295	20131112	18101637	19490707	1	20131112	5
13022510	20130218	17254120	19340825	2	20130218	5
13047091	20120209	16136228	19761010	1	20120209	4
13060894	20130425	17466972	19260611	1	20130425	4
13112339	20131229	18245948	19590330	1	20131229	5
13140346	20130620	17640222	19590803	1	20130620	6
13162179	20111204	15943229	19320505	2	20111204	4
13223295	20111025	15825760	19210303	1	20111025	4
13230325	20131016	18013971	19640224	1	20131016	5
13277731	20120626	16545613	19651017	2	20120626	4
13287440	20120805	16664735	19271224	2	20120805	5
13347778	20110401	15226692	19501212	2	20110401	5
13378273	20121005	16853237	19310125	1	20121005	5
13425417	20130219	17260120	19631111	1	20130219	5
13519749	20120409	16314516	19821123	1	20120409	6
13528682	20130116	17170270	19320608	1	20130116	5
13561825	20120307	16219177	19280129	2	20120307	6
13737934	20111019	15811871	19670601	2	20111019	6
13816392	20131217	18216008	19621217	2	20131217	6
13833039	20111111	15880052	19530807	1	20111111	5
13840374	20120207	16128022	19281229	1	20120207	4
13883573	20130604	17587135	19590201	1	20130604	5
13939372	20110428	15305199	19470815	1	20110428	5
13973701	20130813	17814068	19501123	1	20130813	4
14015668	20130724	17749123	19480903	1	20130724	6
14080694	20120529	16460683	19541226	1	20120529	4
14101361	20131017	18019162	19310319	1	20131017	4
14124584	20111123	15913300	19480725	1	20111123	5
14125156	20120425	16359740	19441117	2	20120425	4
14224352	20121028	16917042	19570317	1	20121028	6
14300324	20130117	17173732	19330215	2	20130117	6
14301065	20130624	17650783	19541210	1	20130624	5
14368195	20130729	17760063	19220101	1	20130729	4
14378280	20121004	16851032	19350823	1	20121004	5
14408594	20130720	17737807	19470327	1	20130720	5
14445944	20130413	17431130	19670331	1	20130413	6
14447177	20110910	15701668	19520813	1	20110910	4
14476621	20130102	17122390	19410305	1	20130102	5
14495911	20110914	15710571	19560912	1	20110914	5
14568628	20131021	18028536	19511205	2	20131021	4
14751167	20120927	16825294	19360718	2	20120927	5
14768640	20111130	15929913	19341120	1	20111130	6
14798788	20110626	15473718	19301214	1	20110626	6
14814870	20121026	16914993	19320819	1	20121026	5
14842376	20110920	15725902	19290405	1	20110920	4
14842581	20110621	15463154	19570627	1	20110621	4
14897859	20120701	16556889	19280523	1	20120701	5
15022089	20131218	18218948	19251126	1	20131218	6

15025646	20121205	17041006	19590220	2	20121205	5
15039891	20130809	17804318	19590508	2	20130809	6
15075204	20120524	16450022	19670714	1	20120524	4
15079693	20110823	15648029	19370705	1	20110823	5
15153896	20130909	17896654	19260611	1	20130909	4
15171365	20110407	15242892	19460214	2	20110407	4
15207008	20130409	17415567	19310620	1	20130409	5
15217706	20110309	15162558	19420703	2	20110309	6
15254598	20130527	17562984	19300730	1	20130527	6
15261184	20110804	15594204	19521211	1	20110804	6
15277368	20120820	16715196	19340307	1	20120820	6
15306146	20120624	16538078	19520221	2	20120624	6
15349914	20121113	16969906	19250924	1	20121113	6
15370033	20120522	16443516	19270512	1	20120522	5
15371003	20110823	15647648	19350225	2	20110823	4
15379096	20130116	17171687	19350830	2	20130116	6
15391089	20130910	17902990	19330525	2	20130910	5
15400881	20120920	16808921	19500121	2	20120920	4
15509350	20130128	17202421	19400916	2	20130128	6
15572164	20120419	16346598	19430510	1	20120419	4
15601840	20130417	17442746	19451012	1	20130417	6
15634692	20110325	15208459	19350122	1	20110325	5
15640887	20110430	15308711	19330320	1	20110430	4
15681057	20120219	16164154	19590819	1	20120219	5
15705836	20130723	17744557	19260614	1	20130723	4
15735123	20110728	15570881	19600626	2	20110728	5
15736320	20130530	17572080	19540929	1	20130530	6
15737436	20110406	15238202	19520606	1	20110406	6
15752746	20110225	15123575	19521028	1	20110225	5
15759383	20120227	16184997	19680929	1	20120227	5
15787287	20131029	18052545	19320320	1	20131029	6
15807597	20120330	16283720	19320410	1	20120330	5
15816009	20110805	15598704	19440205	2	20110805	5
15861399	20120304	16204129	19290508	2	20120304	6
15867364	20120731	16651266	19311223	1	20120731	5
15885800	20130516	17535030	19481213	2	20130516	4
15897311	20110323	15201174	19471001	2	20110323	5
15911667	20130917	17924001	19430728	1	20130917	6
15918919	20120628	16551227	19590204	2	20120628	6
15925969	20110414	15267588	19470525	2	20110414	4
15965998	20120408	16310815	19560901	2	20120408	5
16084530	20120306	16214448	19600224	1	20120306	5
16095822	20110824	15651317	19501027	2	20110824	4
16116771	20110412	15259080	19580415	1	20110412	6
16184284	20110622	15466287	19410311	1	20110622	5
16202996	20120509	16408326	19540826	1	20120509	5
16242005	20130730	17763193	19320529	1	20130730	6
16268998	20130417	17441761	19851214	1	20130417	6
16272881	20110824	15649936	19460707	2	20110824	6
16276383	20130601	17578099	19540812	2	20130601	6
16281882	20120508	16401316	19500216	1	20120508	4
16284972	20110731	15575128	19310812	1	20110731	4
16301783	20110628	15479440	19310801	1	20110628	5
16323969	20131118	18120620	19320405	1	20131118	5
16328668	20130329	17381348	19500301	2	20130329	5

16356744	20131104	18071061	19300603	1	20131104	5
16453568	20121223	17097415	19300319	1	20121223	4
16472154	20110410	15249818	19270304	2	20110410	4
16479417	20120107	16046660	19340723	2	20120107	4
16526424	20110613	15438140	19480210	1	20110613	5
16541507	20131106	18082693	19330815	1	20131106	4
16558660	20110906	15687833	19500128	1	20110906	6
16621731	20120106	16045081	19500325	1	20120106	4
16661339	20130227	17285641	19481201	2	20130227	5
16677002	20131020	18025972	19300520	1	20131020	4
16697113	20110731	15575004	19680524	2	20110731	4
16747141	20120619	16527608	19400420	2	20120619	6
16771350	20110731	15574967	19281229	1	20110731	5
16786940	20130314	17339141	19230713	1	20130314	6
16815546	20120309	16226068	19250202	1	20120309	5
16825506	20130520	17545034	19560605	2	20130520	5
16894023	20131027	18045928	19690608	1	20131027	6
16916862	20110317	15187380	19620702	2	20110317	4
17026458	20130716	17723622	19521004	2	20130716	5
17034445	20120731	16650548	19540912	1	20120731	5
17049466	20131029	18051501	19390102	2	20131029	6
17082110	20111203	15942577	19470320	2	20111203	4
17094814	20120325	16268579	19550910	2	20120325	4
17097722	20111102	15850595	19320329	1	20111102	5
17143565	20120112	16061962	19281107	1	20120112	6
17176259	20130710	17707336	19670617	1	20130710	5
17200769	20130531	17575668	19280820	1	20130531	6
17205786	20130730	17764011	19500401	2	20130730	4
17224509	20111218	15987076	19290722	2	20111218	4
17233306	20120416	16337200	19280601	1	20120416	5
17241235	20111204	15943147	19291122	1	20111204	4
17253111	20120330	16283579	19460317	2	20120330	6
17264425	20110917	15718522	19330616	1	20110917	6
17272069	20130116	17171742	19530907	1	20130116	6
17292432	20120315	16245210	19680902	2	20120315	5
17330584	20130617	17629381	19861110	1	20130617	4
17331703	20121004	16850056	19480808	2	20121004	4
17338599	20130826	17850578	19550730	1	20130826	5
17343747	20120630	16556169	19310722	1	20120630	5
17366824	20120413	16331570	19350301	1	20120413	5
17381930	20130403	17399678	19551224	1	20130403	5
17407975	20120913	16788224	19590112	2	20120913	6
17430432	20130708	17697782	19380608	1	20130708	5
17486429	20120926	16824724	19501224	2	20120926	5
17491757	20110802	15585891	19220223	1	20110802	6
17507867	20120217	16161872	19320404	1	20120217	5
17543634	20120926	16824455	19510223	2	20120926	5
17563507	20131008	17991871	19310728	1	20131008	6
17586071	20120807	16675454	19410721	2	20120807	4
17599154	20120811	16688880	19590921	2	20120811	6
17663762	20120715	16606668	19210406	1	20120715	4
17693797	20131016	18014553	19460212	1	20131016	6
17699488	20111031	15840643	19480603	1	20111031	5
17717714	20130120	17180670	19521022	1	20130120	5
17717816	20130106	17135489	19350717	1	20130106	4

17723921	20121102	16931740	19571013	2	20121102	4
17724811	20120330	16283696	19310410	2	20120330	5
17789423	20110525	15384458	19330216	2	20110525	4
17827315	20120925	16821526	19550306	1	20120925	4
17841428	20120215	16155704	19701028	1	20120215	6
17967101	20110518	15366881	19640409	2	20110518	5
17967929	20110601	15403112	19630725	1	20110601	6
18023217	20130221	17265104	19300323	1	20130221	5
18072838	20121022	16902688	19340930	1	20121022	5
18093306	20130305	17307931	19501213	2	20130305	5
18159814	20130915	17917449	19390326	2	20130915	6
18162055	20111124	15916787	19490702	1	20111124	5
18180499	20130602	17578608	19540316	1	20130602	5
18199398	20130215	17248632	19370219	2	20130215	6
18235397	20120416	16335811	19491007	2	20120416	5
18235886	20131120	18129227	19610326	2	20131120	5
18267748	20130523	17555111	19621223	1	20130523	4
18280767	20120312	16233122	19430221	2	20120312	4
18293657	20130404	17402161	19320112	1	20130404	5
18305823	20130120	17180488	19510115	2	20130120	6
18346617	20121008	16863011	19350515	1	20121008	6
18368746	20130128	17202765	19260623	1	20130128	4
18435808	20110512	15351603	19320205	1	20110512	6
18462867	20130321	17360245	19570907	1	20130321	6
18476329	20110609	15428672	19470827	1	20110609	4
18493282	20120918	16801852	19380104	1	20120918	4
18551025	20120813	16694463	19300406	1	20120813	5
18585109	20120612	16507999	19330228	1	20120612	5
18601622	20120704	16573317	19390311	1	20120704	5
18639844	20110821	15641384	19500520	2	20110821	4
18656934	20130107	17140614	19391124	1	20130107	5
18684792	20130903	17876523	19300203	1	20130903	6
18714766	20110430	15308104	19561028	2	20110430	6
18799934	20121003	16847107	19560120	2	20121003	5
18831419	20111208	15958846	19540628	1	20111208	5
18861693	20120910	16777448	19570909	1	20120910	4
18887177	20130503	17491926	19260507	1	20130503	5
18931721	20131126	18144067	19420624	1	20131126	5
18936373	20110310	15166867	19550520	2	20110310	4
18966502	20111228	16015395	19300625	1	20111228	6
18995614	20110311	15170438	19270101	2	20110311	5
19005200	20130818	17828934	19611203	1	20130818	4
19018430	20120413	16331376	19400710	2	20120413	6
19044054	20110503	15320070	19620414	1	20110503	5
19049413	20130526	17560167	19310416	1	20130526	6
19063628	20131123	18136169	19630123	1	20131123	5
19103721	20120810	16686496	19510218	2	20120810	4
19107290	20131125	18140493	19520112	2	20131125	6
19185047	20111208	15960973	19630830	2	20111208	6
19235699	20130601	17577963	19360827	1	20130601	6
19238392	20130616	17625588	19631016	1	20130616	5
19242149	20110506	15332838	19880319	2	20110506	6
19300573	20110424	15292597	19580702	1	20110424	4
19311558	20110602	15406422	19670608	2	20110602	4
19347898	20110623	15467894	19220626	1	20110623	5

19351554	20120608	16497227	19300412	2	20120608	5
19381205	20110218	15103607	19400515	2	20110218	5
19421955	20130227	17284404	19450416	1	20130227	6
19445228	20120618	16524730	19370926	1	20120618	5
19445477	20110918	15719241	19330509	1	20110918	6
19493120	20111229	16017697	19240904	2	20111229	5
19573945	20130524	17556317	19581213	2	20130524	5
19623144	20111118	15901459	19160306	1	20111118	4
19635473	20120927	16825506	19341223	2	20120927	6
19645080	20120725	16637239	19591012	2	20120725	5
19654570	20130624	17650558	19280407	1	20130624	5
19672458	20121008	16861492	19660413	1	20121008	6
19684801	20121126	17010034	19700601	1	20121126	5
19715232	20121231	17117280	19270219	1	20121231	4
19726900	20110419	15280177	19571020	2	20110419	6
19761843	20111001	15755653	19351016	2	20111001	5
19825662	20110824	15651076	19310802	1	20110824	5
19841419	20110816	15629784	19581105	1	20110816	6
19889328	20130310	17324348	19490218	1	20130310	4
19893744	20121029	16920412	19310920	2	20121029	4
19903152	20111115	15889522	19330213	2	20111115	6
19915969	20120820	16715007	19250615	2	20120820	6
19920764	20110416	15272319	19470201	1	20110416	4
19976739	20130316	17345090	19520102	1	20130316	6
20032513	20120403	16297026	19380513	1	20120403	4
20060382	20120415	16333631	19330518	2	20120415	5
20102696	20131009	17996550	19560801	2	20131009	5
20120734	20130219	17260170	19380616	2	20130219	4
20144803	20130529	17568140	19271210	1	20130529	5
20178705	20121231	17116893	19430218	1	20121231	4
20216084	20110309	15162378	19560809	2	20110309	5
20240408	20131126	18144912	19570305	1	20131126	6
20300612	20130916	17921561	19310203	2	20130916	6
20317935	20131109	18094673	19491226	1	20131109	5
20358014	20120204	16118491	19160412	1	20120204	6
20426760	20110809	15609002	19331111	1	20110809	5
20433312	20120512	16417086	19260909	1	20120512	4
20481485	20120705	16576513	19271005	2	20120705	5
20482671	20121026	16914745	19261214	1	20121026	5
20485023	20110809	15608866	19220508	2	20110809	5
20520483	20121031	16925354	19540323	2	20121031	5
20536270	20110319	15192001	19330505	1	20110319	6
20556109	20120318	16250499	19240926	1	20120318	5
20581311	20110327	15210216	19630819	1	20110327	6
20615672	20120509	16408232	19270902	2	20120509	6
20617189	20120419	16347081	19591215	1	20120419	6
20655167	20111002	15756738	19541020	2	20111002	4
20663267	20111210	15966101	19290430	1	20111210	6
20697067	20110526	15386884	19690717	2	20110526	6
20718767	20120731	16650944	19280324	2	20120731	4
20819516	20110406	15238853	19461226	2	20110406	4
20850800	20110831	15664940	19370908	1	20110831	4
20859056	20121202	17025214	19351109	2	20121202	6
20898106	20120312	16233963	19900607	2	20120312	6
20912452	20121112	16967658	19430819	1	20121112	6

20921851	20111224	16005243	19300120	1	20111224	4
20934536	20130328	17378933	19550428	1	20130328	4
20940345	20120613	16511804	19791025	1	20120613	4
20969942	20131030	18055424	19280108	1	20131030	5
20996069	20131101	18061116	19680930	1	20131101	5
21050691	20111006	15775822	19480920	2	20111006	4
21052959	20120531	16466210	19500225	1	20120531	5
21112118	20130930	17956707	19691009	1	20130930	5
21152885	20130303	17296786	19680531	1	20130303	6
21184985	20120408	16311003	19510223	1	20120408	4
21244451	20131001	17963540	19900816	1	20131001	4
21261234	20110805	15598634	19280707	2	20110805	6
21292422	20130603	17583502	19230605	1	20130603	6
21343179	20130904	17882255	19360220	1	20130904	5
21348823	20121026	16914722	19420824	2	20121026	6
21357697	20110407	15243193	19480824	1	20110407	5
21374981	20110729	15572920	19260901	2	20110729	4
21461494	20120624	16537905	19350908	1	20120624	6
21493925	20120224	16179698	19400506	2	20120224	6
21519437	20111001	15756207	19350219	1	20111001	5
21550630	20120830	16741441	19420201	2	20120830	5
21573240	20121023	16905487	19620105	2	20121023	6
21575268	20110516	15359684	19470216	2	20110516	4
21593704	20111104	15860053	19230707	2	20111104	4
21600806	20110621	15461936	19501203	2	20110621	6
21634695	20130120	17180507	19680713	2	20130120	5
21655958	20130112	17159246	19601105	1	20130112	4
21699490	20120211	16142655	19371001	1	20120211	5
21753059	20120203	16116876	19890524	2	20120203	6
21769335	20130526	17560396	19251218	1	20130526	5
21803054	20130221	17268100	19460604	2	20130221	5
21822913	20111228	16014992	19360324	2	20111228	5
21846093	20111225	16005765	19560120	1	20111225	6
21893970	20120514	16420397	19600713	2	20120514	4
21912572	20110818	15635664	19520128	1	20110818	5
21916018	20130215	17247654	19440304	2	20130215	5
21918832	20130728	17757815	19300719	1	20130728	6
21941662	20110423	15291847	19690322	1	20110423	6
21943759	20131119	18125630	19560210	1	20131119	5
21960838	20111225	16005950	19370116	2	20111225	4
22012244	20110215	15091218	19441201	1	20110215	5
22015118	20121011	16872244	19470201	2	20121011	5
22023172	20120117	16074536	19371017	2	20120117	5
22063189	20120116	16073736	19420515	1	20120116	4
22081578	20110310	15166879	19421125	1	20110310	6
22154978	20131103	18066653	19320901	2	20131103	5
22405910	20120501	16379114	19590922	1	20120501	5
22513979	20110821	15641382	19370419	2	20110821	6
22528718	20120813	16693495	19240420	1	20120813	4
22532769	20121013	16877747	19490815	2	20121013	5
22536374	20120218	16163887	19400629	1	20120218	4
22575980	20110318	15190447	19580429	2	20110318	5
22601816	20111109	15873580	19280613	2	20111109	4
22608613	20131126	18144029	19501218	2	20131126	4
22611150	20111226	16009106	19440822	1	20111226	6

22648891	20111127	15921748	19470428	1	20111127	5
22677232	20110627	15477143	19391020	2	20110627	5
22694899	20130419	17450221	19290510	2	20130419	4
22727599	20120209	16137136	19330118	1	20120209	5
22727839	20120623	16537336	19370513	2	20120623	6
22738825	20120516	16427714	19590609	2	20120516	6
22742514	20111206	15952403	19440324	1	20111206	5
22792310	20131204	18170516	19410129	2	20131204	5
22831454	20130103	17125047	19351012	1	20130103	5
22851612	20121021	16899333	19600107	1	20121021	5
22863565	20130127	17200105	19541020	1	20130127	6
22971375	20131016	18012319	19470309	2	20131016	4
23005529	20110827	15658663	19321211	1	20110827	6
23025914	20130102	17122781	19560720	1	20130102	5
23033605	20110624	15472173	19300128	1	20110624	6
23087867	20130811	17806532	19260506	1	20130811	4
23099856	20121111	16963574	19470417	2	20121111	6
23101726	20131029	18051215	19380215	1	20131029	5
23103448	20130609	17605809	19410320	1	20130609	5
23105284	20120814	16698253	19400405	2	20120814	4
23129364	20120717	16614676	19570226	1	20120717	4
23133520	20120822	16721329	19440420	2	20120822	6
23157199	20120601	16466651	19460714	2	20120601	4
23178361	20111212	15970718	19340322	1	20111212	5
23194254	20120104	16037227	19600510	2	20120104	5
23242162	20131219	18220044	19330607	2	20131219	5
23252133	20130928	17954408	19520401	2	20130928	4
23258039	20130311	17328620	19570528	2	20130311	4
23311220	20110310	15164363	19441123	2	20110310	5
23344130	20110504	15320212	19481206	2	20110504	4
23369199	20130408	17411300	19290915	2	20130408	6
23448262	20111106	15862412	19351013	1	20111106	6
23467154	20130226	17281439	19710621	2	20130226	4
23493927	20131228	18245346	19570401	1	20131228	6
23497725	20120902	16747022	19640507	1	20120902	4
23523991	20110530	15394589	19560810	1	20110530	5
23534523	20131211	18199225	19540221	2	20131211	5
23540310	20130220	17264262	19420705	2	20130220	4
23557724	20111024	15823321	19210409	1	20111024	5
23601265	20110208	15065848	19580623	1	20110208	6
23614144	20110423	15291960	19290226	2	20110423	4
23711571	20120229	16189873	19410410	2	20120229	5
23764854	20110209	15073115	19270606	1	20110209	4
23815454	20131218	18219311	19521104	2	20131218	4
23859747	20120603	16474619	19510828	1	20120603	6
23945177	20131029	18050923	19580906	2	20131029	5
23955319	20120715	16606973	19840427	1	20120715	4
24045623	20130108	17144920	19470508	1	20130108	4
24048757	20120116	16072956	19400124	1	20120116	6
24070404	20111120	15903538	19250204	1	20111120	5
24094186	20120409	16315648	19431001	1	20120409	5
24101175	20111011	15785064	19500325	1	20111011	5
24146836	20110810	15612855	19401014	2	20110810	6
24188601	20121223	17097404	19610312	2	20121223	5
24217627	20131102	18065970	19460607	2	20131102	5

24224804	20120514	16421575	19240108	2	20120514	5
2436868120130203		17220614	19580418	1	20130203	5
24397624	20110616	15450164	19290308	1	20110616	4
24415701	20120603	16474685	19651216	1	20120603	6
24424848	20121022	16903122	19260302	1	20121022	5
24459776	20111202	15940620	19740226	1	20111202	5
24596585	20121105	16943724	19610907	2	20121105	5
2460204820130908		17893722	19580509	2	20130908	4
24694802	20120311	16229832	19551213	2	20120311	4
24711420	20120531	16465788	19300922	1	20120531	6
24720750	20121012	16875028	19280724	1	20121012	6
24748912	20111019	15810780	19250925	1	20111019	5
2478632120130402		17395207	19350102	2	20130402	5
24817898	20110615	15447110	19230910	1	20110615	4
2488225520131002		17967039	19330530	1	20131002	5
24896842	20110917	15718687	19590815	2	20110917	4
24904356	20110711	15522882	19411018	1	20110711	6
24906103	20110627	15476559	19631101	1	20110627	5
2491304020130429		17474527	19311110	1	20130429	6
24918136	20110401	15226858	19531208	1	20110401	5
24921026	20120402	16291290	19700521	1	20120402	6
24952781	20110814	15622334	19370715	1	20110814	5
2495627220130425		17467175	19261012	2	20130425	5
24968910	20121211	17063017	19370905	2	20121211	6
2502265520131128		18151467	19440622	2	20131128	5
25023636	20120607	16489641	19470525	2	20120607	6
25031327	20110322	15198528	19391101	2	20110322	5
2509120720130903		17877109	19580923	1	20130903	5
2509426220130730		17763463	19400512	1	20130730	6
25131877	20120413	16331618	19300214	2	20120413	6
25156869	20110607	15419919	19530724	2	20110607	5
2516754820130318		17349617	19560110	2	20130318	5
2518846920130101		17117804	19481227	2	20130101	5
25208859	20120410	16320127	19291116	2	20120410	5
25238964	20110428	15304528	19281226	1	20110428	5
25316114	20120503	16386919	19590626	1	20120503	4
25435498	20110307	15154429	19450430	2	20110307	4
25448855	20111122	15909564	19241103	1	20111122	6
25462811	20111108	15870401	19540802	1	20111108	5
25495801	20120626	16545154	19501214	1	20120626	5
25524145	20120302	16200623	19370803	2	20120302	6
25558898	20120808	16678932	19740324	2	20120808	6
25574178	20111017	15803620	19520824	2	20111017	5
25578454	20110726	15563116	19280926	1	20110726	6
2560134720130930		17957842	19341010	1	20130930	4
25620013	20110224	15121241	19600716	2	20110224	5
25627263	20110828	15659055	19610414	1	20110828	4
25631510	20120212	16143279	19451014	1	20120212	4
2570178420131119		18125930	19460915	2	20131119	4
25704807	20120219	16164197	19341205	2	20120219	6
2570785120131119		18122269	19521101	2	20131119	6
25727406	20110226	15125721	19601225	1	20110226	6
25733635	20121029	16918516	19370124	2	20121029	5
25744847	20111226	16009291	19630708	2	20111226	5
2577700820131010		17997202	19340328	1	20131010	5

25782847	20130129	17206630	19411214	1	20130129	6
25837529	20120426	16364673	19381017	1	20120426	6
25852044	20130104	17132483	19400106	1	20130104	4
25862797	20130223	17273740	19500808	1	20130223	5
25874333	20111211	15966518	19310115	1	20111211	5
25879714	20120110	16056144	19510126	1	20120110	5
25897670	20111004	15766819	19510106	1	20111004	4
25935380	20110606	15414866	19380502	2	20110606	5
25935788	20131130	18156167	19280810	1	20131130	6
26003112	20110314	15174176	19350220	2	20110314	5
26007567	20110821	15641162	19700514	1	20110821	4
26068928	20111026	15830512	19920803	1	20111026	5
26117135	20130929	17954830	19300901	1	20130929	5
26130643	20111227	16012590	19420630	2	20111227	6
26248191	20111109	15871937	19491209	2	20111109	5
26288459	20120604	16479938	19281212	1	20120604	4
26299729	20120103	16030799	19461028	2	20120103	4
26460491	20120219	16164181	19640328	1	20120219	5
26493356	20130821	17838849	19560520	2	20130821	5
26571937	20130509	17513282	19530625	2	20130509	5
26581248	20110522	15375579	19970529	2	20110522	5
26625898	20110808	15605065	19350605	2	20110808	5
26633294	20120229	16190179	19340808	1	20120229	6
26647734	20131007	17986521	19810806	2	20131007	6
26678002	20120708	16584383	19480205	2	20120708	5
26690755	20110220	15106422	19281114	1	20110220	5
26692795	20110801	15578361	19641019	2	20110801	4
26696082	20120508	16403336	19540606	1	20120508	5
26742869	20121118	16985886	19720710	2	20121118	6
26840960	20111107	15865973	19550925	1	20111107	6
26845498	20121011	16871196	19440520	1	20121011	6
26934850	20130616	17625496	19590225	1	20130616	4
26948436	20111123	15913896	19321004	1	20111123	5
26970467	20120813	16693848	19760614	2	20120813	6
27041607	20121017	16889872	19540504	2	20121017	4
27128081	20110318	15190239	19650609	2	20110318	5
27158141	20121004	16851469	19310120	2	20121004	5
27202875	20130222	17271675	19391029	1	20130222	4
27235547	20120501	16377632	19510425	2	20120501	5
27257132	20110810	15612564	19420102	1	20110810	5
27275236	20130114	17163139	19290519	1	20130114	4
27278724	20131211	18198678	19721015	2	20131211	6
27285058	20110608	15423661	19490725	1	20110608	6
27285649	20131105	18076724	19400324	1	20131105	5
27367244	20120313	16237858	19441203	2	20120313	6
27395217	20121120	16992178	19380318	1	20121120	6
27433803	20120920	16808666	19421209	2	20120920	6
27453174	20130409	17416125	19520215	1	20130409	5
27473218	20111011	15786739	19400110	2	20111011	6
27544703	20120814	16699149	19470610	1	20120814	4
27558970	20110621	15462969	19321011	2	20110621	6
27575593	20110329	15216364	19621120	1	20110329	4
27631170	20130411	17425328	19650607	2	20130411	5
27634226	20130321	17360690	19590120	1	20130321	5
27643476	20130815	17822151	19300127	1	20130815	6

27678633	20111227	16011203	19381222	2	20111227	4
27712545	20121116	16983429	19390104	2	20121116	5
27715000	20111026	15829568	19460911	2	20111026	5
27729620	20120115	16069566	19621010	1	20120115	6
27736807	20111021	15818126	19370118	2	20111021	4
2774035820130504		17495592	19461104	2	20130504	6
27745911	20120617	16520908	19460105	1	20120617	5
27783831	20120821	16717854	19550305	2	20120821	6
2780514720130416		17439481	19520818	2	20130416	6
2781738520130612		17615133	19460525	2	20130612	4
2782687520131121		18131734	19610301	1	20131121	5
2785343620130929		17954834	19670225	1	20130929	5
27858737	20120216	16157451	19200124	1	20120216	5
27859525	20120207	16128771	19380312	2	20120207	6
27865163	20121125	17006613	19540315	2	20121125	5
27879114	20110629	15483131	19650305	2	20110629	6
2790442320130213		17243807	19460802	1	20130213	6
27938438	20121001	16836229	19420616	2	20121001	5
27963935	20120129	16094810	19460908	2	20120129	4
2804075720130520		17544745	19410316	1	20130520	4
28091943	20120315	16245160	19990204	1	20120315	5
28127471	20121212	17066248	19550828	1	20121212	6
28154361	20110620	15458767	19400211	2	20110620	4
28170345	20111013	15795314	19460520	1	20111013	6
28214655	20110901	15670742	19530622	2	20110901	6
2821722320130214		17246352	19521120	2	20130214	5
28228719	20120514	16420632	19540403	1	20120514	5
28280491	20111020	15814447	19370615	2	20111020	4
28327131	20121104	16938805	19520823	1	20121104	4
28337000	20120402	16288762	19270305	1	20120402	5
2834238320130411		17425660	19410622	1	20130411	5
2835870520130917		17924250	19751022	1	20130917	4
2836086320130710		17707138	19501226	1	20130710	4
28377119	20111123	15913713	19320210	2	20111123	5
2844074620130828		17857160	19440515	2	20130828	4
28451129	20120129	16094487	19310821	1	20120129	6
28517411	20120411	16323405	19450314	2	20120411	6
28567580	20121127	17013292	19271110	2	20121127	4
2857801020130409		17416983	19530620	2	20130409	4
28585902	20120811	16690017	19500302	2	20120811	5
2861662820130409		17417096	19561214	1	20130409	4
2863356920130401		17388633	19571101	1	20130401	5
28643369	20110629	15482180	19640409	2	20110629	4
28643687	20110924	15737795	19750327	1	20110924	5
28676917	20111101	15845191	19390408	2	20111101	4
28733688	20111219	15990904	19370514	2	20111219	6
28778576	20111119	15902988	19380803	1	20111119	4
28844726	20120604	16478926	19371104	2	20120604	4
28952536	20111031	15840867	19450122	2	20111031	6
28959606	20120312	16234003	19420308	1	20120312	6
29147215	20121004	16850337	20000912	1	20121004	5
2917152620130710		17705332	19410509	2	20130710	6
29181893	20110222	15112702	19420103	2	20110222	4
2919447620130406		17406878	19700723	2	20130406	5
29207156	20121024	16908916	19480313	2	20121024	5

29269736	20121119	16988422	19291211	1	20121119	6
29298699	20131205	18178333	19360610	2	20131205	5
29388816	20120107	16046862	19860429	1	20120107	4
29454475	20130731	17766268	19641009	2	20130731	6
29511204	20130828	17858054	19490228	1	20130828	5
29547602	20110411	15253240	19641020	2	20110411	6
29562536	20110209	15073466	19540802	1	20110209	6
29566232	20130417	17442422	19521229	1	20130417	6
29600304	20120726	16639970	19591003	1	20120726	4
29618028	20121013	16877804	19410928	2	20121013	4
29625670	20120621	16534179	19600220	1	20120621	6
29730630	20111114	15887043	19400403	1	20111114	6
29731122	20120415	16333388	19420416	2	20120415	4
29758303	20131218	18218413	19490120	1	20131218	6
29774923	20120725	16637458	19270923	2	20120725	6
29792970	20131119	18125570	19270104	1	20131119	5
29823594	20120708	16584243	19910911	1	20120708	5
29850224	20131124	18138068	19590530	1	20131124	4
29882039	20120925	16820713	19500718	1	20120925	6
29932727	20130424	17463829	19360916	1	20130424	5
29940010	20110501	15309052	19451113	2	20110501	5
29951813	20110324	15205383	19630923	2	20110324	6
29964703	20120318	16250550	19611202	1	20120318	6
29983640	20121104	16938444	19551204	1	20121104	6
29987357	20110908	15695634	19360221	1	20110908	6
30007477	20130110	17152350	19520730	1	20130110	5
30132924	20130303	17296716	19350810	2	20130303	6
30144140	20110612	15434407	19441220	1	20110612	5
30150711	20130811	17806782	19520217	1	20130811	4
30171392	20110910	15701712	19340508	1	20110910	5
30200307	20120317	16250126	19331226	1	20120317	4
30272243	20130422	17457022	19480504	1	20130422	5
30283706	20110212	15084201	19440221	1	20110212	5
30288483	20130708	17697800	19580916	2	20130708	6
30290723	20110123	15033909	19660524	1	20110123	5
30293960	20110906	15687150	19620616	1	20110906	4
30323810	20121030	16921644	19420914	1	20121030	4
30335230	20131018	18022197	19620314	2	20131018	4
30366064	20121011	16870640	19750126	1	20121011	5
30406043	20120513	16417530	19580410	1	20120513	4
30427919	20110310	15165619	19540516	1	20110310	6
30457217	20110827	15658561	19371004	1	20110827	5
30496367	20131202	18159154	19420105	2	20131202	4
30515389	20111225	16005879	19430712	2	20111225	4
30521676	20110325	15208531	19620626	2	20110325	6
30556617	20120611	16503317	19280104	2	20120611	4
30559047	20120812	16690403	19620223	1	20120812	4
30592893	20120920	16809251	19560514	1	20120920	4
30599338	20120704	16571628	19420920	2	20120704	5
30607751	20120610	16500092	19550112	1	20120610	6
30623859	20131227	18243904	19370612	2	20131227	6
30649220	20110415	15268818	19270515	2	20110415	4
30678070	20130714	17716111	19481103	1	20130714	4
30685597	20130326	17371965	19600310	1	20130326	5
30696630	20110210	15078213	19541215	2	20110210	6

30701929	20121010	16867909	19570622	1	20121010	5
30709081	20120508	16404117	19481102	2	20120508	5
30716724	20110904	15679064	19780618	1	20110904	4
3073845520130119		17179381	19770920	2	20130119	6
3074119620131104		18070479	19650116	1	20131104	4
30765992	20111218	15987300	19310831	1	20111218	6
30806407	20110316	15183357	19320328	2	20110316	4
30873906	20120618	16524180	19271015	1	20120618	6
3089687820130808		17799674	19431004	1	20130808	5
3091785220131017		18017057	19270626	2	20131017	6
30926659	20110318	15188959	19620808	2	20110318	6
30944800	20110218	15104139	19321216	2	20110218	5
3097051520130731		17766356	19211016	2	20130731	6
31006061	20120531	16465104	19361026	2	20120531	5
31026194	20121003	16845651	19551002	1	20121003	4
3104393320130215		17246588	19360823	1	20130215	4
31058249	20110212	15084162	19751219	2	20110212	6
31095359	20120126	16090837	19340702	1	20120126	5
31109894	20120702	16559877	19560702	1	20120702	5
3113711820131115		18115159	19650821	2	20131115	4
31166800	20120725	16637556	19640207	2	20120725	5
31167847	20120507	16395623	19500115	2	20120507	5
3118534920131223		18231347	19570508	1	20131223	6
3118662620130616		17625479	19390728	2	20130616	5
31206903	20110405	15233802	19290111	1	20110405	6
31298529	20110331	15221072	19311026	1	20110331	4
31316117	20110307	15154483	19901004	1	20110307	5
3136706320130708		17698471	19450505	1	20130708	6
31382668	20111006	15775642	19371125	1	20111006	5
3138824620130320		17356783	19701018	1	20130320	5
3142432520130613		17619174	19461025	1	20130613	5
31460829	20110727	15566897	19281008	1	20110727	5
3146812920130711		17711554	19760822	1	20130711	5
31488912	20121210	17058027	19640820	2	20121210	4
3152730720131105		18078225	19420924	1	20131105	4
31549812	20120718	16618313	19520201	1	20120718	6
31558733	20121204	17036606	19500114	2	20121204	6
3162579320131029		18052624	19340318	1	20131029	5
31688343	20120913	16789117	19550405	1	20120913	5
3169845020130103		17125569	19490820	1	20130103	4
31698836	20111111	15880239	19640213	2	20111111	4
31729132	20120417	16340602	19390623	2	20120417	5
31736648	20121126	17010035	19550326	1	20121126	6
31738951	20120430	16372253	19300924	1	20120430	6
31750046	20110208	15068352	19660908	2	20110208	6
31819348	20110225	15124286	19451212	1	20110225	5
31883020	20110205	15059619	19500109	2	20110205	6
31888285	20120130	16098309	19490123	2	20120130	4
3190709220130929		17954511	19390702	2	20130929	6
31929825	20111107	15866559	19650925	1	20111107	5
31998693	20110326	15209872	19430101	2	20110326	4
32019742	20110917	15718744	19310504	1	20110917	6
3204088320130308		17319902	19491212	2	20130308	4
32053513	20110902	15676384	19580918	2	20110902	5
32068921	20111223	16003634	19430425	1	20111223	5

32092925	20111110	15877719	19311001	1	20111110	4
3213222220131019		18025278	19470115	1	20131019	5
32144448	20120105	16041183	19460812	2	20120105	4
32147094	20111021	15818449	19530331	1	20111021	5
32268965	20110629	15482866	19690226	1	20110629	5
32301476	20120221	16171720	19560812	2	20120221	4
3233180920130519		17540926	19430315	2	20130519	4
3233439720130901		17864943	19430812	1	20130901	5
3238612220130417		17442329	19470117	1	20130417	5
32397232	20110203	15057987	19380821	2	20110203	5
32446029	20111126	15921436	19500719	2	20111126	5
3246243420131216		18212693	19630820	1	20131216	5
3247538020130614		17621637	19380901	1	20130614	5
32477955	20110908	15696454	19341202	2	20110908	4
32501227	20110720	15549210	19391212	2	20110720	6
32509458	20110518	15366537	19450728	1	20110518	5
32515723	20120418	16341486	19510410	1	20120418	4
32553018	20111020	15814924	19260919	2	20111020	5
3260007020131119		18125557	19690620	2	20131119	6
32623680	20120727	16642361	19510715	2	20120727	6
32630641	20110330	15216453	19660120	1	20110330	5
32648343	20120408	16310887	19610717	1	20120408	4
32658745	20120225	16182217	19490528	1	20120225	5
32665422	20110922	15733376	19510521	1	20110922	5
32719076	20121219	17088533	19370311	2	20121219	6
3273179620130729		17760852	19601203	1	20130729	4
3277068620130108		17142558	19340620	2	20130108	5
3277649120130819		17832617	19420511	1	20130819	6
32796773	20110826	15657209	19541005	1	20110826	6
32811180	20121112	16967796	19640818	2	20121112	5
32811931	20110416	15272228	19321217	2	20110416	5
3283255620130321		17360816	19590516	2	20130321	4
32843779	20111205	15947825	19600203	1	20111205	6
32899164	20120228	16187597	19650320	2	20120228	4
32934682	20110315	15180573	19500311	2	20110315	6
32974439	20110510	15343823	19300211	1	20110510	4
32990537	20120904	16756029	19661226	1	20120904	6
33017457	20110313	15172710	19680421	1	20110313	6
33024838	20121005	16855642	19390614	1	20121005	5
33038083	20120814	16698840	19410616	1	20120814	5
3303952020130419		17449651	19321019	1	20130419	6
3306992020130623		17647235	19350828	1	20130623	5
3310134720130124		17195066	19500929	1	20130124	5
3310458420130103		17128437	19420316	2	20130103	5
33118331	20110225	15124305	19420927	2	20110225	4
3314112720131217		18216242	19240406	1	20131217	5
3320679020130826		17849891	19411121	1	20130826	6
33282961	20120213	16147029	19450907	1	20120213	6
33302646	20110315	15179952	19461113	2	20110315	5
3332060420130811		17806445	19560601	1	20130811	4
33339014	20121105	16940397	19741020	1	20121105	6
33353503	20120722	16626722	19420107	2	20120722	6
33367861	20110502	15313961	19640522	1	20110502	6
33433033	20120816	16706639	19370719	1	20120816	5
3344414320130109		17149006	19650921	2	20130109	6

33481833	20120710	16592705	19360712	1	20120710	5
33490389	20111031	15840851	19590320	2	20111031	6
33491702	20111209	15963359	19191216	2	20111209	5
33500902	20130929	17954941	19451009	2	20130929	5
33520228	20110928	15746313	19590220	2	20110928	4
33602470	20130906	17890065	19420211	1	20130906	4
33651808	20111104	15859092	19450908	1	20111104	6
33676390	20121217	17081553	19651228	1	20121217	5
33680921	20130908	17893702	19330215	1	20130908	6
33688787	20130122	17187377	19391022	1	20130122	4
33712082	20121119	16988901	19420614	1	20121119	6
33724606	20120423	16354118	19510113	2	20120423	5
33742744	20110919	15721748	19330511	2	20110919	4
33769674	20110406	15238698	19800911	2	20110406	6
33780766	20120201	16106851	19580308	1	20120201	4
33853939	20111221	15998055	19491006	1	20111221	5
33871680	20120904	16756285	19501128	1	20120904	6
33880216	20130103	17128239	19510425	2	20130103	6
33946622	20120210	16140740	19410317	1	20120210	5
33956466	20110611	15434107	19610614	1	20110611	4
33979849	20131231	18251121	19721117	2	20131231	4
34037542	20130104	17132466	19280821	1	20130104	4
34050743	20110426	15298678	19490413	1	20110426	6
34055602	20130310	17324256	19540205	2	20130310	6
34062958	20111223	16003685	19390815	1	20111223	5
34074287	20111014	15798170	19321107	1	20111014	4
34089537	20120609	16499619	19300327	1	20120609	4
34097784	20131212	18203248	19221201	2	20131212	5
34150704	20110613	15438891	19440411	1	20110613	4
34182768	20130502	17489125	19340716	1	20130502	4
34200389	20110408	15246589	19481229	2	20110408	6
34203957	20130913	17915000	19381226	1	20130913	6
34209568	20121031	16925285	19380102	2	20121031	4
34257540	20130122	17187899	19610402	2	20130122	4
34264341	20110623	15469556	19510502	1	20110623	4
34267293	20120115	16069732	19360417	1	20120115	4
34282252	20110307	15153998	19391104	1	20110307	5
34290636	20120424	16355544	19610322	2	20120424	5
34319465	20111101	15845591	19350518	1	20111101	6
34334004	20130519	17540952	19500615	1	20130519	4
34339441	20110518	15367367	19470102	1	20110518	4
34355210	20111117	15898065	19690725	2	20111117	4
34381641	20121210	17057219	19610329	1	20121210	5
34385803	20120907	16770641	19601203	2	20120907	4
34399343	20120513	16417652	19610225	2	20120513	5
34403231	20131118	18121884	19380722	1	20131118	5
34410407	20120521	16440720	19380731	1	20120521	4
34411842	20130918	17928964	19470701	1	20130918	4
34426738	20130206	17234289	19530309	1	20130206	6
34451917	20120804	16664577	19320503	1	20120804	4
34479215	20121226	17107606	19260529	1	20121226	5
34485013	20110505	15329060	19501115	1	20110505	5
34493044	20130822	17841209	19371021	2	20130822	6
34508435	20130103	17126808	19381225	2	20130103	6
34536679	20120216	16158556	19300416	1	20120216	6

34605113	20111231	16022086	19571109	2	20111231	5
34611126	20110420	15284030	19600730	1	20110420	4
34656605	20121025	16912138	19510908	1	20121025	6
34660929	20131009	17996649	19340102	1	20131009	4
34666416	20111022	15819822	19580411	1	20111022	5
34669404	20110519	15370429	19500106	2	20110519	4
34720791	20120430	16371746	19791212	2	20120430	4
34741087	20110331	15221800	19370709	2	20110331	4
34774351	20110205	15058911	19700530	2	20110205	4
34774748	20121215	17076851	19490424	1	20121215	6
34803333	20110228	15128358	19600119	2	20110228	4
34827300	20120419	16347109	19320102	1	20120419	4
34904148	20130114	17164055	19400602	1	20130114	4
34904875	20110613	15439144	19491216	1	20110613	5
34916080	20121128	17016246	19560515	1	20121128	5
34919034	20131223	18230852	19520310	1	20131223	5
34923734	20111011	15787183	19631130	1	20111011	5
34925854	20110614	15441735	19361126	1	20110614	4
34964835	20111114	15886976	19480520	2	20111114	5
35074523	20120627	16548505	19340721	2	20120627	6
35079255	20130324	17365812	19560628	1	20130324	6
35124017	20110508	15335168	19441019	1	20110508	4
35150835	20130328	17378045	19561213	1	20130328	4
35153210	20110621	15462892	19350219	2	20110621	5
35173901	20111105	15862107	19350622	1	20111105	5
35205459	20120325	16268875	19600217	1	20120325	6
35213877	20110530	15395117	19520828	1	20110530	5
35214621	20110515	15356699	19530920	1	20110515	5
35218463	20120625	16541992	19290608	2	20120625	5
35227884	20121216	17077282	19390508	2	20121216	5
35238143	20110812	15620100	19561223	1	20110812	4
35253862	20110912	15702604	19441101	1	20110912	4
35257886	20110516	15360300	19330508	1	20110516	5
35264052	20120411	16322622	19550530	1	20120411	5
35320968	20110621	15462932	19490216	1	20110621	5
35379885	20130127	17199995	19520330	2	20130127	6
35386528	20111101	15846045	19630127	1	20111101	5
35387554	20131224	18235074	19560831	2	20131224	4
35395096	20110417	15272817	19390409	1	20110417	4
35400227	20110809	15608364	19661213	2	20110809	5
35429391	20110607	15417643	19320923	2	20110607	5
35430252	20130131	17211603	19271212	2	20130131	5
35433568	20130909	17897486	19520916	2	20130909	6
35448670	20110607	15418840	19401223	2	20110607	5
35449548	20110613	15437896	19530324	2	20110613	4
35451402	20111023	15820467	19740329	2	20111023	6
35455108	20110220	15106449	19481120	2	20110220	5
35465964	20110218	15104278	19441130	1	20110218	4
35472210	20110428	15305244	19430605	1	20110428	4
35480069	20130619	17636617	19510906	2	20130619	6
35491986	20110905	15683745	19370111	2	20110905	6
35499673	20110520	15373483	19380707	2	20110520	6
35510884	20110816	15629815	19570815	2	20110816	4
35511365	20121225	17104033	19661005	2	20121225	4
35511934	20121205	17040353	19770610	2	20121205	6

35517067	20130309	17323778	19770126	1	20130309	6
35526375	20130619	17637343	19461203	2	20130619	5
35527425	20110516	15359660	19430816	1	20110516	6
35536391	20111226	16008508	19510415	2	20111226	4
35536835	20111222	16001038	19770826	1	20111222	4
35561912	20110524	15381350	19521026	1	20110524	4
35609202	20120105	16041258	19340112	2	20120105	6
35649151	20131120	18128938	19490820	1	20131120	4
35650023	20110327	15210454	19490901	1	20110327	4
35652676	20110216	15096277	19441117	2	20110216	5
35658345	20130804	17780951	19300226	1	20130804	5
35666252	20120223	16177102	19531201	1	20120223	6
35706399	20130103	17126450	19410910	1	20130103	4
35711150	20131008	17990613	19510901	2	20131008	6
35720322	20120626	16544068	19351010	1	20120626	5
35724095	20120502	16382485	19651013	2	20120502	4
35766893	20111206	15951675	19440129	2	20111206	6
35766939	20120710	16592745	19340301	2	20120710	5
35774186	20130709	17703082	19541216	2	20130709	6
35780133	20110501	15309011	19480830	2	20110501	6
35787292	20110312	15172033	19441005	1	20110312	6
35797967	20120101	16022738	19540208	2	20120101	6
35823484	20120201	16107843	19630629	1	20120201	5
35843404	20110908	15696389	19650606	1	20110908	4
35843835	20110314	15175830	19681031	2	20110314	4
35843880	20120605	16483629	19350209	1	20120605	6
35843937	20131212	18202254	19441111	2	20131212	4
35875264	20110905	15682701	19590730	1	20110905	5
35884287	20110406	15238860	19510903	2	20110406	6
35896505	20110328	15213470	19510415	1	20110328	6
35940251	20110306	15150155	19640711	2	20110306	4
35948506	20111120	15903466	19691107	1	20111120	4
35949361	20120806	16670271	19400709	1	20120806	4
35951883	20130410	17421254	19630727	1	20130410	6
35952820	20120630	16556075	19491206	2	20120630	4
35957701	20121223	17097630	19591202	1	20121223	6
35959810	20121113	16971915	19471110	1	20121113	5
35988819	20121227	17110702	19771206	2	20121227	4
36013599	20111201	15933557	19570409	2	20111201	6
36013862	20130312	17331633	19530401	1	20130312	5
36023833	20110827	15658639	19700402	2	20110827	5
36038207	20130923	17938181	19580121	2	20130923	6
36039255	20120321	16260824	19411204	1	20120321	6
36042850	20130102	17122632	19491012	1	20130102	4
36061924	20111214	15977058	19531211	1	20111214	6
36070538	20120130	16097941	19521010	2	20120130	4
36072692	20110524	15381629	19540609	1	20110524	5
36073866	20130618	17633722	19760227	2	20130618	6
36079251	20111106	15862548	19580824	1	20111106	4
36113129	20120429	16369719	19420610	2	20120429	6
36118146	20110818	15636197	19690901	1	20110818	5
36130684	20120303	16203649	19641010	2	20120303	6
36132317	20130713	17715546	19650127	1	20130713	5
36150488	20121031	16923839	19651120	1	20121031	5
36158948	20110327	15210391	19411001	2	20110327	5

36161087	20110906	15688266	19481215	1	20110906	4
36169989	20120615	16518598	19220117	1	20120615	5
36195401	20110515	15356844	19501025	2	20110515	4
3620194220130109		17148640	19360122	1	20130109	4
36209742	20110306	15150192	19640218	1	20110306	4
3621496720130807		17794532	19731224	2	20130807	5
36218163	20120106	16044278	19651129	1	20120106	5
36238310	20111011	15786414	19550101	1	20111011	4
36238854	20120711	16597109	19600614	1	20120711	6
36253335	20120113	16066937	19571027	1	20120113	6
36266418	20111013	15793421	19600220	1	20111013	6
36275555	20110918	15719438	19400430	1	20110918	6
36276570	20110309	15162136	19400831	2	20110309	6
36294549	20120517	16431132	19900101	1	20120517	4
3629896120130109		17147867	19820703	1	20130109	6
36312308	20110512	15351572	19500415	2	20110512	4
36325947	20110216	15097240	19840305	1	20110216	6
36327034	20110330	15218395	19470114	2	20110330	4
36335929	20110518	15367423	19650817	1	20110518	4
36361645	20110530	15395126	19360615	1	20110530	5
36381358	20120111	16058799	19510522	1	20120111	5
36387754	20110416	15272238	19350305	2	20110416	5
3639847720130925		17945550	19381016	1	20130925	4
3641061420130419		17451048	19490106	2	20130419	5
36419133	20121119	16987532	19640328	1	20121119	6
36429273	20111201	15936407	19560913	1	20111201	4
36449475	20110827	15658384	19600501	2	20110827	6
36454725	20110117	15018581	19621023	1	20110117	6
36456801	20110217	15100688	19590207	1	20110217	4
36473322	20110214	15089025	19501025	2	20110214	6
3649691020130908		17893950	19440901	2	20130908	6
36513903	20110428	15304573	19961118	1	20110428	6
36524455	20110503	15320108	19830603	2	20110503	4
36541272	20111109	15874591	19560205	1	20111109	5
36554446	20120130	16097884	19641123	1	20120130	5
36560380	20120321	16260640	19540907	2	20120321	4
3656470220130904		17880739	19480301	1	20130904	6
36571650	20110913	15705889	19230809	1	20110913	6
36579201	20120521	16440673	19521016	1	20120521	5
3659722520130503		17493450	19490125	1	20130503	4
3660161320130731		17767281	19311116	1	20130731	5
3661180020131201		18156784	19671225	2	20131201	5
36615200	20120702	16562960	19770509	2	20120702	4
36622103	20120108	16047676	19540910	2	20120108	5
36623479	20111129	15927771	19541110	2	20111129	5
36630361	20111231	16021374	19350525	1	20111231	4
36637624	20121006	16857948	19331108	1	20121006	4
36643944	20120817	16708549	19700630	2	20120817	5
3665568220130215		17247707	19580214	2	20130215	5
3665942420131118		18122194	19550120	2	20131118	6
36669644	20120516	16427315	19680425	1	20120516	6
36671531	20120225	16182276	19520226	1	20120225	6
36681911	20120620	16530245	19531113	2	20120620	4
36690172	20110222	15114053	19430705	2	20110222	4
36692587	20110403	15229011	19620215	2	20110403	5

36724615	20110927	15744449	19701027	2	20110927	5
36731450	20111031	15840030	19740304	2	20111031	5
36739272	20121025	16912672	19470913	1	20121025	5
36746551	20110531	15397475	19531014	1	20110531	6
36753885	20110316	15182781	19690621	1	20110316	4
36765250	20110704	15498570	19400411	1	20110704	6
36766515	20130617	17628288	19640412	1	20130617	5
36767381	20110316	15184182	19400123	1	20110316	5
36771456	20110221	15110287	19480726	2	20110221	4
36773838	20110314	15176212	19270625	1	20110314	5
36780764	20111111	15880662	19581023	1	20111111	6
36780979	20120419	16346335	19311017	1	20120419	4
36781745	20110110	14997753	19541103	2	20110110	6
36805659	20111123	15913108	19600529	2	20111123	5
36808330	20110214	15088078	19710109	2	20110214	6
36815040	20110314	15176854	19510307	2	20110314	5
36830236	20110518	15367148	19561002	2	20110518	4
36839255	20110502	15313292	19620219	1	20110502	4
36874518	20130604	17588568	19680608	1	20130604	6
36880474	20110628	15479409	19640310	1	20110628	6
36880985	20110328	15213565	19400406	1	20110328	5
36887635	20110422	15290443	19551022	1	20110422	4
36896534	20121108	16956761	19291115	1	20121108	6
36898267	20130429	17472700	19710218	1	20130429	5
36912751	20120530	16462681	19701029	1	20120530	6
36929518	20130618	17632661	19720326	1	20130618	6
36935587	20120826	16729710	19460317	2	20120826	5
36941647	20110501	15309255	19560201	1	20110501	4
36952768	20120808	16679349	19560730	2	20120808	5
36962466	20130128	17203398	19600517	2	20130128	6
36964382	20120112	16063498	19390711	1	20120112	4
36974466	20110621	15462350	19641110	1	20110621	5
36975754	20130121	17183497	19461030	1	20130121	5
36980720	20111007	15780076	19280206	1	20111007	5
36983729	20120724	16634165	19311007	2	20120724	5
36984733	20110424	15292257	20010926	2	20110424	5
37012627	20111124	15917012	19641104	2	20111124	5
37013868	20121003	16847310	19510114	2	20121003	6
37018352	20130807	17795907	19520827	1	20130807	4
37030298	20110601	15403673	19550406	1	20110601	4
37036752	20111029	15837115	19570524	2	20111029	5
37041422	20130421	17453245	19681006	2	20130421	6
37066516	20110906	15688050	19760915	2	20110906	6
37073986	20120130	16097058	19490413	1	20120130	6
37076703	20110314	15176545	19520515	2	20110314	5
37083957	20111211	15966465	19370112	2	20111211	5
37095377	20130428	17471724	19640903	1	20130428	5
37106460	20110624	15472246	19531212	2	20110624	4
37110717	20110410	15250151	19720907	1	20110410	4
37119770	20121103	16937979	19290121	2	20121103	6
37147118	20120301	16195627	19630710	2	20120301	6
37159787	20130724	17747897	19580719	1	20130724	4
37161403	20120217	16161802	19710108	1	20120217	5
37172886	20110402	15228294	19720925	2	20110402	5
37175205	20130310	17324047	19390101	1	20130310	6

37182506	20130129	17206161	19600218	2	20130129	5
37187103	20130215	17248615	19680706	1	20130215	6
37202645	20120304	16204236	19470426	1	20120304	4
37212514	20130123	17191603	19360301	1	20130123	4
37213131	20110620	15457164	19690519	1	20110620	5
37225062	20110914	15710620	19580610	1	20110914	5
37241488	20120708	16584295	19620618	2	20120708	4
37249824	20110427	15302119	19970116	2	20110427	4
37256487	20120701	16557015	19461020	1	20120701	4
37264678	20130926	17949413	19570302	2	20130926	6
37269355	20130807	17793917	19481201	2	20130807	5
37271106	20110529	15392247	19901018	1	20110529	4
37273362	20120926	16824401	19440701	2	20120926	6
37274718	20110221	15110023	20091219	1	20110221	5
37274796	20110314	15176943	19431102	2	20110314	5
37291728	20110515	15356456	19331220	1	20110515	5
37298412	20110817	15633305	19500301	2	20110817	6
37314253	20130803	17780246	19750228	2	20130803	6
37314457	20110417	15272570	19551020	1	20110417	4
37317785	20110416	15272218	19441103	1	20110416	5
37320619	20111231	16021314	19381014	1	20111231	6
37323992	20120112	16063727	19480315	2	20120112	5
37324202	20110604	15413636	19431029	1	20110604	5
37324917	20110215	15092806	19510613	2	20110215	6
37328282	20110615	15447109	19910901	1	20110615	4
37332095	20111014	15795368	19430104	2	20111014	6
37339256	20110226	15125498	19871128	1	20110226	5
37348882	20130108	17145373	19470314	2	20130108	5
37355207	20110519	15370587	19660311	2	20110519	5
37358615	20111023	15820493	19581126	2	20111023	5
37364048	20111002	15756520	19700208	1	20111002	6
37364651	20130422	17456948	19550411	1	20130422	5
37374304	20110815	15626129	19440527	2	20110815	6
37374746	20130526	17560339	19640803	2	20130526	6
37381536	20120203	16116530	19380130	2	20120203	4
37385094	20110823	15648011	19510404	1	20110823	4
37396524	20120530	16461841	19440103	1	20120530	4
37397572	20130714	17716088	19470820	1	20130714	5
37403911	20110615	15445269	19670421	1	20110615	4
37407435	20110518	15367374	19580128	2	20110518	6
37419286	20121228	17112480	19571222	1	20121228	5
37425562	20130925	17944053	19680615	1	20130925	4
37431677	20110618	15455220	19410905	2	20110618	4
37435726	20110411	15253677	19351212	1	20110411	6
37439911	20110831	15666751	19551220	1	20110831	4
37439933	20110323	15202684	19530923	1	20110323	5
37459908	20110302	15139356	19650608	1	20110302	5
37461204	20131225	18237596	19490417	2	20131225	5
37465002	20120524	16449203	19510615	2	20120524	6
37470181	20120910	16776936	19370225	1	20120910	6
37475255	20110418	15276705	19421111	2	20110418	6
37476269	20110320	15192559	19460728	1	20110320	5
37481837	20110405	15233791	19430221	2	20110405	6
37483764	20111126	15921387	19520812	2	20111126	5
37487551	20120907	16770279	19811025	2	20120907	6

37489284	20110923	15736460	19600226	2	20110923	4
37500860	20110913	15704001	19360806	1	20110913	5
37502106	20110304	15147788	19290312	2	20110304	6
37504942	20110830	15662741	19370803	1	20110830	5
37505241	20120626	16545623	19531108	2	20120626	6
37506302	20110519	15370681	19660715	1	20110519	4
37508466	20110413	15262793	19510926	1	20110413	4
37511323	20131212	18201137	19490612	1	20131212	5
37512235	20110422	15290539	19720118	1	20110422	5
37536064	20111102	15849655	19921029	2	20111102	4
37546604	20130219	17259822	19540812	1	20130219	6
37547447	20130530	17572075	19380102	2	20130530	5
37547538	20111010	15783009	19661115	2	20111010	4
37549874	20110508	15335236	19450302	2	20110508	4
37557634	20110618	15455216	19530321	2	20110618	4
37559630	20110416	15272340	19591206	1	20110416	4
37560819	20120920	16808440	19380528	1	20120920	5
37568368	20110604	15413842	19591025	2	20110604	5
37574575	20110820	15640741	19470121	2	20110820	6
37583689	20110222	15112421	19370901	2	20110222	6
37587590	20110527	15390487	19881118	1	20110527	4
37590151	20131229	18245997	19571007	1	20131229	5
37596433	20130104	17131647	19360308	2	20130104	4
37636503	20120721	16626243	19550514	1	20120721	4
37660336	20120306	16214433	19441208	2	20120306	5
37660756	20120304	16204098	19950407	1	20120304	6
37671399	20130411	17425228	19291202	1	20130411	6
37684518	20130816	17826168	19640417	2	20130816	5
37698478	20130915	17917273	19640918	1	20130915	4
37698741	20110407	15243561	19471225	2	20110407	4
37705605	20111229	16018403	19280204	1	20111229	5
37720584	20130228	17288060	19660509	2	20130228	5
37728000	20130308	17321660	19440211	1	20130308	4
37745883	20110510	15343894	19490404	2	20110510	4
37747823	20120306	16213567	19630502	1	20120306	4
37754066	20120213	16145224	19680229	1	20120213	4
37769463	20130424	17462798	19520220	1	20130424	5
37780384	20110427	15299341	19410507	2	20110427	6
37780497	20110219	15105683	19670430	1	20110219	4
37792431	20110317	15187428	19360923	1	20110317	5
37793241	20120919	16805554	19680801	2	20120919	6
37793592	20110415	15267816	19511027	2	20110415	4
37795690	20111219	15990416	19511116	1	20111219	4
37800387	20120620	16530595	19630206	1	20120620	6
37802065	20121023	16906015	19631220	1	20121023	5
37818567	20110701	15489935	19510222	2	20110701	5
37828505	20130413	17431289	19450611	1	20130413	4
37839955	20110518	15367221	19550205	1	20110518	6
37843826	20110728	15570292	19631213	1	20110728	4
37851062	20110321	15195035	19580310	2	20110321	6
37853557	20110204	15058817	19291215	2	20110204	6
37854889	20120105	16040625	19520828	1	20120105	4
37857300	20110819	15639019	19590705	1	20110819	5
37857775	20110304	15147729	19750708	2	20110304	5
37858234	20110303	15143961	19380614	1	20110303	4

37858789	20110424	15292460	20091028	2	20110424	5
37865900	20120524	16449622	19530710	2	20120524	6
37872745	20120505	16394536	19430606	1	20120505	5
37874070	20120627	16547868	19591228	1	20120627	5
37881882	20111209	15964404	19470411	2	20111209	4
37888952	20110531	15397308	19650916	2	20110531	6
37892765	20110511	15347847	19440502	1	20110511	5
37893417	20110421	15286775	19730512	2	20110421	5
3789350820130819		17833058	19491204	1	20130819	6
3790092820121231		17116689	19520728	1	20121231	5
37903643	20110524	15382228	19580630	1	20110524	5
37905638	20111218	15987154	19281113	1	20111218	6
37911903	20121101	16930889	19631020	1	20121101	5
37926684	20111107	15866223	19590520	1	20111107	6
3793248220130408		17409931	20071009	1	20130408	6
37932993	20110304	15147826	19860320	1	20110304	5
37936699	20120201	16107649	19421128	2	20120201	6
37940548	20111207	15955867	19370215	1	20111207	6
37941392	20111211	15966454	19450708	1	20111211	6
3794210220131104		18069430	19361005	1	20131104	6
37943810	20110425	15293754	19810917	1	20110425	6
37944653	20110319	15192088	19660512	1	20110319	6
37948235	20110217	15100608	19370706	1	20110217	5
3795720320130510		17516043	19660528	2	20130510	4
37966760	20110504	15324690	19590602	2	20110504	4
3796699720130714		17716063	19620212	1	20130714	5
3796707020130101		17117537	19650608	1	20130101	4
37973425	20120718	16618021	19520902	1	20120718	5
37977030	20110907	15692170	19560410	1	20110907	6
3797724520130708		17696943	19321101	1	20130708	5
37977530	20110320	15192419	19330928	2	20110320	5
3798083920130717		17726309	19370627	1	20130717	5
37983269	20110327	15210242	19620223	1	20110327	4
37991654	20110728	15570844	19551220	2	20110728	6
37995112	20110303	15143654	19720903	1	20110303	5
37995270	20110208	15067991	19620908	1	20110208	5
37999716	20110315	15179596	19580821	1	20110315	5
38001353	20110508	15335127	19560310	1	20110508	5
3800256120130326		17373201	19850128	1	20130326	5
38008058	20120314	16238397	19610520	1	20120314	4
38012270	20110310	15167081	19770623	1	20110310	4
38020778	20110412	15259101	19270821	2	20110412	6
38022592	20110612	15434570	19530606	2	20110612	6
38024496	20110618	15455192	19630924	1	20110618	5
38032698	20110207	15062935	19681010	2	20110207	5
3803673620121223		17097389	19360620	2	20121223	5
38046887	20110228	15127299	19300331	1	20110228	4
38047153	20120503	16387696	19530120	2	20120503	4
38048134	20110307	15153980	19940417	2	20110307	6
38051897	20110530	15393044	19521205	2	20110530	4
38053257	20110315	15180242	19500130	2	20110315	6
38053826	20110503	15318304	19570606	2	20110503	5
38054385	20111025	15827168	19690217	1	20111025	4
38062327	20110608	15422948	19240223	1	20110608	6
38062418	20110222	15113955	19610618	1	20110222	5

38067526	20110218	15103616	19360518	2	20110218	5
38071226	20110501	15309376	19570220	1	20110501	5
38071328	20110314	15176462	19591205	2	20110314	4
38077519	20110405	15233069	19691014	2	20110405	6
38077597	20110610	15430497	19730213	2	20110610	4
38077713	20110310	15166296	19580518	2	20110310	5
38082392	20110327	15210369	19590207	2	20110327	6
38087353	20110409	15249260	19600512	2	20110409	4
38093888	20110124	15036922	19821004	2	20110124	4
38118079	20110721	15552794	19571023	2	20110721	6
38120513	20110410	15249773	19340812	2	20110410	4
38121903	20120515	16423931	19750615	1	20120515	5
38123114	20111109	15873478	19470818	2	20111109	5
38123545	20110521	15375080	19570607	2	20110521	6
38129883	20110408	15246391	19441127	1	20110408	4
38130255	20110331	15221739	19390508	1	20110331	4
38131827	20110427	15302025	20090818	1	20110427	6
38131850	20110415	15270638	19660326	1	20110415	6
38132488	20120506	16395059	19500507	2	20120506	4
3813297720130730		17764259	19300521	1	20130730	5
38133572	20110508	15334972	19520515	2	20110508	4
38135374	20110329	15215591	19780510	1	20110329	5
38135589	20110419	15279965	19540319	2	20110419	5
38136673	20110612	15434415	19540628	1	20110612	6
38138839	20110725	15560114	19351015	2	20110725	6
38139763	20120506	16394809	19550128	1	20120506	4
38146520	20111222	16001008	19651015	1	20111222	5
38147090	20110327	15210382	19600101	2	20110327	6
38148297	20110407	15243365	19760122	1	20110407	4
38157765	20111107	15865217	19491205	1	20111107	5
38160860	20110929	15750733	19501010	2	20110929	6
38162399	20110525	15385178	19650906	1	20110525	4
38165105	20110424	15292424	19500327	2	20110424	5
38165309	20110425	15296091	19671126	1	20110425	6
38168693	20110518	15367381	19680508	1	20110518	5
38171663	20110330	15218905	19561201	1	20110330	5
38174651	20110308	15158106	19450808	1	20110308	6
38177003	20110410	15249827	19380815	2	20110410	6
38179485	20111213	15973825	19510724	2	20111213	5
38183889	20110726	15563556	19440925	1	20110726	6
38192915	20110503	15319893	19770815	2	20110503	4
3819394120130911		17907300	19390128	1	20130911	5
38197330	20110510	15343671	19490226	2	20110510	5
38198140	20110417	15272935	19320229	1	20110417	6
38202607	20111128	15925256	19560601	1	20111128	5
3820741920130814		17817583	19690107	1	20130814	4
38209391	20110619	15455654	19590818	1	20110619	6
38212098	20110417	15272945	19700126	1	20110417	5
38212667	20110518	15365825	19590713	2	20110518	5
38212736	20110716	15538605	19611211	1	20110716	6
38223573	20111022	15820003	19410920	2	20111022	4
38224338	20120514	16421616	19580410	2	20120514	5
38230727	20110602	15405602	19470502	1	20110602	5
38234683	20110509	15337664	19680516	2	20110509	5
38241291	20110902	15676356	19390712	1	20110902	4

38250189	20110506	15332802	19570825	1	20110506	4
38250292	20110316	15183960	19420121	1	20110316	6
38258070	20110509	15339895	19620721	2	20110509	5
38258478	20111113	15883320	19530722	1	20111113	6
38260412	20110531	15396888	19620724	1	20110531	4
38261664	20110520	15373589	19620312	1	20110520	4
38265031	20110901	15670315	19291005	1	20110901	5
38267173	20111018	15806994	19530105	2	20111018	4
38269146	20110606	15414870	19900308	2	20110606	4
38273482	20110814	15622109	19620821	2	20110814	4
38282405	20110528	15391754	19490929	2	20110528	5
38287353	20110624	15471868	19580905	2	20110624	5
38288903	20110627	15476783	19490301	2	20110627	6
38290618	20110726	15564400	19540607	2	20110726	6
38291984	20110411	15253920	19640102	1	20110411	5
38294756	20110621	15461999	19501210	1	20110621	4
38298236	20110628	15480337	19460925	2	20110628	5
38299433	20110610	15432284	19600408	2	20110610	5
38308962	20120312	16233100	19710427	2	20120312	4
38311852	20110614	15439693	19550827	1	20110614	6
38318808	20110821	15641173	19450227	1	20110821	6
3831943620130728		17757837	19500820	1	20130728	4
38325303	20110621	15462554	19590502	2	20110621	6
38327150	20111015	15800381	19601231	2	20111015	4
38345072	20120711	16594914	19720217	1	20120711	5
38358940	20110625	15473295	19630622	2	20110625	4
38360188	20110608	15421902	19590101	1	20110608	6
38361045	20110811	15616774	19530406	2	20110811	6
3836110320130321		17360652	19830216	2	20130321	6
38368693	20111226	16008117	19531025	1	20111226	4
38376408	20111123	15913878	19650913	1	20111123	5
3837663520130317		17345700	19450718	1	20130317	4
38377478	20110616	15450218	19620502	1	20110616	5
38382706	20110623	15469432	19510503	1	20110623	5
38383550	20111129	15927322	19261028	2	20111129	5
38384122	20110905	15683217	19520102	2	20110905	5
38396995	20111002	15756640	19460625	2	20111002	4
38398355	20111013	15795084	19570623	1	20111013	4
38399427	20110821	15641313	19590212	1	20110821	5
38401693	20110810	15612861	19680606	2	20110810	4
38403757	20120229	16190325	19611224	1	20120229	4
38405173	20110513	15354550	19351028	2	20110513	6
38415780	20110901	15671224	19711122	1	20110901	5
38416067	20110727	15567479	19541010	1	20110727	4
38419975	20120116	16070356	19800407	2	20120116	6
3843575520130513		17523739	19221112	2	20130513	5
38436736	20110728	15570281	19580502	1	20110728	4
38436952	20110926	15740972	19570304	1	20110926	6
38453031	20111213	15973266	19350623	1	20111213	4
38458172	20110914	15710485	19450217	1	20110914	4
38460321	20110802	15585816	19610820	1	20110802	4
38466976	20110830	15664203	19470217	1	20110830	4
38470370	20110731	15575193	19650501	1	20110731	6
38471908	20111024	15823435	19681202	1	20111024	6
38479037	20110729	15573349	19710713	1	20110729	5

38483919	20110903	15678459	19960926	2	20110903	6
38485993	20110620	15459789	19590520	1	20110620	5
38486816	20111104	15857252	19830218	1	20111104	5
38487115	20111221	15998013	19500616	2	20111221	6
38487217	20110929	15750805	19481205	2	20110929	6
38490969	20111123	15913861	19690103	2	20111123	4
38493695	20110806	15600365	19320205	1	20110806	4
38502328	20110811	15616856	19771125	1	20110811	4
38503467	20110816	15628390	19400204	1	20110816	5
38504595	20111012	15790581	19300515	1	20111012	6
38508951	20110916	15714062	19661031	1	20110916	4
38509136	20120514	16421164	19500302	2	20120514	5
38518728	20110902	15675852	19650903	1	20110902	6
38520273	20110825	15654378	19690101	1	20110825	6
38520295	20131201	18156665	19491129	2	20131201	6
38523103	20120211	16142899	19560702	1	20120211	5
38529123	20110809	15607222	19441101	1	20110809	4
38529349	20130107	17140392	19580624	1	20130107	6
38532999	20120326	16271988	19620213	1	20120326	4
38541376	20131225	18236505	19501203	1	20131225	5
38547590	20111107	15866610	19550320	2	20111107	4
38551096	20120229	16189412	19481129	1	20120229	6
38553309	20110908	15695901	19581224	2	20110908	5
38559045	20110925	15738356	19601210	1	20110925	4
38560666	20110914	15709405	19601201	1	20110914	5
38561170	20110827	15658494	19290915	1	20110827	6
38563267	20110913	15706613	19611126	1	20110913	5
38567350	20110911	15701946	19321010	1	20110911	5
38570513	20111119	15902571	19500926	1	20111119	5
38574617	20110910	15701741	19660722	1	20110910	6
38574855	20111030	15838079	19670625	2	20111030	4
38576248	20110913	15706406	19570318	1	20110913	4
38576293	20110825	15653586	19940410	1	20110825	5
38577036	20110911	15701900	19331204	1	20110911	6
38582557	20110920	15725708	19511001	1	20110920	5
38586173	20111011	15786381	19570101	1	20111011	5
38586559	20111215	15981761	19541105	2	20111215	5
38588657	20111115	15891059	19391210	1	20111115	5
38594126	20110924	15737171	19520910	2	20110924	4
38595630	20130107	17138767	19460810	2	20130107	5
38596622	20111005	15770689	19580711	1	20111005	5
38598537	20111007	15780096	19700107	2	20111007	4
38604261	20110930	15752279	19390914	2	20110930	4
38608172	20120923	16814518	19610503	2	20120923	5
38609517	20130403	17399781	19430825	1	20130403	5
38609697	20130319	17351783	19280208	2	20130319	5
38617902	20111011	15787169	19390408	2	20111011	6
38618132	20111230	16020010	19270405	1	20111230	5
38621237	20120111	16059834	19270824	1	20120111	4
38625046	20110907	15692398	19350312	1	20110907	5
38625842	20120909	16773053	19330125	2	20120909	4
38626583	20120606	16488579	19781029	1	20120606	5
38626743	20120219	16164275	19621215	2	20120219	6
38628669	20120314	16240184	19850411	1	20120314	5
38628987	20110921	15730430	19710412	1	20110921	5

38632847	20110830	15662379	19511110	1	20110830	5
38633260	20120220	16168236	19500225	1	20120220	6
38633782	20120227	16183613	19540627	1	20120227	6
38633986	20120104	16037201	19410105	2	20120104	4
38641702	20120205	16119732	19410108	2	20120205	5
38641906	20110912	15702612	19340804	1	20110912	5
38651604	20120229	16190462	19650611	1	20120229	5
38651671	20111010	15783036	19680626	2	20111010	4
38654772	20120526	16454243	19320804	2	20120526	6
38656267	20111226	16009202	20000117	1	20111226	4
38659186	20110927	15744403	19500416	2	20110927	5
38660332	20120420	16349674	19620714	2	20120420	4
38660605	20120808	16678795	19360810	2	20120808	5
3866098920130508		17506496	19630912	2	20130508	6
38661084	20120101	16022433	19291122	1	20120101	5
38661551	20120305	16208182	19420612	1	20120305	4
38662816	20120906	16766826	19331006	1	20120906	4
38662861	20120108	16047621	19440123	1	20120108	4
38662918	20111113	15883309	19481125	2	20111113	5
38663751	20111214	15977466	19630610	2	20111214	5
3866477620130609		17605507	19750221	2	20130609	6
38665451	20111102	15851792	19550801	2	20111102	4
3866613620131108		18092207	19590820	2	20131108	6
38666432	20110912	15702606	19230806	1	20110912	6
38667106	20120525	16452859	19680702	1	20120525	6
38668881	20120226	16182678	19550624	1	20120226	5
38669986	20111129	15928235	19460615	1	20111129	5
38674178	20120211	16142826	19610220	1	20120211	5
38679640	20111106	15862517	19410104	1	20111106	6
38681037	20111015	15800350	19490128	1	20111015	6
38683817	20121024	16909014	19290226	1	20121024	5
38688516	20120403	16297079	19440630	1	20120403	6
3869266920130108		17144369	19570224	1	20130108	6
3869311720130904		17877718	19550320	2	20130904	5
38698510	20111128	15925260	19211029	2	20111128	4
38701427	20111010	15782610	19560412	1	20111010	4
38703605	20120423	16353798	19321107	1	20120423	4
3870515620130503		17493675	19360510	1	20130503	6
38710086	20111018	15807889	19520301	2	20111018	5
38710666	20121021	16899232	19490926	1	20121021	5
38717872	20120630	16556403	19431215	1	20120630	5
38725278	20111116	15894383	19520825	2	20111116	6
38728540	20120902	16746822	19770903	1	20120902	5
3873231920130915		17917349	19571219	2	20130915	4
38732900	20111021	15817811	19650517	2	20111021	5
38735330	20111114	15887334	19360410	1	20111114	4
38738419	20111114	15886812	19480215	1	20111114	4
38755372	20120110	16056091	19560517	1	20120110	5
38756353	20120107	16046894	19481015	2	20120107	4
38757276	20111111	15881063	19761126	1	20111111	6
3875785620121226		17107239	19320121	2	20121226	6
38758326	20111216	15984945	19391212	2	20111216	6
38759716	20120525	16452888	19211213	1	20120525	5
38760064	20111128	15925124	19441222	1	20111128	6
3876131820130729		17760496	19560329	1	20130729	6

38763483	20111214	15978148	19940708	2	20111214	5
38769107	20120309	16227222	19491206	1	20120309	4
38769209	20120308	16222484	19240909	2	20120308	4
38771970	20120222	16174624	19750505	1	20120222	5
3877509620130923		17938088	19331205	2	20130923	6
38780197	20111125	15919527	19681220	2	20111125	5
38780324	20120509	16407541	19400714	2	20120509	6
38782808	20120603	16474351	19730313	1	20120603	6
38783210	20111107	15864975	19590110	1	20111107	4
38784097	20111121	15906392	19560320	2	20111121	4
38788577	20120125	16089881	19600728	1	20120125	4
38790646	20111114	15886770	19541210	2	20111114	4
38801615	20120209	16136529	19540227	2	20120209	6
38802469	20120208	16132958	19620202	1	20120208	4
3880562820130613		17619493	19641228	2	20130613	6
38808796	20120112	16063840	19470908	1	20120112	5
38810116	20120501	16379174	19340210	1	20120501	6
38811379	20120129	16094594	19751222	2	20120129	5
38818165	20120302	16201366	19470702	1	20120302	4
38818687	20120203	16116420	19581025	1	20120203	5
38821351	20120530	16462832	19671125	2	20120530	4
3882694720130731		17764689	19420529	1	20130731	4
38829355	20120228	16187553	19431217	1	20120228	6
38833453	20120224	16180822	19590614	1	20120224	6
38836009	20120102	16026305	19680801	1	20120102	6
38838072	20120330	16283699	19410113	1	20120330	5
3884057220130108		17144962	19480527	2	20130108	5
38841826	20120326	16271630	19521110	1	20120326	4
38849853	20111226	16008261	19651216	1	20111226	5
38854012	20111225	16005946	19570205	1	20111225	5
38862394	20120225	16182327	19720911	1	20120225	5
38866090	20111219	15990897	19590811	2	20111219	5
38872025	20120103	16032704	19640606	1	20120103	6
38875342	20111204	15943377	19370622	2	20111204	5
38875751	20111223	16003890	19770802	1	20111223	5
38876083	20120304	16204001	19461111	1	20120304	6
38883408	20120203	16116706	19700318	2	20120203	6
38885551	20111218	15987219	19500331	2	20111218	5
38890367	20120208	16131893	19461209	1	20120208	4
38891053	20120310	16229234	19430413	1	20120310	6
38893913	20120321	16259617	19631125	1	20120321	4
38897653	20120708	16584292	19701122	1	20120708	5
38906160	20121111	16963521	19241212	2	20121111	6
38907185	20120520	16436853	19970826	1	20120520	4
3890791420130610		17610325	19261120	2	20130610	4
38908100	20120111	16060073	19801122	2	20120111	4
38911465	20120126	16090355	19290308	1	20120126	4
38916584	20120222	16173858	19290614	2	20120222	6
38919390	20120221	16170813	19550720	1	20120221	5
38925234	20120130	16097030	19570228	1	20120130	6
38926077	20120602	16474119	19811127	2	20120602	6
3892964520130104		17132551	19280215	2	20130104	6
38933878	20120220	16166602	19521005	2	20120220	4
38936800	20120127	16092319	19330815	1	20120127	6
38937972	20120204	16118634	19610908	1	20120204	4

38946633	20120318	16250536	19560520	2	20120318	5
38950639	20130626	17654839	19610528	1	20130626	6
38952113	20120321	16260766	19370626	2	20120321	5
38962480	20120311	16229474	19580808	2	20120311	5
38965570	20120207	16129297	19900724	2	20120207	5
38969243	20120817	16709260	19610513	1	20120817	4
38971049	20120322	16263890	19750502	1	20120322	4
38972188	20120219	16164452	19490423	2	20120219	5
38973852	20120318	16250734	19641222	2	20120318	5
38974173	20120402	16291772	19661217	2	20120402	4
38976168	20120313	16236765	19481126	1	20120313	5
38978197	20120420	16349591	19640306	2	20120420	5
38982762	20120306	16214391	19800202	2	20120306	6
38985056	20120727	16643663	19541201	2	20120727	6
38988191	20120306	16213138	19630330	1	20120306	6
38989478	20120831	16742016	19861102	1	20120831	5
39000210	20120314	16241318	19800731	1	20120314	5
39000823	20120513	16417677	19690212	2	20120513	6
39003366	20120706	16579936	19650115	1	20120706	4
39011273	20120301	16196516	19420120	2	20120301	4
39012787	20120330	16283853	19670217	2	20120330	4
39013053	20120801	16654403	19530727	1	20120801	4
39017102	20121113	16971760	19630202	2	20121113	6
39017533	20121010	16867976	19500427	1	20121010	5
39022407	20120713	16604135	19330825	2	20120713	4
39027946	20120418	16343738	19650529	1	20120418	4
39028472	20120330	16283164	19490321	1	20120330	4
39031873	20120324	16268233	19600518	2	20120324	6
39032821	20120613	16508458	19470102	1	20120613	4
39034338	20121207	17048195	19500901	2	20121207	4
39037019	20120508	16402292	19720502	1	20120508	5
39045233	20120417	16340665	19450616	1	20120417	4
39048072	20120304	16204063	20080409	2	20120304	5
39048630	20120301	16196546	19730911	1	20120301	6
39049133	20120404	16299697	20040817	1	20120404	6
39050629	20120318	16250436	19320904	1	20120318	4
39061648	20120411	16324387	19500130	2	20120411	4
39061911	20120613	16511536	19640103	1	20120613	6
39064578	20120927	16827492	19501207	1	20120927	4
39065695	20120528	16458008	19340901	2	20120528	5
39073160	20120628	16552061	19430829	1	20120628	5
39076385	20120426	16363910	19651031	2	20120426	4
39079715	20120418	16342399	19700617	1	20120418	6
39094321	20120419	16345735	19430107	1	20120419	5
39094525	20120612	16507400	20090626	1	20120612	4
39096010	20120418	16342937	19460801	2	20120418	4
39100748	20130909	17898726	19521120	2	20130909	5
39111085	20120417	16338983	19470228	1	20120417	4
39122708	20130729	17760822	19480306	2	20130729	6
39135041	20120502	16384282	19440718	1	20120502	6
39151332	20120516	16428862	19490103	2	20120516	5
39152982	20120723	16630477	19370217	1	20120723	5
39153123	20131111	18097992	19500520	2	20131111	6
39153383	20121104	16938463	19540326	1	20121104	6
39155630	20120827	16732489	19640126	2	20120827	6

39155834	20120514	16419993	19611031	2	20120514	4
39157250	20120529	16459420	19411001	1	20120529	5
39161789	20121229	17114695	19791001	2	20121229	4
39165850	20120522	16443564	19520921	1	20120522	6
39168020	20121026	16915197	19390605	1	20121026	4
39175503	20120809	16682552	19550522	1	20120809	5
39176755	20120515	16425338	19380501	2	20120515	4
39178693	20120430	16371591	19550415	1	20120430	4
39181516	20120531	16465474	19660321	1	20120531	5
39181776	20120626	16545143	19410506	1	20120626	5
39183830	20120517	16430836	19490103	1	20120517	6
39185676	20121112	16966300	19970904	1	20121112	5
39186011	20120510	16411243	19500830	2	20120510	5
39187514	20120816	16706404	19611205	1	20120816	6
39192499	20120618	16524231	19560825	1	20120618	5
39202250	20121017	16890215	19540614	2	20121017	6
39202545	20120926	16823825	19381126	1	20120926	4
39207868	20120709	16588080	19631218	1	20120709	6
39208667	20120712	16599810	19521002	1	20120712	4
39209386	20120918	16798591	19391225	2	20120918	6
39214523	20120729	16645603	19400606	1	20120729	5
39220003	20121120	16992651	19401207	1	20121120	4
39227888	20130217	17250675	19991013	1	20130217	5
39227924	20120825	16729366	19350401	1	20120825	6
39231793	20120802	16657765	19540620	2	20120802	5
39238545	20131030	18054044	19491229	1	20131030	5
39250323	20130413	17431286	19660510	1	20130413	5
39258043	20120714	16606292	19590513	1	20120714	4
39261115	20130330	17383277	19560330	1	20130330	5
39266790	20120912	16785458	19820408	2	20120912	4
39269197	20120628	16551099	19730920	2	20120628	5
39276625	20120715	16606978	19690208	1	20120715	4
39279919	20121129	17018584	19551022	1	20121129	4
39280790	20120623	16537484	19260914	1	20120623	4
39287166	20120818	16711334	19490613	2	20120818	6
39287597	20120802	16657529	19290504	2	20120802	4
39292712	20120916	16794605	19661031	1	20120916	4
39299360	20130421	17453156	19911114	2	20130421	5
39303043	20120620	16531267	19581105	1	20120620	6
39303894	20120705	16577934	19620809	2	20120705	4
39306359	20120713	16603777	19751208	2	20120713	5
39308128	20120722	16627079	19400107	2	20120722	6
39309176	20120709	16588762	19700731	2	20120709	6
39313821	20120809	16684463	19760327	1	20120809	4
39313832	20120814	16698007	19710216	1	20120814	5
39318848	20120726	16639618	19661104	2	20120726	6
39319534	20121019	16896326	19670728	1	20121019	4
39323518	20120816	16706553	19730307	1	20120816	4
39331174	20120801	16654748	19700912	2	20120801	6
39333067	20130307	17315759	19651201	2	20130307	5
39335916	20120926	16824706	19840105	2	20120926	5
39338584	20121113	16971752	19410819	2	20121113	4
39341383	20130519	17541161	19950712	2	20130519	5
39342682	20131006	17982108	19380622	2	20131006	6
39343607	20120724	16633699	19521126	1	20120724	4

39346877	20120805	16664826	19620607	1	20120805	4
39350793	20120823	16725267	19890830	1	20120823	4
39354308	20120811	16690080	19410114	1	20120811	6
39355094	20120917	16796261	19510314	2	20120917	5
39356688	20121107	16953016	19520102	1	20121107	4
39359109	20120807	16674563	19500823	1	20120807	4
39365032	20120723	16628882	19570329	1	20120723	6
39367356	20130719	17735846	19211005	2	20130719	5
39368564	20120912	16784352	19381203	2	20120912	5
39369829	20120818	16711183	19311021	2	20120818	5
39372571	20121104	16938527	19470827	1	20121104	4
39374873	20120827	16732992	19430916	1	20120827	4
39379538	20130429	17474988	19551023	1	20130429	4
39381765	20120813	16693617	19460126	1	20120813	5
39382086	20121001	16836487	19631224	1	20121001	6
39384300	20130116	17171725	19440626	1	20130116	5
39385949	20121219	17087400	19610401	2	20121219	4
39389576	20120930	16831655	19280103	1	20120930	4
39389827	20130109	17148609	19540603	2	20130109	6
39393083	20130218	17252908	19500407	1	20130218	6
39394780	20121006	16857732	19510201	2	20121006	6
39401928	20130928	17954277	19470326	2	20130928	4
39402603	20120917	16798521	19680613	1	20120917	5
39403093	20121005	16853644	19610815	1	20121005	6
39405077	20130923	17936656	19661114	2	20130923	6
39422269	20130214	17246048	19470630	1	20130214	4
39426590	20120903	16750913	19410701	1	20120903	6
39437677	20120821	16717509	19720626	1	20120821	5
39452089	20121108	16955923	19470107	1	20121108	4
39455817	20121120	16992513	19300712	1	20121120	6
39461217	20130127	17199946	19650617	1	20130127	5
39471302	20130708	17698362	19560209	1	20130708	6
39480983	20121126	17009341	19300123	1	20121126	4
39481997	20130110	17153577	19530810	1	20130110	5
39483971	20130401	17388927	19480910	1	20130401	4
39485126	20121023	16905191	19410111	1	20121023	4
39488307	20130207	17239060	19580620	2	20130207	5
39489822	20121221	17095536	19600213	1	20121221	4
39496032	20120911	16780747	19490821	1	20120911	6
39498981	20130224	17274043	19541115	1	20130224	5
39503383	20120926	16822170	19411204	2	20120926	6
39503521	20121118	16986027	19500426	1	20121118	6
39515985	20120919	16805360	19451106	1	20120919	6
39517038	20121004	16850925	19620311	2	20121004	4
39520860	20121022	16902893	19541002	1	20121022	6
39521363	20121008	16862316	19560904	2	20121008	5
39526846	20130104	17132750	19521001	2	20130104	5
39530717	20120924	16817106	19400518	1	20120924	6
39531243	20120927	16827156	19570205	1	20120927	5
39531652	20121017	16889739	19660425	1	20121017	5
39531732	20121122	17001346	19430801	1	20121122	4
39531845	20130226	17281585	19301118	2	20130226	5
39532064	20121025	16912481	19490831	1	20121025	6
39536453	20121124	17006015	19280705	1	20121124	5
39540051	20121204	17034410	19661216	1	20121204	6

39545998	20121104	16938781	19700519	1	20121104	4
39549832	20121030	16920959	19380104	1	20121030	5
39552335	20121011	16871282	19390127	2	20121011	6
39556213	20121028	16917291	19530610	1	20121028	4
39556348	20130617	17628109	19581227	1	20130617	4
39557216	20130218	17254465	19250913	2	20130218	5
39564551	20121220	17091206	19370710	2	20121220	4
39565065	20121016	16885628	19330506	2	20121016	4
39566502	20121220	17092577	19451025	1	20121220	5
39567312	20121106	16947118	19600408	2	20121106	4
39567867	20121102	16934549	19701221	2	20121102	4
39570417	20121201	17024541	19551202	1	20121201	6
39571261	20121021	16899026	19510813	1	20121021	5
39580433	20121204	17033801	19670422	2	20121204	4
39582666	20121030	16923103	19320214	1	20121030	4
39585610	20121221	17094218	19651014	1	20121221	4
39587398	20130205	17230432	19560214	1	20130205	6
39588799	20121119	16988580	19580622	2	20121119	6
39589316	20130113	17159457	19660522	1	20130113	5
39596413	20121113	16971980	19491015	1	20121113	5
39598715	20121121	16997215	19530808	1	20121121	4
39606707	20130604	17589410	19340424	2	20130604	4
39610952	20121211	17061738	19460901	1	20121211	4
39622963	20130103	17126066	19570824	2	20130103	6
39625359	20130128	17203395	19691103	1	20130128	6
39631191	20121123	17004460	19350421	1	20121123	4
39634101	20121202	17025245	19580711	2	20121202	4
39636970	20130707	17693580	19600605	1	20130707	6
39642198	20130106	17135172	19580607	1	20130106	5
39645153	20121206	17044944	19731119	2	20121206	5
39645233	20130304	17302536	19590115	1	20130304	5
39645722	20121125	17006457	19550110	1	20121125	5
39649859	20130104	17132501	19890421	1	20130104	5
39653231	20121218	17085297	19530520	2	20121218	5
39653924	20121119	16989740	19700616	2	20121119	5
39656354	20130401	17390262	19600915	2	20130401	4
39657266	20130116	17171936	19621021	1	20130116	5
39661342	20130104	17132760	19561016	1	20130104	4
39661466	20121215	17076588	19510701	1	20121215	6
39662685	20130110	17151439	19500422	1	20130110	6
39664727	20130515	17531671	19530201	1	20130515	4
39667384	20121230	17115200	19680310	1	20121230	6
39671197	20130219	17259999	20020730	1	20130219	5
39672598	20130128	17202605	19600425	1	20130128	4
39680018	20131029	18051176	19521020	1	20131029	6
39684850	20130219	17259522	19570301	2	20130219	6
39688852	20121204	17036746	19580530	1	20121204	4
39692381	20130408	17412322	20050612	2	20130408	5
39693680	20130228	17287886	19550110	2	20130228	5
39696667	20130317	17345577	19410516	1	20130317	6
39697319	20130114	17162139	20020128	2	20130114	4
39697580	20121205	17039880	19401229	2	20121205	6
39698174	20121225	17104395	19731010	1	20121225	6
39699337	20121225	17101411	19621231	1	20121225	5
39699804	20130323	17365448	19610710	1	20130323	4

3971005920130217		17250548	19351101	1	20130217	5
3971166520131102		18064828	19300321	1	20131102	5
39711734	20121130	17022187	19801229	1	20121130	5
39717196	20121211	17062603	19821121	1	20121211	6
3972300720131225		18238239	19470203	2	20131225	5
3972432820130403		17400968	19651105	2	20130403	6
3973088620121227		17110024	19301205	2	20121227	5
3973340920130802		17778213	19341110	1	20130802	4
3974009520130129		17203757	19320112	1	20130129	5
3974052820130327		17376136	19640802	1	20130327	5
3974118920130106		17135197	19340419	1	20130106	5
3976883720130108		17145420	19710919	2	20130108	6
3976990920130719		17735282	19501125	1	20130719	6
3977173820130329		17382102	19521108	2	20130329	4
3977193220130113		17159832	19451124	1	20130113	4
3979004020130110		17152239	19670920	2	20130110	4
3979522720130115		17167039	19550517	1	20130115	4
3979604820130901		17865130	19390328	1	20130901	6
3979638820130118		17178291	19511228	1	20130118	6
3980505520130225		17277335	19721220	1	20130225	6
3980528220130206		17234195	19350213	1	20130206	4
3980542020130415		17435492	19680615	2	20130415	5
3981876320130218		17253829	19450829	1	20130218	6
3984237020130220		17262684	19571123	1	20130220	6
3984993920130729		17760803	19650428	1	20130729	5
3986840120130131		17211789	19420105	1	20130131	5
3986939120130903		17876209	19661115	2	20130903	4
3987559720130221		17268477	19370220	2	20130221	6
3988168020130213		17244565	19860325	2	20130213	5
3988508020130128		17203031	19820822	2	20130128	5
3988918420130206		17233444	19590403	2	20130206	6
3989083020130317		17345759	19750612	2	20130317	4
3989783120130223		17273280	19570531	1	20130223	5
3990085120130301		17294030	19860327	2	20130301	6
3990466020130824		17846886	19470401	2	20130824	6
3990689320130521		17547727	19550102	1	20130521	5
3991177820130311		17327881	19430905	1	20130311	5
3991321820130408		17412410	19641130	2	20130408	5
3992013320131125		18140613	19300907	1	20131125	6
3992146520130221		17267442	19350706	1	20130221	5
3992824020130304		17302592	19630525	2	20130304	4
3992911820130919		17929959	19430612	1	20130919	6
3992921020130708		17696278	19390506	1	20130708	4
3993171020130329		17381993	19710906	1	20130329	4
3993262220130730		17763096	19711114	1	20130730	6
3993473120130609		17605656	19510621	1	20130609	6
3993542720130313		17336265	19610215	2	20130313	6
3993797820130714		17716115	19640116	1	20130714	5
3993884620130306		17312546	19781119	2	20130306	6
3993952120130215		17248090	19511121	2	20130215	4
3994057320130320		17356110	20070726	1	20130320	6
3994117820130313		17334877	19511220	1	20130313	5
3994158720130828		17857249	19590615	2	20130828	5
3994181620131031		18058083	19460720	1	20131031	5
3994191820130415		17434085	19691113	1	20130415	5

3996053720130320	17356710	19711017	2	20130320	5
3996113220130225	17277955	19420915	2	20130225	6
3996554320130704	17687037	19580115	2	20130704	5
3997335820130413	17431361	19580624	2	20130413	4
3997372320130408	17412394	19360523	1	20130408	5
3997373420130419	17449109	19551002	1	20130419	6
3997718920130421	17453480	19640728	1	20130421	4
3997905020131205	18178477	19620530	1	20131205	4
3997940320130403	17400874	19660125	1	20130403	6
3998062620130421	17453233	19460417	1	20130421	6
3998143620130415	17434481	19380918	1	20130415	5
3998162920130725	17752445	19570725	1	20130725	6
3998288220130415	17435893	19761021	2	20130415	4
3998289320130317	17345679	19470203	1	20130317	6
3998305620130401	17388554	19620614	1	20130401	6
3998425320130711	17708101	19481124	1	20130711	5
3998485520130612	17615000	19391214	1	20130612	5
3998950920130515	17530769	19610601	1	20130515	5
3999559020130506	17500112	19590808	1	20130506	6
4000398320131012	18002750	19361125	2	20131012	6
4000724720130604	17588533	19420117	2	20130604	5
4000886420130318	17346478	19340823	1	20130318	5
4001096620130411	17424001	19730328	1	20130411	4
4001193620130422	17456973	19880207	2	20130422	4
4001546120130417	17444273	19761217	1	20130417	5
4002019720130419	17450198	19460218	1	20130419	5
4002394720130526	17560384	19470902	1	20130526	5
4002862420130421	17453075	19971209	1	20130421	4
4003233520130718	17732230	19560403	1	20130718	6
4003474020130430	17477666	19380830	1	20130430	4
4003607520130327	17374780	19511202	2	20130327	5
4003880020130517	17537921	19701219	2	20130517	4
4004337620130617	17629265	19550920	2	20130617	6
4004675120130412	17428003	19540516	2	20130412	6
4004901220130415	17434455	19600415	1	20130415	6
4005465720130409	17416163	19500222	1	20130409	4
4006907620130610	17610478	19590612	1	20130610	6
4008023720130414	17431842	19401018	1	20130414	5
4008045320130603	17583033	19820503	1	20130603	5
4008153620130923	17937998	19431018	1	20130923	4
4008264220130523	17555188	19610525	1	20130523	6
4008297120130624	17650824	19360316	1	20130624	5
4008321420130505	17496084	19410727	1	20130505	5
4008555020130525	17559935	19720626	2	20130525	6
4008816220130614	17619542	19510207	1	20130614	5
4013060720130723	17744378	19350721	1	20130723	5
4013246520130727	17757370	19550803	1	20130727	6
4013275020130715	17718854	19600405	1	20130715	4
4013351520131208	18185291	19730701	1	20131208	5
4013359320130716	17723597	19540713	1	20130716	6
4013729920131005	17981622	19481104	1	20131005	5
4013851020130513	17523366	19490711	2	20130513	5
4013960420130505	17496159	19720918	1	20130505	6
4013972820130709	17701439	19661118	1	20130709	6
4013988620130522	17551822	19440511	1	20130522	4

4014077020130930	17958208	19640214	1	20130930	5
4014290320130826	17850756	19510101	1	20130826	4
4014302020131014	18006607	19791102	1	20131014	5
4015071820130508	17509342	19451115	1	20130508	4
4015898320130530	17572489	19680325	1	20130530	5
4017570020130727	17757124	19470704	1	20130727	6
4018214720130721	17738273	19520211	1	20130721	5
4018438120131002	17969037	19270510	2	20131002	6
4019095220130919	17930093	19650905	2	20130919	4
4019572020130624	17650977	19731225	2	20130624	5
4019904020130716	17724357	19401016	1	20130716	6
4019973320130712	17714566	19620204	1	20130712	4
4020112520130608	17605104	19650620	1	20130608	5
4020176120130720	17737719	19330608	1	20130720	5
4020303020130605	17590240	19750905	1	20130605	4
4020323420130617	17628915	19570113	1	20130617	6
4020403320130815	17823487	19590420	1	20130815	4
4020688020130901	17864885	19720205	1	20130901	4
4020772520130602	17578398	19840727	1	20130602	5
4020940320130623	17647069	19340605	1	20130623	4
4021810820130709	17702627	19400126	2	20130709	5
4022325420130605	17593098	19310605	1	20130605	6
4022945620130625	17654017	19670425	1	20130625	5
4023268620130801	17772946	19940519	2	20130801	6
4023542520130604	17589370	19530926	1	20130604	4
4023993820130926	17946587	19590815	1	20130926	5
4024134720130624	17650224	19480120	1	20130624	6
4024143820130703	17681397	19970218	2	20130703	4
4024755020130817	17828750	19480421	1	20130817	4
4024758320131022	18032433	19650527	2	20131022	4
4024789020131109	18094432	19561108	1	20131109	4
4024791420130902	17870366	19631223	2	20130902	6
4025172720130614	17623121	19520913	1	20130614	6
4025370120130707	17693718	19440310	1	20130707	6
4025391620130620	17641623	19500410	1	20130620	4
4026343220130628	17663202	19471116	2	20130628	6
4026355620130725	17752682	19581107	2	20130725	6
4028308720130904	17880174	19341215	2	20130904	6
4028888820130708	17698588	19400409	1	20130708	4
4029082220130927	17951621	19520801	1	20130927	6
4029247520130928	17954261	19630805	2	20130928	6
4029652420130823	17845058	19590109	2	20130823	5
4029920520130802	17777523	20011013	2	20130802	5
4030400520130914	17917064	19461110	2	20130914	4
4030726420130721	17738037	19661214	1	20130721	5
4031105520130809	17801482	19450915	2	20130809	4
4031132820130802	17774536	19500328	2	20130802	4
4038078720130905	17884386	19821228	2	20130905	4
4038230720130916	17921215	19620407	1	20130916	5
4038642320131213	18206450	19260406	1	20131213	4
4038701720131219	18222835	19470203	1	20131219	5
4038932020130909	17897264	19380603	1	20130909	5
4039823020130823	17845384	19751025	1	20130823	5
4040477120131017	18018808	19371212	1	20131017	6
4041601120131112	18104251	19300104	1	20131112	6

4041698620130810	17806280	19790219	2	20130810	5
4041726320131030	18054251	19540914	2	20131030	6
4042014220130815	17822133	19330515	1	20130815	6
4042390320131027	18046101	19490115	1	20131027	5
4042613920131015	18010550	19510327	1	20131015	4
4042639920130813	17814390	19390822	1	20130813	5
4042921820130912	17910406	19481110	2	20130912	6
4043015720130820	17836991	20130129	1	20130820	4
4044047920131020	18025786	19550520	1	20131020	6
4044772120131228	18245493	19490427	2	20131228	5
4045121620130823	17845076	19440920	1	20130823	5
4045263920131030	18055426	19490110	1	20131030	6
4045960720130909	17897922	19250322	2	20130909	6
4047387820131008	17991030	19391015	2	20131008	5
4047418820131006	17982162	19411113	1	20131006	4
4047851120131209	18189641	19681205	2	20131209	5
4048235720130916	17921917	19540910	1	20130916	6
4048564120130909	17896428	19420615	1	20130909	5
4048591420130911	17906616	19511230	1	20130911	6
4048605320131110	18095173	19710202	1	20131110	5
4049011720130911	17907458	19491211	2	20130911	5
4049035520130919	17930039	19420331	1	20130919	5
4049289520130916	17920255	19650826	1	20130916	4
4049799220130913	17915147	19621210	2	20130913	5
4050447020131031	18058423	19520125	1	20131031	5
4050469620131103	18066727	19790811	2	20131103	5
4051664120130915	17917676	19561110	2	20130915	5
4052061420130908	17893967	19580103	1	20130908	6
4052418320131024	18040730	19511007	2	20131024	4
4052714820131110	18094969	19701107	1	20131110	6
4052838920131030	18055294	20031010	1	20131030	6
4053948820131012	18002636	19550825	1	20131012	6
4060661920131018	18022329	19680404	1	20131018	6
4061328320131022	18033532	19631129	2	20131022	6
4062553420131027	18045994	19691104	1	20131027	6
4064674020131118	18121216	19580126	1	20131118	5
4064930720131104	18070037	19671010	1	20131104	5
4065056420131125	18141791	19590530	1	20131125	5
4065080420131112	18104546	19351027	1	20131112	5
4065175020131029	18052607	19610712	2	20131029	5
4065501420131126	18143631	19710521	2	20131126	5
4065911820131111	18097737	19570808	1	20131111	6
4066256420131205	18178427	19500920	2	20131205	5
4066766120131230	18248538	19420804	2	20131230	4
4067170320131124	18138069	19400830	2	20131124	6
4067816820131117	18118190	19710509	1	20131117	5
4069110920131015	18010241	19590715	2	20131015	5
4069166520131216	18212624	19371020	1	20131216	5
4069713020131028	18048707	19520805	1	20131028	6
4072063620131218	18218653	19610301	1	20131218	5
4072243820131216	18212199	19501210	2	20131216	6
4073458520131226	18239247	19680213	2	20131226	5
4076207020131222	18228378	19360521	1	20131222	4
4076209220131213	18205655	19490801	2	20131213	5
4076285420131212	18202616	19580228	1	20131212	6

40772905	20131218	18219972	19751001	2	20131218	4
40863454	20131205	18176707	19491206	1	20131205	5
40863476	20131118	18121330	19410303	1	20131118	5
122634	20111112	15882771	19280506	1	20111112	4
146598	20110425	15294071	19330318	1	20110425	6
227769	20120618	16523680	19301225	1	20120618	5
243992	20130407	17407353	19301226	1	20130407	4
313779	20121108	16957241	19320108	1	20121108	5
361331	20131124	18137984	19420219	2	20131124	6
411530	20130325	17368324	19230120	1	20130325	6
424237	20120103	16032850	19290906	2	20120103	6
428284	20121209	17053395	19241025	1	20121209	4
469590	20120308	16223259	19240706	1	20120308	5
485938	20120326	16270557	19320601	1	20120326	5
577311	20121216	17077342	19260612	1	20121216	6
687343	20120330	16283400	19340915	2	20120330	6
769540	20121104	16938548	19440918	2	20121104	5
781782	20131123	18137535	19450415	2	20131123	6
864722	20120615	16518002	19240903	1	20120615	6
879345	20130802	17778226	19320303	1	20130802	6
884333	20130117	17174137	19251018	1	20130117	5
1028166	20121223	17097604	19261020	1	20121223	6
1178887	20130508	17508752	19670525	2	20130508	6
1195682	20110906	15687823	19670603	1	20110906	4
1199640	20120202	16111956	19330618	1	20120202	6
1242882	20130407	17407143	19421015	2	20130407	6
1342796	20120321	16259794	19281221	1	20120321	4
1384958	20120329	16280362	19360809	2	20120329	4
1424846	20131015	18012127	19470628	2	20131015	5
1488535	20130112	17159037	19320202	1	20130112	4
1556861	20120409	16315108	19330614	1	20120409	5
1612664	20111021	15817325	19281023	1	20111021	4
1777237	20130903	17873850	19240504	2	20130903	4
1822602	20111225	16005711	19360408	1	20111225	5
1832208	20120726	16638771	19251222	1	20120726	5
1904796	20131222	18228239	19300103	1	20131222	5
1947144	20110330	15216873	19220729	1	20110330	6
1984925	20120912	16785120	19190712	1	20120912	4
2107837	20130225	17278288	19290312	1	20130225	6
2131206	20130410	17421185	19411009	2	20130410	4
2160932	20121008	16862877	19221120	1	20121008	5
2251947	20110822	15644114	19491107	2	20110822	6
2327620	20111206	15952505	19260929	1	20111206	5
2386472	20120101	16022498	19340120	1	20120101	5
2418293	20120417	16338844	19330223	1	20120417	6
2468920	20130627	17660954	19230130	1	20130627	4
2508283	20120113	16066743	19241224	2	20120113	6
2533597	20120111	16059992	19290804	1	20120111	6
2559297	20110318	15187542	19671109	2	20110318	5
2571359	20110607	15419185	19391120	2	20110607	4
2605472	20130729	17760828	19390818	1	20130729	6
2655381	20130521	17548460	19280905	1	20130521	5
2691830	20111010	15782947	19390118	2	20111010	6
2892866	20130521	17547417	19490525	1	20130521	4
3035663	20130329	17381927	19291103	1	20130329	5

3261094	20120422	16352075	19280728	2	20120422	6
3599335	20121218	17085452	19500410	1	20121218	6
3678168	20120119	16084206	19510926	1	20120119	5
3696911	20120113	16066795	19370220	1	20120113	6
3740974	20131122	18135050	19411203	1	20131122	5
3839187	20130824	17846903	19240805	2	20130824	5
3877676	20120401	16285447	19280319	1	20120401	5
4180794	20120404	16298683	19300624	1	20120404	6
4234791	20110924	15737403	19361112	2	20110924	5
4273852	20130421	17453136	19500807	2	20130421	6
4278153	20130401	17388670	19310510	1	20130401	4
4309277	20131002	17968303	19240719	1	20131002	4
4458031	20120208	16132896	19540111	1	20120208	5
4766078	20131014	18004472	19291126	2	20131014	4
4791326	20120614	16514499	19271226	2	20120614	6
4826691	20131110	18094841	19510301	1	20131110	4
4915257	20120709	16588198	19390715	2	20120709	4
4919113	20130310	17324177	19510926	2	20130310	5
4956052	20110406	15237981	19390723	1	20110406	6
4984741	20111222	15999460	19300816	1	20111222	5
5170365	20131211	18198330	19471116	1	20131211	5
5267323	20130710	17707561	19450801	2	20130710	5
5272559	20121206	17045659	19260505	1	20121206	6
5522872	20110806	15600302	19470301	1	20110806	4
5559795	20120603	16474593	19310724	1	20120603	4
5606428	20130827	17853673	19310104	1	20130827	4
5631754	20130204	17225524	19571015	2	20130204	6
5646468	20121209	17053067	19320905	1	20121209	6
5707415	20110613	15439182	19421218	1	20110613	4
6436357	20120329	16280401	19260717	1	20120329	4
6469870	20121014	16878330	19470401	2	20121014	4
6554581	20131126	18145410	19710421	2	20131126	4
6660362	20121203	17030662	19590110	2	20121203	4
6766967	20110417	15272513	19560208	2	20110417	5
6804779	20130405	17404769	19310106	2	20130405	5
6920065	20131225	18238281	19261011	1	20131225	5
7091421	20120301	16195276	19260602	1	20120301	5
7282351	20121122	16999626	19310828	1	20121122	4
7291261	20111115	15891130	19310801	1	20111115	5
7422415	20110606	15415163	19200209	1	20110606	5
7790810	20110323	15202140	19270510	1	20110323	4
7869661	20131007	17986990	19251106	2	20131007	6
7871616	20110810	15610691	19420324	1	20110810	5
7910330	20111206	15951367	19401229	2	20111206	4
7966383	20120928	16829778	19500423	1	20120928	6
7982005	20110317	15187318	19360310	1	20110317	4
8009754	20120525	16452895	19260929	1	20120525	4
8140612	20121231	17117028	19270412	1	20121231	6
8196198	20120709	16588753	19450825	2	20120709	4
8302474	20130521	17548358	19330316	1	20130521	5
8372187	20111019	15811506	19360312	2	20111019	4
8377400	20111019	15810823	19290608	1	20111019	6
8419827	20120104	16037074	19270312	1	20120104	5
8609903	20120531	16466271	19470705	2	20120531	4
8617376	20111015	15800325	19411020	1	20111015	5

8667650	20120411	16323715	19570420	2	20120411	6
8683509	20110930	15753476	19391122	2	20110930	6
9080028	20120108	16047509	19501120	2	20120108	4
9132347	20110907	15692451	19480618	1	20110907	4
9133179	20131119	18125749	19290216	1	20131119	4
9226419	20120505	16394639	19540514	2	20120505	5
9485954	20120422	16351760	19300802	2	20120422	4
9518358	20120905	16761085	19320206	1	20120905	4
9557226	20120612	16505946	19600906	2	20120612	6
9584161	20120815	16701173	19320928	2	20120815	4
9587693	20130625	17654365	19401001	2	20130625	5
9685998	20130414	17431578	19310301	1	20130414	5
9868068	20110511	15346702	19521201	1	20110511	4
9984092	20120512	16417118	19540926	2	20120512	6
9990970	20110906	15687990	19740511	2	20110906	6
9991746	20111227	16010369	19570914	1	20111227	5
10074869	20110210	15078063	19330918	1	20110210	4
10131029	20110531	15397772	19370412	1	20110531	6
10201511	20121206	17045887	19550320	1	20121206	6
10333365	20130416	17437874	19280421	1	20130416	5
10343427	20130702	17674066	19300103	1	20130702	6
10432049	20131225	18237210	19270714	1	20131225	6
10754968	20111011	15787253	19520820	2	20111011	4
11284416	20130905	17885978	19420528	1	20130905	6
11575523	20110617	15453433	19420710	1	20110617	6
11598417	20120429	16369354	19381213	2	20120429	4
11722499	20110608	15423994	19370817	2	20110608	6
11776253	20110611	15434252	19370813	2	20110611	6
11777723	20130611	17614577	19290511	1	20130611	5
11787250	20120217	16161411	19620111	2	20120217	6
11819684	20130824	17846624	19621027	1	20130824	6
12018530	20130727	17756874	19310226	1	20130727	5
12110655	20120816	16705656	19521017	2	20120816	6
12219953	20121017	16890347	19630917	2	20121017	6
12238367	20131109	18094577	19520412	1	20131109	6
12245635	20110731	15575142	19400716	2	20110731	5
12453417	20120921	16811870	19230219	1	20120921	4
12548413	20120130	16097985	19570319	1	20120130	4
12587938	20130828	17858076	19580121	1	20130828	6
12639848	20130821	17838096	19551028	2	20130821	6
12640276	20130123	17191703	19460310	2	20130123	6
12677644	20111212	15969390	19240704	1	20111212	5
12688470	20121024	16909553	19551215	2	20121024	5
12693060	20120313	16236894	19750428	1	20120313	5
12723238	20120424	16356668	19361202	2	20120424	4
12795492	20130215	17246905	19310125	1	20130215	6
12798435	20130620	17640795	19510902	1	20130620	5
12857975	20130708	17694935	19551024	1	20130708	5
12940997	20120501	16379186	19821109	1	20120501	5
13007211	20120322	16263671	19390329	2	20120322	5
13058098	20130508	17509567	19531209	1	20130508	5
13060963	20120120	16085564	19350911	2	20120120	5
13251848	20120710	16592389	19701029	1	20120710	4
13378273	20121022	16902982	19310125	1	20121022	6
13412889	20130202	17220050	19250701	2	20130202	6

1342541720130316		17345207	19631111	1	20130316	6
13519158	20110806	15600286	19510905	2	20110806	6
13574431	20111201	15936061	19550122	1	20111201	6
13627468	20111008	15781901	19421123	1	20111008	4
13803297	20120424	16357308	19440528	2	20120424	5
13840374	20120912	16784276	19281229	1	20120912	5
13881577	20120317	16249928	19230125	1	20120317	5
13991827	20120917	16797911	19491026	1	20120917	6
1401566820131129		18154691	19480903	1	20131129	5
1402157920130815		17822523	19490901	1	20130815	4
1405169520130623		17646962	19410226	1	20130623	5
1408448120130810		17806312	19531025	1	20130810	5
14104257	20110506	15329646	19271008	2	20110506	5
14143749	20120426	16363742	19600222	2	20120426	6
14235122	20110825	15654456	19521031	1	20110825	4
14405288	20111030	15837959	19260924	1	20111030	4
1444594420130603		17582569	19670331	1	20130603	5
14477680	20120613	16511658	19340709	2	20120613	5
1449059620130615		17625075	19450602	2	20130615	5
1456862820131209		18187566	19511205	2	20131209	4
14600954	20120224	16179767	19500605	1	20120224	5
14788615	20120806	16669997	19531020	2	20120806	4
14823155	20110317	15187447	19630729	2	20110317	6
14863219	20110621	15463143	19481214	2	20110621	4
1489785920130310		17324261	19280523	1	20130310	4
14926477	20110314	15176683	19460224	2	20110314	5
14992484	20110426	15297309	19540822	2	20110426	5
15142253	20120722	16626958	19730315	1	20120722	4
15379494	20120214	16151552	19510822	2	20120214	6
15411435	20120301	16196590	19500621	2	20120301	4
1549614720130810		17806179	19251128	1	20130810	5
15538257	20120828	16734985	19180505	1	20120828	4
1555151620130417		17443538	19310407	2	20130417	4
15587050	20111004	15766478	19530506	1	20111004	4
1565132820130722		17742047	19560109	2	20130722	4
15657326	20120905	16762296	19340416	2	20120905	5
1567114420130603		17582620	19291231	2	20130603	6
15678509	20111218	15987113	19310815	1	20111218	6
1572775020130531		17575377	19470802	2	20130531	5
15780844	20120603	16474492	19570829	1	20120603	4
15792139	20110424	15292325	19390601	1	20110424	4
15807597	20120805	16664895	19320410	1	20120805	5
15833815	20121005	16854773	19310204	1	20121005	5
15834374	20111015	15800455	19221111	1	20111015	5
1585877220130628		17662350	19291230	1	20130628	4
1602440120121227		17109492	19790805	1	20121227	6
1623321920130202		17219796	19500409	1	20130202	4
1624200520131125		18141585	19320529	1	20131125	5
16249379	20120507	16399174	19310606	1	20120507	5
1632396920131209		18188177	19320405	1	20131209	5
16621731	20120116	16072770	19500325	1	20120116	5
16669106	20110630	15485636	19260820	2	20110630	6
16697113	20110905	15682705	19680524	2	20110905	6
16719963	20120508	16403607	19340628	2	20120508	4
16726059	20111102	15851552	19361211	1	20111102	6

1678694020130410		17419854	19230713	1	20130410	6
16803182	20111221	15996447	19270713	1	20111221	4
16892038	20111128	15925313	19510616	1	20111128	6
1689402320131215		18208875	19690608	1	20131215	5
16899437	20110913	15706357	19700201	2	20110913	4
16956700	20111012	15790872	19540606	1	20111012	5
17065462	20121101	16930702	19570105	1	20121101	5
17196359	20111213	15973794	19370804	1	20111213	6
17261186	20111021	15818415	19520511	2	20111021	5
1726757120130122		17188397	19650208	2	20130122	6
1727206920130404		17403231	19530907	1	20130404	5
1731433920130903		17877181	19600815	2	20130903	5
17518808	20120423	16355190	19621025	1	20120423	6
17530131	20110524	15382109	19330926	1	20110524	6
1757787620130708		17698142	19540924	1	20130708	5
17591230	20121209	17053080	19510525	2	20121209	4
17595801	20110509	15335332	19321127	2	20110509	6
17605117	20120401	16285413	19450107	2	20120401	4
17617708	20110804	15593845	19250715	1	20110804	4
1772210020130131		17212106	19601010	2	20130131	6
17724811	20120609	16499247	19310410	2	20120609	4
18010758	20120717	16613060	19291201	2	20120717	4
18082672	20111002	15756483	19210901	1	20111002	4
18196800	20110518	15364703	19270707	1	20110518	5
18244763	20120520	16436942	19550601	1	20120520	6
18251315	20110409	15249470	19320915	1	20110409	4
18309109	20110523	15378916	19460804	1	20110523	4
1834661720131009		17996503	19350515	1	20131009	5
18460509	20110930	15753597	19340321	2	20110930	4
18491162	20111016	15800817	19440222	1	20111016	5
1852524120130308		17320278	19250819	2	20130308	5
18568677	20110822	15644722	19480824	1	20110822	4
18585609	20111023	15820574	19561210	1	20111023	6
18676432	20120428	16368985	19790602	2	20120428	6
18696736	20120914	16789707	19431115	1	20120914	6
18858338	20110906	15688332	19591004	2	20110906	4
18865015	20110831	15666469	19480403	1	20110831	5
18901412	20120622	16536795	19301230	1	20120622	6
18966502	20120425	16360045	19300625	1	20120425	5
19107052	20110421	15287668	19311230	1	20110421	5
1918592320130107		17139343	19831005	2	20130107	4
19300573	20121126	17010061	19580702	1	20121126	4
19350744	20121118	16985772	19280913	1	20121118	5
19606792	20110504	15324834	19490502	1	20110504	4
19608447	20120612	16508130	19250223	2	20120612	5
19636987	20110324	15205866	19381210	2	20110324	5
1965457020130727		17757002	19280407	1	20130727	6
19726900	20110514	15356228	19571020	2	20110514	5
1984814720130512		17519375	19720704	1	20130512	6
19937963	20120809	16684380	19640315	2	20120809	6
1997673920130421		17453443	19520102	1	20130421	5
20050026	20120612	16508139	19630102	2	20120612	5
2005318320130608		17604595	19311227	2	20130608	5
20271129	20120703	16566852	19430401	2	20120703	5
20426760	20120123	16088748	19331111	1	20120123	4

20428528	20131228	18245435	19650227	1	20131228	4
20486297	20110812	15620220	19590227	1	20110812	4
20488602	20130429	17473358	19520919	1	20130429	6
20490248	20121102	16933733	19231015	1	20121102	6
20684484	20131018	18023407	19261008	2	20131018	4
20717866	20130713	17715368	19530704	2	20130713	6
20772445	20130813	17813937	19440501	2	20130813	6
20872268	20130617	17628401	19650801	1	20130617	5
20940276	20110621	15463097	19410618	1	20110621	4
20989519	20120112	16063890	19320324	1	20120112	4
20990345	20110713	15530974	19301203	1	20110713	5
20996069	20131119	18125239	19680930	1	20131119	6
21012806	20130128	17202273	19341213	2	20130128	4
21094291	20130605	17594243	19321130	2	20130605	6
21101008	20111013	15795006	19270501	1	20111013	4
21151882	20120225	16182255	19420715	1	20120225	5
21184985	20120515	16425320	19510223	1	20120515	5
21280499	20130122	17188319	19520722	2	20130122	4
21360623	20131015	18009834	19260305	1	20131015	5
21436828	20131107	18088463	19261114	1	20131107	4
21438675	20131204	18172906	19580825	1	20131204	5
21441236	20120508	16401852	19231127	1	20120508	5
21458060	20120621	16534178	19310318	1	20120621	6
21516610	20120801	16654823	19751002	1	20120801	5
21527286	20130930	17958075	19490212	2	20130930	5
21619750	20130103	17128413	19680719	2	20130103	6
21647290	20120604	16479849	19270716	1	20120604	5
21655958	20130130	17209321	19601105	1	20130130	6
21674577	20130615	17625093	19390510	2	20130615	6
21674624	20120325	16268628	19510420	2	20120325	5
22246351	20110925	15738232	19651205	2	20110925	5
22337628	20130925	17945684	19480225	2	20130925	5
22568907	20111018	15808374	19361005	2	20111018	6
22575980	20110406	15237783	19580429	2	20110406	4
22578230	20120703	16567175	19281015	1	20120703	6
22648891	20120130	16096238	19470428	1	20120130	5
22668151	20120423	16355482	19311011	1	20120423	4
22694899	20130802	17776032	19290510	2	20130802	4
22831454	20131105	18078404	19351012	1	20131105	4
22881545	20130807	17795981	19380816	2	20130807	4
22932645	20120321	16260917	19630120	2	20120321	5
22989340	20110731	15575218	19471110	1	20110731	6
23005529	20111020	15815205	19321211	1	20111020	6
23084653	20120429	16369728	19680118	2	20120429	5
23105284	20120817	16708369	19400405	2	20120817	6
23129364	20121001	16836681	19570226	1	20121001	6
23133995	20111213	15973925	19481120	1	20111213	6
23157199	20130828	17857893	19460714	2	20130828	5
23175420	20130808	17799706	19580120	1	20130808	6
23178361	20111227	16012635	19340322	1	20111227	4
23265647	20120105	16041281	19361120	1	20120105	4
23410620	20120711	16595918	19680102	1	20120711	4
23419503	20130428	17471548	19310216	1	20130428	5
23523991	20110615	15445414	19560810	1	20110615	4
23540310	20130417	17444015	19420705	2	20130417	5

23601265	20110306	15149994	19580623	1	20110306	5
2376029420131215		18208790	19470104	1	20131215	6
23764854	20110310	15167001	19270606	1	20110310	4
23797571	20110413	15263520	19610130	2	20110413	6
23852020	20120128	16094056	19261214	2	20120128	6
24203938	20110804	15594323	19370224	2	20110804	6
24224804	20121114	16975894	19240108	2	20121114	4
24272591	20120822	16722502	19611125	1	20120822	5
24337222	20120807	16673915	19470510	1	20120807	5
2434011220130625		17654324	19550412	2	20130625	6
24415701	20120705	16576293	19651216	1	20120705	6
24424848	20121119	16990126	19260302	1	20121119	4
24459776	20111228	16015605	19740226	1	20111228	6
2463250620130603		17583168	19270918	1	20130603	6
24748912	20120203	16115806	19250925	1	20120203	4
2479408920130822		17841427	19410927	1	20130822	4
24817672	20111218	15987294	19490404	2	20111218	5
2482614020130228		17288243	19410605	1	20130228	5
24878306	20121106	16946007	19261105	1	20121106	5
24887647	20110513	15354121	19200903	2	20110513	5
24906103	20110715	15536610	19631101	1	20110715	4
25032820	20120406	16306040	19261227	1	20120406	6
2507281520130327		17373737	19310117	1	20130327	6
25074253	20110527	15390476	19570802	2	20110527	4
25131877	20120427	16367305	19300214	2	20120427	4
25156869	20110728	15570945	19530724	2	20110728	6
2521643720130417		17443153	19241012	1	20130417	4
25257641	20120307	16218899	19661130	2	20120307	6
25335299	20120531	16466508	19600404	1	20120531	5
2543387820130412		17427205	19470223	2	20130412	4
25489718	20120516	16428373	19370805	2	20120516	5
25549104	20120601	16471940	19491007	2	20120601	4
25579742	20121116	16982419	19481030	1	20121116	4
25587706	20110321	15196203	19500905	2	20110321	4
25631510	20120417	16338664	19451014	1	20120417	5
2572959320130531		17575498	19311212	2	20130531	6
25837529	20120726	16640283	19381017	1	20120726	4
25874333	20120610	16500184	19310115	1	20120610	5
25982187	20120312	16232547	19540623	1	20120312	4
25987820	20120128	16093609	19570224	2	20120128	4
26062748	20110816	15629216	19680517	1	20110816	6
26126114	20121121	16996604	19371012	1	20121121	5
2613037020130102		17121046	19280816	2	20130102	6
26232199	20110822	15644744	19580102	1	20110822	5
26270860	20110918	15719223	19631127	2	20110918	5
26336685	20111002	15756336	19641207	1	20111002	4
2664773420131113		18108767	19810806	2	20131113	5
26675070	20120319	16254128	19481125	1	20120319	5
26696082	20120514	16420441	19540606	1	20120514	4
26721697	20120322	16263485	19421210	2	20120322	4
2679353320130515		17531595	19400324	1	20130515	4
2679657620131127		18148991	19451219	2	20131127	4
26915684	20111128	15925312	19640605	1	20111128	4
2697046720131105		18076093	19760614	2	20131105	6
26975053	20110923	15736390	19630617	2	20110923	5

27066428	20121104	16938793	19510206	1	20121104	5
27110210	20120210	16140704	19370325	1	20120210	6
27158141	20121021	16899199	19310120	2	20121021	5
27175924	20131117	18117978	19510214	1	20131117	4
27245950	20130820	17835585	19410101	2	20130820	5
27259047	20120416	16337226	19530306	1	20120416	5
27322458	20121116	16983288	19491001	1	20121116	4
27354081	20120524	16450115	19390913	1	20120524	6
27417216	20130218	17254818	19421118	2	20130218	5
27453174	20130513	17521656	19520215	1	20130513	4
27473218	20120204	16119094	19400110	2	20120204	6
27482004	20110809	15607967	19470103	2	20110809	6
27509019	20110924	15737897	19381026	1	20110924	5
27520985	20121217	17081012	19471215	2	20121217	4
27561995	20121031	16925597	19340525	2	20121031	4
27643476	20130823	17844322	19300127	1	20130823	6
27726927	20120322	16262824	19490809	2	20120322	4
27736807	20111218	15986986	19370118	2	20111218	6
27746425	20110405	15233862	19550811	2	20110405	4
27771455	20110518	15367376	19660831	2	20110518	5
27806059	20130505	17496282	19330616	2	20130505	5
27859821	20110621	15462024	19590226	2	20110621	5
27865163	20121205	17041528	19540315	2	20121205	6
27879114	20110817	15633175	19650305	2	20110817	5
27911928	20120925	16820695	19520129	1	20120925	6
27913402	20130308	17321533	19550328	2	20130308	5
27990256	20130219	17259691	19500415	2	20130219	4
28018884	20110404	15231577	19461010	2	20110404	5
28040757	20130607	17602774	19410316	1	20130607	5
28086013	20120728	16643719	19350419	2	20120728	5
28110089	20130118	17178259	19650328	1	20130118	6
28264053	20120316	16247970	19360222	1	20120316	6
28335902	20131119	18124068	19450105	2	20131119	6
28398267	20120827	16732853	19630228	1	20120827	4
28439192	20131111	18098481	19611116	2	20131111	4
28509559	20120620	16531143	19550328	2	20120620	5
28531308	20130909	17898001	19550601	2	20130909	5
28541857	20131121	18132931	19460622	2	20131121	4
28562530	20131204	18172890	19720824	1	20131204	4
28567875	20131014	18005294	19350824	1	20131014	6
28643369	20120108	16047629	19640409	2	20120108	4
28669069	20120728	16644895	19560125	1	20120728	5
28793671	20120109	16051926	19500915	1	20120109	6
28844726	20120625	16540152	19371104	2	20120625	5
28885861	20111004	15766934	19360726	1	20111004	6
28885974	20120610	16500221	19571124	1	20120610	4
28927460	20130618	17633821	19501125	1	20130618	5
28955364	20111201	15936500	19331022	2	20111201	6
29019849	20110605	15414597	19470405	2	20110605	6
29080613	20120824	16727708	19280910	2	20120824	6
29113595	20110425	15294553	19870623	2	20110425	5
29147215	20121018	16891000	20000912	1	20121018	5
29147715	20131203	18164934	19390108	1	20131203	6
29150343	20130924	17940898	19340606	2	20130924	5
29169004	20130707	17693777	19580504	2	20130707	4

29237556	20130823	17843555	19320818	2	20130823	6
29264720	20131029	18051857	19560802	2	20131029	4
29359522	20120403	16294929	19270118	2	20120403	5
29388816	20120331	16284931	19860429	1	20120331	5
29473287	20121231	17116195	19261115	2	20121231	5
29486393	20110806	15599957	19490402	2	20110806	5
29526690	20111026	15830380	19610922	2	20111026	4
29625670	20120724	16633539	19600220	1	20120724	6
29772972	20120703	16565172	19641030	1	20120703	4
29847663	20131124	18137820	19440908	1	20131124	5
29941104	20110919	15723021	19320603	1	20110919	6
29950387	20130111	17157383	19661025	2	20130111	6
30002007	20120514	16419822	19670116	2	20120514	6
30007477	20130202	17219654	19520730	1	20130202	5
30008072	20111122	15910260	19610405	2	20111122	4
30009097	20120202	16112912	19730301	2	20120202	4
30104166	20111109	15874634	19490217	2	20111109	4
30160384	20110517	15362519	19701017	1	20110517	5
30262603	20120305	16209437	19610327	1	20120305	4
30270156	20120704	16571788	19410611	2	20120704	6
30290723	20110228	15128342	19660524	1	20110228	6
30293960	20110928	15748114	19620616	1	20110928	5
30295217	20120424	16358246	19611120	1	20120424	6
30406883	20110603	15409999	19320103	1	20110603	5
30413479	20130609	17605776	19510501	2	20130609	5
30424932	20130120	17180684	19300929	1	20130120	5
30427919	20110319	15191840	19540516	1	20110319	6
30454401	20120130	16095915	19300801	1	20120130	5
30490541	20121226	17107304	19220301	1	20121226	4
30507905	20131014	18007642	19360526	2	20131014	5
30548573	20131029	18052609	19690125	1	20131029	6
30619900	20110919	15723192	19590110	1	20110919	5
30701929	20130512	17519391	19570622	1	20130512	6
30709081	20120529	16460958	19481102	2	20120529	4
30740488	20120103	16031770	19600206	2	20120103	4
30798959	20130428	17471813	19271015	1	20130428	5
30811360	20131014	18007783	19410126	1	20131014	5
30873906	20120926	16823562	19271015	1	20120926	6
30902375	20120905	16761456	19430416	1	20120905	6
30945803	20131129	18154658	19640205	1	20131129	5
30974528	20120416	16337122	19530509	2	20120416	6
31015595	20130620	17641788	19570110	2	20130620	5
31095359	20120201	16107959	19340702	1	20120201	5
31137914	20120920	16809090	19650529	1	20120920	4
31152542	20130928	17954221	19530320	2	20130928	4
31254758	20121011	16872090	19590218	1	20121011	6
31336751	20130528	17566517	19970912	1	20130528	5
31355336	20120802	16657433	19420514	1	20120802	6
31491200	20130717	17728451	19360108	1	20130717	4
31538462	20120212	16143288	19500102	1	20120212	4
31544475	20131215	18208695	19540102	1	20131215	4
31550513	20110506	15332853	19580316	2	20110506	5
31558733	20121211	17062994	19500114	2	20121211	5
31678418	20110221	15110395	19570616	1	20110221	5
31729132	20120515	16425378	19390623	2	20120515	6

3173563420130501		17484016	19371015	1	20130501	6
3173826820131015		18012200	19411110	1	20131015	4
3176565820130515		17530697	19331008	1	20130515	5
31917176	20121127	17012056	19440417	1	20121127	5
31981336	20120321	16260749	19630522	1	20120321	5
3199804620130515		17530606	19610203	2	20130515	6
32012069	20110329	15215382	19670127	1	20110329	4
32044556	20120423	16354369	19450527	1	20120423	6
32050161	20120916	16794537	19301020	2	20120916	5
3206104420130902		17869629	19360322	1	20130902	5
3206659420130924		17942070	19420927	1	20130924	6
32105047	20120202	16112899	19550510	1	20120202	5
32160217	20120619	16527770	19390120	2	20120619	6
32245855	20110309	15163136	19540501	1	20110309	5
32332335	20110929	15750180	19380128	1	20110929	5
3242691820131121		18132170	19550129	1	20131121	5
3247538020131110		18094820	19380901	1	20131110	6
32477955	20110915	15713963	19341202	2	20110915	5
32573652	20111216	15984156	19410227	2	20111216	5
32623680	20120824	16727848	19510715	2	20120824	4
3262665620131120		18127889	19290801	1	20131120	4
32648343	20120720	16623377	19610717	1	20120720	5
3269621220130815		17821803	19690914	1	20130815	5
32742793	20120529	16460758	19640113	1	20120529	4
32776344	20120810	16688097	19650101	1	20120810	6
32797481	20120224	16180773	19510302	1	20120224	6
32818498	20110606	15415181	19651207	1	20110606	4
32899164	20120318	16250520	19650320	2	20120318	4
32916419	20110624	15472212	19540607	2	20110624	6
33047540	20120707	16583646	19521205	1	20120707	5
33052958	20120522	16443059	19521101	1	20120522	4
33089939	20120126	16090738	19480106	2	20120126	6
33118331	20110301	15133244	19420927	2	20110301	4
33135681	20120701	16557146	20040507	2	20120701	5
33140453	20111026	15829845	19540807	1	20111026	6
3314953020130813		17815294	19430527	2	20130813	4
33160440	20110913	15706189	19600321	1	20110913	5
33161454	20121017	16888793	19450116	1	20121017	6
3318083520130714		17715719	19590101	1	20130714	6
33196666	20111204	15943518	19871127	1	20111204	4
33242454	20120814	16695963	19690430	2	20120814	6
33275068	20110808	15605024	19460701	2	20110808	5
33330324	20110425	15296040	19681226	1	20110425	5
33336366	20120809	16684369	19890614	1	20120809	6
33371878	20120131	16101648	19390822	2	20120131	5
33371914	20120427	16367433	19510922	1	20120427	4
33433033	20121014	16878356	19370719	1	20121014	4
3344414320130218		17254820	19650921	2	20130218	4
3345104620130129		17206369	19580918	2	20130129	5
3350090220131202		18162743	19451009	2	20131202	4
33514362	20111025	15827254	19650430	1	20111025	5
33541116	20110422	15290670	19410727	1	20110422	6
3356003120130117		17175063	19340415	2	20130117	6
3358973020130102		17123264	19700409	1	20130102	5
3367639020130102		17123280	19651228	1	20130102	4

3368878720130326		17372193	19391022	1	20130326	4
3371208220130712		17714741	19420614	1	20130712	6
33718206	20111211	15966328	19540925	2	20111211	5
3372811920130521		17547400	19641207	1	20130521	6
33767532	20120907	16770666	19571022	1	20120907	6
33920966	20120813	16694852	19621119	1	20120813	5
34047875	20110812	15620115	19531118	1	20110812	5
34048561	20120824	16727131	19420220	2	20120824	5
34062958	20111228	16015620	19390815	1	20111228	6
3407192620130123		17190598	19430608	1	20130123	5
34074287	20111105	15862041	19321107	1	20111105	6
34089537	20120703	16567866	19300327	1	20120703	4
34130524	20111216	15985009	19480208	1	20111216	6
3414420220130527		17560821	19530529	1	20130527	5
3416240820130105		17134884	19580810	1	20130105	5
34198599	20121213	17071121	19470401	1	20121213	4
34205851	20120218	16163981	19411208	1	20120218	4
3420956820131010		17997086	19380102	2	20131010	5
34219835	20111025	15826331	19620522	1	20111025	4
34278847	20110330	15219187	19600120	2	20110330	4
34305481	20120417	16340613	19410708	2	20120417	5
34355210	20120729	16645537	19690725	2	20120729	5
34377861	20120506	16394973	19490706	1	20120506	5
34399343	20121021	16899203	19610225	2	20121021	4
34403899	20110629	15482900	19460525	2	20110629	6
34420332	20120312	16233269	19631022	2	20120312	4
34435182	20110816	15629447	19240412	2	20110816	5
34444694	20110919	15721848	19611019	1	20110919	4
3445100820130708		17697427	19731011	1	20130708	6
34488396	20111016	15800886	19640224	1	20111016	6
3450843520130409		17416938	19381225	2	20130409	5
34524157	20121014	16878220	19600417	1	20121014	4
3454335620130218		17253893	19670420	1	20130218	5
3461066720130217		17250500	19540301	2	20130217	4
34681511	20120204	16119193	19490510	1	20120204	5
34734015	20111019	15810467	19460821	2	20111019	6
34741690	20120704	16572800	19570202	2	20120704	5
34792568	20121213	17070608	19270606	1	20121213	4
34803015	20111227	16010444	19431204	1	20111227	4
34812629	20120520	16437190	19700805	1	20120520	5
34851382	20110624	15472350	19600628	1	20110624	5
3497842220130214		17245438	19350101	2	20130214	4
3501569120130202		17220002	19580528	1	20130202	5
3505405820130206		17235260	19421007	1	20130206	4
35132026	20111006	15775833	19500219	1	20111006	6
3513963020131029		18050710	19430612	1	20131029	6
35173901	20120129	16094761	19350622	1	20120129	4
3517933020130414		17431657	19600426	2	20130414	5
3521295420130412		17429276	19460514	1	20130412	6
35245455	20110415	15269926	19600815	1	20110415	6
3527303120131031		18057737	19750915	2	20131031	4
35285031	20120108	16047388	19690903	1	20120108	4
35343363	20120921	16811625	19460301	1	20120921	5
35354779	20121007	16858167	19530125	1	20121007	5
35379636	20111023	15820597	19450114	1	20111023	4

35386528	20111129	15927918	19630127	1	20111129	6
35409735	20110925	15738488	19661108	1	20110925	5
35434174	20110729	15573447	19470508	1	20110729	6
35434812	20120613	16511662	19520314	1	20120613	5
35451402	20121204	17036554	19740329	2	20121204	4
35452165	20120213	16146972	19360820	2	20120213	4
35519621	20111206	15952254	19590819	2	20111206	5
35520060	20110324	15205851	19500402	2	20110324	4
3552637520130715		17717671	19461203	2	20130715	4
3562560620131111		18097628	19290313	2	20131111	5
35652676	20110224	15119587	19441117	2	20110224	5
35724095	20121114	16974285	19651013	2	20121114	4
35729896	20110812	15620130	19600705	2	20110812	4
35731716	20111104	15860034	19300920	2	20111104	4
35760691	20111108	15868977	19760326	2	20111108	6
35766939	20120802	16657699	19340301	2	20120802	4
3576727220131211		18199175	19460315	2	20131211	5
3577418620131022		18033537	19541216	2	20131022	4
35780133	20110530	15392931	19480830	2	20110530	4
35787292	20110407	15239059	19441005	1	20110407	6
35823484	20120528	16457474	19630629	1	20120528	5
35940251	20110321	15196171	19640711	2	20110321	5
3595770120130322		17363599	19591202	1	20130322	4
35959810	20121214	17074978	19471110	1	20121214	4
35981205	20110915	15713598	19510723	2	20110915	6
35999576	20110803	15590773	19580303	1	20110803	4
36013599	20120424	16358901	19570409	2	20120424	6
36024289	20120711	16597091	19401122	2	20120711	5
36032209	20120702	16557227	19580903	2	20120702	5
36059195	20110508	15335265	19590105	1	20110508	5
3607386620130705		17691230	19760227	2	20130705	6
3608839820131112		18101404	19570905	2	20131112	6
36099419	20120311	16229483	19370101	2	20120311	4
36118146	20110917	15718757	19690901	1	20110917	4
36150488	20121208	17052807	19651120	1	20121208	5
36167461	20110911	15701958	19610902	2	20110911	6
36170157	20110301	15134532	19550126	1	20110301	5
36186342	20110511	15347872	19640523	2	20110511	5
3618901220130514		17527958	19500518	1	20130514	5
36276570	20110406	15237630	19400831	2	20110406	6
36286472	20120820	16714884	19670914	1	20120820	4
36323601	20121022	16901709	19670603	1	20121022	4
36323758	20120906	16766881	19341201	2	20120906	4
36325947	20110314	15176618	19840305	1	20110314	6
36345810	20110718	15541723	19560510	1	20110718	6
3635841520130313		17335294	20000915	2	20130313	6
36406890	20120707	16583695	19430307	1	20120707	6
36411559	20110507	15334663	19650620	2	20110507	6
36422841	20110831	15666923	19580415	1	20110831	6
36453404	20120824	16727861	19591118	2	20120824	4
36456801	20110306	15150300	19590207	1	20110306	4
36502360	20120514	16421560	19411216	2	20120514	4
36507581	20110409	15249517	19390510	1	20110409	5
3652482020131109		18094594	19650602	1	20131109	5
36542639	20120129	16094605	19500704	2	20120129	4

36543198	20110314	15176664	19520422	1	20110314	4
36555325	20121029	16919281	19380110	1	20121029	5
36593687	20120305	16209596	19540114	1	20120305	5
3659843320130609		17605687	19451022	1	20130609	6
36622103	20120331	16285014	19540910	2	20120331	5
36623479	20111216	15984991	19541110	2	20111216	4
36630361	20120210	16140387	19350525	1	20120210	5
36689846	20110811	15614992	19421230	1	20110811	4
36692587	20110427	15302401	19620215	2	20110427	4
3670611320130811		17806875	19510810	2	20130811	5
36721105	20121216	17077485	19391129	1	20121216	4
36737196	20121113	16971981	19500118	2	20121113	4
36737801	20120323	16266781	19410215	1	20120323	5
36746551	20110905	15683005	19531014	1	20110905	6
36753885	20110327	15210121	19690621	1	20110327	4
3676831720130502		17488994	19371118	1	20130502	5
36778822	20120407	16309242	19460115	2	20120407	6
36791976	20120423	16354849	19400911	1	20120423	6
36796335	20120207	16128809	19610701	2	20120207	5
3679696020130507		17504004	19510228	2	20130507	6
36804929	20110317	15187356	19310219	1	20110317	5
36805659	20120316	16248356	19600529	2	20120316	5
36820050	20120223	16176812	19710504	1	20120223	4
36840423	20110407	15240533	20060412	1	20110407	6
36842736	20110524	15382297	19660424	1	20110524	5
36907832	20110621	15463070	19730802	2	20110621	5
3691275120130924		17942022	19701029	1	20130924	4
3692461520130104		17132700	19591110	2	20130104	4
3693558720130428		17471943	19460317	2	20130428	5
36939432	20111216	15983974	19571210	1	20111216	4
36986035	20120606	16489474	19650327	1	20120606	5
36988553	20120831	16744055	19620325	2	20120831	5
3699081520130501		17484150	19560730	1	20130501	6
36994500	20110406	15237993	19310910	1	20110406	5
3702195720130609		17605744	19660802	2	20130609	5
37063120	20110312	15172115	19530315	1	20110312	5
37064452	20120826	16729825	19480520	1	20120826	5
3706639020130303		17296908	19530524	1	20130303	5
37076703	20110323	15202661	19520515	2	20110323	5
37103278	20111124	15916869	19360606	2	20111124	6
37106460	20120220	16168147	19531212	2	20120220	4
3715978720130804		17781082	19580719	1	20130804	5
3718710320130429		17475086	19680706	1	20130429	6
37197710	20110314	15175845	19600602	2	20110314	5
37225062	20111003	15760940	19580610	1	20111003	5
37250876	20110327	15210442	19570309	1	20110327	4
37260029	20111115	15891011	19861001	2	20111115	5
37265035	20120813	16695048	19421226	1	20120813	6
3727258520130210		17242403	19810411	1	20130210	5
37272676	20121104	16938540	19361125	2	20121104	4
37273135	20110516	15359228	19451119	1	20110516	5
37292812	20110903	15678451	19470608	1	20110903	6
37324917	20110526	15387769	19510613	2	20110526	4
37332095	20111107	15866253	19430104	2	20111107	6
37346284	20110807	15600603	19500828	1	20110807	4

37347221	20120309	16226890	19381120	2	20120309	6
37355207	20110617	15453436	19660311	2	20110617	6
37358773	20110909	15699848	19500301	2	20110909	5
37372853	20110327	15210399	19471010	1	20110327	4
37373210	20111206	15949120	19271024	2	20111206	6
3737891920130924		17941684	19490402	2	20130924	5
37385094	20111011	15787199	19510404	1	20111011	5
37396524	20120830	16741972	19440103	1	20120830	5
3740296320130226		17280812	19530901	1	20130226	4
37407435	20110608	15424506	19580128	2	20110608	4
3742061420130525		17559773	19540423	2	20130525	5
3743401820130825		17847171	19550901	1	20130825	5
3743721120130611		17613946	19660410	1	20130611	5
37459908	20110326	15209814	19650608	1	20110326	5
37474161	20120915	16793440	19540808	1	20120915	4
37474843	20120121	16087402	19570303	2	20120121	5
37481837	20110429	15307444	19430221	2	20110429	5
37483764	20111207	15957201	19520812	2	20111207	6
37489284	20120126	16090886	19600226	2	20120126	6
3751945220130823		17842913	19450811	1	20130823	5
37538082	20120116	16073560	19590320	2	20120116	4
37574542	20120811	16689950	19431118	2	20120811	6
37577961	20120731	16651367	19460718	2	20120731	5
37578986	20110904	15678997	19391003	1	20110904	4
37580215	20110315	15179216	19680614	1	20110315	6
37587501	20111222	15998802	19591012	1	20111222	6
37587590	20111219	15990978	19881118	1	20111219	4
37597630	20111221	15996805	19500226	2	20111221	6
37612218	20110609	15428826	19251213	1	20110609	4
3761235420130430		17476423	19620308	1	20130430	5
37621184	20121104	16938708	19510711	2	20121104	6
37657424	20110604	15413508	19480216	1	20110604	6
3766127120130814		17818421	19550122	1	20130814	6
37664667	20121010	16867898	19560827	2	20121010	6
3768451820130901		17864997	19640417	2	20130901	4
3770629920131217		18215355	19480201	2	20131217	6
37708080	20110410	15250006	19830430	2	20110410	5
37709209	20120506	16394966	19640922	1	20120506	6
37710331	20110502	15315312	19610803	1	20110502	5
37714480	20110908	15696501	19480529	1	20110908	5
37719032	20110509	15339510	19580310	2	20110509	4
37727278	20120106	16044435	19450701	1	20120106	6
37736235	20111114	15885263	19740205	1	20111114	6
37743194	20120815	16700666	19631006	1	20120815	5
37743423	20111020	15813265	19700202	1	20111020	5
37769452	20110615	15446668	19591120	1	20110615	4
3777028820131128		18151041	19410125	1	20131128	5
37781116	20120410	16319779	19540312	1	20120410	6
37790399	20120501	16379175	19541005	1	20120501	5
3779268020131011		18000748	19651025	2	20131011	4
37793241	20120926	16822892	19680801	2	20120926	6
37800638	20111217	15986634	19321218	1	20111217	4
37804152	20110607	15418077	19590401	1	20110607	6
37825766	20120726	16640620	19520315	1	20120726	5
37832352	20110323	15202834	19421223	1	20110323	6

37833913	20130531	17575093	19580417	1	20130531	5
37835420	20110420	15284384	19540108	2	20110420	5
37850934	20111214	15977255	19570406	1	20111214	5
37854992	20120216	16158607	19390912	1	20120216	4
37857775	20110318	15190437	19750708	2	20110318	5
37869797	20110322	15198156	19981102	1	20110322	6
37874070	20121210	17055925	19591228	1	20121210	6
37892765	20110518	15367143	19440502	1	20110518	5
37899288	20130224	17274137	19470916	1	20130224	4
37900928	20130324	17366022	19520728	1	20130324	4
37902060	20121105	16942629	19740618	2	20121105	6
37907065	20120725	16636167	19380925	1	20120725	6
37909470	20110412	15257949	19620914	2	20110412	6
37912713	20110330	15218904	19740717	1	20110330	5
37922853	20110607	15418581	19570722	1	20110607	4
37926684	20111114	15887411	19590520	1	20111114	5
37931376	20111205	15947623	19450512	1	20111205	4
37942102	20131119	18125897	19361005	1	20131119	5
37943547	20110413	15261367	19551107	1	20110413	6
37946068	20110627	15476053	19620108	1	20110627	6
37954282	20110711	15520615	19610625	2	20110711	5
37954862	20120213	16146308	19731011	2	20120213	6
37973425	20121223	17097602	19520902	1	20121223	6
37980851	20130415	17434547	19630820	1	20130415	4
37989110	20111111	15880871	19610416	1	20111111	5
37995112	20110315	15180170	19720903	1	20110315	6
38002107	20120510	16412111	19590427	1	20120510	5
38012270	20110323	15200310	19770623	1	20110323	5
38017399	20110314	15176802	19520725	1	20110314	6
38024496	20111018	15807994	19630924	1	20111018	5
38032698	20110301	15134273	19681010	2	20110301	6
38045191	20110410	15249715	19560818	1	20110410	4
38046730	20110311	15170472	19920219	1	20110311	6
38048134	20110418	15275648	19940417	2	20110418	6
38051659	20110527	15390511	19490712	1	20110527	5
38054385	20111128	15924667	19690217	1	20111128	6
38065042	20111211	15966419	19280725	2	20111211	6
38074372	20120921	16811524	19721017	1	20120921	4
38077519	20110428	15305257	19691014	2	20110428	5
38096774	20110428	15305247	19530106	1	20110428	5
38117838	20120328	16277467	19411228	2	20120328	5
38121903	20120807	16675456	19750615	1	20120807	5
38133425	20111017	15804529	19351009	2	20111017	4
38133958	20110604	15413945	19631111	2	20110604	5
38134155	20110809	15608939	19570205	1	20110809	6
38137961	20110616	15450526	19510711	1	20110616	4
38148593	20111214	15978239	19490415	2	20111214	4
38148628	20110419	15280349	19510515	1	20110419	6
38149029	20120706	16580602	19590322	2	20120706	6
38157969	20120201	16107881	19511014	1	20120201	6
38179485	20120113	16066201	19510724	2	20120113	6
38187701	20121106	16947731	19410418	2	20121106	6
38189627	20110408	15246334	19360604	2	20110408	4
38196360	20110506	15332473	19560528	2	20110506	4
38197330	20110904	15679004	19490226	2	20110904	4

38212098	20120809	16684511	19700126	1	20120809	5
38230885	20110517	15362166	19821108	2	20110517	6
38241291	20110916	15716888	19390712	1	20110916	4
38245715	20110616	15449549	19551213	1	20110616	6
38247357	20111118	15899972	19610412	1	20111118	4
38262338	20110914	15710183	19841120	1	20110914	5
38262601	20110519	15370596	19390408	2	20110519	6
38262985	20110914	15710729	19540815	1	20110914	5
38269146	20110728	15570538	19900308	2	20110728	5
38274827	20120224	16178633	19551021	1	20120224	5
38285391	20130603	17584151	19520827	1	20130603	6
38288685	20110626	15473598	19320503	1	20110626	5
38291984	20110503	15318353	19640102	1	20110503	6
38298236	20110812	15620106	19460925	2	20110812	4
38300979	20120407	16310423	19611122	2	20120407	5
38308280	20110718	15541555	19320211	1	20110718	4
38309261	20120507	16399414	19520805	2	20120507	6
38311852	20110814	15622189	19550827	1	20110814	6
38315058	20130508	17509519	19620916	2	20130508	6
38316539	20121130	17020409	19420506	2	20121130	4
38320897	20111102	15851753	19420214	1	20111102	5
38340088	20110630	15485960	19510727	2	20110630	5
38345072	20120718	16618086	19720217	1	20120718	4
38347987	20110529	15392292	19480710	2	20110529	6
38350571	20120214	16152018	19620325	1	20120214	6
38361103	20130402	17396006	19830216	2	20130402	5
38367963	20111002	15756574	19760219	2	20111002	5
38375643	20110818	15635400	19730224	1	20110818	5
38381543	20130521	17545124	19480102	2	20130521	5
38386593	20111007	15780179	19540816	1	20111007	5
38386695	20111229	16017646	19661002	1	20111229	6
38387734	20110917	15718799	19500113	1	20110917	5
38403086	20120313	16237589	19540129	2	20120313	4
38405173	20110829	15661454	19351028	2	20110829	6
38415199	20111130	15930648	19490402	1	20111130	4
38416272	20110828	15658814	19760715	1	20110828	4
38429991	20130421	17453310	19310207	2	20130421	4
38435755	20130522	17550477	19221112	2	20130522	6
38451035	20120325	16268688	19310708	1	20120325	4
38465951	20111029	15837542	19610404	1	20111029	5
38470370	20110924	15737942	19650501	1	20110924	5
38485766	20120417	16340589	19411228	2	20120417	6
38486816	20131103	18066345	19830218	1	20131103	4
38487217	20111222	16001064	19481205	2	20111222	4
38487239	20110905	15683170	19641208	1	20110905	6
38489962	20130129	17206515	19260725	1	20130129	4
38499853	20130520	17543493	19330815	1	20130520	4
38504595	20111104	15860287	19300515	1	20111104	4
38506717	20130415	17433968	19270823	1	20130415	6
38507743	20111020	15814331	19810515	2	20111020	5
38523103	20120407	16308542	19560702	1	20120407	6
38524695	20111128	15924953	19661216	1	20111128	4
38529349	20130202	17220039	19580624	1	20130202	6
38540975	20110815	15625251	19590628	2	20110815	6
38545107	20111010	15783017	19591101	1	20111010	4

38546360	20111105	15861821	19481018	1	20111105	5
38556160	20111107	15865011	19491001	1	20111107	5
38561170	20111112	15882803	19290915	1	20111112	4
38562128	20111003	15761755	19541002	1	20111003	4
38563267	20111021	15818394	19611126	1	20111021	6
38570513	20111125	15918636	19500926	1	20111125	5
38572906	20111019	15812099	19331202	1	20111019	4
38580982	20111122	15910636	19770318	2	20111122	4
38584122	20120621	16534105	19620825	2	20120621	4
38586640	20120423	16355060	19740501	1	20120423	4
38595038	20111122	15909254	19340127	2	20111122	4
38616692	20111205	15947136	19330507	2	20111205	6
38618132	20120525	16452014	19270405	1	20120525	5
38619806	20111020	15815353	19510712	1	20111020	4
38621099	20121110	16962956	19240627	1	20121110	5
38621237	20120214	16151841	19270824	1	20120214	6
3862524020130129		17206583	19450610	1	20130129	6
38626210	20120704	16572821	19510728	2	20120704	5
38630238	20111110	15877281	19320320	1	20111110	5
38631811	20120419	16347129	19540925	1	20120419	6
3864206720130715		17720333	19271105	1	20130715	6
38645077	20111017	15804616	19681009	1	20111017	6
38651988	20120208	16132831	19400402	1	20120208	4
38656267	20120213	16147589	20000117	1	20120213	5
38656643	20120104	16037197	19650211	2	20120104	5
38656870	20120115	16069774	19660808	2	20120115	5
38660912	20120510	16411406	19800718	2	20120510	5
38660945	20120704	16573241	19671212	2	20120704	4
38661380	20120828	16735725	19490325	1	20120828	4
38661824	20120505	16394631	19491004	1	20120505	5
38663604	20120316	16248452	19440225	1	20120316	5
38673528	20111115	15887480	19570210	2	20111115	4
38681151	20111021	15818271	19510115	2	20111021	6
38684569	20111117	15898216	19511212	2	20111117	5
38688787	20120408	16310838	19650115	1	20120408	5
3869311720131107		18086087	19550320	2	20131107	6
38695737	20120130	16098104	19391216	1	20120130	4
38697200	20111006	15775873	19550930	1	20111006	6
38701869	20120311	16229675	19351001	1	20120311	4
38703605	20120507	16398494	19321107	1	20120507	5
3870515620130607		17603166	19360510	1	20130607	6
38710417	20111130	15931120	19550309	2	20111130	5
38722622	20120110	16055908	19640705	1	20120110	4
38751289	20120706	16581129	19520815	1	20120706	4
38756717	20111028	15835565	19571203	2	20111028	6
38760202	20120526	16454182	19570204	2	20120526	4
38760713	20121222	17096992	19640529	2	20121222	6
38760928	20121216	17077465	19320126	2	20121216	5
38762786	20120306	16209668	19541011	1	20120306	5
38765423	20111125	15919733	19540826	2	20111125	5
38765956	20120125	16090021	19620401	1	20120125	4
38769107	20120327	16275245	19491206	1	20120327	5
38770240	20120104	16036431	19520102	2	20120104	5
38772779	20111228	16015669	19820627	2	20111228	5
38774208	20120227	16185577	19650907	1	20120227	4

38775096	20131030	18054719	19331205	2	20131030	5
38779963	20120613	16510759	19590507	1	20120613	4
38801422	20120209	16135839	19610427	1	20120209	5
38811379	20120424	16358698	19751222	2	20120424	4
38812827	20111228	16015628	19621015	1	20111228	6
38815199	20111207	15956656	19361029	1	20111207	6
38815780	20120324	16268181	19460302	1	20120324	5
38818165	20121218	17085053	19470702	1	20121218	6
38827677	20130826	17849795	19631114	1	20130826	5
38841371	20130429	17474430	19440301	2	20130429	4
38849853	20120102	16027341	19651216	1	20120102	6
38855297	20120228	16187220	19510618	2	20120228	5
38868052	20120710	16591992	19530420	2	20120710	5
38871942	20120809	16682455	19490625	1	20120809	5
38875580	20120808	16679580	19590724	1	20120808	4
38877111	20120126	16090554	19460201	2	20120126	5
38879468	20120320	16257045	19410419	1	20120320	6
38885186	20120618	16524777	19611126	2	20120618	4
38886918	20121223	17097668	19330215	2	20121223	5
38892498	20120401	16285197	19511202	1	20120401	6
38892852	20120310	16229158	19620211	1	20120310	6
38899739	20131205	18178276	19720323	1	20131205	6
38913472	20120320	16257631	19510708	2	20120320	4
38919129	20120428	16368255	19360610	1	20120428	5
38922735	20120130	16096324	19430615	2	20120130	6
38929918	20120429	16369684	19530425	1	20120429	4
38930697	20131226	18241319	19810806	1	20131226	6
38937518	20120411	16324308	19440813	1	20120411	4
38937972	20120225	16181628	19610908	1	20120225	4
38941309	20120314	16240636	19520926	2	20120314	5
38943327	20120906	16766335	19481120	1	20120906	6
38943725	20120308	16222819	19550219	1	20120308	4
38952124	20121217	17080805	19650921	1	20121217	4
38964679	20121023	16903176	19781029	1	20121023	5
38967123	20120324	16268220	19670114	2	20120324	6
38974651	20120330	16283812	19530710	1	20120330	4
38981407	20120415	16333571	19650629	2	20120415	4
38986968	20120521	16440291	19400608	1	20120521	5
38999563	20121009	16865569	19610512	1	20121009	4
39012196	20120530	16463710	19850713	2	20120530	4
39013053	20130519	17541086	19530727	1	20130519	6
39024196	20120916	16794432	19441129	1	20120916	5
39037019	20120619	16526779	19720502	1	20120619	4
39037359	20120403	16294709	19830905	2	20120403	6
39041877	20120802	16657293	19331205	1	20120802	5
39045197	20120316	16247642	19370114	1	20120316	4
39048630	20130501	17483991	19730911	1	20130501	5
39061648	20120504	16392655	19500130	2	20120504	4
39066041	20121010	16867973	19371005	2	20121010	4
39066303	20120329	16279915	19611225	2	20120329	6
39067793	20120613	16511553	19691112	1	20120613	5
39072601	20120824	16727729	19711206	1	20120824	6
39077004	20120615	16518697	19640902	1	20120615	5
39079317	20120615	16518335	19550720	1	20120615	5
39094525	20120629	16552227	20090626	1	20120629	4

39097182	20120513	16417700	19350509	1	20120513	4
39101796	20120604	16479737	19501126	1	20120604	4
39111085	20120512	16416588	19470228	1	20120512	5
39133136	20120606	16489535	19371108	1	20120606	6
39137398	20120726	16640165	19520825	1	20120726	6
39149285	20120701	16556809	19660111	1	20120701	4
39151207	20120509	16406977	19641224	2	20120509	6
39152982	20130111	17157071	19370217	1	20130111	6
39159698	20120607	16492328	19450831	1	20120607	6
39162793	20120713	16604512	19560719	1	20120713	4
39163230	20120810	16688278	19370921	2	20120810	6
39163821	20120708	16584036	19350525	1	20120708	6
39178693	20120507	16396681	19550415	1	20120507	4
39181276	20120921	16811523	19361129	1	20120921	4
39188777	20120715	16606853	19550908	1	20120715	5
39204290	20130222	17271769	19370815	1	20130222	5
39205748	20120712	16600749	19630903	1	20120712	6
39212130	20120904	16755920	19551013	2	20120904	6
39214089	20120622	16537041	19610503	1	20120622	5
39216029	20120707	16583820	19430205	1	20120707	5
39218036	20130903	17876238	19530702	1	20130903	5
39238205	20121006	16857921	19520320	2	20121006	5
39250389	20120826	16729853	19570228	1	20120826	6
39254790	20130602	17578420	20040630	2	20130602	6
39265480	20120716	16610282	19500502	1	20120716	4
39267840	20120814	16699142	20110514	2	20120814	6
39287166	20120910	16776421	19490613	2	20120910	6
39319534	20130622	17646651	19670728	1	20130622	6
39325887	20120916	16794425	19830818	2	20120916	5
39330251	20120805	16664936	19711231	1	20120805	6
39342693	20121030	16921581	19420204	1	20121030	5
39350408	20130620	17641365	19641206	1	20130620	4
39356688	20130518	17540588	19520102	1	20130518	6
39368564	20120926	16822890	19381203	2	20120926	5
39373290	20130802	17778206	19410507	1	20130802	4
39381925	20120825	16727928	19910411	1	20120825	5
39386077	20120920	16809378	19651204	2	20120920	5
39388095	20121031	16926057	19690110	1	20121031	5
39389827	20130218	17253427	19540603	2	20130218	5
39393607	20130623	17647209	19790428	1	20130623	5
39400890	20121130	17022194	19551221	1	20121130	4
39402603	20121001	16837523	19680613	1	20121001	4
39403093	20121203	17027449	19610815	1	20121203	5
39425917	20120918	16800683	19711106	2	20120918	5
39435331	20120916	16794374	19591020	1	20120916	5
39435977	20121101	16929806	19510306	1	20121101	4
39451280	20121102	16936133	19610915	2	20121102	6
39476909	20121005	16854972	19780917	1	20121005	5
39477935	20130627	17660157	19410810	2	20130627	4
39481828	20121014	16878108	19610425	1	20121014	5
39489822	20130202	17219995	19600213	1	20130202	5
39491377	20121015	16881896	19560310	1	20121015	5
39496032	20121002	16841364	19490821	1	20121002	5
39511303	20130114	17163943	19651011	1	20130114	4
39511814	20121021	16899308	19661207	2	20121021	5

39517038	20121114	16975192	19620311	2	20121114	5
39518871	20121216	17077523	19530520	1	20121216	5
39519852	20121121	16997753	19520612	1	20121121	4
39541178	20130115	17166109	19810124	1	20130115	5
39541305	20121115	16979909	19970728	1	20121115	4
39566502	20130123	17191641	19451025	1	20130123	6
39570246	20121120	16994214	19361118	2	20121120	4
39572457	20130224	17274299	19530516	2	20130224	4
39574179	20121129	17019592	19590621	1	20121129	4
39578159	20121231	17116948	19650816	1	20121231	5
39592977	20121105	16943198	19911110	1	20121105	6
39593210	20121115	16979675	19570505	1	20121115	4
39596413	20121121	16997803	19491015	1	20121121	5
39606150	20131006	17981916	19481215	1	20131006	4
39614385	20121216	17077467	19510214	1	20121216	5
39638341	20130310	17324306	19690130	1	20130310	5
39647080	20130113	17159625	19320323	2	20130113	5
39647751	20130102	17123189	19550302	1	20130102	4
39648685	20130218	17255288	19730321	1	20130218	6
39653899	20130218	17253602	19541120	1	20130218	6
39655157	20121217	17080149	19621215	1	20121217	4
39657993	20130307	17317372	19321113	1	20130307	6
39659988	20130311	17326413	19570405	1	20130311	4
39659999	20121217	17081463	19490409	2	20121217	5
39692916	20130823	17845202	19991111	2	20130823	6
39698174	20130115	17168147	19731010	1	20130115	4
39699804	20130615	17625050	19610710	1	20130615	4
39729754	20130119	17180126	19520915	1	20130119	4
39736602	20130314	17339825	19590710	1	20130314	4
39772128	20130319	17353041	19340627	1	20130319	4
39793367	20130429	17474055	19490925	1	20130429	5
39794133	20130525	17559943	19540303	2	20130525	6
39795976	20130225	17278328	19530120	1	20130225	4
39820627	20130304	17302614	19761208	1	20130304	6
39842836	20130215	17248751	19600707	1	20130215	5
39846292	20130429	17474058	19370606	1	20130429	5
39868150	20130226	17281571	19610816	1	20130226	6
39877640	20130225	17276248	19360425	1	20130225	5
39888103	20130213	17243841	19380414	1	20130213	6
39892154	20130310	17324178	19490114	1	20130310	5
39892767	20130320	17356362	19600903	1	20130320	6
39906304	20130411	17425666	19940702	2	20130411	4
39906893	20130806	17790435	19550102	1	20130806	6
39913218	20130422	17456896	19641130	2	20130422	4
39918417	20130319	17352659	19950322	2	20130319	4
39918940	20130402	17393836	19521108	1	20130402	5
39926471	20131021	18027174	19421008	1	20131021	5
39928240	20130606	17597644	19630525	2	20130606	5
39931710	20130422	17456756	19710906	1	20130422	4
39937912	20130430	17478405	19590628	2	20130430	4
39938573	20131022	18032208	19591206	2	20131022	6
39940573	20130814	17819505	20070726	1	20130814	5
39969498	20130430	17477649	19480915	1	20130430	6
39972775	20130430	17477192	19760927	1	20130430	5
39997836	20130512	17519403	19621020	1	20130512	6

4001096620130505	17496020	19730328	1	20130505	6
4001193620130527	17563991	19880207	2	20130527	4
4001554120130808	17800457	19660820	2	20130808	4
4001690820130926	17948693	19400501	1	20130926	5
4001742320131130	18155994	19601222	2	20131130	6
4001886820130527	17563294	19590302	2	20130527	6
4001928120130603	17584405	19480528	1	20130603	6
4002042620131204	18172499	19470121	1	20131204	4
4002394720130811	17806712	19470902	1	20130811	6
4004150720130911	17906220	19631130	1	20130911	6
4004597420130811	17806758	19480817	2	20130811	5
4004986320131004	17979755	19471022	1	20131004	5
4005243520130808	17798936	19560526	1	20130808	5
4005531020130417	17444132	19731205	2	20130417	5
4005551420131013	18003235	19440803	1	20131013	4
4005586520130609	17605565	19730112	1	20130609	5
4005708720130624	17650870	19570803	1	20130624	5
4006191420130529	17568142	19360317	1	20130529	6
4006549420130826	17850983	19641029	2	20130826	5
4006951020130708	17698608	19670623	1	20130708	5
4008555020130624	17649916	19720626	2	20130624	4
4008624620130711	17711796	19501018	2	20130711	5
4013351520131223	18231896	19730701	1	20131223	6
4013851020130901	17864902	19490711	2	20130901	6
4014175120130612	17614879	19570805	1	20130612	6
4015205420130707	17693567	19541225	1	20130707	5
4015370620130612	17615175	19631124	1	20130612	4
4017439820130703	17681176	19560423	1	20130703	6
4018553320130627	17660822	19780829	2	20130627	5
4019033820130831	17864728	19660505	1	20130831	4
4019691620130811	17806560	19521220	1	20130811	5
4020112520130718	17731342	19650620	1	20130718	5
4021816420130822	17841439	19930730	2	20130822	6
4022945620130807	17795112	19670425	1	20130807	4
4023321420131217	18215357	19470119	2	20131217	6
4023557220130721	17738313	19611031	1	20130721	5
4023891320130905	17884614	19480101	1	20130905	5
4024041320130818	17829301	19670106	1	20130818	5
4024613720130708	17696809	19611208	1	20130708	6
4026938320131017	18019217	19570210	1	20131017	5
4027331220131112	18104510	19580325	1	20131112	6
4027363020130724	17749363	19901205	1	20130724	6
4027392520130822	17839113	19630324	1	20130822	5
4027825920130828	17856534	19531010	2	20130828	4
4027827120130903	17877263	19770825	1	20130903	4
4029240820131002	17969516	19470703	2	20131002	5
4029652420130924	17942109	19590109	2	20130924	5
4029912520130921	17933581	19901001	1	20130921	6
4030726420130802	17774527	19661214	1	20130802	5
4030794620130819	17831632	19601204	1	20130819	5
4031253620130829	17860151	19450701	2	20130829	4
4038078720131118	18120308	19821228	2	20131118	5
4040477120131217	18215076	19371212	1	20131217	5
4041737620130909	17897023	19431128	1	20130909	4
4042540920130825	17847594	19740225	1	20130825	5

4045891120131120	18129595	19531220	1	20131120	5
4046280420130920	17932205	19520324	2	20130920	6
4047052820131015	18012209	19770923	1	20131015	5
4047462220131009	17994658	19610202	1	20131009	5
4048235720131015	18009764	19540910	1	20131015	4
4048504920131029	18052388	19740224	1	20131029	4
4050469620131117	18118400	19790811	2	20131117	5
4051766620130916	17919593	19620910	1	20130916	5
4052400320130921	17933327	19470610	1	20130921	4
4053963720131119	18125836	19550121	2	20131119	5
4054038320131110	18094878	19720718	1	20131110	5
4061341020131127	18149015	19570620	1	20131127	4
4062547620131129	18153783	19340623	2	20131129	6
4063902920131217	18213043	19721105	1	20131217	6
4067616220131122	18135806	19450902	1	20131122	6
4069713020131114	18112373	19520805	1	20131114	6
4072063620131231	18251183	19610301	1	20131231	6
4074675620131208	18185412	19310514	1	20131208	5
4086347620131217	18216321	19410303	1	20131217	6
18438320131001	17963374	19250512	1	20131001	5
204308 20120906	16765954	19330721	2	20120906	4
36133120131214	18208137	19420219	2	20131214	5
424237 20120410	16320044	19290906	2	20120410	5
429152 20120305	16207043	19301224	1	20120305	6
429685 20120910	16774392	19270604	1	20120910	6
66029720130912	17911643	19320617	1	20130912	6
1166923 20121110	16962476	19341115	2	20121110	4
117888720130527	17563967	19670525	2	20130527	4
1369706 20120219	16164662	19270728	2	20120219	6
142484620131211	18199119	19470628	2	20131211	6
1492315 20120721	16626251	19360705	1	20120721	5
1556861 20120505	16393478	19330614	1	20120505	4
1852706 20110429	15307618	19320728	1	20110429	5
198492520130523	17554825	19190712	1	20130523	4
205249520130204	17225858	19330107	1	20130204	6
2251947 20111017	15802863	19491107	2	20111017	6
2327620 20120214	16150643	19260929	1	20120214	5
260547220130902	17868610	19390818	1	20130902	5
3232006 20121123	17003500	19381228	2	20121123	5
332357720131021	18028543	19460615	1	20131021	5
3594523 20120306	16210724	19320920	1	20120306	6
3696911 20120211	16142958	19370220	1	20120211	6
3854668 20110630	15485112	19280901	1	20110630	6
3965853 20120626	16543985	19300303	1	20120626	5
4074684 20111004	15765931	19410314	2	20111004	4
4137773 20120621	16534116	19221011	1	20120621	6
4458031 20120619	16527066	19540111	1	20120619	6
4525397 20110328	15212910	19291201	1	20110328	6
4684439 20110828	15658883	19290322	1	20110828	6
470202720130113	17159602	19350710	1	20130113	5
4915257 20120730	16648106	19390715	2	20120730	4
498227820130301	17293998	19370112	1	20130301	5
499190620130508	17507939	19401224	1	20130508	5
500336120130428	17471642	19481108	1	20130428	6
5115935 20120515	16425342	19310921	2	20120515	5

5373091	20120823	16725174	19440519	1	20120823	6
5455118	20110914	15709370	19580925	1	20110914	4
5503242	20120628	16552163	19370707	2	20120628	5
5586016	20131001	17964082	19410610	1	20131001	4
5649592	20111010	15783008	19360210	1	20111010	5
6666666	20120323	16266844	19520821	1	20120323	4
6987913	20120531	16465607	19430124	1	20120531	5
7202940	20130217	17250518	19531211	1	20130217	4
7389404	20130721	17738410	19240815	2	20130721	5
7452439	20121124	17006012	19760602	1	20121124	6
7742769	20121215	17076953	19330729	1	20121215	4
7833387	20110526	15388052	19581003	2	20110526	4
7910330	20120316	16248410	19401229	2	20120316	6
8003063	20110902	15673494	19500401	1	20110902	4
8196198	20120823	16725166	19450825	2	20120823	4
8302474	20131126	18144764	19330316	1	20131126	6
8609903	20120712	16601156	19470705	2	20120712	4
8617376	20111203	15942914	19411020	1	20111203	5
8683509	20111214	15976670	19391122	2	20111214	6
8896024	20111006	15776045	19790911	1	20111006	5
8916323	20120719	16621805	19470715	1	20120719	4
9228993	20120101	16022527	19560517	2	20120101	6
9349137	20130514	17526929	19510205	2	20130514	6
9392169	20120715	16606769	19500601	2	20120715	6
9445867	20111122	15910613	19251221	1	20111122	4
9455145	20131229	18245975	19550102	1	20131229	5
9888748	20111012	15790925	19410510	2	20111012	5
9922252	20110816	15627580	19611027	1	20110816	6
9961584	20120712	16601151	19560308	1	20120712	5
9990970	20120223	16177672	19740511	2	20120223	6
10193938	20121129	17018499	19291005	1	20121129	6
10234261	20111206	15952325	19511011	1	20111206	5
10280101	20131216	18211624	19391219	1	20131216	5
10341001	20130906	17891144	19501118	1	20130906	4
10571036	20120717	16613812	19580128	1	20120717	6
10619359	20120501	16377878	19560703	2	20120501	6
10917109	20121030	16922865	19550509	1	20121030	4
10924115	20130427	17471315	19150116	1	20130427	6
11008381	20130303	17296593	19300603	1	20130303	4
11064178	20121017	16889599	19491001	1	20121017	4
11121667	20121228	17113469	19361013	1	20121228	4
12090276	20111209	15964324	19441114	2	20111209	4
12110097	20120127	16091626	19510623	2	20120127	6
12152613	20120418	16342309	19410727	2	20120418	4
12210592	20111103	15855729	19441021	1	20111103	4
12380642	20110830	15663985	19360205	2	20110830	5
12392186	20110819	15639351	19510909	1	20110819	4
12441348	20120309	16227241	19680417	1	20120309	6
12475159	20110520	15373314	19600712	2	20110520	6
12494596	20120226	16182837	19490720	2	20120226	6
12639848	20130912	17911407	19551028	2	20130912	4
12693060	20120320	16256657	19750428	1	20120320	4
12898269	20120412	16328269	19250102	2	20120412	5
12955781	20130624	17650927	19480305	2	20130624	5
12962060	20130915	17917667	19271028	1	20130915	5

12968080	20120105	16040668	19270919	1	20120105	5
13022510	20131213	18206401	19340825	2	20131213	5
13039866	20110516	15360324	19490701	1	20110516	4
13042643	20130722	17741440	19520201	1	20130722	5
13060963	20120123	16088736	19350911	2	20120123	4
13183023	20110918	15719373	19290205	1	20110918	5
13285524	20120429	16369453	19291201	1	20120429	4
13302631	20120716	16610775	19420528	2	20120716	4
13378273	20121115	16978316	19310125	1	20121115	6
13395669	20121213	17069797	19620814	2	20121213	6
13554397	20130712	17714355	19640323	2	20130712	5
13571749	20120822	16722443	19511112	2	20120822	6
13635488	20111214	15978228	19440626	2	20111214	4
13723972	20110630	15485950	19350725	2	20110630	4
13781049	20130511	17518740	19320305	1	20130511	5
13836083	20110415	15270649	19530515	2	20110415	6
14008389	20130103	17127191	19490319	2	20130103	4
14039237	20130506	17500018	19250114	1	20130506	5
14104257	20110514	15356080	19271008	2	20110514	4
14125156	20120820	16714556	19441117	2	20120820	4
14143749	20120603	16474395	19600222	2	20120603	4
14224352	20121230	17115223	19570317	1	20121230	4
14366848	20130207	17237212	19290614	1	20130207	5
14760022	20120515	16423153	19330801	1	20120515	5
14765663	20111016	15800586	19591023	2	20111016	6
14793885	20120103	16032763	19330107	1	20120103	4
14829108	20130917	17924311	19481014	1	20130917	6
14923343	20120814	16696725	19420223	2	20120814	6
14927516	20130527	17563902	19540820	2	20130527	6
15246885	20130616	17625631	19840209	2	20130616	6
15258534	20120813	16694281	19530912	2	20120813	4
15369536	20120530	16463140	19301030	1	20120530	5
15371003	20110909	15699830	19350225	2	20110909	6
15640887	20121126	17009996	19330320	1	20121126	5
15651328	20130731	17766053	19560109	2	20130731	5
15671144	20130821	17837914	19291231	2	20130821	6
15679206	20110927	15744961	19521015	1	20110927	5
15694652	20130725	17752695	19580625	1	20130725	5
15792139	20110731	15575020	19390601	1	20110731	4
15887919	20120223	16177988	19230520	1	20120223	5
16056330	20130212	17243368	19320906	2	20130212	5
16179127	20131007	17985114	19300110	1	20131007	5
16249379	20120727	16643516	19310606	1	20120727	4
16284972	20110928	15748111	19310812	1	20110928	4
16394459	20120502	16384263	19440808	2	20120502	5
16455495	20121005	16854512	19300328	1	20121005	4
16600105	20131221	18227655	19600226	1	20131221	5
16697113	20110909	15699003	19680524	2	20110909	6
16750655	20130326	17373254	19310304	1	20130326	4
16912995	20120617	16520830	19540402	1	20120617	4
16956700	20111227	16012247	19540606	1	20111227	4
16974666	20131126	18143741	19530915	1	20131126	4
17049955	20110930	15753487	19591008	2	20110930	6
17104379	20110311	15170471	19720811	2	20110311	4
17237897	20120525	16452592	19351020	2	20120525	4

17244143	20110829	15661658	19400101	2	20110829	6
17267571	20131022	18033588	19650208	2	20131022	6
17270778	20120731	16651472	19430306	1	20120731	4
17314339	20131026	18045554	19600815	2	20131026	6
17330584	20130731	17766280	19861110	1	20130731	5
17434934	20120819	16711549	19440908	1	20120819	5
17489508	20110612	15434448	19581112	1	20110612	6
17517043	20110410	15249723	19680730	2	20110410	4
17549223	20120409	16315695	19530701	1	20120409	4
17605117	20120819	16711705	19450107	2	20120819	5
17688685	20130706	17693132	19540112	1	20130706	6
17998526	20130703	17682364	19330111	2	20130703	5
18010758	20120729	16645602	19291201	2	20120729	4
18137821	20130701	17671595	19630919	1	20130701	6
18196800	20110729	15571534	19270707	1	20110729	4
18199398	20130408	17412378	19370219	2	20130408	5
18251315	20110612	15434716	19320915	1	20110612	4
18368893	20110630	15485136	19710802	1	20110630	6
18491162	20111208	15960137	19440222	1	20111208	4
18497126	20111120	15903325	19251115	1	20111120	6
18572628	20121001	16836287	19311020	1	20121001	4
18861693	20121013	16877857	19570909	1	20121013	5
18907410	20110918	15719151	19591201	2	20110918	5
18950895	20130217	17250566	19520121	1	20130217	5
18995614	20110423	15292070	19270101	2	20110423	6
19000023	20130925	17944622	19251115	1	20130925	5
19005777	20131209	18189474	19340319	2	20131209	4
19044054	20110819	15639243	19620414	1	20110819	4
19107052	20110513	15354723	19311230	1	20110513	5
19129192	20110514	15356192	19580618	1	20110514	4
19185923	20131217	18213339	19831005	2	20131217	4
19307267	20130205	17230777	19490312	2	20130205	4
19439986	20120504	16392634	19501212	1	20120504	6
19653817	20120810	16687382	19511229	2	20120810	4
19848443	20130629	17665363	19520120	1	20130629	5
19928940	20110829	15660773	19320508	1	20110829	4
19992995	20110420	15282841	19530125	2	20110420	6
20050026	20120813	16695047	19630102	2	20120813	6
20284666	20111217	15986623	19331210	1	20111217	4
20332256	20120918	16802283	19410512	2	20120918	6
20399988	20120925	16821616	19321119	2	20120925	5
20486297	20111206	15952553	19590227	1	20111206	4
20490248	20130205	17229102	19231015	1	20130205	6
20505537	20130916	17919349	19371126	1	20130916	5
20556109	20120514	16420572	19240926	1	20120514	5
20677865	20121102	16935696	19571008	1	20121102	5
20697067	20110909	15698091	19690717	2	20110909	5
20789553	20120924	16818074	19601105	1	20120924	5
21006804	20110503	15317768	19870721	1	20110503	6
21188829	20120728	16645044	19280302	2	20120728	6
21593704	20120702	16562107	19230707	2	20120702	4
21713768	20130505	17496223	19471120	1	20130505	4
21912572	20111202	15940610	19520128	1	20111202	5
21951688	20120131	16101685	19620126	1	20120131	5
22063189	20120317	16250206	19420515	1	20120317	5

22246351	20111026	15830442	19651205	2	20111026	6
22319988	20111016	15800562	19320827	1	20111016	5
22405910	20120623	16537578	19590922	1	20120623	5
22554514	20120503	16388883	19500428	1	20120503	5
22648891	20120529	16460521	19470428	1	20120529	4
22747724	20121103	16938268	19920529	2	20121103	4
22824448	20120807	16675279	19261219	1	20120807	6
22835036	20120602	16473994	19440623	1	20120602	4
22881545	20130922	17934010	19380816	2	20130922	5
22953237	20131121	18132196	19610907	1	20131121	5
23133995	20120323	16264194	19481120	1	20120323	6
23231596	20110921	15730531	19570206	1	20110921	5
23272711	20131007	17984740	19670820	2	20131007	4
23276304	20120120	16084884	19180301	2	20120120	4
23356992	20111004	15765579	19710725	1	20111004	4
23512494	20130309	17323572	19481101	2	20130309	5
23540310	20130911	17907281	19420705	2	20130911	5
23547162	20110526	15388010	19721114	2	20110526	4
23601265	20110406	15237858	19580623	1	20110406	5
23614144	20120904	16757161	19290226	2	20120904	4
23674057	20130619	17637978	19440518	2	20130619	5
23721428	20130630	17665704	19220507	1	20130630	5
23760294	20131226	18240168	19470104	1	20131226	6
23764854	20110415	15270693	19270606	1	20110415	5
23797571	20110504	15324777	19610130	2	20110504	4
23996567	20130827	17854223	19360730	1	20130827	4
24203938	20110821	15641182	19370224	2	20110821	5
24340112	20131002	17969939	19550412	2	20131002	6
24348003	20130523	17554784	19380617	2	20130523	6
24424848	20121214	17075013	19260302	1	20121214	4
24459776	20120203	16117217	19740226	1	20120203	5
24485652	20130104	17132431	19381103	1	20130104	6
24638446	20120709	16588507	19340110	1	20120709	4
24768330	20111226	16008924	19711227	2	20111226	5
24885801	20111015	15800281	19631125	1	20111015	5
25067054	20130710	17706457	19681029	1	20130710	6
25429189	20130416	17439218	19471230	1	20130416	6
25445798	20130606	17596984	19550331	1	20130606	4
25477285	20131128	18151284	19640529	1	20131128	5
25574178	20130228	17287925	19520824	2	20130228	5
25587706	20110430	15308805	19500905	2	20110430	5
25607743	20110923	15735496	19371202	1	20110923	5
25631510	20120426	16364521	19451014	1	20120426	6
25704807	20120623	16537529	19341205	2	20120623	6
25727406	20110423	15292027	19601225	1	20110423	4
25904238	20110519	15369413	19550404	2	20110519	5
25923697	20120902	16746895	19721213	2	20120902	4
25982187	20120726	16639948	19540623	1	20120726	5
26062748	20111008	15782047	19680517	1	20111008	5
26083807	20130905	17883577	19590215	2	20130905	6
26130370	20130105	17134164	19280816	2	20130105	6
26135977	20111024	15824054	19321222	1	20111024	5
26213467	20130221	17268632	19570503	2	20130221	5
26226506	20120322	16263913	19550606	1	20120322	4
26280284	20130319	17352983	19580110	2	20130319	4

26393873	20130812	17810009	19521104	1	20130812	5
26439281	20130310	17324205	19751228	2	20130310	5
26845498	20121213	17069988	19440520	1	20121213	4
26856644	20120606	16489491	19360318	2	20120606	5
26907391	20131215	18208406	19280503	1	20131215	6
26945653	20130813	17815351	19220505	1	20130813	5
27087509	20120215	16155650	19310224	2	20120215	5
27199662	20110523	15378333	19961119	1	20110523	4
27219096	20120504	16392769	19500225	1	20120504	6
27286857	20120326	16271546	19590820	2	20120326	6
27336910	20120827	16732883	19450808	2	20120827	5
27487634	20110430	15308855	19311019	2	20110430	4
27518872	20110907	15691741	19500102	1	20110907	6
27626182	20120110	16055306	19641225	1	20120110	5
27704730	20120531	16465522	19541215	2	20120531	4
27736807	20120125	16089962	19370118	2	20120125	4
27813838	20111101	15846871	19511021	2	20111101	4
27879114	20110831	15666076	19650305	2	20110831	6
27912498	20110919	15722889	19330206	2	20110919	6
27914938	20130407	17407251	19720702	2	20130407	6
27946629	20130816	17827059	19410906	1	20130816	5
27967073	20130807	17795378	19430820	1	20130807	5
28040757	20130613	17619416	19410316	1	20130613	5
28042559	20131115	18115119	19500718	1	20131115	6
28169815	20130708	17696666	19721006	2	20130708	4
28179079	20110621	15462645	19280123	1	20110621	4
28349964	20110508	15335227	19630924	2	20110508	4
28373742	20120831	16744069	19370730	2	20120831	4
28541857	20131203	18167855	19460622	2	20131203	5
28624115	20130911	17907483	19630220	1	20130911	4
28885861	20111206	15952188	19360726	1	20111206	5
29182818	20120829	16739046	19490606	1	20120829	6
29329688	20121128	17015748	19840214	1	20121128	6
29342254	20120524	16449386	19640606	1	20120524	5
29359522	20120602	16474194	19270118	2	20120602	5
29419929	20110822	15644190	19200725	1	20110822	4
29566232	20130718	17731467	19521229	1	20130718	6
29625670	20120814	16699064	19600220	1	20120814	5
29800213	20130929	17954862	19570703	1	20130929	4
29911624	20131107	18088407	19701208	2	20131107	6
29958063	20110526	15387742	19620220	1	20110526	6
29977308	20120504	16392552	19420609	2	20120504	6
30264007	20120111	16058990	19510604	2	20120111	6
30283706	20110310	15167057	19440221	1	20110310	5
30295217	20120525	16451641	19611120	1	20120525	6
30413479	20130808	17798919	19510501	2	20130808	4
30424932	20130216	17250197	19300929	1	20130216	5
30454401	20120618	16523964	19300801	1	20120618	6
30507905	20131116	18116814	19360526	2	20131116	4
30546840	20130722	17742025	19391228	1	20130722	6
31009082	20130702	17677164	19480403	1	20130702	6
31058249	20110327	15210429	19751219	2	20110327	6
31078032	20110808	15604958	19341231	2	20110808	5
31254758	20121107	16952688	19590218	1	20121107	4
31388246	20130908	17893936	19701018	1	20130908	6

31402796	20120607	16491867	19520506	2	20120607	6
31429319	20130519	17541233	19590711	1	20130519	4
31430418	20110806	15600430	19461125	1	20110806	5
31558733	20121222	17097001	19500114	2	20121222	4
31567187	20121226	17107574	19681015	1	20121226	4
31688343	20121112	16967589	19550405	1	20121112	6
31743405	20111222	16000227	19500418	2	20111222	6
31751118	20130112	17158871	19411012	2	20130112	6
31772426	20121024	16906357	19500401	2	20121024	6
31883020	20110502	15314980	19500109	2	20110502	5
31922744	20130911	17907481	19460715	1	20130911	4
32012069	20110425	15296084	19670127	1	20110425	4
32018589	20130429	17475003	19540406	2	20130429	5
32066594	20131014	18006185	19420927	1	20131014	6
32271151	20110726	15563305	19411016	1	20110726	5
32389905	20130417	17444162	19380205	1	20130417	4
32576413	20121121	16997284	19601201	1	20121121	6
32623680	20120830	16740956	19510715	2	20120830	6
32771985	20130117	17173364	19540223	1	20130117	4
32827057	20120317	16250051	19930709	1	20120317	4
32843780	20121218	17084051	19520610	1	20121218	4
32900597	20120907	16768936	19541202	2	20120907	4
33024838	20130401	17388041	19390614	1	20130401	6
33052958	20120813	16693948	19521101	1	20120813	4
33096105	20130611	17614682	19620212	1	20130611	5
33118331	20110311	15170488	19420927	2	20110311	6
33162264	20120504	16392230	19650104	1	20120504	4
33180835	20130804	17781134	19590101	1	20130804	4
33330324	20110607	15419760	19681226	1	20110607	5
33506604	20120201	16106822	20040614	1	20120201	6
33570864	20120615	16516570	19621107	1	20120615	6
33617219	20130902	17869835	19650110	1	20130902	5
33639597	20130322	17363843	19500918	2	20130322	6
33701267	20120911	16781549	19590510	1	20120911	4
33975358	20121113	16971940	19770214	2	20121113	4
34049644	20121110	16963021	19390812	1	20121110	5
34071926	20130218	17251910	19430608	1	20130218	5
34162408	20130217	17250667	19580810	1	20130217	6
34218752	20130719	17734925	19350310	1	20130719	4
34247682	20120919	16805347	19560421	1	20120919	5
34257540	20130310	17324487	19610402	2	20130310	4
34260327	20130904	17879451	19570216	1	20130904	4
34278847	20110412	15255468	19600120	2	20110412	5
34299520	20120719	16621597	19590830	1	20120719	5
34334004	20131204	18171102	19500615	1	20131204	5
34355210	20130531	17575753	19690725	2	20130531	5
34369658	20130424	17462836	19610822	1	20130424	6
34442347	20120224	16180704	19290915	1	20120224	6
34485013	20110623	15469618	19501115	1	20110623	5
34543356	20130712	17714035	19670420	1	20130712	5
34556440	20121008	16861838	19560802	1	20121008	5
34587661	20120220	16168287	19670715	1	20120220	6
34614341	20120831	16744072	19491010	2	20120831	5
34665991	20130705	17691232	19740705	2	20130705	6
34720791	20120812	16690302	19791212	2	20120812	4

34741690	20120718	16614823	19570202	2	20120718	6
34774748	20130119	17180092	19490424	1	20130119	4
34803333	20110418	15276499	19600119	2	20110418	5
34823808	20111111	15880717	19610620	1	20111111	5
34923734	20130420	17452863	19631130	1	20130420	4
35010378	20130318	17349371	19400102	1	20130318	5
35074523	20121104	16938724	19340721	2	20121104	6
35162562	20110708	15516345	19530726	1	20110708	6
35165265	20130129	17206571	19400125	1	20130129	4
35179330	20130512	17519163	19600426	2	20130512	5
35212954	20130506	17500948	19460514	1	20130506	4
35235655	20130502	17486629	19561102	2	20130502	5
35245455	20110506	15332887	19600815	1	20110506	5
35264052	20120724	16633901	19550530	1	20120724	6
35343943	20110419	15280463	19321108	1	20110419	4
35379636	20111216	15984970	19450114	1	20111216	5
35423428	20130515	17531719	19400512	2	20130515	4
35522931	20111110	15878037	19480220	1	20111110	5
35546486	20130430	17477153	19660304	2	20130430	4
35603351	20120121	16087915	19401112	1	20120121	4
35724095	20130605	17592916	19651013	2	20130605	6
35751816	20111127	15921978	19540615	2	20111127	4
35766939	20120828	16736233	19340301	2	20120828	6
35767498	20120117	16076082	19670615	1	20120117	5
35780133	20120613	16508640	19480830	2	20120613	5
35797967	20120219	16164652	19540208	2	20120219	6
35824556	20111221	15998075	19540415	1	20111221	5
35853077	20110420	15284160	19511115	2	20110420	4
35999576	20111022	15820070	19580303	1	20111022	5
36068005	20120614	16513859	20030123	2	20120614	5
36118146	20120201	16107709	19690901	1	20120201	4
36119649	20130509	17513588	19361104	2	20130509	6
36205842	20111224	16005420	19740416	2	20111224	4
36219382	20130330	17383421	19420221	2	20130330	5
36233064	20130121	17183731	19631210	2	20130121	6
36330286	20111208	15959648	19320315	1	20111208	4
36362295	20131011	18000837	19990205	2	20131011	5
36406890	20120719	16621241	19430307	1	20120719	5
36454725	20110330	15219094	19621023	1	20110330	6
36467795	20120131	16101642	19470822	1	20120131	4
36473322	20110316	15183986	19501025	2	20110316	5
36480065	20120209	16137011	19400619	1	20120209	4
36502360	20120606	16489494	19411216	2	20120606	5
36559178	20120205	16119696	19750625	2	20120205	5
36564702	20131113	18107524	19480301	1	20131113	5
36603379	20120227	16185600	19620501	2	20120227	4
36617320	20110517	15362825	19540922	2	20110517	5
36625828	20110325	15208177	19440810	1	20110325	5
36659184	20110818	15636273	19350928	1	20110818	6
36669951	20130606	17599141	19580214	1	20130606	4
36671531	20120324	16268041	19520226	1	20120324	6
36685344	20120917	16798361	19610313	1	20120917	5
36737196	20130206	17235204	19500118	2	20130206	4
36762900	20110809	15608956	19560915	2	20110809	4
36805659	20120518	16434497	19600529	2	20120518	4

36818094	20120421	16351440	19690307	2	20120421	5
3682169920130303		17297051	19660520	1	20130303	5
3689178820131110		18094971	19490904	1	20131110	5
3689653420130506		17500522	19291115	1	20130506	6
36907832	20110803	15590419	19730802	2	20110803	4
36924955	20120123	16088807	19430414	2	20120123	6
36931994	20120325	16268639	19680302	1	20120325	6
36939432	20120120	16085911	19571210	1	20120120	6
36979154	20110901	15669570	19460501	1	20110901	5
37030298	20110825	15654273	19550406	1	20110825	4
37063120	20110401	15226907	19530315	1	20110401	5
3706536420131103		18066857	19551206	1	20131103	4
3707524420130221		17268604	19571018	2	20130221	4
37141676	20120926	16823899	19420116	1	20120926	4
37197710	20110411	15253767	19600602	2	20110411	4
37217791	20121215	17077004	19480310	2	20121215	6
37225062	20111016	15801041	19580610	1	20111016	5
37265875	20110607	15418903	19440821	2	20110607	5
37268954	20110517	15363284	19670331	2	20110517	4
37273135	20110620	15458632	19451119	1	20110620	5
37274241	20111214	15978093	19970405	2	20111214	6
37274718	20110521	15375112	20091219	1	20110521	6
37317854	20120901	16745989	19350927	1	20120901	6
37328942	20110917	15718838	19800807	2	20110917	5
37339256	20110611	15433917	19871128	1	20110611	6
37347221	20120423	16354582	19381120	2	20120423	4
3735259320130623		17647189	19491215	2	20130623	6
37355207	20110817	15631845	19660311	2	20110817	5
37373210	20120112	16063042	19271024	2	20120112	5
37385094	20111108	15870657	19510404	1	20111108	6
3742061420130619		17636699	19540423	2	20130619	5
3742077220130911		17907319	19701112	1	20130911	5
37428732	20120403	16294793	19220206	1	20120403	4
3745713920131014		18006740	19220810	1	20131014	4
37459908	20110503	15320061	19650608	1	20110503	4
37468012	20121109	16960996	19520122	1	20121109	4
37474843	20120222	16175087	19570303	2	20120222	5
37481837	20110526	15386490	19430221	2	20110526	5
37503654	20120219	16164412	19951127	2	20120219	4
37508466	20110731	15574827	19510926	1	20110731	6
37535287	20110527	15390481	19591026	2	20110527	5
37538082	20120221	16171771	19590320	2	20120221	6
37559630	20110513	15352655	19591206	1	20110513	4
37574542	20121003	16845584	19431118	2	20121003	6
37577961	20120811	16689945	19460718	2	20120811	4
37591201	20110314	15176435	19820927	2	20110314	5
3759504320130703		17681701	19430802	1	20130703	6
37623362	20110815	15626142	19511125	1	20110815	6
37669479	20110523	15379198	19490701	1	20110523	6
3767139920130512		17519077	19291202	1	20130512	4
37680878	20110823	15647193	19440809	2	20110823	6
37694614	20120224	16180860	19571212	2	20120224	6
37709209	20120624	16537770	19640922	1	20120624	5
37717058	20120111	16060154	19631120	2	20120111	4
37726764	20110912	15702642	19810722	2	20110912	6

37734773	20110919	15723387	19900806	1	20110919	5
3773960920130203		17220495	19501207	2	20130203	5
37743194	20120906	16764882	19631006	1	20120906	5
37769190	20110220	15106337	19611023	1	20110220	5
37784353	20110504	15322389	19301201	1	20110504	5
37792431	20110415	15270717	19360923	1	20110415	5
37800387	20120725	16637495	19630206	1	20120725	6
3784987920131010		17997276	19490419	2	20131010	4
37854992	20120817	16708592	19390912	1	20120817	5
37857775	20110406	15238817	19750708	2	20110406	6
37858507	20110330	15219134	19541115	2	20110330	4
37872745	20121004	16850605	19430606	1	20121004	4
3787407020130123		17191450	19591228	1	20130123	6
3788476720130123		17191727	19570608	2	20130123	5
37887517	20110806	15600371	19420624	1	20110806	4
37888270	20120307	16218650	19440205	1	20120307	6
3789031620131010		17997189	19360429	1	20131010	5
37896698	20110913	15704096	19570104	1	20110913	4
37909425	20110428	15305136	19620627	2	20110428	5
37923118	20111225	16005602	19360327	2	20111225	4
37927121	20120104	16037236	19500221	2	20120104	5
37941392	20120210	16138946	19450708	1	20120210	6
37949501	20110527	15390530	19541020	1	20110527	5
37950951	20120103	16028827	19610921	2	20120103	6
37954282	20110820	15640610	19610625	2	20110820	4
37969098	20110822	15644755	19421203	2	20110822	4
38006416	20120131	16101419	19520801	1	20120131	4
38013182	20110517	15363631	19810503	1	20110517	5
38025671	20111112	15882832	19310728	1	20111112	6
38048714	20110425	15295325	19660718	1	20110425	5
38050587	20110529	15392042	19740210	1	20110529	5
38071226	20111031	15840802	19570220	1	20111031	5
38077713	20110425	15295404	19580518	2	20110425	5
3808267620130727		17757379	19271116	2	20130727	6
38110417	20110503	15317375	19440702	1	20110503	6
38114782	20111012	15791458	19460808	1	20111012	6
38118842	20110929	15750854	19550919	2	20110929	5
38128904	20110606	15415057	19600822	1	20110606	6
38133072	20110623	15468881	19570212	1	20110623	4
38139763	20120617	16520978	19550128	1	20120617	5
38145196	20110623	15468099	19680614	1	20110623	6
38150286	20110325	15207814	19961021	1	20110325	5
38162399	20120427	16367257	19650906	1	20120427	5
38166493	20110617	15453828	19661208	2	20110617	5
38176271	20120131	16099062	19630202	1	20120131	4
3817774120130806		17791246	19660519	1	20130806	5
38191810	20120218	16163813	19461102	1	20120218	6
38205139	20110810	15611632	19410801	1	20110810	6
3821222520130723		17744797	19601220	1	20130723	6
38214027	20110801	15581087	19540609	1	20110801	5
38258070	20110531	15397613	19620721	2	20110531	4
38260412	20110724	15557701	19620724	1	20110724	6
38271102	20110923	15736341	19620610	1	20110923	4
3827147520130109		17148892	19520616	1	20130109	5
38271840	20120707	16583900	19400523	2	20120707	5

38282405	20110717	15539046	19490929	2	20110717	4
38282778	20121126	17009843	19490526	1	20121126	5
38287353	20110817	15631743	19580905	2	20110817	6
38289575	20121118	16985840	19480101	2	20121118	6
38292158	20110517	15363838	19920712	1	20110517	6
38295862	20111116	15894759	19561114	1	20111116	5
38307072	20111201	15932983	19530902	1	20111201	5
38311498	20120221	16171277	19331104	1	20120221	5
38316539	20130116	17171359	19420506	2	20130116	4
38367963	20111113	15883288	19760219	2	20111113	4
38368820	20111106	15862586	19681001	1	20111106	6
38395027	20120214	16150763	19470520	1	20120214	4
38402878	20110902	15675475	19780302	1	20110902	5
38416272	20110925	15738166	19760715	1	20110925	5
38422707	20110927	15745007	19790206	1	20110927	5
38435755	20130603	17584432	19221112	2	20130603	6
38459960	20111008	15782061	19421117	1	20111008	4
38487239	20111116	15894741	19641208	1	20111116	5
38496116	20110930	15753606	19621108	2	20110930	6
38497506	20130124	17194766	19510801	1	20130124	5
38502328	20110915	15713977	19771125	1	20110915	6
38509476	20111121	15906951	19640921	1	20111121	5
38510155	20120105	16040774	19551215	2	20120105	4
38510666	20130603	17583816	19481117	1	20130603	6
38524515	20130823	17845092	19370116	2	20130823	5
38524695	20111231	16022078	19661216	1	20111231	6
38532046	20120810	16687185	19590122	2	20120810	5
38532295	20111021	15818385	19620101	1	20111021	4
38543601	20111006	15773763	19951012	1	20111006	4
38545107	20111105	15861978	19591101	1	20111105	6
38561783	20111014	15797025	19990919	2	20111014	5
38569594	20111002	15756690	19620606	1	20111002	4
38585976	20111207	15956183	19680301	1	20111207	4
38601933	20120506	16395077	19680103	1	20120506	6
38602050	20120920	16809243	19691228	2	20120920	6
38605979	20120229	16190036	19630720	2	20120229	4
38616692	20120315	16244523	19330507	2	20120315	4
38625115	20110927	15744992	19710421	1	20110927	5
38625240	20130210	17242245	19450610	1	20130210	4
38625819	20111030	15837987	19571216	1	20111030	5
38626323	20120330	16283181	19640120	1	20120330	5
38637171	20120719	16620843	19351230	1	20120719	5
38642772	20120114	16069077	19740905	2	20120114	6
38651999	20120909	16773012	19540228	1	20120909	6
38652367	20120205	16119787	19290202	1	20120205	6
38654772	20120725	16637380	19320804	2	20120725	5
38656267	20130529	17570160	20000117	1	20130529	4
38660912	20120522	16443926	19800718	2	20120522	4
38661380	20120916	16794107	19490325	1	20120916	4
38663386	20120127	16092973	19411001	1	20120127	6
38665780	20120128	16093979	19480720	1	20120128	4
38676787	20130428	17471992	19800921	2	20130428	5
38679651	20111115	15891124	19500323	2	20111115	5
38681264	20111110	15877704	19461229	1	20111110	5
38688787	20120430	16372958	19650115	1	20120430	4

38690016	20120801	16656366	19530910	2	20120801	4
38698894	20111121	15905195	19620526	1	20111121	4
38700311	20120604	16479858	19590830	2	20120604	5
38701869	20120610	16500164	19351001	1	20120610	5
38717872	20120822	16722550	19431215	1	20120822	6
38729510	20111228	16015676	19920807	1	20111228	6
38737610	20120103	16032868	19490510	1	20120103	5
38751085	20120109	16050904	19540701	1	20120109	5
3877027320130914		17917047	19600710	2	20130914	4
38776599	20120116	16072547	19570831	2	20120116	6
3877838020130702		17676558	19550205	1	20130702	5
3877937220130128		17202530	19301015	1	20130128	5
38788577	20120313	16236748	19600728	1	20120313	6
3878940120130401		17384679	19671123	2	20130401	5
38804056	20121215	17075842	19650203	1	20121215	6
38810116	20120911	16780838	19340210	1	20120911	6
38815199	20120103	16031842	19361029	1	20120103	5
3883679220130908		17894066	19290118	1	20130908	5
38851397	20120102	16027291	19990701	2	20120102	5
38856018	20120211	16142889	19670114	2	20120211	6
38866578	20120212	16143268	19550303	2	20120212	6
3887608320130612		17615110	19461111	1	20130612	4
38888027	20121116	16980934	19360128	2	20121116	4
38892852	20120326	16271770	19620211	1	20120326	6
38910804	20120305	16209506	19600226	1	20120305	6
38911465	20120330	16283829	19290308	1	20120330	5
38913472	20120505	16394648	19510708	2	20120505	6
3891483920130424		17462902	19480812	1	20130424	4
38914920	20120328	16278467	19530906	1	20120328	5
3892313620130904		17882290	19401014	2	20130904	4
38941309	20120423	16354355	19520926	2	20120423	4
3896467920130201		17215493	19781029	1	20130201	4
38966642	20121108	16956809	19500810	1	20121108	4
38969607	20120429	16369614	19601203	1	20120429	5
38972291	20120527	16454780	19520420	1	20120527	5
38978197	20120526	16454062	19640306	2	20120526	5
38986775	20120316	16248411	19740519	1	20120316	5
39011273	20120516	16428734	19420120	2	20120516	5
39018536	20120726	16640701	19401114	1	20120726	5
39049177	20120815	16702441	19640212	1	20120815	6
39061648	20120509	16408302	19500130	2	20120509	6
39063473	20120603	16474814	19941107	1	20120603	5
3906604120131226		18240507	19371005	2	20131226	5
39072601	20120925	16821604	19711206	1	20120925	5
39074061	20120626	16544308	19630402	1	20120626	5
39079317	20120731	16651425	19550720	1	20120731	6
39079715	20120604	16475792	19700617	1	20120604	5
39086890	20120611	16502791	19550815	1	20120611	6
39097137	20120626	16545302	19960620	1	20120626	4
39098378	20120820	16715531	20070101	2	20120820	5
39105287	20120622	16537103	19621115	1	20120622	4
39131890	20120705	16577954	19460824	1	20120705	4
39134695	20120702	16561923	19550906	1	20120702	5
39136884	20120919	16803574	19530315	2	20120919	6
39160617	20120805	16665142	19610724	1	20120805	6

39163230	20120829	16739193	19370921	2	20120829	6
39168939	20120806	16668567	19580316	1	20120806	5
39176697	20120531	16466371	19440926	2	20120531	4
39177338	20121031	16926004	19510830	1	20121031	4
39178626	20120628	16551132	19740812	2	20120628	5
39185609	20120906	16765772	19670825	1	20120906	4
39190266	20121022	16902508	19440123	1	20121022	5
39209784	20130611	17614663	19400628	2	20130611	6
39216029	20120724	16634046	19430205	1	20120724	6
39217760	20130726	17755694	19480522	2	20130726	6
39222190	20120909	16773201	19570819	1	20120909	5
39223693	20121016	16886094	19491010	1	20121016	6
39224629	20120803	16662253	19610217	2	20120803	4
39231486	20120928	16830031	19611211	1	20120928	4
39231793	20120930	16831826	19540620	2	20120930	5
39269197	20120816	16705560	19730920	2	20120816	5
39282230	20120915	16793879	19810426	1	20120915	4
39285171	20120817	16707195	19411113	1	20120817	5
39288034	20120822	16722325	19700817	1	20120822	5
39296043	20121107	16952429	19680920	2	20121107	5
39303043	20121208	17052443	19581105	1	20121208	5
39309778	20120924	16817605	19581215	1	20120924	6
39322286	20121206	17045763	19480701	2	20121206	5
39334173	20131027	18046056	19560305	1	20131027	5
39340222	20121015	16881828	19461015	2	20121015	6
39341383	20130715	17720488	19950712	2	20130715	5
39350408	20130718	17731317	19641206	1	20130718	4
39363401	20120912	16785354	19930225	2	20120912	6
39370939	20130517	17538790	19261109	1	20130517	5
39385507	20121223	17097543	19530529	1	20121223	6
39385698	20130613	17619011	19661126	2	20130613	6
39391601	20130304	17301994	19430215	1	20130304	5
39391805	20121229	17114669	19571126	2	20121229	4
39402090	20130515	17530658	19510311	2	20130515	6
39429986	20130129	17206055	19240918	1	20130129	5
39451939	20130306	17310328	19740729	1	20130306	4
39476318	20121214	17074934	19510208	1	20121214	4
39477935	20130819	17832162	19410810	2	20130819	4
39486265	20130324	17365898	19540819	1	20130324	4
39489822	20130220	17264468	19600213	1	20130220	4
39517776	20130815	17823392	19580419	2	20130815	6
39519852	20121205	17039572	19520612	1	20121205	6
39523121	20130109	17147697	19650520	1	20130109	4
39524011	20130507	17502933	19400220	2	20130507	6
39529823	20130804	17780715	19531228	1	20130804	6
39531378	20130220	17264471	19421017	2	20130220	5
39531732	20130101	17117921	19430801	1	20130101	5
39534902	20121111	16963389	19750528	1	20121111	5
39559847	20130101	17117930	19590219	1	20130101	5
39560526	20121211	17060897	19590715	2	20121211	5
39570417	20130130	17209526	19551202	1	20130130	5
39587398	20130316	17345121	19560214	1	20130316	6
39592977	20121116	16982336	19911110	1	20121116	4
39593845	20121202	17025148	19450302	1	20121202	5
39596413	20121216	17077410	19491015	1	20121216	4

3960615020131024	18040860	19481215	1	20131024	5
3963572820130614	17623173	19600721	1	20130614	5
3963647020130428	17471657	19560125	1	20130428	5
3965100820130218	17253257	19470909	1	20130218	5
3965146220130828	17858072	19470409	1	20130828	6
3965505520131109	18094645	19501101	1	20131109	4
3965977320130130	17209536	19450118	2	20130130	5
3966597920130429	17475154	19440501	1	20130429	6
3968735920130125	17196965	19351129	1	20130125	6
3969417220130107	17138709	19991125	1	20130107	4
3972104520130218	17254024	19690127	1	20130218	5
3972963020130226	17281170	19460317	2	20130226	6
3972975420130205	17230759	19520915	1	20130205	5
3973032020130207	17238425	19340909	2	20130207	4
3973256420130701	17671772	19680225	1	20130701	5
3973291720130412	17429009	19800203	1	20130412	5
3974573820130311	17326966	19740410	1	20130311	5
3976774320131203	18168195	19511101	2	20131203	6
3977193220130219	17259974	19451124	1	20130219	6
3979522720130307	17316010	19550517	1	20130307	4
3979955820130409	17414265	19640605	2	20130409	5
3984030720130418	17445882	19471101	1	20130418	5
3986566220130929	17954848	19610529	1	20130929	6
3989788620130313	17335923	19580327	2	20130313	4
3991954520130604	17584483	19610215	1	20130604	6
3992475920131127	18148911	19401110	1	20131127	5
3993171020130515	17531808	19710906	1	20130515	6
3993338520130519	17540991	19650530	2	20130519	4
3993754720130512	17519361	19700423	2	20130512	4
3993797820131003	17973966	19640116	1	20131003	6
3998288220130705	17690295	19761021	2	20130705	6
4001227920130530	17572892	19990523	1	20130530	4
4001686220130527	17563860	19530114	1	20130527	4
4001886820130617	17628573	19590302	2	20130617	6
4002019720130827	17854321	19460218	1	20130827	5
4002398120131229	18245909	19711119	2	20131229	5
4004986320131022	18033574	19471022	1	20131022	6
4005985620130527	17562066	19630907	2	20130527	6
4007482620130705	17690226	19520910	1	20130705	4
4008045320130723	17744595	19820503	1	20130723	5
4008624620130826	17851014	19501018	2	20130826	6
4015347720130730	17764097	19970910	1	20130730	4
4017136620130718	17730738	19570613	1	20130718	4
4018731320130714	17715864	19641023	2	20130714	4
4020141020130710	17707593	19530911	1	20130710	5
4020773620130923	17937469	19490402	1	20130923	5
4020868220131224	18234931	19700224	1	20131224	6
4023268620130921	17933405	19940519	2	20130921	5
4023966520130825	17847561	19530512	2	20130825	5
4024613720130723	17745770	19611208	1	20130723	4
4024869920131222	18228301	19540604	1	20131222	5
4028294820130829	17860908	19580228	2	20130829	4
4031233220130903	17875087	19370303	1	20130903	6
4038226120130915	17917488	19660118	2	20130915	6
4039823020130926	17949478	19751025	1	20130926	5

4042501220131120	18128823	19571204	1	20131120	5
4042540920130901	17864953	19740225	1	20130901	4
4044457320131223	18231877	19450411	1	20131223	5
4045263920131203	18167834	19490110	1	20131203	5
4046280420130926	17949115	19520324	2	20130926	4
4046433320131217	18216146	19481012	1	20131217	4
4046476420131120	18129509	19641128	1	20131120	4
4047052820131105	18078439	19770923	1	20131105	4
4047462220131101	18061850	19610202	1	20131101	5
4050137920131110	18095109	19330823	1	20131110	4
4051664120131026	18045196	19561110	2	20131026	5
4051766620131006	17981872	19620910	1	20131006	5
122634 20111212	15970680	19280506	1	20111212	5
424237 20120503	16385636	19290906	2	20120503	5
42828420130226	17281329	19241025	1	20130226	5
429685 20120926	16823156	19270604	1	20120926	5
55706220131006	17981993	19210321	1	20131006	5
1030348 20110425	15296074	19580723	2	20110425	4
117888720130611	17613446	19670525	2	20130611	5
1195682 20111021	15817088	19670603	1	20111021	5
1376825 20120801	16654907	19240611	1	20120801	5
1417965 20111118	15901203	19300208	2	20111118	5
157746420121206	17045886	19320502	2	20121206	6
1947144 20110429	15307577	19220729	1	20110429	4
2035098 20120605	16484656	19360105	1	20120605	4
2251947 20111219	15990304	19491107	2	20111219	4
2307484 20120519	16436691	19390612	2	20120519	5
249303020130325	17369264	19290924	1	20130325	4
3594523 20120719	16621453	19320920	1	20120719	5
359933520130912	17911279	19500410	1	20130912	6
364931820130910	17903039	19291208	1	20130910	5
378874720130812	17811241	19330116	1	20130812	4
3965853 20120720	16624141	19300303	1	20120720	4
3967940 20120201	16104239	19500803	1	20120201	6
4011761 20120906	16765631	19291228	1	20120906	5
4012526 20120719	16621813	19391213	2	20120719	4
419969320130130	17208464	19180314	2	20130130	4
4591268 20120831	16743007	19270429	1	20120831	5
4596707 20120717	16613800	19310608	1	20120717	5
4608428 20121104	16938321	19330727	1	20121104	6
4752505 20120505	16394426	19250415	1	20120505	5
4956052 20110608	15421370	19390723	1	20110608	5
498227820130314	17339196	19370112	1	20130314	6
5065441 20120903	16752583	19550103	2	20120903	5
5115935 20120808	16679596	19310921	2	20120808	6
5434899 20110813	15621718	19480312	2	20110813	6
573094920130208	17241790	19551221	1	20130208	6
579585720130515	17531646	19371112	1	20130515	6
602421120131117	18118320	19281226	1	20131117	5
6447809 20121107	16951259	19490910	1	20121107	5
6464240 20120429	16369645	19260323	1	20120429	6
6546765 20120711	16596088	19340404	1	20120711	4
681352120121108	16955445	19260416	1	20121108	6
6889265 20110607	15419787	19290716	2	20110607	4
6987913 20120910	16777026	19430124	1	20120910	6

7116861	20110806	15600209	19410109	1	20110806	6
754164220131025		18043993	19520228	1	20131025	4
765123220131210		18195191	19240706	1	20131210	4
7717773	20110612	15434780	19451201	2	20110612	5
7804220	20120717	16613967	19290325	1	20120717	6
7830333	20120423	16355013	19230225	1	20120423	5
787161620130315		17342608	19420324	1	20130315	5
7910330	20120408	16311034	19401229	2	20120408	4
7966383	20121105	16943698	19500423	1	20121105	6
8211049	20110526	15388089	19590723	2	20110526	4
851587520130306		17310619	19341017	1	20130306	6
8617376	20120406	16308376	19411020	1	20120406	5
8787357	20120921	16810533	19221123	1	20120921	5
948595420130922		17934043	19300802	2	20130922	4
9557226	20120726	16640482	19600906	2	20120726	4
972749520131203		18165573	19320209	2	20131203	6
973372620130520		17544903	19791012	1	20130520	5
9868068	20120217	16162252	19521201	1	20120217	5
10074869	20110303	15143489	19330918	1	20110303	6
10411742	20111206	15952722	19450215	2	20111206	5
10678850	20111118	15898311	19290202	1	20111118	6
10853197	20120703	16568365	19550225	1	20120703	6
1092411520130526		17560164	19150116	1	20130526	6
11032545	20120528	16456887	19351207	2	20120528	5
11101465	20120109	16051889	19510916	1	20120109	4
11140822	20111205	15947643	19290505	1	20111205	5
11604469	20120513	16417695	19500114	2	20120513	4
12429300	20111025	15827436	19430520	2	20111025	5
12475159	20110624	15470734	19600712	2	20110624	5
1261494720130830		17861011	19291205	1	20130830	5
1264027620130227		17285459	19460310	2	20130227	6
1269306020130519		17541116	19750428	1	20130519	6
1279843520131015		18010849	19510902	1	20131015	6
1295578120130724		17749390	19480305	2	20130724	4
12965423	20111005	15770800	19381220	1	20111005	5
12974866	20120129	16094386	19421021	1	20120129	5
13019506	20120519	16436416	19580324	1	20120519	6
13183023	20111002	15756668	19290205	1	20111002	5
13431726	20120926	16823619	19400425	2	20120926	5
1357174920130925		17945206	19511112	2	20130925	6
1403923720130618		17632248	19250114	1	20130618	4
14046107	20121114	16975830	19440708	2	20121114	4
1407477220130524		17558027	19500822	2	20130524	5
1434056820131008		17992278	19510704	2	20131008	4
14406667	20110411	15254406	19291222	1	20110411	5
14591572	20120305	16208620	19450318	2	20120305	5
1529456120131128		18151759	19500329	1	20131128	4
15679206	20120419	16347156	19521015	1	20120419	6
15752417	20120128	16094178	19371207	2	20120128	4
17049955	20111105	15861971	19591008	2	20111105	6
17057260	20120701	16556854	19580730	1	20120701	4
17094814	20120520	16436983	19550910	2	20120520	4
1723955320130811		17806642	19540806	2	20130811	5
17244143	20110928	15748085	19400101	2	20110928	5
17282574	20110504	15324720	19320129	1	20110504	5

17605117	20120912	16784135	19450107	2	20120912	5
17706513	20130605	17593125	19300824	1	20130605	4
17717714	20130401	17390199	19521022	1	20130401	5
18132484	20120329	16280454	19640109	2	20120329	4
18137821	20130729	17760770	19630919	1	20130729	6
18193970	20130409	17416186	19440804	1	20130409	4
18305823	20130408	17410355	19510115	2	20130408	4
18309109	20110926	15741863	19460804	1	20110926	6
18425440	20130506	17500956	19351003	1	20130506	6
18441479	20120904	16757368	19670804	2	20120904	4
18460509	20120112	16063838	19340321	2	20120112	6
18520257	20130906	17890551	19341213	2	20130906	5
18572628	20121020	16898456	19311020	1	20121020	5
19221499	20120116	16072525	19400706	1	20120116	5
19228332	20130118	17178106	19510806	1	20130118	4
19372793	20120605	16482606	19590418	2	20120605	4
19588933	20130629	17665388	19600511	1	20130629	5
19726900	20110607	15419487	19571020	2	20110607	6
19816183	20110628	15478841	19700119	1	20110628	6
19858470	20120805	16665206	19620910	1	20120805	4
19889328	20130528	17566703	19490218	1	20130528	5
20059943	20130918	17929604	19580124	2	20130918	6
20332256	20121011	16872261	19410512	2	20121011	6
20365257	20120209	16136408	19640704	2	20120209	5
20490248	20130415	17433840	19231015	1	20130415	4
20579231	20120218	16163899	19540930	1	20120218	4
21087069	20131114	18111365	19541019	2	20131114	6
21175326	20110829	15661684	19630411	1	20110829	4
21181500	20130910	17902900	19610203	1	20130910	4
21307084	20130131	17212156	19361125	1	20130131	6
21458060	20120727	16642253	19310318	1	20120727	6
21461494	20130301	17293673	19350908	1	20130301	5
21466433	20120424	16355774	19330608	1	20120424	4
21489394	20130905	17885974	19470913	1	20130905	4
21822957	20130403	17398893	19580106	1	20130403	6
21861665	20130531	17573481	19300226	1	20130531	6
21997422	20130624	17648678	19510427	1	20130624	5
22063189	20120603	16474413	19420515	1	20120603	6
22370587	20120705	16577812	19410308	1	20120705	4
22405910	20120730	16645668	19590922	1	20120730	6
22434237	20130923	17938270	19370307	1	20130923	5
22554514	20121106	16948277	19500428	1	20121106	6
22575980	20110517	15363789	19580429	2	20110517	4
22864706	20120421	16351353	19261028	1	20120421	4
22881545	20131003	17972817	19380816	2	20131003	4
23085576	20111012	15790936	19641214	2	20111012	4
23133995	20121009	16867387	19481120	1	20121009	5
23156561	20121102	16936035	19620607	2	20121102	4
23175420	20130917	17925982	19580120	1	20130917	5
23367819	20110620	15459367	19580201	2	20110620	5
23879621	20111107	15866083	19281031	1	20111107	5
24139524	20110422	15290643	19690811	2	20110422	5
24203938	20110904	15678806	19370224	2	20110904	6
24415701	20120921	16810772	19651216	1	20120921	6
24481729	20110427	15302389	19380724	1	20110427	4

24518329	20111127	15921616	19381024	2	20111127	5
24534314	20120111	16059790	19261025	1	20120111	5
24826195	20130125	17196874	19510607	1	20130125	4
24918136	20110912	15702646	19531208	1	20110912	4
25067054	20130804	17780839	19681029	1	20130804	5
25311857	20131207	18184851	19400625	1	20131207	4
25335299	20120707	16583877	19600404	1	20120707	5
25396978	20120508	16403351	19401126	1	20120508	6
25489718	20121028	16917254	19370805	2	20121028	6
25495801	20120909	16773132	19501214	1	20120909	5
25587706	20110517	15363853	19500905	2	20110517	4
25631510	20120501	16379187	19451014	1	20120501	5
25727406	20110517	15363623	19601225	1	20110517	4
25852044	20130314	17340175	19400106	1	20130314	4
25853092	20120625	16541148	19301222	2	20120625	6
25879714	20120411	16324389	19510126	1	20120411	6
25935380	20120603	16474693	19380502	2	20120603	4
26123682	20120229	16189968	19520823	2	20120229	5
26213467	20130319	17353368	19570503	2	20130319	6
26412179	20120910	16777086	19530824	1	20120910	6
26439281	20130716	17724501	19751228	2	20130716	5
26564090	20131024	18039935	19531011	1	20131024	6
26638584	20130824	17847037	19430506	2	20130824	4
26845498	20131022	18033518	19440520	1	20131022	4
26993793	20120413	16331589	19550707	1	20120413	5
27219096	20120716	16610691	19500225	1	20120716	4
27453174	20131017	18018910	19520215	1	20131017	6
27477618	20121022	16903114	19440319	1	20121022	5
27482286	20110318	15190465	19360909	2	20110318	6
27615107	20130707	17693687	19401208	1	20130707	4
27626182	20120501	16378866	19641225	1	20120501	5
27658033	20120127	16092991	19770528	2	20120127	5
27678724	20120218	16164026	19590410	2	20120218	6
27746425	20111011	15784468	19550811	2	20111011	4
27843794	20111225	16005651	19440614	1	20111225	4
27871712	20120722	16626959	19420716	1	20120722	5
27911928	20130219	17259283	19520129	1	20130219	4
27937833	20130114	17162194	19530320	1	20130114	4
28018884	20110809	15607558	19461010	2	20110809	5
28040757	20130626	17657826	19410316	1	20130626	4
28110089	20130315	17343490	19650328	1	20130315	6
28169815	20130813	17813450	19721006	2	20130813	6
28214655	20111106	15862502	19530622	2	20111106	5
28280491	20120502	16381978	19370615	2	20120502	6
28300950	20120217	16162182	19651122	1	20120217	4
28383746	20130417	17444254	19350226	2	20130417	6
28386983	20111027	15833011	19470105	2	20111027	4
28699709	20120804	16664476	19550920	1	20120804	5
28885861	20120223	16177681	19360726	1	20120223	5
29182818	20120906	16764985	19490606	1	20120906	4
29204271	20120318	16250428	19610828	2	20120318	5
29285492	20131019	18025408	19490214	2	20131019	5
29385646	20110616	15449928	19450215	2	20110616	4
29454475	20131004	17979589	19641009	2	20131004	5
29454997	20120130	16098197	19460603	1	20120130	5

29566232	20130910	17902575	19521229	1	20130910	6
29731122	20120605	16482660	19420416	2	20120605	6
29794410	20130604	17588860	19490105	1	20130604	5
29800213	20131018	18021933	19570703	1	20131018	5
29810739	20120219	16164201	19700420	1	20120219	5
29881081	20120511	16413692	19511219	1	20120511	5
29951813	20110529	15392251	19630923	2	20110529	5
30002007	20120604	16479003	19670116	2	20120604	4
30202290	20111031	15840898	19650830	1	20111031	5
30267095	20120615	16518321	19541120	1	20120615	6
30290723	20110501	15309292	19660524	1	20110501	4
30406883	20120202	16111250	19320103	1	20120202	5
30425140	20131002	17968191	19340305	1	20131002	6
30619900	20111021	15818166	19590110	1	20111021	5
30647848	20110517	15363668	19520806	1	20110517	4
30696630	20110416	15272135	19541215	2	20110416	5
30829595	20130321	17360067	19630215	1	20130321	4
30937941	20121001	16835943	19550128	2	20121001	4
30999496	20120624	16538003	19650912	1	20120624	6
31003846	20130408	17412217	19251205	2	20130408	5
31101936	20130526	17560150	19270313	2	20130526	5
31235195	20130731	17766095	19240804	2	20130731	4
31261571	20130314	17340075	19620805	1	20130314	4
31319058	20110412	15259229	19790916	1	20110412	5
31388246	20131103	18066760	19701018	1	20131103	5
31393994	20120410	16320048	19490225	1	20120410	4
31558733	20131020	18025899	19500114	2	20131020	4
31728526	20120912	16785423	19320925	1	20120912	5
31750046	20110322	15199568	19660908	2	20110322	5
31751118	20130219	17258319	19411012	2	20130219	4
31759598	20131028	18049028	19750814	1	20131028	4
31765658	20131005	17981668	19331008	1	20131005	6
31999607	20111214	15976527	19690426	2	20111214	6
32114424	20130725	17751529	19340113	1	20130725	6
32245855	20110415	15270757	19540501	1	20110415	4
32405440	20121130	17022128	19460115	1	20121130	5
32509481	20110510	15343981	19670627	1	20110510	6
32551501	20130513	17522484	19490228	1	20130513	6
32771985	20130217	17250690	19540223	1	20130217	4
32776344	20121001	16837153	19650101	1	20121001	5
32818910	20120503	16388696	19660620	1	20120503	4
32827057	20120622	16536946	19930709	1	20120622	5
32921623	20120629	16554841	19730729	2	20120629	4
32974439	20120710	16591250	19300211	1	20120710	4
33058525	20120507	16399627	19360120	1	20120507	6
33118331	20110325	15208596	19420927	2	20110325	6
33306659	20130714	17716162	19531128	2	20130714	4
33321447	20130525	17559922	19480203	1	20130525	6
33377489	20130110	17153427	19430513	1	20130110	5
33428454	20130819	17833276	19460924	2	20130819	5
33472990	20130820	17835440	19400222	1	20130820	5
33478034	20120623	16537360	19581204	1	20120623	6
33514362	20111228	16015249	19650430	1	20111228	5
33617219	20131007	17987098	19650110	1	20131007	6
33630783	20130223	17273733	19341111	2	20130223	6

33870938	20131115	18115813	19730728	2	20131115	4
33982079	20120424	16358840	19731210	2	20120424	5
34009320	20110905	15683578	19791127	1	20110905	5
34071926	20130414	17431451	19430608	1	20130414	5
34082865	20120102	16027443	19270505	2	20120102	4
34168826	20121001	16837499	19390523	1	20121001	5
34260327	20130925	17945403	19570216	1	20130925	6
34275871	20120219	16164449	19461013	2	20120219	5
34305481	20130911	17906767	19410708	2	20130911	4
34385803	20121123	17004313	19601203	2	20121123	5
34403899	20130318	17348649	19460525	2	20130318	4
34413199	20110512	15351411	19710612	2	20110512	5
34413315	20110510	15343749	19550102	2	20110510	4
34442347	20120405	16303856	19290915	1	20120405	4
34444694	20111027	15833090	19611019	1	20111027	5
34476249	20130925	17945792	19600510	1	20130925	5
34510571	20110428	15303279	19830123	1	20110428	6
34524157	20121203	17029837	19600417	1	20121203	4
34569012	20111116	15894782	19320207	2	20111116	4
34615902	20111121	15906917	19570110	1	20111121	5
34741087	20110629	15483160	19370709	2	20110629	5
34779732	20120102	16026174	19410726	1	20120102	6
34803015	20120325	16268395	19431204	1	20120325	6
34812629	20130214	17246288	19700805	1	20130214	5
34858225	20131007	17985759	19480817	1	20131007	5
34876170	20111121	15906776	19270919	1	20111121	5
34879475	20130530	17573166	19660925	2	20130530	5
34893975	20131001	17963644	19411012	2	20131001	5
34904148	20130410	17421214	19400602	1	20130410	4
34904875	20111013	15795247	19491216	1	20111013	6
35112697	20121025	16912638	19430627	2	20121025	5
35198835	20131011	17999113	19730702	2	20131011	5
35205379	20131224	18234224	19671216	1	20131224	5
35210710	20120502	16384233	19580717	2	20120502	6
35214621	20120205	16119813	19530920	1	20120205	5
35239511	20120712	16600630	19571025	2	20120712	4
35279324	20130902	17871769	19570211	1	20130902	5
35303016	20121130	17021347	19371129	2	20121130	6
35356071	20130207	17237104	19500910	1	20130207	6
35434812	20130206	17235039	19520314	1	20130206	4
35510282	20110912	15702599	19690726	2	20110912	6
35536835	20130514	17528047	19770826	1	20130514	5
35565787	20120401	16285562	19530123	2	20120401	6
35766939	20120904	16755226	19340301	2	20120904	6
35767498	20120130	16098263	19670615	1	20120130	4
35824556	20120126	16090858	19540415	1	20120126	5
35843404	20111029	15837509	19650606	1	20111029	4
35843835	20110510	15343745	19681031	2	20110510	6
35843880	20130415	17436008	19350209	1	20130415	6
35861724	20120530	16463864	19450304	1	20120530	6
35940251	20110512	15351425	19640711	2	20110512	5
35982866	20120214	16150085	19681016	2	20120214	4
36073866	20130828	17858075	19760227	2	20130828	5
36121887	20110607	15419718	19491024	2	20110607	4
36149572	20130423	17460244	19700203	1	20130423	4

36176519	20120112	16062949	19510430	2	20120112	6
36195401	20131006	17981816	19501025	2	20131006	5
36219382	20130619	17638062	19420221	2	20130619	4
36254338	20130714	17715749	19501105	1	20130714	5
36276570	20110510	15343754	19400831	2	20110510	5
36312308	20111220	15994224	19500415	2	20111220	4
36325947	20110529	15392210	19840305	1	20110529	4
36330286	20120404	16299258	19320315	1	20120404	6
36411559	20110811	15616080	19650620	2	20110811	6
36430203	20110918	15719343	19611004	1	20110918	5
36488876	20110511	15347833	19500101	1	20110511	4
36502360	20120702	16563102	19411216	2	20120702	4
36524820	20131220	18226422	19650602	1	20131220	6
36593687	20130313	17336435	19540114	1	20130313	4
36602887	20121204	17036708	19540523	2	20121204	4
36669951	20130710	17707589	19580214	1	20130710	6
36689846	20110903	15677342	19421230	1	20110903	5
36692587	20110515	15356574	19620215	2	20110515	6
36726860	20110513	15354711	19520824	2	20110513	4
36746506	20130428	17471859	19670102	1	20130428	5
36771456	20110526	15386766	19480726	2	20110526	5
36791976	20130312	17331508	19400911	1	20130312	5
36816838	20111127	15921859	19580615	2	20111127	5
36817831	20120722	16627056	19520404	1	20120722	5
36876547	20110527	15390394	19500314	1	20110527	6
36878065	20110916	15715896	19610720	1	20110916	4
36907832	20110818	15636314	19730802	2	20110818	5
36931234	20111027	15833310	20000513	1	20111027	6
36952768	20130307	17317864	19560730	2	20130307	6
36964382	20120221	16171489	19390711	1	20120221	5
37013868	20131125	18141712	19510114	2	20131125	6
37021957	20131107	18086832	19660802	2	20131107	6
37064452	20121201	17024391	19480520	1	20121201	4
37084165	20131022	18033606	19610225	2	20131022	4
37213131	20110922	15733490	19690519	1	20110922	4
37268498	20120228	16186220	19480710	1	20120228	5
37271913	20120423	16355424	19510319	1	20120423	4
37273362	20121017	16890098	19440701	2	20121017	4
37323992	20121021	16899173	19480315	2	20121021	5
37339256	20120414	16333232	19871128	1	20120414	5
37351396	20110721	15552874	19741106	2	20110721	6
37352593	20131121	18132244	19491215	2	20131121	5
37359049	20120111	16060100	19660314	1	20120111	6
37370459	20110728	15569611	19760221	1	20110728	5
37385094	20111208	15960985	19510404	1	20111208	6
37392000	20111205	15947147	19530115	2	20111205	5
37436127	20110311	15170485	19640221	2	20110311	5
37450183	20110805	15598458	19430122	2	20110805	4
37457139	20131030	18054682	19220810	1	20131030	6
37494410	20111108	15870424	19780206	1	20111108	4
37500291	20111114	15887109	19650420	1	20111114	6
37501318	20111207	15957190	19460612	1	20111207	5
37512235	20110629	15483204	19720118	1	20110629	6
37538082	20121107	16952922	19590320	2	20121107	4
37565927	20111117	15898027	19370308	1	20111117	4

37580215	20110509	15339258	19680614	1	20110509	6
37583689	20110419	15279892	19370901	2	20110419	4
37587590	20120320	16257620	19881118	1	20120320	5
37606012	20111122	15910649	20081022	2	20111122	5
37623522	20110804	15594972	19670424	2	20110804	5
37643019	20110928	15748098	19290720	1	20110928	4
37657424	20110908	15696425	19480216	1	20110908	5
37679122	20120325	16268822	19531001	1	20120325	5
37736235	20120621	16533281	19740205	1	20120621	6
37743194	20121015	16882205	19631006	1	20121015	5
37761936	20110508	15335150	19621217	2	20110508	6
37767514	20110512	15351567	19971003	1	20110512	5
37771485	20110610	15431716	19501016	1	20110610	5
37793241	20121022	16901813	19680801	2	20121022	6
37796717	20120218	16163550	19311101	1	20120218	6
37800207	20110627	15476139	19530615	1	20110627	4
37800387	20120926	16824750	19630206	1	20120926	6
37832829	20110509	15338402	19390418	2	20110509	4
37835099	20111016	15800899	19590319	1	20111016	5
37839955	20120226	16182800	19550205	1	20120226	6
3784035020130118		17178239	19490325	1	20130118	5
37850934	20120204	16119147	19570406	1	20120204	6
37851051	20120415	16333572	19520116	1	20120415	5
37851426	20110731	15575209	19800627	1	20110731	4
37851608	20110725	15561353	19490720	2	20110725	4
37872745	20121213	17071412	19430606	1	20121213	5
37873420	20111002	15756657	19540103	2	20111002	5
37881100	20120409	16315777	19721002	1	20120409	5
3789350820131130		18155605	19491204	1	20131130	5
37905638	20120923	16814146	19281113	1	20120923	6
3790706520130323		17365263	19380925	1	20130323	4
37924848	20110509	15339452	19610327	1	20110509	5
37927121	20120205	16119919	19500221	2	20120205	5
37929707	20111230	16020532	19440401	1	20111230	6
3793248220130610		17610195	20071009	1	20130610	6
37946068	20111028	15835433	19620108	1	20111028	4
37950633	20120320	16255800	19460525	2	20120320	6
37954282	20110923	15736278	19610625	2	20110923	5
37979741	20110819	15639285	19390828	1	20110819	5
37993503	20120522	16443531	19580505	2	20120522	6
37994857	20110805	15597832	19661020	1	20110805	4
37995270	20110414	15267330	19620908	1	20110414	5
3802449620130217		17250437	19630924	1	20130217	5
38025977	20110408	15246319	19590208	1	20110408	6
3803488720130621		17643865	19541231	2	20130621	4
3806334220130526		17560494	19390814	2	20130526	5
38086703	20110623	15469609	19820418	2	20110623	4
38096774	20110728	15570896	19530106	1	20110728	4
38097346	20110416	15272192	20100420	1	20110416	5
38112946	20120617	16521105	19330505	1	20120617	6
38121378	20110619	15455746	19590501	1	20110619	6
38123114	20120322	16263898	19470818	2	20120322	5
38130062	20120712	16600949	19511025	1	20120712	6
38130255	20110621	15463003	19390508	1	20110621	4
3813265920130109		17147461	19531108	1	20130109	4

38136673	20111026	15828598	19540628	1	20111026	4
38146166	20110606	15415069	19720712	1	20110606	6
3814902920130101		17117743	19590322	2	20130101	5
3817774120130906		17888915	19660519	1	20130906	4
38183889	20120510	16411261	19440925	1	20120510	5
38238629	20111013	15795280	19541113	1	20111013	5
38242670	20120318	16250444	19480623	2	20120318	5
38250189	20110817	15633193	19570825	1	20110817	6
38252630	20110630	15485987	19680502	1	20110630	4
38260412	20111005	15769967	19620724	1	20111005	4
38271066	20120826	16729726	19610414	1	20120826	4
38288685	20110919	15719887	19320503	1	20110919	4
38291939	20110819	15639054	19470220	1	20110819	5
38299386	20110706	15508049	19630519	1	20110706	6
38311852	20120325	16268674	19550827	1	20120325	6
38368820	20120110	16055609	19681001	1	20120110	5
38369481	20120229	16189820	19630722	1	20120229	6
38386593	20111030	15837915	19540816	1	20111030	6
38395027	20120227	16184883	19470520	1	20120227	6
38402765	20120102	16027256	19450306	1	20120102	4
38402878	20111029	15837454	19780302	1	20111029	5
38405173	20111121	15906192	19351028	2	20111121	5
38410729	20120106	16044030	19490922	2	20120106	6
38428818	20120128	16093738	19510327	2	20120128	6
38458172	20120907	16770103	19450217	1	20120907	6
38473233	20120328	16278361	19490912	2	20120328	5
38477202	20111022	15819983	19390109	1	20111022	4
38480078	20111130	15931149	19860622	2	20111130	4
38502328	20111002	15756743	19771125	1	20111002	4
38507743	20120306	16214588	19810515	2	20120306	4
38508133	20120317	16250101	19450314	1	20120317	4
38510155	20120126	16090649	19551215	2	20120126	4
3854039520130814		17819240	19540208	1	20130814	6
38562128	20111228	16015576	19541002	1	20111228	6
38564691	20121203	17030544	19750203	1	20121203	5
3857293920130930		17957456	19310115	2	20130930	4
38576395	20120104	16036915	19680608	1	20120104	5
38601933	20120708	16584476	19680103	1	20120708	4
38626323	20120501	16378178	19640120	1	20120501	4
38642772	20120403	16297078	19740905	2	20120403	5
38645077	20120220	16168149	19681009	1	20120220	6
38650203	20120406	16308395	19601010	1	20120406	4
38656870	20120324	16268229	19660808	2	20120324	5
38660821	20120813	16694715	19600901	1	20120813	5
38662816	20121125	17006361	19331006	1	20121125	6
38663466	20120106	16044718	19340522	2	20120106	4
38666283	20120220	16167454	20000129	2	20120220	5
38669986	20120306	16209675	19460615	1	20120306	5
3867678720130520		17545095	19800921	2	20130520	6
38677064	20120130	16095242	19671220	1	20120130	5
38701869	20120801	16652682	19351001	1	20120801	5
38703605	20121112	16966851	19321107	1	20121112	5
38733367	20120105	16041237	19320405	2	20120105	5
38751085	20120128	16093899	19540701	1	20120128	6
38756819	20121111	16963473	19520828	2	20121111	6

38781167	20120522	16443876	19590417	1	20120522	5
3880405620130109		17148429	19650203	1	20130109	5
38808796	20120626	16545611	19470908	1	20120626	5
38809835	20120411	16324101	19410521	2	20120411	6
3881011620130311		17327251	19340210	1	20130311	6
3881697620130626		17657748	19570104	1	20130626	6
38831582	20120310	16229194	19530830	2	20120310	4
3883234720130829		17860736	19420321	1	20130829	4
38833453	20120723	16630539	19590614	1	20120723	6
38833942	20120125	16089941	19550907	2	20120125	5
38849853	20120127	16092446	19651216	1	20120127	4
38856665	20121211	17060749	19580505	2	20121211	4
38866578	20120302	16200313	19550303	2	20120302	6
3887608320131124		18137927	19461111	1	20131124	6
38879468	20120925	16820877	19410419	1	20120925	6
3888802720130419		17450071	19360128	2	20130419	6
38897653	20120910	16776139	19701122	1	20120910	5
38910804	20120312	16233123	19600226	1	20120312	6
38915810	20121111	16963576	19551226	1	20121111	5
38927376	20121024	16909616	19550105	2	20121024	6
39011035	20120417	16339894	19660405	2	20120417	6
39017533	20121223	17097624	19500427	1	20121223	5
39022770	20120814	16699085	19510509	2	20120814	5
39061648	20120528	16458120	19500130	2	20120528	6
3906191120130501		17483937	19640103	1	20130501	5
39063473	20120626	16545617	19941107	1	20120626	5
39069846	20120911	16779641	19670917	2	20120911	6
39073546	20120704	16571302	19610120	1	20120704	5
39077004	20120806	16670448	19640902	1	20120806	5
3908369720130508		17509570	19541221	1	20130508	6
39110082	20121005	16854795	19540810	1	20121005	6
39131890	20120729	16645535	19460824	1	20120729	4
39151310	20121219	17089032	19600906	2	20121219	4
3915493320130301		17292714	19671226	2	20130301	6
39155185	20120930	16831697	19430101	1	20120930	6
39165850	20121104	16938661	19520921	1	20121104	5
39168939	20120830	16741880	19580316	1	20120830	6
39177338	20121113	16967910	19510830	1	20121113	4
39178693	20120708	16584490	19550415	1	20120708	5
3918091120130429		17475129	19590617	1	20130429	6
39190028	20120926	16824670	19550626	2	20120926	6
3919026620130119		17179838	19440123	1	20130119	6
39193925	20120730	16646380	19500730	2	20120730	5
3922792420131111		18098936	19350401	1	20131111	5
39232923	20121109	16960331	19590325	1	20121109	5
39238205	20121124	17006149	19520320	2	20121124	5
39259002	20121014	16878298	19440303	1	20121014	5
39261988	20120830	16741886	19950403	1	20120830	5
39266416	20121115	16979920	19551010	1	20121115	6
39268525	20120913	16789085	19681203	1	20120913	4
3927825620130603		17583580	19570701	1	20130603	5
39282230	20121005	16855581	19810426	1	20121005	6
3928803420130115		17167998	19700817	1	20130115	4
39300679	20121125	17006642	19640610	2	20121125	5
39326040	20121030	16923286	19531222	1	20121030	5

39332713	20130118	17178268	19730211	2	20130118	5
39359289	20121031	16925970	19400716	1	20121031	4
39374022	20121116	16983513	19590109	2	20121116	5
39385698	20130807	17795869	19661126	2	20130807	5
39387865	20121128	17016484	19490806	1	20121128	6
39400925	20130114	17162209	19450619	1	20130114	4
39415286	20121016	16885655	19680910	1	20121016	5
39422587	20121129	17018671	19410108	1	20121129	5
39425917	20121020	16898633	19711106	2	20121020	4
39450618	20121221	17094390	19481021	1	20121221	5
39486265	20130422	17456931	19540819	1	20130422	4
39489822	20130315	17343488	19600213	1	20130315	4
39517232	20130424	17463939	19680108	1	20130424	5
39527258	20130107	17140558	19500602	1	20130107	5
39530615	20130530	17572326	19380303	2	20130530	5
39531732	20130130	17209548	19430801	1	20130130	4
39559847	20130127	17200272	19590219	1	20130127	5
39560526	20131030	18053985	19590715	2	20131030	5
39570417	20130312	17332739	19551202	1	20130312	6
39584139	20130605	17593043	19420618	1	20130605	5
39592148	20130131	17212445	19601014	1	20130131	6
39597518	20130222	17272114	19670410	2	20130222	6
39598055	20130120	17180681	19400323	1	20130120	4
39599898	20130305	17307291	19660716	1	20130305	6
39627333	20121223	17097570	19660420	1	20121223	6
39645233	20131203	18168026	19590115	1	20131203	6
39647080	20130612	17615128	19320323	2	20130612	6
39647591	20130217	17250658	19961219	1	20130217	6
39651393	20130815	17822411	19721027	2	20130815	6
39659773	20130227	17285648	19450118	2	20130227	5
39688852	20130511	17518842	19580530	1	20130511	4
39715941	20130806	17791175	19691231	1	20130806	4
39732779	20131027	18046074	19601225	1	20131027	6
39732917	20130430	17477816	19800203	1	20130430	5
39746786	20130601	17577781	19490107	2	20130601	6
39768326	20130301	17292533	19800223	2	20130301	4
39769614	20130331	17383895	19600727	1	20130331	5
39774602	20130513	17523804	20050726	2	20130513	5
39791667	20130331	17383803	19710225	1	20130331	5
39795590	20130424	17463731	19550420	1	20130424	4
39812005	20130315	17343459	19631027	2	20130315	5
39823319	20130512	17519110	19250429	2	20130512	6
39848389	20130312	17331321	19670406	1	20130312	6
39894592	20130818	17829185	19660917	1	20130818	4
39895302	20130424	17463851	19720515	1	20130424	5
39910582	20130607	17603212	19601006	2	20130607	6
39937912	20130718	17732053	19590628	2	20130718	6
39997392	20130508	17509496	19520915	2	20130508	6
39998204	20130702	17675074	20060817	1	20130702	5
40028351	20130530	17573193	19710809	1	20130530	6
40056368	20130624	17648670	19640701	1	20130624	6
40080453	20130816	17825066	19820503	1	20130816	5
40086246	20131011	18000842	19501018	2	20131011	5
40089563	20131006	17982214	20120618	1	20131006	5
40139604	20130823	17843727	19720918	1	20130823	4

4019572020130827		17854221	19731225	2	20130827	5
4019832120131029		18051960	19401005	1	20131029	5
4020781620130921		17933344	19400525	1	20130921	6
4023268620131017		18020250	19940519	2	20131017	6
4025007520131001		17962994	19471118	1	20131001	5
4027637720131101		18060060	19530905	1	20131101	6
4042245520131027		18045808	19620105	1	20131027	4
4042676420131115		18115542	19360308	1	20131115	5
4042995620131030		18053373	19730627	1	20131030	4
4043645120131118		18120735	19630110	2	20131118	4
4046018220131101		18062863	19490223	1	20131101	6
4048953020131126		18144203	19761011	1	20131126	6
4049799220131121		18132326	19621210	2	20131121	6
4050713920131119		18125198	19701107	1	20131119	4
4051664120131112		18104545	19561110	2	20131112	5
4053557720131209		18188237	19661023	1	20131209	5
4062926320131209		18190066	19380405	1	20131209	5
59767	20120311	16229830	19421027	2	20120311	6
8163020130422		17456986	19290916	1	20130422	5
122634	20120117	16076211	19280506	1	20120117	5
32646520131101		18063897	19350218	2	20131101	5
41389820130113		17159742	19240713	1	20130113	6
1298653	20111031	15840957	19501124	2	20111031	5
1417965	20111204	15943149	19300208	2	20111204	5
1445665	20120307	16217560	19510223	2	20120307	5
1492315	20120826	16729869	19360705	1	20120826	5
1669405	20120425	16361889	19210920	1	20120425	6
2307484	20120717	16614639	19390612	2	20120717	6
2979717	20121127	17013330	19440218	1	20121127	5
326868620130307		17317727	19450102	2	20130307	5
3559146	20111006	15776014	19491013	1	20111006	5
364931820130919		17929829	19291208	1	20130919	5
3783742	20111011	15786359	19420522	2	20111011	4
4088975	20120401	16285410	19500314	2	20120401	6
4311799	20120712	16599841	19490315	1	20120712	5
4458031	20120909	16772793	19540111	1	20120909	4
5065441	20120919	16805837	19550103	2	20120919	5
516411420130910		17903156	19501115	1	20130910	6
5301315	20120630	16556483	19301229	2	20120630	5
551804720130714		17715757	19230506	1	20130714	6
5570058	20111205	15947087	19490925	2	20111205	4
5912072	20120409	16315614	19560705	1	20120409	4
5996474	20120708	16584247	19420215	2	20120708	5
6447809	20121206	17046169	19490910	1	20121206	5
6597177	20110928	15747937	19280414	2	20110928	4
691693420130310		17324181	19700103	1	20130310	6
7066093	20120520	16436831	19490302	1	20120520	6
7291261	20120528	16458051	19310801	1	20120528	5
7830333	20120430	16372611	19230225	1	20120430	4
7910330	20120430	16373004	19401229	2	20120430	4
7966383	20121122	17001332	19500423	1	20121122	4
8196198	20121019	16897038	19450825	2	20121019	5
8372187	20121207	17048381	19360312	2	20121207	6
838354820130715		17720334	19451207	1	20130715	5
875530420131014		18007554	19530730	1	20131014	4

9221232	20121028	16917168	19530701	2	20121028	6
9392169	20120813	16694897	19500601	2	20120813	6
9733726	20130606	17597661	19791012	1	20130606	5
10234261	20120805	16664728	19511011	1	20120805	6
10568328	20111023	15820536	19430915	2	20111023	5
10900864	20121113	16969527	19500810	2	20121113	4
11032545	20120613	16511698	19351207	2	20120613	4
11064178	20130731	17766984	19491001	1	20130731	5
11101465	20120229	16190443	19510916	1	20120229	5
11169792	20120308	16221234	19480915	1	20120308	5
11298729	20110826	15657234	19570702	2	20110826	4
11964537	20120218	16163938	19301004	1	20120218	5
11993650	20120714	16605514	19610502	1	20120714	6
12210592	20120419	16347214	19441021	1	20120419	5
12441348	20120806	16670460	19680417	1	20120806	4
12639848	20131206	18180873	19551028	2	20131206	6
12640276	20130408	17412348	19460310	2	20130408	6
12680249	20110710	15518543	19521105	1	20110710	6
12693060	20130626	17657246	19750428	1	20130626	6
12714668	20130429	17474789	19451117	2	20130429	5
12940997	20120730	16648660	19821109	1	20120730	5
12955781	20130813	17815413	19480305	2	20130813	4
12965423	20111017	15804285	19381220	1	20111017	6
13131776	20121014	16878096	19471010	1	20121014	5
13183023	20111016	15800941	19290205	1	20111016	5
13302631	20120822	16719838	19420528	2	20120822	5
13317356	20130123	17191479	19600127	1	20130123	6
13485671	20131015	18009769	19290826	1	20131015	4
13488658	20130907	17893028	19300115	1	20130907	5
13667613	20120314	16241464	19400414	2	20120314	6
13973701	20131105	18075969	19501123	1	20131105	4
14125156	20130530	17573090	19441117	2	20130530	5
14340568	20131017	18018399	19510704	2	20131017	5
14435440	20111130	15931037	19300428	1	20111130	4
14476961	20110811	15613062	19541117	1	20110811	6
14756479	20130720	17737836	19400101	1	20130720	5
14863219	20111011	15787257	19481214	2	20111011	4
15079693	20111114	15886695	19370705	1	20111114	6
15332328	20120414	16332319	19610509	2	20120414	4
15579392	20120208	16132492	19420926	1	20120208	4
15759383	20120613	16511763	19680929	1	20120613	5
15816850	20130304	17299226	19300301	1	20130304	4
16170233	20131023	18037132	19651225	2	20131023	4
16696983	20121001	16837046	19640709	2	20121001	5
16899437	20120617	16520981	19700201	2	20120617	5
16956700	20120326	16270529	19540606	1	20120326	4
17094814	20120601	16469835	19550910	2	20120601	4
17239553	20130817	17828673	19540806	2	20130817	6
17261186	20111226	16008419	19520511	2	20111226	5
17271431	20130318	17348463	19740828	1	20130318	4
17489508	20110920	15726765	19581112	1	20110920	6
17539865	20110424	15292549	19350527	1	20110424	5
17735614	20130322	17363567	19650717	2	20130322	4
17745856	20111130	15930111	19300205	1	20111130	6
17771992	20131003	17972600	19400515	2	20131003	6

17993554	20120117	16077428	19580212	1	20120117	4
18137821	20131001	17964062	19630919	1	20131001	5
18151105	20131223	18231965	19590710	1	20131223	5
18236083	20110814	15622308	19540502	2	20110814	5
18462867	20130418	17447644	19570907	1	20130418	4
18491162	20131117	18118129	19440222	1	20131117	5
18608043	20130526	17560412	19651118	2	20130526	6
18936373	20110519	15370640	19550520	2	20110519	5
18995614	20110729	15573365	19270101	2	20110729	5
19636987	20110421	15286353	19381210	2	20110421	5
19703652	20120306	16213636	19610730	2	20120306	4
19889328	20130618	17633028	19490218	1	20130618	6
20216084	20110504	15322874	19560809	2	20110504	6
20361562	20120312	16233747	19480326	2	20120312	5
20579231	20120302	16201208	19540930	1	20120302	4
20581311	20130215	17248620	19630819	1	20130215	6
21017312	20110819	15639282	19410420	2	20110819	6
21516610	20121219	17087890	19751002	1	20121219	6
21519437	20111218	15987212	19350219	1	20111218	5
21951688	20120712	16601082	19620126	1	20120712	4
21997422	20130719	17735337	19510427	1	20130719	6
22455432	20130128	17203249	19490218	2	20130128	6
22562056	20111228	16015678	19340406	1	20111228	5
22639209	20130805	17784748	19601201	1	20130805	6
22863565	20130528	17567359	19541020	1	20130528	6
22915260	20131110	18095037	19690318	1	20131110	4
23183804	20130313	17334601	19380310	2	20130313	6
23198836	20110411	15254837	19450104	2	20110411	4
23272711	20131021	18027617	19670820	2	20131021	4
23598745	20130706	17693277	19670316	1	20130706	5
23664393	20131203	18168102	19371016	1	20131203	5
23831994	20111218	15987285	19680530	2	20111218	4
24035516	20110611	15434179	19460617	1	20110611	4
24348003	20130625	17653967	19380617	2	20130625	6
24481729	20110506	15332855	19380724	1	20110506	4
24534314	20120126	16090560	19261025	1	20120126	6
24633689	20120529	16460269	19410401	1	20120529	5
24635107	20131021	18029707	19390313	1	20131021	5
24846579	20121016	16886127	19320806	1	20121016	5
24885801	20111116	15894663	19631125	1	20111116	5
24918136	20111210	15966072	19531208	1	20111210	4
24956896	20120526	16454351	19640922	1	20120526	5
25131877	20120903	16752747	19300214	2	20120903	6
25464237	20120104	16037293	19501015	2	20120104	5
25587706	20110603	15412226	19500905	2	20110603	5
25718698	20131110	18095162	19661022	1	20131110	6
25844080	20120527	16454632	19710309	2	20120527	4
25879714	20120416	16335631	19510126	1	20120416	6
25982187	20130616	17625475	19540623	1	20130616	6
25992749	20120115	16069529	19700310	2	20120115	4
26007567	20120101	16022488	19700514	1	20120101	4
26062748	20120114	16069256	19680517	1	20120114	5
26198801	20120807	16675461	19351120	1	20120807	5
26213467	20130412	17429457	19570503	2	20130412	5
26232199	20120517	16432031	19580102	1	20120517	5

26439281	20130729	17759653	19751228	2	20130729	6
26930927	20120511	16414490	19430531	2	20120511	6
27041607	20130114	17164042	19540504	2	20130114	6
27120861	20131127	18147633	19410418	2	20131127	4
27199662	20110731	15575190	19961119	1	20110731	6
27482286	20110325	15208498	19360909	2	20110325	5
27487634	20110729	15573453	19311019	2	20110729	5
27561995	20121212	17066386	19340525	2	20121212	5
27640239	20111111	15880639	19350325	1	20111111	4
27878440	20111101	15844559	19361115	2	20111101	6
28158103	20130130	17209502	19651124	1	20130130	4
28214655	20111130	15931013	19530622	2	20111130	5
28271161	20130410	17419897	19520403	1	20130410	6
28349964	20130423	17458820	19630924	2	20130423	6
28844726	20130701	17668886	19371104	2	20130701	5
28885974	20121001	16837372	19571124	1	20121001	5
29208502	20121120	16993983	19410303	2	20121120	4
29307651	20110809	15608172	19621201	1	20110809	6
29342254	20120802	16657449	19640606	1	20120802	5
29526690	20120106	16044781	19610922	2	20120106	4
29618028	20130227	17284317	19410928	2	20130227	6
29625670	20120918	16802210	19600220	1	20120918	5
29633510	20120522	16443865	19350423	1	20120522	4
29730630	20121015	16882431	19400403	1	20121015	4
29800213	20131204	18170995	19570703	1	20131204	5
29998672	20120129	16094394	19520519	1	20120129	6
30006112	20130325	17368659	19320928	2	20130325	5
30202290	20111123	15911194	19650830	1	20111123	6
30413479	20130828	17858051	19510501	2	20130828	5
30451184	20121121	16997704	19521227	2	20121121	6
30574620	20131016	18015941	19440612	1	20131016	5
30587258	20120130	16097743	19621203	1	20120130	5
30717147	20130704	17686721	19400512	1	20130704	4
30725225	20120205	16119886	19681220	2	20120205	5
30902739	20131117	18118334	19370804	1	20131117	5
30974528	20131112	18100108	19530509	2	20131112	5
31254758	20130218	17253595	19590218	1	20130218	6
31678418	20110912	15702638	19570616	1	20110912	5
31757990	20130814	17819407	19411112	1	20130814	6
31759598	20131118	18122120	19750814	1	20131118	4
32018589	20130620	17640136	19540406	2	20130620	4
32384648	20130306	17311264	19211003	1	20130306	4
32401528	20110528	15391729	19280405	1	20110528	4
32506926	20130421	17453426	19540101	1	20130421	6
32800025	20130707	17693747	19931102	2	20130707	6
32818498	20111012	15791494	19651207	1	20111012	5
33009948	20131124	18138165	19630105	1	20131124	4
33112968	20121202	17025281	19450603	2	20121202	5
33256814	20111104	15858648	19550913	1	20111104	6
33289644	20130720	17737802	19490410	2	20130720	6
33371914	20120606	16488582	19510922	1	20120606	5
33472990	20130825	17847188	19400222	1	20130825	5
33490389	20120306	16214530	19590320	2	20120306	4
33572144	20110910	15701708	19550720	2	20110910	5
33632041	20130604	17588832	19590606	1	20130604	5

33676390	20130203	17220086	19651228	1	20130203	5
33780766	20121219	17089027	19580308	1	20121219	6
34042563	20130922	17934109	19680706	1	20130922	4
34098969	20120327	16274602	19451106	1	20120327	6
34110015	20130826	17850885	19690514	1	20130826	4
34128466	20111122	15910453	19760221	2	20111122	5
34168826	20121119	16988136	19390523	1	20121119	5
34246418	20120310	16229187	19520306	2	20120310	4
34270718	20110921	15727950	19581106	2	20110921	4
34278847	20110627	15477065	19600120	2	20110627	5
34435182	20120206	16122590	19240412	2	20120206	4
34524157	20130107	17138941	19600417	1	20130107	5
34876170	20111130	15929858	19270919	1	20111130	5
34921443	20130911	17907485	19560621	1	20130911	5
34947905	20130301	17292961	19660703	1	20130301	6
34961427	20120905	16760977	19600215	1	20120905	5
35026405	20130125	17197204	20070712	1	20130125	4
35105727	20120402	16288029	19641010	2	20120402	5
35132026	20120320	16256392	19500219	1	20120320	5
35184282	20130228	17288269	19610214	1	20130228	4
35301485	20130506	17500521	19580405	1	20130506	5
35400227	20130306	17312834	19661213	2	20130306	4
35434812	20130501	17484093	19520314	1	20130501	5
35465964	20121227	17109595	19441130	1	20121227	4
35522931	20120127	16092543	19480220	1	20120127	4
35561912	20120112	16063865	19521026	1	20120112	4
35609202	20120412	16328206	19340112	2	20120412	6
35687162	20120114	16069264	19700809	2	20120114	4
35692070	20120803	16662156	19400818	2	20120803	6
35729896	20111009	15782469	19600705	2	20111009	4
35745790	20110804	15595085	19610118	2	20110804	4
35797967	20120323	16266659	19540208	2	20120323	4
35843404	20111119	15903057	19650606	1	20111119	6
35861724	20120821	16719257	19450304	1	20120821	6
36236836	20110606	15415227	19630922	2	20110606	6
36276570	20110531	15396553	19400831	2	20110531	6
36333150	20111207	15957101	19530612	1	20111207	5
36430203	20111001	15756073	19611004	1	20111001	5
36453404	20130309	17323833	19591118	2	20130309	6
36459220	20131204	18173659	19410922	1	20131204	4
36467795	20120229	16190476	19470822	1	20120229	5
36507581	20110925	15738565	19390510	1	20110925	4
36513903	20120412	16327029	19961118	1	20120412	5
36669951	20130809	17804347	19580214	1	20130809	5
36753885	20130302	17296308	19690621	1	20130302	6
36757456	20120201	16107590	19560310	2	20120201	5
36762251	20111021	15818392	19331126	2	20111021	5
36771456	20110616	15450442	19480726	2	20110616	5
36791976	20131114	18112257	19400911	1	20131114	4
36813680	20110825	15653992	19430720	2	20110825	6
36927261	20110727	15567754	19670610	2	20110727	5
36935270	20111225	16005728	19930412	2	20111225	6
36996288	20120509	16406505	19570325	1	20120509	4
37036752	20120313	16237584	19570524	2	20120313	5
37064203	20121031	16925022	19460805	1	20121031	5

37070658	20131023	18037098	19491013	2	20131023	4
37105218	20131224	18234753	19630102	2	20131224	6
37124008	20110817	15632370	19500518	2	20110817	4
37165370	20110514	15356219	19400615	1	20110514	5
37182506	20130624	17650886	19600218	2	20130624	4
37225062	20111223	16003881	19580610	1	20111223	4
37271913	20120605	16484754	19510319	1	20120605	5
37299835	20120101	16022687	19920514	1	20120101	6
37372295	20120208	16132126	19371204	2	20120208	6
37389336	20110906	15687272	19461111	2	20110906	6
37403911	20110926	15739562	19670421	1	20110926	5
37436025	20121125	17006684	19510920	1	20121125	5
37437653	20110913	15706423	19590106	2	20110913	4
37537216	20130408	17412324	19560821	2	20130408	6
37538082	20121126	17009997	19590320	2	20121126	4
37556993	20120311	16229816	19390227	1	20120311	4
37574542	20130804	17781004	19431118	2	20130804	6
37604027	20130222	17268681	19760828	2	20130222	6
37623362	20110907	15692492	19511125	1	20110907	5
37657424	20110927	15743858	19480216	1	20110927	5
37669479	20110816	15629831	19490701	1	20110816	6
37698741	20110616	15450551	19471225	2	20110616	6
37708080	20110614	15443124	19830430	2	20110614	5
37734773	20120207	16127073	19900806	1	20120207	4
37739609	20130724	17749191	19501207	2	20130724	6
37746035	20111209	15963140	20010613	1	20111209	4
37793241	20121108	16955177	19680801	2	20121108	5
37800207	20110801	15579031	19530615	1	20110801	4
37800387	20121225	17102328	19630206	1	20121225	4
37802065	20131126	18145481	19631220	1	20131126	4
37804152	20110929	15749839	19590401	1	20110929	5
37838123	20110731	15575135	19540812	1	20110731	5
37850934	20120916	16794353	19570406	1	20120916	6
37857582	20130323	17365558	19650207	2	20130323	6
37878812	20130206	17235238	19500228	1	20130206	5
37887517	20111007	15778339	19420624	1	20111007	5
37891637	20110629	15482608	19420222	1	20110629	4
37899288	20130613	17618924	19470916	1	20130613	4
37922477	20120309	16227116	19531218	1	20120309	4
37926684	20120221	16171853	19590520	1	20120221	5
37941392	20120326	16271913	19450708	1	20120326	5
37946068	20111113	15883382	19620108	1	20111113	5
37952606	20120505	16394597	19510718	1	20120505	4
37961925	20120520	16437143	19471111	2	20120520	6
37965278	20111108	15870735	19480809	1	20111108	4
37977472	20110623	15469633	19460902	1	20110623	5
37994857	20110829	15661090	19661020	1	20110829	5
37995270	20110422	15290568	19620908	1	20110422	4
38002107	20120910	16775260	19590427	1	20120910	5
38024496	20130318	17348227	19630924	1	20130318	6
38025977	20121024	16908867	19590208	1	20121024	6
38034478	20110511	15345072	19540530	1	20110511	4
38045191	20130507	17503902	19560818	1	20130507	5
38061379	20121210	17057506	19431111	2	20121210	5
38086703	20110720	15550194	19820418	2	20110720	5

38114000	20110816	15629800	19471201	1	20110816	5
38114691	20121126	17010007	19401127	1	20121126	5
38116846	20111219	15990573	19771208	1	20111219	5
38121878	20110626	15473849	19620316	1	20110626	5
38127912	20120320	16257605	19351208	1	20120320	6
38129883	20130902	17871727	19441127	1	20130902	5
38130255	20120307	16219124	19390508	1	20120307	4
38143963	20110620	15459061	19511017	2	20110620	4
38177741	20131009	17996858	19660519	1	20131009	6
38178288	20111006	15776038	19450109	2	20111006	4
38178722	20110823	15647848	19590420	2	20110823	4
38183889	20120801	16655868	19440925	1	20120801	5
38196177	20110724	15558049	19490517	1	20110724	6
38217402	20111109	15874561	19480126	1	20111109	5
38258070	20110822	15644768	19620721	2	20110822	4
38258478	20120820	16713758	19530722	1	20120820	4
38292614	20121008	16860756	19361011	1	20121008	6
38318091	20110925	15738590	19730605	2	20110925	6
38345072	20120823	16725298	19720217	1	20120823	6
38354211	20120214	16149798	19651010	1	20120214	6
38359830	20120725	16637575	19500910	1	20120725	4
38363927	20120422	16351754	19530220	1	20120422	5
38381543	20131125	18141785	19480102	2	20131125	5
38386935	20131006	17982224	19571008	1	20131006	5
38398366	20111117	15898085	19940821	2	20111117	4
38399165	20111122	15910511	19560210	1	20111122	5
38401693	20111020	15815375	19680606	2	20111020	6
38402878	20111109	15873973	19780302	1	20111109	4
38403757	20130804	17780851	19611224	1	20130804	6
38405173	20120426	16364712	19351028	2	20120426	6
38416669	20120310	16229221	19600920	1	20120310	5
38421920	20130324	17366157	19600207	1	20130324	5
38422707	20111221	15996260	19790206	1	20111221	6
38428772	20120309	16226487	19460927	1	20120309	5
38456665	20121016	16886185	19520913	2	20121016	5
38471588	20111108	15869348	19531124	2	20111108	5
38473619	20130306	17311133	19370713	1	20130306	5
38486907	20111023	15820273	19950929	2	20111023	5
38502328	20111023	15820588	19771125	1	20111023	4
38520273	20111212	15970598	19690101	1	20111212	6
38522280	20120527	16454741	19680616	2	20120527	4
38530302	20120916	16794218	19581201	1	20120916	6
38541718	20111104	15860222	19380522	1	20111104	4
38562128	20120214	16151991	19541002	1	20120214	4
38569594	20120228	16187479	19620606	1	20120228	5
38584122	20121010	16867788	19620825	2	20121010	4
38591252	20120701	16556965	19420820	1	20120701	4
38626583	20120814	16698931	19781029	1	20120814	5
38627224	20120731	16651548	19771002	1	20120731	6
38634581	20130818	17829215	19561022	2	20130818	6
38642181	20120508	16403840	19510823	2	20120508	5
38652094	20120309	16227179	19590816	1	20120309	5
38652367	20120408	16310984	19290202	1	20120408	5
38660332	20120714	16605772	19620714	2	20120714	6
38660821	20120927	16827582	19600901	1	20120927	5

38662816	20121213	17070025	19331006	1	20121213	5
38662861	20120527	16454552	19440123	1	20120527	4
38663193	20120306	16213577	19640925	2	20120306	4
38690016	20121005	16855326	19530910	2	20121005	5
38702102	20120115	16069792	19481130	2	20120115	4
38703650	20120401	16285304	19740416	1	20120401	5
38724695	20120326	16270016	19531018	1	20120326	5
38763369	20120624	16538151	19620408	1	20120624	5
38763483	20120223	16178040	19940708	2	20120223	5
38765092	20120329	16280590	19641211	1	20120329	5
38788577	20120517	16432016	19600728	1	20120517	6
38790179	20120508	16404181	19440708	1	20120508	6
38804056	20130212	17243571	19650203	1	20130212	6
38809619	20130321	17360789	19760906	1	20130321	4
38810116	20130328	17378922	19340210	1	20130328	5
38821351	20121125	17006365	19671125	2	20121125	5
38839882	20120520	16437137	19600115	1	20120520	6
38851397	20120201	16106556	19990701	2	20120201	6
38856665	20121218	17084641	19580505	2	20121218	4
38892852	20120512	16417165	19620211	1	20120512	5
38897197	20120623	16537525	19730723	1	20120623	5
38897200	20130905	17887382	19510724	1	20130905	4
38910804	20120402	16290841	19600226	1	20120402	6
38913609	20120614	16513386	19750414	1	20120614	4
38914839	20130606	17599006	19480812	1	20130606	6
38926088	20120522	16443899	19880520	1	20120522	5
38943509	20130228	17287008	19410523	1	20130228	5
38953898	20120503	16388367	19640824	2	20120503	5
38956240	20121116	16983324	19740330	2	20121116	4
38974651	20120519	16436637	19530710	1	20120519	4
39003366	20120904	16756440	19650115	1	20120904	6
39012196	20121017	16890319	19850713	2	20121017	5
39016109	20120522	16443622	19661010	2	20120522	4
39049177	20121011	16871031	19640212	1	20121011	6
39051246	20120809	16683673	19551227	1	20120809	4
39061648	20120812	16690678	19500130	2	20120812	6
39067839	20120812	16690400	19650426	2	20120812	6
39075951	20120905	16762136	19390316	1	20120905	4
39083482	20130415	17432735	19421031	1	20130415	5
39095040	20130508	17509242	19430721	2	20130508	5
39098378	20121006	16857600	20070101	2	20121006	6
39101796	20120808	16679745	19501126	1	20120808	4
39162793	20130206	17235234	19560719	1	20130206	4
39163230	20121106	16948493	19370921	2	20121106	5
39163821	20120911	16781118	19350525	1	20120911	5
39168939	20121003	16844877	19580316	1	20121003	6
39169749	20120724	16634099	19450228	2	20120724	4
39176697	20120628	16551880	19440926	2	20120628	6
39177338	20121224	17098949	19510830	1	20121224	6
39180911	20130607	17602921	19590617	1	20130607	6
39190028	20121206	17046132	19550626	2	20121206	5
39190675	20130715	17719262	19710902	2	20130715	4
39202250	20130123	17191226	19540614	2	20130123	4
39227582	20130908	17893816	19410411	1	20130908	4
39229522	20121028	16917347	19671112	1	20121028	4

39236130	20120802	16657668	19581010	2	20120802	5
39250389	20121007	16858256	19570228	1	20121007	4
39269197	20120929	16830339	19730920	2	20120929	5
39280723	20130904	17882210	19540113	2	20130904	6
39288034	20130131	17212291	19700817	1	20130131	5
39300679	20130204	17225944	19640610	2	20130204	6
39368280	20130517	17538418	19480806	1	20130517	4
39381925	20121115	16978690	19910411	1	20121115	5
39387865	20121229	17114816	19490806	1	20121229	4
39388095	20130314	17340051	19690110	1	20130314	4
39411262	20130111	17156012	19620128	1	20130111	5
39415286	20121022	16902948	19680910	1	20121022	4
39435568	20131021	18028673	19431215	1	20131021	6
39455271	20130326	17372547	19500702	1	20130326	5
39499655	20130621	17644508	19611128	2	20130621	5
39517232	20130513	17523786	19680108	1	20130513	4
39525207	20130201	17217748	19710401	2	20130201	5
39526119	20130122	17185830	19741014	1	20130122	4
39531652	20130702	17676459	19660425	1	20130702	5
39531732	20130301	17294131	19430801	1	20130301	6
39582360	20130217	17250489	19370709	1	20130217	6
39588799	20130312	17331756	19580622	2	20130312	6
39606150	20131114	18112775	19481215	1	20131114	5
39647591	20130323	17365452	19961219	1	20130323	5
39648685	20130501	17484227	19730321	1	20130501	6
39648798	20130306	17312748	19751225	2	20130306	5
39653720	20130618	17633785	19600224	1	20130618	4
39661342	20130322	17363521	19561016	1	20130322	6
39662958	20130515	17531711	20060224	2	20130515	6
39693168	20130715	17720076	20001031	1	20130715	5
39696667	20130708	17698099	19410516	1	20130708	5
39699337	20130405	17405479	19621231	1	20130405	5
39715941	20130910	17901808	19691231	1	20130910	4
39738766	20130718	17732717	19540101	1	20130718	4
39745738	20130506	17501023	19740410	1	20130506	6
39799558	20130713	17715615	19640605	2	20130713	4
39812005	20130328	17379482	19631027	2	20130328	4
39820627	20130606	17599187	19761208	1	20130606	6
39840307	20130620	17639435	19471101	1	20130620	4
39890169	20130326	17372214	19631017	2	20130326	4
39891264	20130426	17468935	19550921	2	20130426	6
39900726	20131229	18245959	19510410	2	20131229	4
39905378	20130516	17535684	19880611	1	20130516	6
39913218	20130523	17555180	19641130	2	20130523	5
39918940	20130610	17610474	19521108	1	20130610	4
39953963	20130531	17575726	19620719	2	20130531	6
39988084	20130615	17624320	19571110	1	20130615	6
39998204	20130818	17829098	20060817	1	20130818	5
40001330	20131122	18135902	19440922	1	20131122	6
40028351	20130623	17647300	19710809	1	20130623	5
40032766	20130922	17934013	19550603	1	20130922	5
40056368	20130723	17743543	19640701	1	20130723	4
40059856	20130728	17757847	19630907	2	20130728	6
40089563	20131105	18078315	20120618	1	20131105	5
40090220	20131003	17975227	19850329	1	20131003	6

4014175120130810		17806184	19570805	1	20130810	5
4015205420130915		17917329	19541225	1	20130915	6
4019119320130930		17958118	19671225	2	20130930	4
4019832120131122		18135392	19401005	1	20131122	5
4020112520130912		17911498	19650620	1	20130912	4
4026355620131208		18185470	19581107	2	20131208	5
4040072420131129		18154029	19520912	2	20131129	5
4041945220131020		18025842	19480529	1	20131020	6
4042995620131113		18106488	19730627	1	20131113	4
4048591420131218		18219300	19511230	1	20131218	5
4051564820131030		18054901	19710522	2	20131030	5
4053557720131216		18211852	19661023	1	20131216	6
822300	20120509	16408241	19491010	2	20120509	5
137682520130509		17512386	19240611	1	20130509	5
1417965	20120203	16117066	19300208	2	20120203	4
1555540	20110409	15249456	19300814	2	20110409	5
1575264	20120818	16711277	19381211	1	20120818	4
1947144	20110601	15403645	19220729	1	20110601	6
2010711	20120520	16437163	19461008	2	20120520	4
2251947	20120423	16354366	19491107	2	20120423	4
2305784	20120505	16394547	19330129	2	20120505	5
2307484	20120807	16673370	19390612	2	20120807	5
258778220130110		17153085	19500928	2	20130110	6
3028657	20120522	16443912	19280601	2	20120522	5
3283087	20120313	16237800	19420905	2	20120313	6
359452320130117		17174719	19320920	1	20130117	4
466827320131125		18141072	19270825	1	20131125	4
4943435	20120422	16351936	19310121	1	20120422	4
6261445	20121111	16963315	19521009	1	20121111	6
6589384	20120830	16741884	19480102	2	20120830	6
6766967	20120123	16088801	19560208	2	20120123	5
7066093	20120606	16489070	19490302	1	20120606	6
7966383	20121208	17051859	19500423	1	20121208	6
8348821	20120109	16051869	19351211	1	20120109	4
8483996	20120417	16340675	19440902	1	20120417	5
851587520130715		17718226	19341017	1	20130715	5
8666497	20120404	16299654	19410116	1	20120404	6
875530420131209		18189031	19530730	1	20131209	6
9143888	20121216	17077623	19730626	1	20121216	6
10271495	20120711	16597180	19430820	1	20120711	6
10537527	20120616	16520429	19490923	1	20120616	6
10754968	20120727	16643644	19520820	2	20120727	5
11967263	20120709	16588570	19420104	2	20120709	4
12075886	20110913	15706645	19501110	2	20110913	5
12110097	20120501	16379180	19510623	2	20120501	6
1211065520130409		17415758	19521017	2	20130409	4
12210592	20120510	16412191	19441021	1	20120510	4
1248146820130909		17898708	19531020	2	20130909	5
12680603	20120709	16584710	19480104	2	20120709	5
1271466820130806		17791135	19451117	2	20130806	6
1281405020131226		18240901	19500425	1	20131226	5
12965423	20120412	16328282	19381220	1	20120412	6
13183023	20111030	15837990	19290205	1	20111030	5
13345681	20111130	15931139	19690114	1	20111130	6
1339566920130314		17339546	19620814	2	20130314	4

1350800420131020		18025605	19240924	1	20131020	5
13639786	20120312	16233649	19640414	2	20120312	5
1364934820131229		18245748	19241020	1	20131229	5
13830110	20110724	15558105	19300414	1	20110724	5
1419968920130605		17592754	19401226	2	20130605	6
14863219	20111129	15927725	19481214	2	20111129	5
15371003	20111007	15780125	19350225	2	20111007	4
1580759720130814		17817935	19320410	1	20130814	5
1617023320131111		18099869	19651225	2	20131111	6
16376800	20120106	16045091	19770111	1	20120106	5
16636127	20111111	15881117	19310302	1	20111111	4
16899437	20120921	16812256	19700201	2	20120921	4
1691587020130428		17471837	19550425	1	20130428	5
16956700	20120612	16505053	19540606	1	20120612	4
17049955	20120413	16331441	19591008	2	20120413	6
17104379	20110418	15276912	19720811	2	20110418	6
17617708	20111219	15990877	19250715	1	20111219	6
1773561420130417		17443597	19650717	2	20130417	6
1777199220131104		18071580	19400515	2	20131104	4
1800630920130305		17307134	19550302	2	20130305	5
1807907720131018		18023787	19361120	2	20131018	6
18110628	20120201	16107691	19540826	1	20120201	5
18270092	20120228	16187391	19331209	1	20120228	6
1856582720131122		18135408	19580617	2	20131122	5
18682763	20120901	16746451	19481101	2	20120901	5
1872257120130325		17368881	19760323	2	20130325	6
18995614	20110906	15688257	19270101	2	20110906	6
19044054	20111127	15921925	19620414	1	20111127	5
1919520120130808		17800088	19420228	2	20130808	4
19542406	20111208	15960949	19400312	2	20111208	4
19726900	20110821	15641299	19571020	2	20110821	4
19731818	20120801	16655009	19360117	2	20120801	6
1978141020130805		17784727	19361002	2	20130805	6
19816183	20121018	16890959	19700119	1	20121018	6
1982522020130219		17258348	19320327	1	20130219	5
19858470	20121220	17092589	19620910	1	20121220	6
1994371620130701		17671167	19460224	1	20130701	6
20043021	20121216	17077641	19541212	2	20121216	6
2005002620130208		17241829	19630102	2	20130208	5
2006727020130223		17273610	19581005	2	20130223	4
2027112920130220		17264431	19430401	2	20130220	6
20486297	20120921	16812447	19590227	1	20120921	6
20579231	20120306	16213938	19540930	1	20120306	4
21017312	20120612	16506759	19410420	2	20120612	5
2108429720131127		18147025	19610903	1	20131127	4
2118498520130306		17312823	19510223	1	20130306	5
21466433	20120509	16404563	19330608	1	20120509	4
2165595820130504		17495156	19601105	1	20130504	5
21951688	20120802	16657734	19620126	1	20120802	6
2216941120131025		18043930	19560626	1	20131025	6
22261423	20120202	16108974	19850628	2	20120202	4
2245543220130329		17381983	19490218	2	20130329	5
22468208	20110815	15626152	19440920	1	20110815	4
23005529	20120419	16346754	19321211	1	20120419	6
23085576	20111128	15924662	19641214	2	20111128	5

23175420	20131023	18037145	19580120	1	20131023	5
23198836	20110502	15312252	19450104	2	20110502	6
23238804	20110806	15599952	19330316	1	20110806	6
23353642	20120713	16604015	19680914	2	20120713	5
23607901	20120626	16545260	19291220	1	20120626	5
24035516	20110726	15564433	19460617	1	20110726	6
24140894	20130223	17273514	19311019	1	20130223	5
24230282	20130122	17188173	19521224	2	20130122	5
24247867	20130218	17255382	19391013	2	20130218	4
24415701	20121224	17100767	19651216	1	20121224	5
24885801	20111130	15931081	19631125	1	20111130	4
25030346	20120223	16177375	19560103	1	20120223	6
25131877	20120923	16814573	19300214	2	20120923	6
25154078	20120703	16566851	19290903	2	20120703	4
25396978	20120619	16527257	19401126	1	20120619	4
25445798	20130925	17942771	19550331	1	20130925	4
25523437	20130422	17455480	19290911	2	20130422	6
25631510	20120525	16452918	19451014	1	20120525	4
25718698	20131206	18182841	19661022	1	20131206	5
25810597	20111204	15943471	19631121	2	20111204	5
26157426	20110816	15629124	19790509	2	20110816	5
26232199	20120618	16524677	19580102	1	20120618	6
26241532	20130407	17407331	19671127	2	20130407	4
26297483	20131024	18040534	19570721	2	20131024	4
26638584	20130919	17930072	19430506	2	20130919	6
26856644	20130322	17363492	19360318	2	20130322	6
26891623	20121102	16936098	19440216	2	20121102	4
26975053	20111215	15981768	19630617	2	20111215	5
27041607	20130222	17270682	19540504	2	20130222	4
27081443	20131114	18112267	19480503	2	20131114	6
27198272	20130415	17436054	19411225	2	20130415	4
27678724	20120509	16408308	19590410	2	20120509	5
27785246	20121007	16858245	19930707	1	20121007	6
27937833	20130911	17906221	19530320	1	20130911	6
28014779	20121101	16931521	19280608	2	20121101	4
28082317	20130917	17925966	19350702	2	20130917	4
28096879	20120202	16112721	19460601	2	20120202	6
28169815	20131107	18086589	19721006	2	20131107	5
28214655	20111222	16000864	19530622	2	20111222	5
28349862	20110614	15443122	19320823	2	20110614	6
28386983	20111126	15921070	19470105	2	20111126	5
28735037	20110902	15676445	19381018	1	20110902	4
29307651	20110816	15629372	19621201	1	20110816	6
29329688	20130613	17618512	19840214	1	20130613	6
29359602	20131106	18083667	19320807	1	20131106	6
29670426	20121129	17019654	19640530	1	20121129	4
29776214	20130805	17785710	19300101	2	20130805	5
29795571	20131114	18112746	19530330	2	20131114	5
29800213	20131222	18228238	19570703	1	20131222	4
29939886	20130308	17318376	19510725	2	20130308	4
30427599	20130117	17175104	19320226	1	20130117	6
30715118	20130425	17465479	19421027	1	20130425	5
30724255	20121022	16901704	19610906	1	20121022	4
30725225	20120310	16228974	19681220	2	20120310	5
30798324	20120528	16457842	19631016	2	20120528	5

30798959	20131211	18197344	19271015	1	20131211	6
30813286	20121208	17052650	19381122	1	20121208	4
30844805	20130411	17425382	19590605	2	20130411	5
31058692	20130524	17558050	19620411	1	20130524	6
31166800	20121121	16997891	19640207	2	20121121	5
31183978	20130610	17610379	19381006	2	20130610	4
31244925	20120101	16022733	19460421	1	20120101	5
31363152	20110618	15455460	19751022	1	20110618	6
31393256	20131216	18212301	19371217	1	20131216	5
31549992	20110704	15498385	20000612	1	20110704	5
31690194	20110915	15710755	19850117	2	20110915	5
31698836	20120903	16752656	19640213	2	20120903	5
31703829	20120919	16805829	19541220	1	20120919	6
31750046	20110506	15332871	19660908	2	20110506	4
31751118	20130819	17833357	19411012	2	20130819	6
31759598	20131211	18199191	19750814	1	20131211	4
31765658	20131123	18137568	19331008	1	20131123	6
31809106	20120904	16757768	19500820	2	20120904	5
32012069	20110807	15600996	19670127	1	20110807	4
32401528	20110628	15480348	19280405	1	20110628	5
32771985	20130331	17384006	19540223	1	20130331	4
32797481	20120608	16497588	19510302	1	20120608	4
32805337	20130912	17910881	19420401	2	20130912	6
32818498	20111106	15862685	19651207	1	20111106	5
33069102	20130510	17517133	19410310	1	20130510	6
33205559	20130422	17456146	19381206	1	20130422	5
33228272	20110807	15600811	19540529	1	20110807	4
33252016	20131112	18104521	19590625	1	20131112	4
33483237	20111106	15862662	19650212	2	20111106	5
33487795	20110818	15634123	19441221	2	20110818	4
33538464	20130424	17463803	19690425	2	20130424	5
33676390	20130613	17619447	19651228	1	20130613	5
34205851	20120608	16497433	19411208	1	20120608	5
34218752	20130921	17933407	19350310	1	20130921	5
34246418	20120319	16253896	19520306	2	20120319	5
34264341	20111117	15896868	19510502	1	20111117	5
34681511	20131021	18028877	19490510	1	20131021	5
34879511	20120813	16695021	19281211	1	20120813	4
34907012	20130226	17281992	19690910	2	20130226	6
34941747	20130109	17149537	19680311	1	20130109	5
35038289	20110829	15661600	19531011	1	20110829	4
35279324	20131209	18190172	19570211	1	20131209	6
35356071	20130412	17429327	19500910	1	20130412	4
35434812	20130918	17929611	19520314	1	20130918	4
35455108	20110616	15449400	19481120	2	20110616	5
35520060	20110531	15397354	19500402	2	20110531	5
35565787	20120510	16412055	19530123	2	20120510	4
35745790	20111002	15756764	19610118	2	20111002	4
35767498	20120301	16196297	19670615	1	20120301	5
35824556	20120403	16297009	19540415	1	20120403	4
35872630	20120915	16793998	19590701	1	20120915	6
36167461	20120224	16180753	19610902	2	20120224	6
36170157	20110514	15356292	19550126	1	20110514	4
36195401	20131210	18191340	19501025	2	20131210	5
36236836	20110625	15473502	19630922	2	20110625	4

36335929	20120331	16284948	19650817	1	20120331	6
3635298420130721		17738312	19341109	1	20130721	5
36355574	20111202	15940810	19460408	1	20111202	4
36389103	20110829	15661622	19610122	2	20110829	4
3639051920130823		17844103	20040730	1	20130823	5
36405604	20111109	15874190	19770712	2	20111109	4
3641438720130313		17336520	19731128	2	20130313	4
3645340420130416		17440381	19591118	2	20130416	6
36456801	20110517	15363775	19590207	1	20110517	5
36689846	20120308	16223178	19421230	1	20120308	5
36781110	20120209	16135761	19500816	2	20120209	6
36805659	20120829	16737551	19600529	2	20120829	6
3682169920130803		17780504	19660520	1	20130803	4
3695276820130418		17447977	19560730	2	20130418	4
3698959020130104		17132033	19570213	1	20130104	6
37030298	20111104	15859141	19550406	1	20111104	6
37052236	20120305	16209419	19640331	2	20120305	5
37066516	20120128	16094033	19760915	2	20120128	5
3708395720131205		18177346	19370112	2	20131205	4
37101818	20120419	16346514	19390929	1	20120419	6
37124008	20110831	15666478	19500518	2	20110831	5
37236058	20120717	16613272	19640825	1	20120717	6
37271913	20120906	16766728	19510319	1	20120906	5
37318631	20120511	16415433	19371118	1	20120511	5
37332095	20120404	16297132	19430104	2	20120404	6
37351396	20110930	15753501	19741106	2	20110930	5
37366895	20120328	16278339	19631021	2	20120328	6
37372295	20120608	16497087	19371204	2	20120608	6
3739652420130416		17440346	19440103	1	20130416	4
37399761	20110826	15656808	19290713	2	20110826	4
37420023	20120118	16079540	19260505	1	20120118	5
37436025	20121205	17041618	19510920	1	20121205	4
37450183	20120217	16162057	19430122	2	20120217	4
37466696	20111002	15756704	19591031	2	20111002	4
37512235	20110913	15706622	19720118	1	20110913	6
37550906	20120120	16084367	19710228	1	20120120	4
37559630	20110909	15700036	19591206	1	20110909	5
37623522	20111021	15817882	19670424	2	20111021	5
37668034	20110502	15315368	19591202	1	20110502	5
37669639	20121109	16961116	19440626	1	20121109	5
37717058	20120809	16683562	19631120	2	20120809	4
3772976320130918		17927927	19490716	2	20130918	6
37762768	20121211	17062988	19560815	1	20121211	6
37767514	20110729	15572695	19971003	1	20110729	4
37780726	20111109	15874475	19571230	1	20111109	4
37800398	20120730	16648684	19570222	1	20120730	5
3782151520130426		17469507	19450208	1	20130426	6
37825142	20110421	15287695	19590507	1	20110421	4
37849153	20111209	15961023	19590208	1	20111209	5
37850934	20121007	16858478	19570406	1	20121007	5
3787274520130218		17252399	19430606	1	20130218	5
37873420	20120219	16164417	19540103	2	20120219	6
37946068	20111202	15940185	19620108	1	20111202	6
37970584	20110910	15701660	19610115	2	20110910	5
3797073320130129		17204415	19631124	1	20130129	5

37977472 20110803	15590827	19460902	1	20110803	6
37994857 20110926	15741219	19661020	1	20110926	4
38032698 20110617	15453792	19681010	2	20110617	6
3804758420130414	17431844	19510320	2	20130414	6
38048714 20111002	15756392	19660718	1	20111002	5
3808681620131110	18094896	19400105	2	20131110	5
38113063 20120322	16263118	20001105	1	20120322	5
38116846 20120207	16127774	19771208	1	20120207	4
38124913 20120813	16692477	19610501	2	20120813	5
38125132 20110624	15472282	19510222	1	20110624	5
38127912 20120617	16521027	19351208	1	20120617	4
38129894 20111019	15812131	19590420	1	20111019	4
38130255 20120406	16308465	19390508	1	20120406	5
38139763 20121219	17088884	19550128	1	20121219	6
38162399 20120730	16648163	19650906	1	20120730	5
38181167 20110803	15590244	19641104	2	20110803	6
3818388920130220	17261933	19440925	1	20130220	5
38189310 20110912	15702411	19480410	1	20110912	6
38215155 20120129	16094379	19700531	2	20120129	4
38218881 20120217	16162403	19940317	1	20120217	6
38243651 20111216	15982701	19790218	1	20111216	4
38257420 20121221	17095042	19600916	1	20121221	6
3826198220130309	17323843	19480907	1	20130309	4
38293559 20111107	15866743	19541120	2	20111107	4
38312833 20111016	15800814	19710926	1	20111016	4
38318091 20120918	16801494	19730605	2	20120918	6
38324811 20120524	16450019	19351030	1	20120524	5
38347987 20111229	16016329	19480710	2	20111229	5
38350571 20120808	16678579	19620325	1	20120808	6
38360188 20111101	15841418	19590101	1	20111101	4
38369481 20120423	16355465	19630722	1	20120423	4
3837536920130310	17324332	19331212	1	20130310	6
38386593 20111214	15978229	19540816	1	20111214	6
3838830620130428	17471854	19660716	1	20130428	6
38402152 20111031	15840893	19591030	1	20111031	5
38435120 20120103	16031623	19660414	2	20120103	6
38477202 20120326	16270112	19390109	1	20120326	4
38486907 20111116	15894701	19950929	2	20111116	6
38497379 20120515	16424946	19381102	2	20120515	5
38502328 20111203	15942907	19771125	1	20111203	4
38556251 20121102	16935984	19490925	2	20121102	6
38562231 20120304	16203922	19500611	2	20120304	5
38575574 20111227	16012631	19601012	2	20111227	4
38575654 20120118	16078918	19530509	1	20120118	5
38641995 20120825	16729391	19751021	2	20120825	4
38642181 20120614	16515299	19510823	2	20120614	5
38652367 20120507	16399838	19290202	1	20120507	6
38654012 20120325	16268850	19500830	2	20120325	5
38663615 20120805	16665036	19540206	1	20120805	4
38677064 20120410	16317745	19671220	1	20120410	4
38683782 20120129	16094584	19630218	1	20120129	4
3870092420130406	17406632	19951104	1	20130406	5
38702102 20120207	16129216	19481130	2	20120207	4
38714113 20120313	16237630	19550623	2	20120313	5
38731758 20120705	16576474	19631006	2	20120705	5

38751085	20120814	16698948	19540701	1	20120814	5
38782808	20130202	17220000	19730313	1	20130202	6
38804056	20130501	17482688	19650203	1	20130501	5
38810116	20130610	17609450	19340210	1	20130610	5
38812827	20130408	17408708	19621015	1	20130408	5
38833453	20121027	16916654	19590614	1	20121027	4
38833942	20121003	16845442	19550907	2	20121003	5
38853064	20120203	16114316	19831003	1	20120203	4
38856018	20120822	16722609	19670114	2	20120822	6
38865666	20120514	16421590	19601003	1	20120514	4
38908100	20120428	16369087	19801122	2	20120428	4
38966642	20130728	17757772	19500810	1	20130728	6
38973852	20120908	16772702	19641222	2	20120908	4
38986775	20120426	16365039	19740519	1	20120426	4
39012196	20121023	16905199	19850713	2	20121023	4
39016405	20130713	17715556	19900523	1	20130713	6
39040863	20120804	16664502	19500323	2	20120804	5
39045197	20120709	16588804	19370114	1	20120709	4
39051871	20121018	16893689	19630120	1	20121018	4
39096907	20130521	17548436	19571227	2	20130521	4
39098378	20121106	16947353	20070101	2	20121106	4
39133669	20120906	16766638	19770828	1	20120906	4
39136884	20130825	17847550	19530315	2	20130825	6
39162793	20130307	17317783	19560719	1	20130307	6
39165269	20121206	17044441	19940908	1	20121206	5
39165849	20121005	16855567	19630911	2	20121005	6
39178626	20120920	16808465	19740812	2	20120920	6
39180911	20130712	17714838	19590617	1	20130712	6
39183965	20121006	16857838	20010529	2	20121006	5
39186011	20121227	17109422	19500830	2	20121227	5
39190028	20130226	17281404	19550626	2	20130226	6
39190675	20130813	17815361	19710902	2	20130813	6
39202250	20130212	17243700	19540614	2	20130212	4
39214567	20121003	16847414	19860811	1	20121003	4
39217533	20130614	17623226	19490531	2	20130614	6
39217760	20131005	17981689	19480522	2	20131005	6
39227582	20131012	18002555	19410411	1	20131012	6
39251064	20120924	16817654	19790518	1	20120924	5
39263757	20120910	16776957	19570801	2	20120910	6
39285284	20130129	17206659	19380913	1	20130129	4
39288034	20130218	17255211	19700817	1	20130218	4
39297035	20130509	17511290	19681117	1	20130509	5
39341532	20130903	17877279	19560226	1	20130903	4
39367016	20131016	18016206	19561021	2	20131016	5
39392693	20130426	17469968	19530804	1	20130426	4
39407379	20130218	17254815	19551127	2	20130218	6
39486265	20131218	18219481	19540819	1	20131218	5
39517232	20130528	17567373	19680108	1	20130528	5
39530682	20131025	18042450	19591014	1	20131025	4
39588799	20130407	17407361	19580622	2	20130407	6
39595169	20130930	17958007	19500826	1	20130930	5
39627333	20130116	17171664	19660420	1	20130116	4
39631986	20130506	17500410	19540323	2	20130506	6
39647591	20130426	17469865	19961219	1	20130426	6
39652670	20130510	17516957	19500619	2	20130510	4

3966571920130620	17641851	19750307	1	20130620	5
3969151520130423	17460086	19480724	1	20130423	5
3973256420130823	17844599	19680225	1	20130823	5
3977050820130626	17656449	19410813	1	20130626	5
3990085120130623	17647185	19860327	2	20130623	5
3990537820130610	17610402	19880611	1	20130610	6
3990948320131127	18148917	19590710	1	20131127	5
3993952120131229	18245911	19511121	2	20131229	4
3994052820131229	18245935	19920523	2	20131229	6
3997322320130922	17934017	19550330	1	20130922	4
4005708720131029	18052599	19570803	1	20131029	5
4007533020130729	17758725	19641005	1	20130729	6
4019119320131215	18208628	19671225	2	20131215	6
4019183120130721	17738156	19571229	2	20130721	4
4019832120131213	18205381	19401005	1	20131213	6
4022088020131201	18156484	19541209	1	20131201	4
4022315220131210	18194950	19420529	1	20131210	5
4025172720130912	17911442	19520913	1	20130912	5
4027637720131127	18148190	19530905	1	20131127	4
4050057020131129	18154843	19491116	2	20131129	6
4053557720131223	18231311	19661023	1	20131223	5
59767 20121024	16909023	19421027	2	20121024	5
9004020130513	17521945	19321207	1	20130513	4
122634 20120412	16328102	19280506	1	20120412	5
429152 20120510	16411741	19301224	1	20120510	4
73946820131128	18152232	19420310	1	20131128	6
133426520130204	17223247	19281201	1	20130204	5
1445665 20120516	16425501	19510223	2	20120516	4
1575264 20120910	16776780	19381211	1	20120910	4
1947144 20110616	15450451	19220729	1	20110616	5
203509820130225	17277108	19360105	1	20130225	6
2251947 20120627	16548162	19491107	2	20120627	4
2516134 20120806	16670476	19580107	1	20120806	6
262435320130314	17338674	19300222	2	20130314	6
297245420130505	17495920	19400126	2	20130505	6
3683747 20121203	17028248	19590923	2	20121203	5
551804720131217	18215135	19230506	1	20131217	5
644511020130415	17433286	19420811	2	20130415	6
6766967 20120217	16161772	19560208	2	20120217	4
689434420130803	17780175	19520419	1	20130803	6
7060846 20120826	16729692	19541215	2	20120826	6
7695525 20111031	15840933	19510818	1	20111031	6
796638320121228	17113515	19500423	1	20121228	6
8003063 20120104	16036894	19500401	1	20120104	6
8348821 20120126	16090573	19351211	1	20120126	6
839517320130329	17382056	19471128	2	20130329	5
891453220130227	17285627	19470210	1	20130227	6
9582507 20120128	16094207	19591111	2	20120128	6
9685954 20120828	16735804	19250703	1	20120828	6
10225793 20120721	16626343	19540815	1	20120721	4
10271495 20120716	16610898	19430820	1	20120716	4
10746288 20120220	16167714	19500818	1	20120220	6
1175243520130121	17184545	19410405	2	20130121	6
12210592 20120601	16471930	19441021	1	20120601	4
1248146820130930	17958288	19531020	2	20130930	4

12670643	20110615	15447004	19470117	1	20110615	5
12906615	20120915	16794047	19440628	2	20120915	4
12965423	20120427	16367647	19381220	1	20120427	4
12974866	20120630	16556460	19421021	1	20120630	4
13019506	20120702	16563073	19580324	1	20120702	6
13342944	20111220	15993078	19330909	2	20111220	5
13723972	20111016	15800964	19350725	2	20111016	6
14104257	20110913	15704889	19271008	2	20110913	4
14183950	20131119	18125915	19550910	2	20131119	5
14241431	20130818	17829263	19601018	1	20130818	5
14844747	20130314	17339527	19260928	1	20130314	5
14863219	20120117	16076186	19481214	2	20120117	6
15525903	20111223	16002710	19220320	1	20111223	6
15651328	20130905	17887301	19560109	2	20130905	6
15679206	20121004	16851797	19521015	1	20121004	5
15733025	20121108	16957132	19310126	1	20121108	4
15752417	20120613	16511744	19371207	2	20120613	5
15759383	20120710	16593084	19680929	1	20120710	6
15798353	20131226	18241366	19540610	1	20131226	5
16000514	20130223	17273487	19600528	1	20130223	6
16289206	20111024	15822774	19250425	2	20111024	5
16636127	20111206	15952664	19310302	1	20111206	4
16887255	20111028	15835774	19550124	2	20111028	6
17104379	20110518	15367409	19720811	2	20110518	5
17237897	20130307	17317661	19351020	2	20130307	6
17292432	20120803	16662328	19680902	2	20120803	4
17617708	20111226	16009279	19250715	1	20111226	6
17735614	20130516	17535630	19650717	2	20130516	5
17789423	20120511	16415298	19330216	2	20120511	6
18748599	20120323	16266732	19510403	1	20120323	4
19228332	20130409	17414918	19510806	1	20130409	5
19825220	20130415	17435462	19320327	1	20130415	4
19858470	20130102	17123309	19620910	1	20130102	5
20008440	20130129	17205690	19701013	2	20130129	6
20216084	20120603	16474450	19560809	2	20120603	4
20489138	20130319	17353238	19420214	2	20130319	6
20663267	20120808	16679752	19290430	1	20120808	4
20677865	20130308	17321757	19571008	1	20130308	5
20828879	20130603	17582186	19391205	1	20130603	6
21207341	20120110	16053651	19380806	1	20120110	4
21746247	20110919	15723044	19700830	2	20110919	4
22148034	20130711	17711659	19330601	1	20130711	5
22319988	20121217	17080832	19320827	1	20121217	5
22361713	20130627	17660964	19300918	1	20130627	6
22562056	20120131	16099594	19340406	1	20120131	4
22578274	20130610	17610227	19250110	1	20130610	4
23084653	20120908	16772535	19680118	2	20120908	6
23253874	20120507	16399246	19491130	1	20120507	6
23265647	20121023	16905955	19361120	1	20121023	4
23356992	20111201	15934209	19710725	1	20111201	4
23797571	20110905	15683571	19610130	2	20110905	6
24424848	20130602	17578782	19260302	1	20130602	4
24534314	20120803	16660649	19261025	1	20120803	4
24633689	20130801	17771127	19410401	1	20130801	6
24817672	20130315	17342863	19490404	2	20130315	5

24956896	20120722	16627090	19640922	1	20120722	5
25445583	20111224	16004253	19620116	1	20111224	6
25587706	20110823	15647993	19500905	2	20110823	6
25733635	20131030	18055581	19370124	2	20131030	5
25810597	20130623	17647193	19631121	2	20130623	5
26213467	20130703	17682430	19570503	2	20130703	5
26232199	20121022	16903105	19580102	1	20121022	4
26632995	20130830	17863274	19381023	1	20130830	5
27198272	20130922	17934069	19411225	2	20130922	5
27219096	20121123	17004202	19500225	1	20121123	5
27487634	20110816	15629864	19311019	2	20110816	6
27535860	20130224	17274010	19650907	2	20130224	4
27680666	20120710	16593091	19591226	2	20120710	4
27806059	20130920	17932052	19330616	2	20130920	5
27869187	20111011	15786185	19521228	1	20111011	5
28158103	20130421	17453363	19651124	1	20130421	4
28616628	20131023	18035852	19561214	1	20131023	6
28989788	20110627	15475860	19800702	1	20110627	4
29454997	20120429	16369388	19460603	1	20120429	6
29625670	20130425	17467012	19600220	1	20130425	5
29897390	20121010	16867979	19430501	2	20121010	4
29897754	20120216	16159267	19541023	2	20120216	6
29951813	20110720	15550024	19630923	2	20110720	6
30724255	20121106	16948519	19610906	1	20121106	6
31750046	20110526	15388006	19660908	2	20110526	6
32052678	20120722	16626937	19551125	2	20120722	6
32579434	20121125	17006343	19281026	1	20121125	6
32665422	20131009	17995946	19510521	1	20131009	6
32818498	20111123	15913608	19651207	1	20111123	4
32866914	20130611	17613169	19410916	1	20130611	6
33022718	20131209	18187198	19421001	1	20131209	5
33034912	20131105	18076433	19601108	1	20131105	5
33289644	20130830	17862147	19490410	2	20130830	4
33487795	20110921	15729334	19441221	2	20110921	5
33576613	20110921	15730492	19671121	1	20110921	4
33780766	20130203	17220629	19580308	1	20130203	5
33981554	20120206	16124615	19650923	2	20120206	5
34042563	20131118	18122193	19680706	1	20131118	4
34141510	20121224	17100330	19651208	2	20121224	6
34189952	20120709	16588558	19421125	1	20120709	4
34449020	20130203	17220637	19520210	1	20130203	4
34488396	20120108	16047495	19640224	1	20120108	4
34524157	20130428	17471744	19600417	1	20130428	5
34740142	20120301	16195411	19410402	1	20120301	5
34831339	20130417	17444066	19321214	1	20130417	5
34905721	20130607	17602369	19490125	1	20130607	5
34941747	20130806	17791469	19680311	1	20130806	6
35184282	20130529	17570256	19610214	1	20130529	5
35212954	20130805	17786229	19460514	1	20130805	4
35238143	20120712	16600959	19561223	1	20120712	4
35386528	20120602	16474082	19630127	1	20120602	6
35400227	20130526	17560504	19661213	2	20130526	6
35687162	20120325	16268872	19700809	2	20120325	4
35797967	20120510	16412173	19540208	2	20120510	6
35843835	20110820	15640618	19681031	2	20110820	6

35957701	20130426	17469748	19591202	1 20130426	6
35969461	20130418	17447973	19630520	1 20130418	5
35993545	20120731	16650554	19611202	2 20120731	5
35999576	20120321	16260837	19580303	1 20120321	4
36073684	20120103	16031198	19590108	2 20120103	6
36170157	20110601	15403706	19550126	1 20110601	4
36186342	20111208	15960781	19640523	2 20111208	6
36236836	20110831	15666955	19630922	2 20110831	4
36420812	20130502	17489262	19550102	2 20130502	5
36456801	20110609	15428900	19590207	1 20110609	4
36624449	20111223	16003894	19630502	1 20111223	6
36693922	20111224	16005338	19580111	2 20111224	6
36703954	20121112	16967812	19520701	1 20121112	4
36757456	20120404	16298863	19560310	2 20120404	4
36762251	20111221	15998066	19331126	2 20111221	6
36795616	20131028	18048097	19550502	2 20131028	4
36802274	20130204	17225940	19600301	1 20130204	6
36880474	20131006	17982010	19640310	1 20131006	4
36887997	20120916	16794376	19491229	1 20120916	4
36935270	20120221	16170630	19930412	2 20120221	4
36994420	20110615	15447029	19551201	1 20110615	5
37041455	20131024	18040820	19721219	2 20131024	6
37063380	20120326	16272044	19670221	2 20120326	4
37076703	20120814	16698963	19520515	2 20120814	6
37124008	20110914	15709892	19500518	2 20110914	4
37155207	20110501	15309361	19580413	2 20110501	6
37213131	20111018	15808099	19690519	1 20111018	4
37324917	20111209	15963982	19510613	2 20111209	6
37351396	20111110	15877764	19741106	2 20111110	5
37374462	20120226	16182614	19490529	1 20120226	4
37395689	20120914	16792079	19600531	2 20120914	6
37437653	20111002	15756635	19590106	2 20111002	5
37499233	20130106	17135460	19520816	2 20130106	5
37537216	20130803	17779214	19560821	2 20130803	5
37550906	20120205	16119905	19710228	1 20120205	6
37559630	20111021	15818458	19591206	1 20111021	6
37577927	20130325	17369323	19461109	2 20130325	5
37584580	20130130	17209203	19731202	1 20130130	4
37621026	20120323	16266722	19770117	1 20120323	6
37698741	20110826	15656243	19471225	2 20110826	5
37762768	20130117	17175317	19560815	1 20130117	5
37791212	20120207	16129192	19690930	1 20120207	4
37825142	20110503	15318965	19590507	1 20110503	5
37873420	20120508	16404190	19540103	2 20120508	5
37899288	20130918	17929392	19470916	1 20130918	4
37922477	20120420	16349962	19531218	1 20120420	4
37922853	20120304	16204174	19570722	1 20120304	5
37929707	20120310	16229166	19440401	1 20120310	4
37968904	20121129	17019499	19410922	1 20121129	5
37973425	20130623	17647289	19520902	1 20130623	5
37995270	20110519	15370516	19620908	1 20110519	6
38046730	20111215	15980529	19920219	1 20111215	5
38047584	20130501	17481523	19510320	2 20130501	6
38051659	20120406	16307364	19490712	1 20120406	4
38086974	20120513	16417788	19550109	1 20120513	4

3808735320131012		18002729	19600512	2	20131012	5
38123114	20120506	16394699	19470818	2	20120506	5
38131827	20111026	15830597	20090818	1	20111026	4
38174833	20121010	16867980	19870606	1	20121010	4
38196177	20120311	16229776	19490517	1	20120311	4
3819702320121211		17060357	19490511	1	20121211	6
3826401620130805		17784261	19340625	2	20130805	6
3828277820130619		17635324	19490526	1	20130619	5
38323681	20120109	16051945	19951212	1	20120109	4
38346951	20120313	16236992	19430203	2	20120313	6
38348355	20111227	16011650	19400118	1	20111227	5
38350571	20120928	16830046	19620325	1	20120928	6
38360188	20121106	16947405	19590101	1	20121106	6
3836265120130628		17663901	19620530	2	20130628	6
38369481	20120527	16454888	19630722	1	20120527	6
38402878	20111225	16005684	19780302	1	20111225	4
3845883220130109		17149570	19540220	2	20130109	4
38473722	20121119	16989932	19380306	1	20121119	4
38516642	20120815	16702306	19760829	2	20120815	4
38521787	20111209	15963856	19761130	2	20111209	5
3852228020130919		17930005	19680616	2	20130919	6
38524695	20120626	16545405	19661216	1	20120626	5
38546360	20120205	16119916	19481018	1	20120205	5
38562128	20120513	16417537	19541002	1	20120513	4
38562231	20120731	16650870	19500611	2	20120731	6
38563267	20120315	16245206	19611126	1	20120315	5
38575574	20120320	16257654	19601012	2	20120320	4
3858066420130106		17135439	19280908	1	20130106	4
38593349	20120515	16422593	19930310	1	20120515	5
38625035	20120510	16412140	19380404	1	20120510	4
38626710	20121106	16947224	19580508	1	20121106	4
38627224	20120908	16772576	19771002	1	20120908	6
38652367	20120527	16454775	19290202	1	20120527	6
38656643	20120322	16263934	19650211	2	20120322	6
3865961920131108		18092736	19430813	1	20131108	5
38660785	20121110	16963042	19660601	1	20121110	5
38681264	20120209	16135193	19461229	1	20120209	4
38713132	20120302	16201171	19801004	2	20120302	6
38751085	20120828	16736206	19540701	1	20120828	4
38757276	20120620	16530275	19761126	1	20120620	4
38785556	20120317	16249939	19590302	2	20120317	5
38836941	20120521	16439133	19920612	2	20120521	4
3883988220121230		17115243	19600115	1	20121230	5
38889995	20120826	16729744	19520421	1	20120826	5
38891202	20120624	16537859	19591010	1	20120624	4
38894803	20120816	16706609	19581114	1	20120816	5
3896830820130308		17321320	19700904	2	20130308	6
38973852	20120922	16814021	19641222	2	20120922	5
3897633920130115		17167938	19380227	2	20130115	6
3899556120131130		18154961	19380423	1	20131130	4
39027946	20121112	16967141	19650529	1	20121112	5
39034736	20120705	16575965	19500118	1	20120705	4
39037837	20120626	16545408	19640301	2	20120626	4
3904086320131219		18221569	19500323	2	20131219	4
39045197	20120731	16651293	19370114	1	20120731	5

39061477	20130526	17560505	19511106	2	20130526	4
39078654	20130831	17864524	19610115	2	20130831	4
39097411	20121112	16967842	20100922	2	20121112	5
39102528	20131218	18219887	19570411	2	20131218	6
39136884	20131108	18091241	19530315	2	20131108	6
39151310	20130627	17661091	19600906	2	20130627	5
39165269	20130102	17123359	19940908	1	20130102	5
39178693	20121116	16983287	19550415	1	20121116	6
39209784	20131001	17964562	19400628	2	20131001	4
39232923	20121228	17113002	19590325	1	20121228	5
39250389	20121104	16938844	19570228	1	20121104	4
39280723	20131106	18082213	19540113	2	20131106	4
39292712	20130503	17492341	19661031	1	20130503	4
39326108	20130920	17932068	19630512	2	20130920	4
39331174	20130116	17171064	19700912	2	20130116	6
39392693	20130610	17608738	19530804	1	20130610	5
39435331	20121219	17088518	19591020	1	20121219	5
39500599	20130114	17160324	19390312	2	20130114	6
39526119	20130204	17223789	19741014	1	20130204	4
39561676	20130306	17312858	19730205	2	20130306	6
39569307	20130218	17255500	19890409	2	20130218	6
39606150	20131226	18241408	19481215	1	20131226	6
39613893	20130425	17467027	19560430	2	20130425	5
39614067	20130921	17933338	19580922	1	20130921	5
39653720	20130819	17833299	19600224	1	20130819	5
39687359	20130613	17619359	19351129	1	20130613	6
39698174	20130811	17806902	19731010	1	20130811	4
39698572	20130807	17795691	19530218	2	20130807	4
39729754	20130518	17540683	19520915	1	20130518	6
39732917	20130611	17613582	19800203	1	20130611	6
39881680	20130709	17703032	19860325	2	20130709	6
39913218	20130614	17623232	19641130	2	20130614	5
39953963	20131020	18025775	19620719	2	20131020	6
39972242	20130604	17588747	19570512	2	20130604	5
39981516	20130715	17720494	19460701	1	20130715	4
39989996	20130711	17711830	19580324	1	20130711	4
40028351	20130812	17811281	19710809	1	20130812	5
40074826	20131017	18020353	19520910	1	20131017	4
40178505	20131023	18037086	19390324	1	20131023	5
40231956	20131223	18230530	19641130	2	20131223	4
40270346	20131129	18154836	19600618	2	20131129	4
40282948	20131202	18162838	19580228	2	20131202	6
40460999	20131220	18225606	19551002	2	20131220	5
40518567	20131225	18238233	19481026	1	20131225	5
89656	20120803	16660232	19260426	1	20120803	6
607396	20120712	16600953	19641114	2	20120712	5
780267	20110501	15309151	19400422	2	20110501	4
1575264	20121002	16842289	19381211	1	20121002	5
1891550	20130422	17455165	19301011	1	20130422	4
2010711	20120611	16504434	19461008	2	20120611	6
2470475	20120413	16331071	19440608	1	20120413	4
2559297	20111129	15928046	19671109	2	20111129	6
2832759	20121222	17097101	19250310	1	20121222	5
3248644	20120326	16271888	19520208	2	20120326	6
4088975	20120803	16660979	19500314	2	20120803	4

4398576	20110826	15657237	19521001	2	20110826	4
4791202	20120325	16268794	19341017	2	20120325	4
5293538	20130426	17469319	19290128	2	20130426	5
6445110	20130613	17617280	19420811	2	20130613	4
6456855	20130811	17806668	19251215	1	20130811	4
6723315	20121221	17093751	19500128	1	20121221	6
6766967	20120223	16177188	19560208	2	20120223	6
7289216	20120422	16351973	19401117	2	20120422	5
7804220	20130609	17605556	19290325	1	20130609	5
8914532	20130402	17392451	19470210	1	20130402	5
9286651	20131109	18094569	19511029	2	20131109	5
9392169	20120916	16794337	19500601	2	20120916	5
10234261	20130108	17141022	19511011	1	20130108	5
10271495	20120730	16648689	19430820	1	20120730	6
11032545	20120824	16727472	19351207	2	20120824	5
11101465	20120830	16740790	19510916	1	20120830	6
12370104	20120420	16349858	19590217	2	20120420	5
12731429	20131226	18241151	19391009	1	20131226	5
12756013	20111108	15870576	19691125	2	20111108	6
13345681	20120208	16133333	19690114	1	20120208	5
13642347	20111017	15805000	19480512	1	20111017	4
13989816	20120501	16375092	19310202	1	20120501	4
14031539	20120827	16732431	19591024	2	20120827	6
14046107	20130407	17407487	19440708	2	20130407	4
14199689	20131016	18015056	19401226	2	20131016	5
14512039	20130612	17615010	19260208	2	20130612	4
15164768	20130728	17757857	19330412	1	20130728	4
15626003	20130418	17447843	19380903	2	20130418	6
15759383	20120726	16640725	19680929	1	20120726	6
16021128	20120307	16219238	19490824	2	20120307	6
16084530	20130709	17703075	19600224	1	20130709	6
16100031	20120424	16358834	19530928	2	20120424	4
16376800	20120221	16170792	19770111	1	20120221	5
16415477	20120316	16248370	19350915	2	20120316	4
16766500	20120413	16331583	19390510	1	20120413	6
17094814	20120805	16664979	19550910	2	20120805	4
17104379	20110808	15605031	19720811	2	20110808	6
17237897	20130507	17504079	19351020	2	20130507	4
17617708	20120131	16101739	19250715	1	20120131	5
19602790	20120629	16554060	19880622	1	20120629	5
20050026	20130918	17928647	19630102	2	20130918	6
20239649	20130728	17757530	19321228	1	20130728	6
20663267	20120818	16711356	19290430	1	20120818	6
20912452	20130406	17406771	19430819	1	20130406	4
21034979	20110521	15374759	19770425	2	20110521	4
21101008	20120629	16553969	19270501	1	20120629	5
21326501	20130904	17880005	19530315	1	20130904	6
21461494	20130619	17636146	19350908	1	20130619	4
21638404	20120423	16355096	19430624	2	20120423	6
21655958	20130720	17737748	19601105	1	20130720	6
21844188	20130419	17451153	19650828	2	20130419	4
21946872	20111231	16021823	19410223	1	20111231	5
23092866	20120510	16412039	19471110	2	20120510	4
23101635	20120313	16237741	19510825	2	20120313	5
23103799	20120215	16155626	19430606	1	20120215	4

23198109	20110616	15450528	19340101	1	20110616	4
2327271120131118		18121324	19670820	2	20131118	4
23356992	20120130	16096482	19710725	1	20120130	6
23879621	20120329	16280628	19281031	1	20120329	5
24906103	20120706	16581046	19631101	1	20120706	6
2558650920130805		17786262	19630221	2	20130805	6
25631510	20120629	16554874	19451014	1	20120629	6
2573363520131113		18108420	19370124	2	20131113	6
26013229	20120409	16314830	19550512	2	20120409	4
27482004	20120130	16096365	19470103	2	20120130	4
27680666	20120720	16624837	19591226	2	20120720	4
2783535420130422		17456941	19710112	2	20130422	6
2811008920130701		17669782	19650328	1	20130701	6
2827753420121230		17115166	19710519	1	20121230	4
2838374620131107		18086368	19350226	2	20131107	6
2861662820131126		18145461	19561214	1	20131126	4
2862411520131215		18208828	19630220	1	20131215	5
28749317	20120321	16260904	19570117	1	20120321	5
2914721520130325		17369595	20000912	1	20130325	4
2938564620130621		17644018	19450215	2	20130621	5
29526690	20120326	16271082	19610922	2	20120326	4
30160384	20110917	15718159	19701017	1	20110917	4
3029521720130926		17949107	19611120	1	20130926	5
3041347920131016		18016148	19510501	2	20131016	4
30427919	20110925	15738350	19540516	1	20110925	4
30436852	20120705	16577964	19250721	1	20120705	4
30587258	20120429	16369553	19621203	1	20120429	5
30619751	20111219	15990376	19500920	2	20111219	4
3071511820130529		17570267	19421027	1	20130529	6
3072425520130104		17132783	19610906	1	20130104	6
30759263	20120702	16563064	19770826	1	20120702	4
3092699920130330		17383312	19690216	2	20130330	5
31152622	20120919	16804883	19511218	1	20120919	5
31244925	20121021	16899269	19460421	1	20121021	4
31703829	20121111	16963331	19541220	1	20121111	4
3186250720131121		18133089	19390119	1	20131121	5
3233233520131118		18120405	19380128	1	20131118	6
3247158220130227		17285644	19380420	2	20130227	5
3257943420130130		17209488	19281026	1	20130130	5
32818910	20121116	16982091	19660620	1	20121116	4
3297314120130327		17375854	19710629	1	20130327	5
3297555620131212		18201205	19411121	1	20131212	6
33118331	20110610	15432406	19420927	2	20110610	5
33816852	20120805	16664883	19540422	1	20120805	5
3408953720130314		17340137	19300327	1	20130314	5
34128466	20120215	16155728	19760221	2	20120215	4
34205851	20120814	16699091	19411208	1	20120814	4
3421875220131107		18087289	19350310	1	20131107	6
3430561820130312		17332518	19760513	1	20130312	4
34488396	20120328	16278121	19640224	1	20120328	5
3452415720130723		17744929	19600417	1	20130723	5
3481526420130121		17184586	19670801	1	20130121	4
3483133920130429		17474070	19321214	1	20130429	6
3494174720131028		18049312	19680311	1	20131028	5
35097531	20110530	15393871	19610221	2	20110530	6

35105727	20130121	17184106	19641010	2	20130121	4
35218407	20130607	17603158	19641212	2	20130607	4
35235655	20131230	18248412	19561102	2	20131230	6
35472210	20111210	15965832	19430605	1	20111210	6
35565787	20120608	16497559	19530123	2	20120608	4
35687162	20120401	16285654	19700809	2	20120401	4
35843835	20110914	15710348	19681031	2	20110914	4
35876983	20120112	16063946	19521101	2	20120112	4
35932811	20120419	16347185	19470212	1	20120419	4
35982866	20120517	16432053	19681016	2	20120517	5
36016598	20131029	18049794	19600501	1	20131029	4
36094663	20120404	16299685	19501012	1	20120404	6
36236836	20110906	15688329	19630922	2	20110906	4
36250201	20111108	15870642	19570628	1	20111108	5
36256618	20120409	16314869	19410628	2	20120409	5
36276570	20110819	15638984	19400831	2	20110819	5
36387118	20120515	16424562	19781020	2	20120515	6
36414387	20130516	17535708	19731128	2	20130516	6
36456801	20110627	15477238	19590207	1	20110627	5
36456834	20111223	16003857	19470401	2	20111223	5
36463920	20130913	17915127	19361113	1	20130913	4
36774240	20131206	18180967	19501012	1	20131206	6
36846545	20120202	16112497	20000922	1	20120202	4
37013095	20120130	16098284	19970605	1	20120130	6
37033399	20120418	16343223	19551001	2	20120418	4
37213131	20111207	15957225	19690519	1	20111207	6
37225062	20120504	16392697	19580610	1	20120504	5
37236058	20120909	16773063	19640825	1	20120909	4
37272096	20111107	15866842	19591113	1	20111107	4
37273362	20130222	17271918	19440701	2	20130222	6
37287380	20110815	15625993	20001222	1	20110815	4
37324917	20120101	16022421	19510613	2	20120101	6
37355207	20120422	16351957	19660311	2	20120422	6
37371612	20121114	16975863	19680626	2	20121114	4
37374304	20120719	16621710	19440527	2	20120719	4
37464292	20120229	16189342	19950805	1	20120229	6
37500291	20120308	16222404	19650420	1	20120308	4
37568368	20111122	15910639	19591025	2	20111122	6
37597630	20120603	16474772	19500226	2	20120603	5
37623908	20120316	16248408	19501123	2	20120316	6
37686810	20110803	15589780	19441223	1	20110803	6
37749738	20120925	16820764	19751215	1	20120925	5
37804152	20111227	16011957	19590401	1	20111227	6
37839626	20120610	16500265	19340623	2	20120610	4
37882885	20131017	18019241	19760119	2	20131017	5
37896052	20120423	16355420	19470520	1	20120423	4
37922477	20120531	16466610	19531218	1	20120531	4
37927121	20120607	16493933	19500221	2	20120607	4
37932482	20131103	18066822	20071009	1	20131103	4
37968904	20121224	17100904	19410922	1	20121224	6
37970584	20111120	15903499	19610115	2	20111120	6
37970733	20130610	17610204	19631124	1	20130610	6
37995270	20110601	15403473	19620908	1	20110601	4
38002107	20121211	17062308	19590427	1	20121211	4
38018610	20110717	15539262	19850504	1	20110717	5

38047584	20130619	17638158	19510320	2	20130619	5
38048714	20111106	15862690	19660718	1	20111106	6
38050441	20111107	15866807	19600822	2	20111107	5
38107925	20130914	17916859	19550416	1	20130914	6
38127912	20121209	17053336	19351208	1	20121209	5
38130255	20120611	16501178	19390508	1	20120611	5
38137085	20120424	16358861	19560420	1	20120424	5
38171721	20120520	16437168	19470817	1	20120520	6
38189627	20110913	15705952	19360604	2	20110913	5
38252403	20110921	15730431	20090901	1	20110921	4
38260536	20120226	16182882	19561118	1	20120226	4
38271840	20121129	17019641	19400523	2	20121129	5
38293559	20120101	16022555	19541120	2	20120101	4
38348355	20120314	16240548	19400118	1	20120314	6
38385227	20120513	16417760	19420212	1	20120513	4
38416998	20120113	16067577	19630630	2	20120113	5
38419000	20121029	16920354	19530120	1	20121029	4
38435755	20130928	17954345	19221112	2	20130928	6
38540975	20120219	16164266	19590628	2	20120219	6
38572871	20120917	16798506	19280517	2	20120917	6
38576293	20111129	15928216	19940410	1	20111129	4
38580664	20130122	17185738	19280908	1	20130122	4
38584075	20130515	17531259	19651005	1	20130515	4
38660912	20121120	16994203	19800718	2	20121120	6
38663193	20120504	16390372	19640925	2	20120504	4
38681253	20130330	17383385	19710109	2	20130330	5
38681264	20120213	16146966	19461229	1	20120213	4
38702102	20120325	16268853	19481130	2	20120325	6
38703638	20121218	17084961	19660801	1	20121218	4
38705076	20131011	18000335	20000918	1	20131011	4
38713132	20120323	16266170	19801004	2	20120323	5
38724695	20121212	17064142	19531018	1	20121212	5
38729156	20120131	16101329	19750213	2	20120131	5
38756819	20130924	17942078	19520828	2	20130924	4
38757276	20120709	16588746	19761126	1	20120709	4
38770240	20120518	16434972	19520102	2	20120518	4
38782808	20130429	17475180	19730313	1	20130429	4
38805628	20130913	17915257	19641228	2	20130913	4
38833942	20121121	16996889	19550907	2	20121121	6
38846070	20120725	16637597	19840920	2	20120725	6
38853064	20120217	16161670	19831003	1	20120217	4
38866578	20120601	16469849	19550303	2	20120601	6
38877735	20120916	16794501	19741128	1	20120916	5
38880385	20120905	16762281	19470419	1	20120905	4
38913961	20121009	16865703	19370214	1	20121009	5
38954517	20120803	16662271	19540303	2	20120803	4
38972291	20130519	17541062	19520420	1	20130519	6
38974651	20120707	16583935	19530710	1	20120707	5
38985738	20120915	16793946	19460626	2	20120915	6
39012196	20121128	17016417	19850713	2	20121128	4
39037359	20121120	16994058	19830905	2	20121120	6
39045197	20120901	16746510	19370114	1	20120901	4
39060225	20120906	16764700	19791115	1	20120906	6
39095324	20130107	17140585	19380610	1	20130107	4
39151310	20130710	17707616	19600906	2	20130710	5

3920978420131022		18031460	19400628	2	20131022	6
39219868	20120916	16794529	19420915	1	20120916	5
3922857420130329		17382013	19471110	2	20130329	4
3927963520130126		17199760	19680517	1	20130126	6
3928477020130819		17832907	19621117	2	20130819	5
3929271220130522		17551862	19661031	1	20130522	5
3930635920121224		17100296	19751208	2	20121224	5
3934138320131025		18044025	19950712	2	20131025	6
39343856	20121202	17025327	19571230	1	20121202	5
3935491120130304		17300208	19491105	1	20130304	4
3937629920130630		17665552	19480101	2	20130630	6
3940209020130727		17757287	19510311	2	20130727	5
3941535520130506		17500604	20100302	2	20130506	6
3943533120130220		17264172	19591020	1	20130220	6
3951887120130527		17564030	19530520	1	20130527	6
3959297720130121		17182927	19911110	1	20130121	4
3961406720131107		18083884	19580922	1	20131107	5
3961879620130413		17431342	19740813	1	20130413	6
3963961720130302		17296328	19640415	1	20130302	5
3965372020130829		17859651	19600224	1	20130829	5
3966295820130812		17810514	20060224	2	20130812	5
3968735920130704		17687024	19351129	1	20130704	6
3969731920131102		18066235	20020128	2	20131102	5
3972975420130618		17633772	19520915	1	20130618	6
3973291720130622		17646539	19800203	1	20130622	6
3991321820130624		17650964	19641130	2	20130624	4
4001193620130830		17863318	19880207	2	20130830	5
4001686220130911		17907388	19530114	1	20130911	5
4003051120130803		17780632	19921024	1	20130803	5
4013105320130806		17790045	19550928	2	20130806	6
4014175120130930		17958260	19570805	1	20130930	4
4027034620131222		18228363	19600618	2	20131222	4
184010	20111230	16020854	19250916	1	20111230	5
822300	20120830	16741955	19491010	2	20120830	5
1298653	20120724	16632844	19501124	2	20120724	4
1311246	20120329	16280773	19381222	2	20120329	6
144566520130804		17780843	19510223	2	20130804	6
2516134	20120915	16794072	19580107	1	20120915	4
357066920130328		17378175	19710809	1	20130328	5
431179920130103		17127964	19490315	1	20130103	4
644511020130709		17700999	19420811	2	20130709	6
672331520130104		17132779	19500128	1	20130104	6
725529020131011		18000799	19380113	2	20131011	5
770374420130411		17423963	19371008	2	20130411	6
891453220130423		17460351	19470210	1	20130423	4
928665120131129		18153872	19511029	2	20131129	5
10006890	20121222	17097011	19530306	2	20121222	5
1003104620130801		17770786	19460214	2	20130801	4
1075496820130501		17484052	19520820	2	20130501	6
11324337	20110911	15702074	19431121	1	20110911	4
1149505920130303		17296992	19540204	1	20130303	4
1348567120131221		18227661	19290826	1	20131221	5
1565132820130920		17932470	19560109	2	20130920	6
1565732620130104		17132799	19340416	2	20130104	6
16415477	20120414	16333227	19350915	2	20120414	6

16766500	20120502	16382221	19390510	1	20120502	4
17963507	20120203	16117017	19670202	1	20120203	5
18012889	20120601	16470789	19570328	1	20120601	4
18334311	20120618	16523628	19531112	1	20120618	4
1833641920131218		18220023	19561115	1	20131218	4
19636987	20120318	16250748	19381210	2	20120318	4
19731818	20121028	16917119	19360117	2	20121028	5
2005002620131019		18025405	19630102	2	20131019	4
2023964920130920		17931877	19321228	1	20130920	6
2091245220130415		17434962	19430819	1	20130415	6
21960838	20120426	16364974	19370116	2	20120426	4
2202317220130519		17541121	19371017	2	20130519	5
22514790	20111212	15970553	19410120	2	20111212	4
2291445020130104		17132666	19470502	2	20130104	6
22936318	20120303	16203454	19580827	1	20120303	4
2311474120131003		17973606	19541201	2	20131003	6
2315656120130111		17157323	19620607	2	20130111	6
2323013920131226		18239444	19581208	2	20131226	4
23356992	20120530	16462468	19710725	1	20120530	6
23367819	20120430	16372956	19580201	2	20120430	5
2558650920130821		17838024	19630221	2	20130821	6
2643928120131010		17997299	19751228	2	20131010	5
27240013	20111025	15827318	19651207	2	20111025	4
2745384520130305		17307152	19481008	1	20130305	5
2787171220130723		17745739	19420716	1	20130723	4
28181295	20110913	15706661	19460211	2	20110913	4
2830095020130312		17331472	19651122	1	20130312	5
2838374620131230		18248830	19350226	2	20131230	4
28749317	20120830	16741273	19570117	1	20120830	6
28763362	20120212	16143405	19690601	2	20120212	4
29194794	20121226	17106592	19500301	2	20121226	6
2962567020130526		17560454	19600220	1	20130526	5
30002007	20120902	16746978	19670116	2	20120902	6
3042791920130226		17281153	19540516	1	20130226	5
30587258	20120527	16454708	19621203	1	20120527	6
3059289320130413		17431247	19560514	1	20130413	5
30725225	20120507	16399806	19681220	2	20120507	5
30759263	20120724	16633881	19770826	1	20120724	5
31319058	20111211	15966615	19790916	1	20111211	6
31430418	20120203	16117252	19461125	1	20120203	4
31703829	20121208	17052793	19541220	1	20121208	4
31894970	20111227	16011809	19641124	2	20111227	5
32198064	20120501	16379214	19370427	2	20120501	6
32402101	20120401	16285510	19371022	1	20120401	5
32506288	20120330	16283717	19470310	1	20120330	5
32818498	20120106	16045066	19651207	1	20120106	5
32939836	20120201	16107953	19470905	1	20120201	6
33371914	20120829	16738148	19510922	1	20120829	6
3408286520130505		17495963	19270505	2	20130505	4
34128466	20120317	16250171	19760221	2	20120317	4
34278847	20111013	15793912	19600120	2	20111013	6
3430561820130423		17460148	19760513	1	20130423	4
34803333	20120130	16096704	19600119	2	20120130	6
3514562120130425		17466087	19571124	1	20130425	4
35343943	20110811	15616121	19321108	1	20110811	6

35351112	20110523	15378958	19500415	2	20110523	4
35386528	20120705	16576739	19630127	1	20120705	4
35465964	20130624	17650899	19441130	1	20130624	5
35565787	20120619	16528272	19530123	2	20120619	4
35687162	20120408	16311111	19700809	2	20120408	5
35797967	20120701	16557111	19540208	2	20120701	6
35843404	20120424	16357273	19650606	1	20120424	6
35932811	20120514	16420808	19470212	1	20120514	6
36276570	20110902	15674957	19400831	2	20110902	5
36330286	20130602	17578455	19320315	1	20130602	5
36420812	20131022	18032255	19550102	2	20131022	5
36456061	20110723	15557497	19981028	2	20110723	5
36609753	20111015	15800430	19370811	2	20111015	5
36612949	20110430	15308846	19660806	2	20110430	4
36762900	20120126	16090588	19560915	2	20120126	5
37016345	20121026	16914530	19621104	1	20121026	4
37066516	20120813	16693756	19760915	2	20120813	6
37124008	20111014	15798804	19500518	2	20111014	4
37250876	20110514	15356312	19570309	1	20110514	6
37265875	20111124	15916688	19440821	2	20111124	5
37367376	20110607	15419906	19790529	1	20110607	4
37371612	20121227	17109873	19680626	2	20121227	6
37374508	20131017	18020247	19691012	1	20131017	5
37389336	20120911	16781168	19461111	2	20120911	4
37398779	20130831	17864663	19410903	1	20130831	6
37431677	20120315	16244443	19410905	2	20120315	4
37466696	20121212	17067014	19591031	2	20121212	5
37474843	20120906	16766658	19570303	2	20120906	6
37550406	20121011	16871596	19961105	1	20121011	6
37559630	20120107	16047017	19591206	1	20120107	6
37568368	20111130	15930675	19591025	2	20111130	4
37597630	20131103	18066860	19500226	2	20131103	5
37641966	20131023	18037117	19601119	2	20131023	4
37693064	20120104	16036848	19871215	2	20120104	4
37694614	20120629	16554884	19571212	2	20120629	4
37698741	20111019	15812069	19471225	2	20111019	5
37708080	20111101	15842398	19830430	2	20111101	6
37749738	20121028	16917099	19751215	1	20121028	5
37792066	20120111	16059654	19801028	2	20120111	4
37840350	20130902	17866824	19490325	1	20130902	5
37873475	20130710	17707173	19400409	1	20130710	4
37891637	20111128	15924439	19420222	1	20111128	5
37922477	20121117	16983639	19531218	1	20121117	6
37922853	20120401	16285594	19570722	1	20120401	5
37973425	20130909	17898660	19520902	1	20130909	6
38022854	20111113	15883277	19400102	1	20111113	5
38048714	20111226	16008302	19660718	1	20111226	5
38053666	20111223	16003432	19600126	2	20111223	4
38062872	20111107	15865079	19590301	2	20111107	4
38073084	20130930	17956947	19620802	1	20130930	6
38087875	20111112	15882920	19410824	2	20111112	5
38134257	20120228	16187621	19681212	1	20120228	4
38134677	20121028	16917343	19570317	1	20121028	4
38150286	20111109	15874079	19961021	1	20111109	6
38220405	20120802	16657738	19560521	1	20120802	6

38252630	20120229	16189991	19680502	1	20120229	6
38274532	20130221	17268609	19570402	2	20130221	5
38369481	20120711	16596588	19630722	1	20120711	4
38375643	20120223	16177760	19730224	1	20120223	5
38396995	20130925	17945420	19460625	2	20130925	5
38497506	20130504	17495557	19510801	1	20130504	5
38502328	20120105	16041326	19771125	1	20120105	4
38546360	20130604	17589330	19481018	1	20130604	6
38561783	20120325	16268534	19990919	2	20120325	4
38564237	20121118	16985932	19750727	1	20121118	6
38572871	20130512	17519411	19280517	2	20130512	5
38584075	20130721	17738394	19651005	1	20130721	4
38627224	20121007	16858246	19771002	1	20121007	5
38631060	20130326	17373252	19581015	2	20130326	4
38635733	20130529	17570272	19620620	1	20130529	4
38645077	20120817	16709525	19681009	1	20120817	4
38681253	20130406	17406772	19710109	2	20130406	4
38683782	20120406	16308440	19630218	1	20120406	5
38721061	20120424	16358898	19880707	1	20120424	5
38756819	20131003	17974399	19520828	2	20131003	6
38770240	20120601	16470212	19520102	2	20120601	4
38811039	20121108	16957110	19471101	1	20121108	5
38835868	20130106	17135392	19551106	1	20130106	4
38846070	20120810	16688271	19840920	2	20120810	5
38848576	20130310	17324131	19500713	1	20130310	5
38891202	20120825	16729315	19591010	1	20120825	6
38892205	20131121	18133120	19470924	1	20131121	4
38894803	20120919	16805967	19581114	1	20120919	5
38974651	20120721	16626508	19530710	1	20120721	4
38989923	20131022	18032176	19671008	2	20131022	4
39015139	20130909	17897871	19580302	1	20130909	6
39068150	20121015	16881644	19581228	1	20121015	4
39077004	20121209	17053200	19640902	1	20121209	4
39154933	20131205	18177132	19671226	2	20131205	6
39202250	20130529	17569603	19540614	2	20130529	4
39280450	20130413	17431231	19360518	1	20130413	5
39292712	20130529	17570271	19661031	1	20130529	6
39315509	20130327	17375566	19500220	1	20130327	5
39331174	20130224	17274054	19700912	2	20130224	6
39341383	20131127	18148399	19950712	2	20131127	6
39359289	20130311	17328676	19400716	1	20130311	4
39373905	20130427	17471388	19570204	1	20130427	6
39435331	20131121	18132663	19591020	1	20131121	4
39552335	20130421	17453398	19390127	2	20130421	6
39580433	20130911	17906696	19670422	2	20130911	6
39592977	20130203	17220432	19911110	1	20130203	5
39596413	20130512	17519218	19491015	1	20130512	5
39610532	20130503	17493333	19770328	1	20130503	4
39614067	20131114	18111348	19580922	1	20131114	5
39634101	20131018	18023133	19580711	2	20131018	4
39645788	20131006	17982124	19520109	2	20131006	5
39687359	20130803	17780379	19351129	1	20130803	6
39732917	20130704	17687142	19800203	1	20130704	6
39737070	20131031	18057089	19951129	2	20131031	4
39774602	20130905	17887329	20050726	2	20130905	5

3981265220130723		17744531	19571206	1	20130723	4
3987358020131208		18185227	19560827	2	20131208	5
3988754220130823		17845261	19450119	2	20130823	6
3991894020130904		17879261	19521108	1	20130904	6
3992165820131007		17987006	19990318	1	20131007	5
4002835120131106		18083751	19710809	1	20131106	6
4007802220131001		17963788	19520825	2	20131001	4
4014175120131012		18002791	19570805	1	20131012	5
4021872220131111		18099874	19510217	2	20131111	4
4040621120131118		18122218	19550313	1	20131118	5
13283220131112		18100450	19460408	1	20131112	6
2251947	20121210	17056277	19491107	2	20121210	4
297245420130813		17815122	19400126	2	20130813	6
3248644	20120508	16404162	19520208	2	20120508	6
4791202	20120910	16776125	19341017	2	20120910	4
500336120131113		18108291	19481108	1	20131113	5
6066031	20120111	16057197	19460220	2	20120111	5
675323720131016		18015335	19300922	1	20131016	5
702064620130523		17554567	19581021	2	20130523	6
770374420130603		17583898	19371008	2	20130603	4
815435620130428		17471654	19510327	1	20130428	5
911885820131006		17982279	19330221	2	20131006	4
1023426120131206		18182581	19511011	1	20131206	4
1188596420130710		17707328	19521029	1	20130710	4
1248146820131205		18178496	19531020	2	20131205	6
12756013	20120131	16101719	19691125	2	20120131	5
1318302320130321		17359253	19290205	1	20130321	6
1336323020130626		17657881	19620701	2	20130626	5
1337489520130930		17957942	19411018	1	20130930	5
1449059620131128		18152108	19450602	2	20131128	5
1451203920130721		17738482	19260208	2	20130721	5
14823155	20120528	16458105	19630729	2	20120528	5
15303001	20121028	16917142	19340804	2	20121028	4
1573302520130417		17443068	19310126	1	20130417	5
16021128	20120805	16665001	19490824	2	20120805	6
16376800	20120319	16254277	19770111	1	20120319	4
16526424	20121008	16862937	19480210	1	20121008	5
1686467820131104		18072377	19460602	2	20131104	4
17294029	20120924	16818012	19480127	1	20120924	5
18012889	20120706	16581717	19570328	1	20120706	6
18236083	20111129	15928045	19540502	2	20111129	4
1863984420130531		17575692	19500520	2	20130531	6
18936373	20110824	15651254	19550520	2	20110824	6
1985847020130822		17841200	19620910	1	20130822	5
19939709	20121010	16867900	19311227	1	20121010	4
2005002620131117		18118339	19630102	2	20131117	4
21011814	20111007	15780128	19400921	1	20111007	4
2235441220130310		17324434	19740906	1	20130310	5
22701184	20120713	16604056	19511122	1	20120713	6
24175777	20111126	15921310	19391112	2	20111126	6
24305224	20111112	15882886	19381013	2	20111112	5
24906103	20121205	17041571	19631101	1	20121205	5
25129468	20120409	16315716	19521105	1	20120409	5
2613064320130304		17300732	19420630	2	20130304	5
26993793	20120923	16814583	19550707	1	20120923	5

27453845	20130413	17431071	19481008	1	20130413	4
28181295	20111105	15862123	19460211	2	20111105	5
28214655	20120511	16412244	19530622	2	20120511	5
28277534	20130213	17244252	19710519	1	20130213	4
29781815	20120224	16180715	19320405	1	20120224	6
30002007	20121009	16866207	19670116	2	20121009	5
30618430	20120524	16449978	19381216	1	20120524	4
30759263	20121125	17006362	19770826	1	20121125	6
30926999	20130521	17548182	19690216	2	20130521	5
31430418	20120229	16190523	19461125	1	20120229	4
31703829	20121229	17114443	19541220	1	20121229	6
31894970	20120103	16032253	19641124	2	20120103	6
32733689	20131029	18052532	19430610	2	20131029	6
33952384	20121108	16956620	19371214	1	20121108	4
34223944	20120217	16161768	19291012	1	20120217	5
34264341	20111226	16009276	19510502	1	20111226	6
34278847	20111230	16020483	19600120	2	20111230	6
34438136	20120517	16432040	19481015	1	20120517	4
34488396	20120511	16414736	19640224	1	20120511	5
34548000	20130622	17646643	19690409	1	20130622	4
35843857	20120211	16142778	19330212	1	20120211	5
35957701	20130517	17538719	19591202	1	20130517	5
35989969	20121217	17081650	19320530	2	20121217	4
36042850	20131121	18133049	19491012	1	20131121	4
36072249	20130708	17698632	19650903	2	20130708	6
36236836	20110921	15730502	19630922	2	20110921	4
36612949	20110506	15332910	19660806	2	20110506	6
36746551	20130515	17531466	19531014	1	20130515	4
36907832	20111216	15984947	19730802	2	20111216	6
37066516	20120828	16735186	19760915	2	20120828	6
37124008	20111028	15836239	19500518	2	20111028	6
37155207	20110809	15608878	19580413	2	20110809	4
37161403	20121113	16971217	19710108	1	20121113	5
37287380	20111003	15761659	20001222	1	20111003	5
37324917	20120207	16129130	19510613	2	20120207	5
37334784	20120203	16117044	19560117	2	20120203	5
37431677	20120412	16327534	19410905	2	20120412	5
37466696	20130124	17194822	19591031	2	20130124	5
37470181	20130922	17934075	19370225	1	20130922	4
37489046	20120721	16626331	19441128	1	20120721	4
37500291	20120421	16351409	19650420	1	20120421	4
37559630	20120220	16165671	19591206	1	20120220	5
37572819	20120429	16369697	19710702	2	20120429	4
37694614	20120708	16584620	19571212	2	20120708	4
37715290	20130818	17829345	19560126	1	20130818	6
37749738	20130818	17828903	19751215	1	20130818	4
37769190	20110416	15272273	19611023	1	20110416	6
37771485	20120404	16297605	19501016	1	20120404	6
37792066	20120129	16094799	19801028	2	20120129	6
37922853	20120415	16333756	19570722	1	20120415	6
37931376	20120918	16801594	19450512	1	20120918	6
37973425	20131106	18083774	19520902	1	20131106	5
37995270	20110616	15449993	19620908	1	20110616	6
38002107	20130128	17201502	19590427	1	20130128	5
38005935	20111114	15887019	19931010	1	20111114	4

38072047	20120525	16452430	19981015	2	20120525	5
38118842	20120202	16112923	19550919	2	20120202	4
38134257	20120507	16399855	19681212	1	20120507	5
38174833	20131107	18088573	19870606	1	20131107	6
38210901	20120616	16520478	19471209	2	20120616	4
38224338	20130610	17610316	19580410	2	20130610	6
38260536	20120604	16479862	19561118	1	20120604	4
38261982	20130626	17657807	19480907	1	20130626	4
38271102	20131103	18066671	19620610	1	20131103	4
38274532	20130408	17409986	19570402	2	20130408	4
38320466	20121230	17115138	19580228	1	20121230	4
38358304	20120210	16140557	19350806	2	20120210	4
38396995	20131103	18066909	19460625	2	20131103	6
38405173	20121008	16862521	19351028	2	20121008	6
38428772	20120707	16583892	19460927	1	20120707	6
38546360	20130704	17686741	19481018	1	20130704	4
38584075	20131218	18219936	19651005	1	20131218	6
38602050	20130511	17518623	19691228	2	20130511	6
38658274	20120404	16299643	19661214	1	20120404	4
38721061	20120601	16471979	19880707	1	20120601	4
38751085	20121009	16867393	19540701	1	20121009	4
38756819	20131022	18032699	19520828	2	20131022	6
38757276	20120801	16656552	19761126	1	20120801	4
38814301	20120615	16518358	19771113	2	20120615	4
38866090	20130929	17954828	19590811	2	20130929	6
38882632	20130509	17513673	19570225	1	20130509	6
38891202	20121005	16852798	19591010	1	20121005	4
38914839	20130907	17893429	19480812	1	20130907	6
38974651	20120806	16670407	19530710	1	20120806	5
39004370	20121113	16970944	19620310	1	20121113	6
39077004	20121218	17085540	19640902	1	20121218	5
39098378	20130311	17328427	20070101	2	20130311	6
39126982	20130710	17706156	19571227	1	20130710	6
39225746	20130304	17302621	19620410	1	20130304	6
39237893	20130104	17131706	19450613	2	20130104	6
39265480	20130722	17742086	19500502	1	20130722	6
39268525	20130302	17296238	19681203	1	20130302	4
39288034	20130416	17440191	19700817	1	20130416	5
39326040	20130304	17300704	19531222	1	20130304	5
39328513	20130420	17452920	19600210	1	20130420	4
39402603	20130925	17945754	19680613	1	20130925	5
39435331	20131223	18229280	19591020	1	20131223	5
39539532	20130216	17250024	19620610	2	20130216	4
39541305	20130620	17641865	19970728	1	20130620	5
39595603	20130924	17938780	20080703	2	20130924	6
39647591	20130810	17806018	19961219	1	20130810	5
39655157	20130501	17483847	19621215	1	20130501	5
39687359	20130827	17854661	19351129	1	20130827	5
39721692	20130704	17687125	19980807	1	20130704	5
39792171	20130608	17604795	19570612	1	20130608	6
39812652	20131103	18066628	19571206	1	20131103	5
39905378	20131106	18081848	19880611	1	20131106	4
39913218	20130715	17720541	19641130	2	20130715	6
39963183	20130715	17720021	19500923	1	20130715	6
40053096	20130825	17847371	19601205	2	20130825	4

40406211	20131202	18162826	19550313	1	20131202	5
780267	20110522	15375496	19400422	2	20110522	6
1298653	20130216	17250199	19501124	2	20130216	6
1445665	20131031	18057528	19510223	2	20131031	6
2106936	20120406	16308370	19400816	1	20120406	4
2251947	20130116	17171707	19491107	2	20130116	4
3559146	20120315	16245193	19491013	1	20120315	4
3696911	20120813	16695074	19370220	1	20120813	6
4398576	20111126	15921399	19521001	2	20111126	6
4638613	20130513	17523724	19570215	2	20130513	6
4807630	20130521	17548378	19470901	1	20130521	6
5846355	20131008	17989049	19270608	1	20131008	4
6469870	20130913	17914401	19470401	2	20130913	4
7596465	20131028	18049025	19540216	2	20131028	6
8348821	20120403	16297010	19351211	1	20120403	5
8914532	20130713	17715325	19470210	1	20130713	6
9065285	20130209	17242216	19471228	1	20130209	4
9599897	20130307	17317434	19400712	1	20130307	5
10568328	20120913	16787355	19430915	2	20120913	6
10853197	20130221	17268385	19550225	1	20130221	4
11024672	20120621	16533236	19590417	2	20120621	4
11032545	20130118	17178278	19351207	2	20130118	5
12134837	20130108	17145297	19411115	1	20130108	4
12756013	20120219	16164462	19691125	2	20120219	6
13019506	20120826	16729887	19580324	1	20120826	6
14031539	20121126	17010114	19591024	2	20121126	6
14793885	20121017	16887370	19330107	1	20121017	4
15258534	20130317	17345764	19530912	2	20130317	4
16669106	20121013	16877817	19260820	2	20121013	4
16803080	20131124	18138150	19521101	2	20131124	5
16864678	20131112	18104579	19460602	2	20131112	4
17244143	20120515	16425408	19400101	2	20120515	4
17261186	20121001	16836826	19520511	2	20121001	6
17735614	20130829	17860937	19650717	2	20130829	6
18334311	20120806	16668873	19531112	1	20120806	4
20515804	20130422	17455682	19571030	1	20130422	4
20737706	20120117	16075715	19630522	2	20120117	4
21525100	20130430	17478403	19591027	2	20130430	6
21631209	20120709	16588674	19491002	1	20120709	4
22611150	20121216	17077295	19440822	1	20121216	6
24101175	20120326	16271825	19500325	1	20120326	6
24301551	20130905	17885950	19571031	1	20130905	5
24596585	20130826	17851075	19610907	2	20130826	5
24906103	20121224	17100900	19631101	1	20121224	6
25129468	20120420	16349938	19521105	1	20120420	4
25586509	20131106	18081364	19630221	2	20131106	6
27172925	20120204	16119365	19651215	2	20120204	5
27240013	20111213	15974022	19651207	2	20111213	6
27738154	20130507	17504955	19480108	1	20130507	5
27835354	20130626	17657338	19710112	2	20130626	5
28300950	20130707	17693727	19651122	1	20130707	6
30334124	20130602	17578403	19480901	2	20130602	4
30413479	20131211	18199190	19510501	2	20131211	4
30841135	20120815	16703023	19641226	2	20120815	5
31058692	20131113	18108735	19620411	1	20131113	6

31750046	20110919	15723276	19660908	2	20110919	4
3197291520130417		17443480	19621112	2	20130417	6
32402101	20120709	16588784	19371022	1	20120709	4
3347911720130804		17781230	19720122	1	20130804	5
33557301	20120424	16358071	19600106	2	20120424	6
3388276720130314		17339768	19490125	2	20130314	6
3430561820130621		17643317	19760513	1	20130621	6
34488396	20120624	16537760	19640224	1	20120624	5
34964835	20120628	16551484	19480520	2	20120628	5
3502640520130928		17952803	20070712	1	20130928	5
35143498	20120313	16237788	19340707	1	20120313	4
35210505	20120318	16250664	19940503	1	20120318	5
35395096	20111026	15830568	19390409	1	20111026	5
35472210	20120302	16200407	19430605	1	20120302	4
35520060	20110916	15715609	19500402	2	20110916	4
3553975420131001		17958306	19660512	2	20131001	4
35565787	20120827	16733116	19530123	2	20120827	4
35843404	20121203	17028408	19650606	1	20121203	5
3589650520130925		17945755	19510415	1	20130925	4
3595770120130524		17557924	19591202	1	20130524	5
36116913	20120106	16044863	20020712	2	20120106	5
3623306420130327		17375551	19631210	2	20130327	4
36609753	20120220	16167074	19370811	2	20120220	5
36907832	20120220	16168078	19730802	2	20120220	6
37041284	20120217	16162369	19570926	1	20120217	4
3707670320130109		17149563	19520515	2	20130109	4
37416970	20120322	16263914	19480111	2	20120322	5
3750002020130324		17366101	19581117	2	20130324	4
37500291	20120507	16399356	19650420	1	20120507	4
3768087820130620		17641027	19440809	2	20130620	6
37693064	20120224	16180422	19871215	2	20120224	5
37696596	20120911	16780997	19561024	2	20120911	5
37724428	20120313	16237699	19411213	1	20120313	5
37792066	20120208	16133075	19801028	2	20120208	6
37829713	20120616	16520058	19750101	1	20120616	6
37922853	20120506	16395134	19570722	1	20120506	4
37929707	20120927	16827529	19440401	1	20120927	5
38034581	20110825	15652905	19900329	2	20110825	4
38053666	20120110	16056089	19600126	2	20120110	6
3807462320130628		17663178	19450125	1	20130628	4
38134677	20121218	17082800	19570317	1	20121218	4
3814652020131109		18094623	19651015	1	20131109	4
38189627	20111019	15811195	19360604	2	20111019	4
38214027	20120226	16182810	19540609	1	20120226	6
3823275620130505		17496289	19611115	2	20130505	5
3826198220130721		17738428	19480907	1	20130721	6
38369481	20121002	16841411	19630722	1	20121002	5
38401762	20120829	16739159	19371129	1	20120829	5
3840517320130425		17466359	19351028	2	20130425	6
3841376220130922		17934033	19500320	1	20130922	4
38428772	20120827	16733194	19460927	1	20120827	5
38432723	20120118	16081022	19490420	1	20120118	6
38486907	20120316	16248279	19950929	2	20120316	5
38524695	20120927	16826091	19661216	1	20120927	6
3854636020130715		17716269	19481018	1	20130715	6

38605719	20131214	18208230	19691019	2 20131214	4
38656052	20130708	17698675	19401120	1 20130708	5
38660821	20130318	17349523	19600901	1 20130318	5
38660912	20130703	17682217	19800718	2 20130703	5
38687580	20130319	17353292	19450220	2 20130319	4
38751085	20121024	16909498	19540701	1 20121024	5
38770240	20120821	16719296	19520102	2 20120821	5
38773647	20130425	17466022	19691119	2 20130425	6
38801115	20130301	17294174	19580906	2 20130301	4
38809619	20130708	17698663	19760906	1 20130708	4
38835868	20130303	17296642	19551106	1 20130303	6
38842512	20130816	17825976	19640301	1 20130816	5
38851397	20130918	17928870	19990701	2 20130918	5
38875911	20120717	16614713	19690615	2 20120717	4
38891202	20130328	17378438	19591010	1 20130328	5
38897197	20130101	17117504	19730723	1 20130101	4
38979394	20121109	16960948	19730827	1 20121109	5
38998457	20130322	17363841	19530102	1 20130322	4
39098378	20130327	17374385	20070101	2 20130327	5
39101796	20121217	17081573	19501126	1 20121217	5
39202250	20130731	17767226	19540614	2 20130731	4
39258043	20130219	17258301	19590513	1 20130219	5
39261955	20121107	16952428	19710614	1 20121107	4
39402603	20131017	18020392	19680613	1 20131017	4
39407379	20130405	17405417	19551127	2 20130405	4
39532075	20130524	17558079	19550715	1 20130524	5
39588799	20130911	17905662	19580622	2 20130911	5
39746786	20131122	18135932	19490107	2 20131122	6
39872361	20130809	17804399	19880505	1 20130809	6
39989996	20130912	17911554	19580324	1 20130912	6
40028351	20131226	18241363	19710809	1 20131226	6
40135759	20131116	18117793	19490819	2 20131116	5
485085	20130719	17735084	19190808	1 20130719	5
2157359	20120318	16250519	19411013	2 20120318	4
2251947	20130219	17259955	19491107	2 20130219	4
2979717	20130427	17471319	19440218	1 20130427	6
3306363	20130622	17646755	19710317	2 20130622	6
5055356	20131121	18132486	19320921	1 20131121	4
7366134	20120128	16094078	19500102	2 20120128	5
7452439	20130530	17573015	19760602	1 20130530	5
7966383	20130422	17457069	19500423	1 20130422	6
8348821	20120424	16358830	19351211	1 20120424	6
9679112	20130824	17846949	19500116	2 20130824	6
11024672	20120628	16550830	19590417	2 20120628	6
11163114	20131124	18137912	19570815	1 20131124	5
11324337	20111015	15800149	19431121	1 20111015	5
11515483	20120403	16296906	19510304	2 20120403	6
12876947	20131027	18046147	19530628	2 20131027	6
13345681	20130715	17720509	19690114	1 20130715	4
14923343	20121113	16968866	19420223	2 20121113	4
16472154	20120530	16463503	19270304	2 20120530	4
16669106	20130111	17157093	19260820	2 20130111	6
17539865	20110619	15455898	19350527	1 20110619	5
17735614	20130923	17936574	19650717	2 20130923	6
17827268	20130507	17503348	19450717	2 20130507	6

17990782	20130714	17715712	19321125	2	20130714	6
18334311	20120820	16714059	19531112	1	20120820	4
18462867	20130620	17641740	19570907	1	20130620	5
18639844	20130816	17825798	19500520	2	20130816	6
19923592	20120806	16670184	19730309	2	20120806	4
20577291	20120925	16821257	19550520	1	20120925	4
21544456	20121225	17103895	19500317	1	20121225	4
21960838	20120612	16507927	19370116	2	20120612	4
22514790	20120112	16063302	19410120	2	20120112	4
22611150	20130706	17693251	19440822	1	20130706	6
22665243	20121122	16997993	19460829	2	20121122	6
24178561	20120816	16705589	19520705	2	20120816	4
24877290	20120221	16171646	19341113	2	20120221	5
25586509	20131215	18208473	19630221	2	20131215	6
26130643	20130603	17578845	19420630	2	20130603	5
26439281	20131121	18133057	19751228	2	20131121	5
27041607	20130401	17387290	19540504	2	20130401	4
27477618	20130603	17582882	19440319	1	20130603	5
28014779	20130722	17742048	19280608	2	20130722	4
29385646	20130915	17917442	19450215	2	20130915	6
30002007	20121122	17001095	19670116	2	20121122	4
30160384	20120329	16280730	19701017	1	20120329	4
31055159	20131125	18141165	19441027	1	20131125	5
31236610	20110821	15641307	19370917	2	20110821	5
31329018	20121117	16985318	19350615	2	20121117	4
33479117	20131108	18092668	19720122	1	20131108	5
33538464	20130812	17811306	19690425	2	20130812	4
33981554	20120514	16420813	19650923	2	20120514	5
34400232	20130202	17219679	19280726	1	20130202	5
34438136	20120708	16584485	19481015	1	20120708	5
34720791	20130609	17605413	19791212	2	20130609	5
35687162	20120701	16557091	19700809	2	20120701	4
35940251	20110925	15738483	19640711	2	20110925	4
35957701	20130531	17575647	19591202	1	20130531	4
35982866	20121202	17025190	19681016	2	20121202	4
36105370	20120824	16727586	19650719	2	20120824	6
36186342	20121021	16898959	19640523	2	20121021	6
36276570	20111018	15808314	19400831	2	20111018	6
36359816	20121016	16886108	19320124	1	20121016	4
36390199	20120810	16688184	19390403	1	20120810	6
36762900	20120416	16337170	19560915	2	20120416	5
36907832	20130611	17614648	19730802	2	20130611	5
37030049	20111217	15986719	19401209	1	20111217	4
37034905	20131115	18114421	19390220	2	20131115	6
37052236	20120507	16399780	19640331	2	20120507	6
37076703	20130215	17248644	19520515	2	20130215	6
37161403	20130612	17615058	19710108	1	20130612	4
37287380	20111109	15874361	20001222	1	20111109	5
37312871	20130528	17566910	19600810	2	20130528	6
37416970	20120421	16351516	19480111	2	20120421	4
37499233	20130625	17654007	19520816	2	20130625	6
37535287	20120202	16112881	19591026	2	20120202	5
37568368	20120206	16124646	19591025	2	20120206	5
37583736	20130103	17128536	19640527	2	20130103	5
37609760	20120415	16333579	19420113	1	20120415	5

37694614	20120819	16711896	19571212	2	20120819	4
37784206	20130716	17724434	19470717	2	20130716	5
37791212	20120418	16344024	19690930	1	20120418	4
37857479	20131112	18104550	20061003	1	20131112	5
37895888	20110629	15483102	19640521	1	20110629	5
37922853	20120520	16437063	19570722	1	20120520	5
37929707	20121019	16896969	19440401	1	20121019	5
37970584	20120404	16297171	19610115	2	20120404	4
37972046	20120102	16024414	19601219	2	20120102	4
38112946	20121226	17106761	19330505	1	20121226	4
38134257	20120612	16506158	19681212	1	20120612	4
38146520	20131116	18117814	19651015	1	20131116	4
38189627	20111129	15928033	19360604	2	20111129	5
38210843	20120103	16032287	19580914	1	20120103	6
38210901	20120715	16607018	19471209	2	20120715	6
38218881	20130116	17171948	19940317	1	20130116	4
38257420	20130420	17452731	19600916	1	20130420	6
38261982	20130816	17826969	19480907	1	20130816	5
38271840	20130810	17806243	19400523	2	20130810	6
38413762	20131015	18012130	19500320	1	20131015	6
38452129	20130212	17243567	19500103	2	20130212	4
38458832	20130326	17373232	19540220	2	20130326	4
38533776	20120402	16291633	19761113	2	20120402	5
38548673	20120601	16470066	20021210	1	20120601	5
38562231	20130115	17168160	19500611	2	20130115	6
38627224	20130313	17336434	19771002	1	20130313	5
38630896	20120908	16772266	19480301	1	20120908	6
38633293	20130321	17359618	19381022	1	20130321	4
38661915	20120429	16369524	19530731	2	20120429	4
38700924	20131001	17964491	19951104	1	20131001	5
38770240	20120827	16732319	19520102	2	20120827	6
38782808	20131011	18000460	19730313	1	20131011	6
38831582	20120723	16630283	19530830	2	20120723	5
38866578	20120922	16813893	19550303	2	20120922	6
38875739	20120715	16607023	19560310	2	20120715	4
38895668	20131203	18166068	19501208	1	20131203	4
38954517	20121109	16961047	19540303	2	20121109	4
39037359	20130223	17273614	19830905	2	20130223	5
39061477	20131026	18045657	19511106	2	20131026	5
39073546	20130805	17785855	19610120	1	20130805	4
39075951	20130301	17294088	19390316	1	20130301	5
39101796	20130226	17281951	19501126	1	20130226	5
39155709	20121103	16938205	19670413	1	20121103	4
39231486	20130617	17629721	19611211	1	20130617	5
39265480	20130806	17791486	19500502	1	20130806	6
39288034	20130516	17535549	19700817	1	20130516	4
39328513	20130911	17907495	19600210	1	20130911	4
39386077	20131111	18096157	19651204	2	20131111	5
39655157	20130617	17628711	19621215	1	20130617	4
39687359	20131009	17996794	19351129	1	20131009	5
39746786	20131127	18146217	19490107	2	20131127	5
39872361	20130913	17915253	19880505	1	20130913	4
39881840	20130716	17724592	19400605	1	20130716	4
40135759	20131211	18198312	19490819	2	20131211	5
2251947	20130326	17373194	19491107	2	20130326	4

283275920130324		17366116	19250310	1	20130324	5
297971720130603		17584301	19440218	1	20130603	5
304393420131204		18172409	19270219	1	20131204	4
4398576	20120116	16072560	19521001	2	20120116	4
642764120130802		17778272	19610110	1	20130802	5
745243920130611		17613199	19760602	1	20130611	6
911885820131228		18245410	19330221	2	20131228	4
957528420130319		17352889	19450517	1	20130319	5
1185653420131207		18184883	19370924	2	20131207	5
1334568120130805		17786199	19690114	1	20130805	4
16472154	20120812	16690451	19270304	2	20120812	4
1680308020131223		18231937	19521101	2	20131223	5
1686467820131226		18241340	19460602	2	20131226	5
16902424	20120928	16829192	19540915	2	20120928	5
1773561420131024		18040846	19650717	2	20131024	4
18089979	20120822	16722205	19660701	2	20120822	4
18936373	20111013	15795243	19550520	2	20111013	4
19923592	20120823	16724884	19730309	2	20120823	4
2004302120130325		17369459	19541212	2	20130325	5
2111119320130720		17737604	19481226	1	20130720	4
21279367	20120904	16752792	19581229	2	20120904	6
21631209	20120921	16810739	19491002	1	20120921	6
22648891	20120905	16762332	19470428	1	20120905	4
2309286620130826		17849544	19471110	2	20130826	6
24877290	20120328	16278146	19341113	2	20120328	6
2683461720130301		17294073	19350517	2	20130301	4
2711539520131202		18161978	19400428	2	20131202	6
27482286	20110520	15373721	19360909	2	20110520	4
2765721220131227		18243336	19590816	2	20131227	5
28091943	20121101	16929939	19990204	1	20121101	5
2837711920130429		17474351	19320210	2	20130429	4
2874931720130417		17444353	19570117	1	20130417	4
2938564620131007		17984678	19450215	2	20131007	4
2974721520130521		17547587	19530718	1	20130521	5
3092699920130708		17698466	19690216	2	20130708	4
31363152	20120301	16196649	19751022	1	20120301	4
3179200420130118		17177589	19701101	2	20130118	5
3239519020131124		18137983	19320626	1	20131124	4
32401528	20120113	16067537	19280405	1	20120113	5
32506288	20121105	16943663	19470310	1	20121105	6
3347368620130618		17633439	19380108	2	20130618	5
3388276720130501		17482027	19490125	2	20130501	6
34128466	20120714	16606085	19760221	2	20120714	6
3414672020131206		18182801	19401107	1	20131206	4
34488396	20120904	16756570	19640224	1	20120904	6
34803333	20120716	16609169	19600119	2	20120716	4
35520060	20111016	15800962	19500402	2	20111016	5
35565787	20121013	16876055	19530123	2	20121013	6
35589163	20120211	16142885	19981212	2	20120211	5
36068005	20121225	17104205	20030123	2	20121225	5
3607224920131205		18178437	19650903	2	20131205	6
36170157	20111022	15820053	19550126	1	20111022	6
3618634220130418		17446522	19640523	2	20130418	6
3670395420130110		17153749	19520701	1	20130110	4
36731450	20120325	16268694	19740304	2	20120325	6

36802274	20130610	17610487	19600301	1	20130610	6
36907832	20130725	17752685	19730802	2	20130725	6
37013540	20130720	17737628	19440531	1	20130720	6
37030049	20120615	16518218	19401209	1	20120615	4
37052236	20120519	16436649	19640331	2	20120519	5
37119770	20131202	18161785	19290121	2	20131202	4
37260029	20120721	16626327	19861001	2	20120721	5
37332095	20130604	17586878	19430104	2	20130604	4
37333598	20111030	15838076	19450427	2	20111030	4
37500020	20130528	17567260	19581117	2	20130528	5
37550406	20130108	17144954	19961105	1	20130108	5
37637119	20120313	16237855	19990901	1	20120313	5
37795667	20131128	18151641	19331025	1	20131128	5
37883684	20121126	17008965	19591212	1	20121126	4
38005935	20120130	16097905	19931010	1	20120130	4
38034478	20111125	15919942	19540530	1	20111125	4
38054670	20120129	16094782	19360415	1	20120129	5
38210843	20120902	16747058	19580914	1	20120902	5
38210901	20120804	16664602	19471209	2	20120804	5
38241291	20120528	16458114	19390712	1	20120528	4
38413762	20131101	18063763	19500320	1	20131101	6
38432723	20120215	16155809	19490420	1	20120215	4
38557312	20130527	17562821	19530126	2	20130527	5
38656552	20120615	16517563	19640101	1	20120615	5
38683657	20131020	18025860	19570826	2	20131020	4
38697200	20120526	16454300	19550930	1	20120526	4
38700924	20131028	18049345	19951104	1	20131028	6
38701869	20130503	17493726	19351001	1	20130503	6
38782808	20131108	18092150	19730313	1	20131108	4
38801115	20130526	17560492	19580906	2	20130526	5
38811039	20130113	17159853	19471101	1	20130113	6
38866578	20121007	16858119	19550303	2	20121007	5
38935738	20130415	17435569	19621130	2	20130415	4
38974651	20121025	16912513	19530710	1	20121025	6
39045653	20130917	17925914	19721005	1	20130917	4
39302040	20130506	17500366	19470821	2	20130506	4
39323687	20130419	17450244	19650302	1	20130419	4
39570246	20130812	17811271	19361118	2	20130812	6
39595170	20131020	18026010	19720802	2	20131020	6
39628643	20130911	17907197	19761108	2	20130911	6
39687359	20131113	18108791	19351129	1	20131113	5
39711381	20130907	17893531	19641113	1	20130907	5
39729754	20131106	18083710	19520915	1	20131106	5
39984935	20131014	18007440	19500625	1	20131014	4
40135759	20131229	18245912	19490819	2	20131229	4
485085	20130816	17825918	19190808	1	20130816	5
2251947	20130607	17602020	19491107	2	20130607	4
2832759	20130407	17407476	19250310	1	20130407	5
5065441	20130313	17336490	19550103	2	20130313	4
9065285	20130322	17363524	19471228	1	20130322	5
11108717	20130903	17875250	19550816	2	20130903	6
12725609	20130724	17749109	19470221	2	20130724	6
12876947	20131119	18125881	19530628	2	20131119	5
13019506	20121014	16878379	19580324	1	20121014	6
13345681	20130902	17871497	19690114	1	20130902	4

14098670	20130307	17317133	19540624	2	20130307	6
16644205	20120529	16461044	19661018	2	20120529	6
17215906	20110825	15654211	19570822	2	20110825	5
17244143	20120628	16552072	19400101	2	20120628	4
18089979	20130203	17220484	19660701	2	20130203	4
18236083	20120214	16151803	19540502	2	20120214	6
19682043	20111118	15901430	19310806	2	20111118	4
20043021	20130401	17389649	19541212	2	20130401	4
20570110	20120212	16143562	19490120	1	20120212	4
22648891	20120917	16798477	19470428	1	20120917	4
23797571	20120130	16098074	19610130	2	20120130	6
23874320	20120210	16139385	19530817	1	20120210	6
24139524	20120911	16781623	19690811	2	20120911	6
24625272	20130516	17535338	19251013	2	20130516	5
24654748	20130102	17120945	19940414	1	20130102	6
25257527	20130929	17954712	19570130	2	20130929	6
25631510	20130317	17345576	19451014	1	20130317	5
26013229	20120722	16626943	19550512	2	20120722	6
26993793	20130105	17134879	19550707	1	20130105	4
27027878	20130330	17383342	19500216	2	20130330	5
27477618	20131130	18156169	19440319	1	20131130	4
28377119	20130714	17715969	19320210	2	20130714	6
29625670	20130813	17815388	19600220	1	20130813	6
30759263	20130513	17523803	19770826	1	20130513	6
32067702	20131105	18077730	19420120	2	20131105	5
32916419	20120719	16621590	19540607	2	20120719	4
33318682	20121130	17022163	19680314	2	20121130	5
33576613	20111225	16005944	19671121	1	20111225	5
33981554	20120625	16541479	19650923	2	20120625	5
34444694	20120629	16554903	19611019	1	20120629	5
35960602	20120724	16634033	19460515	1	20120724	4
36256618	20121220	17091630	19410628	2	20121220	5
36583241	20130818	17829240	19570406	1	20130818	4
36846545	20120909	16773152	20000922	1	20120909	6
37572819	20130707	17693665	19710702	2	20130707	6
37583736	20130327	17375029	19640527	2	20130327	6
37584580	20130822	17840698	19731202	1	20130822	4
37605019	20111218	15987151	19620116	2	20111218	5
37708080	20120401	16285373	19830430	2	20120401	6
37769190	20110514	15356244	19611023	1	20110514	4
37834029	20120101	16022614	19910130	2	20120101	5
37922853	20120719	16621798	19570722	1	20120719	6
38218881	20130329	17382072	19940317	1	20130329	5
38241291	20120610	16500213	19390712	1	20120610	4
38261982	20131106	18083426	19480907	1	20131106	6
38656052	20130902	17871840	19401120	1	20130902	4
38664958	20130731	17767242	19421218	1	20130731	4
38785556	20121101	16931668	19590302	2	20121101	5
38846070	20121206	17046002	19840920	2	20121206	6
38875739	20120820	16715665	19560310	2	20120820	6
38936708	20120501	16378607	19561126	2	20120501	4
38954517	20121125	17006628	19540303	2	20121125	4
39004370	20130219	17259401	19620310	1	20130219	5
39137092	20121213	17071419	19740519	2	20121213	4
39149321	20130701	17670955	19601230	1	20130701	6

39177190	20130215	17248717	19580420	1	20130215	5
39261955	20121211	17062587	19710614	1	20121211	5
39265480	20131022	18033570	19500502	1	20131022	6
39323687	20130505	17496071	19650302	1	20130505	4
39539532	20131127	18148929	19620610	2	20131127	5
39648798	20131222	18228397	19751225	2	20131222	6
39729754	20131124	18138090	19520915	1	20131124	4
39910582	20131123	18137636	19601006	2	20131123	6
2832759	20130421	17453420	19250310	1	20130421	6
4141133	20131202	18162746	19580807	1	20131202	4
5912072	20120921	16812322	19560705	1	20120921	4
7019387	20120213	16147802	19590201	2	20120213	4
7519893	20130116	17171601	19540813	1	20130116	6
9065285	20130411	17425310	19471228	1	20130411	6
9679112	20131016	18016184	19500116	2	20131016	4
12413444	20120715	16606638	19311115	2	20120715	4
14296356	20120607	16493651	19610127	2	20120607	4
14923343	20130102	17119181	19420223	2	20130102	5
16644205	20120629	16554885	19661018	2	20120629	4
17244143	20121209	17053152	19400101	2	20121209	5
17990782	20131102	18066180	19321125	2	20131102	5
18936373	20111112	15882917	19550520	2	20111112	4
21017312	20121123	17004351	19410420	2	20121123	4
21111193	20131128	18151234	19481226	1	20131128	5
22609489	20120915	16793967	19540315	2	20120915	6
22611150	20130825	17847319	19440822	1	20130825	6
22648891	20121003	16847381	19470428	1	20121003	4
22792730	20120514	16421622	19270310	2	20120514	4
22936318	20120821	16719268	19580827	1	20120821	6
26013229	20120907	16770568	19550512	2	20120907	5
26834617	20130410	17421195	19350517	2	20130410	5
28386983	20121111	16963321	19470105	2	20121111	6
28458653	20120528	16458152	19420609	1	20120528	6
29080817	20130901	17865238	19621112	2	20130901	4
29625670	20130827	17854697	19600220	1	20130827	6
30313292	20120630	16556288	19620108	2	20120630	5
30619751	20120507	16398299	19500920	2	20120507	6
30631040	20120325	16268914	19990915	1	20120325	6
30759263	20130707	17693957	19770826	1	20130707	4
31430418	20120816	16706642	19461125	1	20120816	4
31894970	20120401	16285105	19641124	2	20120401	5
32939836	20120517	16432066	19470905	1	20120517	4
33490389	20121001	16837433	19590320	2	20121001	5
34444694	20120712	16599441	19611019	1	20120712	5
34611126	20121215	17077007	19600730	1	20121215	4
35465964	20131021	18027138	19441130	1	20131021	4
35722953	20131030	18054836	19400227	1	20131030	6
35797967	20120910	16777460	19540208	2	20120910	4
35963521	20121227	17109034	19450518	2	20121227	5
36236836	20111104	15860289	19630922	2	20111104	5
36622103	20130724	17749329	19540910	2	20130724	5
36931994	20130216	17250098	19680302	1	20130216	5
37033399	20120820	16714325	19551001	2	20120820	4
37076703	20130714	17716136	19520515	2	20130714	6
37175749	20120603	16474584	19870731	2	20120603	5

37271979	20111213	15974380	19581110	1	20111213	4
37328942	20130123	17191674	19800807	2	20130123	6
37535287	20120422	16352073	19591026	2	20120422	4
37575829	20130308	17320991	19541028	2	20130308	5
37839626	20120905	16762316	19340623	2	20120905	6
37922853	20120821	16718748	19570722	1	20120821	6
37982573	20121014	16878190	19810902	2	20121014	6
38034478	20120114	16069255	19540530	1	20120114	5
38078783	20131117	18118279	19750411	1	20131117	5
38195663	20120508	16402606	19780315	2	20120508	6
38207260	20131016	18015347	20010525	2	20131016	4
38211506	20120508	16404078	19610220	2	20120508	4
38211631	20121001	16836093	19401123	2	20121001	4
38260536	20130119	17180134	19561118	1	20130119	4
38298781	20120222	16174910	19460106	2	20120222	6
38401762	20121211	17062204	19371129	1	20121211	6
38473722	20130515	17531833	19380306	1	20130515	4
38493719	20120204	16119250	19410609	1	20120204	5
38497733	20120828	16736216	19530410	2	20120828	6
38567054	20130118	17177484	20090613	1	20130118	5
38656552	20120726	16640789	19640101	1	20120726	5
38823608	20121126	17009550	19550211	2	20121126	4
38954517	20121210	17058106	19540303	2	20121210	6
39034736	20121121	16996460	19500118	1	20121121	4
39098378	20130619	17637141	20070101	2	20130619	5
39178693	20130502	17488977	19550415	1	20130502	4
39539532	20131208	18185115	19620610	2	20131208	5
39891264	20130930	17957385	19550921	2	20130930	6
40015541	20131115	18116123	19660820	2	20131115	5
2203987	20131101	18061593	19700730	1	20131101	5
2251947	20131004	17978919	19491107	2	20131004	4
6308958	20121116	16983522	19380925	1	20121116	4
6427641	20131011	18000779	19610110	1	20131011	4
8215052	20130917	17924831	19570205	2	20130917	4
8348821	20120701	16557055	19351211	1	20120701	4
8572552	20131016	18015901	19481209	1	20131016	4
10291539	20130723	17745774	19581230	1	20130723	5
11032545	20130501	17483266	19351207	2	20130501	5
12876947	20131222	18228419	19530628	2	20131222	5
13019506	20121111	16963623	19580324	1	20121111	4
14823155	20130508	17509523	19630729	2	20130508	4
17244143	20130114	17162575	19400101	2	20130114	5
20043021	20130422	17456618	19541212	2	20130422	4
20368074	20121212	17065698	19590322	2	20121212	5
21951688	20121203	17031360	19620126	1	20121203	5
22611150	20130910	17903004	19440822	1	20130910	6
22792730	20120526	16454367	19270310	2	20120526	4
27286857	20130526	17560488	19590820	2	20130526	4
27709893	20131224	18234428	19380608	1	20131224	6
28349862	20120317	16250238	19320823	2	20120317	4
28763362	20120807	16674220	19690601	2	20120807	6
31261571	20130718	17732682	19620805	1	20130718	6
31319058	20131113	18108052	19790916	1	20131113	6
31363152	20121008	16862873	19751022	1	20121008	6
32546295	20121112	16967488	19520916	2	20121112	5

32916419	20120920	16809097	19540607	2	20120920	5
32921623	20121217	17081478	19730729	2	20121217	5
3310458420131028		18049241	19420316	2	20131028	6
3353846420131014		18007217	19690425	2	20131014	5
34199649	20120326	16271524	20010528	2	20120326	6
34488396	20121031	16925355	19640224	1	20121031	5
35940251	20111207	15957108	19640711	2	20111207	4
3596352120130111		17154203	19450518	2	20130111	5
3606800520130301		17293601	20030123	2	20130301	5
36236836	20111112	15882936	19630922	2	20111112	4
36276570	20111231	16021872	19400831	2	20111231	4
3670395420130411		17425672	19520701	1	20130411	4
36762900	20120731	16651572	19560915	2	20120731	5
3684654520130101		17117690	20000922	1	20130101	5
36874176	20121204	17036751	19431006	1	20121204	5
36920760	20120630	16556455	19480305	2	20120630	5
36941647	20121209	17053318	19560201	1	20121209	4
36973587	20120715	16607041	19570221	1	20120715	4
37274718	20120531	16466399	20091219	1	20120531	6
37309549	20121111	16963609	19471205	1	20121111	4
3734334320130323		17365271	19570714	2	20130323	4
3746669620130718		17731937	19591031	2	20130718	5
37583689	20120127	16092414	19370901	2	20120127	6
37769190	20110528	15391722	19611023	1	20110528	4
3790181820131023		18037177	19521026	2	20131023	4
37922853	20120908	16772627	19570722	1	20120908	6
3797058420130104		17132671	19610115	2	20130104	4
37982573	20121122	17001240	19810902	2	20121122	6
3813025520130328		17379433	19390508	1	20130328	4
38238072	20111214	15974540	19820408	1	20111214	6
38252403	20120325	16268817	20090901	1	20120325	6
38401693	20121030	16921732	19680606	2	20121030	6
38486907	20121002	16839880	19950929	2	20121002	4
38656552	20120821	16719297	19640101	1	20120821	5
3866495820131106		18083769	19421218	1	20131106	4
38714180	20121227	17110748	19780402	2	20121227	5
3904565320131201		18156771	19721005	1	20131201	4
3917719020130330		17383349	19580420	1	20130330	5
3923292320130503		17491645	19590325	1	20130503	6
3923789320130722		17741778	19450613	2	20130722	4
3926195520130131		17211313	19710614	1	20130131	5
3926548020131123		18137629	19500502	1	20131123	6
3938026220130803		17780551	19510712	2	20130803	6
3941537720131021		18029720	19691122	2	20131021	6
3950059920131201		18156813	19390312	2	20131201	5
3962916820130923		17936774	19610331	2	20130923	5
3976986320130903		17877363	19781117	2	20130903	5
225194720131204		18172791	19491107	2	20131204	4
3696911	20121204	17036613	19370220	1	20121204	4
506544120130601		17578136	19550103	2	20130601	5
552946420130402		17395497	19330214	2	20130402	4
6308958	20121214	17075035	19380925	1	20121214	6
1492334320130122		17185665	19420223	2	20130122	4
1690242420121212		17067254	19540915	2	20121212	6
2146643320130201		17217319	19330608	1	20130201	4

21951688	20121217	17081614	19620126	1	20121217	4
22468208	20121120	16992347	19440920	1	20121120	6
23101635	20121207	17050409	19510825	2	20121207	6
23231596	20120908	16772724	19570206	1	20120908	5
2962567020130925		17945717	19600220	1	20130925	5
3250122720130416		17438084	19391212	2	20130416	5
32558068	20120107	16046899	19680302	1	20120107	4
3310458420131115		18115980	19420316	2	20131115	6
3353846420131111		18099256	19690425	2	20131111	5
33981554	20121014	16878239	19650923	2	20121014	4
3496483520130128		17203030	19480520	2	20130128	6
35940251	20111212	15970400	19640711	2	20111212	4
3596352120130129		17206609	19450518	2	20130129	6
36236836	20111119	15903121	19630922	2	20111119	5
3625661820130214		17246152	19410628	2	20130214	4
36276570	20120128	16093946	19400831	2	20120128	6
3669392220130516		17535624	19580111	2	20130516	5
3670395420130502		17489285	19520701	1	20130502	6
36762900	20120824	16727844	19560915	2	20120824	5
36825475	20120904	16757552	19561031	1	20120904	4
3707670320130923		17938240	19520515	2	20130923	5
37458609	20121119	16987694	19581108	1	20121119	5
37643019	20121203	17029874	19290720	1	20121203	6
3767869620130115		17167678	20030313	2	20130115	6
3781055220130411		17424489	19520226	1	20130411	4
3790181820131113		18108835	19521026	2	20131113	5
3798257320121221		17095497	19810902	2	20121221	4
3818931020130501		17484255	19480410	1	20130501	5
38575278	20120626	16545331	19570805	2	20120626	6
3868337320130621		17643619	19580619	1	20130621	5
38686065	20120823	16724548	19930604	1	20120823	6
3881578020130316		17345109	19460302	1	20130316	6
38823551	20120810	16688185	19570710	2	20120810	4
3888074920130528		17567330	19251112	2	20130528	4
3904565320131206		18180215	19721005	1	20131206	5
3914932120130822		17840823	19601230	1	20130822	6
3917546720130117		17173713	19471110	2	20130117	5
3923292320130517		17537598	19590325	1	20130517	4
3926195520130220		17264046	19710614	1	20130220	5
3929223420131024		18039200	19570101	1	20131024	5
3953207520130923		17938192	19550715	1	20130923	5
3980727720130906		17891436	19620320	1	20130906	5
124	20110719	15546134	19310208	1	20110719	7
6848	20110422	15289801	19240907	1	20110422	8
16886	20110321	15195206	19281111	1	20110321	8
21294	20121112	16967420	19270908	1	20121112	8
36420	20110510	15341958	19240219	1	20110510	10
5015720130820		17835494	19590911	2	20130820	7
50259	20110208	15068488	19280125	1	20110208	10
53543	20110103	14974603	19271112	1	20110103	9
5704520130615		17624222	19260314	1	20130615	8
89009	20120103	16032728	19310616	1	20120103	10
89656	20111130	15931137	19260426	1	20111130	7
10440520130901		17865071	19340402	1	20130901	8
106683	20110828	15659005	19290418	1	20110828	7

108703	20120722	16626788	19291003	1	20120722	7
110258	20131215	18208715	19470924	2	20131215	7
122634	20110831	15664898	19280506	1	20110831	7
126465	20120419	16346741	19260316	1	20120419	10
133915	20110311	15170193	19230601	1	20110311	10
146452	20101219	14936144	19230905	1	20101219	10
167317	20120117	16073815	19290105	1	20120117	10
184383	20120625	16541402	19250512	1	20120625	8
219136	20131005	17981651	19240129	1	20131005	8
230013	20110908	15695619	19251116	1	20110908	7
231492	20110406	15237748	19270116	1	20110406	7
232860	20110918	15719162	19281204	1	20110918	10
286282	20110417	15272493	19311204	1	20110417	9
364692	20131107	18087788	19230709	1	20131107	8
394241	20111010	15782701	19260604	1	20111010	7
395926	20120110	16054361	19250720	1	20120110	8
413898	20111217	15986579	19240713	1	20111217	8
428284	20120623	16537385	19241025	1	20120623	9
443550	20110605	15414541	19201225	1	20110605	8
470019	20110626	15473612	19220103	1	20110626	10
470984	20120307	16218583	19300108	1	20120307	7
498817	20120201	16103762	19420807	2	20120201	8
531964	20131017	18020055	19330605	1	20131017	8
557493	20131118	18121085	19240213	1	20131118	7
567602	20130602	17578531	19320116	1	20130602	8
574185	20111214	15976830	19300316	1	20111214	7
577311	20111220	15993932	19260612	1	20111220	8
577355	20110808	15604158	19270110	1	20110808	8
586867	20120329	16280880	19221027	1	20120329	8
591060	20110513	15354491	19641007	1	20110513	10
591479	20130825	17847204	19471204	1	20130825	8
600055	20110304	15147735	19231012	1	20110304	8
600340	20111031	15839852	19310504	1	20111031	7
610708	20110604	15414037	19591209	1	20110604	9
622957	20110124	15036165	19260608	1	20110124	8
658855	20130124	17195147	19230210	1	20130124	7
676722	20120430	16371778	19281110	1	20120430	7
687343	20110920	15725992	19340915	2	20110920	8
707813	20101227	14957110	19340327	2	20101227	8
738738	20110303	15142744	19310303	1	20110303	8
744730	20110301	15134438	19280511	1	20110301	9
767737	20120224	16180120	19430701	2	20120224	8
784714	20110227	15125973	19280619	1	20110227	8
804411	20130201	17216711	19301227	1	20130201	7
809654	20130127	17199947	19280905	1	20130127	8
822300	20110809	15608900	19491010	2	20110809	10
824113	20120927	16827503	19260513	1	20120927	7
845921	20130811	17806812	19450225	2	20130811	7
858526	20120317	16250028	19271017	1	20120317	8
865350	20120318	16250530	19321212	1	20120318	8
868462	20110528	15391487	19181014	1	20110528	8
884333	20120720	16624386	19251018	1	20120720	7
890539	20130522	17550412	19390804	2	20130522	8
895681	20110316	15183653	19381024	1	20110316	7
951893	20120216	16158407	19251223	1	20120216	7

958963	20110823	15647238	19300401	1	20110823	8
973999	20111018	15807905	19540425	1	20111018	8
978278	20111120	15903400	19211010	1	20111120	7
980234	20120930	16831497	19471216	2	20120930	9
1013769	20110417	15272873	19640326	1	20110417	10
1094377	20130908	17893804	19311107	1	20130908	8
1119637	20130507	17504279	19330430	1	20130507	8
1139077	20120412	16327170	19231010	2	20120412	8
1175139	20121112	16967486	19300814	1	20121112	8
1177873	20130104	17131775	19290718	2	20130104	8
1182747	20120503	16385634	19370707	2	20120503	8
1199640	20110608	15423891	19330618	1	20110608	7
1209569	20131104	18071892	19290923	1	20131104	7
1219803	20111124	15915441	19370617	2	20111124	7
1242882	20121115	16979896	19421015	2	20121115	7
1257223	20110227	15125995	19261201	1	20110227	8
1271596	20110818	15635349	19200826	1	20110818	7
1274415	20110505	15327346	19210302	1	20110505	8
1278462	20120717	16614633	19270612	1	20120717	8
1283187	20110503	15318213	19230319	1	20110503	7
1346107	20111115	15890429	19391120	1	20111115	7
1376825	20110906	15685595	19240611	1	20110906	8
1383922	20110619	15455660	19291102	2	20110619	9
1415298	20110315	15179114	19170408	1	20110315	7
1416713	20121009	16866901	19220116	1	20121009	7
1425769	20111020	15814348	19200206	1	20111020	8
1434624	20120814	16698287	19540712	1	20120814	8
1438842	20121215	17076897	19570115	2	20121215	9
1457063	20120410	16317759	19500122	2	20120410	7
1471836	20121214	17072303	19271106	1	20121214	10
1478155	20121202	17024971	19350307	1	20121202	8
1482775	20120320	16256872	19331030	1	20120320	7
1492315	20120322	16263662	19360705	1	20120322	8
1496942	20110315	15180210	19320201	1	20110315	7
1513617	20110411	15253081	19450610	2	20110411	8
1529777	20130412	17426802	19211106	1	20130412	8
1544065	20120729	16645321	19430608	2	20120729	8
1563219	20120815	16702959	19681121	2	20120815	7
1575264	20120220	16167966	19381211	1	20120220	7
1579471	20130610	17610106	19270617	1	20130610	8
1580105	20130118	17178080	19280816	2	20130118	7
1612664	20110702	15491151	19281023	1	20110702	7
1639323	20131029	18052559	19270918	1	20131029	7
1657892	20110120	15027878	19320112	1	20110120	7
1669405	20110303	15143960	19210920	1	20110303	7
1723202	20110418	15276224	19300506	1	20110418	8
1737468	20110216	15097318	19210729	2	20110216	8
1785100	20110427	15302162	19501113	2	20110427	10
1798921	20120223	16176504	19240715	1	20120223	10
1804995	20120430	16372375	19290617	1	20120430	10
1814853	20110417	15272922	19550713	1	20110417	7
1840795	20130321	17360790	19430225	1	20130321	8
1852706	20110121	15031648	19320728	1	20110121	10
1861774	20110415	15270739	19250406	1	20110415	8
1862288	20120210	16137558	19530812	2	20120210	8

1900476	20130404	17402404	19311207	1	20130404	8
1904649	20110310	15166614	19290826	1	20110310	7
1919988	20110924	15737686	19200606	1	20110924	10
1929733	20110309	15162676	19420902	2	20110309	9
1949195	20130804	17781066	19300815	1	20130804	8
1977362	20121109	16959318	19280203	1	20121109	7
1984301	20110530	15393525	19280711	1	20110530	7
2035872	20111222	16000378	19281223	1	20111222	8
2052495	20110210	15077950	19330107	1	20110210	9
2053341	20110323	15201334	19300413	1	20110323	7
2072459	20110506	15330195	19330425	1	20110506	7
2078935	20130927	17952007	19491130	2	20130927	8
2081698	20121213	17070501	19621105	2	20121213	9
2106936	20110919	15723170	19400816	1	20110919	10
2111902	20120105	16038858	19220112	1	20120105	7
2136427	20120727	16641641	19451001	2	20120727	7
2140398	20110812	15619073	19311020	1	20110812	10
2161719	20110304	15146304	19210321	1	20110304	10
2165119	20130623	17647156	19290524	1	20130623	7
2178225	20110330	15218829	19331201	1	20110330	9
2181171	20111023	15820679	19450509	2	20111023	8
2203987	20120430	16371317	19700730	1	20120430	7
2206986	20110515	15356422	19460215	2	20110515	7
2207467	20110217	15099328	19190906	1	20110217	7
2211714	20130404	17402219	19240424	1	20130404	9
2241158	20110315	15180073	19500827	1	20110315	10
2241998	20111227	16012512	19411028	2	20111227	7
2242526	20110409	15249319	19281029	1	20110409	8
2262364	20121012	16875024	19230610	2	20121012	7
2265227	20130222	17272010	19310803	1	20130222	7
2276553	20130404	17403261	19371105	2	20130404	8
2363395	20130806	17790183	19290104	1	20130806	8
2475458	20110215	15091977	19300810	1	20110215	9
2476542	20110823	15646155	19331116	1	20110823	8
2486977	20120701	16556793	19281111	2	20120701	10
2488699	20130831	17864309	19260208	1	20130831	7
2546330	20121024	16906358	19400325	1	20121024	8
2547139	20110105	14983830	19360127	1	20110105	8
2566714	20111013	15794079	19341220	2	20111013	7
2571359	20110220	15106201	19391120	2	20110220	7
2585128	20130828	17856711	19451022	1	20130828	8
2591904	20111227	16012566	19280720	1	20111227	9
2601798	20111009	15782285	19710914	2	20111009	7
2605121	20120310	16228997	19540314	1	20120310	10
2605472	20130524	17558178	19390818	1	20130524	7
2629041	20110626	15473905	19710928	2	20110626	8
2636933	20110317	15187270	19300212	1	20110317	7
2691830	20110704	15498470	19390118	2	20110704	10
2694737	20130501	17484126	19290912	1	20130501	9
2694964	20130911	17906763	19361228	1	20130911	8
2704098	20120313	16237694	19260403	1	20120313	10
2828888	20110207	15062449	19301227	1	20110207	8
2829449	20110625	15473439	19270118	1	20110625	8
2832759	20120721	16626339	19250310	1	20120721	8
2855972	20101226	14954908	19260529	1	20101226	8

2865556	20110212	15084172	19360220	2	20110212	8
2896471	20111207	15955339	19250706	1	20111207	8
2904827	20110519	15370699	19250620	1	20110519	8
2916021	20110103	14973873	19530920	2	20110103	8
2932107	20110216	15096984	19260720	2	20110216	9
2937077	20130415	17433263	19390926	1	20130415	9
2944845	20111110	15877772	19390805	2	20111110	8
2953653	20130504	17495234	19210818	1	20130504	7
2959775	20120901	16746372	19281013	1	20120901	7
2968583	20121025	16911881	19351022	2	20121025	7
2991380	20120329	16280429	19210706	1	20120329	10
3035663	20120718	16617712	19291103	1	20120718	7
3055365	20120801	16656263	19421104	2	20120801	7
3065063	20111108	15866885	19250215	1	20111108	8
3085287	20110214	15088789	19270624	2	20110214	7
3093978	20120621	16534081	19430517	1	20120621	7
3094379	20110705	15499898	19260814	1	20110705	8
3117860	20110317	15186727	19180715	1	20110317	7
3142209	20130923	17936003	19521109	2	20130923	8
3213885	20110410	15250008	19240123	1	20110410	10
3232006	20120411	16322773	19381228	2	20120411	7
3242588	20120420	16349922	19320508	1	20120420	7
3261094	20110629	15482267	19280728	2	20110629	8
3268686	20120530	16463891	19450102	2	20120530	8
3268733	20111002	15756335	19560225	2	20111002	7
3275556	20120603	16474447	19201128	1	20120603	7
3283258	20120829	16738982	19420619	2	20120829	10
3316856	20110315	15179543	19460717	2	20110315	7
3331111	20131210	18193245	19271020	1	20131210	7
3343100	20120426	16364477	19250709	2	20120426	7
3367508	20130211	17242854	19220908	1	20130211	8
3376156	20111125	15919014	19321005	1	20111125	8
3381053	20110301	15134378	19471208	2	20110301	7
3413181	20110304	15146300	19350827	1	20110304	10
3522789	20130529	17570259	19400806	2	20130529	8
3546869	20110828	15659134	19251202	1	20110828	7
3562230	20110311	15170321	19300108	1	20110311	10
3589648	20120415	16333664	19461010	2	20120415	8
3670448	20110815	15625418	19121026	1	20110815	8
3670937	20110427	15301698	19480212	2	20110427	7
3683747	20120315	16245004	19590923	2	20120315	7
3696911	20111127	15921767	19370220	1	20111127	8
3765013	20110301	15132537	19241002	1	20110301	7
3776167	20110227	15126086	19290118	1	20110227	7
3864208	20131120	18127678	19330708	1	20131120	8
3910938	20110213	15084665	19310223	1	20110213	8
3934449	20130220	17264414	19270327	1	20130220	7
3995264	20120819	16711723	19280706	1	20120819	9
4007505	20130626	17656081	19191120	1	20130626	9
4022597	20130825	17847573	19481002	1	20130825	7
4022699	20110215	15093207	19370304	2	20110215	7
4065876	20130502	17485871	19270315	1	20130502	8
4074402	20130927	17950061	19620730	1	20130927	8
4077092	20120914	16791913	19330301	1	20120914	8
4078380	20110828	15659011	19361101	1	20110828	10

4114130	20110520	15373737	19300510	1	20110520	10
4137897	20110502	15314967	19630511	2	20110502	7
4149273	20120502	16382554	19440517	2	20120502	7
4153860	20111217	15985535	19280712	1	20111217	7
4159391	20110315	15180225	19440525	2	20110315	8
4161222	20130824	17846796	19470103	1	20130824	8
4167684	20111115	15890891	19310315	1	20111115	7
4233425	20110222	15113991	19460508	2	20110222	9
4234791	20110725	15560842	19361112	2	20110725	10
4253865	20110226	15125516	19300720	1	20110226	10
4258439	20130729	17760551	19320101	1	20130729	7
4265729	20110111	15001340	19210714	1	20110111	10
4278153	20130115	17167072	19310510	1	20130115	7
4309904	20110214	15087116	19470108	1	20110214	7
4311799	20111128	15923283	19490315	1	20111128	9
4344950	20110123	15033770	19520415	1	20110123	8
4345135	20111022	15820097	19191213	1	20111022	8
4432455	20131104	18068260	19420922	1	20131104	9
4477478	20110928	15747033	19170907	1	20110928	8
4546025	20121202	17025191	19320724	2	20121202	7
4596707	20110622	15466376	19310608	1	20110622	8
4614975	20130113	17159725	19511226	2	20130113	9
4640782	20110927	15743810	19490201	2	20110927	8
4644273	20110525	15383790	19210218	1	20110525	10
4668273	20120423	16354420	19270825	1	20120423	7
4702027	20111004	15764988	19350710	1	20111004	10
4781093	20110501	15309267	19540724	2	20110501	7
4822575	20110312	15172162	19540801	1	20110312	8
4828039	20120127	16092689	19310705	1	20120127	7
4879623	20111110	15874868	19380429	1	20111110	10
4881189	20120414	16332938	19241027	1	20120414	7
4897034	20110204	15058462	19300906	1	20110204	8
4919113	20121123	17003734	19510926	2	20121123	10
4949159	20110801	15577230	19210506	1	20110801	8
4966192	20110331	15220436	19250605	1	20110331	8
4997960	20131026	18045144	19311124	2	20131026	7
5000942	20120512	16416942	19251226	2	20120512	8
5008402	20130721	17738135	19310829	1	20130721	7
5031845	20120917	16796049	19321130	2	20120917	7
5043561	20130820	17836289	19470409	2	20130820	7
5069841	20130328	17379253	19690608	2	20130328	7
5110690	20110120	15027436	19491101	1	20110120	10
5130063	20110601	15401611	19290409	2	20110601	10
5132230	20120914	16790956	19401008	1	20120914	7
5137440	20110222	15113313	19571214	2	20110222	10
5174572	20110909	15699760	19291001	1	20110909	10
5211223	20130901	17864865	19430420	1	20130901	7
5212373	20110503	15319971	19321110	2	20110503	8
5224760	20110516	15360090	19420701	2	20110516	10
5230115	20121230	17115042	19410303	2	20121230	8
5234866	20111224	16005190	19530212	1	20111224	9
5255867	20121026	16914907	19390526	2	20121026	7
5324356	20130421	17453179	19120723	1	20130421	7
5337859	20120320	16256564	19270211	1	20120320	8
5398436	20131002	17969489	19430508	2	20131002	8

5419396	20110816	15629315	19430405	2	20110816	7
5431754	20110601	15403527	19281113	2	20110601	10
549607120130922		17933982	19250714	1	20130922	8
549613920130130		17208723	19760909	2	20130130	7
5503242	20120504	16392755	19370707	2	20120504	7
5522872	20110513	15354143	19470301	1	20110513	8
5525531	20110713	15527177	19291111	1	20110713	8
5529464	20120427	16367649	19330214	2	20120427	7
5531077	20120915	16793972	19540116	2	20120915	7
5559795	20110326	15209793	19310724	1	20110326	8
5565264	20110518	15366552	19341029	1	20110518	10
5606428	20120611	16503342	19310104	1	20120611	8
5707415	20110503	15320118	19421218	1	20110503	8
5724470	20111026	15829991	19270720	1	20111026	10
5738669	20110324	15203175	19250505	2	20110324	10
5748005	20120126	16090169	19321022	1	20120126	8
5753195	20110624	15472133	19291028	1	20110624	10
5781599	20110208	15066340	19220115	1	20110208	8
5796021	20101219	14936432	19260105	1	20101219	7
582062220130706		17692980	19630823	1	20130706	8
5891918	20110320	15192424	19201114	1	20110320	7
5898124	20120320	16256403	19221101	1	20120320	7
5935068	20120809	16684150	19240909	1	20120809	10
5979806	20110315	15180233	19251110	1	20110315	8
6013907	20120430	16372788	19510828	1	20120430	7
6016575	20111025	15824302	19250123	1	20111025	8
601734120130215		17248762	19510115	2	20130215	9
601760320131008		17991175	19430322	2	20131008	7
603427120130625		17654486	19590610	2	20130625	8
6040751	20120218	16164053	19250107	1	20120218	7
6140041	20120719	16619995	19680724	2	20120719	8
6174783	20111226	16008481	19540113	2	20111226	10
6189464	20110428	15305214	19210316	1	20110428	7
626127420131122		18134540	19540420	1	20131122	7
6261445	20120822	16722339	19521009	1	20120822	10
626277720130430		17477636	19190115	1	20130430	7
626331620130617		17628083	19311011	1	20130617	7
627760720130424		17463930	19641116	2	20130424	7
629414020130223		17273739	19471009	2	20130223	8
6315453	20120828	16736113	19531211	2	20120828	8
6343437	20110720	15549569	19670304	2	20110720	10
635592620130711		17711074	19240405	2	20130711	7
6363877	20120808	16679067	19380516	1	20120808	7
6409887	20110410	15249726	19490415	1	20110410	10
6416519	20110823	15647994	19300313	1	20110823	7
6427641	20111220	15992261	19610110	1	20111220	7
6430495	20110324	15205431	19310506	1	20110324	8
6437349	20110508	15335016	19590609	1	20110508	8
6447809	20120328	16278466	19490910	1	20120328	8
6448562	20120428	16368966	19790812	2	20120428	10
6456855	20110116	15014639	19251215	1	20110116	8
6461810	20110607	15418703	19290508	1	20110607	9
6471563	20110207	15063355	19460801	1	20110207	7
6488455	20120301	16193830	19500321	1	20120301	8
6535291	20111109	15873756	19330128	1	20111109	8

6546765	20120130	16097020	19340404	1	20120130	10
6574523	20121003	16846059	19221127	1	20121003	7
6574954	20111031	15840949	19391214	2	20111031	8
6585144	20110107	14990057	19410830	1	20110107	8
6588018	20110208	15067212	19320513	2	20110208	10
6607814	20110326	15209571	19540221	1	20110326	8
6632800	20110512	15350833	19370128	1	20110512	8
6653947	20110902	15675474	19310726	2	20110902	8
667667120130722		17740751	19320505	1	20130722	7
667868820130615		17624986	19630810	1	20130615	7
6695961	20110814	15622059	19290308	2	20110814	7
6703748	20121020	16897987	19300501	1	20121020	8
6776212	20120104	16035181	19400922	1	20120104	8
681061320130621		17642179	19280813	1	20130621	7
6812017	20111218	15987025	19320924	2	20111218	8
6816611	20111010	15783007	19300629	2	20111010	8
6829818	20111027	15833270	19210214	1	20111027	8
6830804	20110503	15320103	19270512	2	20110503	10
6832004	20120405	16304205	19351029	1	20120405	7
6886733	20110124	15036092	19260815	1	20110124	10
689434420130403		17396094	19520419	1	20130403	8
6920065	20110721	15552674	19261011	1	20110721	7
6926029	20111227	16011324	19251024	1	20111227	7
6937402	20120115	16069330	19301128	1	20120115	8
6947848	20121014	16877923	19310811	1	20121014	8
6948841	20110108	14993778	19760917	2	20110108	7
695395320130305		17306304	19440304	1	20130305	7
6961440	20120622	16535808	19330913	1	20120622	7
697109120130115		17166739	19190915	1	20130115	7
698703720131208		18185148	19201110	1	20131208	8
7001936	20120619	16526361	19270610	1	20120619	8
701965020131122		18134564	19290103	2	20131122	7
7020646	20120221	16171587	19581021	2	20120221	7
7044500	20121123	17003224	19560905	2	20121123	9
704452220130121		17182973	19290510	1	20130121	7
7060846	20120310	16229205	19541215	2	20120310	8
706409720130802		17778194	19520722	1	20130802	7
7124427	20101213	14920931	19430309	2	20101213	10
7153222	20110730	15574516	19340815	1	20110730	7
7163635	20120208	16133159	19590305	2	20120208	10
7183724	20110726	15563658	19540110	1	20110726	8
7211521	20110216	15097129	19280917	1	20110216	8
7242639	20120504	16392537	19511117	1	20120504	8
7255154	20120922	16814068	19170824	1	20120922	9
726617320130416		17439278	19420101	2	20130416	7
726736920131004		17979718	19580213	1	20131004	8
7331455	20110509	15339335	19290218	1	20110509	8
7422642	20121118	16985829	19420325	1	20121118	8
7453396	20110809	15608602	19410719	2	20110809	7
7469865	20111111	15880590	19300911	1	20111111	10
752536220130524		17558031	19780906	1	20130524	8
752939720130228		17288295	19300530	1	20130228	7
7543751	20121012	16875789	19530722	2	20121012	8
7545177	20120821	16716670	19261004	1	20120821	7
7559822	20110404	15231605	19320904	2	20110404	8

7569859	20121231	17115771	19260625	1	20121231	7
7581853	20110220	15106190	19281112	1	20110220	10
7591313	20110420	15283421	19311221	2	20110420	10
7596465	20121016	16884774	19540216	2	20121016	7
7609350	20110928	15746750	19250716	1	20110928	7
7682806	20110629	15483047	19560318	2	20110629	10
7703277	20110619	15455659	19470411	2	20110619	7
7703904	20110519	15369662	19290607	1	20110519	7
7724427	20110213	15084496	19250621	1	20110213	9
7731217	20130624	17650099	19781116	1	20130624	8
7736416	20111013	15793853	19461228	1	20111013	7
7736858	20101226	14954973	19420116	2	20101226	9
7765164	20110302	15138835	19400714	1	20110302	8
7788854	20101215	14926918	19330315	2	20101215	8
7838291	20110320	15192643	19250201	1	20110320	8
7852713	20110122	15033219	19250510	1	20110122	8
7888484	20120627	16548899	19291018	1	20120627	9
7926901	20110124	15037292	19470120	2	20110124	8
7930418	20110103	14970461	19560709	2	20110103	9
7949997	20110713	15528993	19160703	1	20110713	7
7979966	20130306	17311582	19540301	2	20130306	7
8003063	20110505	15327827	19500401	1	20110505	10
8081649	20120808	16679691	19341229	2	20120808	7
8089165	20110111	15001686	19750613	2	20110111	8
8105199	20110301	15134553	19310622	1	20110301	10
8154356	20110529	15391977	19510327	1	20110529	10
8180232	20120620	16530422	19401203	1	20120620	7
8186467	20131228	18245290	19400730	1	20131228	7
8192798	20120229	16189868	19230714	1	20120229	8
8247878	20110822	15644759	19640116	1	20110822	9
8255887	20110523	15377174	19340821	1	20110523	8
8259834	20121004	16850266	19490323	2	20121004	7
8260364	20101122	14857348	19611202	2	20101122	10
8271247	20130814	17817778	19441117	1	20130814	8
8321071	20110116	15014690	19240326	2	20110116	8
8327502	20111028	15835533	19370821	2	20111028	7
8348821	20110916	15716572	19351211	1	20110916	7
8349517	20130926	17949242	19631102	1	20130926	9
8363802	20130617	17629861	19290304	1	20130617	7
8367213	20120702	16562281	19300902	1	20120702	8
8371208	20130317	17345549	19410701	2	20130317	8
8389013	20120223	16177992	19420619	2	20120223	7
8401421	20110831	15666631	19540302	1	20110831	7
8418799	20110929	15748177	19400601	1	20110929	7
8483996	20111222	16001019	19440902	1	20111222	8
8570158	20110119	15024714	19610514	1	20110119	7
8572552	20110728	15570935	19481209	1	20110728	8
8574332	20110116	15014321	19261017	1	20110116	7
8587880	20111026	15827548	19610208	1	20111026	7
8613896	20110508	15335011	19600123	2	20110508	8
8624042	20130608	17604962	19481229	1	20130608	9
8630215	20120222	16175065	19790415	1	20120222	8
8666497	20110304	15147688	19410116	1	20110304	10
8667650	20111206	15951601	19570420	2	20111206	10
8717746	20130428	17471728	19240414	1	20130428	7

8740316	20120505	16394655	19261128	1	20120505	7
8745902	20110414	15264955	19271220	1	20110414	8
8793235	20101223	14949888	19611108	1	20101223	8
8794954	20110208	15066768	19351204	1	20110208	8
8822831	20130421	17453434	19280906	1	20130421	8
8862633	20111018	15808425	19271005	1	20111018	10
8902689	20111115	15889968	19411015	2	20111115	8
8904516	20130615	17624998	19490904	2	20130615	8
8913653	20130612	17614912	19270128	1	20130612	9
8916323	20120308	16222646	19470715	1	20120308	8
8926714	20110820	15640768	19620102	1	20110820	8
8958938	20110516	15360008	19250820	1	20110516	7
8984438	20111108	15868464	19391116	1	20111108	7
8997293	20130912	17911364	19411117	2	20130912	8
9015274	20130801	17770721	19371016	1	20130801	9
9055134	20111007	15779712	19581019	2	20111007	7
9115484	20120420	16349762	19540430	2	20120420	8
9133179	20130324	17366138	19290216	1	20130324	7
9190765	20111105	15862014	19180914	1	20111105	7
9209670	20111004	15765697	19541020	1	20111004	10
9221232	20120410	16318655	19530701	2	20120410	8
9226419	20120206	16124553	19540514	2	20120206	9
9245243	20120216	16157335	19511106	1	20120216	9
9294444	20130509	17513144	19501007	2	20130509	8
9305957	20131119	18123336	19590903	2	20131119	8
9328103	20110814	15622197	19360926	1	20110814	9
9328987	20110926	15740646	19251223	1	20110926	8
9333500	20131013	18003111	19460601	1	20131013	8
9357679	20120526	16454228	19330610	1	20120526	9
9369497	20111229	16018379	19590626	2	20111229	7
9392169	20120430	16372209	19500601	2	20120430	8
9396343	20110310	15165622	19480804	1	20110310	10
9408097	20110904	15678831	19530602	1	20110904	8
9419732	20120622	16536462	19250301	1	20120622	8
9432239	20130414	17431673	19520311	2	20130414	8
9444579	20120529	16460388	19390213	2	20120529	7
9446166	20110405	15233906	19460417	1	20110405	7
9460542	20101213	14920970	19441020	1	20101213	9
9499609	20110601	15402636	19311015	1	20110601	8
9504341	20110319	15192012	19300505	2	20110319	8
9549160	20110401	15226002	19490503	2	20110401	7
9560694	20130617	17629154	19570920	2	20130617	8
9567902	20110303	15142363	19330401	2	20110303	8
9573813	20110717	15539107	19491020	1	20110717	7
9585960	20111026	15830441	19250606	1	20111026	7
9610177	20120213	16145517	19210218	2	20120213	8
9614135	20110426	15298126	19550901	1	20110426	7
9655872	20120812	16690563	19250103	2	20120812	8
9674446	20120221	16171102	19590608	1	20120221	8
9679112	20120602	16473764	19500116	2	20120602	8
9816619	20111028	15835618	19430625	1	20111028	8
9841478	20110425	15294723	19560612	2	20110425	7
9841796	20130903	17877274	19300615	1	20130903	8
9872564	20130126	17199617	19540724	2	20130126	8
9921624	20110422	15290318	19260330	1	20110422	8

9922252	20110306	15150204	19611027	1	20110306	7
9949397	20130925	17945668	19661018	1	20130925	7
9984092	20111220	15994252	19540926	2	20111220	7
9993764	20110414	15264951	19300630	1	20110414	7
10038116	20110309	15162302	19631004	2	20110309	9
10054714	20120806	16667987	19540201	1	20120806	7
10076638	20131107	18087209	19270417	2	20131107	7
10091197	20110519	15369300	19471011	1	20110519	9
10110222	20120504	16392694	19500614	2	20120504	9
10162535	20110717	15539096	19420302	1	20110717	8
10193507	20120830	16740232	19270606	1	20120830	7
10240854	20130312	17331185	19511113	2	20130312	7
10271495	20120301	16194713	19430820	1	20120301	7
10299146	20110602	15405954	19240510	1	20110602	7
10311587	20130307	17317658	19570127	2	20130307	7
10324842	20130527	17563925	19451001	2	20130527	9
10327238	20130305	17303834	19190501	1	20130305	8
10330786	20110524	15382066	19560512	1	20110524	10
10341001	20130609	17605470	19501118	1	20130609	8
10350477	20130704	17685280	19630410	2	20130704	8
10406458	20111228	16015517	19220511	1	20111228	8
10422965	20130905	17886039	19610811	1	20130905	7
10432049	20120802	16657398	19270714	1	20120802	9
10523305	20110918	15719157	19590920	2	20110918	8
10567938	20120811	16690043	19580321	2	20120811	9
10587356	20110621	15462339	19530826	1	20110621	8
10602263	20110615	15447090	19400608	2	20110615	8
10638832	20120130	16097043	19710726	1	20120130	8
10640718	20120616	16520157	19191024	1	20120616	8
10678850	20110217	15100066	19290202	1	20110217	7
10681819	20130107	17139599	19480118	2	20130107	8
10702586	20120225	16182239	19520125	1	20120225	7
10718293	20110717	15538856	19630925	1	20110717	9
10739749	20130825	17847317	19500110	2	20130825	7
10786853	20120729	16645477	19300802	1	20120729	7
10801259	20120522	16442643	19501112	2	20120522	8
10826945	20120810	16687685	19560907	1	20120810	7
10900080	20131127	18147725	19380324	1	20131127	8
10939818	20120129	16094702	19431225	1	20120129	8
10949685	20110311	15167790	19320606	1	20110311	8
10979314	20120401	16285444	19250608	1	20120401	10
11001700	20120604	16476232	19300909	1	20120604	7
11006272	20130401	17389397	19611121	1	20130401	7
11024047	20120411	16324430	19300405	1	20120411	8
11031348	20120518	16434025	19540520	1	20120518	7
11032545	20120317	16249965	19351207	2	20120317	8
11035179	20130717	17728537	19550424	2	20130717	8
11055848	20121121	16994334	19641202	2	20121121	8
11093224	20120823	16724543	19291214	1	20120823	9
11101465	20111007	15779583	19510916	1	20111007	7
11208030	20110912	15702465	19521130	2	20110912	9
11264349	20120529	16461054	19591106	2	20120529	7
11276441	20120722	16626891	19300225	1	20120722	10
11279202	20111219	15989150	19251115	1	20111219	7
11304919	20130522	17551394	19500104	2	20130522	8

1132150920130802		17777091	19300616	1	20130802	7
11351181	20110919	15723255	19460628	1	20110919	10
11383167	20110103	14974970	19410903	2	20110103	8
11386348	20121201	17024548	19360303	2	20121201	10
11407979	20110409	15249382	19410128	2	20110409	8
11440270	20110108	14993704	19461226	2	20110108	7
1146231020131103		18066585	19300912	1	20131103	8
1147008920130221		17267951	19280220	1	20130221	9
1149753320130522		17551200	19420327	2	20130522	7
11506051	20120507	16399820	19300202	1	20120507	7
11515483	20111011	15787133	19510304	2	20111011	7
11598417	20120205	16119392	19381213	2	20120205	8
1161709720130219		17258336	19501110	1	20130219	8
11618727	20110914	15710567	19260719	1	20110914	9
11653502	20120101	16022239	19301207	1	20120101	9
11689422	20120817	16708692	19290505	1	20120817	7
11714399	20120407	16310399	19500218	2	20120407	8
11722499	20110216	15097224	19370817	2	20110216	7
1177938720130316		17345195	19810226	2	20130316	8
11793263	20120727	16643398	19341227	1	20120727	8
1186392620131123		18137652	19460601	2	20131123	8
11885964	20111124	15915646	19521029	1	20111124	10
11913921	20120214	16151782	19660522	2	20120214	7
11949385	20110313	15172604	19340913	2	20110313	8
11950451	20111124	15916966	19630812	2	20111124	7
11954500	20110608	15422139	19420323	1	20110608	7
1196481120131216		18209120	19480108	1	20131216	8
1197277320130212		17243644	19550815	1	20130212	9
11978180	20110317	15187421	19640912	1	20110317	7
11993945	20120902	16746595	19440524	1	20120902	9
12032905	20110114	15011677	19340804	1	20110114	10
12037911	20120228	16187580	19290604	2	20120228	7
12044643	20111119	15902870	19531121	1	20111119	10
12072672	20110524	15382266	19500726	2	20110524	7
12086452	20110918	15719100	19321018	1	20110918	7
12086690	20121010	16867718	19470225	1	20121010	8
12088732	20120724	16634000	19620723	2	20120724	7
12089382	20120104	16035821	19520503	2	20120104	7
12102737	20120628	16551144	19480107	2	20120628	7
12111396	20111128	15922227	19510305	2	20111128	7
1211518320130610		17610068	19270302	1	20130610	8
12116459	20110817	15631359	19230809	2	20110817	8
12137427	20120430	16371648	19760423	1	20120430	8
12166937	20110310	15166794	19271208	1	20110310	10
12178448	20120525	16450229	19540328	1	20120525	7
12195050	20120904	16756563	19630310	2	20120904	7
12201331	20120214	16151497	19391225	2	20120214	8
12218212	20121021	16899084	19570519	2	20121021	7
1222703120130729		17760392	19350209	2	20130729	7
12244212	20110427	15301633	19620515	1	20110427	10
12252561	20110509	15338240	19281224	1	20110509	7
12257011	20110222	15113374	19411016	1	20110222	8
12263386	20110522	15375486	19540627	2	20110522	8
1234118320131013		18003241	19681220	2	20131013	7
1234844620130804		17781015	19280319	1	20130804	8

1236817120121229		17114523	19530610	2	20121229	7
12370104	20111024	15822863	19590217	2	20111024	10
12378880	20121123	17002190	19460907	2	20121123	10
12441348	20110315	15180493	19680417	1	20110315	8
1246846120130305		17307900	19550918	2	20130305	7
12481468	20120728	16644912	19531020	2	20120728	8
12512708	20110315	15180497	19320621	1	20110315	7
12514099	20110410	15250124	19480507	1	20110410	10
12541092	20101203	14890620	19520106	2	20101203	8
12561136	20111107	15864693	19441019	2	20111107	10
12577309	20120127	16092220	19580101	2	20120127	7
12587381	20120923	16814460	19350315	2	20120923	8
12591423	20110930	15752906	19240601	1	20110930	8
1260736220130926		17948035	19600104	1	20130926	9
12690312	20110905	15683703	19560826	1	20110905	10
12741581	20110221	15109774	19510429	2	20110221	7
12756013	20110105	14983801	19691125	2	20110105	10
12762300	20111005	15771503	19250720	1	20111005	7
12774184	20111223	16002159	19510826	1	20111223	7
1279843520121106		16947500	19510902	1	20121106	7
12832187	20110119	15025520	19350317	2	20110119	7
12836463	20120501	16378328	19340818	2	20120501	9
12844916	20120506	16395001	19550301	2	20120506	7
1286160820130723		17744003	19440216	2	20130723	8
12891382	20110613	15439093	19470620	2	20110613	10
12893833	20110208	15067140	19410625	2	20110208	9
1290787820131011		17998166	19530312	2	20131011	8
12914726	20110612	15434463	19380620	1	20110612	8
12924913	20111220	15993342	19610308	2	20111220	7
12951870	20110419	15279824	19440328	2	20110419	8
12961329	20110104	14979186	19280201	1	20110104	10
12974866	20110902	15673169	19421021	1	20110902	7
12977661	20110117	15017297	19251215	2	20110117	10
1298918320130716		17723207	19280215	1	20130716	7
13007211	20111117	15897915	19390329	2	20111117	7
13014863	20111122	15909270	19610218	1	20111122	7
13017953	20111213	15973765	19590129	2	20111213	7
1303851220130531		17575608	19391004	1	20130531	8
13048050	20111207	15954873	19450724	1	20111207	9
13048732	20110608	15423164	19411115	1	20110608	8
1305809820130121		17184046	19531209	1	20130121	8
1306052120130224		17273978	19620810	2	20130224	9
13069186	20110213	15084477	19760531	2	20110213	8
1307068320130524		17558051	19560706	2	20130524	8
13085386	20101224	14953255	19430603	1	20101224	10
13098538	20110411	15253883	19401018	1	20110411	7
13112691	20110306	15150152	19320301	1	20110306	8
13122059	20120220	16167657	19290707	1	20120220	7
1312357420130427		17471283	19370911	1	20130427	9
1314034620130110		17153714	19590803	1	20130110	7
13161143	20111023	15820472	19310620	1	20111023	7
13201473	20101222	14945829	19250906	2	20101222	10
13204530	20110119	15025062	19560622	1	20110119	10
13210270	20110501	15309044	19270117	1	20110501	9
13224787	20121104	16938563	19631203	2	20121104	8

13261068	20110318	15190446	19560425	1	20110318	8
1326372420130613		17618434	19331128	1	20130613	9
13285524	20110530	15394101	19291201	1	20110530	10
13303838	20111204	15943422	19430119	1	20111204	7
13324044	20110627	15477127	19311016	2	20110627	8
13342944	20110815	15624756	19330909	2	20110815	8
13345681	20110602	15406365	19690114	1	20110602	8
13345783	20120101	16022586	19401001	2	20120101	7
13372968	20111208	15960715	19540730	2	20111208	8
13381083	20110724	15557907	19560308	2	20110724	7
1338616820130312		17331420	19241203	1	20130312	7
13403480	20121031	16926101	19451225	1	20121031	8
13431726	20110526	15387614	19400425	2	20110526	8
13472565	20120517	16431587	19270915	1	20120517	10
1348251420130104		17131703	19300801	1	20130104	7
13484872	20110112	15005932	19390514	2	20110112	7
13488181	20110513	15353867	19391030	2	20110513	8
1349866320130225		17276844	19530221	2	20130225	7
13519749	20120303	16203500	19821123	1	20120303	10
1352634620131008		17991491	19250820	2	20131008	8
1352863720130625		17653244	19120102	1	20130625	9
1353135620130226		17279959	19270815	1	20130226	8
13549898	20111122	15909739	19401030	1	20111122	10
13561825	20110216	15096465	19280129	2	20110216	7
13568860	20120502	16383164	19430912	2	20120502	10
13574431	20110620	15459712	19550122	1	20110620	10
13640647	20120413	16331401	19550421	2	20120413	7
13642507	20110915	15711500	19430220	2	20110915	7
13667613	20110214	15087579	19400414	2	20110214	7
13670036	20110609	15428300	19530706	2	20110609	10
13703270	20110102	14969781	19510607	1	20110102	8
13707432	20110322	15199225	19370806	2	20110322	8
13723972	20110429	15307620	19350725	2	20110429	8
1372550320131220		18224944	19290620	1	20131220	8
13770815	20111110	15877139	19570825	2	20111110	9
13774282	20110530	15394966	19650726	1	20110530	8
13785858	20110907	15691199	19570111	2	20110907	9
13794699	20111121	15906498	19460713	1	20111121	8
13803297	20111112	15882594	19440528	2	20111112	8
13823659	20110111	15002060	19350505	2	20110111	10
13823944	20111106	15862315	19270411	1	20111106	9
13830110	20110222	15112074	19300414	1	20110222	7
13836083	20110124	15036856	19530515	2	20110124	10
13836823	20121208	17052388	19630329	2	20121208	7
13840374	20110525	15384216	19281229	1	20110525	7
13846714	20110418	15276555	19551210	2	20110418	9
1386444320130408		17411930	19571013	1	20130408	7
13907454	20110322	15198451	19420620	1	20110322	8
13919863	20121216	17077430	19150821	1	20121216	8
13922424	20120819	16711636	19590319	2	20120819	7
13939372	20110315	15179241	19470815	1	20110315	8
13949854	20110209	15070252	19531017	1	20110209	8
13959223	20110113	15008024	19550115	1	20110113	10
1400928020131202		18158576	19350802	2	20131202	8
1401566820130709		17700797	19480903	1	20130709	9

14031539	20110210	15077408	19591024	2	20110210	8
14058583	20120505	16394450	19401110	1	20120505	8
14063742	20130421	17453024	19260118	1	20130421	7
14074772	20130325	17368677	19500822	2	20130325	8
14091486	20120401	16285414	19560819	1	20120401	10
14111138	20110523	15378796	19521112	1	20110523	8
14125156	20120327	16273774	19441117	2	20120327	8
14196828	20130613	17617704	19691129	2	20130613	7
14222141	20131004	17975881	19310101	1	20131004	7
14224090	20130522	17550566	19190213	1	20130522	8
14224352	20111011	15785857	19570317	1	20111011	8
14246710	20131017	18017447	19290308	1	20131017	7
14249811	20110116	15014790	19510523	1	20110116	7
14291168	20110628	15480061	19391123	2	20110628	8
14453248	20110925	15738395	19320202	1	20110925	10
14466832	20121201	17024699	19470605	1	20121201	7
14495911	20110216	15095943	19560912	1	20110216	7
14501269	20131013	18003281	19521009	2	20131013	7
14501827	20120110	16055982	19771230	1	20120110	10
14503061	20131111	18098416	19520605	2	20131111	7
14504553	20111104	15858505	19300415	1	20111104	9
14506117	20110221	15110310	19591020	1	20110221	10
14513054	20121016	16885408	19200723	1	20121016	7
14525281	20111202	15940657	19410320	1	20111202	8
14528939	20130503	17493592	19281203	1	20130503	7
14536539	20110513	15353991	19360613	2	20110513	7
14559718	20120714	16606161	19550620	1	20120714	8
14591572	20110922	15733274	19450318	2	20110922	8
14595176	20130506	17500730	19270418	1	20130506	7
14610743	20120528	16456979	19630802	1	20120528	8
14622050	20131209	18190036	19260102	1	20131209	7
14630321	20120325	16268876	19281107	1	20120325	7
14635984	20120513	16417693	19510108	2	20120513	8
14655017	20111211	15966440	19551104	2	20111211	7
14657057	20130521	17547501	19311012	2	20130521	8
14684243	20110210	15077686	19251006	1	20110210	8
14740999	20130112	17159349	19321118	2	20130112	7
14743590	20110814	15622283	19631201	2	20110814	7
14744457	20120704	16571872	19310906	2	20120704	8
14756479	20110922	15732911	19400101	1	20110922	7
14765663	20110511	15345900	19591023	2	20110511	7
14768822	20110830	15663531	19471028	2	20110830	7
14778326	20101221	14943705	19521129	1	20101221	10
14797092	20110120	15027634	19321115	1	20110120	10
14798642	20120611	16503914	19311109	2	20120611	7
14823155	20110114	15011985	19630729	2	20110114	8
14829108	20120510	16411841	19481014	1	20120510	8
14842581	20110322	15199652	19570627	1	20110322	7
14863219	20110509	15339903	19481214	2	20110509	8
14890507	20120804	16664568	19581023	2	20120804	8
14896823	20130812	17809723	19290320	2	20130812	7
14897837	20120705	16577766	19281020	1	20120705	9
14903005	20110208	15068524	19360703	1	20110208	7
14922839	20130713	17715356	19500302	2	20130713	7
14999510	20130531	17574104	19261228	1	20130531	7

15022089	20131107	18084865	19251126	1	20131107	8
15022501	20131007	17986419	19450105	2	20131007	7
15031900	20110316	15183772	19311103	1	20110316	7
15056334	20120511	16414369	19290218	1	20120511	7
15076014	20110830	15663265	19331228	1	20110830	7
15096476	20131209	18190113	19600123	2	20131209	7
15096932	20110114	15012150	19351011	2	20110114	10
15102973	20130302	17296324	19581116	2	20130302	7
15105256	20131121	18132328	19241217	1	20131121	7
15112944	20120910	16775739	19781223	1	20120910	10
15156986	20110425	15295382	19580910	2	20110425	8
15164768	20111117	15897334	19330412	1	20111117	9
15190597	20121028	16917081	19560607	2	20121028	8
15233086	20120414	16333243	19250424	1	20120414	7
15247548	20101104	14805592	19550330	2	20101104	10
15254214	20111219	15989118	19310404	1	20111219	10
15254430	20130911	17905634	19560213	2	20130911	7
15277368	20120718	16617241	19340307	1	20120718	8
15289619	20120509	16408033	19570202	2	20120509	7
15294561	20121005	16855058	19500329	1	20121005	8
15323214	20110316	15183828	19301010	1	20110316	7
15332328	20111209	15963350	19610509	2	20111209	8
15345229	20131031	18056899	19300126	1	20131031	7
15369536	20110318	15187573	19301030	1	20110318	7
15370884	20120609	16499566	19450607	1	20120609	7
15379494	20111017	15803950	19510822	2	20111017	9
15388928	20120603	16474400	19330910	1	20120603	9
15433315	20130624	17650822	19230501	1	20130624	8
15448825	20120809	16684165	19231127	1	20120809	9
15528151	20121211	17061699	19471031	2	20121211	8
15528639	20110814	15622387	19300429	2	20110814	9
15530231	20110725	15560678	19570813	2	20110725	8
15543449	20130527	17563865	19501116	2	20130527	7
15544384	20110421	15287199	19610811	2	20110421	8
15552688	20110126	15044157	19290909	1	20110126	7
15559667	20111207	15957051	19420826	2	20111207	10
15565421	20120102	16027713	19480901	1	20120102	8
15572164	20110725	15560153	19430510	1	20110725	8
15584937	20130616	17625573	19770407	2	20130616	8
15603608	20130315	17343494	19560309	2	20130315	7
15620061	20111029	15837419	19500525	1	20111029	8
15636085	20111124	15916754	19410725	1	20111124	10
15651328	20111103	15851860	19560109	2	20111103	7
15658125	20110820	15640707	19321109	1	20110820	7
15672330	20110323	15201372	19520115	2	20110323	9
15718862	20110517	15360619	19651205	1	20110517	7
15718964	20120902	16746856	19131005	2	20120902	7
15728968	20121105	16942868	19441201	2	20121105	7
15757854	20121204	17036403	19530814	1	20121204	9
15759383	20111227	16011847	19680929	1	20111227	7
15759598	20111214	15978268	19510517	1	20111214	7
15781290	20130123	17191332	19230604	2	20130123	8
15787287	20131006	17981977	19320320	1	20131006	8
15800405	20111020	15814646	19381210	2	20111020	8
15816850	20110512	15350444	19300301	1	20110512	8

15833815	20120806	16665258	19310204	1	20120806	8
15849486	20110317	15187403	19410225	2	20110317	7
15860818	20110426	15298849	19350504	1	20110426	7
15867364	20120706	16580056	19311223	1	20120706	8
1589793520130123		17189381	19300415	1	20130123	9
1592371420130318		17349402	19570325	2	20130318	8
15948728	20110512	15350753	19631110	1	20110512	8
15975232	20120115	16069778	19550623	1	20120115	8
16000514	20110819	15638939	19600528	1	20110819	7
1603465420130219		17258168	19250226	1	20130219	7
1607428520130614		17623102	19650212	1	20130614	8
16083888	20121216	17077452	19691103	2	20121216	10
16084530	20110902	15675927	19600224	1	20110902	8
16087131	20110218	15104351	19480505	1	20110218	7
16093086	20110925	15738475	19580717	1	20110925	10
16095822	20110817	15633307	19501027	2	20110817	8
16122159	20121102	16932661	19411109	2	20121102	10
16148917	20111108	15868730	19300903	1	20111108	8
16153949	20111020	15814573	19250930	1	20111020	7
16162019	20101126	14869383	19410315	2	20101126	9
16162359	20111121	15906288	19551003	2	20111121	8
16197118	20120915	16793974	19571208	1	20120915	7
1623251220130630		17665836	19330216	2	20130630	8
16233219	20121212	17067099	19500409	1	20121212	10
1626219420130913		17914946	19470531	2	20130913	8
16269435	20101231	14968230	19531115	2	20101231	8
16287448	20120808	16678796	19250622	1	20120808	8
16298207	20120710	16592530	19290626	1	20120710	7
16303290	20111109	15873130	19420624	2	20111109	7
16309049	20121210	17055907	19590730	2	20121210	8
16332517	20110310	15166875	19311028	1	20110310	10
16334499	20111129	15927503	19570322	2	20111129	10
16337147	20110821	15641183	19680807	1	20110821	9
1641418920130304		17300153	19630301	2	20130304	7
16415477	20110926	15741895	19350915	2	20110926	7
16419559	20111225	16005600	19311010	1	20111225	8
16420727	20120301	16196278	19550725	2	20120301	7
16433742	20120206	16121504	19310101	1	20120206	8
16441433	20110811	15616352	19470118	2	20110811	8
16482374	20111210	15966015	19280228	1	20111210	10
16482761	20110228	15126275	19220909	1	20110228	7
1648282920130604		17589275	19280301	1	20130604	7
16490792	20120828	16734577	19410122	2	20120828	8
16491660	20111013	15794832	19320218	1	20111013	7
16526424	20110522	15375362	19480210	1	20110522	8
1653953020131029		18052311	19310419	2	20131029	7
16579434	20110917	15718782	19410907	2	20110917	8
16593990	20120511	16415283	19570310	1	20120511	10
16600105	20110316	15183263	19600226	1	20110316	8
16605020	20110204	15058757	19610330	1	20110204	8
16643586	20110423	15291947	19160219	1	20110423	10
16644261	20111204	15943390	19680216	1	20111204	8
1664823020130522		17549646	19311226	1	20130522	7
16657311	20111025	15826800	19410703	2	20111025	7
16661339	20121202	17025084	19481201	2	20121202	7

16668396	20121024	16908333	19290303	1	20121024	8
16706095	20120805	16665201	19551017	1	20120805	8
16726059	20110313	15172352	19361211	1	20110313	8
16750213	20110307	15154935	19500210	1	20110307	10
16771350	20110615	15446356	19281229	1	20110615	7
16795645	20120622	16536191	19300221	1	20120622	7
16803182	20110905	15682441	19270713	1	20110905	10
16829633	20130516	17534904	19530312	2	20130516	9
16830527	20120712	16601088	19330916	2	20120712	8
16833048	20110207	15063563	19460905	2	20110207	8
16834927	20110610	15432308	19520917	1	20110610	8
16856896	20121202	17024970	19571130	2	20121202	7
16861828	20131024	18039889	19360815	2	20131024	7
16894192	20121004	16851072	19351102	2	20121004	8
16899186	20110831	15666779	19550522	1	20110831	10
16926286	20110717	15539209	19240906	1	20110717	7
16927610	20110113	15006170	19660713	2	20110113	7
16954760	20110321	15193225	19381129	2	20110321	7
16974666	20130314	17339051	19530915	1	20130314	7
16991176	20130420	17452341	19310628	1	20130420	7
17001502	20120130	16097441	19300511	1	20120130	8
17034478	20130331	17383714	19610529	1	20130331	9
17052005	20110521	15374286	19570205	2	20110521	7
17064812	20110413	15263206	19371120	2	20110413	7
17089177	20111107	15865620	19440110	2	20111107	8
17095328	20110316	15184211	19440518	1	20110316	7
17121129	20121015	16882266	19510814	1	20121015	8
17143565	20111208	15959427	19281107	1	20111208	8
17150924	20131002	17968936	19460625	1	20131002	8
17158484	20130222	17270379	19350120	1	20130222	9
17165616	20120422	16351799	19270410	1	20120422	7
17174662	20120709	16586256	19231015	1	20120709	8
17179145	20110117	15018250	19260519	1	20110117	8
17187778	20130531	17575524	19271107	2	20130531	8
17190011	20131126	18143679	19440201	1	20131126	7
17198004	20120617	16520942	19440418	1	20120617	10
17204421	20110317	15185827	19260425	1	20110317	8
17227495	20110217	15099235	19480314	1	20110217	7
17233259	20110211	15080301	19490220	1	20110211	9
17246763	20120229	16188956	19260206	1	20120229	7
17292352	20110809	15608546	19280305	1	20110809	8
17296412	20110808	15603960	19230513	2	20110808	10
17296876	20120315	16244283	19420102	2	20120315	10
17300651	20120621	16531400	19280511	1	20120621	7
17311545	20120729	16645560	19570811	2	20120729	8
17312662	20120810	16688174	19260313	1	20120810	9
17314339	20121115	16979526	19600815	2	20121115	10
17338704	20110413	15259972	19611201	2	20110413	10
17366824	20120328	16277603	19350301	1	20120328	7
17368784	20131010	17997097	19260915	1	20131010	7
17382671	20120416	16336176	19290328	1	20120416	8
17401842	20111011	15786246	19500928	1	20111011	7
17406438	20120716	16609005	19520222	1	20120716	7
17443446	20131019	18025284	19521103	2	20131019	8
17478987	20110223	15117595	19151021	1	20110223	7

17517043	20110127	15045917	19680730	2	20110127	7
17518808	20111221	15996863	19621025	1	20111221	7
17521083	20120528	16457728	19300224	1	20120528	7
17522644	20110908	15696482	19440303	1	20110908	10
17545130	20110613	15434875	19261119	1	20110613	8
1757787620130304		17298229	19540924	1	20130304	8
17586071	20120719	16620215	19410721	2	20120719	7
17612021	20121028	16916917	19530124	2	20121028	8
17612214	20110220	15106348	19450807	2	20110220	9
17676845	20111014	15798263	19401207	1	20111014	10
17697642	20110209	15072710	19450727	2	20110209	7
17699262	20110903	15678112	19260414	1	20110903	9
17699488	20110828	15658914	19480603	1	20110828	10
17703809	20110914	15710349	19241116	1	20110914	7
17706513	20110829	15661712	19300824	1	20110829	9
17717816	20121217	17081347	19350717	1	20121217	8
17721787	20110728	15570604	19221010	2	20110728	10
17723921	20121017	16889564	19571013	2	20121017	7
17725450	20110625	15473480	19350927	1	20110625	8
17742846	20110928	15747406	19251023	1	20110928	7
17745856	20110506	15332771	19300205	1	20110506	8
17750479	20120607	16493225	19270321	1	20120607	8
17767225	20121026	16914040	19591001	1	20121026	8
17791514	20110509	15339665	19610929	1	20110509	8
17857555	20101019	14758975	19630420	1	20101019	8
17884343	20110501	15309167	19340724	1	20110501	8
17902293	20120624	16538016	19510926	1	20120624	8
1791547820130120		17180266	19510915	2	20130120	8
1791780520130402		17394418	19630129	2	20130402	9
17938271	20110209	15073137	19341012	2	20110209	8
17946086	20110301	15133659	19440626	1	20110301	7
1800116620130602		17578543	19580208	1	20130602	8
18039913	20120720	16624923	19211224	1	20120720	7
18051462	20111106	15862287	19360319	2	20111106	7
18068478	20111022	15819962	19211228	1	20111022	8
18089979	20120110	16056147	19660701	2	20120110	8
18091548	20121024	16909587	19340601	2	20121024	10
1810087320130610		17608626	19530615	2	20130610	7
18109574	20120711	16595691	19380410	2	20120711	7
18110628	20110308	15159078	19540826	1	20110308	7
18128251	20110214	15086302	19340813	2	20110214	10
18129572	20111206	15952668	19311218	1	20111206	7
18155970	20110112	15004760	19490620	1	20110112	10
18177850	20120221	16171430	19870119	2	20120221	7
18180604	20120808	16679767	19560108	1	20120808	10
18185712	20120721	16626363	19330705	1	20120721	9
18225837	20110628	15478116	19631201	1	20110628	8
1822737720130410		17420529	19590425	1	20130410	8
18235397	20111120	15903335	19491007	2	20111120	7
1823588620130705		17689907	19610326	2	20130705	9
18255726	20120423	16355018	19330720	1	20120423	10
18263451	20110621	15462746	19331225	1	20110621	7
18293157	20110518	15365951	19350113	1	20110518	7
1829365720130328		17378909	19320112	1	20130328	7
18315838	20110104	14979243	19761105	2	20110104	7

18324555	20110322	15199517	19260815	1	20110322	9
18358297	20110411	15254000	19330104	1	20110411	8
18366488	20110414	15267551	19280810	2	20110414	8
18373461	20120213	16147269	19330706	1	20120213	7
18421813	20110219	15105626	19630319	2	20110219	10
18448436	20110923	15735758	19220112	2	20110923	8
18462867	20110908	15695172	19570907	1	20110908	8
1846413620130401		17388166	19520513	1	20130401	8
18484827	20120116	16073418	19140124	1	20120116	8
18487804	20120222	16172163	19640708	1	20120222	7
18493282	20120713	16601896	19380104	1	20120713	7
18493555	20111114	15885686	19710609	2	20111114	8
18515485	20110822	15642096	19301225	1	20110822	9
18516648	20110904	15678794	19371225	1	20110904	8
18569227	20120618	16524700	19271228	2	20120618	8
1858374920130421		17453112	19541129	2	20130421	8
18590722	20110316	15184065	19410228	2	20110316	8
18598806	20111127	15921757	19490111	1	20111127	8
18601622	20120605	16484123	19390311	1	20120605	10
18611091	20120504	16391957	19250525	1	20120504	8
1861742020131127		18148565	19540729	1	20131127	8
18639844	20110718	15541657	19500520	2	20110718	8
18676987	20121114	16975183	19290618	2	20121114	7
1868479220130529		17569771	19300203	1	20130529	7
1868984420131127		18148225	19261024	2	20131127	7
18699268	20110321	15194613	19400525	2	20110321	8
1869971320130915		17917478	19310915	2	20130915	7
18715338	20120913	16788314	19280606	1	20120913	10
18718962	20110218	15101262	19201030	1	20110218	7
18722571	20110322	15198475	19760323	2	20110322	10
18736077	20110518	15366359	19560711	2	20110518	8
1877321220131117		18118125	19380122	2	20131117	7
18790437	20110302	15137933	19450131	1	20110302	10
18799934	20120918	16802209	19560120	2	20120918	10
18809773	20110409	15249445	19530709	1	20110409	7
18809900	20120217	16161713	19341215	1	20120217	8
18840205	20120211	16142710	19500826	2	20120211	7
18861693	20120814	16698271	19570909	1	20120814	7
18865015	20110501	15309248	19480403	1	20110501	8
1887141520130506		17499539	19260602	2	20130506	9
1888717720130305		17306429	19260507	1	20130305	9
18901412	20120328	16276888	19301230	1	20120328	8
18901796	20120328	16278450	19510227	1	20120328	10
18934253	20110106	14987178	19300319	1	20110106	8
18936373	20110227	15126069	19550520	2	20110227	8
18966502	20110805	15597486	19300625	1	20110805	7
18992853	20110122	15033086	19310330	2	20110122	8
18995614	20110216	15097244	19270101	2	20110216	7
1900577720131106		18081277	19340319	2	20131106	8
19024329	20110614	15440242	19661205	2	20110614	10
19030332	20110728	15570379	19280703	1	20110728	7
19034685	20120713	16604257	19591125	2	20120713	8
19044054	20110312	15172204	19620414	1	20110312	9
1906199520130408		17410806	19550901	1	20130408	8
1911068020130915		17917408	19590919	1	20130915	8

19132128	20110210	15077362	19330917	2	20110210	8
19140853	20111225	16005626	19260506	1	20111225	7
1914216620130129		17205536	19520202	1	20130129	7
19153878	20111206	15952463	19611116	2	20111206	10
19185923	20110215	15092777	19831005	2	20110215	7
19242149	20110225	15124178	19880319	2	20110225	8
19253124	20120514	16419005	19321206	1	20120514	7
19272550	20120921	16811277	19350701	2	20120921	8
19300302	20110302	15135816	19300813	1	20110302	10
19328531	20120302	16200073	19270808	1	20120302	8
19351554	20120403	16297095	19300412	2	20120403	10
19391583	20110914	15710671	19520901	2	20110914	8
19402541	20110223	15117575	19530210	2	20110223	8
19415328	20110102	14969834	19610301	2	20110102	8
1941692320130518		17539045	19290313	1	20130518	8
19429904	20121219	17088650	19360430	1	20121219	8
19430934	20110114	15011730	19681023	2	20110114	7
19432305	20110518	15367214	19660228	2	20110518	8
19439986	20111229	16018327	19501212	1	20111229	8
19440143	20110925	15738414	19310727	2	20110925	7
19455506	20111117	15897843	19620313	2	20111117	8
19542406	20101224	14953173	19400312	2	20101224	10
19580917	20120106	16044794	19721115	2	20120106	9
19602790	20110801	15580788	19880622	1	20110801	9
1960896920130524		17558189	19160608	1	20130524	7
19635473	20120905	16760499	19341223	2	20120905	10
19635893	20110516	15358589	19330113	2	20110516	7
19644189	20111207	15955476	19540313	1	20111207	8
19645080	20120606	16489122	19591012	2	20120606	8
19672458	20120628	16551842	19660413	1	20120628	8
19697962	20120108	16047459	19341010	1	20120108	7
19703652	20111028	15835933	19610730	2	20111028	10
19706720	20121023	16905280	19270524	2	20121023	7
19709172	20110430	15308772	19810809	2	20110430	10
1972234020130125		17196802	19560202	1	20130125	7
19726900	20110308	15158378	19571020	2	20110308	8
19740568	20120711	16596845	19421205	2	20120711	7
1974077320130915		17917336	19490823	1	20130915	7
1976565220131108		18092753	19361001	2	20131108	8
19782128	20111101	15845365	19261024	2	20111101	7
19825220	20120316	16247426	19320327	1	20120316	10
19825526	20110609	15427701	19310602	1	20110609	8
19841419	20101220	14939760	19581105	1	20101220	8
1984844320130417		17443577	19520120	1	20130417	8
19870689	20110510	15343748	19600828	2	20110510	10
19880058	20111021	15816857	19280217	1	20111021	7
1988932820130211		17242928	19490218	1	20130211	7
1989138420131002		17968223	19890830	1	20131002	7
19893744	20120927	16827437	19310920	2	20120927	8
19904337	20121028	16917175	19510210	2	20121028	8
19920764	20110321	15196374	19470201	1	20110321	7
19923592	20111009	15782164	19730309	2	20111009	7
19926262	20120917	16797363	19611229	1	20120917	7
19926740	20101211	14915968	19540618	1	20101211	8
19937963	20120606	16489503	19640315	2	20120606	7

19971405	20131117	18118362	19261213	1	20131117	8
19976739	20120719	16621283	19520102	1	20120719	7
19980882	20110502	15313577	19580202	2	20110502	9
19981330	20110913	15705247	19291204	1	20110913	10
19992995	20110208	15068184	19530125	2	20110208	8
20008440	20120529	16459989	19701013	2	20120529	8
20011750	20131113	18107485	19390928	1	20131113	7
20025574	20121116	16983423	19640312	2	20121116	10
20026179	20101230	14966556	19380303	2	20101230	8
20035523	20120428	16368402	19250424	1	20120428	9
20057049	20110417	15272486	19791102	2	20110417	10
20067930	20111127	15921779	19550428	1	20111127	7
20068740	20120712	16599765	19570910	1	20120712	8
20091081	20120702	16561167	19530704	2	20120702	7
20098877	20110607	15419781	19330810	2	20110607	9
20126878	20110321	15195702	19621227	1	20110321	7
20137079	20110302	15139456	19340117	2	20110302	7
20209169	20120208	16132179	19520802	2	20120208	7
20236877	20110330	15218236	19510801	2	20110330	9
20242357	20131006	17982032	19370126	1	20131006	8
20242415	20121108	16956045	19340122	2	20121108	8
20263041	20130102	17121114	19550811	2	20130102	7
20267781	20110810	15612461	19291202	1	20110810	10
20276646	20130501	17483371	19300512	2	20130501	7
20319077	20120609	16499609	19240512	1	20120609	7
20341860	20120416	16335481	19320127	1	20120416	7
20383884	20111205	15946556	19780914	2	20111205	7
20387546	20130324	17365911	19631012	1	20130324	7
20428528	20120323	16265959	19650227	1	20120323	10
20433312	20120423	16355446	19260909	1	20120423	8
20442346	20130524	17557961	19560521	1	20130524	8
20449290	20110207	15063613	19650802	2	20110207	8
20452726	20130903	17875480	19431213	2	20130903	9
20462468	20120519	16436688	19620510	1	20120519	8
20496940	20130102	17118011	19600414	2	20130102	8
20502925	20110406	15238814	19371202	2	20110406	8
20505275	20130523	17555127	19390309	1	20130523	7
20515804	20121024	16909293	19571030	1	20121024	7
20528012	20131118	18121341	19410624	2	20131118	7
20543695	20110118	15021062	19250707	1	20110118	7
20566476	20131116	18116566	19631130	1	20131116	7
20570110	20110111	15001003	19490120	1	20110111	10
20579231	20111026	15829580	19540930	1	20111026	7
20581311	20110221	15109931	19630819	1	20110221	10
20584525	20130804	17780693	19460218	1	20130804	7
20604482	20110603	15411739	19280404	1	20110603	9
20612037	20131110	18094950	19691124	2	20131110	7
20629043	20130414	17431633	19431228	1	20130414	9
20656762	20111212	15969683	19310514	2	20111212	7
20663267	20110831	15666896	19290430	1	20110831	9
20663756	20110406	15237901	19300204	1	20110406	10
20667190	20110830	15663427	19620519	1	20110830	8
20680368	20110414	15266026	19521113	2	20110414	8
20704512	20111011	15786545	19320208	2	20111011	8
20710809	20110224	15120227	19410209	1	20110224	7

20745113	20110307	15153297	19300227	2	20110307	7
20753633	20120920	16808500	19331225	2	20120920	7
20758843	20110912	15702361	19510205	1	20110912	8
20759868	20120411	16322780	19831006	2	20120411	8
20762190	20110222	15112626	19690424	2	20110222	8
20765257	20110717	15539254	19380315	1	20110717	7
20834246	20110430	15308538	19500417	1	20110430	7
2084166120130530		17572440	19440601	2	20130530	8
20892482	20110725	15561250	19270913	2	20110725	8
2093453620130110		17152750	19550428	1	20130110	7
2094198420130623		17646941	19310510	1	20130623	7
20950430	20120709	16587338	19420102	2	20120709	7
20950918	20110920	15726446	19270515	1	20110920	10
20961802	20101225	14954264	19490401	2	20101225	7
20965542	20110624	15471006	19241016	1	20110624	9
20973346	20110224	15121228	19460226	1	20110224	9
20977097	20110815	15624625	19311009	1	20110815	8
2098551720131113		18107301	19450203	2	20131113	7
20991199	20121214	17074096	19621024	2	20121214	7
21006804	20110119	15024331	19870721	1	20110119	7
21038517	20120402	16291666	19430414	1	20120402	7
2108348720130422		17455868	19530228	2	20130422	7
2108429720130228		17286733	19610903	1	20130228	7
2108706920130322		17362078	19541019	2	20130322	9
21087989	20110607	15419738	19490411	2	20110607	8
21098168	20110421	15285674	19360904	2	20110421	8
21101008	20110729	15573139	19270501	1	20110729	7
21102965	20120629	16554219	19491208	2	20120629	7
21112470	20120108	16047603	19690720	2	20120108	7
2113030320131116		18117725	19221001	2	20131116	7
21151882	20110523	15378473	19420715	1	20110523	8
21154847	20110628	15478156	19481029	1	20110628	9
21161762	20120828	16736121	19610428	1	20120828	7
2118349320131022		18033577	19501217	1	20131022	7
21205185	20120708	16584224	19650209	2	20120708	10
21207772	20120727	16643073	19220506	1	20120727	8
21218235	20120904	16756262	19350326	1	20120904	7
21225796	20120912	16785524	19580817	1	20120912	7
21239907	20120827	16732957	19270904	1	20120827	7
21248964	20111204	15943430	19280301	1	20111204	8
21261234	20110517	15363293	19280707	2	20110517	10
21267538	20120423	16354803	19650502	2	20120423	7
21273665	20120808	16678392	19620409	1	20120808	7
2127472620131101		18063321	19290226	1	20131101	7
2127875120130121		17183623	19560210	1	20130121	7
21286055	20121122	17001206	19311212	2	20121122	7
21324390	20110222	15113779	19311209	1	20110222	8
2133216120130628		17663459	19311018	1	20130628	7
21332854	20110620	15457042	19881211	2	20110620	10
21373397	20121121	16996793	19660515	1	20121121	7
21377026	20120711	16597157	19550910	1	20120711	10
2138968420130522		17548708	19890427	2	20130522	8
21430375	20110424	15292521	19351208	2	20110424	7
21438482	20110213	15084451	19471202	1	20110213	9
21461494	20120523	16446072	19350908	1	20120523	8

21461530	20130609	17605407	19310115	1	20130609	8
21487069	20110516	15358380	19511217	2	20110516	7
21487763	20121130	17022157	19290622	1	20121130	7
21489850	20110823	15647940	19410929	1	20110823	8
21493925	20120216	16159008	19400506	2	20120216	7
21495385	20110323	15202833	19340218	2	20110323	8
21512538	20130114	17162662	19751108	2	20130114	7
21513020	20120909	16773048	19381101	1	20120909	7
21516610	20120704	16572638	19751002	1	20120704	8
21519437	20110904	15678992	19350219	1	20110904	8
21527286	20130721	17738239	19490212	2	20130721	9
21531204	20111005	15769812	19280920	1	20111005	7
21531351	20110414	15267585	19680109	2	20110414	7
21536232	20110917	15718311	19870510	2	20110917	10
21538625	20110223	15117649	19660409	1	20110223	8
21544456	20110304	15143985	19500317	1	20110304	7
21552523	20121127	17012063	19300215	1	20121127	10
21556810	20110718	15542886	19250601	1	20110718	9
21575268	20110420	15282879	19470216	2	20110420	10
21626120	20110423	15292040	19480202	1	20110423	10
21634695	20120902	16746753	19680713	2	20120902	8
21655958	20121122	17001075	19601105	1	20121122	7
21738114	20110316	15183125	19560804	1	20110316	10
21741651	20120626	16544739	19581212	1	20120626	7
21764001	20111025	15827487	19600125	1	20111025	7
21774196	20110307	15154872	19510120	1	20110307	7
21822913	20110329	15215608	19360324	2	20110329	10
21824919	20120928	16828972	19491115	1	20120928	8
21850602	20110928	15747491	19710210	2	20110928	8
21862339	20130323	17365443	19350328	2	20130323	7
21869658	20120206	16124014	19341024	1	20120206	7
21877098	20110304	15144017	19290214	1	20110304	10
21878411	20131118	18122251	19320727	1	20131118	7
21880035	20130804	17780840	19331105	1	20130804	8
21884399	20110420	15282012	19561231	1	20110420	7
21890824	20120821	16717664	19730926	1	20120821	10
21896479	20110614	15443048	19481201	2	20110614	9
21906558	20110930	15753025	19401201	2	20110930	8
21936867	20111006	15775844	19370518	1	20111006	8
21937462	20121025	16911697	19440118	1	20121025	7
21941662	20110112	15004587	19690322	1	20110112	8
21943759	20130328	17379462	19560210	1	20130328	9
21946872	20110516	15360207	19410223	1	20110516	7
21951688	20110807	15600523	19620126	1	20110807	8
21960838	20111125	15919936	19370116	2	20111125	10
21972430	20111031	15840189	19600229	1	20111031	8
21978518	20111215	15981596	19660101	2	20111215	7
21983437	20110406	15237528	19470315	1	20110406	7
21989582	20130221	17267756	19490310	1	20130221	8
21994649	20131107	18085990	19300808	1	20131107	8
21997057	20120417	16340155	19240309	1	20120417	9
22002535	20121208	17051960	19640318	1	20121208	8
22004462	20130527	17563039	19460105	1	20130527	8
22017034	20110612	15434514	19250420	1	20110612	9
22046157	20130731	17766620	19450610	2	20130731	9

22101128	20110410	15249857	19361026	1	20110410	8
2211359320130708		17698704	19321108	1	20130708	7
22148909	20110308	15158889	19650325	2	20110308	7
2216941120130308		17321057	19560626	1	20130308	7
22177895	20110209	15069932	19600326	2	20110209	7
22182112	20110803	15590218	19421204	2	20110803	8
22189420	20120905	16761548	19460320	2	20120905	8
2219815820130923		17937374	19280522	1	20130923	8
22231670	20120319	16253423	19510910	1	20120319	9
22261423	20110320	15192536	19850628	2	20110320	8
22333944	20110627	15476884	19571228	2	20110627	8
22334072	20111013	15794214	19410207	1	20111013	8
22334958	20110603	15411698	19401126	1	20110603	8
2233762820130408		17411416	19480225	2	20130408	7
22343766	20120130	16097484	19310503	1	20120130	7
22346992	20121001	16837538	19650206	2	20121001	8
22373893	20120331	16284855	19320715	1	20120331	10
2240115620130730		17762544	19400725	1	20130730	8
22421836	20120905	16760907	19420120	1	20120905	8
22443396	20110219	15105814	19371125	2	20110219	9
22448095	20111016	15800846	19250114	1	20111016	8
22448108	20110730	15574356	19280817	1	20110730	8
22449907	20110824	15650958	19601226	1	20110824	10
22456060	20120103	16029128	19390417	2	20120103	7
2246303220131031		18058666	19490301	1	20131031	8
22469609	20101014	14746013	19591022	2	20101014	9
22472102	20120413	16330465	19311016	2	20120413	7
22480804	20110228	15128285	19620202	2	20110228	8
22487134	20110303	15142002	19260205	1	20110303	8
22498391	20120210	16139820	19600517	1	20120210	7
22509279	20120425	16362110	19440715	1	20120425	8
2251030120121207		17049963	19370428	1	20121207	9
22529755	20110302	15134615	19220921	2	20110302	10
22532510	20120321	16259069	19421028	2	20120321	7
22537797	20110110	14995642	19530524	1	20110110	8
22544098	20120119	16084195	19280915	2	20120119	7
2259228520130910		17901137	19720110	2	20130910	8
2260008420130708		17697652	19660928	2	20130708	7
22625065	20110324	15204893	19250921	1	20110324	7
2263744120130412		17429311	19540511	1	20130412	7
22639209	20120603	16474770	19601201	1	20120603	7
22648120	20120720	16624910	19250818	1	20120720	7
22655169	20111207	15957097	19641221	2	20111207	7
2265768720121122		17000231	19500228	1	20121122	7
22670673	20110406	15234896	19481202	1	20110406	7
22710221	20120312	16233934	19310718	2	20120312	8
22710594	20120723	16629979	19420611	1	20120723	9
22736998	20120529	16460392	19221225	1	20120529	7
22747724	20120731	16648967	19920529	2	20120731	8
2275537920130122		17188147	19350108	2	20130122	8
22755675	20111214	15977896	19290826	2	20111214	8
22769397	20110113	15009437	19290626	1	20110113	9
2279231020131120		18128796	19410129	2	20131120	7
22814206	20111026	15829407	19600628	1	20111026	8
22817998	20110523	15376510	19271103	1	20110523	8

2283005320130523		17554048	19270824	1	20130523	7
22835127	20110525	15383674	19291021	1	20110525	7
22844753	20120807	16675029	19300524	1	20120807	8
22852228	20110322	15199713	19340913	1	20110322	7
22860997	20110418	15275463	19490115	1	20110418	10
2286356520121218		17084146	19541020	1	20121218	8
2287641120130821		17838219	19381028	2	20130821	8
22884180	20110717	15538953	19190228	1	20110717	8
22909746	20120229	16188149	19430915	1	20120229	8
22927635	20121004	16851727	19330705	1	20121004	8
2298105120130712		17712740	19711126	1	20130712	8
23005529	20110318	15189844	19321211	1	20110318	8
23034040	20110829	15661661	19350615	2	20110829	8
2304321220130711		17709809	19530902	1	20130711	8
23045536	20120208	16132106	19470315	2	20120208	8
23072028	20110918	15719450	19581105	1	20110918	7
23084653	20110419	15280639	19680118	2	20110419	9
23085576	20110420	15284101	19641214	2	20110420	10
2310114620130725		17752631	19580329	2	20130725	7
23105284	20120722	16626946	19400405	2	20120722	8
2311474120130118		17178160	19541201	2	20130118	7
23125895	20111130	15930397	19391125	1	20111130	7
23127733	20111229	16018183	19221127	1	20111229	10
23129364	20120504	16392692	19570226	1	20120504	7
2313115920131025		18043936	19480615	1	20131025	8
23132323	20110628	15480407	19271201	1	20110628	8
2319213420131127		18146979	19270705	1	20131127	7
23198109	20101223	14949629	19340101	1	20101223	7
23238804	20110109	14994250	19330316	1	20110109	7
23246880	20120920	16809351	19650318	2	20120920	8
2325213320130908		17894011	19520401	2	20130908	7
23253874	20110120	15028172	19491130	1	20110120	8
23275049	20101229	14963754	19420717	1	20101229	8
23309151	20121213	17070598	19480523	1	20121213	8
23356992	20110717	15538993	19710725	1	20110717	9
23367819	20110218	15104271	19580201	2	20110218	10
2337466520130505		17496120	19361017	1	20130505	7
23389948	20121115	16979056	19500304	2	20121115	7
23413389	20110510	15343925	19471103	1	20110510	8
23426199	20111121	15907176	19431128	1	20111121	8
23432726	20111003	15761021	19461225	1	20111003	10
23440440	20120104	16033138	19580312	1	20120104	9
23467892	20121021	16898873	19530115	1	20121021	7
23479256	20110107	14990974	19331009	1	20110107	7
23479552	20111218	15986985	19290105	1	20111218	7
2348022020131023		18037044	19660527	1	20131023	7
23492151	20110105	14984177	19430109	2	20110105	8
2353357520130613		17618524	19570906	1	20130613	8
23535957	20120107	16046933	19360816	1	20120107	7
23537113	20120205	16119611	19560303	2	20120205	10
23601265	20110117	15017794	19580623	1	20110117	10
23648740	20120927	16826322	19731119	2	20120927	8
23650557	20111227	16012367	19271211	1	20111227	7
23657445	20110306	15149932	19500210	2	20110306	8
23706834	20110127	15046491	19250914	1	20110127	8

23764854	20101224	14953174	19270606	1	20101224	8
23782641	20110210	15076534	19590920	1	20110210	8
23784818	20110129	15050595	19400921	2	20110129	8
23789824	20101226	14954978	19490317	2	20101226	7
23823101	20130423	17459079	19550420	2	20130423	7
23829198	20110423	15292007	19350802	1	20110423	10
23844442	20120616	16520000	19180105	2	20120616	9
23885838	20121007	16858138	19480712	2	20121007	10
23886999	20111019	15810667	19510528	1	20111019	8
23908567	20130907	17893395	19550504	1	20130907	8
23953824	20130127	17200078	19650102	1	20130127	7
24042873	20121012	16875167	19651211	2	20121012	8
24045623	20130103	17128244	19470508	1	20130103	8
24059754	20111203	15941796	19530105	2	20111203	8
24070017	20110612	15434364	19521015	2	20110612	7
24098440	20110321	15194820	19340303	1	20110321	8
24124558	20110506	15330918	19660912	2	20110506	8
24140098	20111013	15794662	19410427	2	20111013	8
24175777	20110805	15598354	19391112	2	20110805	10
24192389	20131128	18151495	19700529	2	20131128	7
24203938	20110420	15282000	19370224	2	20110420	8
24210159	20120405	16304167	19470710	2	20120405	10
24215314	20120529	16459834	19370313	2	20120529	9
24238082	20110106	14985406	19500720	1	20110106	10
24247050	20110120	15028279	19280701	1	20110120	7
24247867	20120729	16645409	19391013	2	20120729	8
24265325	20121126	17008959	19300924	2	20121126	8
24306409	20110117	15018664	19670324	1	20110117	8
24318089	20110927	15744624	19330627	2	20110927	8
24356885	20130714	17716192	19450324	2	20130714	8
24369117	20120924	16816629	19530826	1	20120924	8
24415701	20120430	16371476	19651216	1	20120430	7
24447583	20130117	17174683	19520730	1	20130117	9
24457532	20120929	16831219	19480602	2	20120929	8
24459630	20110307	15154777	19320227	2	20110307	8
24459776	20111018	15807374	19740226	1	20111018	10
24473107	20110114	15010897	19590901	1	20110114	8
24487090	20111205	15946067	19330204	1	20111205	10
24552021	20130116	17169924	19470826	1	20130116	8
24581235	20120214	16151284	19250326	1	20120214	7
24588305	20120312	16232899	19380815	2	20120312	8
24592436	20110302	15139397	19590708	2	20110302	10
24593780	20130226	17281828	19280217	2	20130226	7
24661470	20120225	16181300	19580115	1	20120225	8
24671714	20111215	15980846	19580520	2	20111215	7
24686666	20110716	15538459	19240119	1	20110716	8
24709839	20120723	16630435	19730210	2	20120723	8
24729360	20110419	15279873	19531024	2	20110419	8
24732841	20120803	16662218	19541011	1	20120803	10
24762843	20130926	17948938	19510110	2	20130926	8
24768330	20110928	15747391	19711227	2	20110928	7
24771504	20110829	15661532	19940420	1	20110829	10
24793144	20130119	17179818	19371128	2	20130119	7
24793166	20110215	15092259	19270319	1	20110215	9
24802617	20110817	15633091	19621110	1	20110817	8

2481083120130315		17341093	19241112	1	20130315	7
24812860	20110624	15471371	19371025	2	20110624	10
2481626020130510		17516912	19200203	2	20130510	7
24904356	20110215	15091854	19411018	1	20110215	8
2490589320130902		17869377	19550809	1	20130902	7
24906103	20110406	15238016	19631101	1	20110406	10
24913040	20120208	16129313	19311110	1	20120208	8
24915739	20120702	16562915	19551110	2	20120702	8
24923408	20110228	15128047	19380616	1	20110228	8
24929837	20110811	15615663	19660625	1	20110811	7
24943984	20120130	16098192	19251114	1	20120130	9
24952087	20120521	16440600	19331201	1	20120521	9
2495516620130123		17191665	19351010	2	20130123	8
24956896	20120319	16254224	19640922	1	20120319	8
24989137	20120611	16503831	19401001	2	20120611	8
25006171	20111012	15791457	19540425	1	20111012	8
25022928	20120207	16127764	19550507	1	20120207	8
25030857	20110124	15036203	19270611	1	20110124	7
25045323	20110517	15362138	19640928	2	20110517	8
25053865	20110808	15603974	19240505	1	20110808	8
2509120720130515		17529534	19580923	1	20130515	8
25093214	20110413	15261972	19211020	1	20110413	8
25097078	20110320	15192525	19380307	2	20110320	8
25111426	20110617	15453736	19290109	1	20110617	7
25135051	20120203	16115079	19261016	2	20120203	7
2515114820130101		17117482	19340625	2	20130101	7
25155651	20110713	15530623	19281104	1	20110713	7
25156869	20110422	15290585	19530724	2	20110422	7
25185937	20110901	15670453	19311103	1	20110901	7
25216608	20110822	15644078	19260624	1	20110822	7
2521693720130530		17572412	19671210	1	20130530	7
25242211	20111101	15845181	19621101	1	20111101	9
2525752720130224		17273982	19570130	2	20130224	8
25257754	20120222	16175096	19450504	2	20120222	10
25291729	20120504	16392187	19720315	1	20120504	7
25300167	20110427	15302367	19201022	1	20110427	7
25335244	20121218	17085187	19620901	1	20121218	9
25335299	20120405	16303516	19600404	1	20120405	10
25345613	20110630	15486088	19300825	2	20110630	8
25361426	20120319	16254175	19330615	1	20120319	7
25398747	20110510	15343334	19440117	1	20110510	8
2541201520130725		17751770	19680423	2	20130725	7
2542874420131101		18063368	19270416	1	20131101	8
25433390	20110106	14988190	19670207	2	20110106	9
25433845	20110621	15462675	19340321	1	20110621	10
25440113	20110801	15580656	19690408	2	20110801	8
2544244820130909		17898670	19430119	2	20130909	8
2544740920130608		17604995	19530331	2	20130608	7
25457425	20110724	15557705	19240505	1	20110724	8
25462811	20110827	15658339	19540802	1	20110827	8
25471550	20110109	14994430	19540825	2	20110109	10
25476259	20101220	14940279	19561115	1	20101220	7
25507339	20110724	15557808	19420409	1	20110724	10
2552306220121220		17092622	19440703	1	20121220	7
25524145	20120207	16129263	19370803	2	20120207	8

25524189	20110627	15477260	19670419	2	20110627	8
25547482	20110819	15639356	19400209	2	20110819	8
25552618	20110608	15423403	19260106	1	20110608	8
25574178	20110922	15732781	19520824	2	20110922	8
25578896	20110213	15084737	19540406	1	20110213	8
25605327	20110619	15455923	19530428	1	20110619	7
25620013	20110205	15059654	19600716	2	20110205	8
25627263	20110726	15563445	19610414	1	20110726	7
25632433	20110215	15091187	19291202	2	20110215	7
25640475	20120301	16195527	19771024	1	20120301	7
25643689	20110828	15658881	19410208	1	20110828	7
25647352	20110906	15687161	19561207	1	20110906	7
2568006220130808		17798239	19330706	1	20130808	9
25704807	20111228	16014455	19341205	2	20111228	10
25779128	20110621	15462990	19470420	1	20110621	10
2579255620131222		18228208	19791009	2	20131222	7
25802453	20111115	15891029	19460108	1	20111115	8
25808371	20110115	15014280	19481022	2	20110115	10
25811852	20111018	15806595	19421108	2	20111018	10
25834360	20120314	16241654	19531011	2	20120314	7
25837529	20120314	16240502	19381017	1	20120314	7
2585360520130514		17526850	19801015	2	20130514	7
25874333	20110529	15392081	19310115	1	20110529	7
2588634420130828		17857926	19290102	2	20130828	8
25926812	20120821	16718514	19480621	1	20120821	7
25935380	20110301	15134405	19380502	2	20110301	8
25951182	20110830	15663323	19640824	1	20110830	10
25972047	20120216	16157321	19371118	2	20120216	10
25982187	20110228	15127933	19540623	1	20110228	8
2598842520130807		17795994	19681211	1	20130807	7
25992749	20110220	15106430	19700310	2	20110220	7
26013707	20120705	16576652	19250605	1	20120705	9
26023916	20120210	16140773	19330119	2	20120210	10
26061529	20101215	14926630	19660609	1	20101215	8
26068928	20110907	15691020	19920803	1	20110907	9
26123682	20111028	15835880	19520823	2	20111028	9
26153139	20110318	15190434	19320123	1	20110318	7
26157426	20110215	15093290	19790509	2	20110215	7
26174969	20111004	15763180	19281108	2	20111004	10
2617948620130412		17428339	19911110	2	20130412	8
26185875	20110403	15228886	19541119	1	20110403	8
26187235	20110419	15279241	19480730	2	20110419	7
26213467	20121127	17013377	19570503	2	20121127	8
2622620020130410		17417426	19340914	2	20130410	7
26241532	20111212	15968217	19671127	2	20111212	8
26246800	20110405	15233627	19270209	1	20110405	7
26248191	20111026	15830295	19491209	2	20111026	8
2626123420131025		18041705	19690716	1	20131025	9
26265941	20110125	15040908	19290807	1	20110125	8
26269705	20120129	16094522	19260613	1	20120129	7
26290915	20120602	16474088	19421025	1	20120602	7
26294031	20121107	16951626	19560831	1	20121107	8
26299729	20111205	15948046	19461028	2	20111205	10
2631005420130124		17194353	19540811	1	20130124	8
26336685	20110622	15464076	19641207	1	20110622	10

26350403	20111130	15929134	19561121	2 20111130	8
26412179	20120213	16145871	19530824	1 20120213	7
26430546	20111217	15986627	19501226	1 20111217	8
26453929	20110128	15049471	19380918	1 20110128	7
26460924	20111226	16007448	19150327	1 20111226	7
26474237	20110930	15753437	19390108	1 20110930	7
26484402	20110522	15375617	19311021	2 20110522	8
26491554	20131106	18083194	19340301	2 20131106	7
26503606	20110224	15120662	19721207	2 20110224	8
26505975	20110714	15533953	19390328	2 20110714	8
26508258	20110312	15172243	19711124	2 20110312	8
26528905	20121203	17028682	19530920	1 20121203	9
26537906	20111007	15780263	19450203	2 20111007	9
26556503	20101227	14957942	19260804	2 20101227	10
26567282	20130521	17548170	19680321	2 20130521	7
26577786	20131117	18118263	19671225	1 20131117	7
26585386	20120925	16821564	19481203	2 20120925	7
26592303	20120518	16434110	19290216	2 20120518	7
26593135	20130823	17845268	19521224	2 20130823	7
26608719	20130317	17345729	19690417	2 20130317	7
26638584	20121022	16900329	19430506	2 20121022	10
26647734	20130826	17849814	19810806	2 20130826	7
26658764	20120819	16711698	19770813	2 20120819	7
26692795	20110328	15210709	19641019	2 20110328	8
26721697	20120208	16132990	19421210	2 20120208	8
26728110	20120813	16693940	19410220	1 20120813	10
26760576	20110326	15209828	19360409	2 20110326	10
26773193	20120225	16181247	19251024	2 20120225	7
26783426	20120812	16690466	19840620	2 20120812	10
26815554	20110720	15550092	19250716	2 20110720	7
26832122	20130901	17864898	19741205	2 20130901	8
26856644	20120210	16140706	19360318	2 20120210	8
26889623	20120519	16436090	19630917	1 20120519	10
26900594	20111122	15910014	19300728	1 20111122	7
26930994	20110506	15331302	19490216	1 20110506	8
26934112	20110209	15069928	19430726	1 20110209	7
26941640	20130109	17148980	19600526	1 20130109	7
26948436	20110722	15556139	19321004	1 20110722	8
26951188	20120224	16180682	19490331	2 20120224	7
26978518	20110415	15269195	19570601	1 20110415	8
27013689	20120704	16573316	19550116	2 20120704	8
27063656	20120108	16047648	19291003	2 20120108	7
27065174	20110725	15561123	19390202	2 20110725	7
27079465	20130528	17566713	19600922	1 20130528	7
27087509	20110310	15166622	19310224	2 20110310	8
27110312	20120726	16638495	19461017	1 20120726	8
27115395	20130411	17423580	19400428	2 20130411	7
27118918	20110424	15292416	19250530	1 20110424	9
27126927	20110303	15143487	19421123	2 20110303	8
27134732	20120507	16399433	19760527	2 20120507	8
27142058	20130718	17732725	19461025	2 20130718	8
27143993	20120227	16184765	19491024	1 20120227	8
27159020	20130716	17724131	19791114	2 20130716	7
27167664	20130604	17588036	19600726	1 20130604	8
27219096	20111003	15761582	19500225	1 20111003	8

27238193	20110210	15078109	19890529	2	20110210	8
27258146	20130922	17934073	19440927	1	20130922	7
27259558	20110405	15233894	19331109	2	20110405	8
27285058	20110228	15128151	19490725	1	20110228	10
27314541	20131111	18098534	19500423	1	20131111	7
27317368	20110818	15636371	19380531	2	20110818	8
27322458	20111012	15791146	19491001	1	20111012	8
27323428	20121111	16963513	19490602	1	20121111	7
27336181	20110928	15748017	19410401	2	20110928	8
27348352	20110528	15391758	19281006	1	20110528	9
27359440	20120416	16336114	19980526	2	20120416	10
27372709	20110415	15270600	19890407	1	20110415	10
27374896	20130819	17832386	19380228	2	20130819	8
27405272	20111112	15882792	19601217	1	20111112	8
27407029	20100913	14653595	19471015	1	20100913	10
27421609	20110719	15545428	19661219	2	20110719	10
27425963	20120510	16411572	19320721	1	20120510	7
27430520	20110522	15375558	19410915	2	20110522	10
27433803	20110224	15119742	19421209	2	20110224	9
27434088	20121222	17096940	19490502	1	20121222	8
27452886	20131016	18015777	19540203	2	20131016	7
27471698	20120508	16404030	19380617	2	20120508	7
27471994	20110110	14998914	19320818	2	20110110	8
27473218	20110727	15567642	19400110	2	20110727	8
27478177	20110522	15375351	19280108	2	20110522	8
27556145	20131123	18137547	19300723	1	20131123	7
27561995	20120917	16796353	19340525	2	20120917	7
27562261	20110609	15427549	19530921	1	20110609	7
27582281	20111028	15834768	19391112	2	20111028	8
27596356	20130215	17248192	19340827	1	20130215	8
27609967	20120615	16518083	19490526	1	20120615	8
27626182	20110320	15192504	19641225	1	20110320	8
27635887	20120211	16142871	19340114	2	20120211	10
27667329	20110929	15750656	19411005	1	20110929	8
27678724	20110810	15613028	19590410	2	20110810	7
27698802	20110508	15334967	19341220	1	20110508	10
27701526	20111109	15874571	19560110	2	20111109	7
27702212	20120910	16776870	19410128	1	20120910	7
27713968	20130329	17381331	19440704	1	20130329	7
27715055	20130314	17340256	19410415	2	20130314	8
27720907	20110118	15021747	19370330	2	20110118	8
27723224	20130702	17675414	19261122	1	20130702	8
27741828	20101216	14930895	19691106	1	20101216	10
27787946	20111105	15862052	19631212	1	20111105	10
27805147	20130411	17423814	19520818	2	20130411	7
27813010	20121105	16941566	19520924	1	20121105	7
27819687	20120827	16732692	19780613	2	20120827	9
27827107	20130821	17838807	19610827	2	20130821	7
27891992	20131113	18108200	19570120	2	20131113	7
27904423	20130118	17178146	19460802	1	20130118	8
27911928	20120702	16561212	19520129	1	20120702	10
27916945	20110809	15608942	19380227	2	20110809	10
27955482	20121201	17024540	19300916	2	20121201	7
27987855	20130626	17655985	19521229	2	20130626	7
27996663	20121125	17006368	19291201	1	20121125	10

27999902	20110508	15335171	19390827	2	20110508	9
28011496	20111221	15997323	19570806	2	20111221	7
2804075720130315		17343087	19410316	1	20130315	7
2807148920130131		17212250	19621209	1	20130131	7
28082317	20120312	16233266	19350702	2	20120312	7
28091943	20120202	16112887	19990204	1	20120202	8
28110089	20121106	16945838	19650328	1	20121106	9
28133019	20110102	14969648	19730308	2	20110102	9
28136416	20121101	16930159	19451129	1	20121101	7
2815303920130905		17886850	19360819	2	20130905	7
28165404	20111206	15951516	19491127	2	20111206	10
28179079	20110405	15233385	19280123	1	20110405	10
2819911320130520		17543334	19591212	2	20130520	8
28204173	20120709	16586945	19690616	2	20120709	10
28213710	20110605	15414474	19270321	1	20110605	8
28214655	20110228	15128386	19530622	2	20110228	7
28228719	20120505	16394577	19540403	1	20120505	7
28237618	20120601	16470579	19550217	1	20120601	8
28239283	20110823	15647946	19300227	1	20110823	8
28239443	20110410	15249647	19200619	1	20110410	7
2825092220130326		17373032	19500811	2	20130326	7
28264053	20111213	15974421	19360222	1	20111213	9
2827266420131113		18107787	19260108	2	20131113	7
28277534	20120610	16500243	19710519	1	20120610	7
28280491	20110826	15656380	19370615	2	20110826	10
28285338	20110407	15242806	19570622	1	20110407	7
28287061	20120314	16237873	19351226	2	20120314	8
28302376	20111016	15800757	19490102	1	20111016	8
28312052	20101230	14966044	19560415	1	20101230	9
28349862	20110107	14988462	19320823	2	20110107	7
28349964	20110214	15085671	19630924	2	20110214	10
28357928	20110102	14969774	19590418	1	20110102	8
2835832920121229		17114512	19530114	1	20121229	8
2836086320130621		17643790	19501226	1	20130621	8
2838125120130421		17452968	19300723	1	20130421	7
28386983	20110622	15466039	19470105	2	20110622	8
28389197	20120528	16458032	19460425	2	20120528	8
28389619	20120310	16227259	19390914	2	20120310	7
28396909	20121016	16885397	19251109	2	20121016	7
28398267	20120702	16557915	19630228	1	20120702	8
2843919220131030		18054155	19611116	2	20131030	7
28441045	20110222	15112917	19660402	1	20110222	7
28451129	20110126	15043553	19310821	1	20110126	8
28460233	20120411	16323692	19590508	1	20120411	10
2846611720130622		17646629	19661224	2	20130622	8
28491432	20111201	15935050	19660409	2	20111201	10
28492082	20121125	17006465	19400308	2	20121125	7
28504270	20110811	15615433	19650923	1	20110811	7
2850540020130120		17180588	19490225	1	20130120	7
28508681	20120328	16278298	19480515	1	20120328	10
28510545	20110219	15105755	19980423	2	20110219	10
28514207	20110424	15292360	19610910	1	20110424	9
28530736	20111219	15989125	19791027	1	20111219	8
2853130820130818		17829200	19550601	2	20130818	7
28537602	20120326	16270894	19480619	1	20120326	9

28538627	20110216	15096051	19600620	1	20110216	7
28541857	20131019	18024808	19460622	2	20131019	8
28544845	20110213	15084615	19550921	1	20110213	8
28550687	20120827	16731562	19310525	2	20120827	7
28560465	20110319	15191002	19361218	2	20110319	10
28562530	20131026	18045150	19720824	1	20131026	8
28575577	20120608	16496089	19280105	1	20120608	7
28580101	20110811	15615088	19390217	2	20110811	7
28664439	20110830	15663910	19310722	1	20110830	8
28669069	20120604	16479885	19560125	1	20120604	7
28718312	20110411	15253465	19790514	2	20110411	10
28726945	20130930	17957559	19391210	2	20130930	7
28767842	20111105	15861751	19560915	2	20111105	10
28795473	20131121	18132518	19611025	1	20131121	8
28818088	20110731	15574931	19770607	2	20110731	8
28839385	20121016	16885413	19560705	1	20121016	7
28840564	20110814	15622364	19321110	2	20110814	10
28844726	20111227	16011367	19371104	2	20111227	8
28881416	20120713	16602676	19401004	2	20120713	7
28885861	20110423	15291932	19360726	1	20110423	8
28885974	20110922	15733485	19571124	1	20110922	10
28912858	20111218	15987127	19730522	1	20111218	9
28977084	20130530	17571855	19650529	2	20130530	7
28983520	20111031	15840171	19430423	1	20111031	8
29032700	20110715	15536921	19390828	1	20110715	7
29045327	20130102	17122707	19421123	1	20130102	9
29065825	20110801	15580974	19290211	1	20110801	8
29147215	20120904	16757492	20000912	1	20120904	8
29197577	20110314	15176626	19390730	2	20110314	10
29204271	20111127	15921750	19610828	2	20111127	7
29208795	20101230	14965099	19600808	1	20101230	8
29258079	20111228	16015561	19370122	1	20111228	7
29270959	20101226	14955093	19691010	2	20101226	10
29293149	20110220	15106122	19410504	1	20110220	8
29298699	20131104	18072453	19360610	2	20131104	7
29316903	20110308	15158842	19400418	1	20110308	7
29337084	20130822	17841007	19250601	2	20130822	8
29350274	20111031	15840063	19521104	1	20111031	7
29379860	20110904	15678734	19560514	2	20110904	8
29410988	20120822	16722123	19700831	2	20120822	7
29413863	20110615	15445769	19410323	2	20110615	10
29422999	20110530	15394530	19430907	1	20110530	8
29444915	20110226	15125566	19330619	2	20110226	8
29448473	20131005	17981691	19760211	2	20131005	7
29454475	20130703	17680739	19641009	2	20130703	7
29486393	20101228	14960492	19490402	2	20101228	7
29491245	20130508	17509143	19521102	1	20130508	7
29496795	20110116	15014570	19570130	2	20110116	9
29496842	20121116	16982363	19440122	1	20121116	7
29511657	20110616	15450464	19510925	1	20110616	8
29531826	20110328	15213553	19360201	2	20110328	8
29547602	20110104	14979401	19641020	2	20110104	7
29549131	20110514	15356251	19320630	2	20110514	7
29566232	20130309	17323773	19521229	1	20130309	7
29619429	20110313	15172691	19421111	2	20110313	8

29625670	20120601	16471813	19600220	1	20120601	7
29629898	20121026	16912997	19591019	2	20121026	8
29659914	20110426	15298083	19390206	1	20110426	8
29667650	20110618	15455317	19740418	2	20110618	9
29672717	20121129	17018842	19351120	2	20121129	8
29673389	20110420	15281474	19600106	2	20110420	7
29677778	20111229	16017318	19271225	1	20111229	8
29682733	20110427	15302092	19531227	2	20110427	8
29684444	20110401	15224201	19340927	1	20110401	7
29684853	20121003	16845356	19480120	2	20121003	7
29700401	20110116	15014393	19711026	1	20110116	7
29711635	20110823	15647990	19740923	2	20110823	8
29715126	20120509	16407832	19420715	2	20120509	7
29718749	20110108	14993800	19220101	1	20110108	8
29731122	20120220	16167523	19420416	2	20120220	9
29791319	20110906	15688315	19340811	2	20110906	10
29794410	20120503	16388586	19490105	1	20120503	7
29801841	20120215	16155717	19481122	1	20120215	10
29822944	20121028	16917170	19410128	2	20121028	8
29839529	20120630	16556445	19541016	1	20120630	7
29856960	20110525	15385260	19520102	2	20110525	9
2986276820130130		17209446	19351226	2	20130130	9
29863114	20110623	15468058	20000427	2	20110623	10
29882039	20120813	16693802	19500718	1	20120813	10
29902861	20120918	16801470	19520314	1	20120918	8
29904709	20121103	16937818	19310528	1	20121103	8
29936456	20110902	15676222	19300107	2	20110902	8
29939886	20121002	16839448	19510725	2	20121002	8
29940010	20110216	15097280	19451113	2	20110216	10
29943575	20120212	16143062	19790727	1	20120212	8
29958063	20110114	15012570	19620220	1	20110114	7
29980629	20101225	14954277	19540120	1	20101225	7
29987357	20110222	15113870	19360221	1	20110222	9
30003362	20121024	16909543	19360602	1	20121024	9
30006112	20110510	15343797	19320928	2	20110510	9
30016412	20110828	15658882	19350322	1	20110828	8
30052063	20111002	15756695	19640224	2	20111002	8
30067482	20110510	15343003	19460109	2	20110510	8
3008468520131211		18198376	19800722	2	20131211	7
30104166	20110113	15009414	19490217	2	20110113	8
30111810	20110805	15598468	19321110	1	20110805	8
30144140	20110519	15369155	19441220	1	20110519	7
30152331	20120605	16484606	19350114	1	20120605	7
30157756	20120126	16090218	19440119	2	20120126	8
30183234	20110724	15557869	19651027	1	20110724	8
30192348	20120705	16576263	19490116	2	20120705	7
30204547	20110410	15249835	19360516	2	20110410	7
30213139	20110306	15150235	19721206	1	20110306	10
30223326	20110430	15308391	19610908	1	20110430	9
3022941520130517		17538896	19470913	2	20130517	9
30232258	20110526	15387457	19300531	1	20110526	8
30237162	20110115	15013416	19670827	1	20110115	10
30238507	20110723	15557416	19260510	2	20110723	8
30259348	20120606	16489581	19230215	2	20120606	7
30295217	20111207	15955799	19611120	1	20111207	8

30313441	20120429	16369554	19500102	1	20120429	7
30323810	20120815	16701843	19420914	1	20120815	10
30324642	20101227	14957833	19530827	1	20101227	10
30335230	20111123	15912797	19620314	2	20111123	7
3038628820130113		17159541	19570712	2	20130113	7
30405233	20120927	16827523	19550309	1	20120927	7
30426416	20120610	16500116	19330718	2	20120610	8
30443482	20120814	16699155	19550901	1	20120814	7
30451184	20120828	16735559	19521227	2	20120828	8
30454401	20111113	15883143	19300801	1	20111113	8
30471057	20111126	15921200	19440510	1	20111126	7
30472152	20121210	17057735	19591104	1	20121210	9
30505330	20110303	15139549	19840406	2	20110303	10
30521676	20110309	15163105	19620626	2	20110309	9
30534066	20110802	15581102	19811126	2	20110802	9
30534679	20120812	16690356	19630608	2	20120812	8
30551474	20110726	15563846	19650308	1	20110726	7
30554111	20110528	15391583	19330508	2	20110528	7
3056606420131016		18014004	19611113	1	20131016	9
30570402	20121012	16875903	19330804	1	20121012	8
30589027	20120815	16702365	19670722	1	20120815	7
3059124320130812		17809301	19420802	2	20130812	8
30605415	20110529	15392283	19550402	2	20110529	9
30607751	20111024	15823316	19550112	1	20111024	10
30619751	20110417	15272572	19500920	2	20110417	7
3062385920131205		18177922	19370612	2	20131205	8
30631040	20110408	15246173	19990915	1	20110408	10
30647848	20110206	15060221	19520806	1	20110206	10
3066063020130809		17803467	19581018	1	20130809	8
30689226	20110722	15555071	19301003	1	20110722	8
30696630	20110119	15025402	19541215	2	20110119	8
30705169	20110902	15675686	19440915	2	20110902	7
30724255	20110526	15388153	19610906	1	20110526	10
30725225	20110926	15740710	19681220	2	20110926	7
3073609520130303		17296816	19710904	2	20130303	8
30736562	20120701	16556883	19370226	1	20120701	7
30738397	20110205	15059234	19530307	2	20110205	8
30767647	20120711	16597179	19410323	2	20120711	9
3078098420131207		18184712	19590607	2	20131207	7
30781147	20110414	15267045	19771007	1	20110414	7
30798959	20121113	16969973	19271015	1	20121113	7
30800250	20110208	15066981	19301011	1	20110208	10
30810914	20120911	16781194	19560220	1	20120911	8
30822005	20120313	16237832	19451206	1	20120313	8
30824692	20110720	15549970	19370304	1	20110720	8
30844805	20120430	16373006	19590605	2	20120430	8
3089687820130530		17572692	19431004	1	20130530	7
30903005	20121203	17031259	19521124	2	20121203	7
30916360	20110125	15040915	19610619	2	20110125	7
3091785220130522		17551332	19270626	2	20130522	8
30934306	20120924	16817953	19820202	2	20120924	7
30935263	20120826	16729712	19640817	1	20120826	9
30987258	20120529	16460846	19500330	1	20120529	7
3099227920130819		17831257	19600708	2	20130819	7
30992633	20101116	14842111	19560627	1	20101116	8

30998857	20130627	17660248	19470301	2	20130627	7
31002581	20120910	16777370	19360704	2	20120910	8
31006492	20131110	18095025	19540515	2	20131110	7
31015595	20111026	15828727	19570110	2	20111026	10
31027302	20130514	17526885	19520918	2	20130514	7
31058249	20110103	14975026	19751219	2	20110103	7
31074405	20131023	18035860	19540328	2	20131023	8
31078032	20110421	15285051	19341231	2	20110421	9
31078156	20110104	14979674	19611224	1	20110104	8
31104811	20130906	17890561	19300814	1	20130906	7
31109894	20120527	16454661	19560702	1	20120527	8
31140202	20130401	17390305	19370122	2	20130401	7
31142979	20110814	15622160	19450306	2	20110814	7
31157503	20110222	15113487	19530925	1	20110222	8
31158233	20131103	18066788	19670112	1	20131103	8
31165192	20111104	15859893	19490520	1	20111104	8
31186057	20130619	17635550	19551006	1	20130619	7
31203299	20130522	17549403	19530404	2	20130522	7
31209140	20131102	18066261	19290209	1	20131102	7
31214865	20110601	15403589	19451112	2	20110601	10
31216861	20110424	15292308	19661203	2	20110424	8
31235800	20121024	16909521	19381026	2	20121024	7
31243217	20120609	16499577	19230808	2	20120609	8
31254156	20110811	15615727	19410713	1	20110811	7
31256287	20130827	17852332	19370620	2	20130827	8
31261571	20110926	15741869	19620805	1	20110926	10
31272067	20110309	15162195	19321127	2	20110309	8
31277108	20110822	15644159	19790310	2	20110822	10
31285888	20130815	17822747	19911028	2	20130815	8
31290092	20120601	16470920	19701115	1	20120601	7
31298529	20101224	14953252	19311026	1	20101224	7
31316117	20110104	14977991	19901004	1	20110104	10
31331303	20120219	16164434	19670304	1	20120219	8
31339034	20110103	14972888	19860125	1	20110103	8
31345707	20130701	17670410	19531025	2	20130701	9
31355336	20120314	16240286	19420514	1	20120314	8
31362488	20130528	17567387	19521215	2	20130528	7
31363196	20110731	15575158	19710210	2	20110731	10
31382668	20110614	15442701	19371125	1	20110614	10
31386795	20120911	16781205	19360825	2	20120911	7
31396971	20120828	16735381	19760826	2	20120828	7
31403186	20130104	17132720	19180326	1	20130104	8
31417911	20120805	16665127	19620310	1	20120805	7
31425373	20110718	15542991	19330601	2	20110718	8
31448347	20131223	18231945	19551230	2	20131223	7
31456981	20131029	18052594	19691025	2	20131029	7
31465777	20120914	16791983	19400602	2	20120914	8
31486198	20130414	17431695	19611010	1	20130414	8
31495597	20121013	16877725	19231218	1	20121013	8
31526848	20110314	15176843	19481007	2	20110314	9
31537561	20130402	17394046	19350724	1	20130402	7
31538462	20111231	16021851	19500102	1	20111231	7
31547645	20131115	18116005	19470806	1	20131115	7
31549812	20120602	16474076	19520201	1	20120602	8
31561474	20130408	17410491	19651004	2	20130408	8

31567187	20110513	15353449	19681015	1	20110513	8
3159017720130517		17537429	19370117	2	20130517	9
31599027	20111225	16005777	19490104	1	20111225	7
3162579320130617		17627957	19340318	1	20130617	8
3163320220130617		17629066	19460424	1	20130617	8
3165385720130422		17456960	19841214	1	20130422	7
31656732	20110107	14990880	19481022	2	20110107	8
31678418	20110103	14975041	19570616	1	20110103	10
31684512	20120507	16399700	19550727	1	20120507	10
31698450	20121021	16899245	19490820	1	20121021	7
31703727	20110923	15735182	19510914	1	20110923	7
3172295920130331		17383723	19381112	1	20130331	8
31725118	20110308	15158423	20010822	2	20110308	10
31750046	20110109	14994369	19660908	2	20110109	9
31756828	20111109	15871563	19340323	1	20111109	8
3176565820130323		17365608	19331008	1	20130323	8
31785429	20110508	15335175	19480210	1	20110508	7
31792004	20120310	16229103	19701101	2	20120310	8
31831160	20110511	15347047	19420315	2	20110511	7
31872114	20120429	16369558	19630329	1	20120429	8
3187469820130520		17544872	19690418	1	20130520	8
31877835	20110317	15187191	19420925	2	20110317	7
31894970	20110219	15105690	19641124	2	20110219	8
31895326	20110829	15659516	19431229	1	20110829	7
31915534	20110710	15518393	19370331	2	20110710	10
31929825	20111005	15771364	19650925	1	20111005	8
31955790	20110220	15106276	19630124	2	20110220	9
31981336	20120217	16162358	19630522	1	20120217	8
31995478	20110409	15249549	19400512	2	20110409	8
31999607	20110530	15394754	19690426	2	20110530	10
32009908	20110427	15301851	19391013	2	20110427	8
32014690	20110207	15063330	19770129	2	20110207	7
32049186	20111030	15837789	19440805	2	20111030	10
3210836420130215		17247769	19250812	1	20130215	8
32116635	20120611	16503455	19720423	2	20120611	7
32121021	20120710	16590859	19430506	1	20120710	7
32122273	20110320	15192430	19430530	2	20110320	8
3213777220130302		17296140	19410408	1	20130302	9
3215286620130630		17665606	19340509	1	20130630	8
32153676	20120927	16827392	19250115	2	20120927	8
32159265	20121026	16914832	19380808	1	20121026	7
32160217	20120425	16361970	19390120	2	20120425	7
3216479720121227		17109455	19530515	1	20121227	7
32186984	20110228	15128440	19701112	2	20110228	7
32205677	20111002	15756488	19680320	1	20111002	8
32221811	20121001	16835972	19961118	2	20121001	7
32241386	20110924	15737916	19410129	1	20110924	8
32245800	20110923	15735833	19461210	1	20110923	10
32249620	20110216	15094965	19291010	1	20110216	7
32249653	20110526	15388131	19220814	1	20110526	8
32265659	20120819	16711702	19291016	1	20120819	7
32267086	20120404	16299744	19410813	2	20120404	10
32271151	20110124	15037553	19411016	1	20110124	9
32281213	20120408	16310899	19600727	1	20120408	10
32296836	20110112	15005727	19651129	1	20110112	10

32308331	20110621	15463109	19470420	1	20110621	7
32310422	20110831	15666732	19581029	2	20110831	8
32310535	20130422	17456322	19631118	1	20130422	8
32322591	20111018	15808153	19360301	1	20111018	8
32327143	20120609	16499393	19621220	2	20120609	8
32337943	20131027	18045851	19530625	1	20131027	7
32385378	20110411	15254647	19531226	1	20110411	8
32418170	20120924	16816994	19471022	1	20120924	7
32463255	20110720	15550038	19350223	1	20110720	8
32477955	20110429	15307119	19341202	2	20110429	7
32485782	20130508	17507994	19570810	1	20130508	7
32491784	20110603	15412298	19650611	1	20110603	8
32493735	20120806	16668485	19710808	1	20120806	7
32493804	20120820	16714293	19480702	2	20120820	7
32500973	20111018	15808522	19470115	2	20111018	8
32556084	20130818	17829111	19431202	2	20130818	7
32576413	20110718	15540657	19601201	1	20110718	8
32579434	20110225	15124280	19281026	1	20110225	10
32594846	20110613	15438222	19330717	2	20110613	7
32626656	20130725	17751567	19290801	1	20130725	8
32641557	20110921	15728902	19680517	2	20110921	8
32649824	20131118	18121290	19330811	1	20131118	7
32697317	20110227	15126133	19520108	1	20110227	7
32709470	20110811	15616841	19650922	2	20110811	8
32731796	20130523	17554431	19601203	1	20130523	8
32740662	20131118	18121174	19410808	2	20131118	8
32764560	20131008	17992016	19480212	2	20131008	7
32776491	20130518	17540542	19420511	1	20130518	7
32796319	20111210	15966078	19540605	2	20111210	7
32797481	20111207	15957155	19510302	1	20111207	7
32808438	20110409	15249372	19490105	2	20110409	8
32811180	20121005	16855391	19640818	2	20121005	8
32817768	20110925	15738451	19730417	1	20110925	8
32818498	20110405	15233364	19651207	1	20110405	10
32821208	20130131	17212237	19591210	2	20130131	8
32833322	20110107	14991398	19311025	1	20110107	8
32840178	20131006	17982078	19620320	1	20131006	9
32850070	20120817	16709652	19370220	1	20120817	8
32857946	20120303	16203499	19780805	2	20120303	7
32858938	20131022	18033505	19611031	1	20131022	7
32860110	20120613	16510213	19680904	1	20120613	7
32866914	20110521	15375089	19410916	1	20110521	7
32911754	20131221	18227837	19450528	1	20131221	8
32912699	20130818	17829023	19610719	1	20130818	7
32912780	20120531	16466022	19490901	2	20120531	8
32916419	20110507	15334662	19540607	2	20110507	10
32931218	20121212	17066892	19650227	1	20121212	7
32934682	20110219	15105893	19500311	2	20110219	10
32941278	20110625	15472392	19760101	2	20110625	8
32950928	20110215	15093092	19390908	2	20110215	8
32954588	20131120	18128272	19640807	1	20131120	8
32973141	20120316	16247742	19710629	1	20120316	10
32979821	20110112	15005937	19691224	1	20110112	10
32982620	20110329	15215523	19520229	2	20110329	9
32989610	20110523	15377783	19391001	1	20110523	8

32992624	20110919	15719528	19570515	2	20110919	8
32997981	20120905	16761496	19560217	1	20120905	10
33000952	20110503	15318770	19540321	2	20110503	8
33022478	20110117	15015713	19510212	1	20110117	8
33022683	20121017	16889948	19320213	1	20121017	7
33039440	20120722	16626952	19910917	2	20120722	8
3303952020130306		17311154	19321019	1	20130306	8
33049239	20111024	15823215	19440716	2	20111024	8
33064390	20110927	15743674	19250215	1	20110927	8
33096105	20110414	15265978	19620212	1	20110414	8
3309746020130522		17550155	19401204	1	20130522	8
3310134720121226		17106808	19500929	1	20121226	7
33113790	20121122	17001043	19550509	2	20121122	8
3312876620130821		17837773	19510427	1	20130821	7
33130006	20110415	15270672	19471231	2	20110415	10
33135681	20110805	15598732	20040507	2	20110805	7
33135885	20120711	16596490	19270113	1	20120711	7
33137110	20121026	16913986	19510119	1	20121026	8
33152431	20110623	15468655	19480604	1	20110623	8
33164088	20120703	16568220	19351215	2	20120703	7
33188191	20110525	15385253	19631111	1	20110525	10
33205559	20120512	16417134	19381206	1	20120512	8
33219839	20101215	14928287	19340612	1	20101215	10
33226765	20111020	15812443	19230807	1	20111020	7
33238061	20111012	15789693	19450726	1	20111012	7
33242454	20111201	15935620	19690430	2	20111201	7
3326125520130508		17506714	19531018	1	20130508	8
3326999720131103		18066633	19490120	1	20131103	9
33275068	20110523	15378529	19460701	2	20110523	7
3328964420130311		17328707	19490410	2	20130311	8
33292056	20110308	15156268	19901217	1	20110308	8
33294994	20120331	16284471	19470626	2	20120331	8
33330324	20101229	14964233	19681226	1	20101229	9
33367861	20110307	15153208	19640522	1	20110307	10
33371914	20111117	15897911	19510922	1	20111117	7
33377489	20120828	16735536	19430513	1	20120828	8
33385465	20110716	15538774	19941112	1	20110716	9
33391536	20111115	15889504	19601215	1	20111115	8
3339428420121213		17069329	19540128	1	20121213	8
33403600	20120823	16724646	19491018	1	20120823	10
33411095	20110208	15067633	19441122	1	20110208	8
33420723	20120821	16718911	19520201	2	20120821	8
33426812	20110731	15575125	19280415	1	20110731	8
3342736020130112		17159165	19661207	2	20130112	7
33431195	20111113	15883249	19391018	1	20111113	8
33431399	20110325	15208140	19551216	1	20110325	9
33433033	20120510	16410425	19370719	1	20120510	8
3344964820130817		17828671	19360401	1	20130817	9
33462145	20120527	16454749	19480507	2	20120527	8
3346558620130604		17588453	19480601	2	20130604	8
33468245	20110320	15192503	19511225	1	20110320	10
33471759	20120626	16545507	19660122	1	20120626	10
33472398	20111226	16008878	19600726	1	20111226	8
3347567120130721		17738250	19360814	1	20130721	7
33481833	20120531	16466258	19360712	1	20120531	10

33487795	20110119	15024674	19441221	2	20110119	9
33503514	20110406	15238822	19460803	1	20110406	7
33503569	20120212	16143212	19690121	1	20120212	8
33506604	20110220	15106026	20040614	1	20110220	10
3350845120130315		17343452	19520124	1	20130315	8
33514362	20110624	15470479	19650430	1	20110624	9
3351806820130507		17503957	19251221	1	20130507	8
33525927	20120906	16766044	19561125	1	20120906	10
33525950	20110208	15067495	19580707	2	20110208	9
33526088	20101213	14920440	19380320	1	20101213	9
33532557	20110208	15065618	19570903	2	20110208	8
33532604	20110123	15033802	19741217	2	20110123	10
33532739	20111123	15912574	19250416	2	20111123	10
33555872	20120617	16520850	19490505	1	20120617	9
3357302320130827		17853805	19330520	1	20130827	9
3357758120131201		18156667	19490418	1	20131201	8
33592313	20110322	15199191	19610504	2	20110322	7
3360247020130708		17696780	19420211	1	20130708	7
3361819820130716		17723137	19291030	2	20130716	7
33620494	20110129	15049723	19431006	1	20110129	10
3364125720130408		17412202	19461209	1	20130408	8
3365014520130702		17675851	19310217	2	20130702	8
33651808	20111021	15818241	19450908	1	20111021	8
33682370	20121006	16857777	19370211	2	20121006	7
3368818520130517		17538823	19350928	1	20130517	9
33706853	20110411	15253984	19560901	1	20110411	8
33712082	20121104	16938772	19420614	1	20121104	7
33715127	20120811	16690040	19570128	2	20120811	7
33718660	20110121	15030985	19300406	1	20110121	8
33743009	20110314	15176004	19230820	2	20110314	7
33767532	20120518	16434326	19571022	1	20120518	8
33777956	20120624	16537967	19451213	2	20120624	8
33780766	20110612	15434590	19580308	1	20110612	7
33784246	20121023	16905326	19610318	2	20121023	7
33794477	20110619	15455597	19361120	1	20110619	10
33795436	20110121	15031952	19340528	2	20110121	7
33797976	20111011	15785110	19570902	1	20111011	7
33830023	20110305	15148998	20010128	1	20110305	9
33830943	20111113	15883251	19580715	2	20111113	8
33840378	20121015	16879528	19390220	1	20121015	7
33841724	20110113	15007464	19331030	1	20110113	10
33846989	20111210	15965463	19711008	1	20111210	7
33853939	20110420	15284363	19491006	1	20110420	7
33868370	20110412	15259069	19640906	1	20110412	10
33868983	20110404	15230792	19360319	1	20110404	8
33904926	20110731	15575059	19350816	1	20110731	8
33912913	20110304	15145019	19310508	1	20110304	8
33920966	20110920	15726880	19621119	1	20110920	8
33926146	20121024	16907309	19430820	1	20121024	8
33952384	20120322	16263221	19371214	1	20120322	10
33981554	20110605	15414447	19650923	2	20110605	10
33983674	20110705	15503127	19340606	2	20110705	10
3400374220131221		18227693	19400612	2	20131221	7
3403611820130917		17924408	19520622	1	20130917	8
34047660	20110201	15057341	19621016	2	20110201	9

34056990	20130722	17742030	19411010	1	20130722	9
34071926	20110417	15272867	19430608	1	20110417	7
34072963	20120806	16669313	19921208	1	20120806	8
34084203	20130513	17523716	19341223	2	20130513	7
34088578	20111121	15906757	19321020	1	20111121	10
34097784	20110411	15254777	19221201	2	20110411	8
34128466	20110531	15397494	19760221	2	20110531	7
34128502	20110726	15562952	19541104	2	20110726	10
34130444	20110418	15275608	19751201	1	20110418	7
34131118	20110616	15449554	19260709	2	20110616	7
34147563	20110730	15574246	19500302	1	20110730	7
34150704	20110421	15287654	19440411	1	20110421	10
34164722	20131220	18225896	19720103	2	20131220	8
34168406	20110407	15242707	19610311	2	20110407	10
34170962	20111106	15862602	19600229	1	20111106	8
34189123	20120720	16623996	19460606	1	20120720	8
34189827	20120525	16452289	19720519	1	20120525	8
34200389	20110321	15194913	19481229	2	20110321	8
34202363	20110519	15370270	19630620	1	20110519	8
34210258	20110410	15250025	19560315	2	20110410	7
34216825	20120625	16539808	19351208	1	20120625	10
34220843	20130613	17618869	19300703	1	20130613	9
34220912	20110403	15228683	19640114	1	20110403	7
34223944	20110623	15469673	19291012	1	20110623	7
34224356	20120312	16233830	19451001	1	20120312	8
34239015	20111125	15919409	19541102	1	20111125	9
34248936	20110113	15007706	19451115	2	20110113	7
34260327	20130621	17643330	19570216	1	20130621	7
34270638	20130507	17503973	19421130	1	20130507	7
34280687	20130124	17192695	19221129	2	20130124	7
34283619	20120116	16071315	19970408	1	20120116	8
34284054	20111102	15850369	19591120	2	20111102	7
34290636	20120314	16240829	19610322	2	20120314	10
34296098	20110426	15299213	19480715	1	20110426	10
34296350	20130521	17547391	19680906	2	20130521	7
34299520	20120124	16089317	19590830	1	20120124	10
34305618	20110926	15741364	19760513	1	20110926	10
34310957	20130220	17262679	19820907	1	20130220	7
34335950	20110123	15033970	19910118	1	20110123	8
34342057	20110425	15295650	19551207	2	20110425	10
34347289	20110808	15604889	19371201	1	20110808	7
34354660	20111024	15822728	19671220	2	20111024	7
34356224	20130408	17412313	19310127	2	20130408	8
34363456	20111113	15883415	19180502	2	20111113	7
34383238	20110509	15336903	19370816	1	20110509	8
34385803	20120729	16645294	19601203	2	20120729	8
34395669	20101214	14924800	19951008	2	20101214	10
34399014	20120225	16180944	19400530	1	20120225	10
34411842	20130715	17720257	19470701	1	20130715	7
34415424	20110404	15231736	19581212	1	20110404	7
34421777	20110323	15202098	19310724	2	20110323	8
34423386	20111219	15989554	19480304	1	20111219	8
34435182	20110425	15294118	19240412	2	20110425	7
34444694	20110618	15455448	19611019	1	20110618	8
34451008	20130430	17478036	19731011	1	20130430	7

34451917	20110611	15434053	19320503	1	20110611	10
34453231	20131008	17987916	19281123	1	20131008	8
34466609	20120319	16252381	19420505	2	20120319	8
34477640	20110223	15117282	19970904	1	20110223	7
34485013	20110427	15302336	19501115	1	20110427	8
34488396	20110807	15600883	19640224	1	20110807	9
34524157	20120610	16500138	19600417	1	20120610	9
34536679	20110609	15428447	19300416	1	20110609	8
34548000	20110901	15672232	19690409	1	20110901	8
34563945	20130613	17619402	19580120	2	20130613	8
34570600	20111214	15977098	19431021	1	20111214	10
34575183	20110620	15459760	19490221	2	20110620	8
34586442	20120128	16094155	19510220	1	20120128	8
34604392	20131016	18016164	19600704	1	20131016	7
34629862	20111219	15990314	19810603	2	20111219	8
34633211	20110224	15120772	19650510	1	20110224	10
34646123	20110105	14982812	19690114	2	20110105	9
34649688	20111228	16014985	19600105	1	20111228	8
34656605	20110309	15162688	19510908	1	20110309	8
34669404	20110510	15342281	19500106	2	20110510	7
34689015	20110305	15148635	19461129	1	20110305	8
34710264	20130518	17540608	19390826	1	20130518	8
34716046	20130619	17638059	19440505	1	20130619	8
34747472	20110208	15066836	19780328	1	20110208	10
34759176	20130927	17951807	19771228	1	20130927	7
34759416	20120619	16527314	19510810	1	20120619	9
34767856	20111217	15986676	19510621	1	20111217	7
34773176	20130901	17865036	19980103	2	20130901	8
34774646	20120228	16187550	19510125	2	20120228	7
34774748	20121125	17006597	19490424	1	20121125	8
34792568	20121108	16953068	19270606	1	20121108	8
34822338	20110511	15346787	19261024	1	20110511	8
34825086	20130815	17823506	19460805	1	20130815	9
34831566	20130611	17611278	19651013	1	20130611	7
34834281	20110712	15526469	19350314	1	20110712	10
34846087	20131004	17978620	19431014	1	20131004	8
34854621	20120806	16669872	19520623	1	20120806	7
34862083	20110901	15670491	19430208	1	20110901	8
34876170	20110504	15324753	19270919	1	20110504	7
34916080	20121028	16917201	19560515	1	20121028	9
34928444	20130315	17343325	19540510	1	20130315	9
34936044	20110612	15434501	19681110	2	20110612	9
34948964	20110428	15305202	19420616	1	20110428	7
34950248	20120604	16478934	19510528	1	20120604	7
34965747	20110802	15584283	19660722	1	20110802	8
34982155	20110820	15640776	19631022	1	20110820	10
34990799	20110531	15397787	19340211	1	20110531	10
35007319	20120419	16346398	19490429	2	20120419	8
35010378	20110821	15641077	19400102	1	20110821	7
35040870	20110811	15615877	19590418	1	20110811	8
35043211	20130707	17693752	19710507	1	20130707	8
35057579	20101216	14931362	19531231	1	20101216	9
35078616	20110217	15099384	19480620	2	20110217	10
35079255	20110731	15575002	19560628	1	20110731	8
35091566	20110722	15554964	19700526	1	20110722	10

35092694	20110123	15033754	19600806	1	20110123	8
35101065	20111206	15952051	19491118	1	20111206	7
35111412	20110114	15012407	19330504	1	20110114	10
35115561	20110304	15146519	19960703	2	20110304	10
35119687	20110224	15120821	19560808	1	20110224	9
35121972	20120829	16738410	19411114	2	20120829	8
35122840	20110210	15073557	19540208	1	20110210	8
35123821	20110406	15238369	19591121	2	20110406	8
35159218	20110327	15210335	19540329	1	20110327	7
35162562	20110428	15304277	19530726	1	20110428	10
35170491	20110302	15137257	20050706	1	20110302	10
35173901	20110914	15710679	19350622	1	20110914	8
35176217	20110329	15216332	19370824	2	20110329	8
35176944	20111126	15921337	19260521	2	20111126	8
3517880420130324		17365983	19790531	2	20130324	8
3518107820131215		18208642	19391128	2	20131215	7
35183198	20120428	16367859	19230203	2	20120428	10
35184282	20120922	16813950	19610214	1	20120922	7
35210856	20101216	14931643	19771112	1	20101216	9
35213877	20110504	15324892	19520828	1	20110504	8
35218463	20111221	15997286	19290608	2	20111221	7
35225093	20101228	14959676	19551025	1	20101228	10
35238223	20110220	15106274	19460420	2	20110220	8
35245455	20110306	15150217	19600815	1	20110306	7
3526333320130105		17134435	19640718	1	20130105	7
3526726620130110		17153762	19251226	1	20130110	7
3526765320130617		17628556	19591201	2	20130617	7
35278854	20121015	16882369	19490215	1	20121015	7
35284367	20110317	15184745	19540117	1	20110317	7
35285031	20110501	15309243	19690903	1	20110501	8
35288132	20120220	16167878	19601012	2	20120220	8
35304235	20111012	15791438	19600722	2	20111012	8
35320968	20110605	15414253	19490216	1	20110605	8
35349463	20110307	15154674	19311130	2	20110307	10
3535351620130131		17212183	19520218	2	20130131	7
35361490	20110208	15066474	19561220	2	20110208	8
35362368	20120927	16826315	19421111	1	20120927	8
35363394	20110916	15715781	19300319	1	20110916	7
35370140	20120912	16785349	19730818	1	20120912	10
3537768520130519		17541094	19350824	1	20130519	7
35379636	20110421	15287096	19450114	1	20110421	7
35380428	20110207	15063587	19780824	1	20110207	8
35381216	20110115	15014141	19460714	2	20110115	8
35386244	20121204	17036670	19500917	1	20121204	8
35395096	20110222	15114021	19390409	1	20110222	10
35400227	20110627	15477193	19661213	2	20110627	7
3541516820130102		17120458	19621220	1	20130102	8
35418214	20110315	15180542	19331004	1	20110315	8
35442014	20120513	16417545	19491110	1	20120513	7
35453453	20110627	15477209	19640822	2	20110627	10
35461951	20120211	16142790	19450805	1	20120211	10
35475606	20111020	15815354	19540510	1	20111020	7
35488938	20110306	15150096	19651017	2	20110306	10
35496618	20111228	16015154	19341118	1	20111228	7
35521461	20111017	15804982	19441005	1	20111017	8

35527425	20110419	15279532	19430816	1	20110419	8
35536835	20110302	15138295	19770826	1	20110302	7
35538375	20110406	15238547	19530503	2	20110406	9
35539754	20110328	15212335	19660512	2	20110328	10
35546486	20120720	16624306	19660304	2	20120720	8
35546873	20101223	14950325	19370830	1	20101223	7
35547296	20120114	16067630	19490302	1	20120114	8
35560680	20120202	16112908	19610515	1	20120202	8
35561912	20110517	15362677	19521026	1	20110517	7
35565787	20110903	15678210	19530123	2	20110903	7
35570800	20101228	14960660	19520504	1	20101228	8
35572215	20110516	15360175	19751210	2	20110516	7
3559090020130530		17573141	19481006	2	20130530	8
3559659920130830		17861524	19610617	2	20130830	7
3559829920130527		17563984	19810802	1	20130527	7
35598584	20110115	15012790	19530328	2	20110115	10
35600114	20110901	15671748	19750420	2	20110901	7
35604207	20110515	15356685	19360102	1	20110515	10
35609202	20111031	15840229	19340112	2	20111031	8
35641351	20110916	15716927	19380415	1	20110916	8
3564622120130910		17902761	19290104	2	20130910	7
3564683420131111		18097857	19550220	1	20131111	7
35650023	20110207	15063310	19490901	1	20110207	7
3565844720130214		17246090	19701215	2	20130214	7
35679891	20110624	15472194	19460805	1	20110624	7
35693926	20110726	15564268	19870604	1	20110726	7
3570122520130910		17902426	19471028	2	20130910	8
35702308	20110122	15032649	19510429	2	20110122	9
35702546	20110207	15062170	19590217	2	20110207	8
3570293320130904		17882226	19671207	1	20130904	8
35706399	20111018	15807255	19410910	1	20111018	9
35719881	20110612	15434519	19681212	2	20110612	10
35720300	20120508	16403903	19701024	1	20120508	8
35724471	20110523	15378651	19610221	1	20110523	7
35726693	20120222	16173146	19620804	1	20120222	8
35729896	20110526	15388049	19600705	2	20110526	7
35732468	20110517	15362892	19490401	2	20110517	7
3573552520130812		17810821	19560226	1	20130812	8
35740013	20110930	15753490	19620325	1	20110930	7
3575072220130908		17893922	19300917	1	20130908	8
3575751820130723		17745784	19650930	1	20130723	8
3575987620130727		17755819	19611025	2	20130727	7
35760691	20110807	15600933	19760326	2	20110807	9
35766893	20111107	15866779	19440129	2	20111107	7
35772817	20111118	15899022	19610925	1	20111118	8
3577418620130506		17501010	19541216	2	20130506	7
35776955	20110517	15363575	19341201	2	20110517	10
35799452	20110828	15658904	19420822	1	20110828	7
35806292	20111013	15794554	19721113	2	20111013	8
35806510	20120806	16670222	19380705	2	20120806	10
35807239	20110822	15644673	19540116	1	20110822	7
35823484	20110918	15719055	19630629	1	20110918	8
3582911920121213		17069653	19311219	2	20121213	7
35843404	20110411	15254872	19650606	1	20110411	9
35843642	20110324	15205729	19520727	1	20110324	8

35853077	20110104	14979429	19511115	2	20110104	8
35853828	20110807	15600862	19370325	1	20110807	10
35856032	20110124	15036231	19861102	2	20110124	8
35861724	20110316	15183885	19450304	1	20110316	8
35862807	20111006	15775040	19740116	2	20111006	7
35863446	20131212	18202142	20030818	1	20131212	7
35886421	20131003	17974404	19370304	2	20131003	8
35896505	20110213	15084688	19510415	1	20110213	8
35916097	20130605	17593100	19341006	2	20130605	7
35926842	20110106	14988359	19841201	1	20110106	10
35931045	20120727	16642659	19400506	2	20120727	7
35931261	20110613	15434852	19560430	1	20110613	7
35935809	20110118	15019687	19290120	1	20110118	7
35939356	20110103	14973360	19301204	1	20110103	8
35940251	20110124	15037300	19640711	2	20110124	8
35946419	20130128	17201945	19610101	2	20130128	7
35951690	20111215	15981688	19311010	1	20111215	8
35951883	20121209	17053385	19630727	1	20121209	8
35955330	20110503	15318418	19701017	1	20110503	10
35958066	20120804	16663928	19341128	1	20120804	10
35959810	20121024	16908849	19471110	1	20121024	7
35963521	20110319	15190655	19450518	2	20110319	9
35966757	20110508	15335122	19580604	1	20110508	10
35982866	20110430	15308874	19681016	2	20110430	9
35989754	20110809	15608978	19301109	1	20110809	8
35989969	20110408	15246427	19320530	2	20110408	8
35990400	20110220	15106342	19310123	2	20110220	10
36001157	20111224	16005426	19791019	2	20111224	7
36013599	20110403	15228927	19570409	2	20110403	8
36015391	20121113	16970551	19630313	2	20121113	7
36060125	20130223	17273676	19200805	2	20130223	7
36060794	20101224	14952955	19880807	1	20101224	9
36068776	20120226	16182512	19590425	1	20120226	10
36069246	20110607	15418656	19360926	1	20110607	10
36069257	20101125	14865643	19581225	2	20101125	10
36070538	20110808	15604892	19521010	2	20110808	7
36071188	20130409	17415710	19660822	1	20130409	7
36072487	20120506	16395030	19580811	1	20120506	7
36072750	20110816	15629493	19960102	2	20110816	10
36073684	20110612	15434754	19590108	2	20110612	9
36088036	20120828	16735811	19841118	2	20120828	7
36092678	20130920	17931333	19410118	1	20130920	7
36117847	20121019	16896350	19540903	1	20121019	8
36122186	20120214	16150986	19660208	1	20120214	7
36126031	20110111	15002623	19610912	2	20110111	10
36129245	20120520	16437090	19970708	2	20120520	9
36131198	20110513	15354622	19300417	1	20110513	8
36141818	20130614	17622606	19421208	2	20130614	7
36149254	20120529	16459911	19350912	1	20120529	9
36158948	20110122	15032509	19411001	2	20110122	7
36164768	20120805	16664862	19440316	2	20120805	7
36167461	20110517	15363615	19610902	2	20110517	10
36171014	20110125	15039798	19341011	2	20110125	7
36171092	20120629	16552704	19300416	1	20120629	8
36186342	20110221	15107682	19640523	2	20110221	8

36187447	20110414	15267235	19341226	1	20110414	10
3619732720130814		17817351	19600205	2	20130814	7
36202514	20110623	15468946	19420313	1	20110623	10
36206367	20110628	15480094	19491112	2	20110628	7
36211344	20110430	15308686	19520124	2	20110430	8
3625299020130124		17194694	19880129	2	20130124	8
3625317520131002		17969169	19730831	2	20131002	7
36256914	20110104	14979687	19581002	2	20110104	10
36257473	20111215	15980958	20010718	1	20111215	10
36272363	20120501	16378757	19500801	1	20120501	9
36294549	20120202	16108978	19900101	1	20120202	7
36297695	20120218	16163875	19410519	1	20120218	10
36299066	20110217	15100665	19550818	1	20110217	7
36307332	20110920	15726786	19640722	1	20110920	8
36316311	20110107	14991862	19620610	1	20110107	7
36325947	20101220	14940402	19840305	1	20101220	10
36333150	20110311	15170325	19530612	1	20110311	10
36335929	20110426	15299259	19650817	1	20110426	7
36356464	20110608	15424316	19541214	1	20110608	10
3636229520130708		17698016	19990205	2	20130708	7
36370544	20111129	15928233	19450219	2	20111129	8
36376633	20110110	14997561	19590115	2	20110110	7
36383309	20110125	15040902	19670913	2	20110125	8
36391352	20120424	16358902	19580624	1	20120424	7
36396277	20110220	15106077	19350731	1	20110220	8
36397134	20110323	15202805	19380413	1	20110323	8
36400972	20110120	15028792	19710524	1	20110120	10
36405604	20110507	15334677	19770712	2	20110507	10
36406890	20111021	15817442	19430307	1	20111021	8
36420812	20120302	16201236	19550102	2	20120302	10
3642160020130629		17664634	19660102	1	20130629	9
36424325	20110326	15209931	19560511	1	20110326	9
36424687	20110312	15171922	19510420	1	20110312	8
3642760820131101		18063830	19610821	2	20131101	7
36429273	20110610	15432053	19560913	1	20110610	8
36449475	20110812	15620123	19600501	2	20110812	7
36453404	20111101	15844741	19591118	2	20111101	10
36455740	20110502	15314436	19500825	1	20110502	7
36460283	20110225	15123653	19730929	2	20110225	10
36473322	20110128	15049488	19501025	2	20110128	9
36500251	20120416	16336769	19560423	1	20120416	7
36517290	20110319	15192002	19861114	1	20110319	7
3652774920131013		18003079	19190103	1	20131013	7
36530413	20110617	15453100	19320621	2	20110617	8
36530980	20111105	15862004	19280802	1	20111105	8
36541034	20110727	15567669	19371030	2	20110727	7
36542639	20110716	15538648	19500704	2	20110716	10
36543198	20110126	15043859	19520422	1	20110126	7
36556408	20110104	14979668	19770814	2	20110104	10
36563301	20110628	15480272	19481129	1	20110628	10
36581336	20110825	15653678	19610522	1	20110825	10
36585418	20110727	15566996	19610516	2	20110727	8
36586182	20110106	14988351	19531016	1	20110106	9
3659722520130409		17416961	19490125	1	20130409	7
36598660	20110215	15092919	19701216	2	20110215	7

36609139	20110516	15360224	19630624	1	20110516	9
36614241	20110902	15674622	19621104	1	20110902	10
36615200	20120603	16474732	19770509	2	20120603	8
36617353	20120404	16298318	19310311	1	20120404	7
36637635	20110225	15123822	19540623	1	20110225	7
36638763	20110327	15210204	19470331	1	20110327	10
36642792	20110514	15356157	19490622	2	20110514	8
36654350	20110822	15644094	19430414	1	20110822	9
36656674	20130608	17604859	19441104	2	20130608	8
36659184	20110319	15191800	19350928	1	20110319	8
36659424	20131016	18015255	19550120	2	20131016	7
36660738	20120324	16267864	19390113	2	20120324	10
36669951	20120924	16817267	19580214	1	20120924	8
36675657	20110721	15552147	19560105	2	20110721	7
36680576	20110325	15208548	19441108	2	20110325	9
36692689	20120227	16184522	19230522	1	20120227	7
36701129	20110309	15162121	19330329	2	20110309	7
36701889	20110226	15125752	19360207	1	20110226	10
36712557	20101119	14851546	19540713	1	20101119	10
36725878	20121108	16955222	19410408	1	20121108	8
36726860	20110224	15119994	19520824	2	20110224	8
36743267	20110304	15147891	19871009	2	20110304	7
36753885	20110221	15109516	19690621	1	20110221	7
36767381	20110216	15097200	19400123	1	20110216	7
36770920	20110215	15093300	19390125	1	20110215	9
36771456	20101208	14905428	19480726	2	20101208	9
36776268	20110408	15247061	20000607	1	20110408	10
36782248	20121030	16922856	19301223	1	20121030	7
36784448	20110626	15473668	19431111	2	20110626	8
36785645	20110217	15100195	19671120	2	20110217	7
36789181	20120205	16119998	19491011	1	20120205	8
36791738	20120216	16158943	19650105	2	20120216	7
36791976	20110321	15194918	19400911	1	20110321	7
36794088	20121004	16851762	19690310	2	20121004	7
36802730	20110112	15005882	19830811	2	20110112	10
36805659	20110214	15084841	19600529	2	20110214	9
36808330	20110107	14991350	19710109	2	20110107	8
36809059	20120509	16408321	19281208	1	20120509	8
36815040	20110304	15147876	19510307	2	20110304	8
36820072	20101231	14968699	19590609	1	20101231	10
36821699	20120402	16290626	19660520	1	20120402	7
36833520	20110213	15084548	19541230	2	20110213	7
36856958	20110511	15347821	19680526	2	20110511	7
36866770	20101211	14915791	19590316	1	20101211	8
36880474	20110525	15385237	19640310	1	20110525	7
36887635	20110308	15158293	19551022	1	20110308	9
36889664	20120615	16517861	19411231	1	20120615	10
36891788	20130906	17891388	19490904	1	20130906	7
36897082	20110315	15179818	19370704	2	20110315	8
36898267	20130422	17454764	19710218	1	20130422	7
36899533	20111110	15875367	19390908	1	20111110	10
36903863	20130127	17200255	19420526	1	20130127	8
36904457	20101112	14831587	19620712	1	20101112	10
36907832	20110509	15339840	19730802	2	20110509	9
36912751	20120206	16124288	19701029	1	20120206	7

3691355020130610		17609093	19550815	1	20130610	7
36931461	20110105	14984213	19521105	2	20110105	8
36939294	20110504	15322912	19510320	2	20110504	7
36948875	20110206	15059841	19690630	2	20110206	9
36951674	20111216	15984935	19620811	1	20111216	8
36952768	20120725	16636991	19560730	2	20120725	7
36955416	20110111	15002203	19401218	1	20110111	9
36966491	20110427	15302248	19520910	1	20110427	10
36970260	20111205	15947638	19550516	1	20111205	8
36975005	20110208	15065273	19490712	2	20110208	8
36978855	20110102	14970065	19570620	1	20110102	10
36986035	20120320	16255942	19650327	1	20120320	10
36988019	20120625	16541466	20000722	1	20120625	7
36989590	20120423	16353275	19570213	1	20120423	10
36996288	20110328	15213381	19570325	1	20110328	7
36998217	20110304	15146967	19540924	2	20110304	7
37002770	20110306	15150080	19390505	2	20110306	7
37007640	20101206	14898722	19510111	1	20101206	10
37012605	20110507	15333637	20041016	2	20110507	10
37013788	20110113	15009379	19661010	2	20110113	7
37013868	20110912	15702576	19510114	2	20110912	8
37018147	20110102	14970006	19450216	1	20110102	10
3702195720130202		17218118	19660802	2	20130202	8
37023306	20120726	16640575	19271101	1	20120726	7
37030276	20110214	15088067	19660702	2	20110214	9
37033424	20110305	15149616	19470206	1	20110305	10
37033902	20110623	15468858	19430101	1	20110623	8
37034927	20110606	15415077	19350619	1	20110606	10
3703551120130609		17605398	19740620	2	20130609	7
37041422	20120530	16462800	19681006	2	20120530	7
37060030	20121216	17077503	19450406	1	20121216	8
37062649	20111117	15897453	19661118	2	20111117	8
37064452	20120301	16196453	19480520	1	20120301	8
3706536420130908		17893752	19551206	1	20130908	7
37066390	20120907	16770232	19530524	1	20120907	7
37074354	20110426	15298446	19500401	1	20110426	7
37076703	20110121	15031577	19520515	2	20110121	10
37079473	20110205	15059596	19751222	1	20110205	7
37085931	20120327	16274600	19450516	1	20120327	10
37086183	20110614	15443192	19680823	2	20110614	8
37094125	20110219	15105855	19690529	1	20110219	10
37097055	20110305	15149591	19371005	1	20110305	9
37108739	20101211	14915900	19491012	2	20101211	8
37111152	20120401	16285479	19440615	1	20120401	8
37122615	20110313	15172525	19870709	1	20110313	10
37124008	20110407	15238905	19500518	2	20110407	10
37138059	20120204	16119157	19441014	1	20120204	7
37147118	20120220	16166903	19630710	2	20120220	7
37158397	20110522	15375724	19331215	1	20110522	10
3715978720130615		17625153	19580719	1	20130615	7
37163852	20110217	15100640	19380324	1	20110217	8
37165370	20110125	15040591	19400615	1	20110125	8
37172886	20110111	15002202	19720925	2	20110111	10
37180099	20110626	15473815	19571015	1	20110626	10
37187556	20110812	15617971	19261114	2	20110812	8

37191427	20130706	17693167	19590712	1	20130706	7
37197710	20110214	15088493	19600602	2	20110214	9
37198417	20110222	15113992	19621123	1	20110222	10
37199125	20110115	15014229	19461010	1	20110115	7
37199976	20111117	15898217	19340330	1	20111117	8
37201619	20110421	15287356	19481019	2	20110421	7
37220885	20110117	15018379	19630615	1	20110117	9
37225062	20110411	15254974	19580610	1	20110411	10
37235737	20121220	17090644	19421025	1	20121220	10
37236058	20110227	15126134	19640825	1	20110227	8
37237028	20111028	15835928	19360608	1	20111028	10
37241626	20110903	15678168	19330312	1	20110903	7
37253455	20110728	15570642	19541104	1	20110728	8
37256487	20120625	16541401	19461020	1	20120625	10
37263915	20120908	16772626	19681225	2	20120908	10
37264258	20101213	14920142	19571221	1	20101213	10
37265706	20121113	16971866	19901002	2	20121113	9
37268954	20110123	15033952	19670331	2	20110123	8
37271106	20110202	15057496	19901018	1	20110202	10
37271184	20110308	15157795	19341219	1	20110308	7
37273362	20120912	16784615	19440701	2	20120912	7
37273384	20110722	15555775	19520915	1	20110722	10
37277671	20110414	15267564	20021007	1	20110414	7
37277773	20110126	15044133	19760602	2	20110126	8
37287233	20110613	15438223	19310820	1	20110613	8
37311981	20110502	15315295	19940206	1	20110502	9
37314457	20110320	15192331	19551020	1	20110320	10
37324202	20110502	15314830	19431029	1	20110502	8
37332391	20130306	17311330	19510210	1	20130306	8
37335958	20111027	15833372	19590812	2	20111027	7
37340479	20110331	15220502	19540624	1	20110331	7
37349330	20130328	17379355	19621010	1	20130328	7
37350133	20110108	14993945	19711222	1	20110108	7
37351114	20131001	17963611	19530521	2	20131001	7
37357703	20110302	15139440	19461015	2	20110302	8
37358773	20110410	15249969	19500301	2	20110410	8
37359049	20110406	15235061	19660314	1	20110406	10
37365289	20110322	15198736	19900207	2	20110322	7
37366555	20110302	15138362	19521215	2	20110302	10
37370186	20110510	15343796	19660701	1	20110510	8
37374155	20110418	15275690	19600925	1	20110418	8
37374279	20110902	15676353	19481004	1	20110902	7
37374508	20120402	16290353	19691012	1	20120402	8
37381536	20110417	15272861	19380130	2	20110417	8
37391234	20110328	15213174	19490618	1	20110328	10
37399294	20111227	16012626	19530306	1	20111227	8
37403911	20110508	15335035	19670421	1	20110508	10
37407071	20110112	15004663	19681208	1	20110112	8
37407286	20111008	15781917	19421224	1	20111008	7
37416390	20110304	15146804	19560714	2	20110304	7
37420114	20110809	15608545	19430816	1	20110809	8
37423997	20101223	14950390	19580331	1	20101223	10
37434132	20111029	15837564	19640115	2	20111029	9
37436127	20110102	14970025	19640221	2	20110102	8
37439911	20110407	15242791	19551220	1	20110407	8

37453966	20110311	15169685	19751025	1	20110311	9
37454801	20111109	15874383	19310620	2	20111109	10
37455677	20120920	16808404	19711206	2	20120920	7
37459908	20110107	14990220	19650608	1	20110107	10
3746120420131027		18045892	19490417	2	20131027	9
37462218	20120703	16566037	19510223	1	20120703	7
37462923	20120515	16423641	19510220	1	20120515	7
37464292	20110127	15047130	19950805	1	20110127	7
37465002	20110328	15213576	19510615	2	20110328	10
37471117	20110420	15284295	19601215	2	20110420	8
37476269	20110225	15123440	19460728	1	20110225	7
37478903	20110208	15065486	19810509	2	20110208	8
37482307	20120508	16402345	19611015	1	20120508	7
37484267	20110301	15133330	19601116	1	20110301	10
37486070	20110401	15226933	19460202	1	20110401	10
37486547	20101230	14966115	19330505	2	20101230	7
37489046	20120130	16097902	19441128	1	20120130	7
37494410	20110116	15014737	19780206	1	20110116	10
37496461	20110920	15725616	19610313	1	20110920	8
37497839	20110718	15542981	19520302	1	20110718	8
37497920	20110503	15320100	19450905	2	20110503	10
37499233	20110822	15644582	19520816	2	20110822	7
37499799	20101107	14812993	19390726	1	20101107	10
37500019	20110213	15084707	19330718	2	20110213	10
37503994	20110622	15466202	19430816	2	20110622	10
37506302	20110216	15097265	19660715	1	20110216	7
37512235	20110329	15216342	19720118	1	20110329	10
3751444620130921		17933322	19491207	1	20130921	8
37525158	20120926	16821643	19430509	2	20120926	9
37529729	20121223	17097582	19510911	1	20121223	10
37534193	20120614	16515458	19510530	2	20120614	10
37535287	20110302	15139375	19591026	2	20110302	7
37539494	20101124	14863154	19630424	1	20101124	8
37549385	20110316	15183936	19270126	1	20110316	10
37549772	20110109	14994323	19660828	2	20110109	7
37550280	20110619	15455616	20081120	1	20110619	7
37554588	20110729	15572847	19641126	2	20110729	8
37560342	20110807	15600943	19531020	1	20110807	8
37561107	20110502	15314234	19621106	1	20110502	10
37562735	20110302	15137591	19500203	1	20110302	10
37566475	20110419	15279847	19510401	1	20110419	8
37567876	20110222	15113756	19960817	1	20110222	10
37568153	20110401	15226813	19730228	1	20110401	10
37571338	20110404	15229402	19480930	2	20110404	7
37575841	20110503	15319681	19521018	1	20110503	8
37580919	20121002	16841420	19500510	2	20121002	7
37582346	20110621	15463024	19540120	1	20110621	9
37582404	20110828	15658897	19500515	1	20110828	10
37584580	20120323	16265701	19731202	1	20120323	8
37588231	20120614	16514229	19540714	1	20120614	7
37591369	20110105	14984035	19551025	2	20110105	9
37596433	20120403	16297091	19360308	2	20120403	10
3759664820130410		17421112	19410323	1	20130410	9
37604027	20120316	16245557	19760828	2	20120316	9
37606012	20110222	15110473	20081022	2	20110222	10

3761388020130731		17767141	19340806	2	20130731	7
3761530820131114		18110862	19561006	2	20131114	8
37621026	20110207	15063485	19770117	1	20110207	8
37622154	20120209	16136877	19450220	1	20120209	8
37627831	20120801	16656529	19551122	2	20120801	10
37631166	20110424	15292324	19591210	1	20110424	7
37634596	20101225	14954381	19621220	1	20101225	10
37635168	20101207	14902789	19541125	1	20101207	7
37642890	20101225	14954097	19451119	2	20101225	10
37654403	20110323	15202216	19271212	1	20110323	7
37655508	20110221	15110377	19540302	1	20110221	10
37656272	20121004	16850400	19720502	1	20121004	8
37658121	20101226	14954802	19651030	2	20101226	10
37663608	20110117	15017673	19521215	1	20110117	9
37664667	20120831	16743761	19560827	2	20120831	8
37666050	20110616	15450459	19630615	1	20110616	7
37669468	20101220	14940130	19530427	1	20101220	8
37676236	20121115	16979392	19220507	1	20121115	8
37679122	20110419	15279341	19531001	1	20110419	10
37679826	20121210	17055657	19591231	2	20121210	8
37680878	20110308	15157991	19440809	2	20110308	10
37684507	20120515	16424885	19740519	1	20120515	10
3768451820130804		17780981	19640417	2	20130804	7
37685760	20110920	15726488	19630320	1	20110920	8
37688792	20111021	15817478	19361021	2	20111021	10
37696596	20110218	15101667	19561024	2	20110218	9
37697680	20110606	15414855	19650803	2	20110606	7
37698741	20110315	15179717	19471225	2	20110315	10
37699040	20110322	15198336	19821003	2	20110322	9
37702264	20110105	14984028	19350210	1	20110105	9
3770443120130123		17190954	19560822	1	20130123	8
3770474820130225		17277333	19680702	2	20130225	7
37706299	20110615	15446402	19480201	2	20110615	7
37710193	20120706	16580237	19541031	1	20120706	7
37714480	20110503	15318515	19480529	1	20110503	8
37715290	20120826	16729620	19560126	1	20120826	8
37716704	20120427	16367074	19970310	2	20120427	7
3772070020130817		17828663	19590407	2	20130817	7
37722784	20110928	15747984	19291214	1	20110928	7
37723572	20120805	16664908	19170921	2	20120805	7
37723663	20110121	15031659	19350307	1	20110121	8
37725169	20110411	15254081	19740930	2	20110411	10
37728000	20110110	14998015	19440211	1	20110110	7
37728237	20110116	15014420	19420901	1	20110116	9
37734831	20110913	15705312	19610609	1	20110913	7
37743423	20110712	15527159	19700202	1	20110712	7
37747356	20111007	15779831	19370419	2	20111007	7
37749476	20120528	16457172	19520110	1	20120528	7
3775467920131204		18171222	19400527	1	20131204	7
37761936	20110120	15028451	19621217	2	20110120	7
37762144	20110208	15066663	19400517	1	20110208	7
37763625	20110228	15128064	19810129	1	20110228	7
37766464	20111003	15759144	19540215	1	20111003	7
37767149	20101121	14853875	19711101	1	20101121	8
3776871120130708		17696425	19461201	1	20130708	7

37769190	20110105	14984284	19611023	1	20110105	7
37770517	20110811	15616508	19550305	1	20110811	8
37770788	20101120	14853288	19441008	2	20101120	10
37773129	20110106	14988129	19560607	2	20110106	8
37773630	20110621	15462623	19531210	1	20110621	8
37782971	20101114	14834883	19390501	1	20101114	8
37785856	20130225	17275990	19460725	1	20130225	7
37787318	20101229	14964046	19551205	1	20101229	10
37789223	20110808	15605045	19390816	2	20110808	7
37790399	20120330	16283524	19541005	1	20120330	10
37791256	20120416	16336576	19330606	2	20120416	10
37792680	20130705	17689838	19651025	2	20130705	9
37793241	20120709	16588790	19680801	2	20120709	9
37793592	20110405	15233735	19511027	2	20110405	8
37796137	20110103	14974657	19521020	1	20110103	9
37798804	20110105	14982960	19550710	1	20110105	8
37798859	20110104	14978019	19330118	1	20110104	7
37800207	20110116	15014748	19530615	1	20110116	8
37801131	20120520	16436949	19600215	2	20120520	7
37802452	20130806	17791339	19311216	1	20130806	7
37804152	20110207	15062425	19590401	1	20110207	8
37806783	20121029	16920091	19620905	2	20121029	7
37808494	20110106	14985018	19531113	1	20110106	7
37811260	20130922	17934030	19421026	2	20130922	8
37815024	20130418	17445591	19760809	1	20130418	8
37820034	20110127	15046436	19460926	2	20110127	7
37820954	20110219	15105813	19380927	1	20110219	8
37825766	20110816	15628386	19520315	1	20110816	8
37833913	20130423	17459181	19580417	1	20130423	7
37834029	20110227	15126023	19910130	2	20110227	7
37835099	20110103	14973800	19590319	1	20110103	8
37838123	20110108	14993830	19540812	1	20110108	7
37840123	20110818	15635278	19640102	2	20110818	10
37840350	20110223	15117554	19490325	1	20110223	8
37840930	20110503	15319791	19660105	2	20110503	8
37841206	20120911	16780876	19720214	2	20120911	8
37842425	20130930	17958133	19550319	2	20130930	7
37847306	20120524	16450027	19570220	2	20120524	8
37850285	20110912	15702210	19631213	1	20110912	8
37851175	20110326	15209803	19631226	1	20110326	7
37851426	20110105	14983717	19800627	1	20110105	9
37852872	20110109	14994521	19480803	1	20110109	8
37853251	20111207	15957199	19690205	2	20111207	7
37853557	20110103	14975012	19291215	2	20110103	8
37854889	20110208	15067380	19520828	1	20110208	8
37854992	20110316	15182894	19390912	1	20110316	7
37857582	20120328	16277240	19650207	2	20120328	8
37857617	20101027	14779784	19340330	2	20101027	7
37858187	20101231	14967946	19620423	1	20101231	10
37858201	20110101	14969270	19650715	1	20110101	10
37858289	20110103	14974862	19361023	1	20110103	9
37858530	20110127	15046910	19731102	1	20110127	7
37858972	20110221	15110273	19650523	2	20110221	8
37860665	20121121	16997733	19250625	2	20121121	7
37868669	20130723	17745808	19440806	2	20130723	9

37869797	20110123	15033963	19981102	1	20110123	7
3787256320130917		17925999	19450116	1	20130917	7
37872610	20110304	15146956	19620216	2	20110304	10
37879575	20110424	15292286	19300612	1	20110424	8
37881100	20101123	14860022	19721002	1	20101123	8
37887619	20111009	15782325	19390310	1	20111009	9
37891013	20110226	15124777	19290315	1	20110226	8
37891773	20110904	15678681	19520907	2	20110904	7
37892390	20110104	14978227	19590209	1	20110104	9
37898456	20111027	15833311	19260827	1	20111027	10
37899288	20110325	15207999	19470916	1	20110325	8
37900928	20121220	17092559	19520728	1	20121220	8
37901761	20110408	15247265	19301130	2	20110408	10
37902888	20110207	15062226	19650914	1	20110207	10
37903836	20101201	14880290	19440815	2	20101201	7
37905638	20110214	15088195	19281113	1	20110214	9
37911903	20110223	15117324	19631020	1	20110223	10
37912531	20121105	16941907	19590225	2	20121105	8
37914424	20110216	15096236	19510604	1	20110216	8
37915472	20110223	15116755	19420205	1	20110223	10
37915632	20120611	16503925	19520730	2	20120611	8
37916384	20110218	15102193	19541128	1	20110218	8
37920062	20110729	15572602	19680705	1	20110729	8
37922513	20110425	15295306	19540722	2	20110425	7
37922853	20110116	15014711	19570722	1	20110116	10
37923732	20110226	15125747	19650318	1	20110226	10
37924939	20110116	15014493	19571031	1	20110116	8
37926684	20111016	15800920	19590520	1	20111016	7
37927734	20120301	16196300	19611120	2	20120301	7
37928691	20110407	15242218	19370112	1	20110407	8
3793248220130319		17352627	20071009	1	20130319	8
37933601	20110212	15084220	19500301	1	20110212	10
37934900	20110313	15172522	19610404	1	20110313	8
37940548	20110318	15190426	19370215	1	20110318	10
37941392	20110316	15184064	19450708	1	20110316	8
37941803	20110414	15266674	19730119	2	20110414	8
37942442	20110324	15205255	19750909	1	20110324	7
37943547	20110208	15066510	19551107	1	20110208	8
37943810	20110204	15058471	19810917	1	20110204	10
37943865	20110113	15008535	19620218	2	20110113	8
37945963	20110213	15084470	19650618	2	20110213	7
37946068	20110330	15217981	19620108	1	20110330	9
37946422	20110225	15124031	19261118	1	20110225	7
37947050	20101209	14910433	19570906	1	20101209	10
37950633	20101230	14965899	19460525	2	20101230	10
37951750	20110101	14969280	19490412	2	20110101	8
37953278	20110101	14969255	19710216	2	20110101	8
37954679	20110114	15012598	19660726	1	20110114	7
37957032	20120522	16443256	19591201	2	20120522	10
37965303	20101227	14957758	19440110	1	20101227	8
37972739	20101220	14939209	19330110	2	20101220	9
37973425	20101228	14961026	19520902	1	20101228	8
37977030	20110622	15466391	19560410	1	20110622	7
37979025	20110315	15180534	19570315	2	20110315	8
37979058	20110315	15180481	19461111	2	20110315	8

37980077	20130218	17254679	19570828	1	20130218	7
37980099	20120617	16520774	19540813	2	20120617	10
37984057	20110905	15683125	19760416	1	20110905	10
37989110	20110324	15204740	19610416	1	20110324	9
37990753	20110320	15192305	19780614	1	20110320	7
37990797	20110829	15660779	19350328	2	20110829	7
37991563	20110327	15210308	19820426	2	20110327	9
37991654	20110322	15198424	19551220	2	20110322	8
37993503	20110324	15205771	19580505	2	20110324	8
37995270	20101226	14954750	19620908	1	20101226	9
37998713	20110309	15162338	19530614	1	20110309	7
38001228	20110421	15286786	19680214	2	20110421	10
38002107	20120202	16112075	19590427	1	20120202	10
38005559	20110323	15202276	19771004	2	20110323	8
38005935	20110324	15203706	19931010	1	20110324	8
38006416	20110419	15280384	19520801	1	20110419	8
38007317	20110404	15230627	19671211	2	20110404	8
38007339	20110322	15199463	19500723	2	20110322	7
38008058	20110404	15229838	19610520	1	20110404	8
38008489	20110322	15197988	19681205	1	20110322	7
38008854	20110404	15231660	19411020	1	20110404	8
38009857	20130925	17944692	19710101	1	20130925	7
38012270	20110210	15078173	19770623	1	20110210	9
38013182	20110210	15075216	19810503	1	20110210	10
38015826	20130328	17379423	19420627	1	20130328	7
38018610	20101213	14920924	19850504	1	20101213	8
38019920	20110314	15176964	19290925	2	20110314	8
38020778	20110327	15210240	19270821	2	20110327	10
38022241	20110413	15260158	19580924	1	20110413	8
38022592	20110328	15213101	19530606	2	20110328	7
38022854	20110331	15221609	19400102	1	20110331	8
38024407	20110411	15253536	19541214	1	20110411	7
38024429	20110421	15286723	19420120	2	20110421	9
38024496	20110405	15233157	19630924	1	20110405	8
38025853	20101228	14961297	19300907	2	20101228	10
38028352	20120101	16022580	19560110	1	20120101	7
38031708	20101230	14965935	19570825	2	20101230	9
38031786	20101226	14954632	19510610	1	20101226	10
38032132	20120704	16572084	19460601	1	20120704	8
38032803	20110114	15011921	19640516	1	20110114	7
38034887	20101226	14954882	19541231	2	20101226	7
38035164	20101225	14954161	19470416	1	20101225	8
38035799	20110625	15473307	19380815	2	20110625	8
38038936	20110116	15014816	19310826	2	20110116	10
38040323	20111107	15865791	19671231	2	20111107	10
38041279	20110104	14979466	19541124	1	20110104	7
38044712	20120627	16548659	19530326	1	20120627	9
38045566	20110329	15216126	19531023	2	20110329	7
38048429	20110105	14983105	19510110	1	20110105	8
38049568	20110125	15040494	19430218	2	20110125	10
38050656	20131123	18136442	19770529	1	20131123	7
38051897	20110302	15139502	19521205	2	20110302	10
38052754	20110218	15101600	19370909	1	20110218	7
38053655	20110308	15158320	19510202	1	20110308	10
38053666	20110227	15126090	19600126	2	20110227	8

38054385	20110303	15143273	19690217	1	20110303	7
38055253	20110223	15116891	19840303	1	20110223	7
38056450	20110308	15159167	19760731	1	20110308	9
38058718	20110322	15199492	19421027	2	20110322	9
38062327	20110228	15128204	19240223	1	20110228	7
38063557	20110116	15014686	19590917	1	20110116	10
38063671	20110126	15041875	19580415	1	20110126	8
38066523	20110115	15014195	19630825	2	20110115	9
38066658	20110207	15062068	19741019	2	20110207	8
38066761	20110115	15014154	19490901	1	20110115	8
38067355	20110208	15068204	20021106	2	20110208	8
38071384	20120318	16250740	19731001	1	20120318	10
38071500	20110117	15018479	19340905	1	20110117	10
38071942	20110120	15028184	19610422	2	20110120	9
38072047	20110309	15162924	19981015	2	20110309	7
38072058	20110320	15192270	19340731	1	20110320	7
38072901	20111215	15981037	19450115	1	20111215	7
38073051	20110114	15011889	19260609	1	20110114	9
38075922	20110214	15088306	19670311	1	20110214	7
38078830	20110208	15067515	19620119	2	20110208	7
38078885	20110204	15058705	19630422	1	20110204	10
38080034	20110414	15267484	19680924	2	20110414	10
38081628	20110209	15072226	19530320	2	20110209	8
38082187	20110214	15087920	19500103	2	20110214	8
38082676	20110207	15061933	19271116	2	20110207	8
38084832	20110127	15047103	19381120	2	20110127	9
38086703	20110127	15044839	19820418	2	20110127	8
38087104	20110222	15111356	19431006	2	20110222	8
38087875	20110215	15093292	19410824	2	20110215	8
38088652	20110210	15074372	19691218	1	20110210	10
38089713	20110221	15109666	19561002	1	20110221	7
38091906	20110119	15025443	19410202	2	20110119	10
38093720	20101208	14906667	19301214	2	20101208	7
38093888	20110103	14974950	19821004	2	20110103	7
38097084	20111013	15794767	19260707	1	20111013	8
38110031	20110208	15068069	19600724	2	20110208	8
38110097	20110802	15585190	19630723	2	20110802	7
38110417	20110209	15072205	19440702	1	20110209	7
38113438	20110301	15132381	19600525	1	20110301	8
38113881	20110210	15076692	19621227	2	20110210	8
38114691	20110303	15140430	19401127	1	20110303	8
38115387	20110217	15099556	19630716	1	20110217	7
38116153	20110211	15081305	19520402	1	20110211	8
38117861	20110209	15071625	19310805	1	20110209	7
38123352	20110416	15272109	19410423	1	20110416	8
38125632	20110216	15095124	19581001	2	20110216	10
38127365	20110225	15122644	19421210	2	20110225	7
38127774	20110328	15211273	19490925	2	20110328	8
38127898	20110615	15445527	19550528	2	20110615	8
38127912	20110329	15215455	19351208	1	20110329	10
38128084	20110215	15093118	19710429	1	20110215	8
38128608	20110306	15149955	19970326	2	20110306	7
38129190	20120421	16351389	19650928	1	20120421	10
38129894	20110228	15128296	19590420	1	20110228	7
38130255	20110218	15102785	19390508	1	20110218	10

38130288	20110224	15121233	19590208	1	20110224	8
38131816	20110318	15188965	19970529	1	20110318	10
38131850	20110320	15192205	19660326	1	20110320	7
38132320	20110502	15313838	19421209	1	20110502	10
38132397	20110506	15329949	19430430	2	20110506	8
38132693	20110514	15356151	19500123	1	20110514	7
38133481	20110407	15240752	19320720	1	20110407	8
38133765	20110417	15272688	19360108	1	20110417	8
38133936	20110501	15309244	19400814	1	20110501	9
38134257	20110619	15455917	19681212	1	20110619	7
38134780	20110608	15421063	19850225	2	20110608	8
38136673	20110531	15397663	19540628	1	20110531	8
38137085	20110425	15296041	19560420	1	20110425	8
38137869	20110306	15150215	19311210	2	20110306	9
38137961	20110302	15137960	19510711	1	20110302	10
38142766	20110317	15185309	19530109	2	20110317	9
3814819520130324		17365961	19390411	1	20130324	8
38148628	20110314	15174672	19510515	1	20110314	9
38151201	20110209	15072476	19630211	1	20110209	8
38156160	20110309	15163071	19380414	1	20110309	8
38157765	20110910	15701474	19491205	1	20110910	7
38158246	20110124	15036757	19440709	1	20110124	10
38158451	20110318	15188079	19620813	2	20110318	10
38158600	20120226	16182845	19461111	2	20120226	8
38160860	20110302	15137913	19501010	2	20110302	8
38162399	20110411	15254305	19650906	1	20110411	7
38163314	20110418	15275144	19551030	1	20110418	8
38165718	20110511	15346842	19390622	1	20110511	8
38166200	20110320	15192389	19520423	1	20110320	9
38166222	20110323	15202456	19681217	1	20110323	8
38168693	20110419	15280633	19680508	1	20110419	9
38168831	20110506	15332751	19591115	2	20110506	7
38169652	20110511	15346729	19611127	1	20110511	8
38170580	20110304	15146175	19491227	1	20110304	8
38171016	20110406	15237035	19510412	1	20110406	10
38171072	20110315	15180474	19360615	2	20110315	9
38171094	20110320	15192448	19530828	1	20110320	9
38171721	20110322	15199335	19470817	1	20110322	10
38173330	20120130	16097663	19321130	1	20120130	10
38175121	20110306	15150033	19331201	1	20110306	8
38175187	20110307	15154797	19441226	1	20110307	8
38177310	20110321	15194756	19250331	2	20110321	10
38177694	20110405	15233032	19740828	2	20110405	9
38177741	20110329	15215031	19660519	1	20110329	10
38178131	20110509	15338270	19411204	2	20110509	8
38182557	20110414	15266569	19690628	2	20110414	8
38187369	20110317	15186983	19540117	2	20110317	7
38187427	20110612	15434820	19630518	2	20110612	8
38187767	20110310	15165766	19530828	1	20110310	9
38188691	20110314	15175979	19240527	1	20110314	7
38188997	20110314	15175685	19421115	2	20110314	7
38189343	20110320	15192357	19920515	1	20110320	8
38190113	20110315	15179614	19370618	1	20110315	9
38190680	20110410	15250043	19341016	1	20110410	7
38194900	20110406	15236608	19710105	1	20110406	10

38195152	20110407	15242195	19780814	1	20110407	8
38196177	20110406	15238245	19490517	1	20110406	10
38196315	20110415	15270066	19610317	1	20110415	8
38197090	20110626	15473913	19770726	2	20110626	10
38197818	20110403	15228877	19650110	2	20110403	7
38197910	20110404	15230927	19420514	2	20110404	7
38201273	20110202	15057645	19420612	2	20110202	8
38204067	20110301	15132464	19671028	1	20110301	10
38204136	20110206	15060127	19370905	2	20110206	10
38205139	20110416	15272094	19410801	1	20110416	8
3820741920130625		17654405	19690107	1	20130625	8
38208207	20110417	15272663	19330120	2	20110417	10
38210332	20110507	15334273	19610831	1	20110507	8
38210785	20110408	15245720	19600719	1	20110408	10
38211459	20110503	15319938	19680117	1	20110503	8
38211631	20110406	15236909	19401123	2	20110406	10
38212510	20110406	15236117	19300416	1	20110406	8
38212963	20110902	15675097	19240919	2	20110902	8
38213524	20110424	15292300	19490124	2	20110424	8
38213819	20110518	15366368	19460105	1	20110518	8
38214027	20110421	15287510	19540609	1	20110421	8
38215702	20110408	15246604	19580505	1	20110408	10
38216125	20110422	15288818	19281228	1	20110422	8
38219420	20110501	15309330	19760715	2	20110501	10
38219497	20110514	15356031	19660109	1	20110514	8
38219908	20110501	15309195	19590413	1	20110501	10
38221726	20110606	15415035	19570405	2	20110606	9
38223335	20110415	15270140	19480821	1	20110415	9
38223562	20110421	15287540	19420507	2	20110421	8
38223573	20110818	15635382	19410920	2	20110818	7
38232074	20110417	15272890	19670616	2	20110417	7
38232756	20110828	15659080	19611115	2	20110828	7
38233215	20120806	16670100	19381030	1	20120806	8
38233282	20110419	15280544	19420104	1	20110419	8
38238072	20110422	15289860	19820408	1	20110422	9
38241291	20110502	15313525	19390712	1	20110502	8
38242670	20110421	15286582	19480623	2	20110421	8
38243208	20110425	15295316	19700813	2	20110425	7
38245102	20110517	15363274	19641205	2	20110517	7
38245191	20110612	15434581	19510911	2	20110612	8
38246536	20110509	15338212	19650919	1	20110509	10
38247915	20110504	15323903	19690614	1	20110504	10
38250189	20110321	15196176	19570825	1	20110321	8
38250203	20110209	15073092	19300218	1	20110209	10
38250292	20110302	15138915	19420121	1	20110302	7
38250736	20110209	15073405	19611215	1	20110209	7
38250769	20110209	15073468	19640301	1	20110209	8
38252163	20110214	15088804	19481123	2	20110214	8
38252389	20110217	15100203	19680910	1	20110217	10
3825239020130417		17442627	19701223	1	20130417	8
38252403	20110315	15180447	20090901	1	20110315	10
38252630	20110216	15094926	19680502	1	20110216	10
38254965	20110510	15341996	19520312	1	20110510	8
38256029	20110420	15283711	19570115	1	20110420	8
38257420	20110422	15289815	19600916	1	20110422	8

38258752	20110512	15350721	19420404	1	20110512	8
38259288	20110427	15301632	19670331	2	20110427	7
38260161	20111001	15756137	19300503	1	20111001	8
38260536	20110506	15332221	19561118	1	20110506	7
38260887	20110518	15366865	19400219	1	20110518	7
38264027	20110618	15455409	19391021	1	20110618	10
38265279	20110505	15326285	19691127	1	20110505	7
38266158	20120731	16651476	19580415	1	20120731	7
38266352	20110428	15304985	19791118	2	20110428	7
38267355	20110501	15309186	19560515	1	20110501	10
38267673	20120102	16027455	19920109	2	20120102	10
38268007	20110428	15304709	19441224	2	20110428	8
38270870	20110426	15298482	19701008	2	20110426	8
38271000	20110510	15341060	19400930	2	20110510	8
38271102	20110523	15378148	19620610	1	20110523	8
38272887	20110519	15368125	19451016	1	20110519	10
38273222	20110531	15397175	19700829	2	20110531	10
38273482	20110607	15419100	19620821	2	20110607	7
38274292	20110623	15469201	19780225	1	20110623	9
38275171	20110607	15418906	19650327	2	20110607	10
38277224	20110705	15502821	19620325	1	20110705	7
38280523	20110511	15347944	19600429	1	20110511	8
38281719	20110613	15439109	19470501	1	20110613	8
38283124	20110514	15356051	19470910	1	20110514	8
38285493	20110531	15396815	19640310	1	20110531	7
38287080	20110508	15335273	19301115	1	20110508	10
38287375	20110519	15370205	19300930	1	20110519	9
38288903	20110509	15336793	19490301	2	20110509	10
38290323	20110225	15124327	19520618	2	20110225	7
38290527	20110226	15125462	19351007	2	20110226	8
38290618	20110307	15154551	19540607	2	20110307	10
38291622	20110319	15192082	19230304	1	20110319	7
38291984	20110313	15172581	19640102	1	20110313	8
38293559	20110307	15153577	19541120	2	20110307	8
38294121	20110310	15166755	19280605	1	20110310	8
38294176	20110312	15171923	19280904	1	20110312	10
38294756	20110517	15363073	19501210	1	20110517	7
38295511	20110313	15172769	19530205	1	20110313	10
3829607020130506		17500846	19241112	2	20130506	7
38297040	20110319	15191920	19410121	2	20110319	10
38298032	20110322	15199695	19710522	1	20110322	10
38298236	20110324	15205366	19460925	2	20110324	8
38298612	20110325	15208519	19320211	1	20110325	10
38299104	20110504	15324740	19380203	1	20110504	8
38299433	20110330	15218092	19600408	2	20110330	9
38299831	20110330	15218394	19530906	1	20110330	7
38301052	20110604	15413970	19290124	1	20110604	8
38301198	20110518	15366352	19301101	2	20110518	7
38301518	20110509	15339259	19521220	1	20110509	8
38303321	20110516	15357120	19600210	2	20110516	8
38304380	20110506	15332693	19410313	2	20110506	7
38305010	20110513	15354449	19550507	1	20110513	9
38306897	20110521	15375041	19510425	1	20110521	8
38308984	20110619	15455576	19570118	2	20110619	8
38309374	20110516	15358745	19721230	2	20110516	8

38310666	20110515	15356540	19780125	1	20110515	8
38311498	20110821	15641164	19331104	1	20110821	8
38312297	20110527	15390443	19390528	1	20110527	7
38313790	20110520	15373074	19590405	2	20110520	8
38313836	20110530	15393541	19720207	2	20110530	8
38314997	20110517	15363433	19400118	1	20110517	8
38315058	20120625	16541943	19620916	2	20120625	7
38315230	20110521	15374985	19530731	2	20110521	8
38318886	20110528	15391684	19531102	1	20110528	10
38320466	20110616	15450602	19580228	1	20110616	8
38320897	20110521	15375096	19420214	1	20110521	7
38321005	20110626	15473830	19580914	2	20110626	7
38321710	20110525	15384614	19960526	1	20110525	8
38323681	20110526	15386761	19951212	1	20110526	8
38325767	20110528	15391580	19631012	2	20110528	8
38328142	20110612	15434522	19480225	1	20110612	7
38329098	20110526	15387670	19510907	2	20110526	7
38334393	20120803	16662167	19500303	1	20120803	8
38341832	20110408	15244304	19511219	1	20110408	7
38342562	20110406	15234006	19331018	1	20110406	7
38342993	20110408	15244543	19811213	1	20110408	8
38343021	20110408	15247502	19460527	1	20110408	9
38346815	20110416	15272284	19441112	1	20110416	9
38347261	20110417	15272776	19971007	1	20110417	9
38347987	20110420	15284208	19480710	2	20110420	10
38348355	20110421	15287536	19400118	1	20110421	10
38348855	20110422	15289226	19271105	2	20110422	9
3834952920131206		18181590	19520918	2	20131206	7
38352248	20110607	15419228	19551124	1	20110607	8
38358304	20110614	15442851	19350806	2	20110614	7
38358940	20110613	15437613	19630622	2	20110613	8
38360086	20110529	15392144	19650220	2	20110529	7
38360097	20110520	15373706	19530122	1	20110520	9
38361921	20110612	15434703	19550405	1	20110612	8
38363950	20110622	15464051	19730414	2	20110622	8
38364293	20110626	15473755	19670628	1	20110626	8
38366802	20110808	15605041	19750712	2	20110808	8
38367032	20110802	15584309	19450513	1	20110802	7
38367598	20110715	15537179	19500415	2	20110715	7
38368886	20110919	15719929	19650409	1	20110919	8
38369403	20110723	15556421	19680720	1	20110723	8
38375438	20110620	15459062	19611201	1	20110620	8
38376408	20111004	15766728	19650913	1	20111004	10
38378675	20110616	15447143	19471112	1	20110616	10
38379316	20110613	15437928	19710203	1	20110613	8
38382900	20110622	15466318	19550619	1	20110622	9
38384097	20110613	15437515	19521125	1	20110613	10
38384122	20110621	15463052	19520102	2	20110621	7
38385023	20110904	15678969	19541201	1	20110904	7
38386593	20110823	15648051	19540816	1	20110823	10
38386695	20111017	15804746	19661002	1	20111017	7
38386935	20111227	16012619	19571008	1	20111227	7
38391036	20110711	15522592	19571015	1	20110711	10
38391070	20110630	15485276	20050620	2	20110630	10
38391865	20110718	15542881	19631209	1	20110718	8

38395867	20110708	15516299	19530801	2	20110708	10
38396724	20110726	15563589	19740703	1	20110726	10
38397216	20120202	16111576	19410707	1	20120202	8
38399165	20110814	15621995	19560210	1	20110814	7
38399676	20110815	15625467	19470103	1	20110815	8
38400850	20110719	15546732	19650529	2	20110719	8
38402765	20110810	15612543	19450306	1	20110810	8
38403086	20110625	15473380	19540129	2	20110625	10
38403757	20110602	15407943	19611224	1	20110602	7
38408967	20110430	15308776	19460219	2	20110430	8
38409006	20110429	15307410	19301003	1	20110429	9
38409073	20110612	15434647	19640112	1	20110612	10
38409200	20110430	15308875	19340707	2	20110430	8
38409302	20110501	15309105	19290725	1	20110501	8
38410763	20110505	15329056	19420301	1	20110505	9
38412770	20110517	15361716	19540916	1	20110517	10
38413762	20130417	17444135	19500320	1	20130417	7
38415600	20110602	15407045	19711202	1	20110602	10
38415780	20110614	15443067	19711122	1	20110614	8
38416625	20120319	16254136	19331210	1	20120319	10
38416669	20110715	15537053	19600920	1	20110715	7
38416921	20120706	16578818	19470309	2	20120706	7
38421920	20110826	15656572	19600207	1	20110826	9
38422138	20111211	15966450	19610331	1	20111211	10
38423813	20111031	15838999	19450728	2	20111031	10
38425080	20131204	18172461	19650519	1	20131204	7
38425535	20110825	15651365	19600424	2	20110825	7
38425591	20110717	15539218	19500929	2	20110717	10
38425853	20110724	15557970	19420731	1	20110724	8
38425922	20110727	15566602	20040112	1	20110727	10
38426005	20110617	15453650	19161102	1	20110617	8
38426312	20110623	15468316	19640105	1	20110623	8
38427020	20110715	15536619	20040401	2	20110715	7
38427213	20110628	15479896	19270602	2	20110628	8
38429968	20110801	15580649	19421018	2	20110801	7
38432756	20110627	15476886	19950918	1	20110627	10
38435335	20110621	15462532	19570118	2	20110621	7
38435880	20120830	16739395	19391213	1	20120830	8
38436952	20110816	15628438	19570304	1	20110816	8
38458832	20111025	15827497	19540220	2	20111025	10
38460321	20110625	15473340	19610820	1	20110625	8
38463820	20110629	15483194	19971201	1	20110629	10
38465713	20110626	15473907	19520601	1	20110626	10
38466976	20110726	15564573	19470217	1	20110726	10
38470096	20110625	15473329	19310228	1	20110625	7
38471908	20110730	15574485	19681202	1	20110730	9
38473722	20110821	15641306	19380306	1	20110821	7
38475626	20110621	15461727	19410416	1	20110621	10
38475706	20110621	15462964	19620915	2	20110621	9
38477075	20111114	15887147	19440723	1	20111114	8
38477202	20110627	15476291	19390109	1	20110627	9
38478078	20120321	16260391	19231012	2	20120321	8
38483088	20130324	17365888	19820217	1	20130324	8
38483919	20110701	15489136	19960926	2	20110701	10
38485766	20110524	15382024	19411228	2	20110524	8

38486087	20110525	15382353	19540321	1	20110525	10
38486601	20110603	15412081	19490401	2	20110603	7
38486769	20110527	15389902	19430312	1	20110527	7
38486805	20110527	15389805	19651115	1	20110527	7
38486907	20110527	15390385	19950929	2	20110527	10
38487922	20110602	15408031	19461204	2	20110602	7
38488798	20110604	15413969	19310828	1	20110604	7
38490516	20110607	15419480	19330819	1	20110607	8
38490776	20110607	15419524	19821207	2	20110607	10
38490969	20110608	15422989	19690103	2	20110608	10
38493139	20110820	15640342	19360907	1	20110820	10
38493559	20110614	15443205	19560502	1	20110614	9
38497506	20121126	17009487	19510801	1	20121126	8
38497733	20110715	15536709	19530410	2	20110715	7
38501621	20110818	15635331	19580707	1	20110818	10
38502306	20110805	15597573	19310408	1	20110805	8
38505690	20110803	15589120	19600824	2	20110803	7
38506422	20120501	16376504	19540228	2	20120501	7
38506717	20120913	16787097	19270823	1	20120913	7
38507378	20110807	15600585	19720614	1	20110807	10
38510155	20110717	15538995	19551215	2	20110717	7
38516642	20110719	15546663	19760829	2	20110719	8
38517850	20110804	15594058	19610126	1	20110804	10
38519958	20110717	15539093	19480225	1	20110717	9
38520273	20110621	15460937	19690101	1	20110621	10
38521243	20110620	15459601	19630210	1	20110620	7
38521787	20110622	15466482	19761130	2	20110622	10
38521801	20110624	15471124	19460923	1	20110624	10
38522280	20110624	15471998	19680616	2	20110624	7
38522428	20110624	15472362	19250913	2	20110624	9
38522473	20120624	16537995	19580731	1	20120624	7
38523556	20110717	15539353	19511220	1	20110717	7
38525392	20110727	15567845	19430903	1	20110727	8
38527898	20111003	15761734	19740812	2	20111003	7
38529123	20110717	15538998	19441101	1	20110717	8
38529930	20110718	15540828	19710620	1	20110718	8
38530664	20110824	15650123	19470528	1	20110824	7
38532295	20110717	15539072	19620101	1	20110717	7
38533776	20110720	15549871	19761113	2	20110720	7
38540919	20110728	15569524	19380930	2	20110728	7
38541376	20110726	15563403	19501203	1	20110726	7
38545107	20110821	15641363	19591101	1	20110821	7
38545130	20111008	15781810	19411018	1	20111008	9
38547909	20110810	15612753	19710405	1	20110810	8
38548673	20110822	15642577	20021210	1	20110822	8
38554108	20110906	15688318	19731029	1	20110906	7
38555930	20110815	15624481	19610830	1	20110815	8
38556739	20110908	15696323	19380612	1	20110908	7
38558133	20110823	15647929	19920411	1	20110823	8
38558597	20110825	15654112	19580910	2	20110825	8
38559329	20110925	15738089	19680423	2	20110925	7
38560666	20110725	15560710	19601201	1	20110725	10
38560939	20110801	15580630	19680423	2	20110801	8
38561783	20110722	15553545	19990919	2	20110722	8
38562128	20110819	15639347	19541002	1	20110819	10

38562457	20110724	15558053	19760224	1	20110724	7
38564237	20110812	15618270	19750727	1	20110812	8
38564691	20110802	15584655	19750203	1	20110802	8
38567054	20110728	15570203	20090613	1	20110728	10
38567838	20110801	15578935	19510503	1	20110801	8
38568819	20110728	15570499	19970324	2	20110728	10
38570513	20110728	15569287	19500926	1	20110728	8
38572100	20110801	15578745	19600819	1	20110801	9
38572597	20110809	15608317	19380808	2	20110809	10
38575461	20110805	15598549	19590718	2	20110805	7
38575905	20110821	15641291	19741226	2	20110821	8
38576293	20110805	15597477	19940410	1	20110805	8
38576395	20110808	15604684	19680608	1	20110808	7
38576635	20110807	15600749	19430911	1	20110807	10
38577105	20111003	15760588	19361117	1	20111003	8
38577707	20110811	15615567	19570522	1	20110811	9
38580255	20110812	15618424	19670817	1	20110812	10
38584075	20111120	15903598	19651005	1	20111120	7
38586559	20111017	15804927	19541105	2	20111017	10
38587245	20110908	15693502	19590921	1	20110908	10
38588599	20111017	15804218	19430124	1	20111017	8
38588657	20111019	15810048	19391210	1	20111019	10
38588851	20111231	16021856	19420520	1	20111231	8
38589263	20111101	15845187	19440108	1	20111101	8
38589321	20111025	15824802	19430120	1	20111025	7
38589650	20111027	15832774	19920902	1	20111027	7
38590566	20110815	15625337	19570313	2	20110815	7
38593349	20110816	15629231	19930310	1	20110816	10
38593918	20110821	15641145	19720222	2	20110821	7
38594137	20110814	15622079	19510510	2	20110814	7
38596622	20110815	15623265	19580711	1	20110815	8
38599938	20110905	15683266	19490915	1	20110905	7
38602050	20110912	15702523	19691228	2	20110912	10
38603677	20110925	15738316	19390909	1	20110925	8
38604181	20111014	15795926	19420822	2	20111014	8
38605786	20120102	16025949	19810111	2	20120102	10
38605979	20120112	16062342	19630720	2	20120112	7
38606314	20120724	16632976	19430715	1	20120724	8
38609335	20121218	17082763	19550421	2	20121218	7
3860947120130102		17123318	19621020	2	20130102	9
3860951720130228		17288279	19430825	1	20130228	7
38613535	20110920	15725765	19430907	1	20110920	7
38616250	20110726	15564086	19590128	1	20110726	8
38616692	20110730	15574647	19330507	2	20110730	7
38617059	20110730	15574705	19630610	1	20110730	8
38617355	20110731	15574773	19910307	2	20110731	8
38617902	20110802	15585829	19390408	2	20110802	8
38618609	20120508	16404012	19550908	1	20120508	9
38619011	20110807	15600657	19541024	1	20110807	7
38619088	20111209	15962886	19710829	1	20111209	8
3862050720131219		18223333	19401128	1	20131219	8
38621099	20110818	15634658	19240627	1	20110818	8
38621680	20120326	16269313	19500707	1	20120326	7
38622592	20110821	15640911	20051218	2	20110821	7
38623200	20120826	16729742	19350506	2	20120826	10

38623324	20110824	15650719	19380715	2	20110824	7
38623846	20110826	15657098	19240403	2	20110826	8
38624963	20110829	15661649	19550401	1	20110829	10
38625115	20110801	15580824	19710421	1	20110801	10
38625331	20110808	15604067	19611124	1	20110808	9
38625397	20110905	15683778	19301219	2	20110905	8
38626196	20111213	15972858	19801110	2	20111213	7
38626221	20111215	15981273	19240323	1	20111215	7
38626323	20111216	15982552	19640120	1	20111216	7
38626572	20120202	16111267	19340213	2	20120202	7
38626696	20120207	16128188	19490721	1	20120207	7
38626710	20120208	16131396	19580508	1	20120208	8
38627611	20120301	16196310	19560201	1	20120301	8
38627713	20120321	16260855	19600529	1	20120321	7
38627780	20120304	16203996	19481012	2	20120304	8
38627871	20120305	16208412	19510813	1	20120305	7
38629231	20110924	15736557	19540830	1	20110924	8
38630181	20121020	16898477	20110721	1	20121020	9
38630330	20110907	15688737	19570819	2	20110907	9
38630896	20110818	15635778	19480301	1	20110818	7
38631275	20110803	15590310	19260121	1	20110803	10
38631366	20110801	15578781	19801212	1	20110801	9
3863272320130812		17806961	19400302	1	20130812	8
38633884	20110927	15744135	19471007	2	20110927	8
38635437	20110819	15637917	20100522	1	20110819	8
38639371	20110829	15661490	19480816	1	20110829	7
38641508	20110828	15658847	19750207	2	20110828	7
38641702	20111202	15940654	19410108	2	20111202	7
38641995	20110830	15664256	19751021	2	20110830	9
38642523	20110916	15716586	19740613	2	20110916	10
38642772	20110828	15659088	19740905	2	20110828	8
38643106	20110831	15666639	19520228	2	20110831	7
38645102	20110901	15671621	19310602	1	20110901	8
38647119	20110907	15692458	19670606	1	20110907	8
38650065	20110915	15712394	19681001	1	20110915	7
38650203	20110916	15716503	19601010	1	20110916	9
38650736	20110917	15718800	19430331	1	20110917	10
38651615	20110920	15725878	19380904	2	20110920	9
38651671	20110923	15736378	19680626	2	20110923	7
38651988	20110926	15741846	19400402	1	20110926	7
38652094	20110922	15732644	19590816	1	20110922	7
38652367	20110923	15736199	19290202	1	20110923	7
38652527	20110924	15736521	19360626	1	20110924	8
38653235	20110927	15744993	19340120	1	20110927	9
38654012	20110929	15750546	19500830	2	20110929	7
38654669	20111001	15756005	19350310	1	20111001	7
38654692	20111028	15835671	19470831	1	20111028	7
38656143	20111024	15823894	19440115	1	20111024	7
38656267	20111028	15836127	20000117	1	20111028	8
38656905	20120812	16690518	20030911	2	20120812	9
38656938	20111115	15891065	19580604	1	20111115	7
38658274	20110905	15682732	19661214	1	20110905	7
38659426	20110913	15705761	19600815	1	20110913	8
38659700	20110926	15740649	19380827	1	20110926	8
38659857	20110929	15750404	19771011	2	20110929	8

38660296	20120324	16268014	19760203	1	20120324	10
38660332	20120326	16271357	19620714	2	20120326	8
38660570	20120402	16288241	19590224	2	20120402	7
38660638	20120413	16330976	20050928	1	20120413	7
38660912	20120409	16315436	19800718	2	20120409	8
38661017	20111221	15996921	19490516	1	20111221	10
38661084	20111222	16000405	19291122	1	20111222	8
38662010	20110930	15752775	19550808	1	20110930	7
38662894	20111017	15804168	19640103	2	20111017	8
38662963	20111018	15806436	19610101	1	20111018	8
38663193	20111130	15930091	19640925	2	20111130	7
38663239	20111123	15911596	19610220	1	20111123	7
38663604	20111204	15943498	19440225	1	20111204	8
38663751	20111128	15924853	19630610	2	20111128	7
38665042	20110913	15706257	19570724	2	20110913	8
38665268	20110912	15702322	19490915	1	20110912	7
38665451	20110831	15666939	19550801	2	20110831	9
38665882	20110904	15678789	19641105	1	20110904	10
38666283	20111124	15916982	20000129	2	20111124	8
38668847	20111016	15800767	19540412	1	20111016	8
38668881	20111127	15921863	19550624	1	20111127	8
38669419	20110913	15705297	19460415	1	20110913	7
38669986	20111007	15779913	19460615	1	20111007	10
38671760	20120109	16051036	19721207	1	20120109	10
38673608	20110918	15719332	19400912	2	20110918	7
38674521	20110926	15739972	19870712	2	20110926	8
38677768	20110908	15696405	19691010	1	20110908	8
38681264	20110908	15695820	19461229	1	20110908	8
38682052	20120830	16741155	19510705	1	20120830	7
38682950	20110914	15708479	19741115	2	20110914	9
38683782	20110921	15730130	19630218	1	20110921	8
38686305	20111025	15826822	19640105	1	20111025	7
38686350	20110925	15738175	19501015	1	20110925	8
38688583	20111014	15798094	19550209	2	20111014	8
38688787	20120129	16094533	19650115	1	20120129	8
38691202	20111113	15883383	19420613	2	20111113	10
38691280	20111030	15837895	19540920	2	20111030	8
38693468	20111008	15781872	19491005	2	20111008	8
38693731	20111011	15786375	19901206	1	20111011	7
38695953	20110918	15719193	19570606	1	20110918	7
38697200	20110919	15722774	19550930	1	20110919	9
38700311	20110920	15726603	19590830	2	20110920	8
38700924	20111011	15786703	19951104	1	20111011	8
38701836	20111012	15791469	19470303	2	20111012	8
38703605	20110921	15729800	19321107	1	20110921	7
38703638	20111018	15808488	19660801	1	20111018	9
38703650	20110928	15747423	19740416	1	20110928	8
38703934	20111015	15799624	19370105	1	20111015	8
38706615	20111014	15798517	19370726	1	20111014	8
38706922	20110926	15739511	19640919	2	20110926	8
38707469	20110930	15752135	19481012	1	20110930	7
38711352	20111004	15766831	19590924	2	20111004	7
38711374	20111018	15808505	19830121	2	20111018	8
38718615	20111214	15977552	19600703	1	20111214	7
38721834	20111019	15810997	19450109	1	20111019	7

38722622	20111017	15804484	19640705	1	20111017	10
38727967	20120401	16285266	19650207	1	20120401	8
38728277	20111119	15903023	19400427	1	20111119	8
38730777	20111011	15785955	19530312	2	20111011	7
38731543	20111004	15766719	19371224	2	20111004	8
38732897	20111105	15861974	19560203	2	20111105	7
38733367	20111102	15851480	19320405	2	20111102	7
38735330	20111019	15812144	19360410	1	20111019	8
38746826	20120506	16394930	19491022	1	20120506	8
38747932	20111023	15820667	19530320	2	20111023	10
38750173	20111017	15805018	19530123	1	20111017	10
38750537	20111021	15816322	19591031	1	20111021	8
38750548	20111025	15825430	19610412	2	20111025	7
38750560	20111026	15829549	19630527	1	20111026	9
38750695	20111106	15862571	19481102	1	20111106	8
38754051	20111114	15887320	19530110	2	20111114	7
38754197	20120216	16159072	19420523	1	20120216	8
3875530520130108		17145176	19210302	1	20130108	9
38756353	20111007	15779998	19481015	2	20111007	8
38757276	20111010	15782923	19761126	1	20111010	7
38758326	20111016	15800815	19391212	2	20111016	7
38760064	20111021	15818281	19441222	1	20111021	8
38761181	20111026	15830339	19560218	1	20111026	8
38761318	20111025	15827515	19560329	1	20111025	10
38761329	20111025	15827488	19870912	2	20111025	7
38762117	20111028	15836074	19780221	1	20111028	8
38763336	20111104	15859482	19640912	1	20111104	8
38763369	20111105	15862032	19620408	1	20111105	7
38763461	20111104	15859021	19560922	1	20111104	7
38763563	20111219	15990698	19330903	1	20111219	8
38766299	20111122	15910720	19341217	1	20111122	10
38770295	20111113	15883439	19530423	2	20111113	8
38771152	20111112	15882813	19520505	1	20111112	9
38772859	20111111	15880598	19300922	2	20111111	8
38773603	20111205	15947355	19560512	1	20111205	10
38773647	20111117	15897265	19691119	2	20111117	8
38778380	20111121	15906371	19550205	1	20111121	10
38778608	20111129	15927516	19511007	1	20111129	8
38779101	20111121	15905338	19401020	1	20111121	8
38780197	20111023	15820235	19681220	2	20111023	8
38780891	20111105	15861988	19530324	1	20111105	7
38781167	20111107	15865287	19590417	1	20111107	8
38782808	20111102	15850696	19730313	1	20111102	10
38785556	20111027	15832417	19590302	2	20111027	8
38789401	20111030	15838063	19671123	2	20111030	8
38789581	20111102	15850687	19510922	2	20111102	7
38789605	20111031	15838987	19561031	1	20111031	10
38790179	20111114	15887177	19440708	1	20111114	10
38802469	20111130	15930599	19620202	1	20111130	10
38804056	20111206	15951206	19650203	1	20111206	8
38806654	20111224	16005168	19481201	1	20111224	10
38811197	20111110	15875734	19310122	1	20111110	8
38812907	20111110	15877720	19350615	1	20111110	7
38813488	20111114	15887346	19790806	2	20111114	8
38814232	20111114	15884391	19460125	1	20111114	7

38814685	20111109	15873128	19930710	2	20111109	8
38814958	20111110	15874675	19331130	1	20111110	8
38815724	20111116	15894267	19310420	1	20111116	8
38817548	20111110	15877518	19701101	2	20111110	8
38818687	20111211	15966361	19581025	1	20111211	10
38820041	20120220	16166887	19570419	2	20120220	8
38820198	20111121	15906201	19501117	2	20111121	8
38821953	20120903	16751598	19820511	2	20120903	8
38829355	20111114	15886346	19431217	1	20111114	8
38830874	20111203	15942776	19730302	2	20111203	8
38833420	20111210	15965887	19510825	1	20111210	7
38833453	20111120	15903637	19590614	1	20111120	8
38833942	20111118	15901129	19550907	2	20111118	7
38834707	20120109	16051619	19470110	1	20120109	7
38835755	20120102	16027243	19630209	2	20120102	8
38836612	20120102	16024313	19690310	1	20120102	7
38836861	20120316	16247402	19790804	2	20120316	8
38836941	20120112	16062851	19920612	2	20120112	7
38837217	20120102	16027351	19521008	2	20120102	7
38838072	20120215	16155307	19410113	1	20120215	8
38840027	20111210	15965541	19530314	1	20111210	7
38841393	20120111	16058830	19741029	2	20120111	10
38841826	20120207	16128370	19521110	1	20120207	7
38842078	20120129	16094457	19560309	2	20120129	8
38845873	20111205	15947419	19501025	1	20111205	8
38849853	20111124	15916538	19651216	1	20111124	10
38850123	20111108	15870697	19620517	1	20111108	10
38850338	20111109	15874586	19310625	2	20111109	9
38850769	20111111	15881190	19560802	2	20111111	8
38852221	20111117	15897194	19620722	1	20111117	7
38854170	20111124	15917038	19641030	1	20111124	8
38854181	20111125	15917086	20100711	1	20111125	10
38855606	20111129	15927312	19431008	2	20111129	8
38855640	20120206	16124531	19680108	1	20120206	9
38856018	20111130	15930982	19670114	2	20111130	8
38856665	20111212	15970565	19580505	2	20111212	8
38858036	20111209	15964190	19421019	2	20111209	7
38858398	20111209	15964055	19460515	1	20111209	8
38859675	20111215	15980787	19391010	1	20111215	7
38861175	20111123	15912825	19450818	2	20111123	7
38861653	20111124	15915489	19700203	2	20111124	10
38863160	20120130	16097862	19741006	2	20120130	7
38863182	20111213	15973776	19580217	1	20111213	7
38863897	20111128	15923990	19390810	1	20111128	8
38866090	20111130	15930945	19590811	2	20111130	7
38866910	20120424	16357141	19550605	1	20120424	8
38868041	20111219	15990853	19910518	2	20111219	8
38868052	20111129	15927919	19530420	2	20111129	8
38868212	20111211	15966249	19301127	1	20111211	7
38870223	20120127	16092122	19610904	2	20120127	8
38870381	20111218	15986799	19541022	1	20111218	9
38871613	20111222	16000759	19290423	1	20111222	7
38871919	20111223	16003807	19671209	2	20111223	8
38871920	20111223	16003754	19620428	2	20111223	10
38872025	20111224	16005051	19640606	1	20111224	7

38873131	20111228	16012673	19960806	1	20111228	10
38873506	20111229	16018426	19411130	1	20111229	7
38873573	20111229	16018264	19981206	2	20111229	10
38875911	20111210	15965819	19690615	2	20111210	8
38877677	20120915	16793983	19500623	2	20120915	7
38877735	20111213	15974251	19741128	1	20111213	8
38879491	20120126	16090707	19630108	1	20120126	8
38880001	20111213	15973975	19570823	2	20111213	8
38880716	20120114	16068961	19740613	2	20120114	7
38880749	20120226	16182752	19251112	2	20120226	8
38880772	20120112	16061831	19650202	1	20120112	8
38882632	20111213	15974429	19570225	1	20111213	7
38884718	20111209	15963589	19821211	1	20111209	8
38888265	20111215	15980458	19741210	1	20111215	10
38889995	20111215	15980270	19520421	1	20111215	7
38890798	20111221	15997558	19590325	1	20111221	8
38891202	20120130	16095113	19591010	1	20120130	7
38891304	20111228	16015514	19421023	1	20111228	9
38894370	20120110	16055961	19830919	1	20120110	7
38895226	20111215	15980609	19590603	1	20111215	8
38897584	20120528	16457124	19591206	2	20120528	7
38897722	20121207	17049047	19620516	1	20121207	8
38906615	20111221	15997166	19710615	1	20111221	7
38907185	20120205	16119657	19970826	1	20120205	7
38909636	20120211	16142837	19570128	1	20120211	7
38910279	20120108	16047557	19540115	1	20120108	8
38910439	20111226	16009259	19551029	2	20111226	8
38914839	20121104	16938646	19480812	1	20121104	9
38915014	20120112	16063772	19810113	2	20120112	10
38916346	20120131	16100825	19951129	1	20120131	7
38919801	20120213	16147279	19700429	1	20120213	8
38920580	20111226	16008640	19380530	2	20111226	7
38922439	20111228	16015110	19940522	1	20111228	8
38926077	20120106	16045079	19811127	2	20120106	10
38938395	20120203	16115788	19690310	2	20120203	8
38939321	20131219	18223020	19440604	1	20131219	7
38939832	20120116	16073554	19641114	1	20120116	8
38943021	20120114	16069108	19460113	1	20120114	7
38943203	20130312	17332728	19511020	1	20130312	9
38943509	20120130	16096770	19410523	1	20120130	8
38943656	20120129	16094633	19550201	2	20120129	8
38948219	20120112	16063851	19670514	2	20120112	8
38948355	20120112	16063767	19770209	2	20120112	8
38950322	20120114	16069143	19611207	2	20120114	7
38953661	20120131	16100781	19660509	2	20120131	10
38953898	20120130	16095609	19640824	2	20120130	8
38954255	20120119	16083040	19951007	1	20120119	10
38954608	20120126	16090401	19680716	2	20120126	7
38955543	20120306	16214461	19540427	2	20120306	7
38956240	20120329	16280970	19740330	2	20120329	8
38956422	20120907	16770370	19550625	2	20120907	10
38957925	20120206	16121820	19510708	2	20120206	8
38958917	20120202	16112699	19520322	2	20120202	7
38958940	20120221	16170699	19580526	1	20120221	10
38958973	20120203	16115946	19490528	1	20120203	10

38959125	20120213	16145199	19581211	1	20120213	7
38959636	20120217	16161689	19661119	2	20120217	10
38964975	20120208	16131943	19651106	1	20120208	7
38968308	20120130	16096449	19700904	2	20120130	8
38970080	20120208	16131940	19440509	2	20120208	7
38971049	20120131	16100200	19750502	1	20120131	8
38972188	20120204	16118653	19490423	2	20120204	7
38972291	20120202	16111702	19520420	1	20120202	8
38973852	20120208	16130332	19641222	2	20120208	10
38974173	20120304	16204060	19661217	2	20120304	7
38978197	20120304	16203989	19640306	2	20120304	7
38979010	20120407	16310296	19471005	1	20120407	7
38980164	20120313	16237254	19700105	1	20120313	7
38980733	20120227	16184641	19730828	2	20120227	8
38981565	20120317	16250097	19580312	1	20120317	7
38983038	20120319	16253152	19420927	2	20120319	7
38983516	20120302	16200897	19550908	2	20120302	7
38987256	20120206	16124678	19411007	2	20120206	7
38988191	20120214	16150783	19630330	1	20120214	8
3898883920130829		17859916	19810215	2	20130829	7
38989229	20120208	16131101	19811225	2	20120208	7
38989478	20120516	16428650	19861102	1	20120516	7
38997216	20120626	16544497	20000615	1	20120626	8
38998457	20120219	16164174	19530102	1	20120219	8
38999847	20120324	16268048	19501125	1	20120324	10
39001451	20120225	16182089	19680121	1	20120225	8
39003366	20120227	16185116	19650115	1	20120227	8
39003388	20120226	16182862	19900906	1	20120226	9
39010338	20120130	16096044	19650804	1	20120130	10
39011273	20120202	16111826	19420120	2	20120202	10
39012196	20120316	16245309	19850713	2	20120316	10
39012890	20121101	16929628	19480410	2	20121101	7
39012969	20120209	16135717	19361107	1	20120209	8
39013439	20120209	16136995	19490402	1	20120209	10
39013655	20120210	16140645	19550406	1	20120210	7
39016405	20120223	16177600	19900523	1	20120223	8
39017248	20120224	16180045	19530915	2	20120224	7
39019777	20120221	16171250	19331205	1	20120221	7
39021346	20120222	16174998	19610614	2	20120222	7
39022349	20120222	16173618	19510312	2	20120222	10
39022678	20120225	16182323	19210206	1	20120225	7
39025235	20121110	16962903	19570611	1	20121110	8
39027015	20120226	16182537	19600731	2	20120226	7
39027946	20120305	16206200	19650529	1	20120305	8
39028518	20120314	16240991	19500908	2	20120314	8
39029908	20120305	16209050	19821024	2	20120305	7
39034509	20120311	16229667	19551128	2	20120311	7
39036130	20120417	16337878	19680725	1	20120417	8
39037019	20120316	16248368	19720502	1	20120316	7
39037451	20120410	16319854	19490313	2	20120410	7
39037519	20120327	16273596	19480126	2	20120327	8
39038363	20120314	16240512	19530313	2	20120314	9
39045233	20120222	16174800	19450616	1	20120222	8
39048072	20120221	16170279	20080409	2	20120221	8
39049133	20120224	16179406	20040817	1	20120224	9

39049291	20120224	16180912	19641120	2	20120224	10
39050209	20120227	16185444	19680428	1	20120227	7
39050334	20120227	16185644	19620606	1	20120227	10
39050629	20120228	16187576	19320904	1	20120228	7
39052852	20120307	16214645	19250307	1	20120307	7
39053071	20120307	16218187	19360710	1	20120307	9
39053366	20120315	16245139	19480227	1	20120315	8
39053822	20120312	16233743	19610414	2	20120312	8
39061648	20120309	16225263	19500130	2	20120309	7
39063348	20120313	16236542	19470102	2	20120313	7
39063473	20120314	16241645	19941107	1	20120314	8
39065695	20120310	16229157	19340901	2	20120310	8
39065979	20120406	16306531	19620622	1	20120406	10
39066041	20120328	16278408	19371005	2	20120328	7
39067793	20120410	16319096	19691112	1	20120410	10
39073160	20120426	16364298	19430829	1	20120426	8
39073320	20120402	16291683	19560225	2	20120402	8
39077902	20120402	16287778	19401115	1	20120402	8
39078290	20120329	16281193	19430321	2	20120329	7
39079317	20120416	16334248	19550720	1	20120416	7
39079942	20120418	16342674	19290119	1	20120418	7
3908149720130923		17938225	19690119	1	20130923	7
39084338	20120329	16280622	19410601	1	20120329	7
3908521720130725		17751683	19800823	2	20130725	7
39086823	20120319	16253342	19690110	1	20120319	8
3908962820130621		17641918	19230602	2	20130621	8
39090136	20120314	16241512	19410323	1	20120314	8
39092392	20120322	16263939	19531227	2	20120322	8
39093715	20120326	16271879	19360815	1	20120326	8
3909374820130517		17539003	19210922	1	20130517	7
39093839	20120327	16273786	19701104	2	20120327	8
39094127	20120328	16278297	19591117	1	20120328	9
39094718	20120329	16280242	19691123	1	20120329	8
39094821	20120330	16283026	19510218	1	20120330	8
39095324	20120403	16296852	19380610	1	20120403	8
39096010	20120404	16299334	19460801	2	20120404	8
39096258	20120403	16294924	19620218	1	20120403	8
39097137	20120406	16307232	19960620	1	20120406	10
39097502	20120407	16310508	19860130	1	20120407	8
39099133	20120413	16331494	19250902	1	20120413	8
39099360	20121108	16956795	19520425	2	20121108	10
39101150	20120320	16256909	19980219	2	20120320	7
39103043	20120406	16307209	19470820	2	20120406	10
39110811	20120418	16343424	19391001	1	20120418	10
39111234	20120328	16278435	19641101	2	20120328	7
39116682	20120417	16339755	19560608	2	20120417	7
39117516	20120403	16297004	20000711	1	20120403	8
39119261	20120406	16307719	19280606	1	20120406	8
39125025	20120404	16299605	19561118	1	20120404	7
39126799	20120409	16315115	19510424	2	20120409	8
39129210	20120425	16361322	19350224	2	20120425	7
39130319	20120416	16336090	19381215	1	20120416	8
39131549	20120515	16423779	19700915	2	20120515	8
39132235	20120501	16375515	19590718	1	20120501	10
39133318	20120424	16356619	19700411	2	20120424	7

39135063	20120408	16310744	19550518	1	20120408	8
39136884	20120423	16355392	19530315	2	20120423	7
39145863	20120421	16351215	19520721	1	20120421	7
39149025	20120513	16417461	19590616	1	20120513	8
39149309	20120613	16511606	19540313	1	20120613	8
39149321	20120526	16454209	19601230	1	20120526	7
39150146	20120501	16376137	19490502	1	20120501	7
39150771	20120424	16358770	19830420	2	20120424	7
39151310	20120830	16741975	19600906	2	20120830	10
39153383	20121018	16890773	19540326	1	20121018	7
39153714	20120426	16364638	19760824	2	20120426	8
39153725	20120605	16483342	19700205	1	20120605	8
39154717	20120508	16402716	19531123	1	20120508	8
39154933	20120727	16643148	19671226	2	20120727	7
39155038	20120415	16333749	19520504	2	20120415	8
39155050	20120416	16335723	19670814	1	20120416	8
39155083	20120419	16345721	19801223	1	20120419	7
39155787	20120507	16399540	19490913	1	20120507	8
39155845	20120508	16403945	19510623	1	20120508	7
39156246	20120618	16523697	19510402	2	20120618	7
39157556	20120530	16462451	19750723	2	20120530	8
39159198	20130507	17505385	19400721	1	20130507	8
39160617	20120510	16411869	19610724	1	20120510	8
39160764	20120510	16411168	19610410	1	20120510	7
39161154	20120718	16617360	19390516	1	20120718	8
39161789	20121021	16899062	19791001	2	20121021	8
39162793	20120430	16372999	19560719	1	20120430	9
39164551	20120522	16442503	19640321	1	20120522	8
39165009	20120430	16372802	19530601	1	20120430	8
39165269	20120501	16376743	19940908	1	20120501	9
39166126	20120504	16391855	19590708	1	20120504	10
39168008	20120419	16345818	19580306	2	20120419	10
39168020	20120418	16344088	19390605	1	20120418	9
39171045	20120429	16369701	20090521	1	20120429	10
39171885	20120511	16412235	20080923	1	20120511	10
39172980	20120515	16423338	19370324	1	20120515	7
39173256	20120515	16425247	19540327	1	20120515	10
39173483	20120516	16428784	19770720	2	20120516	8
39178320	20120507	16398225	19690612	2	20120507	8
39178693	20120419	16346487	19550415	1	20120419	7
39178784	20120419	16346104	19551011	1	20120419	9
39179221	20120422	16351992	19611107	1	20120422	7
39180080	20120430	16372272	19600310	1	20120430	8
39180251	20120419	16345804	19510205	2	20120419	10
39180397	20120904	16757675	19450808	1	20120904	8
39180911	20120422	16351738	19590617	1	20120422	8
39181129	20130109	17148347	19751226	2	20130109	7
39181812	20120424	16356925	19511013	2	20120424	7
39181834	20120429	16369378	19331019	1	20120429	10
39183067	20120428	16368991	19681016	1	20120428	8
39183410	20120423	16354624	19741009	2	20120423	9
39183738	20120424	16357575	19570726	2	20120424	8
39183965	20120424	16358182	20010529	2	20120424	8
39184071	20120429	16369721	19661222	2	20120429	7
39185358	20130318	17348680	19451024	1	20130318	8

39188095	20120529	16459900	19570625	1	20120529	7
39188277	20120626	16545271	19570205	1	20120626	8
39189690	20120525	16452158	19990914	2	20120525	8
39190266	20120425	16360439	19440123	1	20120425	7
39190299	20120425	16360838	19540719	1	20120425	7
39190426	20120502	16384090	19540701	1	20120502	7
39194611	20120909	16773059	19720606	2	20120909	7
3919488220131030		18055689	19510928	1	20131030	8
39198475	20120518	16434725	19630503	2	20120518	8
39201451	20120513	16417403	19740109	2	20120513	10
39202250	20120516	16428272	19540614	2	20120516	7
39202772	20120511	16414807	19761006	1	20120511	7
39204290	20120514	16420543	19370815	1	20120514	7
39205748	20120525	16451486	19630903	1	20120525	8
39206218	20120527	16454484	19270212	2	20120527	8
39207846	20120601	16471899	19361130	1	20120601	10
39207868	20120601	16471848	19631218	1	20120601	8
39209057	20120606	16489396	19950502	2	20120606	10
39209171	20120607	16493938	19191102	2	20120607	8
39209262	20120608	16497092	19351117	1	20120608	10
39209386	20120608	16497719	19391225	2	20120608	10
39210496	20120518	16435039	19620415	1	20120518	10
39211557	20120530	16462964	19621126	1	20120530	10
39212130	20120522	16443554	19551013	2	20120522	8
39215377	20120530	16463789	19500822	1	20120530	7
39216029	20120508	16401669	19430205	1	20120508	7
39216541	20120508	16401238	19590326	2	20120508	7
39216643	20120507	16399202	19530120	2	20120507	7
39216665	20120510	16412100	19660608	1	20120510	10
39218036	20120709	16588161	19530702	1	20120709	10
39219073	20120620	16530569	19410214	1	20120620	8
39222190	20120515	16425170	19570819	1	20120515	9
39224505	20120522	16442930	19700809	2	20120522	9
39224527	20120529	16460399	19560510	1	20120529	9
39225940	20120522	16442432	19510127	1	20120522	7
39227924	20120711	16596277	19350401	1	20120711	10
39229522	20120713	16603470	19671112	1	20120713	7
39230563	20120604	16479037	19611101	1	20120604	8
39231102	20120623	16537406	19501011	2	20120623	8
39231486	20120724	16634177	19611211	1	20120724	7
39231793	20120622	16537118	19540620	2	20120622	8
39232923	20120914	16791834	19590325	1	20120914	7
39233880	20120702	16562781	19620325	1	20120702	7
39234781	20120828	16735465	19580828	1	20120828	7
39235284	20120517	16432037	19521231	2	20120517	7
39237406	20120522	16443745	19510428	1	20120522	7
39246816	20120812	16690408	19541201	1	20120812	8
39252534	20120604	16479280	19520703	2	20120604	8
39256605	20120708	16584384	19630320	1	20120708	7
39257200	20120625	16540066	19630205	2	20120625	10
39261115	20120527	16454596	19560330	1	20120527	7
39261988	20120524	16449263	19950403	1	20120524	7
39263359	20120525	16452009	19700903	2	20120525	8
39264727	20120607	16492892	19540105	2	20120607	7
39265480	20120605	16484245	19500502	1	20120605	10

39266438	20120603	16474457	19390217	1	20120603	7
39268149	20130617	17629603	19510220	1	20130617	7
39276114	20120606	16487846	19701011	2	20120606	8
39276250	20120619	16528029	19410217	2	20120619	10
39276625	20120609	16499467	19690208	1	20120609	7
39278256	20120610	16499956	19570701	1	20120610	7
39278881	20120617	16520823	19410329	1	20120617	7
39279920	20120608	16497576	19570218	1	20120608	10
39280756	20120613	16511442	19670507	2	20120613	10
39281895	20120619	16525355	19610206	1	20120619	8
39282230	20120621	16533777	19810426	1	20120621	8
39286890	20130917	17924265	19420409	1	20130917	7
39287166	20120618	16524089	19490613	2	20120618	8
39287575	20120618	16524539	19950807	1	20120618	7
39287597	20120620	16530792	19290504	2	20120620	8
39288034	20120622	16536132	19700817	1	20120622	7
39289753	20120626	16545641	19520217	2	20120626	8
39289800	20120627	16548013	19251011	1	20120627	7
39291106	20120618	16522551	19360410	1	20120618	7
39292712	20120722	16626853	19661031	1	20120722	9
39295255	20120823	16724885	19490107	2	20120823	7
39298527	20120609	16498423	19491118	2	20120609	7
39298969	20120611	16503984	19490912	1	20120611	10
39299848	20120613	16511719	19640102	1	20120613	8
39309245	20120625	16541788	19611002	2	20120625	7
39310786	20120629	16554921	19561112	2	20120629	8
39313070	20120707	16583926	19550102	2	20120707	10
39313821	20120709	16588222	19760327	1	20120709	7
39314700	20120713	16603454	19640618	1	20120713	7
39315656	20120624	16537927	20001123	2	20120624	8
39315838	20121006	16857823	19620815	2	20121006	10
39317572	20120701	16556775	19781117	1	20120701	7
39326482	20120729	16645416	19590910	1	20120729	8
39327645	20120920	16808818	19430820	1	20120920	7
39327861	20131121	18129665	19321103	1	20131121	7
39331594	20120625	16541692	19650822	1	20120625	8
39333067	20120627	16548344	19651201	2	20120627	7
39333589	20120708	16584372	19571012	1	20120708	7
39337218	20120919	16805380	19420323	1	20120919	7
39337809	20121030	16923352	19471214	1	20121030	8
39339521	20121015	16881933	19480801	1	20121015	9
39340222	20120712	16601083	19461015	2	20120712	10
39340799	20120704	16570664	19710125	2	20120704	7
39342728	20120716	16610791	19660401	1	20120716	7
39348135	20121230	17115098	19550910	2	20121230	8
39356075	20120812	16690370	19800322	1	20120812	8
39359289	20120731	16651492	19400716	1	20120731	7
39363401	20120719	16620656	19930225	2	20120719	7
39365509	20120714	16605364	20010531	1	20120714	8
39367334	20120720	16624336	19291111	1	20120720	8
39367505	20120824	16727754	19510513	2	20120824	10
39369307	20120727	16643497	19271024	2	20120727	10
39370564	20120802	16657569	19560430	1	20120802	10
39373290	20120811	16690107	19410507	1	20120811	8
39373905	20120823	16724889	19570204	1	20120823	10

39374022	20120813	16695000	19590109	2	20120813	8
39374077	20120814	16695111	19710518	1	20120814	8
39374851	20120817	16709620	19430305	1	20120817	10
39376299	20120806	16670419	19480101	2	20120806	8
39380502	20120801	16656477	19560323	1	20120801	7
39381925	20120724	16633167	19910411	1	20120724	8
39382086	20120728	16644953	19631224	1	20120728	7
39383341	20120730	16647146	19470404	2	20120730	8
39384300	20120718	16616874	19440626	1	20120718	7
3938470820130622		17646684	19801008	1	20130622	8
39385507	20120721	16626236	19530529	1	20120721	9
39385905	20120806	16670253	19300601	1	20120806	7
39385949	20120805	16664989	19610401	2	20120805	8
39386431	20121202	17025001	19640319	1	20121202	9
39386828	20120911	16780025	19240116	1	20120911	7
39386851	20120902	16746815	19370521	1	20120902	8
39386862	20120903	16749102	19571120	2	20120903	7
39388095	20120807	16672623	19690110	1	20120807	9
39390142	20120724	16634072	19430702	1	20120724	7
39391178	20120828	16736117	19380420	2	20120828	8
39391645	20120911	16781547	19571231	2	20120911	7
39392693	20121001	16835792	19530804	1	20121001	7
39393607	20120909	16773076	19790428	1	20120909	7
39394837	20120923	16814494	19570122	1	20120923	8
39395512	20120719	16620224	19660706	1	20120719	7
3939561420130121		17184596	19531102	1	20130121	7
39396275	20120726	16640766	19471031	2	20120726	8
39398953	20120806	16668691	19390902	1	20120806	7
39399694	20120808	16679792	19610617	2	20120808	10
39400856	20120805	16664833	19490724	1	20120805	7
39400890	20120725	16637416	19551221	1	20120725	8
39400925	20120725	16636939	19450619	1	20120725	7
39400969	20120726	16639625	19501028	1	20120726	7
39401199	20121004	16851550	19580613	1	20121004	8
39406058	20120916	16794316	19950101	1	20120916	7
39406752	20121016	16884443	19530812	2	20121016	8
39407108	20120924	16818078	19340501	1	20120924	7
39415708	20120810	16685885	19460817	1	20120810	7
39417362	20120920	16809316	19521030	2	20120920	10
39421197	20120810	16688202	19331202	1	20120810	8
39422678	20120927	16827130	19720501	1	20120927	7
39427253	20121104	16938753	19510210	1	20121104	10
39428552	20120807	16673804	19370127	1	20120807	7
39432581	20120821	16716924	19551218	1	20120821	8
39435568	20120823	16723849	19431215	1	20120823	8
39436209	20120807	16672883	19480801	1	20120807	7
39438830	20120827	16733017	19410103	2	20120827	7
39439355	20120908	16772548	19701204	2	20120908	9
39455271	20120903	16752474	19500702	1	20120903	8
39456183	20121128	17013506	19550719	1	20121128	8
39458963	20120923	16814350	19611210	2	20120923	7
39461217	20120909	16773047	19650617	1	20120909	8
39467862	20120826	16729683	19520303	1	20120826	10
39471302	20120823	16724164	19560209	1	20120823	7
39473262	20120912	16785115	19590407	2	20120912	7

39475279	20120817	16709600	19780911	1	20120817	7
39476318	20120827	16733121	19510208	1	20120827	8
39477606	20120823	16724462	19460913	2	20120823	7
39477877	20120824	16727894	19671219	1	20120824	10
39478303	20120920	16808392	19520902	1	20120920	8
39481873	20120904	16755749	19521009	2	20120904	7
39482989	20121007	16858199	19600117	2	20121007	8
39490761	20120820	16714874	19360811	1	20120820	7
39495711	20120906	16763875	19490108	1	20120906	7
39495868	20120825	16728756	19590330	1	20120825	10
39496098	20120821	16716871	19550618	2	20120821	10
39502733	20120826	16729701	19810723	1	20120826	8
39510479	20120901	16746396	19720127	1	20120901	8
39511325	20121020	16898532	19430210	1	20121020	8
39511814	20120830	16741907	19661207	2	20120830	10
39512395	20120910	16777475	19710324	2	20120910	8
39513423	20120923	16814355	19700331	2	20120923	7
39513854	20120905	16760586	19280821	1	20120905	8
39520586	20120918	16802186	19460529	2	20120918	7
39520860	20120912	16782984	19541002	1	20120912	8
39521501	20120926	16823640	19510926	1	20120926	7
39523121	20120917	16797612	19650520	1	20120917	9
39526119	20120920	16809205	19741014	1	20120920	10
39526426	20120917	16795732	19230509	1	20120917	7
39527349	20121107	16951927	19350429	1	20121107	8
39528660	20120923	16814375	19641008	2	20120923	7
39529425	20121108	16955899	19501010	1	20121108	8
39529538	20121015	16882194	19500513	1	20121015	8
39529721	20121021	16899216	19621107	1	20121021	7
39531243	20120909	16773118	19570205	1	20120909	7
39531652	20120921	16810251	19660425	1	20120921	9
39531721	20121007	16858226	19671103	2	20121007	7
39531867	20121001	16836598	19370518	1	20121001	8
3953193620121224		17099481	19470102	2	20121224	7
39532213	20121004	16851300	19450423	1	20121004	7
39532235	20121004	16849767	19950831	1	20121004	7
39537343	20120918	16801517	19560215	2	20120918	7
39539509	20121024	16906394	19611005	1	20121024	7
39540051	20121114	16975891	19661216	1	20121114	10
39541178	20120914	16792457	19810124	1	20120914	8
39542762	20120919	16805565	19690528	2	20120919	7
39543787	20120922	16814031	19240910	1	20120922	7
39545965	20120927	16826077	19560827	1	20120927	9
39546651	20121029	16919191	19261118	1	20121029	8
39558855	20121204	17034918	19361204	2	20121204	7
39560526	20121002	16841283	19590715	2	20121002	8
39560899	20121008	16861821	19740403	2	20121008	8
39561767	20121015	16881758	19610828	1	20121015	7
39563183	20121003	16845718	19570511	2	20121003	9
39564197	20121004	16849997	19561031	1	20121004	10
39564573	20121026	16914663	19460121	2	20121026	7
39565907	20121002	16841611	19630525	1	20121002	7
39566400	20121031	16926068	19491002	1	20121031	7
39566911	20121021	16899188	19701209	2	20121021	7
39570417	20121014	16878458	19551202	1	20121014	9

39571261	20120930	16831542	19510813	1	20120930	9
39571750	20121002	16839815	19670306	1	20121002	10
39580433	20121008	16863082	19670422	2	20121008	10
39581083	20121011	16868446	19400114	2	20121011	8
39582360	20121015	16882262	19370709	1	20121015	7
39584399	20121023	16905076	19331118	1	20121023	10
39585610	20121003	16846668	19651014	1	20121003	10
39587398	20121203	17027124	19560214	1	20121203	7
39587729	20120927	16827281	19600704	1	20120927	7
39588017	20121007	16858293	19620324	1	20121007	8
39588799	20121014	16878230	19580622	2	20121014	9
39590802	20121001	16836612	19470220	2	20121001	7
39592057	20121030	16923089	19480619	1	20121030	10
39592251	20121010	16867781	19390213	2	20121010	7
39592364	20121007	16858081	19460618	1	20121007	8
39595136	20130626	17657322	19571028	2	20130626	7
39595294	20121026	16915288	19661029	2	20121026	7
39597825	20121105	16942607	19910529	2	20121105	9
39598055	20121106	16947997	19400323	1	20121106	8
39598715	20121109	16961143	19530808	1	20121109	8
39603093	20121209	17053184	19470725	2	20121209	7
39606150	20121010	16867739	19481215	1	20121010	7
39606707	20130221	17267318	19340424	2	20130221	8
39606956	20121009	16865062	19390324	1	20121009	8
39607426	20121005	16855336	19621116	1	20121005	8
39607482	20121013	16877769	19700914	2	20121013	8
39612254	20121028	16917134	19600625	1	20121028	7
39613893	20121015	16882069	19560430	2	20121015	7
39621266	20121017	16890107	19470302	2	20121017	7
39622963	20121018	16892803	19570824	2	20121018	8
39625359	20121103	16938105	19691103	1	20121103	7
39625995	20121026	16914341	19511201	1	20121026	8
39626249	20121108	16955250	19500302	1	20121108	10
39627333	20121022	16901021	19660420	1	20121022	8
39628303	20121026	16914378	19530320	1	20121026	8
39628347	20130116	17171859	19530128	1	20130116	7
39628643	20121022	16902297	19761108	2	20121022	7
39629715	20121120	16991359	19320429	2	20121120	7
39631986	20121031	16925076	19540323	2	20121031	7
39638341	20121026	16913495	19690130	1	20121026	8
39638749	20121220	17090669	19420811	2	20121220	8
39639800	20121115	16979153	19550111	2	20121115	7
39642596	20121124	17005782	19691109	1	20121124	8
39645039	20121024	16909296	19611119	2	20121024	8
39645233	20121101	16929148	19590115	1	20121101	8
39645788	20130222	17271698	19520109	2	20130222	7
39645960	20121119	16988312	19401218	1	20121119	7
39647751	20121129	17018456	19550302	1	20121129	10
39649859	20121203	17030622	19890421	1	20121203	8
39650981	20130128	17202201	19710413	1	20130128	8
39651008	20121217	17080208	19470909	1	20121217	7
39653720	20121108	16956568	19600224	1	20121108	7
39653899	20121207	17049909	19541120	1	20121207	10
39654358	20121130	17021270	19780224	1	20121130	7
39655577	20121108	16956717	19490103	1	20121108	7

39656354	20121127	17012787	19600915	2	20121127	7
39656570	20121128	17015398	19610202	1	20121128	7
39656923	20121204	17035141	19790806	1	20121204	8
39657982	20121115	16979041	19531013	1	20121115	8
39659739	20121116	16982004	19690414	2	20121116	8
39661466	20121114	16973463	19510701	1	20121114	9
39661977	20121215	17076550	19690911	2	20121215	10
3966571920130131		17212404	19750307	1	20130131	8
3966648320130126		17199402	19430925	2	20130126	7
39666938	20121120	16992884	19290203	1	20121120	7
39667271	20121121	16997706	19560120	1	20121121	9
3966928820121128		17016248	19271106	1	20121128	9
39669459	20121129	17018730	19611010	1	20121129	10
39669551	20121130	17019745	19300301	1	20121130	7
39669755	20121130	17022063	19300523	1	20121130	7
3967119720121206		17045438	20020730	1	20121206	8
39671664	20121209	17053266	19690630	2	20121209	7
39672576	20121212	17066660	19710816	2	20121212	7
3967499220121220		17092527	19450608	1	20121220	8
39684907	20121105	16943327	19560204	2	20121105	10
39686403	20121113	16971906	19490102	2	20121113	9
3969238120130108		17143848	20050612	2	20130108	8
3969242720130111		17157051	19340707	2	20130111	7
3969251820130112		17159060	19370529	2	20130112	7
3969255220130125		17197614	19261018	2	20130125	8
39693339	20121127	17013288	19861006	2	20121127	7
39693475	20121205	17039151	19990707	1	20121205	7
3969351120121211		17060151	19461216	2	20121211	8
39697820	20121126	17009527	19521025	2	20121126	8
39699337	20121203	17031150	19621231	1	20121203	10
3971138120130109		17147827	19641113	1	20130109	8
39711869	20121119	16987777	19580830	2	20121119	7
39711983	20121126	17009585	19331206	1	20121126	10
39712384	20121125	17006477	19501015	2	20121125	8
39712486	20121122	17000649	19530118	1	20121122	8
39715792	20121203	17029886	19260823	1	20121203	10
39715849	20121207	17050482	19591215	2	20121207	10
39717196	20121129	17018466	19821121	1	20121129	7
39722719	20121204	17036475	19610306	1	20121204	9
39723290	20121219	17087956	19600718	1	20121219	7
3972432820130309		17323586	19651105	2	20130309	8
3972455520130116		17170601	19610428	2	20130116	8
39727190	20121203	17031354	19351119	2	20121203	10
39729754	20121126	17008464	19520915	1	20121126	8
3973228020130103		17128139	19481113	1	20130103	9
39732882	20121216	17077461	19601108	2	20121216	10
3973777420121219		17087983	19591205	2	20121219	8
3973876620130114		17162601	19540101	1	20130114	8
3973948520131113		18106619	19431111	1	20131113	7
3974012020130324		17365851	19500326	2	20130324	8
3974266020121231		17116683	19881108	1	20121231	7
39765543	20121203	17029982	19660515	2	20121203	7
3976680820121203		17028966	19430714	2	20121203	8
39768713	20121215	17076647	19680513	1	20121215	7
39771261	20121209	17053120	19630112	2	20121209	7

3979262620130301	17290802	19420819	1 20130301	7
3979301620130502	17489029	19630712	1 20130502	7
3979705220130121	17183242	19520407	1 20130121	7
3980293220130103	17127530	19430928	1 20130103	7
3980319520130221	17267246	20030120	1 20130221	8
3980527120130507	17504385	19710311	2 20130507	7
3980749320130130	17209475	19220408	2 20130130	7
3980806520130106	17135398	19940630	1 20130106	9
3980875820130330	17383242	19510907	1 20130330	8
3981209420121225	17103641	19721016	2 20121225	8
3981496520131128	18151164	19440726	2 20131128	7
3981526420121221	17095699	19500610	1 20121221	7
3981956220130108	17144523	19510502	1 20130108	8
3982133520130114	17161845	19980416	2 20130114	8
3982301320130122	17187200	19640815	2 20130122	8
3982313720130122	17188052	19550129	1 20130122	7
3982360420130123	17191650	19510131	2 20130123	8
3984521120130214	17246029	19510726	1 20130214	8
3984629220121228	17111021	19370606	1 20121228	7
3986515120130423	17460275	19631029	2 20130423	8
3986815020130102	17119770	19610816	1 20130102	9
3986832120130130	17208897	19470122	2 20130130	9
3987042320130107	17135935	19600208	1 20130107	8
3987073020130121	17184092	19800505	2 20130121	7
3987236120130110	17152778	19880505	1 20130110	7
3989648520130228	17287037	19430508	1 20130228	8
3989783120130202	17219714	19570531	1 20130202	8
3990284620130125	17195611	19731218	2 20130125	7
3990315620130202	17219680	19520112	2 20130202	7
3990584520130131	17212418	19520829	2 20130131	9
3990765820130207	17238897	19610110	1 20130207	8
3990776120130208	17240105	19600727	2 20130208	9
3990784120130208	17241383	19220430	2 20130208	7
3991092420130302	17295821	19490707	2 20130302	8
3991173420130220	17264178	19501015	2 20130220	7
3991177820130223	17273632	19430905	1 20130223	8
3991290820130224	17274263	19261002	1 20130224	8
3991321820130225	17278236	19641130	2 20130225	8
3991348920130227	17284472	19370421	2 20130227	7
3991702720130529	17570299	19700313	2 20130529	7
3991737820130125	17197146	19391129	1 20130125	9
3991898420130219	17258009	19610513	2 20130219	8
3991957820130204	17224316	19480804	1 20130204	7
3992165820130129	17206294	19990318	1 20130129	7
3992475920130409	17415580	19401110	1 20130409	7
3993078420130219	17258612	19460922	1 20130219	9
3993171020130219	17259018	19710906	1 20130219	7
3993228020130222	17271263	19490206	1 20130222	8
3993262220130221	17267852	19711114	1 20130221	8
3993473120130320	17356843	19510621	1 20130320	8
3993564320130217	17250570	19530810	1 20130217	7
3993727420130201	17215907	19780515	2 20130201	9
3993791220130214	17245982	19590628	2 20130214	8
3993884620130203	17220643	19781119	2 20130203	9
3994005120130130	17208614	19491206	2 20130130	7

3994029920130201	17217299	19560502	2	20130201	8
3994096020130227	17283486	19621129	2	20130227	7
3994158720130318	17348036	19590615	2	20130318	8
3994195220130401	17390188	19630627	1	20130401	8
3994362920130227	17284897	19441018	1	20130227	9
3995093120130625	17654527	19260302	1	20130625	8
3995297120130207	17238743	19440416	1	20130207	8
3995400220130204	17224943	19581023	2	20130204	7
3996082220130223	17273524	19610818	2	20130223	7
3996470020130224	17274018	19721106	2	20130224	7
3996911420130310	17324258	19420313	2	20130310	7
3997004220130307	17315875	19660421	1	20130307	9
3997224220130317	17345583	19570512	2	20130317	7
3997336920130305	17307908	19600404	2	20130305	7
3997354120130305	17306698	19521127	1	20130305	7
3997490820130324	17365758	19470810	1	20130324	7
3997816020130225	17277647	19490202	2	20130225	9
3998291720130306	17311788	19631025	2	20130306	7
3998599420130313	17336503	19720331	2	20130313	8
3999737020130325	17369816	19550504	2	20130325	9
3999820420130314	17338916	20060817	1	20130314	9
4001037520130410	17419778	19370810	1	20130410	8
4001096620130313	17333850	19730328	1	20130313	7
4001190320130317	17345526	19630714	1	20130317	8
4001203120130331	17383678	19640508	1	20130331	8
4001292820130316	17345112	19671203	1	20130316	7
4001546120130307	17317603	19761217	1	20130307	7
4001689520130312	17332635	19370316	1	20130312	8
4001850620130730	17763263	19731217	1	20130730	8
4001873320130321	17360799	19471025	1	20130321	8
4001973620130324	17365654	19540828	1	20130324	9
4002042620130326	17373294	19470121	1	20130326	8
4002106520130509	17513608	19651011	1	20130509	8
4002247720130403	17400834	19560902	1	20130403	7
4002365220130408	17411470	19530105	1	20130408	8
4002399220130410	17418407	19810901	1	20130410	9
4002564720130324	17365923	19440302	1	20130324	8
4002821520130318	17349387	19820905	2	20130318	8
4002835120130327	17375367	19710809	1	20130327	7
4003117220130416	17438815	19580710	1	20130416	8
4003221120130429	17473283	19771025	2	20130429	7
4003521020130325	17369806	19350226	1	20130325	8
4003607520130322	17363734	19511202	2	20130322	8
4003897920130418	17447898	19510124	1	20130418	8
4004052620130523	17554637	19631215	1	20130523	7
4004137020130420	17452898	19770402	2	20130420	7
4004257720130430	17477628	19550521	1	20130430	8
4004597420130325	17368012	19480817	2	20130325	8
4004882620130404	17402430	19551226	1	20130404	7
4005309620130327	17375823	19601205	2	20130327	7
4005513820130503	17492480	19520401	2	20130503	9
4005521820130411	17422769	19530810	1	20130411	8
4005708720130331	17383885	19570803	1	20130331	8
4005876220130415	17434490	19370218	1	20130415	8
4005952720131106	18081339	19490403	1	20131106	9

4006358920130412	17427627	19640718	1	20130412	9
4006549420130409	17416374	19641029	2	20130409	9
4006757020130823	17845241	19551009	2	20130823	8
4006885720130422	17455366	20120516	2	20130422	7
4006937220130507	17504687	19510326	2	20130507	7
4006951020130416	17439048	19670623	1	20130416	7
4007045920130421	17453309	19710804	2	20130421	7
4007743820130407	17407099	19680215	2	20130407	7
4008207320130429	17474986	19650128	2	20130429	7
4008211920130426	17469686	19610110	2	20130426	8
4008264220130415	17436111	19610525	1	20130415	8
4008283520130416	17438482	20070810	1	20130416	7
4008291520130414	17431637	19520529	2	20130414	8
4008297120130601	17578128	19360316	1	20130601	7
4008404620130412	17426563	19601028	1	20130412	8
4008555020130416	17437309	19720626	2	20130416	8
4008785220130424	17463031	19931205	1	20130424	8
4008911020130430	17478155	19470903	1	20130430	8
4009049120130506	17497814	19700223	1	20130506	8
4009318320130513	17521374	19980311	2	20130513	8
4013060720130703	17680062	19350721	1	20130703	7
4013063020131127	18147797	19581106	2	20131127	8
4013105320130415	17434792	19550928	2	20130415	7
4013500020130428	17471685	19590922	2	20130428	7
4013652520130513	17523762	19601211	1	20130513	7
4013705120130529	17568797	19810905	2	20130529	7
4013706220130531	17574375	19941116	2	20130531	7
4013812320130430	17478276	19470314	1	20130430	7
4013824720130421	17453376	19730611	1	20130421	7
4013829220130424	17463887	19500905	2	20130424	7
4013833820130428	17471791	19420708	2	20130428	9
4013895220130605	17591687	19480215	1	20130605	7
4014070320130502	17485296	19500619	2	20130502	8
4014216320130523	17553706	19491205	1	20130523	7
4014302020130602	17578654	19791102	1	20130602	7
4015090120130424	17462835	19480106	1	20130424	8
4015167520130613	17619018	19821107	2	20130613	7
4015205420130523	17553132	19541225	1	20130523	9
4015308020130428	17471564	19440214	1	20130428	9
4015347720130425	17466014	19970910	1	20130425	7
4015373920130502	17489107	19430530	1	20130502	9
4015432320130429	17474054	19641226	2	20130429	8
4015438920130429	17474551	19630531	2	20130429	8
4015779720130505	17496078	19730818	1	20130505	8
4015898320130501	17479444	19680325	1	20130501	8
4017598220130519	17541120	19540328	1	20130519	7
4017800520130509	17511028	19781027	2	20130509	8
4017801620130509	17511020	19850301	2	20130509	7
4017967720130727	17757111	19560327	1	20130727	7
4017973520130513	17522843	19651103	2	20130513	7
4018214720130530	17573053	19520211	1	20130530	7
4018313920130517	17538328	19470810	2	20130517	9
4018437020130703	17680546	19430414	1	20130703	7
4018438120130605	17592367	19270510	2	20130605	7
4018751720130513	17522454	19441101	1	20130513	7

4018947720130512	17519212	19650112	2	20130512	7
4019033820130521	17548311	19660505	1	20130521	8
4019096320130526	17560422	19541017	1	20130526	7
4019119320130528	17565954	19671225	2	20130528	8
4019256120130602	17578662	19620929	2	20130602	7
4019508220130714	17715970	19310101	1	20130714	7
4019532220130610	17608614	19460410	1	20130610	9
4019621220130614	17622574	19510317	1	20130614	7
4019674520130615	17625034	19900406	2	20130615	8
4019792020130619	17638181	19670722	1	20130619	7
4019811620130620	17640715	19611015	1	20130620	9
4019832120130621	17644822	19401005	1	20130621	9
4019851420130621	17645053	19431130	1	20130621	7
4019940420130625	17651045	19800827	1	20130625	9
4020129420130515	17530638	19901026	2	20130515	8
4020176120130509	17511330	19330608	1	20130509	8
4020285520130514	17527352	19620701	1	20130514	8
4020391620130515	17531051	19730111	1	20130515	8
4020781620130517	17538394	19400525	1	20130517	8
4020807920130610	17607078	19570213	2	20130610	9
4020868220130702	17676898	19700224	1	20130702	7
4020871720130514	17527685	19471201	2	20130514	8
4020897720130517	17538179	19641018	2	20130517	8
4022291120130719	17735759	19510701	2	20130719	7
4022469920130528	17566355	19680704	2	20130528	8
4022487120130528	17566064	19741026	1	20130528	7
4023133220130702	17676661	19730818	2	20130702	7
4023151420130608	17605054	19631010	1	20130608	8
4023268620130605	17592937	19940519	2	20130605	9
4023966520130522	17551738	19530512	2	20130522	7
4024001520130813	17814575	19511125	1	20130813	8
4024041320130606	17598981	19670106	1	20130606	8
4024143820130523	17554901	19970218	2	20130523	7
4024237320130617	17629414	19561201	2	20130617	7
4024614820130604	17588579	19370524	2	20130604	9
4024633120130616	17625440	19500202	1	20130616	8
4024643320130619	17637811	19530902	1	20130619	7
4024760720130801	17768363	19620914	1	20130801	9
4024825720130827	17852557	19640306	2	20130827	8
4024835920130901	17865158	19580710	1	20130901	7
4024837120130830	17863026	19610530	1	20130830	8
4024923820131014	18007496	19161101	2	20131014	7
4024955620131117	18118292	19391024	1	20131117	7
4024984120131121	18133060	19410201	1	20131121	7
4025324520130531	17574562	19550708	2	20130531	7
4025519420130805	17785617	19731016	1	20130805	7
4026005720130629	17665228	19810704	2	20130629	8
4026089720130605	17591472	19620216	1	20130605	9
4026166320130624	17650570	19470824	1	20130624	7
4026853920130611	17611772	19570917	2	20130611	8
4027022220130703	17681150	19631212	2	20130703	9
4027221720130618	17632339	19501012	2	20130618	7
4027392520130630	17665582	19630324	1	20130630	8
4027515820130613	17617926	20000818	1	20130613	7
4027559020130625	17653385	19551027	1	20130625	8

4028026020130614	17621293	19710622	1	20130614	8
4028141220130625	17653844	19400110	2	20130625	8
4028923420131125	18141026	19710204	2	20131125	7
4029076420130726	17754065	19611111	1	20130726	7
4029351420130704	17686776	19550122	1	20130704	7
4029604620130702	17675217	19471010	1	20130702	7
4030036520130716	17724543	19391209	1	20130716	8
4030054720130717	17728656	19630726	2	20130717	8
4030191520130722	17741979	19650410	1	20130722	7
4030233820130724	17748265	19590220	2	20130724	8
4030249620130725	17751410	19301103	1	20130725	8
4030279220130726	17755672	19351221	1	20130726	8
4030304620130727	17757252	19351114	2	20130727	8
4030508620130626	17654907	19571010	1	20130626	7
4030726420130702	17676886	19661214	1	20130702	7
4031052920131119	18125785	19550128	2	20131119	8
4031211620130624	17650181	19390715	1	20130624	8
4031369720130626	17657617	19590720	2	20130626	7
4031466720130629	17665298	19571124	1	20130629	7
4037517520130702	17677258	19650511	2	20130702	8
4037708020130626	17655725	19500214	2	20130626	9
4038078720130715	17719268	19821228	2	20130715	8
4038696720130922	17934087	19680117	2	20130922	9
4038701720130922	17934077	19470203	1	20130922	8
4038725520130923	17937358	19291120	2	20130923	7
4038787920130805	17786315	19710526	1	20130805	7
4038853220130808	17800535	19520828	1	20130808	7
4038872520130809	17803792	19780612	1	20130809	9
4039002120130714	17715910	19500325	2	20130714	7
4040076820130714	17715870	19410807	2	20130714	7
4040373420130708	17697627	19400928	2	20130708	9
4040477120130827	17852845	19371212	1	20130827	8
4040575220130721	17738157	19300901	2	20130721	7
4040622220130710	17707596	19401110	1	20130710	8
4040643720130804	17781003	19910814	1	20130804	7
4040716720130723	17743091	19420212	2	20130723	7
4040926520131016	18014926	19790520	1	20131016	7
4041945220130725	17751586	19480529	1	20130725	7
4042373220130810	17805952	19560617	1	20130810	7
4042415520130808	17798829	19521108	1	20130808	8
4042462220130809	17804242	19560616	1	20130809	7
4042507820130711	17710948	19370503	1	20130711	9
4042611720130811	17806620	19711029	1	20130811	8
4042613920130804	17781046	19510327	1	20130804	7
4042921820130809	17804206	19481110	2	20130809	7
4042968320130902	17869169	19490415	2	20130902	8
4043010220130722	17740037	19300110	1	20130722	7
4043064620130904	17880125	19670826	1	20130904	8
4043158120130731	17764845	19340607	1	20130731	7
4043238020130728	17757621	19580519	2	20130728	9
4043465920130731	17767297	19550212	1	20130731	8
4043686020130929	17954598	19560109	2	20130929	7
4044299720130815	17822658	19591026	1	20130815	7
4044366120130731	17767223	19760716	2	20130731	7
4044457320130916	17921665	19450411	1	20130916	9

4044772120130801	17767342	19490427	2	20130801	9
4044797020131002	17969866	19540810	2	20131002	8
4044799220130803	17780463	19351223	1	20130803	7
4044969220130812	17810601	19861014	1	20130812	7
4045557020130730	17763265	19620331	1	20130730	8
4045577420130806	17789753	19691207	1	20130806	9
4045818220130812	17810224	19500417	2	20130812	7
4045821720130814	17816754	19741124	1	20130814	8
4046104920130731	17765690	19401102	1	20130731	7
4046226820130811	17806842	19711112	1	20130811	7
4046352320131218	18219982	19721210	1	20131218	8
4046998520130916	17920967	19501103	2	20130916	7
4047052820130812	17811264	19770923	1	20130812	8
4047281920130903	17876457	19591112	1	20130903	7
4047387820130917	17925944	19391015	2	20130917	7
4048062420130902	17871763	19711219	2	20130902	7
4048541420130826	17850348	19831129	2	20130826	7
4048561820130815	17823302	19671220	1	20130815	8
4048565220130820	17836695	19541102	1	20130820	8
4048605320130918	17929563	19710202	1	20130918	9
4048700120131009	17994351	19550416	2	20131009	8
4048969820130912	17911626	19510315	2	20130912	8
4049035520130819	17833264	19420331	1	20130819	8
4049045720131227	18242932	19580929	1	20131227	9
4049062820130818	17829220	19481214	1	20130818	8
4049799220130817	17828809	19621210	2	20130817	8
4050160820130822	17841282	19380912	1	20130822	7
4050405020131006	17982042	19690116	2	20131006	8
4050447020130904	17881902	19520125	1	20130904	8
4050469620130905	17887395	19790811	2	20130905	8
4050805220130916	17921407	19440105	2	20130916	8
4051049420130901	17864998	19640923	2	20130901	7
4051720220130914	17916887	19541201	1	20130914	7
4051856720130905	17887422	19481026	1	20130905	7
4051981120131028	18048239	19381103	2	20131028	7
4052316820131004	17978008	19371001	2	20131004	7
4052369120131013	18003061	19500115	1	20131013	7
4052429620130909	17897216	19680601	2	20130909	8
4052592620130910	17902508	19501224	1	20130910	7
4052740020130918	17927481	19630122	2	20130918	8
4052838920130916	17921696	20031010	1	20130916	9
4053002720130929	17954913	19380530	2	20130929	7
4053409620130926	17947143	20010325	1	20130926	7
4053480320131029	18051934	19571216	2	20131029	7
4053805420130913	17914263	19410322	1	20130913	7
4053963720130925	17944960	19550121	2	20130925	8
4054363320130914	17916349	19600930	2	20130914	8
4054444320131110	18095014	19570126	2	20131110	7
4060661920130917	17922358	19680404	1	20130917	9
4060915220131004	17978389	19510128	2	20131004	7
4061144720131007	17985254	19381021	2	20131007	7
4062531820130925	17945464	19240410	1	20130925	9
4062694620130828	17856869	19680821	2	20130828	7
4062759620131008	17989278	19590903	1	20131008	7
4063524320130908	17894045	19710616	2	20130908	7

4063589020130911	17903574	19600921	1	20130911	7
4063640620130913	17914909	19270110	1	20130913	8
4063725020130927	17952767	19270723	2	20130927	8
4063845720131002	17969864	19581106	1	20131002	8
4064419720131021	18028380	19601202	2	20131021	7
4064515620131013	18003092	19581024	2	20131013	7
4064557620131005	17980926	19350921	1	20131005	7
4064691120131024	18040682	19530730	1	20131024	9
4064696620131016	18015573	19600808	2	20131016	7
4064720920131202	18161348	19561114	1	20131202	8
4064834820131020	18025818	19371117	1	20131020	9
4065081520131025	18041456	19870105	1	20131025	7
4065208220131013	18003369	19511102	2	20131013	7
4065572920131029	18051637	19540115	1	20131029	8
4065743020131112	18103612	19371211	1	20131112	8
4066391020131105	18076876	19640528	2	20131105	7
4066506320131024	18040696	19480425	2	20131024	8
4066882420131030	18054328	19461217	2	20131030	8
4066974720131112	18103480	19530601	2	20131112	7
4067090420131116	18117762	19740211	2	20131116	7
4067586320131019	18025155	19261130	1	20131019	8
4067616220131028	18049268	19450902	1	20131028	8
4067731420131022	18032512	20051025	2	20131022	8
4068171820131021	18029540	19480723	1	20131021	8
4068460420131222	18228202	19491220	2	20131222	8
4069151820131010	17997190	19570910	2	20131010	7
4069752720131001	17963604	19610831	1	20131001	7
4069920520131210	18192273	19651026	1	20131210	8
4070048920131112	18101342	19660802	1	20131112	7
4070592820131024	18038592	19780814	1	20131024	7
4070961320131125	18141707	19521224	2	20131125	7
4071916220131222	18228306	19530618	1	20131222	7
4072243820131126	18142493	19501210	2	20131126	7
4072377120131205	18177321	19510224	2	20131205	7
4072614520131112	18101595	19530314	2	20131112	8
4072648520131124	18137920	19401103	2	20131124	9
4073193920131221	18227602	19420307	1	20131221	7
4073196220131106	18082676	19660726	1	20131106	7
4074092920131202	18159329	19410222	1	20131202	7
4074675620131015	18011292	19310514	1	20131015	8
4074763520131018	18023807	19671110	1	20131018	9
4074773720131208	18185400	19510726	1	20131208	9
4076484920131125	18139357	19570201	2	20131125	8
4076548820131202	18160856	19540122	2	20131202	7
4076802320131203	18166281	19661203	2	20131203	7
4076852320131218	18218091	19660825	1	20131218	8
4076856720131221	18227760	19700326	2	20131221	8
4077323720131127	18147856	19331019	2	20131127	7
4077364620131211	18196870	19630215	1	20131211	7
4077471820131202	18159704	19781014	1	20131202	7
4077723920131203	18166618	19400901	2	20131203	7
4078141720131224	18234918	19510523	2	20131224	9
4078331120131226	18239503	19561215	1	20131226	7
4079410320131211	18198767	19610723	2	20131211	7
4079982420131218	18219482	19550308	1	20131218	9

4085626620131222		18228126	19640611	2	20131222	7
4085679920131216		18211105	19740902	2	20131216	8
4085688020131226		18239308	19690908	1	20131226	8
4085738320131217		18216288	19900523	2	20131217	7
4086017120131028		18048650	19420211	2	20131028	9
4086104920131101		18063786	19420307	1	20131101	8
4086177620131104		18071969	19320319	2	20131104	7
4086365820131113		18106749	19590326	1	20131113	7
4089793620131203		18167789	19560328	2	20131203	8
4101013320131213		18206173	19650211	1	20131213	7
4101087120131217		18216279	19681016	1	20131217	7
4101143220131220		18226504	19650620	1	20131220	7
4101154520131221		18227918	19440325	2	20131221	8
4101323420131229		18245636	19750929	1	20131229	9
24113	20111123	15912811	19251227	1	20111123	7
121722	20110623	15469337	19250408	1	20110623	7
133915	20110817	15633244	19230601	1	20110817	10
146598	20110321	15194341	19330318	1	20110321	8
148334	20111227	16010626	19300201	1	20111227	7
178961	20121112	16966540	19310505	1	20121112	8
230013	20111017	15803791	19251116	1	20111017	10
232860	20111021	15817561	19281204	1	20111021	9
243992	20120916	16794456	19301226	1	20120916	8
33359520131023		18036448	19411030	2	20131023	8
36133120131017		18018981	19420219	2	20131017	7
389208	20120929	16831328	19470701	1	20120929	7
426846	20120605	16484746	19201219	1	20120605	8
443550	20110828	15658863	19201225	1	20110828	7
465543	20111020	15814516	19330404	1	20111020	8
470984	20120829	16739152	19300108	1	20120829	8
577311	20121114	16975173	19260612	1	20121114	7
591060	20111003	15761591	19641007	1	20111003	8
66006020131021		18027393	19311029	2	20131021	9
679209	20120226	16182885	19230301	1	20120226	9
687343	20111229	16018079	19340915	2	20111229	8
767737	20120504	16391046	19430701	2	20120504	8
769540	20121008	16863055	19440918	2	20121008	10
82411320130102		17123130	19260513	1	20130102	8
862442	20110630	15486009	19300825	1	20110630	7
864722	20120310	16228775	19240903	1	20120310	10
87934520130607		17602640	19320303	1	20130607	9
88433320130108		17145189	19251018	1	20130108	8
89053920130702		17674275	19390804	2	20130702	8
98599920131012		18002730	19490908	1	20131012	8
98612920130604		17587996	19301229	1	20130604	8
993511	20110623	15467090	19260110	1	20110623	10
102858620130608		17604997	19311215	1	20130608	7
111963720130606		17599103	19330430	1	20130606	7
1195682	20110621	15461748	19670603	1	20110621	8
1199640	20111228	16015404	19330618	1	20111228	10
121038420131109		18094541	19321204	1	20131109	7
1253367	20111017	15803901	19111206	1	20111017	8
1402944	20111205	15945965	19290905	1	20111205	8
142484620130528		17567267	19470628	2	20130528	7
1488535	20120701	16556778	19320202	1	20120701	10

1556861	20120224	16179686	19330614	1	20120224	8
1590085	20130208	17241766	19660723	2	20130208	7
1629136	20130911	17906648	19480228	1	20130911	7
1650233	20120405	16303029	19340102	2	20120405	9
1700363	20131128	18152133	19250911	2	20131128	7
1766741	20120614	16513252	19441005	2	20120614	10
1798921	20120524	16448964	19240715	1	20120524	7
1840795	20130416	17440286	19430225	1	20130416	9
1933171	20110501	15309311	19521010	2	20110501	8
1961553	20110505	15328966	19200929	1	20110505	7
1987560	20110614	15439826	19300205	2	20110614	8
2076564	20110917	15718700	19540708	1	20110917	7
2095058	20120325	16268548	19301011	1	20120325	8
2217449	20110610	15432402	19331121	1	20110610	8
2262364	20130719	17735274	19230610	2	20130719	9
2326332	20120901	16746435	19251126	1	20120901	8
2468920	20120628	16550794	19230130	1	20120628	7
2488417	20120428	16369148	19280915	1	20120428	8
2537680	20110108	14993930	19280906	1	20110108	10
2537986	20110428	15305076	19280721	1	20110428	8
2571359	20110227	15125781	19391120	2	20110227	10
2589142	20110913	15706663	19300510	1	20110913	7
2704098	20120405	16303834	19260403	1	20120405	8
2832759	20120910	16777178	19250310	1	20120910	8
2974007	20110826	15655322	19260325	1	20110826	8
2983746	20121025	16912377	19320104	1	20121025	8
3035663	20130120	17180512	19291103	1	20130120	7
3148285	20110810	15612565	19251107	1	20110810	8
3211436	20130610	17610469	19430404	1	20130610	8
3242588	20120610	16500285	19320508	1	20120610	7
3358110	20110621	15462340	19120816	1	20110621	10
3412371	20130227	17283673	19290613	2	20130227	9
3506294	20110730	15574672	19300320	1	20110730	9
3522789	20130813	17813525	19400806	2	20130813	7
3597588	20130217	17250498	19430221	1	20130217	7
3624337	20110303	15143157	19460904	2	20110303	9
3740270	20130507	17504600	19230301	2	20130507	8
3783742	20110424	15292441	19420522	2	20110424	9
3812495	20110422	15290667	19420314	2	20110422	10
3854668	20110226	15125585	19280901	1	20110226	8
3884422	20120213	16146637	19460720	2	20120213	7
3965853	20111021	15817226	19300303	1	20111021	10
4155048	20131030	18055622	19290519	2	20131030	9
4234791	20110804	15593552	19361112	2	20110804	7
4266971	20110529	15392054	19230513	1	20110529	7
4278153	20130228	17288303	19310510	1	20130228	7
4360489	20120902	16746884	19270508	1	20120902	7
4363659	20120405	16301609	19360720	2	20120405	10
4458031	20120121	16087944	19540111	1	20120121	8
4467394	20111101	15846358	19261004	1	20111101	7
4865592	20130726	17755796	19281127	2	20130726	8
4897034	20110310	15166953	19300906	1	20110310	7
4905980	20111128	15922987	19300915	1	20111128	8
4915257	20120530	16463098	19390715	2	20120530	7
4929537	20120129	16094489	19281009	1	20120129	8

4982278	20121220	17092536	19370112	1	20121220	8
4984741	20111216	15984811	19300816	1	20111216	8
5040697	20130304	17301746	19760205	2	20130304	7
5055356	20130213	17243954	19320921	1	20130213	8
5110690	20110217	15099709	19491101	1	20110217	9
5170365	20131020	18025531	19471116	1	20131020	7
5195788	20110907	15691858	19481028	1	20110907	8
5267323	20130604	17589333	19450801	2	20130604	8
5300152	20130810	17806268	19270204	1	20130810	8
5324356	20130521	17547442	19120723	1	20130521	7
5337199	20110310	15166880	19311228	1	20110310	7
5381317	20110925	15738242	19471119	2	20110925	8
5559795	20110522	15375526	19310724	1	20110522	7
5595948	20121008	16862692	19500213	1	20121008	8
5646468	20111230	16019806	19320905	1	20111230	8
5797411	20120415	16333802	19301029	1	20120415	8
5820622	20130802	17778042	19630823	1	20130802	9
5954610	20120620	16529974	19510502	1	20120620	8
5972883	20130819	17829938	19540527	1	20130819	7
5982332	20110507	15334571	19240921	1	20110507	8
6017341	20130311	17328641	19510115	2	20130311	7
6135699	20130825	17847489	19550304	1	20130825	8
6217012	20130701	17671774	19560207	2	20130701	8
6645029	20130128	17202487	19350803	1	20130128	8
6682366	20110515	15356548	19500510	1	20110515	7
6707524	20120212	16143211	19270627	1	20120212	10
6717233	20130723	17745128	19490216	2	20130723	7
6736669	20120203	16115160	19150823	1	20120203	8
6766967	20110304	15147652	19560208	2	20110304	7
6804779	20130314	17339938	19310106	2	20130314	8
6862017	20130521	17547161	19460822	2	20130521	7
6872931	20120701	16556806	19310505	1	20120701	7
6916934	20121201	17024680	19700103	1	20121201	7
7060846	20120411	16323704	19541215	2	20120411	7
7064097	20131017	18019891	19520722	1	20131017	8
7090144	20120605	16484256	19250418	1	20120605	7
7108738	20110821	15641332	19261225	1	20110821	8
7166963	20111112	15882480	19400113	1	20111112	7
7455096	20110216	15097023	19260101	1	20110216	8
7469865	20120603	16474622	19300911	1	20120603	7
7657149	20130228	17286627	19191018	1	20130228	9
7662524	20130530	17572348	19251115	1	20130530	7
7695525	20110513	15354007	19510818	1	20110513	8
7724427	20120826	16729936	19250621	1	20120826	9
7730612	20120307	16219121	19250917	1	20120307	9
7736858	20110413	15260671	19420116	2	20110413	7
7788854	20110418	15275942	19330315	2	20110418	9
7796794	20120515	16424428	19200601	1	20120515	7
7852713	20110330	15218831	19250510	1	20110330	10
7878764	20121012	16874783	19240712	1	20121012	7
7966383	20120628	16550746	19500423	1	20120628	8
7982005	20110304	15145913	19360310	1	20110304	7
8022546	20111011	15787108	19380130	2	20111011	8
8036768	20110418	15276929	19541129	1	20110418	8
8140612	20121220	17091911	19270412	1	20121220	7

8244119	20110907	15690074	19230212	1	20110907	10
8367213	20120726	16639474	19300902	1	20120726	7
8371208	20130402	17395997	19410701	2	20130402	9
8390054	20131017	18020090	19390829	2	20131017	9
8419827	20110831	15666218	19270312	1	20110831	7
8513880	20111029	15836294	19471130	1	20111029	10
8570158	20110225	15124179	19610514	1	20110225	10
8617376	20110218	15104259	19411020	1	20110218	8
8653483	20120921	16810867	19301001	1	20120921	7
8666497	20110515	15356765	19410116	1	20110515	7
8667650	20120103	16032380	19570420	2	20120103	7
8717746	20131205	18177285	19240414	1	20131205	7
8740316	20120606	16489291	19261128	1	20120606	8
8755304	20130321	17360543	19530730	1	20130321	7
8764134	20110823	15647433	19461106	2	20110823	8
8793235	20110120	15028583	19611108	1	20110120	9
8813954	20121111	16963382	19320521	1	20121111	8
8832846	20130218	17254632	19790324	1	20130218	8
8908369	20111225	16005836	19280206	1	20111225	7
8911920	20110812	15619180	19271006	1	20110812	7
8913653	20130715	17718829	19270128	1	20130715	9
8914532	20110630	15485851	19470210	1	20110630	7
8941217	20110403	15229113	19241003	2	20110403	7
9000822	20130724	17749344	19260820	1	20130724	8
9015274	20130819	17833179	19371016	1	20130819	9
9170654	20130923	17936915	19220622	1	20130923	8
9245243	20130624	17647603	19511106	1	20130624	8
9323379	20120226	16182830	19560701	1	20120226	7
9396343	20110406	15237958	19480804	1	20110406	7
9451096	20130908	17893949	19550301	1	20130908	7
9469141	20111226	16009265	19310626	2	20111226	7
9584161	20120719	16621587	19320928	2	20120719	9
9714027	20120314	16240807	19250925	2	20120314	8
9714878	20111211	15966503	19280202	1	20111211	7
9779480	20120618	16523883	19430916	1	20120618	10
9816620	20130106	17135334	19480619	2	20130106	8
9888748	20110414	15266584	19410510	2	20110414	8
9991746	20111220	15994469	19570914	1	20111220	7
10043740	20110218	15103166	19330103	1	20110218	10
10058089	20110925	15738229	19480211	2	20110925	8
10094505	20120105	16040576	19281010	1	20120105	10
10250132	20130603	17582277	19431014	1	20130603	8
10267433	20110102	14969651	19470613	2	20110102	7
10333365	20130128	17203347	19280421	1	20130128	7
10353910	20130904	17882077	19500713	2	20130904	7
10406458	20121126	17009407	19220511	1	20121126	8
10576564	20110208	15067367	19570925	2	20110208	7
10627051	20111220	15994237	19410305	1	20111220	10
10757014	20111013	15794655	19390116	2	20111013	7
10785985	20110302	15139068	19270811	1	20110302	8
10822794	20130825	17847264	19521223	1	20130825	7
11008381	20120919	16804736	19300603	1	20120919	7
11045117	20111128	15924604	19430211	1	20111128	7
11046541	20130424	17461993	19591102	2	20130424	7
11050127	20120825	16728919	19561031	2	20120825	10

11100917	20130127	17200023	19520827	1	20130127	8
11119816	20130612	17614999	19480320	1	20130612	7
11134999	20111109	15872841	19340621	1	20111109	8
11153881	20120403	16294291	19380308	2	20120403	7
11264349	20120625	16541494	19591106	2	20120625	10
11304919	20131105	18078452	19500104	2	20131105	7
11321509	20130826	17851082	19300616	1	20130826	7
11351181	20111031	15840159	19460628	1	20111031	7
11440270	20110124	15037530	19461226	2	20110124	7
11471015	20120530	16462880	19321010	2	20120530	7
11618727	20111013	15794677	19260719	1	20111013	9
11777723	20130417	17443652	19290511	1	20130417	7
11870545	20110510	15343050	19370815	1	20110510	7
11950451	20120410	16319536	19630812	2	20120410	9
12018530	20130524	17557859	19310226	1	20130524	7
12021113	20120921	16809756	19410426	2	20120921	7
12091893	20130425	17466054	19300626	1	20130425	7
12110655	20120704	16571358	19521017	2	20120704	10
12257011	20120610	16500045	19411016	1	20120610	8
12293468	20111109	15874017	19320727	1	20111109	10
12312242	20130804	17781254	19630112	1	20130804	8
12492692	20110614	15441365	19240624	2	20110614	8
12520433	20130916	17921825	19510420	1	20130916	7
12548413	20120103	16032734	19570319	1	20120103	7
12639848	20130720	17737411	19551028	2	20130720	8
12814050	20130824	17846940	19500425	1	20130824	8
12876947	20110329	15216159	19530628	2	20110329	7
12931247	20110307	15152328	19331010	1	20110307	10
12961329	20110321	15195369	19280201	1	20110321	10
12962060	20121109	16957816	19271028	1	20121109	7
13007211	20111203	15942780	19390329	2	20111203	7
13085386	20110209	15073380	19430603	1	20110209	10
13187876	20121106	16948066	19550816	2	20121106	8
13200958	20120228	16187504	19220817	1	20120228	7
13201473	20110509	15338451	19250906	2	20110509	8
13204530	20110331	15220385	19560622	1	20110331	8
13215753	20110225	15123006	19211107	1	20110225	8
13302631	20120419	16346265	19420528	2	20120419	8
13319498	20130107	17139078	19380615	1	20130107	7
13424301	20120215	16155697	19600815	1	20120215	7
13508559	20130520	17544965	19280503	1	20130520	7
13531356	20130330	17383344	19270815	1	20130330	7
13684587	20130412	17429427	19260522	1	20130412	8
13692201	20121008	16861995	19350404	1	20121008	7
13763638	20111211	15966633	19291208	1	20111211	9
13770815	20120131	16099405	19570825	2	20120131	10
13781049	20120604	16478780	19320305	1	20120604	10
13803297	20120224	16180182	19440528	2	20120224	8
13826749	20120522	16442969	19260101	1	20120522	8
13898674	20110317	15187401	19271222	1	20110317	8
13907454	20110403	15228869	19420620	1	20110403	10
13927101	20130731	17767120	19441205	1	20130731	7
13930580	20130803	17780209	19450102	1	20130803	8
13942911	20131008	17992112	19501020	2	20131008	7
13943981	20110628	15479370	19620614	2	20110628	7

13991827	20120717	16614302	19491026	1	20120717	7
13991850	20120527	16454625	19510510	1	20120527	8
1402157920130710		17705822	19490901	1	20130710	7
14043993	20110622	15463181	19230717	2	20110622	7
14125032	20121015	16881629	19611226	1	20121015	7
14156231	20120808	16679194	19630605	2	20120808	7
1417217920131117		18118299	19500402	1	20131117	7
14173230	20120323	16266262	19450701	1	20120323	8
14386051	20110524	15380855	19700413	1	20110524	8
14451935	20111205	15946480	19330330	1	20111205	8
14453248	20111130	15931121	19320202	1	20111130	8
1449059620130527		17563938	19450602	2	20130527	7
14525281	20120222	16174849	19410320	1	20120222	7
1464341520131202		18161870	19290714	1	20131202	7
14650647	20120427	16365891	19461112	2	20120427	8
14702091	20111022	15819833	19290826	1	20111022	10
14765663	20110804	15594808	19591023	2	20110804	9
14768082	20111123	15913238	19280322	1	20111123	8
1484221820130605		17592581	19390326	2	20130605	7
1489783720121225		17103713	19281020	1	20121225	8
1506457020131114		18110926	19521014	2	20131114	8
1511337820130306		17311973	19460506	2	20130306	8
1518483520130818		17829167	19260712	1	20130818	7
15234523	20111003	15758399	19440711	2	20111003	8
15258534	20120709	16584655	19530912	2	20120709	8
15411435	20120204	16119323	19500621	2	20120204	7
15476070	20121001	16834837	19370523	2	20121001	9
15538257	20120707	16583808	19180505	1	20120707	10
1555151620121110		16963013	19310407	2	20121110	8
15575470	20120130	16098273	19520713	1	20120130	7
15594635	20111212	15969856	19310803	1	20111212	7
15637044	20110626	15473826	19510117	1	20110626	10
15657326	20120808	16679678	19340416	2	20120808	10
15661899	20120229	16190236	19330122	1	20120229	7
15666747	20111013	15794729	19391023	1	20111013	7
1568001020130913		17915285	19310128	1	20130913	7
15713889	20121212	17067073	19560905	2	20121212	10
15759598	20120922	16814008	19510517	1	20120922	7
15827471	20110121	15031291	19600201	2	20110121	7
15833815	20120911	16781513	19310204	1	20120911	7
1585877220130615		17624838	19291230	1	20130615	7
15945003	20120924	16816385	19370303	2	20120924	9
15975232	20120129	16094819	19550623	1	20120129	10
16000514	20111011	15786564	19600528	1	20111011	8
16002736	20111024	15822453	19490310	1	20111024	10
16093086	20111101	15846889	19580717	1	20111101	7
1609882120131028		18048566	19511116	1	20131028	8
16135914	20110605	15414581	19320922	2	20110605	9
16162359	20111230	16020438	19551003	2	20111230	10
16249493	20111018	15807955	19410213	1	20111018	7
16257344	20121118	16985968	19250620	1	20121118	8
16327369	20120214	16151570	19351006	1	20120214	10
16394459	20120207	16129284	19440808	2	20120207	8
16435986	20110615	15445835	19470909	2	20110615	10
16455495	20120523	16446672	19300328	1	20120523	7

16482761	20110320	15192189	19220909	1	20110320	9
16485964	20131016	18015390	19291001	1	20131016	8
16549181	20120725	16637451	19390308	2	20120725	7
16566624	20130724	17747913	19490918	1	20130724	7
16574871	20120507	16399617	19551012	1	20120507	8
16579434	20111223	16003741	19410907	2	20111223	8
16583134	20110407	15242622	19700206	1	20110407	10
16605020	20120514	16420345	19610330	1	20120514	7
16631655	20131202	18161430	19380127	2	20131202	8
16635168	20121120	16994064	19380110	2	20121120	7
16668396	20130625	17652328	19290303	1	20130625	7
16746682	20110228	15127932	19611028	2	20110228	10
16750213	20110316	15182394	19500210	1	20110316	9
16803182	20110922	15732294	19270713	1	20110922	8
16866141	20120329	16279854	19611226	1	20120329	10
16906868	20110818	15636134	19380701	1	20110818	8
16915905	20110724	15557756	19521006	1	20110724	8
16956700	20110824	15649791	19540606	1	20110824	8
16974666	20130911	17907022	19530915	1	20130911	7
16999465	20121227	17109622	19530125	2	20121227	8
17031946	20121016	16884140	19650120	1	20121016	10
17102475	20121007	16858353	19210328	2	20121007	10
17104379	20110107	14991887	19720811	2	20110107	7
17147943	20131114	18111098	19400528	1	20131114	8
17158484	20130424	17463569	19350120	1	20130424	8
17198004	20120812	16690669	19440418	1	20120812	7
17211551	20110512	15351604	19490315	1	20110512	10
17233259	20120714	16606397	19490220	1	20120714	7
17244143	20110619	15455882	19400101	2	20110619	7
17246763	20130515	17530720	19260206	1	20130515	9
17279775	20120612	16507237	19290109	1	20120612	9
17286327	20110908	15694857	19340930	2	20110908	9
17314339	20130420	17452701	19600815	2	20130420	7
17339343	20121117	16985140	19490911	1	20121117	9
17517043	20110212	15083879	19680730	2	20110212	9
17518808	20120130	16098399	19621025	1	20120130	10
17595801	20110406	15237507	19321127	2	20110406	7
17722100	20130116	17171783	19601010	2	20130116	7
17745549	20131002	17969532	19261211	1	20131002	7
17750742	20110429	15307276	19490322	2	20110429	8
17757652	20130314	17339134	19301016	1	20130314	9
17759885	20110504	15324059	19610822	1	20110504	8
17771992	20110515	15356656	19400515	2	20110515	8
17798413	20120826	16729657	19351207	1	20120826	8
17835460	20130225	17277427	19251120	1	20130225	8
17853473	20131117	18118128	19300920	2	20131117	8
17857555	20110220	15106340	19630420	1	20110220	10
17890981	20110406	15237001	19311118	1	20110406	8
17904517	20120320	16255913	19310828	1	20120320	7
17949609	20120311	16229752	19361104	1	20120311	8
17979952	20120221	16171202	19560911	1	20120221	9
17985932	20111206	15952412	19650925	1	20111206	10
18100873	20131204	18173596	19530615	2	20131204	7
18128922	20110903	15678254	19340816	2	20110903	9
18146026	20120910	16776350	19401120	1	20120910	10

18196800	20110316	15182794	19270707	1	20110316	8
18201086	20120212	16143301	19320507	1	20120212	7
18251315	20110314	15176798	19320915	1	20110314	8
18309109	20110322	15199570	19460804	1	20110322	7
18398260	20110611	15434159	19640907	1	20110611	7
18414818	20120319	16252939	19440222	1	20120319	8
18432423	20110312	15172108	19280401	2	20110312	7
18452841	20110830	15663924	19220716	1	20110830	7
18590722	20110416	15272277	19410228	2	20110416	10
18695006	20120224	16180838	19380118	1	20120224	10
18729356	20110620	15459722	19390504	2	20110620	8
18749956	20110113	15006613	19341220	1	20110113	8
18761165	20120712	16599930	19290708	1	20120712	10
18816767	20110704	15498518	19330416	1	20110704	8
18842303	20130810	17806161	19610115	2	20130810	7
18884587	20130316	17345210	19270912	1	20130316	7
18932144	20121125	17006582	19381026	1	20121125	10
18967903	20130118	17178156	19301020	1	20130118	7
19003384	20121226	17105714	19370820	1	20121226	8
19185923	20110317	15186904	19831005	2	20110317	9
19228332	20120903	16750855	19510806	1	20120903	8
19288609	20121015	16881455	19390406	2	20121015	8
19377787	20120807	16671718	19501224	1	20120807	8
19474772	20110412	15258581	19330720	1	20110412	7
19542406	20110511	15347903	19400312	2	20110511	7
19633773	20130615	17625036	19490316	1	20130615	8
19700799	20130824	17846974	19841206	1	20130824	7
19709172	20110618	15455415	19810809	2	20110618	10
19713736	20131028	18048926	19370918	1	20131028	8
19721778	20110305	15148695	19550707	2	20110305	8
19731818	20110513	15353972	19360117	2	20110513	7
19740568	20130616	17625423	19421205	2	20130616	7
19740773	20131015	18011016	19490823	1	20131015	8
19848147	20130414	17431545	19720704	1	20130414	8
19924448	20120615	16518562	19241211	1	20120615	8
19943716	20130501	17483546	19460224	1	20130501	7
20059943	20121118	16986024	19580124	2	20121118	10
20137079	20110727	15565986	19340117	2	20110727	8
20167468	20120516	16428499	19560801	2	20120516	7
20239649	20110914	15710029	19321228	1	20110914	7
20284666	20110423	15291915	19331210	1	20110423	8
20391746	20110222	15113918	19391021	2	20110222	10
20475074	20130420	17452849	19660213	2	20130420	7
20491116	20110508	15335104	19321226	1	20110508	10
20505537	20130404	17403388	19371126	1	20130404	8
20505673	20130401	17387046	19470908	1	20130401	8
20629043	20130609	17605527	19431228	1	20130609	8
20638168	20130328	17378419	19300722	1	20130328	8
20657298	20120701	16556881	19660928	2	20120701	8
20663756	20120627	16547781	19300204	1	20120627	8
20745113	20110328	15211292	19300227	2	20110328	10
20759868	20120521	16440540	19831006	2	20120521	9
20789553	20120806	16670362	19601105	1	20120806	7
20817134	20130226	17281544	19440327	1	20130226	7
20828879	20120613	16511258	19391205	1	20120613	8

20836446	20120522	16442400	19471001	1	20120522	10
20951717	20120603	16474470	19270606	2	20120603	10
21004988	20110830	15664331	19691003	2	20110830	7
21151882	20111204	15943368	19420715	1	20111204	10
21154847	20120309	16226094	19481029	1	20120309	10
21324390	20110807	15600810	19311209	1	20110807	8
21377026	20120920	16808869	19550910	1	20120920	10
21423018	20130319	17351427	19240927	1	20130319	8
21438675	20130901	17865049	19580825	1	20130901	7
21448806	20110411	15254883	19350103	2	20110411	8
21458060	20120425	16360587	19310318	1	20120425	10
21465236	20110215	15091872	19620819	2	20110215	10
21536232	20120229	16189973	19870510	2	20120229	10
21536390	20111221	15996305	19281004	1	20111221	7
21665838	20131218	18219494	19391117	1	20131218	8
21743680	20120115	16069539	19550712	2	20120115	8
21849230	20130830	17862849	19390303	2	20130830	7
21852448	20130409	17416733	19491106	1	20130409	8
21928347	20111116	15894198	19260904	1	20111116	10
22216053	20130910	17901592	19630118	1	20130910	8
22246351	20110727	15566591	19651205	2	20110727	7
22302530	20111022	15819980	19360202	2	20111022	9
22361713	20120726	16640682	19300918	1	20120726	8
22371615	20110803	15586785	19571105	1	20110803	8
22396005	20131019	18025466	19320626	1	20131019	7
22449907	20110913	15703915	19601226	1	20110913	8
22461901	20110228	15128403	19480316	1	20110228	9
22509279	20120518	16434953	19440715	1	20120518	10
22533444	20130409	17416522	19500104	1	20130409	8
22535348	20120720	16624783	19271001	2	20120720	7
22554514	20111129	15926581	19500428	1	20111129	10
22639209	20120809	16684465	19601201	1	20120809	10
22640933	20121129	17018928	19481218	2	20121129	10
22665243	20110915	15713936	19460829	2	20110915	7
22670673	20110420	15283675	19481202	1	20110420	9
22710594	20120825	16728968	19420611	1	20120825	7
22736998	20120703	16566882	19221225	1	20120703	8
22747724	20120922	16814056	19920529	2	20120922	8
22814206	20111126	15921361	19600628	1	20111126	7
22855910	20110717	15539038	19400523	1	20110717	8
22914450	20111124	15917005	19470502	2	20111124	8
22927635	20121112	16965814	19330705	1	20121112	7
22932645	20120125	16089502	19630120	2	20120125	10
22951219	20120923	16814436	19330226	1	20120923	10
22965464	20130524	17557044	19530730	1	20130524	7
22997291	20130505	17496021	19600301	1	20130505	7
23004231	20131212	18202016	19390901	1	20131212	7
23008186	20130301	17289509	19460730	2	20130301	7
23010120	20121021	16899166	19530424	1	20121021	7
23024115	20130804	17781102	19420410	2	20130804	8
23025607	20110810	15612023	19431007	1	20110810	8
23084653	20111215	15981809	19680118	2	20111215	10
23092866	20111214	15976904	19471110	2	20111214	8
23114741	20130207	17239038	19541201	2	20130207	7
23132323	20110813	15621847	19271201	1	20110813	7

23133995	20110625	15473379	19481120	1	20110625	8
23156561	20120404	16299672	19620607	2	20120404	8
23157597	20111202	15939249	19310501	1	20111202	10
23178565	20110918	15719088	19361016	2	20110918	9
23198109	20110220	15106245	19340101	1	20110220	7
2331003420131120		18128675	19690102	1	20131120	7
23338854	20110317	15187448	19310101	1	20110317	9
23367819	20110317	15187440	19580201	2	20110317	9
23383428	20120408	16310564	19300519	1	20120408	7
23415465	20120109	16051860	19290421	1	20120109	10
23463016	20120426	16362581	19340222	2	20120426	7
23513522	20121017	16890250	19421013	2	20121017	7
2353357520131019		18025403	19570906	1	20131019	8
23576332	20111103	15855604	19630326	1	20111103	8
23592010	20110425	15295546	19401214	1	20110425	8
23596614	20110925	15738360	19471210	2	20110925	7
23706732	20110505	15329013	19521114	1	20110505	8
2373390620130929		17954695	19510831	1	20130929	7
2374624920130704		17685726	19560122	2	20130704	8
23767977	20110503	15319445	19330424	1	20110503	10
23784818	20110212	15084180	19400921	2	20110212	7
2382310120130727		17757303	19550420	2	20130727	7
23886999	20111226	16008155	19510528	1	20111226	9
24018006	20120518	16434200	19350317	2	20120518	7
24161920	20110802	15584881	19271015	1	20110802	7
24162445	20110504	15324046	19280615	1	20110504	8
24203938	20110610	15431600	19370224	2	20110610	10
24256299	20121101	16930324	19311019	1	20121101	8
24268722	20120301	16195095	19580710	1	20120301	7
24272591	20120509	16407379	19611125	1	20120509	8
24295554	20111223	16003134	19610620	1	20111223	7
24337222	20120717	16614350	19470510	1	20120717	8
2434011220130612		17614920	19550412	2	20130612	8
24358325	20110516	15360338	19630417	2	20110516	7
24367008	20110207	15063576	19340707	2	20110207	7
24377239	20110916	15716688	19320110	1	20110916	8
24440480	20110522	15375611	19531221	2	20110522	9
24481729	20110224	15120975	19380724	1	20110224	7
24485652	20120508	16401121	19381103	1	20120508	9
2453136020130917		17923712	19470613	1	20130917	7
24531928	20110902	15676056	19500429	1	20110902	10
2455202120130120		17180316	19470826	1	20130120	7
2463401320130516		17535615	19380124	2	20130516	8
24654748	20120130	16095943	19940414	1	20120130	10
2479408920130812		17809958	19410927	1	20130812	7
24799788	20120313	16235319	19340208	2	20120313	7
24812860	20110815	15624709	19371025	2	20110815	9
24884819	20120829	16739067	19371029	1	20120829	8
24955257	20110402	15228315	19381028	1	20110402	7
25011341	20120621	16534245	19690713	1	20120621	7
2502020620130818		17829202	19350909	1	20130818	9
25028631	20111130	15928927	19291004	1	20111130	7
25032228	20111125	15918328	19431020	1	20111125	7
25152787	20120729	16645588	19580310	1	20120729	10
25205587	20110303	15141879	19560813	1	20110303	10

25245458	20120204	16119145	19730219	1	20120204	8
25257641	20120206	16124477	19661130	2	20120206	7
25273476	20120909	16773184	19550427	1	20120909	10
25281816	20110629	15483207	19541221	1	20110629	7
25300167	20110505	15327719	19201022	1	20110505	7
25303939	20110214	15089120	19690910	2	20110214	8
25319317	20110423	15291078	19320428	1	20110423	7
25340049	20121226	17106647	19340317	1	20121226	8
25345613	20110923	15736324	19300825	2	20110923	7
25361426	20120921	16811558	19330615	1	20120921	8
25398747	20110517	15363646	19440117	1	20110517	7
25433878	20121025	16912603	19470223	2	20121025	8
25445583	20110407	15242769	19620116	1	20110407	8
25463052	20111129	15927813	19500419	1	20111129	7
25476259	20110224	15121130	19561115	1	20110224	9
25477285	20120401	16285556	19640529	1	20120401	8
25521895	20130330	17383284	19410628	1	20130330	8
25524189	20110904	15678673	19670419	2	20110904	10
25547482	20121120	16991774	19400209	2	20121120	9
25608075	20110913	15704947	19190723	1	20110913	10
25608564	20110205	15059613	19540620	1	20110205	10
25726878	20130823	17844715	19550718	1	20130823	7
25741428	20110307	15154075	19701113	2	20110307	7
25799955	20130307	17316508	19561019	2	20130307	7
25842346	20110918	15719334	19401120	2	20110918	7
25872371	20120215	16154911	19530624	2	20120215	10
25904238	20110220	15106259	19550404	2	20110220	7
26023916	20120325	16268463	19330119	2	20120325	9
26047256	20131224	18234292	19690623	2	20131224	8
26095716	20111205	15947012	19430628	2	20111205	10
26213467	20130102	17123339	19570503	2	20130102	9
26275398	20130602	17578721	19400107	1	20130602	8
26336685	20110727	15566427	19641207	1	20110727	8
26385240	20110918	15719309	19550119	2	20110918	8
26538409	20120514	16420759	19521221	1	20120514	10
26610037	20130427	17471149	19570930	2	20130427	8
26802891	20110315	15180570	19630102	2	20110315	7
26856644	20120425	16362145	19360318	2	20120425	8
26915684	20110922	15732819	19640605	1	20110922	10
26922156	20130815	17821347	19730522	1	20130815	7
26974538	20130516	17535645	19320927	2	20130516	7
26992018	20131124	18137928	19521005	2	20131124	7
26993793	20120131	16101729	19550707	1	20120131	7
27001918	20110917	15717236	19320211	2	20110917	10
27006628	20130702	17676890	19400519	1	20130702	7
27065174	20111011	15783148	19390202	2	20111011	8
27079465	20131013	18003283	19600922	1	20131013	7
27110210	20120106	16044960	19370325	1	20120106	8
27118918	20110717	15539058	19250530	1	20110717	9
27125140	20120913	16789005	19360222	2	20120913	7
27143993	20120518	16434020	19491024	1	20120518	9
27238193	20110405	15233916	19890529	2	20110405	8
27245950	20130807	17793995	19410101	2	20130807	7
27255852	20110408	15246680	19600728	1	20110408	7
27259047	20110211	15081812	19530306	1	20110211	10

27275565	20110328	15213551	19510623	1	20110328	8
27278246	20120913	16789150	19550508	2	20120913	8
27298346	20110331	15221569	19721117	2	20110331	7
27298744	20110912	15702487	19400815	1	20110912	8
27336910	20120615	16518490	19450808	2	20120615	8
27371284	20120629	16554542	19540509	2	20120629	8
27421609	20111105	15862064	19661219	2	20111105	9
27440353	20120228	16186060	19540228	1	20120228	9
27518872	20110426	15298185	19500102	1	20110426	7
27544770	20111009	15782455	19360405	1	20111009	9
27570963	20110609	15426306	19400604	1	20110609	8
27614897	20110614	15442392	19530301	1	20110614	7
27626182	20110515	15356584	19641225	1	20110515	10
27702212	20121001	16834574	19410128	1	20121001	7
27869187	20110410	15249881	19521228	1	20110410	7
27896806	20121128	17013694	19320420	1	20121128	7
27913402	20130217	17250738	19550328	2	20130217	8
27916945	20120204	16119135	19380227	2	20120204	8
27931971	20130602	17578617	19960215	2	20130602	8
27946629	20130223	17273472	19410906	1	20130223	7
27999902	20120504	16390973	19390827	2	20120504	8
28026597	20120208	16133234	19440519	1	20120208	7
28071489	20130224	17274116	19621209	1	20130224	7
28136096	20110922	15733181	19330526	2	20110922	10
28144334	20130115	17167946	19781120	2	20130115	8
28153404	20130424	17461992	19490501	2	20130424	8
28169815	20130604	17588985	19721006	2	20130604	8
28179079	20110521	15375144	19280123	1	20110521	8
28204173	20130519	17541039	19690616	2	20130519	8
28258551	20131126	18144889	19550208	1	20131126	7
28264053	20120203	16113923	19360222	1	20120203	7
28335902	20131108	18092608	19450105	2	20131108	7
28410788	20110426	15299187	19830530	2	20110426	8
28423474	20120101	16022486	19500110	2	20120101	8
28466117	20130716	17722201	19661224	2	20130716	7
28508681	20120607	16493600	19480515	1	20120607	10
28514207	20110806	15600313	19610910	1	20110806	7
28541857	20131112	18104479	19460622	2	20131112	7
28547684	20120502	16384250	19360120	2	20120502	7
28562530	20131108	18092561	19720824	1	20131108	7
28573457	20131221	18227915	19361025	2	20131221	7
28655416	20131204	18173624	19520103	1	20131204	8
28669069	20120706	16581542	19560125	1	20120706	8
28718163	20130419	17449726	19511201	2	20130419	7
28726945	20131025	18042943	19391210	2	20131025	8
28763362	20110817	15632796	19690601	2	20110817	7
28767842	20120425	16360852	19560915	2	20120425	8
28793671	20111222	16001069	19500915	1	20111222	8
28809974	20130218	17252330	19470113	2	20130218	7
28820000	20120116	16073664	19400715	2	20120116	8
28839385	20130812	17810923	19560705	1	20130812	8
28885861	20110813	15621705	19360726	1	20110813	7
29007623	20110203	15058224	19510723	1	20110203	7
29113595	20110330	15217690	19870623	2	20110330	7
29148172	20130924	17940518	19490417	2	20130924	8

29182818	20111027	15833276	19490606	1	20111027	7
29221758	20120424	16357761	19571220	1	20120424	7
29251841	20120821	16716547	19970501	1	20120821	10
2926472020130729		17760948	19560802	2	20130729	8
29266555	20120613	16510504	19410824	1	20120613	8
29269725	20111212	15969534	19341215	2	20111212	10
29270959	20110211	15082044	19691010	2	20110211	10
29323533	20121021	16899257	19500130	1	20121021	8
29329688	20120828	16735774	19840214	1	20120828	9
29346892	20111212	15967790	19520101	2	20111212	10
29355337	20120703	16566227	19620830	2	20120703	10
29385646	20110408	15246953	19450215	2	20110408	8
2940414620131106		18083804	19610927	2	20131106	8
2941257520131104		18069836	19470910	1	20131104	9
29448553	20120603	16474673	19650301	1	20120603	7
29473287	20121210	17056533	19261115	2	20121210	8
29486393	20110516	15360234	19490402	2	20110516	10
2951091620131222		18228423	19520715	1	20131222	8
29531826	20110607	15419464	19360201	2	20110607	7
29549131	20110520	15373724	19320630	2	20110520	7
29680260	20110407	15243421	19510701	1	20110407	7
29710712	20110702	15493133	19230510	1	20110702	7
29711635	20111021	15818359	19740923	2	20111021	7
29765159	20110801	15580972	19470119	2	20110801	8
29776214	20120720	16623567	19300101	2	20120720	8
29812804	20121028	16917174	19390201	1	20121028	7
29839529	20120815	16702877	19541016	1	20120815	8
29941104	20110813	15621743	19320603	1	20110813	7
29958063	20110217	15100521	19620220	1	20110217	10
30064303	20110722	15555454	19370210	1	20110722	7
30114422	20120129	16094794	19361130	1	20120129	8
30160384	20110327	15210052	19701017	1	20110327	8
3021498320130412		17426760	19250815	1	20130412	8
30237162	20110503	15320101	19670827	1	20110503	8
30258958	20111214	15978154	19270602	1	20111214	7
3026122420131016		18013770	19490106	2	20131016	9
30262056	20110217	15100413	19510512	1	20110217	7
30262603	20120218	16163663	19610327	1	20120218	7
30270156	20120506	16394877	19410611	2	20120506	10
30278876	20121020	16898732	19291105	1	20121020	7
30295217	20120319	16253030	19611120	1	20120319	8
30324642	20110503	15319169	19530827	1	20110503	10
3041176820130102		17121171	19270704	1	20130102	8
3041347920130502		17487558	19510501	2	20130502	8
3043512220130830		17863281	19580826	2	20130830	8
30454401	20111227	16012003	19300801	1	20111227	7
30490541	20120503	16386479	19220301	1	20120503	9
30507905	20120213	16145071	19360526	2	20120213	7
3059918920130519		17540962	19570930	1	20130519	7
30631040	20110522	15375762	19990915	1	20110522	7
30647848	20110302	15139260	19520806	1	20110302	7
30682236	20120212	16143091	19381029	2	20120212	9
30689226	20110815	15624688	19301003	1	20110815	9
30724255	20110711	15523090	19610906	1	20110711	10
30740488	20111206	15952323	19600206	2	20111206	7

30759150	20110525	15384974	19540420	1	20110525	7
30781147	20110523	15377463	19771007	1	20110523	8
3081136020130828		17857779	19410126	1	20130828	8
30839908	20121029	16920265	19400430	1	20121029	9
30994413	20111224	16005274	19520920	1	20111224	7
31055159	20121112	16965273	19441027	1	20121112	10
3110702520131007		17984853	19570804	1	20131007	8
31109850	20111020	15814311	19311215	1	20111020	7
3113645520130608		17604955	19441220	1	20130608	7
3115926920130122		17188187	19370408	2	20130122	7
31244925	20110501	15309160	19460421	1	20110501	8
31257531	20120306	16213281	19660813	2	20120306	7
31272067	20110416	15271191	19321127	2	20110416	9
3127833820130903		17875989	19381015	1	20130903	7
31331303	20120318	16250614	19670304	1	20120318	7
31341272	20110504	15324191	19440202	1	20110504	10
31363152	20110318	15190430	19751022	1	20110318	10
31413771	20110507	15334520	19260806	1	20110507	8
31447797	20120411	16323395	19650112	2	20120411	7
3148129520130115		17166736	19650505	2	20130115	7
3156071120131009		17996795	19360809	2	20131009	7
31567187	20110624	15472278	19681015	1	20110624	7
31592311	20111030	15838011	19631022	1	20111030	7
31768351	20110215	15092780	19461015	2	20110215	7
31823004	20110522	15375662	19541222	1	20110522	7
3187086720130904		17879861	19520622	1	20130904	8
3187211420130830		17862535	19630329	1	20130830	8
31883053	20110313	15172744	20010515	1	20110313	7
31890810	20110122	15033140	19470730	1	20110122	10
31917176	20121030	16923198	19440417	1	20121030	7
31981336	20120307	16218942	19630522	1	20120307	7
3199804620130414		17431709	19610203	2	20130414	7
31999607	20111114	15884390	19690426	2	20111114	8
32049186	20120301	16193107	19440805	2	20120301	8
3205937320130501		17482817	19640221	1	20130501	7
32059782	20110905	15682165	19580520	1	20110905	8
3206263820130114		17163537	19710528	2	20130114	7
32105047	20111103	15856150	19550510	1	20111103	10
32116635	20120902	16746873	19720423	2	20120902	8
32198064	20110811	15615283	19370427	2	20110811	10
32206078	20111026	15829242	19600805	1	20111026	8
32212229	20110601	15400749	19510226	1	20110601	8
32216094	20111004	15766271	19581025	1	20111004	10
3221737120130331		17383711	19420401	1	20130331	7
32226952	20120304	16203785	19630812	1	20120304	7
3224782820130216		17250047	19200820	1	20130216	7
32261215	20121109	16960890	19231214	2	20121109	8
32271151	20110311	15170255	19411016	1	20110311	7
32284994	20110916	15716871	19680310	1	20110916	7
32296836	20110208	15066574	19651129	1	20110208	10
3229733920130809		17802407	19291010	1	20130809	7
32332335	20110815	15623382	19380128	1	20110815	8
3234437920131014		18005195	19450910	1	20131014	7
32344517	20110919	15723330	19500316	2	20110919	7
3234611520131011		17999243	19631220	1	20131011	7

32358262	20110312	15171140	19370628	1	20110312	10
32358422	20131106	18081847	19630912	1	20131106	7
32382460	20120812	16690592	19610323	1	20120812	7
32385378	20110624	15471735	19531226	1	20110624	10
32408881	20130303	17296819	19621019	1	20130303	9
32423099	20120317	16248804	19660725	2	20120317	7
32426918	20131029	18050331	19550129	1	20131029	7
32456965	20120902	16746754	19430124	2	20120902	7
32471559	20110620	15459674	19431223	1	20110620	8
32475437	20110422	15290625	19581020	1	20110422	8
32493735	20120903	16750150	19710808	1	20120903	9
32537421	20121004	16847932	19591104	1	20121004	10
32626656	20131022	18032732	19290801	1	20131022	7
32649766	20120827	16733147	19560625	1	20120827	8
32776344	20120406	16306966	19650101	1	20120406	8
32805337	20130609	17605446	19420401	2	20130609	7
32816721	20120127	16092895	19381120	2	20120127	10
32844716	20110728	15570882	19280202	2	20110728	10
32857946	20120706	16581720	19780805	2	20120706	10
32884494	20110927	15744285	19421001	1	20110927	7
32894874	20110320	15192563	19410101	1	20110320	9
32912337	20130826	17850917	19661031	1	20130826	9
32939836	20110907	15692263	19470905	1	20110907	7
32958400	20130408	17411307	19681223	2	20130408	7
32992624	20111203	15940837	19570515	2	20111203	8
32997981	20130108	17145374	19560217	1	20130108	7
33022047	20110502	15314955	19550209	1	20110502	8
33032950	20110919	15722195	19320510	1	20110919	8
33049239	20120104	16036323	19440716	2	20120104	10
33096105	20111207	15956212	19620212	1	20111207	7
33152431	20110830	15664180	19480604	1	20110830	9
33188191	20110624	15470784	19631111	1	20110624	10
33193214	20110920	15726906	19720212	1	20110920	10
33242454	20120524	16448312	19690430	2	20120524	8
33294870	20110327	15210258	19660910	1	20110327	10
33302588	20131109	18094387	19421029	1	20131109	8
33330324	20110301	15134296	19681226	1	20110301	10
33336366	20120706	16581972	19890614	1	20120706	8
33357674	20110502	15314604	19610315	1	20110502	10
33361670	20110509	15339542	19590603	1	20110509	10
33371878	20111020	15815357	19390822	2	20111020	10
33371914	20120208	16133249	19510922	1	20120208	8
33395312	20120625	16539485	19580707	2	20120625	8
33395516	20110509	15339707	19580222	2	20110509	7
33435700	20130725	17752442	19370111	2	20130725	8
33445624	20110601	15401444	19640227	1	20110601	10
33472398	20120527	16454864	19600726	1	20120527	7
33514362	20110828	15659076	19650430	1	20110828	10
33516926	20110221	15110279	19440814	2	20110221	10
33573023	20131001	17962074	19330520	1	20131001	7
33582239	20120409	16313580	19420925	2	20120409	10
33602185	20110903	15678361	19691025	1	20110903	8
33651922	20120516	16427613	19391216	2	20120516	8
33654249	20110913	15705667	19461010	2	20110913	8
33684978	20120121	16087899	19530602	2	20120121	8

33712902	20111001	15756034	19470204	1	20111001	10
3372030820130219		17259137	19611029	2	20130219	9
33728119	20121113	16971772	19641207	1	20121113	8
33743338	20110223	15117059	19530913	1	20110223	9
33784246	20121125	17006550	19610318	2	20121125	7
33795732	20120724	16633638	19701116	1	20120724	7
33816852	20111113	15883442	19540422	1	20111113	7
33828192	20121225	17104416	19790125	2	20121225	7
33830023	20111123	15912205	20010128	1	20111123	8
3387123720130830		17863208	19551102	2	20130830	8
33920966	20120724	16634096	19621119	1	20120724	7
3401482920130624		17649917	19820626	1	20130624	8
34032092	20111214	15976771	19600208	1	20111214	8
34043066	20121022	16902283	19320121	1	20121022	7
34047875	20110623	15469284	19531118	1	20110623	8
34048561	20120714	16606209	19420220	2	20120714	10
34069142	20110322	15199468	19531020	1	20110322	10
3408996820130403		17396091	19700910	1	20130403	8
34106075	20110215	15093200	19450805	2	20110215	9
3411001520130531		17575467	19690514	1	20130531	7
3411188120131117		18118144	19301122	2	20131117	8
34131118	20121011	16870977	19260709	2	20121011	7
3414420220130504		17495271	19530529	1	20130504	8
34162408	20121127	17012833	19580810	1	20121127	7
34173223	20111029	15837433	19390227	2	20111029	7
34181743	20110506	15332382	19480124	1	20110506	10
34223944	20110731	15575021	19291012	1	20110731	8
34237973	20110902	15675118	19360728	1	20110902	7
34276410	20110324	15205774	19560116	1	20110324	10
34278847	20110301	15134395	19600120	2	20110301	10
34291333	20120329	16281112	19490920	2	20120329	8
34305618	20120508	16402497	19760513	1	20120508	8
34363456	20111226	16008312	19180502	2	20111226	8
34413315	20110206	15060245	19550102	2	20110206	9
34430676	20121001	16837005	19510122	1	20121001	10
34477640	20110422	15289498	19970904	1	20110422	10
34488396	20110906	15688107	19640224	1	20110906	8
34495982	20110809	15608989	19530725	1	20110809	8
34499279	20121113	16971887	19490715	1	20121113	8
34504057	20110626	15473930	19620522	1	20110626	9
34520973	20110216	15096469	19680728	1	20110216	10
34524157	20120722	16626863	19600417	1	20120722	8
34578308	20120311	16229588	19621023	1	20120311	7
3459353820130630		17665605	19590319	1	20130630	7
34601586	20120519	16436430	19431031	1	20120519	8
34612674	20120616	16520432	19230906	1	20120616	7
34615902	20110903	15678354	19570110	1	20110903	8
3461610920131218		18219952	19571011	1	20131218	7
34646123	20110113	15009236	19690114	2	20110113	10
3477317620131020		18025755	19980103	2	20131020	7
34792568	20121129	17018525	19270606	1	20121129	7
34794951	20110513	15354008	19620629	1	20110513	7
34822338	20110617	15451994	19261024	1	20110617	8
3484608720131009		17995157	19431014	1	20131009	7
34854621	20120901	16744565	19520623	1	20120901	7

34870343	20110922	15733218	19420123	1	20110922	8
34902959	20110826	15657055	19490215	2	20110826	8
34905721	20110405	15233391	19490125	1	20110405	7
34906882	20110916	15717138	19270801	1	20110916	7
34913661	20111030	15837905	19550925	2	20111030	8
34951285	20111010	15782827	19350819	1	20111010	8
34966604	20110407	15243538	19590212	1	20110407	10
3497449720131209		18188053	19640301	1	20131209	7
34995794	20110731	15575102	19390102	1	20110731	9
35022389	20111118	15901245	19560103	2	20111118	7
3503763920130529		17569574	19390102	1	20130529	7
35038676	20120213	16146502	19701220	1	20120213	8
35039908	20110209	15071523	19501125	2	20110209	9
35041793	20110630	15484310	19500626	1	20110630	8
35075071	20111216	15984941	19620702	1	20111216	7
35112697	20120630	16556362	19430627	2	20120630	9
35119687	20110320	15192475	19560808	1	20110320	10
35167841	20110314	15176794	19430115	1	20110314	10
35169029	20110323	15201678	19311201	1	20110323	9
35210856	20110202	15057359	19771112	1	20110202	10
35210903	20110901	15670764	19721024	2	20110901	7
35238381	20121112	16966519	19781124	2	20121112	7
35245455	20110329	15216378	19600815	1	20110329	10
35279324	20110721	15552888	19570211	1	20110721	8
35285031	20110918	15719190	19690903	1	20110918	8
3528813220130124		17193445	19601012	2	20130124	9
3534556320130103		17126480	19640717	1	20130103	7
35353652	20110328	15210492	19371119	1	20110328	8
35370140	20121109	16960026	19730818	1	20121109	10
35406996	20110408	15247460	19441111	1	20110408	10
35434174	20110613	15439179	19470508	1	20110613	9
35487913	20111010	15782764	19490828	2	20111010	7
35488938	20110905	15683713	19651017	2	20110905	10
35496845	20120214	16150731	19611010	1	20120214	8
35578882	20120325	16268865	19750717	1	20120325	10
35585569	20110403	15228932	19520818	1	20110403	9
35594844	20111122	15908139	19650114	1	20111122	10
35598584	20110208	15064911	19530328	2	20110208	8
35623860	20120623	16537415	19430827	2	20120623	8
35631891	20120326	16271266	19470720	1	20120326	8
3564896520121209		17053374	19590130	1	20121209	8
35679891	20110802	15583742	19460805	1	20110802	10
3569392620130530		17572507	19870604	1	20130530	8
3570293320131105		18076984	19671207	1	20131105	8
3575072220131216		18210237	19300917	1	20131216	7
35751894	20111227	16012424	19880103	1	20111227	7
35772817	20120117	16077484	19610925	1	20120117	8
35807239	20111205	15947513	19540116	1	20111205	10
35843642	20110524	15382219	19520727	1	20110524	10
35867595	20110517	15362492	19460111	1	20110517	9
35926400	20120501	16377573	19681008	2	20120501	9
35926842	20110219	15105865	19841201	1	20110219	8
35931261	20110625	15473349	19560430	1	20110625	9
35935003	20120227	16185593	19411013	2	20120227	8
3597441520131110		18094976	19370920	1	20131110	7

35989969	20120606	16489510	19320530	2	20120606	8
36003675	20120826	16729813	19401118	2	20120826	8
3603519520130123		17191539	19561018	1	20130123	8
36059480	20120123	16088597	19440517	2	20120123	7
36060794	20110204	15058529	19880807	1	20110204	10
36069246	20111204	15943146	19360926	1	20111204	8
36099419	20120229	16190513	19370101	2	20120229	7
3611024420130923		17937191	19301010	1	20130923	8
3611790520130513		17523805	19410720	2	20130513	7
3611964920130222		17272131	19361104	2	20130222	8
36126031	20110207	15062447	19610912	2	20110207	7
36142457	20120602	16474093	19500405	1	20120602	9
36160186	20110531	15396556	19430120	1	20110531	10
3617358720130916		17920909	19530912	1	20130916	7
3619394920131017		18019585	19320102	1	20131017	8
36209231	20120206	16124359	19520706	1	20120206	8
36211344	20110611	15434290	19520124	2	20110611	8
36283097	20110415	15270185	19421209	1	20110415	9
36296943	20110504	15323709	19640314	1	20110504	9
36333150	20110322	15199133	19530612	1	20110322	10
3633511220130401		17390330	19520828	1	20130401	8
36345810	20110605	15414528	19560510	1	20110605	7
36350262	20111227	16012395	19480415	1	20111227	7
36356464	20111230	16019950	19541214	1	20111230	8
36397134	20110625	15472997	19380413	1	20110625	8
3642160020130721		17738076	19660102	1	20130721	8
36453404	20120729	16645506	19591118	2	20120729	9
36488876	20110121	15031847	19500101	1	20110121	7
36514600	20110318	15190497	19680131	1	20110318	7
36530082	20110208	15066606	19500818	2	20110208	9
36530980	20111225	16005635	19280802	1	20111225	8
36555325	20120513	16417502	19380110	1	20120513	7
3656009520131126		18144224	19550215	2	20131126	7
3656235320130915		17917591	19350605	2	20130915	8
36563301	20110820	15640815	19481129	1	20110820	9
36622498	20121105	16942620	19360825	1	20121105	8
36637635	20110602	15407933	19540623	1	20110602	7
3665275220130722		17740979	19540415	2	20130722	7
3665476920130102		17118679	19530329	2	20130102	8
36659957	20120227	16184403	19511202	1	20120227	10
36712557	20110301	15132689	19540713	1	20110301	10
36718931	20110717	15539128	19570322	2	20110717	7
36726860	20110303	15143134	19520824	2	20110303	8
36746506	20110329	15216264	19670102	1	20110329	8
36760244	20120407	16310385	19310303	1	20120407	9
36779585	20110320	15192508	19800201	1	20110320	10
36791738	20120312	16233776	19650105	2	20120312	7
3680242320121214		17074949	19360127	2	20121214	7
36802730	20110312	15172223	19830811	2	20110312	10
36802843	20110201	15056983	19590418	1	20110201	8
3682865620130610		17610365	19411025	1	20130610	7
36842838	20110221	15110065	19960403	2	20110221	8
36868254	20111110	15877770	20031211	2	20111110	10
36876547	20110409	15248370	19500314	1	20110409	8
3688161520130529		17569515	19551225	2	20130529	8

36886972	20110216	15097276	19560806	2	20110216	10
3688970020131119		18124233	19681027	1	20131119	7
36904457	20110408	15247550	19620712	1	20110408	10
36921694	20111009	15782421	19460202	1	20111009	7
36939294	20110625	15473137	19510320	2	20110625	10
36939432	20111115	15890086	19571210	1	20111115	8
36947452	20110726	15564627	19540420	1	20110726	7
36975005	20110222	15112049	19490712	2	20110222	9
36977192	20110623	15468466	19460816	1	20110623	8
36978855	20110208	15066465	19570620	1	20110208	7
36994420	20110216	15097297	19551201	1	20110216	9
36994500	20110122	15033447	19310910	1	20110122	7
36996915	20120625	16540757	19250319	2	20120625	7
37007640	20110302	15139293	19510111	1	20110302	10
3701286520131124		18137988	19580404	1	20131124	7
37013551	20121223	17097572	19431013	1	20121223	10
37016345	20110918	15719041	19621104	1	20110918	8
37020589	20110502	15314810	19661116	1	20110502	8
37021800	20110825	15654321	19800823	2	20110825	8
37022712	20110321	15196310	19511121	2	20110321	7
37033424	20110331	15221802	19470206	1	20110331	9
37040929	20120211	16141792	19361018	1	20120211	8
37052236	20111214	15978183	19640331	2	20111214	10
37052601	20110301	15134371	19331205	1	20110301	10
37057015	20111207	15956571	19240904	1	20111207	10
37079473	20110311	15170508	19751222	1	20110311	10
37086183	20121031	16925969	19680823	2	20121031	8
3709412520131001		17963696	19690529	1	20131001	8
37118040	20110826	15657218	19670406	2	20110826	8
37124008	20110418	15276663	19500518	2	20110418	8
37165370	20110210	15077288	19400615	1	20110210	10
37178533	20121020	16898140	19590508	2	20121020	8
37178544	20120311	16229658	19400628	2	20120311	7
37197710	20110301	15133860	19600602	2	20110301	8
37198417	20110426	15296895	19621123	1	20110426	8
3720148220130111		17155797	19530325	1	20130111	8
37201619	20110619	15455915	19481019	2	20110619	7
3720254320130408		17411480	19391104	2	20130408	8
37207797	20111205	15945748	19500228	1	20111205	7
37221662	20120422	16351688	19490819	1	20120422	9
37237028	20120202	16111972	19360608	1	20120202	8
37268750	20110324	15205888	19940504	1	20110324	8
37272585	20121018	16893852	19810411	1	20121018	10
37272676	20120310	16229278	19361125	2	20120310	7
37273135	20110405	15233058	19451119	1	20110405	8
37273771	20110310	15166868	19340202	2	20110310	10
37278798	20110723	15557504	19690508	1	20110723	10
37297679	20110913	15706654	19590103	1	20110913	7
37309549	20110521	15374538	19471205	1	20110521	7
37312520	20110316	15184202	19390822	2	20110316	7
37313512	20110114	15012585	19550128	2	20110114	7
37317854	20111005	15769625	19350927	1	20111005	10
37325169	20120523	16446156	19410118	2	20120523	8
37328942	20110531	15397590	19800807	2	20110531	7
37340479	20110622	15465156	19540624	1	20110622	9

3734288420130930		17955388	19810222	2	20130930	7
37346284	20110725	15561115	19500828	1	20110725	7
37347221	20111206	15949847	19381120	2	20111206	7
37350133	20110125	15037774	19711222	1	20110125	10
3735770320130709		17703153	19461015	2	20130709	8
37358773	20110720	15550213	19500301	2	20110720	9
37359049	20110907	15689666	19660314	1	20110907	8
37366248	20110110	14998908	19390905	1	20110110	10
37370186	20110902	15676456	19660701	1	20110902	8
37370459	20110411	15254897	19760221	1	20110411	10
37374279	20111109	15874627	19481004	1	20111109	8
37389336	20110501	15309277	19461111	2	20110501	7
37391289	20111220	15992792	19550118	1	20111220	7
37395689	20111130	15930943	19600531	2	20111130	10
37407071	20110304	15147767	19681208	1	20110304	8
37407786	20110216	15096874	19440301	2	20110216	7
37417791	20120529	16460083	19940825	1	20120529	8
37419173	20110317	15187481	19410516	1	20110317	8
37423997	20110131	15053544	19580331	1	20110131	7
37424161	20110314	15176897	19650811	1	20110314	8
37428732	20111201	15936570	19220206	1	20111201	8
37436127	20110127	15047180	19640221	2	20110127	7
37474843	20111205	15947294	19570303	2	20111205	8
37488985	20110802	15585926	19561004	1	20110802	8
37496610	20111127	15921754	19391210	1	20111127	7
37497920	20110618	15455295	19450905	2	20110618	10
37499233	20110921	15728316	19520816	2	20110921	7
37521189	20110315	15180211	19460204	1	20110315	7
37527529	20110224	15121243	19780626	1	20110224	7
37536575	20110630	15485878	19530525	2	20110630	10
37537307	20110321	15196353	19501102	1	20110321	8
37539494	20110207	15061723	19630424	1	20110207	8
37549034	20110217	15098725	19680328	2	20110217	8
37565927	20110803	15590330	19370308	1	20110803	7
37567876	20110323	15202447	19960817	1	20110323	7
37571338	20110615	15446926	19480930	2	20110615	7
37579616	20111119	15902972	19680901	2	20111119	8
37581934	20111225	16005970	19390222	2	20111225	10
37582346	20110810	15611375	19540120	1	20110810	9
3758322520130404		17402579	19200115	1	20130404	8
37584002	20120512	16417114	19360209	2	20120512	7
3758450220130314		17339442	19321224	2	20130314	8
37587501	20110918	15719444	19591012	1	20110918	10
37591201	20110212	15084049	19820927	2	20110212	8
37595043	20120726	16640505	19430802	1	20120726	7
37598020	20120728	16643724	19491019	1	20120728	7
3759825720130927		17950561	19620210	1	20130927	7
37601926	20110608	15424446	19491002	1	20110608	8
37611613	20110518	15367234	19490728	1	20110518	9
3761235420130326		17372674	19620308	1	20130326	7
37633617	20110329	15214561	19640311	1	20110329	7
37636683	20110207	15062260	19550825	1	20110207	10
37646121	20121115	16976919	19630909	2	20121115	9
37663608	20110214	15088683	19521215	1	20110214	10
37673840	20110502	15313506	19331229	1	20110502	10

3767982620130121		17183030	19591231	2	20130121	8
37688792	20111115	15889808	19361021	2	20111115	10
37693495	20111011	15786988	19810813	2	20111011	8
37694614	20120110	16056122	19571212	2	20120110	7
37702242	20120319	16254208	19700114	2	20120319	7
37710079	20110419	15280648	19470414	1	20110419	8
37710331	20110221	15110353	19610803	1	20110221	8
37711765	20110217	15100289	19720901	2	20110217	7
37714480	20110620	15459724	19480529	1	20110620	7
37716704	20120514	16421613	19970310	2	20120514	7
37725169	20110525	15384854	19740930	2	20110525	10
37727278	20111118	15899362	19450701	1	20111118	10
37743423	20110815	15623623	19700202	1	20110815	10
37749238	20110220	15106319	19450521	1	20110220	9
37749352	20120422	16351858	19640707	1	20120422	7
37756164	20110725	15560700	19520908	2	20110725	8
37758648	20110608	15423326	19590602	1	20110608	8
37762906	20110207	15063344	19430618	1	20110207	8
37767194	20120118	16080741	19530127	2	20120118	10
37770517	20120304	16203940	19550305	1	20120304	7
37774360	20110716	15538613	19491012	1	20110716	8
37785878	20120715	16606918	19710317	2	20120715	8
37789461	20111126	15921395	19520114	1	20111126	9
37790399	20120410	16320078	19541005	1	20120410	7
37791552	20110726	15564512	19470902	1	20110726	8
37792066	20110323	15202850	19801028	2	20110323	8
3779268020130925		17945678	19651025	2	20130925	8
37793638	20110320	15192486	19580818	1	20110320	10
37796717	20110308	15157037	19311101	1	20110308	7
3780678320130110		17152863	19620905	2	20130110	8
37813244	20120217	16162404	19270221	1	20120217	7
37819026	20110815	15625744	19591001	2	20110815	8
37821515	20110320	15192416	19450208	1	20110320	8
37823704	20121109	16961148	19590126	1	20121109	7
37823919	20111229	16018453	19860919	1	20111229	10
37832352	20110304	15147226	19421223	1	20110304	7
3783391320130504		17495279	19580417	1	20130504	7
37834029	20110323	15202754	19910130	2	20110323	8
37840894	20110505	15328910	19400126	1	20110505	9
37845480	20110505	15329032	19570426	1	20110505	9
37848945	20110104	14978985	19740823	1	20110104	8
37849153	20110419	15280303	19590208	1	20110419	7
37854992	20111229	16017789	19390912	1	20111229	10
37866890	20110322	15199377	20090129	1	20110322	8
37869797	20110228	15128658	19981102	1	20110228	10
37873420	20110329	15216400	19540103	2	20110329	8
3787957520130416		17440377	19300612	1	20130416	7
37880969	20111226	16006077	19710707	1	20111226	7
37881100	20111213	15974489	19721002	1	20111213	8
37883355	20120624	16537724	19300501	1	20120624	8
37885271	20110208	15066330	19680426	1	20110208	7
37903836	20110121	15030553	19440815	2	20110121	7
37904066	20110620	15459093	19511004	2	20110620	7
37909470	20110329	15215685	19620914	2	20110329	7
37912531	20121112	16967721	19590225	2	20121112	10

37914424	20111001	15755945	19510604	1	20111001	7
37915472	20110505	15328909	19420205	1	20110505	10
37916306	20110522	15375518	19580423	2	20110522	8
37920062	20111012	15791529	19680705	1	20111012	8
37922853	20110324	15205838	19570722	1	20110324	8
37923118	20111115	15889549	19360327	2	20111115	8
37923732	20110317	15187033	19650318	1	20110317	8
37924166	20110317	15186928	19550206	2	20110317	7
37924848	20110220	15106005	19610327	1	20110220	7
37924939	20110226	15125224	19571031	1	20110226	10
37927121	20110916	15716753	19500221	2	20110916	8
37927734	20120617	16520657	19611120	2	20120617	10
37929707	20111027	15833374	19440401	1	20111027	7
37931376	20111113	15883347	19450512	1	20111113	9
37937614	20110316	15184115	19450208	1	20110316	9
37938684	20110207	15063516	19560901	1	20110207	7
37943581	20110316	15180808	19561222	2	20110316	10
37946068	20110525	15385168	19620108	1	20110525	7
37954282	20110322	15198205	19610625	2	20110322	9
37954679	20110824	15651335	19660726	1	20110824	9
37954862	20120204	16119152	19731011	2	20120204	8
37989110	20110424	15292254	19610416	1	20110424	10
37994813	20110423	15291627	19310123	1	20110423	8
3799755020130720		17737800	19530420	2	20130720	8
38000043	20110321	15195869	19340904	2	20110321	8
38002107	20120227	16184925	19590427	1	20120227	10
38002481	20110103	14972430	19640121	1	20110103	7
38007317	20110425	15294240	19671211	2	20110425	8
38008116	20110531	15396470	19340703	1	20110531	7
38013182	20110308	15159011	19810503	1	20110308	8
38013820	20121008	16863080	19990529	2	20121008	10
38018223	20111011	15787161	19301019	1	20111011	9
38022923	20110405	15233901	19630513	1	20110405	8
38024407	20110511	15347768	19541214	1	20110511	8
38031708	20110210	15078049	19570825	2	20110210	8
38033588	20110531	15397802	19610325	2	20110531	8
3804032320130815		17822381	19671231	2	20130815	8
38046730	20110207	15062947	19920219	1	20110207	8
38049568	20110321	15196277	19430218	2	20110321	8
38056676	20110331	15221396	19560906	2	20110331	8
38058616	20110519	15370532	19580210	1	20110519	8
38066761	20110216	15097148	19490901	1	20110216	8
3806735520131222		18228326	20021106	2	20131222	8
38076378	20110724	15557772	19700114	2	20110724	7
38080034	20110524	15382232	19680924	2	20110524	8
38080512	20110512	15350447	19690501	1	20110512	9
38082676	20120903	16751990	19271116	2	20120903	7
38088981	20110310	15166739	19660301	1	20110310	7
38094096	20120810	16686408	19360718	1	20120810	7
38098736	20110227	15125978	19400210	2	20110227	8
38110097	20110823	15648033	19630723	2	20110823	8
38110542	20110405	15233792	19571031	2	20110405	8
38112946	20110627	15477214	19330505	1	20110627	10
38123307	20120227	16185618	19500110	1	20120227	8
38124980	20110317	15187317	20010707	1	20110317	7

38125632	20110508	15335283	19581001	2	20110508	10
38129190	20120628	16552050	19650928	1	20120628	10
38130891	20110409	15249263	19541001	2	20110409	9
38134155	20110607	15419896	19570205	1	20110607	10
38134257	20110810	15611961	19681212	1	20110810	8
38134600	20110918	15719152	19960127	1	20110918	8
38134735	20110719	15545421	19390110	1	20110719	8
38139570	20110301	15134383	19680714	2	20110301	7
38140102	20111010	15782703	19571008	1	20111010	8
38148628	20110324	15203668	19510515	1	20110324	8
38156160	20110404	15230797	19380414	1	20110404	8
38157152	20110210	15075811	19301016	1	20110210	7
38158382	20120516	16427454	19790312	1	20120516	7
38158451	20110423	15291795	19620813	2	20110423	10
38161409	20110529	15392178	19960430	2	20110529	8
38171072	20110324	15205879	19360615	2	20110324	9
38175121	20110321	15196419	19331201	1	20110321	10
38177514	20110405	15233144	19350929	1	20110405	10
38177741	20110723	15557474	19660519	1	20110723	10
3818023320130614		17623078	19520908	2	20130614	8
38188997	20110330	15218986	19421115	2	20110330	10
38194966	20110407	15242612	19651006	1	20110407	7
38196177	20110422	15289699	19490517	1	20110422	7
38197192	20111006	15775969	19920321	1	20111006	8
38212963	20111210	15965331	19240919	2	20111210	10
38215702	20110513	15354473	19580505	1	20110513	7
38219908	20110626	15473844	19590413	1	20110626	10
38223335	20110601	15402487	19480821	1	20110601	9
38232063	20110508	15335267	19480131	2	20110508	10
38239531	20110810	15611091	19330504	2	20110810	7
38245715	20110509	15338429	19551213	1	20110509	8
38246081	20110509	15337276	19320317	1	20110509	8
38246536	20110524	15381283	19650919	1	20110524	9
38247302	20111117	15898204	19490503	1	20111117	8
38247915	20110602	15408006	19690614	1	20110602	10
38248098	20110804	15594837	19821014	1	20110804	7
38250565	20110619	15455756	19510104	2	20110619	9
38254965	20110526	15387767	19520312	1	20110526	8
38257420	20121021	16899186	19600916	1	20121021	8
38258503	20111121	15906999	19540619	1	20111121	8
38262985	20110822	15642914	19540815	1	20110822	7
38264027	20110820	15640812	19391021	1	20110820	8
38271102	20110808	15603583	19620610	1	20110808	10
38271135	20111026	15828232	19560315	1	20111026	8
38271840	20120328	16278370	19400523	2	20120328	7
38272887	20110620	15459654	19451016	1	20110620	10
38273153	20110611	15434100	19860721	2	20110611	7
38292001	20110423	15292035	19500620	1	20110423	8
38300311	20110530	15394677	19560810	1	20110530	8
38300979	20120207	16128925	19611122	2	20120207	7
38303321	20111012	15790137	19600210	2	20111012	7
38304880	20110728	15570256	20090704	2	20110728	8
3831505820130418		17447949	19620916	2	20130418	8
38320331	20110606	15415066	19700514	2	20110606	8
38325767	20111005	15771321	19631012	2	20111005	10

38340088	20110512	15351558	19510727	2	20110512	7
38342971	20110518	15367450	19460918	2	20110518	7
38342993	20110424	15292128	19811213	1	20110424	9
38343021	20110507	15334608	19460527	1	20110507	9
38346519	20110514	15356156	19480825	2	20110514	10
38347987	20110510	15343762	19480710	2	20110510	7
38348355	20110523	15379101	19400118	1	20110523	8
38354211	20110801	15577894	19651010	1	20110801	8
38358304	20110622	15466405	19350806	2	20110622	8
38359738	20120509	16407960	19641117	1	20120509	10
38360086	20110812	15619941	19650220	2	20110812	10
38360097	20110614	15442884	19530122	1	20110614	7
38361807	20110608	15423648	19720831	1	20110608	10
38367009	20110926	15740322	19670320	1	20110926	8
38368820	20110924	15737620	19681001	1	20110924	8
38384097	20110912	15702435	19521125	1	20110912	8
38386695	20111209	15963956	19661002	1	20111209	8
38395027	20110826	15656650	19470520	1	20110826	9
38396724	20110808	15603083	19740703	1	20110808	9
38401955	20110904	15678988	19840613	1	20110904	10
38402152	20110715	15536135	19591030	1	20110715	9
3841110820130603		17583114	19331206	1	20130603	7
38415097	20110821	15641253	19500129	1	20110821	8
3841605620131221		18227734	19500726	1	20131221	8
38416669	20110914	15709950	19600920	1	20110914	8
38421839	20111228	16015646	19460118	2	20111228	7
38425922	20110912	15702315	20040112	1	20110912	10
38435880	20120917	16794711	19391213	1	20120917	8
38451637	20111220	15993909	19580625	1	20111220	8
38475706	20110801	15579768	19620915	2	20110801	10
38487239	20110611	15434120	19641208	1	20110611	7
38490516	20110928	15747649	19330819	1	20110928	10
3849313920130504		17495589	19360907	1	20130504	7
38506911	20110811	15613103	19560525	1	20110811	7
38521221	20110701	15490178	19480929	1	20110701	7
38521356	20110720	15550150	19881209	1	20110720	10
38522428	20110919	15722852	19250913	2	20110919	8
38523909	20111022	15819972	19601206	1	20111022	8
38524695	20110928	15747898	19661216	1	20110928	10
38525574	20120321	16260494	19950828	2	20120321	7
38528506	20120525	16451911	19510802	2	20120525	7
3853383420131021		18027578	19730422	1	20131021	8
38545107	20110827	15658550	19591101	1	20110827	10
38546360	20110816	15628482	19481018	1	20110816	8
38554108	20121008	16863043	19731029	1	20121008	7
38557312	20111004	15764963	19530126	2	20111004	10
38558133	20111127	15921618	19920411	1	20111127	10
38558597	20121029	16920404	19580910	2	20121029	9
38561783	20110901	15672004	19990919	2	20110901	8
38562048	20111008	15781331	19640315	2	20111008	7
38562128	20110915	15713888	19541002	1	20110915	7
38564237	20110914	15709297	19750727	1	20110914	8
38564691	20111003	15759211	19750203	1	20111003	7
38568819	20110815	15625130	19970324	2	20110815	7
38573910	20110830	15662485	19491030	2	20110830	7

38579394	20110919	15723278	19641218	1	20110919	7
38586640	20111012	15789552	19740501	1	20111012	8
38602050	20120411	16322538	19691228	2	20120411	10
3860311120121206		17046268	19641129	2	20121206	7
38605719	20120117	16074856	19691019	2	20120117	9
38621099	20111211	15966425	19240627	1	20111211	7
38623200	20120929	16831275	19350506	2	20120929	8
38626210	20120309	16226111	19510728	2	20120309	7
38626221	20120101	16022619	19240323	1	20120101	7
38627224	20120505	16394503	19771002	1	20120505	8
38631811	20120401	16285468	19540925	1	20120401	8
38637171	20120201	16107089	19351230	1	20120201	8
38639371	20120328	16276583	19480816	1	20120328	8
38642067	20121210	17057325	19271105	1	20121210	7
38642396	20110920	15726966	19531219	1	20110920	10
38642772	20110906	15686361	19740905	2	20110906	10
38643106	20111105	15862038	19520228	2	20111105	8
38650203	20111031	15839986	19601010	1	20111031	10
38651400	20111206	15951515	19420725	1	20111206	10
38651615	20111005	15768706	19380904	2	20111005	8
38651988	20111120	15903501	19400402	1	20111120	8
38652527	20111011	15786510	19360626	1	20111011	8
38656198	20120129	16094661	19600314	2	20120129	7
38656870	20111214	15978139	19660808	2	20111214	10
38657271	20110922	15733046	19581228	1	20110922	8
38658365	20110915	15711901	19530901	1	20110915	7
3865936820130807		17796030	19400104	1	20130807	8
38659482	20111030	15837708	20071214	2	20111030	7
38660525	20120414	16333183	19440923	1	20120414	8
38660912	20120430	16372562	19800718	2	20120430	9
38661120	20120127	16092961	19370502	2	20120127	9
38663477	20120311	16229714	19501206	1	20120311	7
38669011	20111126	15921372	19320730	1	20111126	8
38671760	20120523	16447050	19721207	1	20120523	7
38673120	20111103	15855803	19960524	1	20111103	9
38673539	20120801	16656578	19460309	1	20120801	7
3867678720130323		17365579	19800921	2	20130323	8
38677768	20111001	15756153	19691010	1	20111001	8
38678169	20110915	15712668	19510910	1	20110915	8
38684569	20111013	15795113	19511212	2	20111013	8
38687819	20111021	15817154	19530202	2	20111021	10
38693060	20111025	15826008	19600429	2	20111025	7
38695000	20110918	15719436	19600730	2	20110918	8
38695953	20111205	15947784	19570606	1	20111205	7
38697200	20110926	15741260	19550930	1	20110926	10
38700220	20111221	15996500	19460331	1	20111221	10
3870092420121119		16987901	19951104	1	20121119	7
38703650	20111112	15882855	19740416	1	20111112	7
38706342	20111107	15866573	19531110	1	20111107	8
38707469	20111023	15820316	19481012	1	20111023	8
38709954	20111222	16000356	19420113	1	20111222	9
38721061	20111023	15820562	19880707	1	20111023	9
38726135	20111109	15874254	19390102	2	20111109	7
38727467	20111230	16020243	19701025	1	20111230	8
3873578320131203		18168159	19780326	2	20131203	7

38737734	20111109	15873182	19181205	1	20111109	8
38738259	20111107	15865146	19750306	2	20111107	8
38747932	20111111	15880960	19530320	2	20111111	10
38750173	20120104	16037295	19530123	1	20120104	7
38750537	20111030	15837861	19591031	1	20111030	8
38760713	20111113	15883273	19640529	2	20111113	7
38760928	20120812	16690526	19320126	2	20120812	10
38762297	20111221	15997956	19620315	1	20111221	7
38763369	20111219	15989147	19620408	1	20111219	7
38767838	20111117	15897013	19610520	1	20111117	7
38769221	20120604	16478121	19471120	2	20120604	7
38772779	20111205	15948185	19820627	2	20111205	8
38773603	20120130	16096656	19560512	1	20120130	8
38777116	20111122	15910473	19750918	2	20111122	10
3877838020121231		17115572	19550205	1	20121231	8
38783630	20111101	15843536	19690823	1	20111101	10
38785090	20111111	15879064	19430709	2	20111111	8
38789401	20111206	15950135	19671123	2	20111206	8
38789605	20111124	15915559	19561031	1	20111124	10
38809619	20120915	16794059	19760906	1	20120915	8
38810332	20111121	15904545	19450624	2	20111121	8
38824305	20120214	16150762	19411003	1	20120214	7
38829979	20121119	16990113	19371118	2	20121119	10
38835460	20120110	16054665	19490415	2	20120110	10
38835755	20120404	16299548	19630209	2	20120404	7
38839531	20120420	16349857	19500810	1	20120420	8
38841371	20120428	16369035	19440301	2	20120428	8
38848576	20120601	16471917	19500713	1	20120601	10
38851397	20111128	15924024	19990701	2	20111128	7
38852221	20111204	15943102	19620722	1	20111204	10
38855606	20120315	16245031	19431008	2	20120315	7
38866578	20111214	15977648	19550303	2	20111214	7
3886691020130506		17499432	19550605	1	20130506	8
38867275	20111219	15990515	19301208	1	20111219	8
38871920	20120209	16136161	19620428	2	20120209	10
38874156	20120213	16147876	19580902	1	20120213	10
38879468	20120206	16121040	19410419	1	20120206	10
38880772	20120130	16098065	19650202	1	20120130	10
3888496720131001		17964459	19711015	1	20131001	8
38885186	20111218	15986935	19611126	2	20111218	7
38886316	20111218	15987173	19371011	1	20111218	8
38886918	20121014	16878158	19330215	2	20121014	8
38888027	20120403	16296626	19360128	2	20120403	8
38888356	20121027	16915898	19650405	2	20121027	8
3888962220121226		17106752	19560902	2	20121226	7
38891202	20120412	16326841	19591010	1	20120412	10
38892852	20120123	16088767	19620211	1	20120123	9
38896241	20120104	16036372	19360426	1	20120104	8
38897471	20120622	16535817	19530816	2	20120622	8
38908257	20120131	16101355	19400525	1	20120131	8
38910279	20120404	16299655	19540115	1	20120404	7
38910804	20120201	16107920	19600226	1	20120201	7
38913609	20120206	16123904	19750414	1	20120206	7
3891483920130113		17159759	19480812	1	20130113	7
38915014	20120122	16088240	19810113	2	20120122	10

38916904	20120619	16527864	19601226	2	20120619	7
38920580	20120606	16489373	19380530	2	20120606	7
38925892	20120526	16453570	20030627	2	20120526	10
38929918	20120317	16249409	19530425	1	20120317	8
38930697	20121118	16985925	19810806	1	20121118	7
38939832	20120202	16111806	19641114	1	20120202	8
38945652	20120617	16520749	19560102	1	20120617	8
38948355	20120221	16171764	19770209	2	20120221	7
3895897320130522		17551773	19490528	1	20130522	8
38959636	20120511	16415492	19661119	2	20120511	8
38974651	20120309	16227184	19530710	1	20120309	7
38979394	20120405	16304224	19730827	1	20120405	7
38979690	20120603	16474594	19620429	1	20120603	7
38986968	20120414	16332461	19400608	1	20120414	8
38987256	20120701	16556981	19411007	2	20120701	7
38989229	20120417	16340405	19811225	2	20120417	9
38989923	20120305	16208003	19671008	2	20120305	7
38994739	20120328	16275691	19640510	1	20120328	8
38995823	20120908	16772662	19330811	1	20120908	7
38997681	20120624	16538207	19360810	1	20120624	7
38999847	20120601	16471710	19501125	1	20120601	7
39010338	20120922	16813949	19650804	1	20120922	7
39012196	20120514	16420189	19850713	2	20120514	7
39014385	20120812	16690470	19410403	2	20120812	10
39016245	20121203	17029840	19450901	1	20121203	7
39016405	20120403	16296500	19900523	1	20120403	8
39018854	20120305	16209064	19510816	2	20120305	9
39037837	20120422	16351835	19640301	2	20120422	7
39038512	20120418	16343293	19481103	1	20120418	8
39040216	20120320	16257085	19311124	1	20120320	9
39049177	20120426	16364256	19640212	1	20120426	8
39050265	20120610	16500229	19780804	1	20120610	7
39055931	20120417	16340513	19500612	2	20120417	7
39065264	20120429	16369741	19540526	1	20120429	8
39069846	20120403	16296443	19670917	2	20120403	8
39072601	20120618	16524773	19711206	1	20120618	7
39076647	20120411	16323638	19630811	1	20120411	7
39077902	20120711	16597140	19401115	1	20120711	8
39088498	20120824	16727839	19440715	1	20120824	8
39092347	20120508	16404163	19510821	2	20120508	7
39097137	20120517	16431259	19960620	1	20120517	9
39097502	20120523	16446744	19860130	1	20120523	7
39098210	20120425	16362002	19510205	1	20120425	7
39102528	20120605	16483457	19570411	2	20120605	8
39106053	20120913	16788298	19490523	2	20120913	8
39108479	20120524	16450081	19770305	2	20120524	7
39117516	20120723	16629703	20000711	1	20120723	8
39117561	20120420	16349767	19651013	2	20120420	7
39125865	20120804	16662532	19290614	1	20120804	10
39130319	20120514	16420281	19381215	1	20120514	10
39130671	20120502	16380583	19541110	1	20120502	10
39133136	20120424	16358879	19371108	1	20120424	8
39133669	20120510	16412000	19770828	1	20120510	10
39139463	20120615	16518145	19491210	1	20120615	7
39141930	20121011	16871061	19641013	2	20121011	7

39150771	20120611	16504573	19830420	2	20120611	10
39155561	20120507	16399582	19571102	1	20120507	8
39155823	20120728	16644958	19480809	1	20120728	7
39156019	20120629	16554255	19490128	1	20120629	7
3915754520130414		17431595	19560504	2	20130414	9
39162793	20120608	16497640	19560719	1	20120608	9
39165849	20120529	16459871	19630911	2	20120529	10
3917480620130202		17217828	19341125	1	20130202	8
3918112920130129		17205672	19751226	2	20130129	8
39182439	20120508	16402998	19440417	1	20120508	7
39186000	20120516	16428430	19490210	1	20120516	7
3918813120130221		17268194	19440118	1	20130221	7
39193925	20120702	16560573	19500730	2	20120702	7
39201451	20120527	16454547	19740109	2	20120527	8
39203957	20121029	16920090	19540808	2	20121029	8
39204018	20120705	16577856	19441020	2	20120705	10
39209171	20120715	16606649	19191102	2	20120715	7
39209784	20120710	16591581	19400628	2	20120710	7
3921212920130702		17675040	19660402	2	20130702	7
39214089	20120606	16489155	19610503	1	20120606	7
39223966	20120817	16709361	19320603	1	20120817	7
39224629	20120601	16471005	19610217	2	20120601	10
39232923	20120928	16829522	19590325	1	20120928	7
39252534	20120905	16761728	19520703	2	20120905	7
39255146	20120902	16746847	19530210	2	20120902	10
39266416	20120628	16551859	19551010	1	20120628	7
39278256	20120711	16595716	19570701	1	20120711	10
3927959920130312		17332577	19410103	1	20130312	8
39281000	20120722	16627062	19791125	2	20120722	10
39282230	20120728	16644985	19810426	1	20120728	8
39288034	20120723	16629908	19700817	1	20120723	7
39301569	20120723	16630670	19750721	1	20120723	8
39306531	20120710	16591816	19390702	1	20120710	7
3930733020130910		17902998	19471028	1	20130910	8
39313070	20120811	16690021	19550102	2	20120811	9
39323687	20121014	16878179	19650302	1	20121014	10
39334173	20120930	16831728	19560305	1	20120930	9
3933780920121226		17107499	19471214	1	20121226	7
39366206	20120727	16641954	19560914	1	20120727	10
39374022	20120923	16814302	19590109	2	20120923	7
39374986	20120918	16801892	19500212	1	20120918	7
39385234	20120903	16751201	19540117	1	20120903	7
39390415	20121006	16857707	19641125	2	20121006	7
39391178	20121005	16855735	19380420	2	20121005	7
3939360720130106		17135332	19790428	1	20130106	8
39402589	20121118	16985919	19701117	1	20121118	8
39403628	20120826	16729796	19640523	1	20120826	7
3940870320130820		17836917	19551007	2	20130820	7
39411262	20120819	16711486	19620128	1	20120819	7
39415377	20120910	16777361	19691122	2	20120910	10
39417895	20120924	16815937	19210415	1	20120924	7
39419562	20120829	16738093	19370903	1	20120829	8
39419619	20121016	16885860	19370519	2	20121016	10
39420898	20120807	16673887	19771109	2	20120807	7
3943019820130410		17421206	19520112	1	20130410	9

39455271	20121017	16890139	19500702	1	20121017	10
39486265	20121217	17081596	19540819	1	20121217	7
39498323	20121031	16926100	19560313	2	20121031	10
39511814	20120930	16831831	19661207	2	20120930	8
39514711	20121116	16982770	19680923	2	20121116	9
39516466	20121030	16922451	19480220	1	20121030	10
39518871	20121121	16997843	19530520	1	20121121	9
39519852	20121109	16960682	19520612	1	20121109	7
39520586	20121012	16875453	19460529	2	20121012	8
39523198	20131015	18010938	19520614	1	20131015	9
39525047	20121112	16967810	19630726	1	20121112	7
39529538	20130128	17202977	19500513	1	20130128	8
39529721	20130218	17255469	19621107	1	20130218	7
39532075	20121011	16871921	19550715	1	20121011	10
39532213	20121118	16986076	19450423	1	20121118	10
39541305	20121023	16906318	19970728	1	20121023	7
39542762	20130525	17559473	19690528	2	20130525	8
39545965	20121023	16905842	19560827	1	20121023	8
39546651	20121123	17004255	19261118	1	20121123	7
39550635	20130119	17180144	19210410	1	20130119	8
39550646	20120930	16831849	19700730	1	20120930	9
39550942	20121012	16875922	19470715	1	20121012	7
39556917	20130930	17957331	19290103	1	20130930	7
39566091	20121009	16865379	19580228	1	20121009	7
39574000	20130724	17749266	19570130	1	20130724	8
39580966	20121018	16890422	19370202	1	20121018	7
39581083	20121121	16997695	19400114	2	20121121	7
39582906	20121101	16930231	19730120	1	20121101	7
39587729	20121031	16925344	19600704	1	20121031	9
39590039	20130217	17250317	19490416	1	20130217	7
39590540	20121007	16858323	19390402	2	20121007	7
39590777	20130707	17693564	19490514	1	20130707	7
39593878	20121029	16919639	19560225	1	20121029	7
39596800	20130103	17128524	20060908	1	20130103	7
39597825	20130313	17335807	19910529	2	20130313	8
39607697	20121024	16909324	19380317	2	20121024	7
39612209	20121024	16909561	19510316	1	20121024	10
39623035	20121104	16938458	19730927	1	20121104	8
39635728	20130127	17200217	19600721	1	20130127	7
39638341	20121220	17091927	19690130	1	20121220	10
39638807	20121120	16992387	19390926	1	20121120	8
39645039	20121222	17097110	19611119	2	20121222	9
39645788	20130306	17310804	19520109	2	20130306	8
39645835	20121202	17024898	19521028	1	20121202	7
39647557	20130307	17317530	19470301	1	20130307	7
39648823	20131204	18173633	19670522	2	20131204	8
39653899	20130101	17117789	19541120	1	20130101	8
39655055	20121223	17097622	19501101	1	20121223	9
39662958	20130214	17246101	20060224	2	20130214	8
39670116	20131108	18092556	19391013	2	20131108	7
39682150	20130126	17199433	19800727	1	20130126	8
39682592	20130104	17131154	19600910	2	20130104	8
39684907	20130101	17117611	19560204	2	20130101	7
39698107	20130304	17302487	19470401	1	20130304	9
39698572	20130316	17345122	19530218	2	20130316	8

3971370720130619	17636827	19731030	2	20130619	8
3971596320131119	18125827	19410117	1	20131119	8
3974071120130418	17446981	19470111	1	20130418	8
3974678620130418	17447687	19490107	2	20130418	9
3979336720130412	17428170	19490925	1	20130412	7
3980548620130120	17180703	19621128	1	20130120	7
3981200520130106	17135519	19631027	2	20130106	8
3982010520130129	17205204	19420315	1	20130129	7
3982422120130224	17274040	19590611	1	20130224	8
3984629220130214	17245759	19370606	1	20130214	8
3987518820130218	17253596	19351101	1	20130218	8
3988389120130218	17253782	19740706	1	20130218	7
3990234620130303	17296612	19481112	1	20130303	9
3990261920130315	17341204	19510311	2	20130315	8
3990630420130305	17307879	19940702	2	20130305	7
3990819520130822	17840460	19270217	1	20130822	7
3991841720130218	17252560	19950322	2	20130218	8
3992211720130324	17366141	19890112	2	20130324	8
3993103820130401	17386560	19641201	1	20130401	8
3993391020131013	18003379	19360911	2	20131013	8
3996963620130911	17907439	19580820	2	20130911	7
3997909420130401	17387987	19590827	1	20130401	8
3998291720130321	17359557	19631025	2	20130321	7
3999739220130327	17376171	19520915	2	20130327	7
4000826220130318	17348295	19570225	1	20130318	8
4001203120130428	17471979	19640508	1	20130428	9
4001247320130419	17450939	19830720	2	20130419	8
4001292820130802	17777483	19671203	1	20130802	8
4001554120130321	17360570	19660820	2	20130321	9
4001779620130514	17526365	19521108	1	20130514	9
4001886820130327	17375266	19590302	2	20130327	7
4002600420130325	17369315	19440715	1	20130325	7
4003521020130614	17622306	19350226	1	20130614	9
4004653520130806	17788838	19700425	2	20130806	9
4005513820130519	17541266	19520401	2	20130519	7
4005566120131124	18138042	19490501	2	20131124	8
4005586520130411	17425358	19730112	1	20130411	7
4005708720130519	17541172	19570803	1	20130519	8
4006068220130421	17453201	19340110	1	20130421	8
4006278020130612	17615178	19401229	1	20130612	8
4006358920130619	17637765	19640718	1	20130619	9
4006549420130702	17674849	19641029	2	20130702	7
4007321020130707	17693883	19320119	1	20130707	7
4008504920130716	17724173	19340113	2	20130716	8
4009022020130519	17541132	19850329	1	20130519	8
4015205420130616	17625282	19541225	1	20130616	8
4017136620130508	17507426	19570613	1	20130508	8
4017391920130612	17615102	19480617	1	20130612	8
4017598220130809	17802997	19540328	1	20130809	7
4017801620130526	17560392	19850301	2	20130526	9
4018935320130905	17886790	19591201	1	20130905	8
4018947720130624	17650453	19650112	2	20130624	7
4019674520130725	17752740	19900406	2	20130725	7
4019731720130823	17845182	19661018	2	20130823	7
4020691520130917	17925800	19560510	1	20130917	8

4020773620130603	17583267	19490402	1	20130603	7
4021816420130704	17687123	19930730	2	20130704	7
4022487120130610	17608834	19741026	1	20130610	7
4023854820130607	17602478	19610817	2	20130607	8
4023891320130723	17743975	19480101	1	20130723	7
4024060620130610	17608903	19510927	1	20130610	7
4024811120130922	17933770	19540131	2	20130922	8
4024815520130923	17937266	19170218	1	20130923	8
4024955620131127	18147398	19391024	1	20131127	7
4025694820130823	17844432	19610324	2	20130823	7
4026688420131226	18241222	19490515	1	20131226	7
4026888020131127	18147522	19390502	2	20131127	7
4026938320130905	17884880	19570210	1	20130905	7
4027637720130707	17693579	19530905	1	20130707	8
4028499920131111	18095235	19541231	2	20131111	7
4029052620130801	17773046	19670707	1	20130801	7
4029240820130904	17881198	19470703	2	20130904	9
4029351420130929	17954584	19550122	1	20130929	8
4029560120131125	18141767	19341118	1	20131125	7
4029660420131102	18066281	19360112	1	20131102	8
4029912520130813	17813274	19901001	1	20130813	9
4030508620130716	17723831	19571010	1	20130716	8
4030794620130708	17698622	19601204	1	20130708	9
4031251420130717	17728439	19830120	1	20130717	9
4039710220130815	17821820	19400921	2	20130815	9
4040622220130728	17757867	19401110	1	20130728	9
4042727820130821	17838506	19560629	1	20130821	8
4042841920130905	17887351	19930917	2	20130905	8
4043305420130902	17868949	19611016	1	20130902	8
4043784120131218	18219991	19760913	1	20131218	7
4044168720130810	17806176	19770225	2	20130810	8
4044446020130903	17872461	19781101	2	20130903	7
4045111420130806	17790184	19881018	1	20130806	8
4045577420131017	18020221	19691207	1	20131017	8
4046226820130906	17891420	19711112	1	20130906	8
4046386320130910	17903083	19570225	2	20130910	7
4046527820130827	17852713	19580817	1	20130827	9
4046579020130827	17853241	19541206	1	20130827	9
4048159220131028	18048904	19621022	2	20131028	7
4048276620131001	17959228	19281015	1	20131001	8
4048561820131127	18148475	19671220	1	20131127	7
4050115320130910	17901905	19671020	1	20130910	8
4050181320130928	17954370	19691010	2	20130928	7
4050713920130901	17864899	19701107	1	20130901	8
4053058320131014	18006197	19420928	1	20131014	7
4061114120131127	18148377	19580410	2	20131127	8
4061341020131103	18066846	19570620	1	20131103	7
4062531820131105	18072722	19240410	1	20131105	8
4062694620131107	18087301	19680821	2	20131107	7
4063116120131102	18064610	19531202	2	20131102	7
4063589020131113	18108868	19600921	1	20131113	9
4064046820131013	18003053	19551214	1	20131013	8
4065572920131221	18227912	19540115	1	20131221	8
4066506320131204	18171115	19480425	2	20131204	7
4067090420131222	18228064	19740211	2	20131222	9

4067133820131112	18100691	19720526	1	20131112	7
4067555620131029	18051368	19520801	2	20131029	7
4067603720131020	18025900	19480312	2	20131020	7
4069172320131024	18040509	19301211	1	20131024	8
4076663020131203	18167454	19590124	2	20131203	7
4086365820131205	18178225	19590326	1	20131205	7
53543 20120528	16457665	19271112	1	20120528	8
232860 20120208	16131817	19281204	1	20120208	9
360827 20120818	16711275	19130218	1	20120818	7
413898 20120527	16454781	19240713	1	20120527	10
470984 20121008	16861359	19300108	1	20121008	9
48621520130327	17375215	19300924	1	20130327	8
55706220130107	17140621	19210321	1	20130107	8
738738 20110925	15738253	19310303	1	20110925	8
780267 20110313	15172508	19400422	2	20110313	8
1048517 20121117	16984796	19370709	2	20121117	7
105495120130210	17242393	19200303	2	20130210	7
1166923 20120630	16556056	19341115	2	20120630	8
159008520130212	17243476	19660723	2	20130212	7
1597213 20121016	16884581	19410717	1	20121016	7
162023120130523	17553839	19230815	1	20130523	8
1669405 20110827	15658643	19210920	1	20110827	7
169861920130203	17220309	19231030	2	20130203	8
182235120130724	17747923	19330919	1	20130724	7
1849429 20121124	17006086	19410721	2	20121124	7
2307837 20111116	15894834	19480612	2	20111116	9
244155620130224	17274181	19430415	2	20130224	8
244203720131009	17996805	19210712	1	20131009	9
2573184 20120926	16823832	19351011	1	20120926	7
2624353 20120601	16470824	19300222	2	20120601	7
2832759 20120926	16824708	19250310	1	20120926	8
285597220130124	17195040	19260529	1	20130124	7
304393420130310	17324165	19270219	1	20130310	8
3316469 20120430	16372393	19520827	2	20120430	7
332357720130305	17305295	19460615	1	20130305	8
3370794 20110815	15625638	19511010	2	20110815	7
341237120130313	17336354	19290613	2	20130313	8
352278920131001	17964510	19400806	2	20131001	8
3783742 20110506	15332178	19420522	2	20110506	8
3853869 20120223	16177043	19210505	1	20120223	8
3854668 20110525	15385286	19280901	1	20110525	10
3921537 20120516	16427657	19690801	2	20120516	7
4014055 20110927	15745058	19520916	2	20110927	10
4074684 20110909	15698711	19410314	2	20110909	8
4164130 20120308	16222692	19231230	1	20120308	8
4233425 20111012	15791522	19460508	2	20111012	7
4596707 20120512	16416043	19310608	1	20120512	8
476421020130127	17200075	19430428	1	20130127	7
4812117 20110411	15253618	19460323	1	20110411	9
4861487 20110512	15351644	19260528	1	20110512	8
498227820130108	17145284	19370112	1	20130108	9
4991906 20121111	16963376	19401224	1	20121111	9
5065441 20120724	16634110	19550103	2	20120724	10
5143919 20110422	15290718	19371113	2	20110422	8
5337199 20110502	15311995	19311228	1	20110502	8

5842795	20120529	16458959	19330306	1	20120529	7
5984725	20131103	18066462	19270207	1	20131103	7
6016575	20120707	16584006	19250123	1	20120707	8
6053992	20130102	17123259	19610621	1	20130102	8
6135699	20130915	17917607	19550304	1	20130915	8
6206515	20131120	18128239	19290606	1	20131120	8
6269836	20131204	18170088	19320101	2	20131204	8
6285489	20130821	17838841	19311108	2	20130821	7
6355926	20131224	18235060	19240405	2	20131224	8
6409887	20110509	15337495	19490415	1	20110509	10
6416462	20110516	15359253	19290806	1	20110516	7
6682366	20130121	17184045	19500510	1	20130121	7
7081972	20130418	17447857	19250715	1	20130418	8
7202940	20121226	17107095	19531211	1	20121226	7
7444760	20120604	16474932	19550101	1	20120604	8
7525362	20130722	17741350	19780906	1	20130722	7
7736858	20110701	15490546	19420116	2	20110701	9
7852713	20120523	16446981	19250510	1	20120523	10
7930418	20120220	16167069	19560709	2	20120220	7
7966929	20120516	16428104	19340228	2	20120516	7
8180232	20131017	18020029	19401203	1	20131017	7
8331677	20130207	17238230	19281003	1	20130207	7
8354129	20120615	16517433	19390830	2	20120615	7
8513880	20120314	16239960	19471130	1	20120314	7
8653483	20121024	16909335	19301001	1	20121024	7
8718669	20131115	18115565	19411001	2	20131115	9
9683209	20120805	16665026	19330825	1	20120805	7
9790141	20120129	16094463	19320913	2	20120129	7
9922252	20110511	15347785	19611027	1	20110511	8
10126064	20120820	16715503	19550813	1	20120820	10
10193938	20121109	16960941	19291005	1	20121109	10
10234261	20111106	15862453	19511011	1	20111106	7
10341001	20130825	17847432	19501118	1	20130825	8
10411742	20111102	15851772	19450215	2	20111102	8
10430565	20110413	15262542	19530217	2	20110413	10
10434727	20120721	16626471	19441231	1	20120721	7
10678850	20110424	15292283	19290202	1	20110424	7
10684272	20110602	15408096	19471102	1	20110602	10
10917109	20110425	15296044	19550509	1	20110425	8
10939818	20120504	16391336	19431225	1	20120504	9
11008381	20120929	16831129	19300603	1	20120929	7
11018998	20110725	15560890	19490610	2	20110725	7
11045117	20111216	15983116	19430211	1	20111216	8
11045833	20120522	16443861	19420301	1	20120522	8
11050127	20120911	16781441	19561031	2	20120911	9
11140822	20110804	15594814	19290505	1	20110804	10
11169792	20111028	15835540	19480915	1	20111028	8
11330839	20120102	16027579	19310912	2	20120102	7
11351181	20111123	15913549	19460628	1	20111123	8
11412923	20120809	16683670	19390513	2	20120809	8
11779387	20130630	17665906	19810226	2	20130630	9
11842221	20110527	15389529	19530720	2	20110527	8
11950451	20120416	16335883	19630812	2	20120416	8
11972115	20110706	15506439	19530610	1	20110706	8
12105598	20120209	16137180	19421113	1	20120209	7

12110097	20120103	16032377	19510623	2	20120103	9
12207511	20120314	16241256	19470608	2	20120314	10
12270949	20130531	17575701	19510401	1	20130531	8
12473766	20131210	18194575	19661221	1	20131210	7
12475159	20110417	15272835	19600712	2	20110417	8
12494596	20111114	15886577	19490720	2	20111114	7
12690312	20111022	15819865	19560826	1	20111022	8
12865962	20130501	17484131	19261021	1	20130501	7
12931247	20110613	15436810	19331010	1	20110613	9
13022510	20130519	17541090	19340825	2	20130519	8
13069186	20121216	17077487	19760531	2	20121216	9
13188288	20131018	18023043	19260518	1	20131018	7
13204530	20110523	15378856	19560622	1	20110523	8
13285524	20120117	16077314	19291201	1	20120117	10
13345681	20110830	15664322	19690114	1	20110830	7
13571749	20120510	16412058	19511112	2	20120510	7
13770815	20120301	16193582	19570825	2	20120301	10
13802498	20130410	17420302	19330114	1	20130410	8
13823659	20120608	16496669	19350505	2	20120608	10
14107052	20130603	17584192	19481107	1	20130603	7
14447177	20110930	15753560	19520813	1	20110930	7
14453248	20111212	15970645	19320202	1	20111212	10
14495911	20120219	16164365	19560912	1	20120219	10
14592166	20110330	15218449	19270824	1	20110330	7
14842581	20110714	15533422	19570627	1	20110714	10
14927652	20110520	15373540	19470515	1	20110520	8
15184835	20131227	18242153	19260712	1	20131227	8
15246885	20130523	17554990	19840209	2	20130523	8
15254214	20120215	16155458	19310404	1	20120215	10
15370033	20120622	16536976	19270512	1	20120622	8
15371003	20110902	15675990	19350225	2	20110902	10
15476070	20121202	17025188	19370523	2	20121202	7
15510835	20110619	15455904	19440126	2	20110619	9
15575470	20120406	16308454	19520713	1	20120406	7
15661899	20120315	16244358	19330122	1	20120315	8
15759383	20120430	16371952	19680929	1	20120430	10
15759598	20120930	16831756	19510517	1	20120930	9
15761587	20110812	15619352	19640602	2	20110812	7
15827471	20110205	15059391	19600201	2	20110205	8
15975232	20120221	16170869	19550623	1	20120221	10
16002736	20120111	16059228	19490310	1	20120111	7
16140811	20131215	18208411	19541024	2	20131215	7
16162359	20120216	16157249	19551003	2	20120216	10
16234778	20120518	16434929	19350125	1	20120518	7
16257344	20130217	17250553	19250620	1	20130217	7
16258325	20131023	18034564	19301110	1	20131023	9
16272881	20110921	15729729	19460707	2	20110921	8
16279688	20120311	16229833	19250202	1	20120311	10
16394459	20120319	16253885	19440808	2	20120319	7
16455495	20120822	16721689	19300328	1	20120822	8
16579434	20120222	16175081	19410907	2	20120222	9
16605020	20120828	16736297	19610330	1	20120828	9
16766500	20110409	15249347	19390510	1	20110409	8
16906868	20110927	15744841	19380701	1	20110927	10
16974666	20131016	18015184	19530915	1	20131016	8

17031946	20130522	17551720	19650120	1	20130522	8
17049682	20110818	15636350	19740813	1	20110818	7
17049955	20110724	15558080	19591008	2	20110724	10
17097722	20120615	16518705	19320329	1	20120615	8
17198004	20120906	16766799	19440418	1	20120906	10
17244143	20110714	15532054	19400101	2	20110714	10
17286327	20111019	15810445	19340930	2	20111019	8
17349267	20110413	15263598	19361009	1	20110413	7
17517043	20110221	15109545	19680730	2	20110221	10
17549223	20120320	16256451	19530701	1	20120320	8
17720682	20130628	17663261	19490121	1	20130628	8
17771992	20120219	16164530	19400515	2	20120219	7
17827315	20120927	16825980	19550306	1	20120927	10
17857555	20110405	15233221	19630420	1	20110405	10
17927821	20131016	18016092	19220524	2	20131016	8
18023217	20131004	17978657	19300323	1	20131004	9
18039913	20130329	17381240	19211224	1	20130329	8
18199398	20130313	17336485	19370219	2	20130313	8
18462867	20130328	17379412	19570907	1	20130328	7
18590722	20110524	15382259	19410228	2	20110524	8
18601622	20120715	16606724	19390311	1	20120715	7
18842303	20131102	18066128	19610115	2	20131102	7
19250534	20120210	16140701	19300429	1	20120210	7
19311558	20110614	15441007	19670608	2	20110614	10
19328531	20120402	16291739	19270808	1	20120402	7
19439986	20120216	16159136	19501212	1	20120216	10
19573945	20130722	17741729	19581213	2	20130722	8
19635893	20110819	15639161	19330113	2	20110819	8
19889328	20130329	17379528	19490218	1	20130329	8
19920764	20110613	15439152	19470201	1	20110613	8
20025574	20130121	17182850	19640312	2	20130121	7
20060382	20120629	16554935	19330518	2	20120629	7
20108025	20121207	17049323	19191110	1	20121207	7
20137079	20110930	15753565	19340117	2	20110930	8
20216084	20110316	15183474	19560809	2	20110316	7
20245914	20120804	16664524	19600728	1	20120804	8
20284666	20111120	15903425	19331210	1	20111120	7
20433312	20121111	16963358	19260909	1	20121111	10
20634780	20131211	18196332	19290508	1	20131211	8
20657298	20121226	17107402	19660928	2	20121226	8
20719260	20131217	18216031	19520427	1	20131217	7
20828879	20120803	16658902	19391205	1	20120803	8
20859056	20130205	17230708	19351109	2	20130205	8
20969942	20131229	18245840	19280108	1	20131229	7
21004988	20111020	15815383	19691003	2	20111020	7
21006804	20110402	15228517	19870721	1	20110402	8
21105351	20131021	18026667	19510910	1	20131021	8
21377026	20121223	17097616	19550910	1	20121223	9
21422924	20131002	17968866	19681017	1	20131002	8
21743680	20120203	16117196	19550712	2	20120203	10
21791037	20120629	16554803	19261009	1	20120629	8
21824055	20120809	16683716	19520920	1	20120809	7
21912572	20111016	15800802	19520128	1	20111016	9
21931099	20120205	16119950	19470914	1	20120205	10
21980881	20130517	17537718	19580801	1	20130517	9

21997422	20130324	17366175	19510427	1	20130324	8
22025576	20110313	15172574	19520505	2	20110313	9
22101128	20120421	16351108	19361026	1	20120421	10
22261423	20110502	15315306	19850628	2	20110502	10
22269245	20111204	15943508	19260607	2	20111204	7
22428337	20111107	15865028	19250129	2	20111107	7
22488886	20131205	18176860	19440926	1	20131205	7
22536374	20120429	16369546	19400629	1	20120429	7
22539759	20131005	17981757	19310525	1	20131005	7
22554514	20120103	16031992	19500428	1	20120103	8
22639209	20130310	17324088	19601201	1	20130310	7
22936318	20110330	15217331	19580827	1	20110330	9
23025607	20131022	18033591	19431007	1	20131022	7
23034040	20111025	15827414	19350615	2	20111025	7
23085576	20110809	15608517	19641214	2	20110809	10
23132323	20110915	15713874	19271201	1	20110915	10
23246880	20121107	16952940	19650318	2	20121107	8
23253874	20110411	15254587	19491130	1	20110411	7
23258039	20130624	17650766	19570528	2	20130624	7
23272711	20130930	17957116	19670820	2	20130930	7
23310034	20131224	18235094	19690102	1	20131224	8
23335106	20110614	15443137	19640601	1	20110614	8
23511775	20130412	17426793	19350207	1	20130412	7
23512494	20130113	17159714	19481101	2	20130113	8
23543148	20120301	16196052	19531201	2	20120301	7
23557724	20120326	16271040	19210409	1	20120326	7
23784818	20110225	15124220	19400921	2	20110225	7
23886999	20120417	16338788	19510528	1	20120417	9
24210159	20121112	16966229	19470710	2	20121112	8
24256299	20121123	17001402	19311019	1	20121123	10
24295554	20120503	16385941	19610620	1	20120503	9
24367008	20110319	15191954	19340707	2	20110319	8
24445247	20110425	15295062	19671126	2	20110425	8
24481729	20110329	15216239	19380724	1	20110329	7
24534314	20110727	15567778	19261025	1	20110727	9
24657350	20131208	18185374	19570215	2	20131208	8
24799788	20120903	16752521	19340208	2	20120903	8
24826195	20121212	17064235	19510607	1	20121212	7
24913040	20130516	17534157	19311110	1	20130516	7
24934574	20111227	16011377	19530704	1	20111227	8
25396978	20120325	16268528	19401126	1	20120325	8
25398747	20110905	15683573	19440117	1	20110905	8
25462811	20111221	15997622	19540802	1	20111221	10
25558898	20130206	17235216	19740324	2	20130206	8
25574178	20130127	17200121	19520824	2	20130127	9
25607743	20110903	15678450	19371202	1	20110903	10
25704807	20120601	16470168	19341205	2	20120601	7
25705106	20120507	16398833	19381209	2	20120507	8
25727406	20110313	15172795	19601225	1	20110313	8
25862797	20130420	17452715	19500808	1	20130420	8
25904238	20110407	15240966	19550404	2	20110407	10
26068928	20111217	15986707	19920803	1	20111217	8
26164114	20121227	17110578	19820405	2	20121227	8
26174969	20130226	17281935	19281108	2	20130226	7
26213467	20130128	17203444	19570503	2	20130128	7

2627539820130730		17763415	19400107	1	20130730	8
2631486320130213		17244291	19341010	1	20130213	8
26483829	20120614	16515200	19600310	2	20120614	7
26802891	20110510	15343904	19630102	2	20110510	7
26815554	20120609	16499676	19250716	2	20120609	7
26939684	20111127	15921656	19551026	1	20111127	9
27076115	20110904	15678991	19761002	2	20110904	7
2708144320130505		17496217	19480503	2	20130505	7
27199662	20110510	15343899	19961119	1	20110510	10
27421609	20111229	16017337	19661219	2	20111229	7
27518872	20110703	15493475	19500102	1	20110703	8
27544770	20111125	15919934	19360405	1	20111125	7
27552756	20120905	16762341	19361220	1	20120905	8
27575593	20110604	15413998	19621120	1	20110604	7
2763422620130411		17425541	19590120	1	20130411	7
27657212	20111101	15846800	19590816	2	20111101	10
27678724	20120114	16069252	19590410	2	20120114	8
27704730	20120521	16440128	19541215	2	20120521	8
27729620	20120902	16746625	19621010	1	20120902	7
27871712	20120226	16182731	19420716	1	20120226	9
2790442320130222		17271978	19460802	1	20130222	7
2791493820130218		17255334	19720702	2	20130218	8
27937833	20121029	16920154	19530320	1	20121029	8
27959791	20120627	16548146	19450505	1	20120627	8
2798785520130928		17954312	19521229	2	20130928	7
2803673920130130		17209324	19310713	2	20130130	7
28040291	20110912	15702562	19420624	2	20110912	7
2807148920130331		17383605	19621209	1	20130331	8
2808231720130505		17495889	19350702	2	20130505	7
2816981520130613		17617422	19721006	2	20130613	7
28179079	20110607	15416358	19280123	1	20110607	8
2820417320131120		18128290	19690616	2	20131120	7
28280491	20111203	15941390	19370615	2	20111203	8
28300950	20111217	15986574	19651122	1	20111217	8
28315937	20110730	15574558	19340812	2	20110730	10
28442811	20120503	16387002	19490222	2	20120503	7
2846023320130915		17917487	19590508	1	20130915	7
28504270	20120316	16247907	19650923	1	20120316	7
28508681	20120715	16606676	19480515	1	20120715	10
28546896	20121021	16898935	19520622	2	20121021	8
2859251020130920		17932257	19391220	1	20130920	9
28698513	20120213	16145429	19290502	1	20120213	10
28733688	20121210	17056288	19370514	2	20121210	9
28763362	20110903	15678108	19690601	2	20110903	9
28938570	20110831	15666683	19480301	1	20110831	8
28952536	20111116	15894382	19450122	2	20111116	10
28959606	20120605	16484750	19420308	1	20120605	8
29032700	20120517	16431456	19390828	1	20120517	7
29293149	20110509	15336640	19410504	1	20110509	10
29346892	20120518	16432974	19520101	2	20120518	8
2956623220130605		17593912	19521229	1	20130605	9
29670426	20120916	16794534	19640530	1	20120916	8
29681912	20111124	15915880	19450728	1	20111124	7
29730630	20120109	16051741	19400403	1	20120109	7
29731122	20120515	16425434	19420416	2	20120515	10

29791999	20111120	15903347	19521110	2	20111120	10
29884513	20110820	15640813	19480905	1	20110820	10
29940010	20111107	15864798	19451113	2	20111107	7
29958063	20110401	15225013	19620220	1	20110401	10
29977308	20111215	15981651	19420609	2	20111215	7
29987357	20120822	16721747	19360221	1	20120822	8
30213139	20110521	15375219	19721206	1	20110521	8
30267095	20120223	16177910	19541120	1	20120223	7
30271488	20111012	15789636	19381113	1	20111012	9
30272243	20130710	17707542	19480504	1	20130710	8
30283706	20110223	15117727	19440221	1	20110223	10
30354031	20131126	18144336	19370115	1	20131126	8
30411768	20130329	17381655	19270704	1	20130329	8
30435122	20131003	17972841	19580826	2	20131003	7
30607751	20130429	17474604	19550112	1	20130429	9
30619728	20110829	15661666	19370505	1	20110829	7
30724255	20110828	15659147	19610906	1	20110828	10
30759263	20110501	15309271	19770826	1	20110501	8
30829595	20121218	17084518	19630215	1	20121218	7
31006061	20120703	16566828	19361026	2	20120703	10
31058249	20110311	15170463	19751219	2	20110311	8
31105336	20130730	17764172	19430129	2	20130730	8
31164575	20110831	15665739	19320327	2	20110831	10
31214865	20120125	16089825	19451112	2	20120125	8
31284318	20130703	17681596	19360708	2	20130703	8
31355267	20111203	15942891	19250228	1	20111203	8
31363141	20110418	15276948	19510507	2	20110418	10
31402796	20120320	16257578	19520506	2	20120320	7
31430418	20110611	15434239	19461125	1	20110611	7
31548762	20110824	15651168	19641118	1	20110824	7
31703829	20111102	15849696	19541220	1	20111102	7
31725118	20110529	15392216	20010822	2	20110529	7
31733934	20110819	15639222	19190724	2	20110819	10
31736648	20130312	17332721	19550326	1	20130312	7
31824789	20120419	16346298	19430826	1	20120419	9
31883020	20110223	15114313	19500109	2	20110223	10
31888285	20120309	16227139	19490123	2	20120309	9
31998693	20110426	15299262	19430101	2	20110426	7
32059782	20120304	16203939	19580520	1	20120304	9
32206078	20111129	15927755	19600805	1	20111129	8
32212229	20110621	15461682	19510226	1	20110621	10
32249357	20130311	17327088	19411203	1	20130311	8
32249697	20120326	16271166	20000924	2	20120326	7
32271151	20110415	15270103	19411016	1	20110415	10
32276747	20110924	15737868	19660701	1	20110924	10
32348224	20110824	15651282	19830922	2	20110824	8
32385378	20110719	15546327	19531226	1	20110719	10
32471559	20110802	15585921	19431223	1	20110802	7
32475437	20110521	15375175	19581020	1	20110521	7
32509481	20110418	15276771	19670627	1	20110418	8
32537421	20121219	17087987	19591104	1	20121219	8
32582119	20130311	17327503	19551214	1	20130311	8
32665422	20111102	15851683	19510521	1	20111102	8
32688974	20130411	17423966	19320715	2	20130411	7
32816721	20121004	16851071	19381120	2	20121004	8

32832556	20130417	17442130	19590516	2	20130417	8
32844716	20110927	15744530	19280202	2	20110927	7
32934682	20110330	15219202	19500311	2	20110330	7
33022047	20110629	15483263	19550209	1	20110629	7
33053097	20121203	17031429	19380818	2	20121203	10
33096105	20120612	16507957	19620212	1	20120612	8
33101347	20130203	17220673	19500929	1	20130203	8
33120717	20131009	17996824	19541126	2	20131009	7
33177467	20131122	18134324	19510126	1	20131122	7
33193214	20111014	15798400	19720212	1	20111014	10
33205559	20120820	16714798	19381206	1	20120820	8
33426812	20111004	15766104	19280415	1	20111004	9
33489815	20110918	15719370	19531017	2	20110918	7
33524435	20131117	18118304	19431008	1	20131117	7
33570864	20120424	16356955	19621107	1	20120424	8
33617219	20130318	17347979	19650110	1	20130318	9
33650145	20130918	17928163	19310217	2	20130918	9
33651808	20111126	15920548	19450908	1	20111126	8
33690298	20110221	15109938	19440730	2	20110221	9
33706853	20120405	16301567	19560901	1	20120405	7
33771243	20110219	15105539	19460204	1	20110219	10
33880216	20130312	17332441	19510425	2	20130312	8
33925096	20111214	15974791	19380815	2	20111214	8
33946622	20120706	16581025	19410317	1	20120706	7
33986913	20130822	17840945	19411126	1	20130822	7
34002410	20110510	15339962	19470607	1	20110510	8
34042563	20130215	17248631	19680706	1	20130215	7
34131118	20121205	17040736	19260709	2	20121205	8
34189123	20121104	16938626	19460606	1	20121104	7
34216927	20120509	16407005	19400804	1	20120509	7
34247682	20120307	16218992	19560421	1	20120307	7
34291333	20120510	16411278	19490920	2	20120510	10
34334004	20130803	17778693	19500615	1	20130803	8
34369658	20130309	17323796	19610822	1	20130309	7
34410407	20120614	16512563	19380731	1	20120614	10
34413315	20110312	15172103	19550102	2	20110312	9
34426738	20130227	17285490	19530309	1	20130227	8
34442347	20111027	15833148	19290915	1	20111027	7
34495982	20111011	15787202	19530725	1	20111011	8
34504057	20120103	16032294	19620522	1	20120103	8
34716046	20130918	17929712	19440505	1	20130918	8
34720791	20120711	16596404	19791212	2	20120711	7
34767607	20110421	15287569	19580529	1	20110421	8
34769078	20110903	15678440	19330801	1	20110903	8
34773790	20120126	16090580	19390923	2	20120126	8
34774646	20120610	16500127	19510125	2	20120610	9
34779732	20110421	15287072	19410726	1	20110421	8
34925854	20110802	15583934	19361126	1	20110802	8
34990222	20120130	16095990	19590220	1	20120130	10
34995794	20120129	16094362	19390102	1	20120129	8
35010378	20120310	16228962	19400102	1	20120310	7
35075071	20120109	16051284	19620702	1	20120109	8
35162562	20110617	15453761	19530726	1	20110617	8
35167841	20110407	15243223	19430115	1	20110407	10
35210710	20120106	16042883	19580717	2	20120106	8

35227884	20130316	17344668	19390508	2 20130316	8
35235655	20130411	17423840	19561102	2 20130411	7
35241475	20110425	15296068	19500730	1 20110425	10
35313872	20120912	16785430	19650516	1 20120912	10
35324846	20121107	16951690	19510210	1 20121107	8
35339765	20120323	16264387	19430913	1 20120323	8
35373343	20130413	17430742	19371107	2 20130413	7
35400227	20120204	16119265	19661213	2 20120204	10
35465964	20120805	16664962	19441130	1 20120805	8
35470792	20121026	16913911	19641020	1 20121026	9
35487913	20111027	15833149	19490828	2 20111027	7
35488938	20120112	16063525	19651017	2 20120112	7
35496845	20120312	16232072	19611010	1 20120312	8
35527425	20120814	16697986	19430816	1 20120814	8
35536391	20120222	16174996	19510415	2 20120222	7
35561912	20110806	15600442	19521026	1 20110806	10
35585569	20111216	15984779	19520818	1 20111216	7
35594844	20120731	16650568	19650114	1 20120731	9
35598584	20110309	15162828	19530328	2 20110309	10
35711150	20131106	18082675	19510901	2 20131106	8
35767498	20110523	15379084	19670615	1 20110523	10
35843642	20110614	15443066	19520727	1 20110614	8
35861724	20111108	15870656	19450304	1 20111108	8
35876983	20110715	15537218	19521101	2 20110715	8
35900471	20130527	17563878	19760627	2 20130527	7
35926842	20110318	15190483	19841201	1 20110318	10
35982866	20120103	16029672	19681016	2 20120103	8
35994184	20111029	15837525	19570607	1 20111029	8
36001157	20120305	16209145	19791019	2 20120305	7
36072750	20110925	15738513	19960102	2 20110925	8
36093740	20120702	16562932	19660613	1 20120702	10
36113129	20120907	16769310	19420610	2 20120907	7
36116913	20110422	15289805	20020712	2 20110422	9
36126031	20110329	15216257	19610912	2 20110329	10
36169309	20130817	17828751	19530930	2 20130817	7
36180071	20130803	17779795	19600210	1 20130803	8
36195401	20130822	17841360	19501025	2 20130822	8
36201942	20130121	17183963	19360122	1 20130121	8
36209231	20120611	16504360	19520706	1 20120611	7
36238310	20120821	16717629	19550101	1 20120821	7
36296943	20110617	15453403	19640314	1 20110617	7
36327034	20110422	15289566	19470114	2 20110422	7
36333150	20110408	15247320	19530612	1 20110408	9
36335929	20110527	15390507	19650817	1 20110527	7
36371376	20120603	16474743	19510120	1 20120603	8
36429273	20120301	16196424	19560913	1 20120301	8
36432572	20120114	16067634	19380610	1 20120114	8
36433735	20111011	15785961	19250429	1 20111011	8
36454725	20110309	15163042	19621023	1 20110309	9
36456061	20110327	15210338	19981028	2 20110327	7
36460283	20110318	15190473	19730929	2 20110318	8
36513903	20110629	15482935	19961118	1 20110629	8
36524455	20110530	15395160	19830603	2 20110530	10
36530082	20110321	15196433	19500818	2 20110321	8
36571650	20111014	15795773	19230809	1 20111014	7

3658511220130621		17643453	19260907	2	20130621	7
36617160	20110819	15639202	19520111	2	20110819	8
36637635	20110613	15438733	19540623	1	20110613	10
36680509	20110928	15748034	19531010	2	20110928	7
36743267	20110513	15354475	19871009	2	20110513	8
36765250	20110912	15702617	19400411	1	20110912	7
36776268	20110504	15324027	20000607	1	20110504	10
36795398	20111110	15877472	19640327	1	20111110	10
3680242320130228		17288256	19360127	2	20130228	7
36802730	20110401	15226251	19830811	2	20110401	10
36808330	20110316	15183771	19710109	2	20110316	10
36842838	20110303	15143949	19960403	2	20110303	7
36846545	20111012	15791206	20000922	1	20111012	10
3685407620131107		18087950	19660428	1	20131107	7
36878065	20110501	15309338	19610720	1	20110501	7
36880474	20110906	15686553	19640310	1	20110906	8
36919296	20111030	15838062	19941007	1	20111030	8
36930311	20120928	16830024	19430122	1	20120928	7
36935270	20111106	15862490	19930412	2	20111106	8
36952213	20110408	15247609	19341205	2	20110408	10
3695516520130216		17249936	19630312	2	20130216	8
36966184	20110907	15692419	19750410	1	20110907	8
36984733	20120601	16470234	20010926	2	20120601	9
37007640	20110730	15574674	19510111	1	20110730	10
37013788	20110221	15110394	19661010	2	20110221	8
3701835220131116		18117642	19520827	1	20131116	8
37020589	20110530	15394440	19661116	1	20110530	8
37038952	20120219	16164376	19380115	1	20120219	8
37052236	20120108	16047613	19640331	2	20120108	8
37052601	20110411	15253070	19331205	1	20110411	7
37057015	20111213	15972735	19240904	1	20111213	7
37075244	20120126	16090704	19571018	2	20120126	10
37079473	20110324	15205881	19751222	1	20110324	10
3708618320130103		17128279	19680823	2	20130103	8
37118040	20110904	15678705	19670406	2	20110904	7
37172886	20110430	15308332	19720925	2	20110430	8
3719765220131205		18178347	19630510	2	20131205	7
3720254320130511		17517229	19391104	2	20130511	8
37213937	20110408	15247596	19510115	1	20110408	8
37249824	20110830	15664140	19970116	2	20110830	8
37271979	20110313	15172740	19581110	1	20110313	7
37299835	20110329	15215478	19920514	1	20110329	10
37312520	20110423	15292061	19390822	2	20110423	7
37313512	20110225	15124027	19550128	2	20110225	10
3732001720130702		17676557	19550420	2	20130702	8
37339256	20110323	15202816	19871128	1	20110323	7
37340479	20111004	15762103	19540624	1	20111004	10
37350768	20111217	15986734	19621212	1	20111217	10
37359049	20111117	15898043	19660314	1	20111117	8
37372171	20110522	15375740	19501209	1	20110522	8
3737345820130510		17516583	19780212	2	20130510	7
37374279	20111130	15931096	19481004	1	20111130	9
37389336	20110524	15381975	19461111	2	20110524	9
37391289	20120209	16135338	19550118	1	20120209	7
37419173	20110413	15263565	19410516	1	20110413	8

37424161	20110408	15247327	19650811	1	20110408	10
3742556220131021		18028039	19680615	1	20131021	8
37437653	20110814	15622263	19590106	2	20110814	7
37447475	20110516	15360332	19481003	2	20110516	10
3745713920130120		17180441	19220810	1	20130120	7
37506302	20110616	15450620	19660715	1	20110616	10
37520255	20111009	15782317	19411115	1	20111009	8
37536575	20110810	15612908	19530525	2	20110810	10
37537307	20110616	15450583	19501102	1	20110616	9
37539494	20110305	15149517	19630424	1	20110305	8
3754744720131105		18077590	19380102	2	20131105	8
37556379	20111007	15780090	19680215	1	20111007	10
37579809	20120603	16474500	19490915	2	20120603	8
37583689	20110322	15198230	19370901	2	20110322	7
37591085	20110506	15330751	19370609	2	20110506	7
37591201	20110228	15128690	19820927	2	20110228	9
37609997	20120217	16162039	19401018	2	20120217	8
3765250920130202		17219730	19381110	2	20130202	8
37659317	20111012	15791180	19591108	1	20111012	7
37663608	20110507	15334499	19521215	1	20110507	10
3767139920130421		17453402	19291202	1	20130421	8
37702242	20120907	16770739	19700114	2	20120907	8
37702264	20110223	15117121	19350210	1	20110223	7
37711163	20110511	15344005	19481115	2	20110511	8
37711765	20110310	15167018	19720901	2	20110310	10
37734773	20110712	15525179	19900806	1	20110712	7
37749352	20120526	16454023	19640707	1	20120526	8
37750086	20110507	15334640	19481120	1	20110507	9
37759732	20110430	15308537	19840806	1	20110430	10
37762906	20110304	15147208	19430618	1	20110304	8
37767514	20110328	15213631	19971003	1	20110328	10
37773129	20110210	15078080	19560607	2	20110210	10
37773958	20110407	15243376	19410515	2	20110407	7
37786166	20111214	15977692	19551224	2	20111214	10
37788786	20111120	15903441	19610119	1	20111120	8
37791552	20110924	15737073	19470902	1	20110924	7
37792431	20110328	15213650	19360923	1	20110328	9
37800398	20120205	16119542	19570222	1	20120205	7
3780678320130224		17273938	19620905	2	20130224	8
37810610	20120716	16610583	19591124	1	20120716	7
37820329	20110430	15308757	19541110	2	20110430	8
3782370420130515		17528143	19590126	1	20130515	9
37823919	20120130	16097264	19860919	1	20120130	7
37824730	20111231	16022157	19370104	1	20111231	8
37839955	20110907	15692240	19550205	1	20110907	8
37840894	20111001	15755699	19400126	1	20111001	10
37850649	20110331	15221753	19510601	2	20110331	9
37853353	20110413	15263601	19521208	1	20110413	7
37853557	20110327	15210327	19291215	2	20110327	8
37854889	20120115	16069712	19520828	1	20120115	8
37857300	20111003	15761454	19590705	1	20111003	9
37858530	20110311	15170390	19731102	1	20110311	7
3787105920130301		17291980	19560815	1	20130301	9
37873420	20110620	15458427	19540103	2	20110620	8
37880969	20120815	16701941	19710707	1	20120815	7

37881882	20120105	16039716	19470411	2	20120105	7
37887517	20110623	15469483	19420624	1	20110623	8
37923732	20110330	15218606	19650318	1	20110330	10
37924848	20110321	15196342	19610327	1	20110321	9
37927121	20111212	15970379	19500221	2	20111212	7
37927734	20120716	16609728	19611120	2	20120716	10
3793248220130410		17419507	20071009	1	20130410	8
37936699	20120514	16421514	19421128	2	20120514	8
37937614	20110524	15382324	19450208	1	20110524	7
37937954	20110307	15154619	19510713	1	20110307	9
37954362	20120430	16371719	19511222	1	20120430	10
37977030	20111006	15775821	19560410	1	20111006	7
37977530	20110409	15249424	19330928	2	20110409	8
37978088	20110514	15356064	19520120	1	20110514	8
37991541	20110525	15384802	19571107	1	20110525	10
37995054	20110824	15651257	19480115	1	20110824	8
37999716	20110416	15272157	19580821	1	20110416	8
38013182	20110330	15219194	19810503	1	20110330	7
3801902220130515		17531848	19410201	1	20130515	8
38020392	20110608	15420736	19750512	1	20110608	7
38024407	20110621	15462937	19541214	1	20110621	8
38031786	20110409	15249389	19510610	1	20110409	10
38034887	20120809	16682378	19541231	2	20120809	10
38035733	20110628	15480371	19770922	2	20110628	10
38053257	20110503	15318200	19500130	2	20110503	8
38076378	20110823	15646407	19700114	2	20110823	7
38080034	20110614	15441384	19680924	2	20110614	9
38087353	20120627	16547329	19600512	2	20120627	8
38094096	20120903	16749072	19360718	1	20120903	7
38110097	20110913	15706617	19630723	2	20110913	8
38114782	20110405	15233726	19460808	1	20110405	8
38115741	20120614	16515268	19431020	1	20120614	8
38123545	20110731	15574957	19570607	2	20110731	8
38132002	20110902	15673336	19431118	2	20110902	8
38139763	20120522	16443123	19550128	1	20120522	7
38148219	20110819	15639008	19451102	1	20110819	7
38148297	20110502	15315086	19760122	1	20110502	9
38156160	20110805	15598324	19380414	1	20110805	7
38157152	20110306	15149786	19301016	1	20110306	10
38162399	20110711	15522814	19650906	1	20110711	8
38165309	20110914	15709944	19671126	1	20110914	10
38171663	20110409	15249570	19561201	1	20110409	10
38171721	20110706	15505117	19470817	1	20110706	8
38176271	20110420	15284094	19630202	1	20110420	9
3817774120130523		17554473	19660519	1	20130523	9
38179601	20120307	16217760	19481001	1	20120307	9
3818023320130731		17764308	19520908	2	20130731	9
38182557	20121028	16917206	19690628	2	20121028	7
38183889	20111006	15775172	19440925	1	20111006	8
38189310	20110522	15375642	19480410	1	20110522	8
38208478	20110926	15740909	19240701	1	20110926	7
38209391	20110821	15641128	19590818	1	20110821	9
38213159	20110612	15434706	19661218	1	20110612	10
38223335	20110624	15471753	19480821	1	20110624	7
38232063	20110529	15392244	19480131	2	20110529	7

3823321520130326		17371952	19381030	1	20130326	8
38238072	20110610	15432110	19820408	1	20110610	7
38239564	20120819	16711683	19550116	1	20120819	7
38246536	20110618	15455388	19650919	1	20110618	7
38258503	20120206	16124567	19540619	1	20120206	8
38260887	20120101	16022186	19400219	1	20120101	8
38267173	20111114	15886999	19530105	2	20111114	9
38272887	20110906	15685396	19451016	1	20110906	10
38289575	20120827	16733123	19480101	2	20120827	7
38291279	20111014	15798420	19671001	1	20111014	8
38291939	20110512	15351638	19470220	1	20110512	7
38314011	20110905	15683553	19380531	1	20110905	10
3831739420130520		17545102	19490901	1	20130520	7
38320068	20110627	15477200	19510707	1	20110627	7
38320466	20111012	15791463	19580228	1	20111012	7
38323681	20110819	15639362	19951212	1	20110819	10
38345663	20110506	15331476	19410303	2	20110506	8
38346519	20110818	15633981	19480825	2	20110818	8
38360188	20110829	15660065	19590101	1	20110829	8
38367872	20120202	16112633	19610820	1	20120202	10
38375438	20111014	15797742	19611201	1	20111014	7
38377478	20110715	15535920	19620502	1	20110715	10
38380891	20111025	15827336	19411231	2	20111025	7
38385227	20110825	15654434	19420212	1	20110825	7
38395027	20110912	15702629	19470520	1	20110912	8
38403757	20120423	16354013	19611224	1	20120423	8
38422707	20110824	15651230	19790206	1	20110824	10
38428818	20110926	15740079	19510327	2	20110926	7
38435120	20110912	15702507	19660414	2	20110912	10
38435880	20121028	16917041	19391213	1	20121028	8
38471908	20111210	15966046	19681202	1	20111210	9
38486907	20110805	15598695	19950929	2	20110805	8
38489019	20111128	15925034	19300212	1	20111128	8
38508951	20111002	15756667	19661031	1	20111002	8
38509476	20111025	15827346	19640921	1	20111025	7
38520273	20110923	15736346	19690101	1	20110923	8
38529123	20110826	15656816	19441101	1	20110826	7
38530437	20111109	15873940	19280116	2	20111109	8
38555349	20111004	15762052	19691105	2	20111004	8
38564691	20120104	16037199	19750203	1	20120104	8
38568819	20110824	15649597	19970324	2	20110824	7
38579827	20110922	15732050	19340403	1	20110922	8
38586559	20120305	16209544	19541105	2	20120305	8
38596622	20111108	15870077	19580711	1	20111108	7
38621771	20111121	15906958	19400218	1	20111121	8
38625524	20111024	15823575	19500320	2	20111024	7
38625819	20110911	15702129	19571216	1	20110911	8
38626323	20120216	16159378	19640120	1	20120216	10
38626583	20120616	16520433	19781029	1	20120616	8
38629231	20120703	16567115	19540830	1	20120703	8
38634036	20111113	15883165	19350706	1	20111113	8
38642396	20111026	15829419	19531219	1	20111026	8
38642772	20111201	15936506	19740905	2	20111201	10
38650203	20120130	16098055	19601010	1	20120130	7
38661084	20120111	16060209	19291122	1	20120111	7

38663386	20120112	16063007	19411001	1	20120112	7
38664958	20110922	15732352	19421218	1	20110922	8
38667106	20120630	16556175	19680702	1	20120630	7
38678169	20111025	15825299	19510910	1	20111025	8
38679833	20130606	17597044	19730628	2	20130606	7
38690016	20120227	16184634	19530910	2	20120227	7
38703650	20111205	15946305	19740416	1	20111205	10
38706342	20111212	15966688	19531110	1	20111212	9
38710940	20111108	15870556	19521003	1	20111108	7
38722779	20120109	16051932	19780121	1	20120109	8
38732319	20131209	18189250	19571219	2	20131209	8
38733856	20130615	17624967	19570201	1	20130615	8
38760064	20120105	16039724	19441222	1	20120105	10
38761318	20130827	17854353	19560329	1	20130827	7
38769209	20130815	17822183	19240909	2	20130815	7
38776599	20120108	16047371	19570831	2	20120108	8
38778380	20130222	17270929	19550205	1	20130222	7
38783878	20111205	15947571	19490320	1	20111205	8
38789401	20120605	16484507	19671123	2	20120605	7
38801604	20120724	16634137	19700323	2	20120724	7
38802469	20120223	16177129	19620202	1	20120223	7
38802721	20120528	16457529	19511119	2	20120528	10
38804056	20120214	16151546	19650203	1	20120214	7
38808796	20120531	16466321	19470908	1	20120531	8
38829571	20120229	16190259	19480828	1	20120229	7
38833737	20120729	16645344	19560202	2	20120729	8
38837217	20120302	16199104	19521008	2	20120302	8
38852221	20130512	17519182	19620722	1	20130512	7
38855606	20120406	16307240	19431008	2	20120406	8
38862394	20120312	16232892	19720911	1	20120312	7
38872025	20120219	16164361	19640606	1	20120219	9
38876083	20120604	16478483	19461111	1	20120604	8
38877735	20120307	16216977	19741128	1	20120307	7
38880772	20120328	16277537	19650202	1	20120328	7
38888027	20120726	16640561	19360128	2	20120726	8
38891053	20120518	16433787	19430413	1	20120518	7
38907914	20130821	17838375	19261120	2	20130821	7
38910279	20120619	16527987	19540115	1	20120619	7
38913609	20120308	16221791	19750414	1	20120308	8
38914839	20130315	17343352	19480812	1	20130315	8
38926077	20120827	16732973	19811127	2	20120827	7
38943509	20130102	17122163	19410523	1	20130102	8
38943963	20120424	16358888	19710925	1	20120424	8
38945652	20120705	16575677	19560102	1	20120705	10
38946633	20120409	16315628	19560520	2	20120409	8
38958973	20131206	18181817	19490528	1	20131206	8
38964975	20120423	16355318	19651106	1	20120423	10
38973852	20120329	16281229	19641222	2	20120329	10
38978197	20120517	16431929	19640306	2	20120517	7
38979690	20120813	16694926	19620429	1	20120813	7
38980733	20121129	17018186	19730828	2	20121129	7
38984973	20120518	16434879	19340204	1	20120518	9
38989229	20120523	16444029	19811225	2	20120523	8
38994239	20121224	17100654	19340619	1	20121224	7
39011273	20120504	16392780	19420120	2	20120504	8

39014125	20120408	16310845	19431029	1	20120408	8
3901513920130218		17254751	19580302	1	20130218	7
39016405	20120531	16466208	19900523	1	20120531	10
39018854	20120604	16479273	19510816	2	20120604	7
39029771	20120415	16333648	19820208	2	20120415	7
3904913320130303		17296805	20040817	1	20130303	8
39051199	20120318	16250786	19680418	2	20120318	7
39061911	20120919	16805717	19640103	1	20120919	8
39064578	20121015	16882442	19501207	1	20121015	7
39065695	20121225	17102454	19340901	2	20121225	7
39074061	20120430	16372769	19630402	1	20120430	8
39082945	20120806	16668367	19510725	1	20120806	8
3908348220130316		17345203	19421031	1	20130316	7
39094718	20120827	16731640	19691123	1	20120827	10
39113592	20120719	16619837	19700801	2	20120719	9
39136884	20120726	16640759	19530315	2	20120726	8
3915338320130111		17156646	19540326	1	20130111	7
3915529820130507		17503941	19480205	1	20130507	8
39155561	20120716	16610582	19571102	1	20120716	8
39165849	20120714	16606254	19630911	2	20120714	8
39167936	20120711	16597212	19581103	1	20120711	8
39168008	20120819	16711842	19580306	2	20120819	8
39175503	20120830	16740360	19550522	1	20120830	7
39177338	20120531	16465221	19510830	1	20120531	8
3918016020130724		17749347	19540518	2	20130724	7
39180911	20120712	16599396	19590617	1	20120712	7
3918243920131225		18237110	19440417	1	20131225	8
39187514	20120910	16777230	19611205	1	20120910	8
39190142	20120903	16752652	19321010	1	20120903	8
39190299	20120827	16733217	19540719	1	20120827	8
3919261520130511		17518843	19681018	2	20130511	9
39201451	20120601	16469925	19740109	2	20120601	10
39216665	20120702	16563100	19660608	1	20120702	7
3922190420130129		17206587	19380516	2	20130129	8
39230654	20120710	16592320	19510810	1	20120710	7
3925032320130430		17478148	19660510	1	20130430	8
39250492	20120917	16796676	19760731	1	20120917	8
39266416	20120720	16624767	19551010	1	20120720	8
39276625	20120904	16757654	19690208	1	20120904	7
39278256	20120828	16736110	19570701	1	20120828	8
39280450	20120814	16699063	19360518	1	20120814	7
3933721820130430		17478406	19420323	1	20130430	7
39354159	20120808	16678737	19381128	1	20120808	8
39363989	20120911	16781578	19410524	1	20120911	8
3938208620130130		17208436	19631224	1	20130130	8
3938583620130319		17353199	19650408	2	20130319	7
39386737	20120926	16823515	19570205	1	20120926	10
3938957620130223		17273622	19280103	1	20130223	8
39392693	20121117	16985531	19530804	1	20121117	8
3940209020130415		17435777	19510311	2	20130415	8
3940258920130303		17296813	19701117	1	20130303	8
39411262	20121001	16835253	19620128	1	20121001	10
39452089	20121121	16996436	19470107	1	20121121	8
3947130220130924		17941708	19560209	1	20130924	7
39476318	20121031	16925936	19510208	1	20121031	9

39481997	20130128	17203544	19530810	1	20130128	7
39498323	20130118	17177626	19560313	2	20130118	8
39503769	20121023	16905925	19400301	1	20121023	7
39514711	20130102	17119830	19680923	2	20130102	8
39519921	20121212	17066847	19830513	1	20121212	10
39520848	20131111	18096764	19580218	2	20131111	7
39520860	20130318	17348438	19541002	1	20130318	8
39529721	20130311	17328689	19621107	1	20130311	8
39542762	20130801	17771333	19690528	2	20130801	7
39561563	20121210	17057135	19441202	1	20121210	8
39574000	20130829	17860143	19570130	1	20130829	7
39579890	20130221	17268478	19970701	1	20130221	9
39584139	20130417	17441395	19420618	1	20130417	7
39591258	20130903	17875942	19651201	1	20130903	8
39595169	20130204	17225297	19500826	1	20130204	8
39597825	20130407	17407416	19910529	2	20130407	8
39606707	20130713	17715513	19340424	2	20130713	7
39635728	20130321	17359885	19600721	1	20130321	8
39637133	20121217	17081346	19480613	1	20121217	7
39645039	20130210	17242346	19611119	2	20130210	7
39645233	20130522	17551847	19590115	1	20130522	7
39647557	20130619	17637549	19470301	1	20130619	7
39651462	20130810	17806006	19470409	1	20130810	7
39665719	20130401	17389040	19750307	1	20130401	7
39667271	20130113	17159837	19560120	1	20130113	7
39686890	20130326	17373224	19641113	2	20130326	9
39693975	20130220	17264443	19510917	1	20130220	7
39698232	20130606	17598165	19560201	2	20130606	8
39724328	20130421	17453080	19651105	2	20130421	9
39725183	20130107	17137124	19540524	1	20130107	8
39732917	20130226	17281226	19800203	1	20130226	7
39740711	20130815	17821138	19470111	1	20130815	8
39746786	20130502	17488988	19490107	2	20130502	8
39765112	20130803	17780276	19990819	1	20130803	9
39769636	20130327	17375294	19440718	1	20130327	9
39805420	20130623	17647049	19680615	2	20130623	7
39808065	20130226	17280674	19940630	1	20130226	7
39881680	20130227	17284617	19860325	2	20130227	7
39902346	20130409	17414024	19481112	1	20130409	8
39919545	20130428	17471948	19610215	1	20130428	9
39931038	20130602	17578540	19641201	1	20130602	9
39932622	20130826	17849904	19711114	1	20130826	8
39969636	20131111	18099858	19580820	2	20131111	7
39970042	20130514	17527342	19660421	1	20130514	7
39982882	20130529	17570092	19761021	2	20130529	7
39982917	20130429	17474008	19631025	2	20130429	7
39995590	20130531	17575619	19590808	1	20130531	7
40012473	20130530	17572456	19830720	2	20130530	7
40018733	20130424	17463853	19471025	1	20130424	8
40023652	20130602	17578764	19530105	1	20130602	8
40028351	20130417	17444247	19710809	1	20130417	8
40032551	20130529	17570228	19540301	1	20130529	8
40046535	20130827	17853428	19700425	2	20130827	8
40055138	20130622	17646732	19520401	2	20130622	8
40055218	20130927	17950865	19530810	1	20130927	9

4005636820130424	17462923	19640701	1	20130424	7
4006358920130806	17791148	19640718	1	20130806	7
4007533020130422	17456159	19641005	1	20130422	8
4008153620131113	18108267	19431018	1	20131113	7
4008264220130602	17578702	19610525	1	20130602	8
4015898320130704	17684241	19680325	1	20130704	8
4018214720131208	18185038	19520211	1	20131208	7
4018766420130701	17666678	19670426	1	20130701	7
4018828120130818	17829296	19570516	1	20130818	7
4018935320131018	18023036	19591201	1	20131018	9
4019011220130714	17715786	19510909	1	20130714	7
4019256120130912	17911618	19620929	2	20130912	7
4019731720130926	17948510	19661018	2	20130926	8
4020773620130708	17696977	19490402	1	20130708	8
4022133820130721	17738285	19480610	1	20130721	8
4023268620130827	17854683	19940519	2	20130827	8
4023854820130714	17716121	19610817	2	20130714	8
4024143820131001	17963447	19970218	2	20131001	7
4024755020131104	18071846	19480421	1	20131104	7
4025694820131013	18003433	19610324	2	20131013	8
4038872520131104	18071988	19780612	1	20131104	7
4038903520131014	18005933	19540919	1	20131014	7
4039225420130807	17792690	19640118	2	20130807	7
4040622220130829	17859442	19401110	1	20130829	8
4042841920131016	18015871	19930917	2	20131016	9
4045620020130827	17854677	19481105	1	20130827	8
4046226820131005	17980741	19711112	1	20131005	7
4046386320130921	17933413	19570225	2	20130921	8
4051430520131207	18184842	19901004	1	20131207	8
4066256420131220	18225538	19500920	2	20131220	8
59767 20111205	15947114	19421027	2	20111205	9
105997 20120101	16022259	19330216	1	20120101	7
32987320130419	17450435	19340905	1	20130419	9
738738 20111024	15822904	19310303	1	20111024	7
75970620130627	17660139	19330717	1	20130627	7
780267 20110320	15192325	19400422	2	20110320	8
1048517 20121127	17013282	19370709	2	20121127	8
1342796 20120405	16303331	19281221	1	20120405	7
1376825 20120113	16065081	19240611	1	20120113	8
1445665 20120120	16085768	19510223	2	20120120	7
1502416 20111015	15799744	19250405	1	20111015	8
182235120130808	17799332	19330919	1	20130808	7
186229920130816	17826843	19290822	2	20130816	7
1987560 20120806	16668728	19300205	2	20120806	8
2035098 20120126	16090726	19360105	1	20120126	9
207515220131022	18030527	19350325	2	20131022	8
2307837 20111206	15951674	19480612	2	20111206	9
2320414 20110907	15692057	19320320	2	20110907	8
241435920131113	18106329	19540321	1	20131113	8
2573184 20121116	16982945	19351011	1	20121116	8
2587782 20120824	16727803	19500928	2	20120824	8
263447120130428	17471902	19440927	2	20130428	8
2691830 20111214	15977071	19390118	2	20111214	8
2979717 20121025	16910534	19440218	1	20121025	7
3314792 20120302	16201064	19200310	2	20120302	8

3412371	20130403	17400553	19290613	2	20130403	7
3418608	20121111	16963660	19491108	1	20121111	7
3522789	20131122	18135832	19400806	2	20131122	8
4145942	20130630	17665625	19501209	1	20130630	7
4307737	20120608	16497206	19401012	1	20120608	7
4752505	20120131	16099958	19250415	1	20120131	7
4797335	20120127	16091961	19340310	1	20120127	8
5542381	20121122	16998566	19260506	2	20121122	7
5707415	20110810	15612823	19421218	1	20110810	10
5730949	20130130	17208397	19551221	1	20130130	7
5795857	20130428	17471895	19371112	1	20130428	7
5912072	20120120	16086857	19560705	1	20120120	10
6129891	20111215	15981034	19291007	1	20111215	8
6409887	20110609	15428795	19490415	1	20110609	10
6546765	20120628	16552129	19340404	1	20120628	10
6565840	20111031	15839853	19251010	1	20111031	10
6766967	20110611	15433790	19560208	2	20110611	7
7291261	20120215	16155779	19310801	1	20120215	9
7455096	20110519	15369447	19260101	1	20110519	7
7525362	20130824	17846915	19780906	1	20130824	8
7541642	20130915	17917660	19520228	1	20130915	8
7736858	20110817	15633369	19420116	2	20110817	10
7808540	20130423	17459140	19390222	1	20130423	8
7966383	20121018	16893874	19500423	1	20121018	9
8180232	20131101	18061436	19401203	1	20131101	9
8383548	20130314	17338925	19451207	1	20130314	8
8630782	20130829	17859394	19300210	2	20130829	7
9000822	20130913	17915035	19260820	1	20130913	7
9485954	20120727	16642341	19300802	2	20120727	10
9614135	20110815	15624965	19550901	1	20110815	7
9685998	20130708	17696462	19310301	1	20130708	7
9790141	20120227	16185645	19320913	2	20120227	7
10074869	20110225	15124137	19330918	1	20110225	9
10353910	20131118	18122185	19500713	2	20131118	7
10434727	20120729	16645533	19441231	1	20120729	7
10939818	20120604	16477272	19431225	1	20120604	7
11045117	20120405	16303911	19430211	1	20120405	7
11351181	20111225	16005755	19460628	1	20111225	8
11598417	20121106	16945168	19381213	2	20121106	7
11752435	20120626	16545498	19410405	2	20120626	8
11987603	20120906	16765815	19460809	1	20120906	9
11993650	20120616	16520158	19610502	1	20120616	7
12110655	20121023	16906263	19521017	2	20121023	8
12219953	20121114	16975855	19630917	2	20121114	7
12426232	20120916	16794409	19270104	1	20120916	7
12481468	20130613	17619459	19531020	2	20130613	8
12550220	20110620	15459567	19551014	2	20110620	9
12587938	20130912	17909524	19580121	1	20130912	9
12614947	20130626	17657590	19291205	1	20130626	8
12798435	20130923	17935486	19510902	1	20130923	9
13007211	20120329	16279760	19390329	2	20120329	9
13019506	20110704	15495676	19580324	1	20110704	7
13280745	20120812	16690455	19420425	1	20120812	10
13287440	20130112	17158765	19271224	2	20130112	7
13823659	20120828	16736213	19350505	2	20120828	7

13854314	20110606	15415082	19541109	1	20110606	7
13939372	20110713	15530007	19470815	1	20110713	8
14031539	20111024	15823153	19591024	2	20111024	10
14447177	20111125	15919817	19520813	1	20111125	8
14477680	20121027	16916698	19340709	2	20121027	8
14600954	20120411	16322800	19500605	1	20120411	9
14927652	20111204	15943113	19470515	1	20111204	10
14992484	20110512	15351592	19540822	2	20110512	7
15575470	20120417	16340038	19520713	1	20120417	8
15735123	20110829	15660639	19600626	2	20110829	8
15759383	20120516	16428824	19680929	1	20120516	8
15883600	20110625	15473431	19580525	1	20110625	7
15975232	20120326	16271679	19550623	1	20120326	10
16002736	20120226	16182441	19490310	1	20120226	8
16084530	20120708	16584521	19600224	1	20120708	10
16095822	20120222	16175082	19501027	2	20120222	8
16272881	20111121	15905649	19460707	2	20111121	7
16383907	20121015	16881068	19330125	1	20121015	7
16449266	20110825	15653131	19601030	1	20110825	9
16556017	20111021	15818481	19501215	2	20111021	7
16621731	20120212	16143546	19500325	1	20120212	7
16726059	20120127	16092141	19361211	1	20120127	7
16798064	20111209	15964261	19500701	1	20111209	10
1682550620130729		17760982	19560605	2	20130729	7
17143565	20120222	16174325	19281107	1	20120222	8
17211551	20110809	15608845	19490315	1	20110809	9
1727206920130901		17864795	19530907	1	20130901	7
17349267	20110506	15332202	19361009	1	20110506	7
17366824	20120607	16493749	19350301	1	20120607	8
1738193020130730		17764118	19551224	1	20130730	8
17413320	20120907	16768602	19320902	1	20120907	8
1777199220130417		17444271	19400515	2	20130417	8
17789423	20110818	15634752	19330216	2	20110818	7
1792782120131209		18189687	19220524	2	20131209	7
18067011	20120105	16040610	19490302	2	20120105	10
18327292	20111224	16005358	19640416	2	20111224	10
1838842620131014		18007806	19871208	1	20131014	8
18406183	20111111	15880386	19320911	1	20111111	9
18568677	20110918	15719390	19480824	1	20110918	8
18749956	20110224	15120431	19341220	1	20110224	10
1884230320131116		18116463	19610115	2	20131116	8
18966502	20120601	16469538	19300625	1	20120601	7
19372793	20120323	16266676	19590418	2	20120323	8
1968480120130816		17826989	19700601	1	20130816	8
19719869	20111129	15928010	19620522	2	20111129	7
19816183	20110509	15339900	19700119	1	20110509	8
1994371620130606		17598966	19460224	1	20130606	7
19961081	20110621	15463077	19520117	2	20110621	7
2005994320130724		17749269	19580124	2	20130724	7
20216084	20110323	15201824	19560809	2	20110323	8
20245914	20120914	16791314	19600728	1	20120914	8
20547799	20120218	16163762	19300514	1	20120218	8
20579231	20120118	16080418	19540930	1	20120118	7
20596207	20121017	16888463	19390302	1	20121017	7
20655167	20111113	15883473	19541020	2	20111113	8

20859056	20130312	17332464	19351109	2	20130312	8
20902130	20111023	15820532	19550205	1	20111023	10
20996069	20131128	18151197	19680930	1	20131128	9
21012806	20130228	17287315	19341213	2	20130228	7
21101008	20111122	15909781	19270501	1	20111122	7
21544456	20110727	15567462	19500317	1	20110727	7
21674624	20120408	16310903	19510420	2	20120408	7
21822957	20130322	17363722	19580106	1	20130322	8
21861665	20130121	17184557	19300226	1	20130121	8
21965117	20131120	18127182	19630326	1	20131120	7
22025576	20110425	15295425	19520505	2	20110425	7
22101128	20120613	16510768	19361026	1	20120613	10
22455432	20120910	16777394	19490218	2	20120910	9
22639209	20130516	17532458	19601201	1	20130516	7
22710709	20120311	16229651	19710610	2	20120311	10
23005529	20111231	16022119	19321211	1	20111231	8
23084653	20120510	16411356	19680118	2	20120510	8
23085576	20110909	15698289	19641214	2	20110909	10
23132323	20110922	15732767	19271201	1	20110922	8
23246880	20121211	17061939	19650318	2	20121211	10
23265647	20120207	16129202	19361120	1	20120207	8
23367819	20110508	15335298	19580201	2	20110508	9
23784818	20110311	15170391	19400921	2	20110311	10
23886999	20120514	16421502	19510528	1	20120514	8
23896186	20121006	16857917	19940204	1	20121006	8
24175777	20110913	15705563	19391112	2	20110913	7
24301551	20120619	16525923	19571031	1	20120619	7
24481729	20110404	15230645	19380724	1	20110404	10
24576054	20120714	16605997	19241002	1	20120714	7
24654748	20120311	16229460	19940414	1	20120311	9
24896671	20120607	16490907	19430417	1	20120607	9
24913040	20130629	17665282	19311110	1	20130629	7
24934574	20120123	16088634	19530704	1	20120123	7
24956272	20131102	18066193	19261012	2	20131102	8
25028631	20130717	17728565	19291004	1	20130717	7
25156869	20110914	15710622	19530724	2	20110914	7
25257641	20120330	16283702	19661130	2	20120330	8
25258666	20110803	15589972	19640217	1	20110803	8
25273476	20121028	16917310	19550427	1	20121028	8
25421732	20120822	16721198	19390817	1	20120822	7
25445583	20110824	15650537	19620116	1	20110824	8
25489718	20120801	16655666	19370805	2	20120801	8
25741428	20110910	15701596	19701113	2	20110910	7
25989008	20121113	16969996	19630606	2	20121113	8
26007567	20111115	15890269	19700514	1	20111115	9
26164114	20130115	17167330	19820405	2	20130115	9
26275398	20130824	17847047	19400107	1	20130824	7
27004155	20120619	16525367	19500325	1	20120619	8
27298744	20111019	15811471	19400815	1	20111019	9
27359440	20120627	16548012	19980526	2	20120627	8
27544770	20130711	17711736	19360405	1	20130711	7
27552756	20120916	16794589	19361220	1	20120916	8
27614897	20110801	15581081	19530301	1	20110801	7
27722469	20120410	16318698	19280212	2	20120410	10
27745911	20120911	16779659	19460105	1	20120911	7

27877527	20110606	15415063	19580211	2	20110606	8
27911928	20130104	17131282	19520129	1	20130104	8
27913402	20130616	17625525	19550328	2	20130616	8
27987855	20131030	18055601	19521229	2	20131030	7
28082317	20130515	17531089	19350702	2	20130515	9
28170345	20111116	15894867	19460520	1	20111116	8
28214655	20111016	15800810	19530622	2	20111016	8
28264053	20120423	16353652	19360222	1	20120423	7
28349862	20110405	15233915	19320823	2	20110405	8
28375522	20120118	16080971	19561216	1	20120118	10
28509559	20120702	16563057	19550328	2	20120702	7
28531308	20130915	17917347	19550601	2	20130915	8
28669069	20120823	16725307	19560125	1	20120823	8
28938570	20110915	15713881	19480301	1	20110915	8
28952536	20111130	15930150	19450122	2	20111130	7
28955364	20111217	15986635	19331022	2	20111217	7
29269725	20130925	17944224	19341215	2	20130925	7
29385646	20110525	15384197	19450215	2	20110525	7
29410988	20121026	16915290	19700831	2	20121026	10
29454475	20130913	17915130	19641009	2	20130913	8
29619429	20110630	15485991	19421111	2	20110630	8
29731122	20120529	16460716	19420416	2	20120529	8
29776214	20121210	17057412	19300101	2	20121210	7
29839529	20121016	16885493	19541016	1	20121016	7
29856960	20110918	15719146	19520102	2	20110918	10
29884513	20111002	15756360	19480905	1	20111002	8
29897754	20110905	15683667	19541023	2	20110905	7
29940010	20111128	15924237	19451113	2	20111128	10
29951813	20110507	15334670	19630923	2	20110507	7
29987357	20121010	16867722	19360221	1	20121010	7
30110179	20130101	17117576	19610804	1	20130101	7
30202290	20111001	15756165	19650830	1	20111001	8
30271488	20111207	15955900	19381113	1	20111207	7
30290723	20110331	15221430	19660524	1	20110331	8
30411768	20130519	17541308	19270704	1	20130519	8
30548573	20131123	18137509	19690125	1	20131123	8
30574620	20130529	17569980	19440612	1	20130529	8
30587258	20110925	15738017	19621203	1	20110925	7
30607751	20130527	17562985	19550112	1	20130527	8
30709081	20120608	16497541	19481102	2	20120608	7
30724255	20120327	16275288	19610906	1	20120327	10
30798959	20130707	17693742	19271015	1	20130707	8
30807488	20110520	15373681	19320903	2	20110520	10
30811360	20131028	18049119	19410126	1	20131028	7
30902375	20120923	16814158	19430416	1	20120923	10
30916360	20110608	15422307	19610619	2	20110608	7
30946964	20111230	16019916	19631218	1	20111230	8
30966100	20130101	17117871	19410911	1	20130101	8
30974528	20120711	16597171	19530509	2	20120711	8
31109850	20121128	17015179	19311215	1	20121128	8
31214865	20120222	16173943	19451112	2	20120222	10
31261571	20130303	17296951	19620805	1	20130303	7
31355267	20120628	16551257	19250228	1	20120628	7
31355336	20120913	16788413	19420514	1	20120913	10
31363152	20110425	15296056	19751022	1	20110425	8

31412427	20111005	15770105	19490901	1	20111005	8
31538462	20120421	16351341	19500102	1	20120421	7
31678418	20110526	15387042	19570616	1	20110526	10
31698836	20120430	16372921	19640213	2	20120430	7
31729132	20130615	17625114	19390623	2	20130615	8
31957296	20130119	17179967	19341223	1	20130119	7
31981336	20120414	16333059	19630522	1	20120414	8
32061044	20131124	18137923	19360322	1	20131124	8
32105047	20120222	16175005	19550510	1	20120222	7
32198064	20111102	15849093	19370427	2	20111102	8
32206078	20120207	16128417	19600805	1	20120207	10
32385378	20110827	15658297	19531226	1	20110827	7
32475437	20110722	15555618	19581020	1	20110722	10
32509481	20110429	15306236	19670627	1	20110429	7
32537421	20130329	17381314	19591104	1	20130329	7
32582119	20130625	17653136	19551214	1	20130625	7
32648343	20130331	17383821	19610717	1	20130331	8
32696212	20130923	17936711	19690914	1	20130923	7
32709470	20111110	15877997	19650922	2	20111110	10
32776344	20120827	16732555	19650101	1	20120827	7
32797481	20120316	16248396	19510302	1	20120316	7
32805337	20130723	17745745	19420401	2	20130723	7
32818910	20111107	15866626	19660620	1	20111107	10
32934682	20110425	15296075	19500311	2	20110425	8
32973141	20120917	16797462	19710629	1	20120917	7
33009948	20130519	17541284	19630105	1	20130519	7
33101347	20130313	17336402	19500929	1	20130313	7
33135681	20120917	16798515	20040507	2	20120917	8
33140453	20111219	15990949	19540807	1	20111219	7
33161454	20130103	17123418	19450116	1	20130103	8
33275068	20110909	15700049	19460701	2	20110909	10
33288652	20130214	17246350	19411104	2	20130214	9
33294870	20120621	16533432	19660910	1	20120621	7
33391536	20120214	16151816	19601215	1	20120214	8
33471759	20120910	16777472	19660122	1	20120910	10
33478034	20120608	16497266	19581204	1	20120608	10
33490389	20111128	15924939	19590320	2	20111128	7
33514362	20111128	15922241	19650430	1	20111128	9
33517225	20120131	16101406	19860813	1	20120131	8
33556579	20130513	17520743	19480915	2	20130513	8
33573023	20131106	18080939	19330520	1	20131106	8
33767532	20120925	16820692	19571022	1	20120925	9
33853939	20121216	17077540	19491006	1	20121216	7
33871237	20131228	18245306	19551102	2	20131228	8
33890469	20110607	15416744	19510701	2	20110607	10
33989650	20120619	16527654	19560428	1	20120619	10
34010565	20120610	16500267	19561002	1	20120610	10
34084203	20130831	17864363	19341223	2	20130831	7
34098969	20110916	15716826	19451106	1	20110916	8
34403899	20111108	15869894	19460525	2	20111108	7
34420332	20120420	16349856	19631022	2	20120420	8
34436732	20130628	17663404	19300618	2	20130628	8
34504057	20120120	16086564	19620522	1	20120120	9
34741087	20110523	15379098	19370709	2	20110523	10
34747154	20120403	16297042	19500112	1	20120403	8

34770859	20120226	16182488	19960620	2	20120226	7
34774351	20110605	15414267	19700530	2	20110605	10
34779732	20110901	15672041	19410726	1	20110901	7
34925854	20110813	15621676	19361126	1	20110813	8
35241475	20110503	15320165	19500730	1	20110503	8
3529779120130128		17200864	19241009	2	20130128	9
3530134920131016		18015212	19561001	1	20131016	8
35354779	20121023	16905819	19530125	1	20121023	8
35400227	20120713	16604416	19661213	2	20120713	9
35434174	20110807	15600590	19470508	1	20110807	10
35487913	20120308	16222867	19490828	2	20120308	7
35488938	20120130	16097949	19651017	2	20120130	9
35517158	20110523	15379062	19901226	2	20110523	8
35536835	20120903	16750479	19770826	1	20120903	10
35561912	20111022	15819847	19521026	1	20111022	8
35609202	20120223	16177879	19340112	2	20120223	8
35794504	20120424	16357843	19620927	1	20120424	10
35823484	20120717	16613953	19630629	1	20120717	10
35843835	20110429	15306381	19681031	2	20110429	8
35915652	20120511	16414521	19320424	1	20120511	8
35926842	20110519	15370669	19841201	1	20110519	10
35946022	20111222	16000922	19481112	2	20111222	7
3595981020130206		17232299	19471110	1	20130206	8
35988444	20110410	15250117	19481129	1	20110410	10
35994184	20111116	15893931	19570607	1	20111116	9
36028816	20120427	16367733	19500906	1	20120427	7
36059195	20110718	15542614	19590105	1	20110718	10
36073742	20110312	15172245	19951016	2	20110312	10
3611784720130415		17435987	19540903	1	20130415	7
36151016	20110827	15658511	19620609	1	20110827	10
3616578320130715		17720295	19500628	1	20130715	8
3620194220130219		17260051	19360122	1	20130219	7
3632546920130308		17321591	19330724	2	20130308	9
36325947	20110504	15321132	19840305	1	20110504	10
36333150	20110628	15480333	19530612	1	20110628	10
36345810	20111014	15797423	19560510	1	20111014	8
36356464	20120405	16303496	19541214	1	20120405	10
36374240	20111202	15940780	19660119	1	20111202	10
36405604	20110821	15641333	19770712	2	20110821	8
36433735	20111114	15883938	19250429	1	20111114	8
36453404	20120927	16827013	19591118	2	20120927	10
36456061	20110407	15242379	19981028	2	20110407	8
36524455	20110803	15589289	19830603	2	20110803	10
36543198	20110402	15228515	19520422	1	20110402	8
3656038020130110		17152549	19540907	2	20130110	8
36617160	20110923	15736210	19520111	2	20110923	8
36692587	20110507	15333117	19620215	2	20110507	7
36733105	20110929	15750350	19470205	1	20110929	8
3674650620130415		17435648	19670102	1	20130415	7
36793665	20110627	15477248	19351206	2	20110627	8
36796335	20120218	16163555	19610701	2	20120218	10
36802274	20121210	17057779	19600301	1	20121210	8
3680242320130508		17508455	19360127	2	20130508	9
36876547	20110513	15354674	19500314	1	20110513	9
36927261	20110513	15353698	19670610	2	20110513	8

36983296	20120819	16711689	19560726	2	20120819	10
36994420	20110320	15192391	19551201	1	20110320	8
36994500	20120216	16159138	19310910	1	20120216	7
37002770	20110506	15332805	19390505	2	20110506	10
3700317120130826		17848374	19490521	1	20130826	7
37007640	20110919	15721658	19510111	1	20110919	10
37013788	20110414	15267495	19661010	2	20110414	7
3702195720130912		17911669	19660802	2	20130912	7
3706639020130418		17447576	19530524	1	20130418	9
37076703	20120211	16142658	19520515	2	20120211	8
37079473	20110427	15299317	19751222	1	20110427	9
37165370	20110312	15172112	19400615	1	20110312	10
37172886	20110614	15442609	19720925	2	20110614	10
37175749	20111101	15846547	19870731	2	20111101	8
3720254320130705		17689992	19391104	2	20130705	8
37257560	20111228	16015569	19860329	1	20111228	7
37272096	20110530	15395204	19591113	1	20110530	8
37287380	20110415	15270386	20001222	1	20110415	8
37292812	20120103	16028785	19470608	1	20120103	10
37312520	20110514	15356307	19390822	2	20110514	10
37314457	20110522	15375389	19551020	1	20110522	8
37324917	20110810	15610937	19510613	2	20110810	7
37359049	20111201	15936494	19660314	1	20111201	7
3737345820130916		17921990	19780212	2	20130916	7
37374279	20111221	15998070	19481004	1	20111221	7
37407435	20110627	15477147	19580128	2	20110627	10
37436127	20110221	15110375	19640221	2	20110221	7
37502899	20110913	15706662	19631201	2	20110913	8
37506302	20110729	15573173	19660715	1	20110729	8
37512235	20110607	15419899	19720118	1	20110607	7
3751945220130920		17931729	19450811	1	20130920	8
37536575	20111005	15771435	19530525	2	20111005	7
3754744720131223		18231848	19380102	2	20131223	7
37556379	20111107	15866786	19680215	1	20111107	8
37565927	20111013	15793935	19370308	1	20111013	8
37587501	20120117	16074921	19591012	1	20120117	7
37698183	20120621	16534272	19560321	1	20120621	10
37710331	20110921	15730469	19610803	1	20110921	7
3774782320131226		18240827	19630502	1	20131226	9
37749738	20110729	15572456	19751215	1	20110729	8
37759732	20110526	15387848	19840806	1	20110526	8
37791552	20111102	15851765	19470902	1	20111102	8
37793241	20121011	16870514	19680801	2	20121011	7
37820329	20110625	15473312	19541110	2	20110625	10
37832352	20110405	15233449	19421223	1	20110405	7
37835420	20110907	15692397	19540108	2	20110907	10
37841206	20121105	16942855	19720214	2	20121105	7
37850649	20110505	15329087	19510601	2	20110505	10
37853353	20110512	15350818	19521208	1	20110512	10
37905638	20120614	16514668	19281113	1	20120614	7
37912713	20110424	15292562	19740717	1	20110424	10
37923732	20110413	15262880	19650318	1	20110413	7
37926684	20111216	15984587	19590520	1	20111216	7
37927734	20120825	16729229	19611120	2	20120825	8
3793669920130509		17512733	19421128	2	20130509	9

37943150	20121205	17041435	19450425	1	20121205	10
37946068	20110711	15521440	19620108	1	20110711	7
37967070	20130217	17250438	19650608	1	20130217	8
37982573	20110420	15284351	19810902	2	20110420	7
37982959	20110613	15438939	19560408	2	20110613	8
37993003	20110601	15403049	19660115	2	20110601	7
37993503	20120515	16425223	19580505	2	20120515	7
37994857	20110619	15455712	19661020	1	20110619	8
38002107	20120531	16465988	19590427	1	20120531	10
38008489	20110807	15601027	19681205	1	20110807	7
38024496	20111108	15869165	19630924	1	20111108	7
38035733	20111208	15960347	19770922	2	20111208	10
38041279	20130821	17838516	19541124	1	20130821	9
38046730	20110414	15267518	19920219	1	20110414	9
38110031	20111212	15966985	19600724	2	20111212	8
38110097	20111007	15780205	19630723	2	20111007	10
38110542	20110805	15598257	19571031	2	20110805	7
38111272	20110509	15337149	19520410	1	20110509	8
38121903	20121014	16878450	19750615	1	20121014	10
38123307	20120531	16465405	19500110	1	20120531	7
38129883	20120514	16420893	19441127	1	20120514	8
38131850	20110608	15422963	19660326	1	20110608	10
38132002	20111001	15755332	19431118	2	20111001	7
38135181	20110509	15339123	19510828	1	20110509	8
38148219	20110919	15723026	19451102	1	20110919	10
38156160	20111026	15830019	19380414	1	20111026	8
38158600	20120528	16458020	19461111	2	20120528	7
38161409	20110822	15644569	19960430	2	20110822	8
38171505	20110531	15396673	19650910	1	20110531	10
38179601	20120329	16278485	19481001	1	20120329	10
38181805	20120314	16241601	19410610	1	20120314	9
38182557	20121113	16971807	19690628	2	20121113	8
38183889	20111231	16021974	19440925	1	20111231	7
38190851	20111014	15798694	19420102	1	20111014	10
38195663	20110526	15387726	19780315	2	20110526	9
38197192	20111126	15921424	19920321	1	20111126	8
38202607	20120627	16548187	19560601	1	20120627	10
38217402	20110918	15719261	19480126	1	20110918	8
38219997	20110929	15750831	19330915	2	20110929	8
38230330	20110522	15375553	19591115	2	20110522	10
38232063	20110619	15455933	19480131	2	20110619	10
38241291	20111018	15808549	19390712	1	20111018	10
38242670	20120301	16196304	19480623	2	20120301	7
38262338	20111019	15811325	19841120	1	20111019	10
38262601	20130328	17378011	19390408	2	20130328	8
38267673	20120718	16618269	19920109	2	20120718	8
38269146	20110905	15683064	19900308	2	20110905	8
38270949	20110813	15621793	19410922	1	20110813	9
38273482	20111204	15943269	19620821	2	20111204	7
38274827	20120310	16228994	19551021	1	20120310	7
38292545	20111221	15997449	19970326	1	20111221	10
38311852	20111023	15820227	19550827	1	20111023	7
38315058	20130523	17554278	19620916	2	20130523	7
38323272	20120330	16283705	19380922	1	20120330	10
38343021	20121231	17116468	19460527	1	20121231	8

38347987	20110728	15570850	19480710	2	20110728	8
38375438	20111102	15851357	19611201	1	20111102	10
38386593	20111018	15808078	19540816	1	20111018	7
38401693	20110916	15717103	19680606	2	20110916	7
38401955	20120308	16222972	19840613	1	20120308	10
38402765	20111120	15903570	19450306	1	20111120	8
38416067	20111227	16012592	19541010	1	20111227	7
38416669	20120117	16077024	19600920	1	20120117	9
38426312	20111108	15869290	19640105	1	20111108	10
38436736	20110928	15747145	19580502	1	20110928	8
38480078	20111106	15862535	19860622	2	20111106	7
38486907	20110821	15641050	19950929	2	20110821	10
3848996220130219		17257553	19260725	1	20130219	7
38490969	20120128	16094204	19690103	2	20120128	8
38498305	20120411	16323837	19421015	2	20120411	7
38508951	20111103	15854595	19661031	1	20111103	9
3852850620130524		17557287	19510802	2	20130524	7
38532999	20120710	16592643	19620213	1	20120710	8
38564691	20120827	16732117	19750203	1	20120827	7
38579394	20120116	16069938	19641218	1	20120116	10
38586559	20120323	16265255	19541105	2	20120323	8
38586640	20120924	16817671	19740501	1	20120924	8
38588657	20120320	16257529	19391210	1	20120320	9
3860324620130805		17785471	19681224	1	20130805	8
38618132	20120723	16629913	19270405	1	20120723	8
38618405	20111210	15965790	19680612	1	20111210	7
38625922	20111123	15910753	19850413	1	20111123	10
38629231	20121002	16841792	19540830	1	20121002	8
38642181	20120306	16214152	19510823	2	20120306	7
38650203	20120315	16245209	19601010	1	20120315	8
38656643	20120113	16063990	19650211	2	20120113	8
38656870	20120127	16092062	19660808	2	20120127	10
38662656	20111215	15981280	19650308	2	20111215	8
38669986	20120202	16110933	19460615	1	20120202	10
3868781920130920		17932158	19530202	2	20130920	9
38710940	20111220	15993522	19521003	1	20111220	8
38749687	20120314	16240313	19560715	1	20120314	10
38760064	20120619	16528182	19441222	1	20120619	7
38762297	20120321	16260685	19620315	1	20120321	10
38763483	20120114	16069225	19940708	2	20120114	7
3876595620131014		18006410	19620401	1	20131014	8
38767838	20120202	16109138	19610520	1	20120202	7
38772779	20120130	16097339	19820627	2	20120130	10
38779963	20120701	16557024	19590507	1	20120701	8
38790179	20120316	16248397	19440708	1	20120316	7
38802469	20120327	16275197	19620202	1	20120327	8
38808796	20120609	16499680	19470908	1	20120609	9
38814301	20120103	16028207	19771113	2	20120103	8
3882767720131006		17982102	19631114	1	20131006	8
38836941	20120313	16234044	19920612	2	20120313	7
38848576	20121023	16906230	19500713	1	20121023	8
38849853	20120117	16076820	19651216	1	20120117	7
38850769	20120719	16621548	19560802	2	20120719	8
38855297	20120331	16284984	19510618	2	20120331	10
38862394	20120719	16620682	19720911	1	20120719	8

38877735	20120326	16271381	19741128	1	20120326	8
38878103	20120522	16442428	19431207	1	20120522	7
38879468	20120619	16528088	19410419	1	20120619	8
38891053	20120609	16499546	19430413	1	20120609	8
38897653	20120822	16721776	19701122	1	20120822	10
38938748	20120423	16354184	19640403	1	20120423	8
38943509	20130117	17174047	19410523	1	20130117	8
38953898	20120330	16283457	19640824	2	20120330	8
38958406	20120507	16398630	19540505	1	20120507	9
38980733	20130812	17809107	19730828	2	20130812	8
38984973	20120901	16746252	19340204	1	20120901	7
38993521	20120513	16417552	19640328	1	20120513	8
39003366	20120720	16624008	19650115	1	20120720	8
39012196	20120815	16702928	19850713	2	20120815	7
39016405	20121106	16947903	19900523	1	20121106	8
39048630	20130715	17720465	19730911	1	20130715	8
39050629	20120624	16538002	19320904	1	20120624	7
39051199	20120403	16295135	19680418	2	20120403	7
39069846	20120710	16592857	19670917	2	20120710	8
39072372	20120520	16436947	19571002	1	20120520	8
39086823	20120611	16503708	19690110	1	20120611	7
39094718	20120911	16779296	19691123	1	20120911	10
39097411	20120708	16584567	20100922	2	20120708	8
39110082	20120719	16621477	19540810	1	20120719	9
39139463	20130311	17328188	19491210	1	20130311	7
39151570	20120711	16596530	19710809	1	20120711	9
39155185	20120819	16711837	19430101	1	20120819	8
39155298	20130822	17842142	19480205	1	20130822	7
39165849	20120719	16621329	19630911	2	20120719	10
39178693	20120515	16423233	19550415	1	20120515	7
39180911	20130223	17273669	19590617	1	20130223	8
39181516	20120714	16606307	19660321	1	20120714	8
39187514	20121012	16874388	19611205	1	20121012	10
39193663	20120703	16568362	19520608	1	20120703	9
39201451	20120615	16517466	19740109	2	20120615	7
39204869	20130929	17954673	19470520	1	20130929	7
39205748	20120723	16630735	19630903	1	20120723	10
39214590	20121022	16901180	19591104	1	20121022	10
39216665	20120801	16656602	19660608	1	20120801	7
39218172	20121216	17077366	19990220	1	20121216	8
39227582	20130416	17440279	19410411	1	20130416	7
39255146	20130914	17916899	19530210	2	20130914	7
39266416	20120813	16695009	19551010	1	20120813	10
39278256	20121004	16850536	19570701	1	20121004	8
39287166	20121012	16874590	19490613	2	20121012	8
39308606	20120910	16777302	19660825	1	20120910	7
39315509	20121009	16867512	19500220	1	20121009	7
39355094	20121104	16938804	19510314	2	20121104	10
39359289	20120923	16814581	19400716	1	20120923	10
39367505	20130115	17167166	19510513	2	20130115	7
39389827	20130610	17610376	19540603	2	20130610	8
39392693	20121207	17050296	19530804	1	20121207	8
39403628	20121108	16957014	19640523	1	20121108	8
39417000	20130925	17945042	19600822	1	20130925	8
39476909	20121016	16886177	19780917	1	20121016	9

39518871	20130128	17203451	19530520	1	20130128	8
39520860	20130402	17395098	19541002	1	20130402	9
39541305	20121220	17092665	19970728	1	20121220	8
39566400	20130119	17180135	19491002	1	20130119	9
39566502	20130220	17262676	19451025	1	20130220	8
39587729	20130123	17191770	19600704	1	20130123	7
39589316	20131104	18071922	19660522	1	20131104	7
39597825	20130511	17518711	19910529	2	20130511	7
39612254	20131117	18118188	19600625	1	20131117	8
39626125	20130116	17171400	20031113	2	20130116	9
39632047	20130102	17123097	19790808	2	20130102	8
39639617	20121211	17062251	19640415	1	20121211	8
39642198	20131105	18076608	19580607	1	20131105	7
39645039	20130325	17369847	19611119	2	20130325	9
39645233	20130916	17921419	19590115	1	20130916	7
39647080	20130312	17332639	19320323	2	20130312	8
39667384	20130226	17281803	19680310	1	20130226	7
39680698	20130924	17941909	19610319	2	20130924	8
39693168	20130402	17395863	20001031	1	20130402	9
39724328	20130513	17523193	19651105	2	20130513	8
39736602	20130513	17523337	19590710	1	20130513	7
39746786	20130517	17538928	19490107	2	20130517	8
39768326	20130210	17242549	19800223	2	20130210	7
39769636	20131002	17969748	19440718	1	20131002	9
39808065	20130416	17437402	19940630	1	20130416	9
39892767	20130423	17458607	19600903	1	20130423	7
39906893	20130907	17893542	19550102	1	20130907	7
39910582	20130505	17496263	19601006	2	20130505	8
39923450	20130618	17633398	19510102	1	20130618	7
39937912	20130504	17495515	19590628	2	20130504	8
39941087	20131216	18212152	19620120	1	20131216	8
39969636	20131206	18181471	19580820	2	20131206	7
39979403	20130517	17537231	19660125	1	20130517	8
39995590	20130903	17876245	19590808	1	20130903	7
40011936	20130630	17665921	19880207	2	20130630	8
40015541	20130814	17818543	19660820	2	20130814	7
40019736	20130702	17677123	19540828	1	20130702	8
40046535	20130917	17924184	19700425	2	20130917	8
40055138	20130709	17702458	19520401	2	20130709	8
40065494	20131027	18046140	19641029	2	20131027	8
40066624	20130705	17688932	19411020	1	20130705	7
40069076	20130723	17745834	19590612	1	20130723	8
40082642	20130724	17747492	19610525	1	20130724	9
40153706	20130627	17660787	19631124	1	20130627	8
40178016	20130807	17793922	19850301	2	20130807	8
40187664	20131105	18074552	19670426	1	20131105	7
40189353	20131116	18117731	19591201	1	20131116	8
40201125	20130812	17811282	19650620	1	20130812	9
40209403	20130929	17954773	19340605	1	20130929	8
40235572	20130805	17782115	19611031	1	20130805	8
40247550	20131115	18116128	19480421	1	20131115	7
40256948	20131120	18129581	19610324	2	20131120	9
40272546	20130903	17875335	19610917	1	20130903	8
40273925	20130917	17923037	19630324	1	20130917	9
40296524	20131030	18055656	19590109	2	20131030	7

40299125	20131016	18015172	19901001	1	20131016	8
40309033	20130830	17862699	19630312	2	20130830	8
40388725	20131125	18141499	19780612	1	20131125	8
40417376	20130924	17939243	19431128	1	20130924	7
40432824	20131001	17964597	19980126	2	20131001	7
40436451	20131106	18083169	19630110	2	20131106	7
40449705	20130910	17902739	19411111	1	20130910	7
40481592	20131119	18125546	19621022	2	20131119	8
40482357	20131026	18045507	19540910	1	20131026	8
40484308	20131203	18168223	19650330	1	20131203	7
40485914	20131023	18035844	19511230	1	20131023	8
40613283	20131119	18123658	19631129	2	20131119	8
81630	20130328	17379428	19290916	1	20130328	7
235030	20110904	15678814	19310514	1	20110904	10
759706	20131031	18058291	19330717	1	20131031	7
858526	20120911	16781149	19271017	1	20120911	7
2307837	20120416	16336977	19480612	2	20120416	7
2587782	20120906	16766249	19500928	2	20120906	8
3334756	20120220	16167505	19600320	1	20120220	8
3514792	20110425	15293982	19620307	2	20110425	10
3696911	20120221	16171823	19370220	1	20120221	7
3854668	20110802	15584533	19280901	1	20110802	10
3991853	20110613	15438941	19481001	2	20110613	7
4180794	20130924	17941683	19300624	1	20130924	7
4520165	20130912	17910144	19320815	1	20130912	8
5265634	20121111	16963638	19590405	2	20121111	7
5542381	20130109	17149505	19260506	2	20130109	8
6280882	20130706	17693253	19360822	2	20130706	8
6343437	20120612	16508033	19670304	2	20120612	8
6409887	20110627	15476347	19490415	1	20110627	7
6456855	20110523	15375966	19251215	1	20110523	10
7066093	20120314	16241699	19490302	1	20120314	7
7452439	20121215	17077001	19760602	1	20121215	7
7455096	20110607	15419328	19260101	1	20110607	10
7567820	20120624	16537912	19570304	1	20120624	8
7790810	20110629	15482036	19270510	1	20110629	9
8012906	20110704	15498729	19630608	2	20110704	8
8383548	20130428	17471787	19451207	1	20130428	8
8958938	20120309	16226245	19250820	1	20120309	7
9469141	20130430	17478377	19310626	2	20130430	8
9682988	20130921	17933426	19350201	2	20130921	7
9685998	20130806	17790123	19310301	1	20130806	8
9922252	20110914	15709495	19611027	1	20110914	7
10420765	20120409	16315773	19411212	2	20120409	7
10434727	20120823	16725197	19441231	1	20120823	9
10917109	20131212	18200599	19550509	1	20131212	8
11045117	20120606	16489010	19430211	1	20120606	7
11050127	20130115	17167169	19561031	2	20130115	8
11554522	20120725	16635919	19250607	2	20120725	8
11598417	20121223	17097298	19381213	2	20121223	7
11752435	20120713	16604374	19410405	2	20120713	10
12110097	20120315	16243918	19510623	2	20120315	8
12154471	20110608	15423816	19511028	1	20110608	7
12365683	20120506	16394918	19570728	1	20120506	7
12380642	20120305	16208882	19360205	2	20120305	7

12441348	20120602	16474247	19680417	1	20120602	9
12481468	20130719	17735949	19531020	2	20130719	8
12550220	20110623	15468684	19551014	2	20110623	8
12639848	20131001	17959903	19551028	2	20131001	8
12757721	20110730	15574677	19490201	1	20110730	10
13287440	20130124	17195038	19271224	2	20130124	8
13302631	20120806	16670230	19420528	2	20120806	7
13374895	20120429	16369398	19411018	1	20120429	10
13574431	20120127	16093026	19550122	1	20120127	9
13627468	20111208	15960068	19421123	1	20111208	8
13649600	20120917	16798131	19490719	1	20120917	7
13897342	20130222	17271561	19570806	1	20130222	7
14235122	20111102	15851817	19521031	1	20111102	7
14506117	20111129	15928227	19591020	1	20111129	8
14823155	20110718	15543121	19630729	2	20110718	10
14863219	20110913	15706655	19481214	2	20110913	7
14961514	20110612	15434838	19441208	1	20110612	8
15657326	20121010	16867858	19340416	2	20121010	8
16002736	20120301	16194767	19490310	1	20120301	10
16272881	20111206	15949377	19460707	2	20111206	8
16303938	20130113	17159577	19470901	1	20130113	7
16472154	20110815	15624791	19270304	2	20110815	7
16798064	20111221	15997902	19500701	1	20111221	10
17049682	20111004	15764813	19740813	1	20111004	10
17324639	20130331	17383553	19320229	1	20130331	8
17366824	20120814	16697096	19350301	1	20120814	7
17489508	20110829	15661587	19581112	1	20110829	8
17517043	20110611	15434180	19680730	2	20110611	10
17518808	20120719	16621635	19621025	1	20120719	7
18006309	20121220	17092595	19550302	2	20121220	7
18196800	20111102	15849341	19270707	1	20111102	7
18493282	20121119	16989113	19380104	1	20121119	8
18608043	20130421	17453123	19651118	2	20130421	7
18785938	20120522	16442916	19430916	1	20120522	8
18799934	20130614	17623249	19560120	2	20130614	9
18861693	20121027	16916778	19570909	1	20121027	9
18986135	20120910	16777253	19580506	1	20120910	7
18995614	20110621	15463098	19270101	2	20110621	7
19044054	20110916	15717098	19620414	1	20110916	10
19439986	20120611	16504190	19501212	1	20120611	10
19542406	20111006	15776043	19400312	2	20111006	7
19684801	20130929	17954741	19700601	1	20130929	7
19928940	20120130	16097471	19320508	1	20120130	7
19937963	20120906	16765349	19640315	2	20120906	7
19943716	20130617	17629466	19460224	1	20130617	8
20050026	20121130	17022196	19630102	2	20121130	7
20216084	20110409	15249421	19560809	2	20110409	8
20245914	20121013	16877720	19600728	1	20121013	9
20547799	20120510	16411170	19300514	1	20120510	10
20902130	20111114	15887138	19550205	1	20111114	10
21084297	20130616	17625441	19610903	1	20130616	8
21516610	20121119	16989779	19751002	1	20121119	7
21655958	20130311	17327900	19601105	1	20130311	7
21743680	20121120	16993936	19550712	2	20121120	10
21951688	20120227	16185569	19620126	1	20120227	8

22034102	20120326	16270005	19260113	1	20120326	10
22428337	20121011	16871058	19250129	2	20121011	7
22863565	20130326	17373235	19541020	1	20130326	8
22896884	20120110	16056003	19261222	1	20120110	8
22932645	20120501	16379210	19630120	2	20120501	7
22989340	20111205	15948150	19471110	1	20111205	10
23084653	20120524	16450170	19680118	2	20120524	8
23265647	20120217	16161292	19361120	1	20120217	8
23338854	20110520	15373740	19310101	1	20110520	8
23512494	20131114	18112440	19481101	2	20131114	7
23601265	20111201	15935603	19580623	1	20111201	10
23886999	20120604	16477493	19510528	1	20120604	7
24146836	20120322	16263760	19401014	2	20120322	10
24459776	20120306	16214573	19740226	1	20120306	8
24596585	20130124	17195124	19610907	2	20130124	8
24707333	20131022	18032710	19581115	1	20131022	7
24885801	20111031	15840839	19631125	1	20111031	7
24896671	20120623	16537566	19430417	1	20120623	8
25074253	20110805	15598700	19570802	2	20110805	8
25154078	20111127	15921732	19290903	2	20111127	8
25156869	20111207	15957217	19530724	2	20111207	7
25208768	20120229	16190221	19440131	1	20120229	8
25351262	20121120	16994204	19580523	2	20121120	7
25445583	20110925	15738245	19620116	1	20110925	7
25445798	20130726	17754776	19550331	1	20130726	7
25601121	20110626	15473768	19510906	1	20110626	8
25624913	20131109	18094595	19240808	2	20131109	7
25718698	20130805	17786302	19661022	1	20130805	7
25744847	20120422	16351816	19630708	2	20120422	10
25810597	20111107	15866791	19631121	2	20111107	7
26007567	20111204	15943272	19700514	1	20111204	7
26062748	20111115	15891024	19680517	1	20111115	7
26068928	20120220	16166890	19920803	1	20120220	10
26204922	20130130	17208650	19331209	1	20130130	9
26391968	20110513	15354139	19550801	2	20110513	9
26958645	20111027	15832401	19411020	1	20111027	10
27259047	20120504	16392731	19530306	1	20120504	10
27286857	20120804	16664319	19590820	2	20120804	8
27570963	20120311	16229697	19400604	1	20120311	10
27878440	20110905	15681726	19361115	2	20110905	8
27912498	20111019	15811357	19330206	2	20111019	9
28042559	20131204	18172568	19500718	1	20131204	8
28170345	20111214	15977269	19460520	1	20111214	8
28375522	20120125	16089828	19561216	1	20120125	10
28885974	20120907	16769495	19571124	1	20120907	10
28952536	20111212	15970701	19450122	2	20111212	10
28989788	20110411	15253502	19800702	1	20110411	10
29032700	20130418	17447360	19390828	1	20130418	9
29258079	20120916	16794627	19370122	1	20120916	7
29388816	20120516	16428766	19860429	1	20120516	8
29410988	20121026	16915290	19700831	2	20121026	8
29776214	20130213	17244225	19300101	2	20130213	8
29940010	20131205	18178251	19451113	2	20131205	8
29987357	20121201	17024257	19360221	1	20121201	9
29998672	20111225	16005683	19520519	1	20111225	8

30104166	20111216	15985024	19490217	2	20111216	10
30110179	20130125	17198098	19610804	1	20130125	8
30160384	20110728	15569802	19701017	1	20110728	8
30295217	20120920	16809159	19611120	1	20120920	10
30388295	20120126	16090868	19540702	1	20120126	8
30433591	20111215	15980883	19470424	1	20111215	7
30587258	20111218	15987060	19621203	1	20111218	9
30631040	20110828	15659145	19990915	1	20110828	8
30724255	20120723	16630629	19610906	1	20120723	8
30740488	20120215	16155828	19600206	2	20120215	8
30798959	20131010	17997192	19271015	1	20131010	7
31058249	20110420	15284340	19751219	2	20110420	9
31214865	20120303	16203137	19451112	2	20120303	10
31254758	20121222	17097009	19590218	1	20121222	7
31429319	20130901	17865307	19590711	1	20130901	8
31430418	20110903	15678486	19461125	1	20110903	8
31550513	20110620	15459811	19580316	2	20110620	8
31567187	20130731	17767200	19681015	1	20130731	8
31678418	20110830	15664286	19570616	1	20110830	8
31819348	20120609	16499245	19451212	1	20120609	10
31981336	20120522	16442554	19630522	1	20120522	8
32013711	20110808	15601647	19760101	2	20110808	8
32061044	20131227	18244049	19360322	1	20131227	7
32105047	20120410	16319453	19550510	1	20120410	9
32206078	20120413	16330966	19600805	1	20120413	10
32475437	20110803	15589170	19581020	1	20110803	10
32477955	20120503	16387875	19341202	2	20120503	7
32537421	20130802	17776002	19591104	1	20130802	7
32696212	20131020	18025547	19690914	1	20131020	8
32765143	20130601	17577943	19790815	1	20130601	8
32797481	20120406	16308379	19510302	1	20120406	8
32811180	20130115	17168135	19640818	2	20130115	9
32934682	20110519	15370715	19500311	2	20110519	8
32973141	20121126	17008793	19710629	1	20121126	7
33024838	20130404	17402092	19390614	1	20130404	7
33038083	20130312	17331227	19410616	1	20130312	9
33067048	20110924	15737985	19550505	1	20110924	10
33089939	20120301	16193860	19480106	2	20120301	8
33101347	20130327	17376211	19500929	1	20130327	8
33162264	20120601	16470146	19650104	1	20120601	8
33256814	20111017	15802805	19550913	1	20111017	8
33487795	20110508	15335161	19441221	2	20110508	9
33490389	20111220	15994534	19590320	2	20111220	7
33559034	20131014	18007742	19430519	1	20131014	8
33767532	20121004	16851789	19571022	1	20121004	8
34042563	20130822	17842070	19680706	1	20130822	7
34047875	20111021	15818410	19531118	1	20111021	7
34198599	20130115	17166180	19470401	1	20130115	8
34347154	20131010	17997141	19510509	1	20131010	7
34435182	20120202	16112533	19240412	2	20120202	8
34570600	20120317	16249272	19431021	1	20120317	7
34665991	20131012	18002712	19740705	2	20131012	7
34747154	20120412	16326363	19500112	1	20120412	9
34961427	20120826	16729527	19600215	1	20120826	7
35132026	20120115	16069453	19500219	1	20120115	8

35245455	20110522	15375751	19600815	1	20110522	7
3528527920130501		17482886	19650721	2	20130501	7
3540521120130118		17176799	19531028	1	20130118	7
35434174	20110902	15675376	19470508	1	20110902	10
35448976	20111113	15883185	19640122	1	20111113	7
35455108	20110410	15250035	19481120	2	20110410	8
35465964	20121109	16960319	19441130	1	20121109	8
35488938	20120603	16474667	19651017	2	20120603	10
35745790	20110501	15309423	19610118	2	20110501	7
35759398	20110902	15676230	19440202	1	20110902	7
35823484	20120902	16747145	19630629	1	20120902	9
35926842	20110720	15549435	19841201	1	20110720	10
3595282020131219		18222076	19491206	2	20131219	9
35994184	20111222	16000636	19570607	1	20111222	10
3600115720130107		17138694	19791019	2	20130107	8
3605022320130913		17913224	19440111	2	20130913	7
3615048820130326		17373095	19651120	1	20130326	8
36324308	20110816	15629366	19970201	2	20110816	8
36333150	20111110	15877457	19530612	1	20111110	8
36345810	20111110	15877369	19560510	1	20111110	8
36356464	20120706	16580782	19541214	1	20120706	7
36374240	20120130	16098261	19660119	1	20120130	10
36405604	20110903	15678335	19770712	2	20110903	9
36406890	20120808	16677797	19430307	1	20120808	10
36454725	20110617	15452277	19621023	1	20110617	8
36543198	20110420	15284344	19520422	1	20110420	8
3655532520131028		18048228	19380110	1	20131028	8
36563301	20111127	15921974	19481129	1	20111127	8
36671531	20121022	16902953	19520226	1	20121022	7
3673719620130308		17321379	19500118	2	20130308	8
36776268	20110924	15736732	20000607	1	20110924	7
3679492020130204		17225822	19340318	2	20130204	7
36811968	20120701	16557029	19480915	2	20120701	8
36813680	20110609	15428886	19430720	2	20110609	8
36927261	20110623	15468728	19670610	2	20110623	7
36979154	20111019	15810033	19460501	1	20111019	7
36980888	20110512	15350477	19561215	1	20110512	10
36986035	20120827	16732734	19650327	1	20120827	8
36989590	20120725	16637561	19570213	1	20120725	10
3700317120130926		17949365	19490521	1	20130926	7
37052236	20120129	16094672	19640331	2	20120129	10
3706536420131114		18111802	19551206	1	20131114	8
3710521820131210		18194143	19630102	2	20131210	7
37124008	20110805	15598666	19500518	2	20110805	8
37127698	20120425	16361190	19580112	1	20120425	8
3715978720130904		17881215	19580719	1	20130904	9
3716235920121219		17088999	19620511	2	20121219	7
37165370	20110421	15286147	19400615	1	20110421	10
37175749	20111111	15879904	19870731	2	20111111	8
3725648720130407		17407378	19461020	1	20130407	9
37366895	20120213	16147536	19631021	2	20120213	10
37372295	20111212	15969184	19371204	2	20111212	10
37372853	20110417	15272751	19471010	1	20110417	10
3737345820131010		17997025	19780212	2	20131010	8
37374279	20120110	16056179	19481004	1	20120110	7

3741928620130331		17383932	19571222	1	20130331	8
3746575120130505		17496052	19481220	2	20130505	8
37468012	20121129	17018465	19520122	1	20121129	8
37486070	20110727	15567690	19460202	1	20110727	10
37493020	20110829	15660936	20050720	1	20110829	8
37527529	20120816	16706324	19780626	1	20120816	9
37556379	20111208	15960748	19680215	1	20111208	9
37567876	20110504	15324515	19960817	1	20110504	10
37584477	20110807	15600627	19560827	1	20110807	8
3759504320130714		17715817	19430802	1	20130714	8
37621026	20110502	15315259	19770117	1	20110502	8
37663608	20110628	15479397	19521215	1	20110628	10
37669479	20110531	15396838	19490701	1	20110531	8
37698183	20120814	16699158	19560321	1	20120814	9
37743423	20111211	15966462	19700202	1	20111211	7
3774947620130322		17362942	19520110	1	20130322	7
37749738	20110809	15608086	19751215	1	20110809	7
37750086	20110624	15472290	19481120	1	20110624	9
37759732	20120216	16158484	19840806	1	20120216	8
37820329	20110728	15570764	19541110	2	20110728	10
37824730	20121026	16915007	19370104	1	20121026	7
37829848	20111123	15913897	19611230	1	20111123	10
37832352	20110913	15705457	19421223	1	20110913	7
37835748	20120925	16819711	19910713	1	20120925	7
37857300	20111220	15992683	19590705	1	20111220	7
37858290	20120201	16106092	19521210	2	20120201	8
37858507	20110428	15305164	19541115	2	20110428	7
37865251	20110607	15419872	20091203	1	20110607	10
37887517	20110916	15717112	19420624	1	20110916	8
37891637	20110609	15428866	19420222	1	20110609	8
37896698	20111025	15826086	19570104	1	20111025	8
37912713	20110512	15351544	19740717	1	20110512	8
37913852	20111014	15797521	19480727	2	20111014	8
37922853	20120115	16069809	19570722	1	20120115	8
37923732	20110519	15369866	19650318	1	20110519	7
37941392	20120311	16229681	19450708	1	20120311	8
37943536	20110509	15338868	19630212	1	20110509	7
37961925	20120424	16358848	19471111	2	20120424	7
3797704120131211		18199216	19751210	1	20131211	9
37991654	20110926	15741324	19551220	2	20110926	10
37998893	20110509	15339852	19660602	1	20110509	8
38002107	20120822	16722608	19590427	1	20120822	10
38005935	20110621	15462973	19931010	1	20110621	8
38032698	20110425	15295744	19681010	2	20110425	7
38035733	20111227	16010840	19770922	2	20111227	10
38036361	20120221	16171710	19481201	2	20120221	8
38046730	20110614	15443210	19920219	1	20110614	8
38054385	20120515	16423292	19690217	1	20120515	8
38066761	20110503	15319687	19490901	1	20110503	7
38077713	20110511	15347344	19580518	2	20110511	7
38093888	20110405	15233714	19821004	2	20110405	8
38098101	20110513	15354412	19490211	1	20110513	8
38110097	20111028	15836150	19630723	2	20111028	9
38113063	20110801	15580128	20001105	1	20110801	8
3811812620130329		17381119	19530805	2	20130329	7

38133992	20120612	16508065	19370720	1	20120612	8
38143963	20110526	15388125	19511017	2	20110526	7
38148219	20111028	15836061	19451102	1	20111028	8
38157969	20120709	16588846	19511014	1	20120709	9
38171505	20110824	15649770	19650910	1	20110824	10
38217402	20111004	15766916	19480126	1	20111004	8
38218881	20110927	15743583	19940317	1	20110927	10
38219997	20111221	15997792	19330915	2	20111221	8
38230330	20110711	15523102	19591115	2	20110711	8
38238072	20110718	15542602	19820408	1	20110718	8
38243651	20110918	15719290	19790218	1	20110918	8
38260536	20110927	15744973	19561118	1	20110927	10
3826260120130420		17452546	19390408	2	20130420	7
38269146	20111024	15823382	19900308	2	20111024	10
38274827	20120518	16434434	19551021	1	20120518	8
38295497	20110611	15434152	19511220	1	20110611	8
3831505820130906		17891450	19620916	2	20130906	8
3834302120130322		17363226	19460527	1	20130322	7
38361045	20111026	15830479	19530406	2	20111026	8
38401693	20111005	15771492	19680606	2	20111005	8
38402152	20110916	15717202	19591030	1	20110916	7
38416272	20111017	15802925	19760715	1	20111017	10
3841692120130618		17633794	19470309	2	20130618	8
38425922	20111024	15821898	20040112	1	20111024	10
38426312	20111130	15930503	19640105	1	20111130	8
38459960	20111103	15855676	19421117	1	20111103	8
3851856820130910		17900913	19680714	1	20130910	8
38520273	20111102	15850325	19690101	1	20111102	10
38532046	20121010	16867594	19590122	2	20121010	8
38558995	20120320	16257658	19441005	1	20120320	8
38567054	20111121	15907224	20090613	1	20111121	7
38580982	20120212	16143524	19770318	2	20120212	7
38586559	20120423	16355211	19541105	2	20120423	10
38589650	20120210	16140817	19920902	1	20120210	10
38617059	20120415	16333500	19630610	1	20120415	7
38625046	20111218	15987014	19350312	1	20111218	7
38625819	20120203	16116044	19571216	1	20120203	8
38626470	20120406	16307967	19371117	2	20120406	8
38626583	20120722	16627028	19781029	1	20120722	7
38637171	20121029	16920435	19351230	1	20121029	10
38651615	20111202	15939977	19380904	2	20111202	8
38652367	20120303	16203033	19290202	1	20120303	10
38660785	20120811	16690095	19660601	1	20120811	10
38684569	20120206	16123847	19511212	2	20120206	7
38707469	20120104	16035982	19481012	1	20120104	7
38737610	20120128	16094186	19490510	1	20120128	7
38752248	20120210	16137333	19430419	2	20120210	8
38763369	20120527	16454720	19620408	1	20120527	7
38763483	20120201	16107804	19940708	2	20120201	7
38767838	20120327	16273382	19610520	1	20120327	8
38772779	20120625	16541849	19820627	2	20120625	8
38783210	20111212	15969685	19590110	1	20111212	10
3878387820130122		17187035	19490320	1	20130122	7
38790179	20120331	16284967	19440708	1	20120331	7
38821351	20120919	16805966	19671125	2	20120919	8

38862394	20120917	16796730	19720911	1	20120917	8
38874316	20120408	16310652	19340708	1	20120408	7
38877735	20120712	16601032	19741128	1	20120712	10
38879811	20121125	17006433	19340205	2	20121125	8
38926088	20120410	16320072	19880520	1	20120410	8
38938748	20120521	16440186	19640403	1	20120521	10
38942755	20120503	16388822	19490922	1	20120503	8
38943509	20130210	17242362	19410523	1	20130210	8
38945652	20120822	16722478	19560102	1	20120822	10
38967805	20120731	16649951	19480329	2	20120731	9
38969607	20120528	16455881	19601203	1	20120528	7
38972291	20130202	17219924	19520420	1	20130202	8
38984973	20120914	16789219	19340204	1	20120914	10
39012196	20120926	16823346	19850713	2	20120926	8
39013053	20130719	17734748	19530727	1	20130719	8
39046521	20120726	16639471	19610320	1	20120726	8
39073160	20120917	16797824	19430829	1	20120917	8
39086823	20120625	16541354	19690110	1	20120625	8
39133136	20130528	17567355	19371108	1	20130528	8
39156677	20130109	17149516	19630725	1	20130109	7
39161370	20130319	17353085	19401125	1	20130319	7
39162793	20121002	16842491	19560719	1	20121002	10
39183965	20120807	16673685	20010529	2	20120807	10
39201451	20120629	16554095	19740109	2	20120629	7
39202545	20131009	17994966	19381126	1	20131009	8
39204869	20131103	18066831	19470520	1	20131103	8
39209784	20130624	17650941	19400628	2	20130624	8
39214089	20120817	16709459	19610503	1	20120817	7
39216665	20120901	16746534	19660608	1	20120901	8
39231486	20121016	16886106	19611211	1	20121016	7
39231793	20121225	17102617	19540620	2	20121225	7
39239957	20120831	16743277	19530526	1	20120831	8
39252136	20120912	16785186	19520115	1	20120912	7
39265480	20120906	16766844	19500502	1	20120906	10
39269197	20120910	16775097	19730920	2	20120910	7
39287166	20121103	16938257	19490613	2	20121103	10
39292712	20130222	17272171	19661031	1	20130222	8
39297579	20121025	16911361	19591010	1	20121025	8
39300088	20120730	16648233	19581101	2	20120730	7
39308606	20121120	16994120	19660825	1	20121120	10
39325887	20121108	16955555	19830818	2	20121108	8
39340222	20121114	16975869	19461015	2	20121114	8
39350408	20131001	17958301	19641206	1	20131001	7
39367505	20130207	17239055	19510513	2	20130207	7
39385836	20130912	17911446	19650408	2	20130912	7
39389827	20130715	17720331	19540603	2	20130715	8
39503521	20130131	17212176	19500426	1	20130131	8
39560991	20121205	17040796	19640928	1	20121205	9
39572242	20130215	17248666	19761226	2	20130215	9
39582360	20130120	17180634	19370709	1	20130120	8
39595169	20130406	17406840	19500826	1	20130406	8
39597825	20130607	17603096	19910529	2	20130607	8
39635728	20131106	18083777	19600721	1	20131106	8
39636470	20130514	17525146	19560125	1	20130514	7
39651086	20130424	17462464	19541101	1	20130424	8

3966571920130504	17495737	19750307	1	20130504	7
3966738420130322	17363746	19680310	1	20130322	8
3968689020130614	17622904	19641113	2	20130614	8
3971719620130502	17489102	19821121	1	20130502	9
3972432820130603	17582622	19651105	2	20130603	9
3972777020130408	17409024	19560502	1	20130408	8
3979597620130429	17474616	19530120	1	20130429	8
3986815020130505	17495861	19610816	1	20130505	7
3988389120130812	17809514	19740706	1	20130812	7
3990435320131023	18033627	19430330	1	20131023	8
3990630420130718	17732645	19940702	2	20130718	8
4000133020131017	18020334	19440922	1	20131017	8
4001546120130711	17711532	19761217	1	20130711	8
4001928120131001	17964132	19480528	1	20131001	7
4004653520131013	18003116	19700425	2	20131013	9
4004985220130522	17550463	19980702	1	20130522	7
4009022020130906	17890820	19850329	1	20130906	7
4013848520131127	18148904	19611226	1	20131127	7
4015205420130807	17793850	19541225	1	20130807	7
4018766420131222	18228251	19670426	1	20131222	7
4018935320131227	18243536	19591201	1	20131227	7
4018953520130706	17693331	19620704	1	20130706	7
4019234520131014	18007812	20101005	1	20131014	8
4026355620131022	18033589	19581107	2	20131022	8
4027515820130912	17910681	20000818	1	20130912	8
4031251420131129	18154916	19830120	1	20131129	8
4031369720130923	17935592	19590720	2	20130923	9
4041737620131014	18006209	19431128	1	20131014	7
4045263920131217	18216052	19490110	1	20131217	7
4048430820131216	18211256	19650330	1	20131216	7
232860 20121018	16892592	19281204	1	20121018	8
1700216 20120511	16414664	19250524	1	20120511	7
2251947 20120309	16226535	19491107	2	20120309	8
2307837 20120517	16431942	19480612	2	20120517	10
237162220130826	17850947	19260726	1	20130826	8
2624353 20120917	16798561	19300222	2	20120917	8
2832759 20121105	16943718	19250310	1	20121105	9
3891698 20120318	16250691	19250115	1	20120318	7
4752505 20120602	16473944	19250415	1	20120602	7
579585720130627	17660927	19371112	1	20130627	8
6261445 20121029	16919532	19521009	1	20121029	10
6343437 20120911	16780282	19670304	2	20120911	7
6766967 20111225	16005898	19560208	2	20111225	8
738940420130825	17847468	19240815	2	20130825	8
754164220131106	18083826	19520228	1	20131106	7
7790810 20111104	15859726	19270510	1	20111104	8
8578550 20120117	16076189	19400610	1	20120117	8
8666497 20120104	16037217	19410116	1	20120104	9
891632320131221	18227905	19470715	1	20131221	9
10074869 20110311	15170192	19330918	1	20110311	8
10411742 20111230	16020728	19450215	2	20111230	7
1070680620130410	17421273	19330601	1	20130410	7
11045117 20120628	16551988	19430211	1	20120628	7
11140822 20120205	16119730	19290505	1	20120205	9
1159841720130117	17175128	19381213	2	20130117	7

11722499	20111013	15795218	19370817	2	20111013	8
12110097	20120410	16320141	19510623	2	20120410	8
12110655	20130302	17296404	19521017	2	20130302	8
12219953	20130211	17242891	19630917	2	20130211	7
12683806	20110802	15585527	19520410	2	20110802	10
12974866	20120423	16354506	19421021	1	20120423	8
13019506	20120607	16493571	19580324	1	20120607	7
13431726	20121116	16982199	19400425	2	20121116	9
13574431	20120215	16155770	19550122	1	20120215	8
13604223	20110825	15654361	19380806	1	20110825	8
14538659	20111208	15960188	19281227	1	20111208	7
14600954	20120912	16785362	19500605	1	20120912	8
14823155	20110827	15658584	19630729	2	20110827	7
14897859	20131215	18208662	19280523	1	20131215	8
14961514	20110702	15493144	19441208	1	20110702	7
15171365	20130308	17321534	19460214	2	20130308	7
16766500	20110906	15688082	19390510	1	20110906	10
17049682	20111102	15849725	19740813	1	20111102	10
17049955	20120111	16060257	19591008	2	20120111	10
17366824	20121030	16921250	19350301	1	20121030	8
18103816	20120409	16315601	19360219	2	20120409	8
18196800	20120201	16104865	19270707	1	20120201	8
18749956	20110429	15306416	19341220	1	20110429	7
18793743	20111214	15975972	19330629	1	20111214	7
18799934	20130620	17641237	19560120	2	20130620	8
18986135	20121120	16993385	19580506	1	20121120	7
19044054	20111016	15800822	19620414	1	20111016	9
19048283	20121012	16875889	19280316	1	20121012	8
19195201	20130624	17650534	19420228	2	20130624	7
19445228	20120826	16729852	19370926	1	20120826	8
19542406	20111025	15827437	19400312	2	20111025	7
19653817	20130115	17167706	19511229	2	20130115	7
19684801	20131015	18011688	19700601	1	20131015	7
19726900	20110724	15557795	19571020	2	20110724	8
19825220	20130124	17194221	19320327	1	20130124	8
19841624	20120529	16460950	19530301	2	20120529	8
19858470	20121211	17062155	19620910	1	20121211	8
19937963	20120928	16829753	19640315	2	20120928	10
19992995	20110606	15415168	19530125	2	20110606	8
20271129	20130110	17152953	19430401	2	20130110	8
21655958	20130410	17420941	19601105	1	20130410	9
22019938	20121105	16941554	19210707	1	20121105	7
22319988	20120415	16333428	19320827	1	20120415	7
22371615	20130131	17212441	19571105	1	20130131	7
22575980	20110807	15600745	19580429	2	20110807	7
22796629	20110604	15414175	19481015	2	20110604	7
22864706	20130707	17693628	19261028	1	20130707	7
22932645	20120515	16425418	19630120	2	20120515	7
22989340	20131009	17994799	19471110	1	20131009	7
23005529	20120229	16188624	19321211	1	20120229	8
23084653	20120611	16504416	19680118	2	20120611	8
23133995	20130108	17145196	19481120	1	20130108	7
23397606	20121021	16899052	19470227	2	20121021	7
23601265	20120113	16066330	19580623	1	20120113	8
24203938	20110917	15718805	19370224	2	20110917	8

24305224	20110613	15439244	19381013	2	20110613	7
24424848	20130416	17440313	19260302	1	20130416	9
24562547	20111107	15865692	19680205	2	20111107	8
24769786	20130922	17933948	19640801	1	20130922	8
25154078	20120202	16112615	19290903	2	20120202	10
25156869	20120128	16094205	19530724	2	20120128	8
25445583	20111125	15918967	19620116	1	20111125	8
25489718	20130114	17164072	19370805	2	20130114	8
25495801	20120924	16815785	19501214	1	20120924	7
25704807	20120802	16657264	19341205	2	20120802	10
26063490	20120917	16797149	19510122	2	20120917	8
26068928	20120512	16417212	19920803	1	20120512	8
26638448	20120219	16164615	19451220	1	20120219	8
26642784	20120917	16798465	19260808	1	20120917	7
26697983	20120703	16568148	19550719	2	20120703	7
27322458	20130731	17766486	19491001	1	20130731	8
27336910	20120924	16817784	19450808	2	20120924	7
27411387	20131101	18062537	19460604	2	20131101	7
27626182	20120709	16588838	19641225	1	20120709	7
27678724	20120404	16299684	19590410	2	20120404	7
27871712	20121012	16874919	19420716	1	20121012	10
28042559	20131221	18227892	19500718	1	20131221	8
28086013	20130607	17603085	19350419	2	20130607	8
28110089	20130415	17435463	19650328	1	20130415	8
28170345	20120125	16090093	19460520	1	20120125	10
28375522	20120419	16346283	19561216	1	20120419	10
28530736	20120724	16633162	19791027	1	20120724	8
28699709	20121001	16837156	19550920	1	20121001	7
28749317	20120210	16140739	19570117	1	20120210	10
28938570	20111010	15782795	19480301	1	20111010	10
29032700	20130609	17605755	19390828	1	20130609	7
29120841	20120301	16195759	19400901	1	20120301	8
29385646	20110821	15641304	19450215	2	20110821	7
29619429	20130621	17641900	19421111	2	20130621	8
29776214	20130516	17535197	19300101	2	20130516	8
29795571	20131024	18040849	19530330	2	20131024	7
29951813	20110618	15455462	19630923	2	20110618	10
30104166	20120123	16088884	19490217	2	20120123	8
30160384	20110820	15640795	19701017	1	20110820	8
30267095	20120623	16537580	19541120	1	20120623	9
30300686	20120117	16076060	19560810	2	20120117	10
30388295	20120320	16256204	19540702	1	20120320	10
30433591	20130125	17197417	19470424	1	20130125	8
30619900	20120204	16119194	19590110	1	20120204	8
30696630	20110424	15292493	19541215	2	20110424	7
30844805	20130408	17412302	19590605	2	20130408	8
30946964	20120313	16237697	19631218	1	20120313	8
31055159	20130218	17251306	19441027	1	20130218	8
31079308	20121027	16916735	19550222	2	20121027	7
31091119	20130109	17148201	19360207	1	20130109	8
31152622	20120617	16520659	19511218	1	20120617	7
31262676	20131031	18058259	19461020	1	20131031	8
31277108	20111212	15970722	19790310	2	20111212	10
31355267	20130430	17478243	19250228	1	20130430	9
31488912	20130317	17345772	19640820	2	20130317	8

31567187	20130901	17865296	19681015	1	20130901	8
31672443	20130302	17296091	19490110	1	20130302	8
31698836	20120806	16668606	19640213	2	20120806	8
31750046	20110413	15263445	19660908	2	20110413	8
31972915	20110801	15580881	19621112	2	20110801	10
31998046	20130607	17600133	19610203	2	20130607	7
32044556	20120708	16584231	19450527	1	20120708	7
32328544	20130925	17945488	19711012	2	20130925	9
32332335	20120906	16766119	19380128	1	20120906	8
32776344	20130117	17175002	19650101	1	20130117	8
32797481	20120516	16428829	19510302	1	20120516	7
32811180	20130717	17727467	19640818	2	20130717	8
33038083	20130423	17459868	19410616	1	20130423	8
33058525	20130110	17153732	19360120	1	20130110	7
33118331	20110422	15290699	19420927	2	20110422	7
33330324	20120612	16508039	19681226	1	20120612	7
33511658	20130413	17431270	20050417	2	20130413	9
33514362	20120202	16112655	19650430	1	20120202	8
33853939	20130603	17584345	19491006	1	20130603	8
33975358	20130426	17469972	19770214	2	20130426	7
33982079	20121103	16937749	19731210	2	20121103	8
34024323	20130125	17198111	19551002	1	20130125	8
34062958	20130110	17153165	19390815	1	20130110	8
34116444	20130729	17760998	19430505	2	20130729	7
34218752	20130831	17864588	19350310	1	20130831	7
34260327	20131118	18120642	19570216	1	20131118	8
34587661	20120930	16831758	19670715	1	20120930	8
34741690	20121210	17057980	19570202	2	20121210	7
34794951	20111126	15921294	19620629	1	20111126	10
35213560	20110625	15473228	19470825	1	20110625	9
35214621	20120326	16271498	19530920	1	20120326	7
35220703	20120323	16264767	19310411	1	20120323	7
35238143	20120117	16077064	19561223	1	20120117	10
35409735	20120327	16274936	19661108	1	20120327	7
35455108	20110514	15355993	19481120	2	20110514	8
35488938	20121213	17071060	19651017	2	20121213	9
35760691	20120305	16209267	19760326	2	20120305	8
35767498	20120214	16151847	19670615	1	20120214	9
35797978	20130124	17195090	19620125	2	20130124	8
35824556	20120303	16203507	19540415	1	20120303	8
35853077	20111113	15883471	19511115	2	20111113	9
35994184	20120112	16060275	19570607	1	20120112	7
36001157	20131023	18035502	19791019	2	20131023	9
36028816	20120709	16587968	19500906	1	20120709	8
36032152	20130227	17285651	19460906	1	20130227	8
36170157	20110426	15299256	19550126	1	20110426	7
36176519	20120128	16094005	19510430	2	20120128	8
36197327	20131226	18240485	19600205	2	20131226	7
36295053	20131021	18029742	19390223	2	20131021	8
36325947	20110601	15398085	19840305	1	20110601	7
36335929	20110804	15595046	19650817	1	20110804	7
36352984	20130609	17605568	19341109	1	20130609	7
36356464	20130515	17531861	19541214	1	20130515	7
36371376	20120709	16588856	19510120	1	20120709	8
36374240	20120508	16403280	19660119	1	20120508	10

36382124	20130828	17857394	19520210	1	20130828	7
36414387	20121220	17092433	19731128	2	20121220	7
36454725	20110801	15580596	19621023	1	20110801	8
36534711	20130716	17724542	19300917	1	20130716	7
36563301	20120201	16104483	19481129	1	20120201	10
36603379	20120622	16534967	19620501	2	20120622	7
36625828	20110422	15290341	19440810	1	20110422	7
36726860	20110615	15447094	19520824	2	20110615	8
36733105	20120306	16214143	19470205	1	20120306	7
36752837	20120411	16322790	19641110	2	20120411	9
36793665	20111013	15795334	19351206	2	20111013	7
36796335	20120504	16392503	19610701	2	20120504	7
36817886	20111007	15780039	19520320	2	20111007	7
36840423	20110705	15503360	20060412	1	20110705	10
36876547	20110606	15415126	19500314	1	20110606	9
36896534	20130908	17893860	19291115	1	20130908	7
36920237	20110706	15508073	19410318	2	20110706	8
37021957	20131119	18125507	19660802	2	20131119	8
37089615	20130926	17947595	19420310	1	20130926	7
37127698	20120511	16415375	19580112	1	20120511	8
37197710	20110720	15549704	19600602	2	20110720	7
37220885	20110928	15748076	19630615	1	20110928	8
37250876	20110417	15272956	19570309	1	20110417	7
37256487	20130424	17462246	19461020	1	20130424	8
37260029	20120330	16283007	19861001	2	20120330	7
37314457	20110622	15465919	19551020	1	20110622	7
37328282	20120410	16319873	19910901	1	20120410	8
37355207	20111113	15883377	19660311	2	20111113	7
37359049	20120127	16092013	19660314	1	20120127	10
37366895	20120318	16250512	19631021	2	20120318	7
37371612	20120730	16647297	19680626	2	20120730	7
37373458	20131020	18025625	19780212	2	20131020	7
37373561	20120926	16824038	20060331	1	20120926	9
37393730	20130125	17197879	19610216	1	20130125	7
37436127	20110401	15226687	19640221	2	20110401	10
37459908	20110607	15419879	19650608	1	20110607	10
37486070	20110825	15654469	19460202	1	20110825	8
37489284	20130507	17504331	19600226	2	20130507	9
37500020	20121104	16938641	19581117	2	20121104	10
37502899	20111202	15940789	19631201	2	20111202	10
37527529	20130121	17184553	19780626	1	20130121	9
37550906	20111205	15947366	19710228	1	20111205	7
37556379	20111219	15990097	19680215	1	20111219	8
37567876	20110529	15392149	19960817	1	20110529	10
37574575	20110926	15741510	19470121	2	20110926	8
37623522	20110923	15736061	19670424	2	20110923	8
37668034	20110419	15280691	19591202	1	20110419	7
37694614	20120406	16308502	19571212	2	20120406	10
37743423	20120113	16067397	19700202	1	20120113	10
37749476	20130415	17436052	19520110	1	20130415	7
37750086	20110724	15558120	19481120	1	20110724	8
37767514	20110605	15414488	19971003	1	20110605	10
37780726	20111019	15811958	19571230	1	20111019	10
37800398	20120605	16484790	19570222	1	20120605	9
37800741	20131003	17973857	19500227	2	20131003	8

37825197	20111223	16003729	19411214	2	20111223	9
37832829	20120723	16629670	19390418	2	20120723	10
37835099	20111023	15820507	19590319	1	20111023	10
37865251	20110817	15633074	20091203	1	20110817	8
3787274520130101		17117525	19430606	1	20130101	7
3787407020130305		17307864	19591228	1	20130305	7
37900633	20120716	16610714	19420317	2	20120716	7
37912713	20110524	15381749	19740717	1	20110524	10
37922853	20120204	16119249	19570722	1	20120204	8
37923732	20110610	15428919	19650318	1	20110610	8
37925590	20110519	15369741	19670801	2	20110519	8
37927121	20120228	16187565	19500221	2	20120228	7
37929707	20120116	16073758	19440401	1	20120116	8
3794180320131219		18223165	19730119	2	20131219	9
37943536	20110623	15469254	19630212	1	20110623	10
37970733	20121014	16878345	19631124	1	20121014	9
37993503	20120530	16463665	19580505	2	20120530	8
38012270	20110511	15347989	19770623	1	20110511	9
38028352	20120902	16746780	19560110	1	20120902	10
38034581	20110417	15272874	19900329	2	20110417	7
38046730	20110801	15577276	19920219	1	20110801	10
38054385	20120627	16548872	19690217	1	20120627	8
38062316	20110622	15466044	19291015	1	20110622	10
38082392	20111019	15811787	19590207	2	20111019	7
38113063	20120321	16260712	20001105	1	20120321	7
38124980	20110519	15369726	20010707	1	20110519	9
38125132	20110609	15428796	19510222	1	20110609	10
38129496	20110808	15605084	19710419	2	20110808	8
38130062	20120808	16679672	19511025	1	20120808	7
38131827	20110908	15696337	20090818	1	20110908	10
38161409	20110910	15701727	19960430	2	20110910	7
38189627	20110531	15397239	19360604	2	20110531	10
38210843	20110808	15603903	19580914	1	20110808	10
38215155	20120119	16084234	19700531	2	20120119	8
38218881	20120105	16040269	19940317	1	20120105	10
38238629	20111112	15882883	19541113	1	20111112	7
38260536	20111115	15891175	19561118	1	20111115	9
3827087020130506		17499683	19701008	2	20130506	7
38291757	20110921	15730359	19531219	1	20110921	10
38311852	20120604	16479602	19550827	1	20120604	8
38320897	20120714	16606252	19420214	1	20120714	7
38325303	20120307	16219265	19590502	2	20120307	10
38347987	20111116	15892698	19480710	2	20111116	10
38366926	20120306	16214085	19310614	1	20120306	8
38369481	20120324	16268102	19630722	1	20120324	10
38386593	20111123	15913876	19540816	1	20111123	7
38402152	20111005	15771516	19591030	1	20111005	7
3841072920130525		17559024	19490922	2	20130525	7
38425922	20111121	15905572	20040112	1	20111121	10
38463820	20110928	15747969	19971201	1	20110928	8
38477202	20120102	16027417	19390109	1	20120102	8
38496116	20111120	15903645	19621108	2	20111120	8
38507743	20120426	16365037	19810515	2	20120426	10
3856469120130408		17409518	19750203	1	20130408	9
38586559	20120513	16417626	19541105	2	20120513	10

38596097	20111204	15943313	19671026	2	20111204	7
38598640	20120221	16171896	19890801	2	20120221	7
3862592220130308		17321734	19850413	1	20130308	8
38626710	20120912	16785359	19580508	1	20120912	7
38631300	20111015	15800494	19531110	1	20111015	8
38642772	20120705	16577986	19740905	2	20120705	9
38650203	20120424	16358843	19601010	1	20120424	8
3865160420130713		17715456	19650611	1	20130713	7
38656870	20120521	16440554	19660808	2	20120521	10
38660785	20120909	16773135	19660601	1	20120909	8
38660912	20120730	16648632	19800718	2	20120730	7
38663604	20120508	16403578	19440225	1	20120508	8
38666283	20120316	16248425	20000129	2	20120316	7
3867678720130702		17671831	19800921	2	20130702	9
38703934	20120311	16229710	19370105	1	20120311	7
38707469	20120411	16322938	19481012	1	20120411	10
38751085	20120725	16636045	19540701	1	20120725	10
3875785620130420		17452929	19320121	2	20130420	7
3876071320130907		17893481	19640529	2	20130907	7
38765423	20120321	16260880	19540826	2	20120321	7
3878280820130112		17159330	19730313	1	20130112	8
38785556	20120209	16137234	19590302	2	20120209	10
38833453	20121018	16893357	19590614	1	20121018	8
38862394	20121011	16871922	19720911	1	20121011	10
38877735	20120729	16645594	19741128	1	20120729	10
3891581020121217		17081385	19551226	1	20121217	7
38940635	20120416	16337012	19950803	1	20120416	10
38967805	20120819	16711619	19480329	2	20120819	8
3897229120130305		17307932	19520420	1	20130305	9
38984973	20121004	16850792	19340204	1	20121004	7
3901640520130515		17531411	19900523	1	20130515	7
39060010	20120909	16773034	19490910	2	20120909	8
39073546	20120729	16645356	19610120	1	20120729	7
39075575	20121209	17052978	19360729	2	20121209	8
39111085	20120801	16656651	19470228	1	20120801	7
39131890	20120817	16709637	19460824	1	20120817	7
39132235	20121026	16915265	19590718	1	20121026	10
39135041	20120807	16674222	19440718	1	20120807	10
39178693	20120723	16630243	19550415	1	20120723	8
39179209	20120719	16621433	20030416	2	20120719	10
39183067	20121107	16952911	19681016	1	20121107	10
39201451	20120713	16603839	19740109	2	20120713	7
39206230	20120916	16794306	19590626	1	20120916	10
39214089	20120824	16727315	19610503	1	20120824	8
39216803	20120804	16664498	19520226	1	20120804	8
39251064	20120821	16717084	19790518	1	20120821	9
39279635	20121104	16938836	19680517	1	20121104	8
3928716620121227		17110526	19490613	2	20121227	7
39297579	20121118	16986105	19591010	1	20121118	8
3930860620121218		17085570	19660825	1	20121218	8
3935040820131013		18003039	19641206	1	20131013	7
3936701620121224		17099591	19561021	2	20121224	7
3936750520130226		17281247	19510513	2	20130226	8
3938399820121219		17088084	19610521	2	20121219	7
3940089020130906		17890061	19551221	1	20130906	7

3948039220130808	17800522	19830831	2	20130808	8
3948982220130331	17383845	19600213	1	20130331	9
3953068220130714	17716000	19591014	1	20130714	8
3957041720130405	17404944	19551202	1	20130405	9
3959782520130714	17716077	19910529	2	20130714	7
3962733320130107	17140095	19660420	1	20130107	9
3962916820130310	17324054	19610331	2	20130310	9
3963572820131212	18201875	19600721	1	20131212	9
3964503920130619	17638195	19611119	2	20130619	7
3964708020130725	17751948	19320323	2	20130725	7
3964771720130310	17324154	19390518	1	20130310	8
3969381720130811	17806694	19370216	2	20130811	8
3969857220130622	17646660	19530218	2	20130622	8
3972432820130624	17650286	19651105	2	20130624	9
3972777020131008	17991376	19560502	1	20131008	7
3973707020130409	17415528	19951129	2	20130409	8
3974678620130613	17619310	19490107	2	20130613	8
3976883720130327	17376289	19710919	2	20130327	7
3989459220131009	17996757	19660917	1	20131009	7
3993171020130906	17891470	19710906	1	20130906	8
3999783620130629	17665099	19621020	1	20130629	7
4001096620130902	17871154	19730328	1	20130902	7
4004653520131110	18094898	19700425	2	20131110	8
4008045320130831	17863367	19820503	1	20130831	8
4019234520131026	18045263	20101005	1	20131026	9
4023268620131108	18092606	19940519	2	20131108	8
4027216020131022	18031363	19610224	1	20131022	7
4030903320130923	17936607	19630312	2	20130923	8
4042666220131104	18070535	19540808	1	20131104	7
122634 20120305	16209035	19280506	1	20120305	8
607396 20120311	16229443	19641114	2	20120311	8
1195682 20111207	15956473	19670603	1	20111207	8
214404920130929	17954851	19700612	1	20130929	8
222102520130217	17250379	19390306	2	20130217	7
2307837 20120606	16487426	19480612	2	20120606	8
326868620130319	17351964	19450102	2	20130319	7
3696911 20120407	16310352	19370220	1	20120407	7
4592567 20120415	16333584	19301217	1	20120415	8
4596707 20121105	16941867	19310608	1	20121105	9
505535620130612	17615031	19320921	1	20130612	8
5212373 20121031	16925773	19321110	2	20121031	8
579585720130723	17744608	19371112	1	20130723	8
5996474 20120729	16645413	19420215	2	20120729	7
7060846 20120710	16592825	19541215	2	20120710	10
8003063 20111116	15893316	19500401	1	20111116	9
837120820131117	18118324	19410701	2	20131117	7
9073147 20121011	16872203	19500514	1	20121011	7
9615638 20110806	15600192	19250203	1	20110806	10
9961584 20120905	16762314	19560308	1	20120905	7
10074869 20110417	15272773	19330918	1	20110417	9
11032545 20120705	16577590	19351207	2	20120705	7
11101465 20120624	16538126	19510916	1	20120624	8
11298729 20111114	15887283	19570702	2	20111114	10
11515483 20120119	16084214	19510304	2	20120119	7
1231224220131126	18141792	19630112	1	20131126	8

12441348	20130824	17845464	19680417	1	20130824	7
12621533	20111029	15837478	19690228	2	20111029	7
12756013	20110915	15713459	19691125	2	20110915	7
12906615	20120821	16719255	19440628	2	20120821	7
13342944	20111208	15960835	19330909	2	20111208	10
13372968	20120508	16403064	19540730	2	20120508	7
14125156	20130624	17650770	19441117	2	20130624	7
14183950	20131022	18033304	19550910	2	20131022	7
14959321	20120211	16142553	19311020	1	20120211	8
15138702	20110819	15639189	19260604	1	20110819	8
15501765	20111202	15940746	19640925	2	20111202	10
15727750	20130824	17846622	19470802	2	20130824	7
15798353	20131121	18133122	19540610	1	20131121	7
16000514	20121231	17117260	19600528	1	20121231	7
16258007	20110921	15729758	19341216	1	20110921	10
17049682	20120603	16474737	19740813	1	20120603	9
17366824	20121116	16982983	19350301	1	20121116	7
17539865	20110501	15309377	19350527	1	20110501	8
17990782	20130408	17411644	19321125	2	20130408	7
18336419	20130820	17837059	19561115	1	20130820	7
18462867	20130425	17467020	19570907	1	20130425	7
18585609	20120301	16193557	19561210	1	20120301	10
18608043	20130616	17625671	19651118	2	20130616	8
18749956	20110606	15415021	19341220	1	20110606	8
18799934	20130719	17734658	19560120	2	20130719	8
18858338	20130204	17222534	19591004	2	20130204	7
19228332	20130331	17383978	19510806	1	20130331	8
19300573	20130417	17444337	19580702	1	20130417	9
19588933	20130926	17949502	19600511	1	20130926	8
19602790	20120101	16022780	19880622	1	20120101	10
19684801	20131108	18092623	19700601	1	20131108	7
19961081	20111223	16003170	19520117	2	20111223	8
20121044	20130507	17505394	19281222	1	20130507	9
20216084	20111214	15977674	19560809	2	20111214	8
20580238	20131020	18026021	19591010	1	20131020	8
20677865	20130125	17197476	19571008	1	20130125	7
20733475	20110820	15640805	19401020	1	20110820	7
20828879	20130301	17293032	19391205	1	20130301	8
21097063	20120728	16644818	19340514	1	20120728	7
21207341	20111031	15840538	19380806	1	20111031	9
21912572	20120913	16788908	19520128	1	20120913	8
22575980	20110824	15650379	19580429	2	20110824	10
22670673	20130624	17650834	19481202	1	20130624	7
22932645	20120809	16684474	19630120	2	20120809	10
23133995	20130417	17444169	19481120	1	20130417	9
23598745	20130730	17763081	19670316	1	20130730	8
23955319	20121031	16925939	19840427	1	20121031	10
24102043	20110518	15367391	19471011	1	20110518	8
24139524	20110517	15363864	19690811	2	20110517	9
24146836	20120713	16601213	19401014	2	20120713	10
24203938	20111002	15756470	19370224	2	20111002	7
24305224	20110714	15534171	19381013	2	20110714	8
24424848	20130510	17517129	19260302	1	20130510	8
24459776	20120516	16428068	19740226	1	20120516	10
24481729	20110514	15355840	19380724	1	20110514	8

24512854	20130805	17783945	19620611	2	20130805	7
24612188	20130311	17327395	19331103	1	20130311	7
24625272	20130127	17200111	19251013	2	20130127	7
25156869	20120411	16323716	19530724	2	20120411	8
25257527	20130519	17541099	19570130	2	20130519	9
25445583	20111220	15993907	19620116	1	20111220	7
25704807	20120907	16769352	19341205	2	20120907	7
25727406	20110703	15493726	19601225	1	20110703	10
26062748	20120211	16142966	19680517	1	20120211	7
26130643	20121001	16835509	19420630	2	20121001	9
26187166	20110915	15714019	19381003	1	20110915	9
26628206	20120831	16744013	19360107	1	20120831	8
27219096	20120804	16664375	19500225	1	20120804	10
27322458	20130918	17928978	19491001	1	20130918	8
27411387	20131110	18094787	19460604	2	20131110	8
27487634	20110806	15600380	19311019	2	20110806	8
28053841	20131230	18248996	19510220	2	20131230	8
28110089	20130502	17489182	19650328	1	20130502	8
28989788	20110526	15387272	19800702	1	20110526	7
29385646	20130510	17515152	19450215	2	20130510	8
29526690	20120119	16084015	19610922	2	20120119	9
29619429	20130710	17704850	19421111	2	20130710	7
29670460	20130727	17756828	19630525	2	20130727	7
29673389	20111010	15782813	19600106	2	20111010	10
29951813	20110630	15485843	19630923	2	20110630	10
30002007	20120620	16529128	19670116	2	20120620	8
30028081	20120514	16419708	19470806	2	20120514	10
30104166	20120315	16245121	19490217	2	20120315	8
30160384	20110827	15658543	19701017	1	20110827	7
30267095	20120707	16583968	19541120	1	20120707	10
30388295	20120401	16285270	19540702	1	20120401	9
30903005	20130623	17647089	19521124	2	20130623	7
30926659	20111124	15916363	19620808	2	20111124	9
31091119	20130131	17211159	19360207	1	20130131	7
31388246	20131225	18238035	19701018	1	20131225	7
32296836	20110801	15580075	19651129	1	20110801	10
32630641	20111125	15919225	19660120	1	20111125	8
32811180	20131005	17981675	19640818	2	20131005	8
33109001	20111009	15782298	19460228	2	20111009	10
33118331	20110426	15299268	19420927	2	20110426	8
33371914	20120705	16576107	19510922	1	20120705	7
33473686	20121129	17017487	19380108	2	20121129	7
33767532	20121102	16935124	19571022	1	20121102	8
33982079	20130511	17518765	19731210	2	20130511	8
34010565	20121223	17097474	19561002	1	20121223	7
34047875	20120204	16119234	19531118	1	20120204	7
34290841	20120906	16766250	19530707	2	20120906	7
34524157	20130408	17410472	19600417	1	20130408	8
34879475	20130714	17716150	19660925	2	20130714	7
34961427	20120914	16792456	19600215	1	20120914	8
34998953	20130430	17477821	19670830	2	20130430	9
35170491	20110717	15539258	20050706	1	20110717	9
35238143	20120304	16204227	19561223	1	20120304	8
35325292	20120212	16143127	19170606	1	20120212	8
35488938	20130513	17523296	19651017	2	20130513	8

35517158	20110813	15621776	19901226	2	20110813	8
35539754	20110918	15719111	19660512	2	20110918	9
35589163	20110808	15601045	19981212	2	20110808	7
35609202	20120508	16404060	19340112	2	20120508	10
35687162	20120206	16122801	19700809	2	20120206	9
35692070	20120830	16741862	19400818	2	20120830	9
35797967	20120430	16373016	19540208	2	20120430	8
35993545	20120703	16568353	19611202	2	20120703	7
35994184	20120208	16133310	19570607	1	20120208	8
3600115720131204		18170822	19791019	2	20131204	9
36028816	20120917	16796181	19500906	1	20120917	7
36181494	20111007	15779181	19300408	1	20111007	8
36333150	20120116	16073648	19530612	1	20120116	9
3638212420131001		17962606	19520210	1	20131001	8
36430203	20111023	15820386	19611004	1	20111023	8
36563301	20120317	16250107	19481129	1	20120317	9
36609753	20110830	15664122	19370811	2	20110830	8
36615200	20121127	17013439	19770509	2	20121127	9
3666995120130828		17855798	19580214	1	20130828	8
36726860	20110728	15569709	19520824	2	20110728	9
36757456	20120227	16185223	19560310	2	20120227	10
36795398	20120826	16729651	19640327	1	20120826	8
36817886	20111028	15836044	19520320	2	20111028	7
36876547	20110716	15538657	19500314	1	20110716	10
37013095	20110922	15733125	19970605	1	20110922	7
37013788	20110519	15370280	19661010	2	20110519	9
37036752	20120403	16294221	19570524	2	20120403	9
37155207	20110424	15292540	19580413	2	20110424	7
37225062	20120227	16185604	19580610	1	20120227	8
37272096	20110923	15736486	19591113	1	20110923	7
37324917	20111117	15898020	19510613	2	20111117	7
37339256	20120718	16618251	19871128	1	20120718	8
3737345820131128		18149414	19780212	2	20131128	8
37378180	20110620	15459702	19430307	1	20110620	7
37436127	20110412	15258562	19640221	2	20110412	10
37499233	20121209	17053164	19520816	2	20121209	8
37500291	20120212	16143190	19650420	1	20120212	10
37501318	20121028	16917389	19460612	1	20121028	10
37565927	20120426	16364668	19370308	1	20120426	7
3760402720130311		17327478	19760828	2	20130311	9
37667520	20120504	16392778	19420922	1	20120504	9
37708080	20110717	15539161	19830430	2	20110717	8
37734773	20120407	16310347	19900806	1	20120407	9
3774947620130516		17533189	19520110	1	20130516	7
37793241	20121110	16962608	19680801	2	20121110	8
3780038720130127		17200170	19630206	1	20130127	8
37804152	20111031	15839971	19590401	1	20111031	7
37829713	20120313	16237819	19750101	1	20120313	8
37835099	20111207	15955296	19590319	1	20111207	10
37865251	20111026	15830521	20091203	1	20111026	8
3787407020130420		17452949	19591228	1	20130420	7
37926684	20120405	16299778	19590520	1	20120405	8
37950633	20120713	16604509	19460525	2	20120713	7
38008489	20111118	15900624	19681205	1	20111118	9
38012270	20110604	15414088	19770623	1	20110604	10

38018610	20110527	15390422	19850504	1	20110527	8
38046730	20111018	15808512	19920219	1	20111018	10
38054670	20110808	15604373	19360415	1	20110808	7
38066761	20110904	15678959	19490901	1	20110904	7
38118842	20111118	15901474	19550919	2	20111118	9
38121378	20111026	15830103	19590501	1	20111026	9
38134677	20120717	16614754	19570317	1	20120717	7
38137085	20120224	16180880	19560420	1	20120224	10
38148628	20111111	15880509	19510515	1	20111111	7
3817166320130520		17544347	19561201	1	20130520	8
38210843	20110826	15656882	19580914	1	20110826	7
38220405	20120519	16436608	19560521	1	20120519	8
38224338	20120918	16802277	19580410	2	20120918	7
38238072	20110910	15701464	19820408	1	20110910	8
38238629	20120102	16027627	19541113	1	20120102	10
38252630	20111011	15786659	19680502	1	20111011	7
3825602920130512		17519192	19570115	1	20130512	8
38258070	20120117	16076478	19620721	2	20120117	10
38288685	20111206	15948647	19320503	1	20111206	8
38325303	20120509	16408182	19590502	2	20120509	7
38363927	20120508	16402264	19530220	1	20120508	9
38375438	20120114	16069251	19611201	1	20120114	9
3839917620130717		17727252	19550803	1	20130717	8
38416272	20111124	15916822	19760715	1	20111124	10
38425922	20111212	15969040	20040112	1	20111212	10
38428772	20120413	16331541	19460927	1	20120413	10
3845666520130130		17207923	19520913	2	20130130	9
38473722	20121002	16842216	19380306	1	20121002	7
38487239	20121017	16890125	19641208	1	20121017	8
38496116	20111228	16015280	19621108	2	20111228	8
38520273	20111226	16009127	19690101	1	20111226	7
38522280	20120614	16515456	19680616	2	20120614	7
38564237	20120420	16349462	19750727	1	20120420	10
38568819	20111024	15822005	19970324	2	20111024	8
38574855	20120129	16094712	19670625	2	20120129	7
38625046	20120925	16821442	19350312	1	20120925	10
38625115	20111108	15870740	19710421	1	20111108	10
38626323	20120714	16606409	19640120	1	20120714	7
38626710	20121018	16893129	19580508	1	20121018	10
38628987	20111113	15883429	19710412	1	20111113	8
3863717120130801		17771175	19351230	1	20130801	8
38637466	20111207	15957188	19690216	2	20111207	10
38642772	20120720	16624909	19740905	2	20120720	8
38650203	20120510	16409870	19601010	1	20120510	8
38660785	20121007	16858418	19660601	1	20121007	7
38660821	20121121	16997941	19600901	1	20121121	8
38660912	20120910	16777254	19800718	2	20120910	8
3867678720130801		17773251	19800921	2	20130801	8
38681253	20120409	16315053	19710109	2	20120409	8
38687580	20120621	16532907	19450220	2	20120621	8
38688787	20120731	16651535	19650115	1	20120731	8
3869001620130216		17250088	19530910	2	20130216	9
38703934	20120323	16265879	19370105	1	20120323	8
38707469	20120504	16391410	19481012	1	20120504	9
38711352	20120415	16333681	19590924	2	20120415	7

38721061	20120204	16119312	19880707	1	20120204	10
38725405	20111212	15968741	19550905	2	20111212	7
38760713	20131003	17974619	19640529	2	20131003	7
38763483	20120329	16281222	19940708	2	20120329	7
38765423	20120418	16343980	19540826	2	20120418	10
38788577	20120607	16493896	19600728	1	20120607	10
38790179	20120530	16463653	19440708	1	20120530	7
38814685	20120131	16100075	19930710	2	20120131	7
38818790	20130831	17864650	19661115	2	20130831	8
38877735	20120813	16695031	19741128	1	20120813	7
38879811	20130212	17243654	19340205	2	20130212	8
38891202	20120531	16465869	19591010	1	20120531	7
38892498	20120620	16530691	19511202	1	20120620	7
38913336	20120603	16474595	19570210	1	20120603	8
38914839	20130704	17687091	19480812	1	20130704	8
38953898	20120517	16431730	19640824	2	20120517	9
38972291	20130411	17421806	19520420	1	20130411	8
38979792	20120709	16588704	19560207	1	20120709	10
39018536	20130814	17816516	19401114	1	20130814	9
39027946	20120904	16757483	19650529	1	20120904	7
39049177	20121106	16947449	19640212	1	20121106	8
39062618	20130613	17619514	19390928	2	20130613	7
39097411	20120920	16809094	20100922	2	20120920	8
39111085	20120902	16747094	19470228	1	20120902	8
39131890	20121017	16887928	19460824	1	20121017	8
39155083	20130725	17752750	19801223	1	20130725	9
39163821	20130408	17412423	19350525	1	20130408	7
39168939	20121109	16960937	19580316	1	20121109	8
39238205	20130201	17217307	19520320	2	20130201	7
39278256	20130712	17714748	19570701	1	20130712	8
39287166	20130107	17140303	19490613	2	20130107	8
39306531	20130122	17187355	19390702	1	20130122	7
39308606	20130122	17187665	19660825	1	20130122	8
39326108	20130811	17806896	19630512	2	20130811	8
39340222	20130105	17134785	19461015	2	20130105	7
39359289	20121130	17022154	19400716	1	20121130	9
39362577	20130926	17945824	19590902	1	20130926	7
39363401	20121215	17077019	19930225	2	20121215	7
39435568	20131125	18140410	19431215	1	20131125	7
39455271	20130927	17952586	19500702	1	20130927	9
39476318	20130329	17382029	19510208	1	20130329	9
39511814	20130302	17296460	19661207	2	20130302	7
39518871	20130330	17383392	19530520	1	20130330	7
39531732	20130314	17340154	19430801	1	20130314	8
39541305	20130318	17349615	19970728	1	20130318	8
39561676	20130220	17264458	19730205	2	20130220	7
39570417	20130501	17481469	19551202	1	20130501	8
39576620	20130127	17199953	19910305	1	20130127	7
39597825	20130814	17819025	19910529	2	20130814	8
39606150	20131212	18203213	19481215	1	20131212	7
39632605	20130911	17906417	19720509	1	20130911	7
39693168	20130908	17893943	20001031	1	20130908	7
39698174	20130723	17745635	19731010	1	20130723	7
39699337	20130602	17578571	19621231	1	20130602	9
39724328	20130716	17723374	19651105	2	20130716	7

3972719020130706		17693108	19351119	2	20130706	7
3973291720130528		17567305	19800203	1	20130528	8
3974678620130627		17660891	19490107	2	20130627	8
3979638820131109		18094644	19511228	1	20131109	8
3982062720130702		17677226	19761208	1	20130702	7
3989287020130525		17559842	19480404	1	20130525	7
3995396320130717		17728681	19620719	2	20130717	9
3999820420130927		17951466	20060817	1	20130927	7
4002835120130715		17720499	19710809	1	20130715	9
4004653520131202		18161793	19700425	2	20131202	9
4007482620130925		17945134	19520910	1	20130925	7
4007700720130922		17934026	19650505	1	20130922	8
4009022020131004		17978584	19850329	1	20131004	8
4017850520131008		17990601	19390324	1	20131008	7
4019691620131205		18178116	19521220	1	20131205	7
4023268620131209		18190063	19940519	2	20131209	7
4045121620131201		18156746	19440920	1	20131201	8
1669405	20120614	16514843	19210920	1	20120614	8
2217449	20111227	16011289	19331121	1	20111227	7
2470475	20120316	16247990	19440608	1	20120316	7
2832759	20121207	17050501	19250310	1	20121207	9
3283087	20120323	16266813	19420905	2	20120323	10
3891698	20120408	16311053	19250115	1	20120408	8
4233425	20120325	16268831	19460508	2	20120325	8
4398576	20110727	15567907	19521001	2	20110727	7
5912072	20120521	16439074	19560705	1	20120521	10
6261445	20121207	17048225	19521009	1	20121207	10
6456855	20120903	16747912	19251215	1	20120903	10
716363520131111		18097206	19590305	2	20131111	8
7291261	20120928	16830019	19310801	1	20120928	10
8666497	20120704	16573180	19410116	1	20120704	7
8896024	20120312	16233968	19790911	1	20120312	8
9073147	20121105	16943692	19500514	1	20121105	8
911548420131113		18108732	19540430	2	20131113	9
9392169	20120905	16760416	19500601	2	20120905	7
993076120130716		17724530	19500220	1	20130716	7
11101465	20120816	16706319	19510916	1	20120816	9
11298729	20111221	15997990	19570702	2	20111221	9
1130307420121224		17100261	19420406	1	20121224	7
12110097	20120625	16541733	19510623	2	20120625	8
12756013	20111006	15774086	19691125	2	20111006	7
13183023	20111113	15883355	19290205	1	20111113	7
13642347	20110908	15696434	19480512	1	20110908	7
13667613	20121116	16983518	19400414	2	20121116	10
14296356	20120215	16155479	19610127	2	20120215	7
14823155	20111105	15862088	19630729	2	20111105	8
16021128	20111126	15921319	19490824	2	20111126	7
16084530	20121012	16875878	19600224	1	20121012	10
16095822	20120418	16344046	19501027	2	20120418	7
16746682	20111201	15933466	19611028	2	20111201	9
17518808	20120921	16812126	19621025	1	20120921	9
18155970	20120731	16651105	19490620	1	20120731	9
18251315	20120513	16417668	19320915	1	20120513	10
1846286720130502		17489244	19570907	1	20130502	8
19129192	20111203	15941178	19580618	1	20111203	8

19195201	20130815	17821977	19420228	2	20130815	8
19474772	20120527	16454903	19330720	1	20120527	10
19542406	20111224	16005343	19400312	2	20111224	8
19602790	20120613	16511379	19880622	1	20120613	7
19726900	20110827	15658064	19571020	2	20110827	7
19943716	20130715	17720117	19460224	1	20130715	7
20239649	20130426	17468132	19321228	1	20130426	9
20368074	20111221	15998050	19590322	2	20111221	8
20486297	20121030	16923253	19590227	1	20121030	8
20581311	20130428	17471940	19630819	1	20130428	7
20733475	20111015	15800341	19401020	1	20111015	8
21097063	20120812	16690223	19340514	1	20120812	7
21184985	20130409	17416988	19510223	1	20130409	7
21466433	20120814	16697524	19330608	1	20120814	7
21519437	20120213	16147440	19350219	1	20120213	7
21655958	20130614	17622743	19601105	1	20130614	8
21951688	20120820	16713759	19620126	1	20120820	9
21972430	20120429	16369767	19600229	1	20120429	10
22405910	20121227	17110667	19590922	1	20121227	7
22665243	20120401	16285643	19460829	2	20120401	8
23092866	20120405	16304210	19471110	2	20120405	10
23198836	20110618	15455288	19450104	2	20110618	8
24035516	20110901	15672221	19460617	1	20110901	7
24481729	20110522	15375392	19380724	1	20110522	10
24694802	20130802	17776565	19551213	2	20130802	8
24885801	20111214	15978108	19631125	1	20111214	10
25489718	20130416	17440215	19370805	2	20130416	8
26123682	20120828	16735191	19520823	2	20120828	7
26157426	20110911	15702102	19790509	2	20110911	7
26638584	20131010	17997273	19430506	2	20131010	7
26887229	20120813	16694290	19460502	2	20120813	7
26980574	20110912	15702285	19370615	2	20110912	10
27411387	20131115	18115414	19460604	2	20131115	9
28014779	20121224	17100715	19280608	2	20121224	7
28082317	20131101	18063369	19350702	2	20131101	8
28110089	20130624	17650723	19650328	1	20130624	7
28169815	20131205	18176153	19721006	2	20131205	7
28277534	20121120	16994297	19710519	1	20121120	8
28373742	20121224	17100989	19370730	2	20121224	7
28885974	20121115	16979898	19571124	1	20121115	7
29385646	20130605	17592093	19450215	2	20130605	8
29526690	20120305	16208510	19610922	2	20120305	9
30002007	20120710	16592678	19670116	2	20120710	10
30104166	20120409	16314650	19490217	2	20120409	9
30160384	20110908	15695613	19701017	1	20110908	10
30171756	20111212	15970288	19320612	1	20111212	9
30267095	20120809	16684536	19541120	1	20120809	10
30295217	20130429	17475168	19611120	1	20130429	8
30709081	20120827	16733139	19481102	2	20120827	10
30844429	20130411	17425529	19680927	1	20130411	8
31006061	20130222	17272070	19361026	2	20130222	8
31698836	20120913	16788804	19640213	2	20120913	9
31751118	20131003	17973074	19411012	2	20131003	7
32401528	20110722	15556135	19280405	1	20110722	10
32402101	20111213	15974348	19371022	1	20111213	8

32630641	20111222	16000987	19660120	1	20111222	10
32797481	20120701	16556758	19510302	1	20120701	8
32916419	20120210	16140049	19540607	2	20120210	8
33118331	20110520	15373745	19420927	2	20110520	8
3367639020130702		17677255	19651228	1	20130702	8
33846547	20120605	16484404	19491016	1	20120605	10
34047875	20120305	16208352	19531118	1	20120305	7
3406295820130218		17254581	19390815	1	20130218	7
3421875220131013		18003196	19350310	1	20131013	8
34246418	20120403	16295529	19520306	2	20120403	7
34278847	20110805	15598415	19600120	2	20110805	7
3435521020131107		18086043	19690725	2	20131107	8
34413315	20110801	15580498	19550102	2	20110801	7
3461590220131223		18231396	19570110	1	20131223	9
3477404420131112		18101510	19480928	2	20131112	7
3491608020130512		17518967	19560515	1	20130512	8
3499895320130528		17567200	19670830	2	20130528	8
3535607120130519		17541227	19500910	1	20130519	8
35519621	20120402	16291402	19590819	2	20120402	8
3553975420130630		17665729	19660512	2	20130630	7
35745790	20111103	15856236	19610118	2	20111103	8
35766893	20120531	16466452	19440129	2	20120531	8
35767498	20120315	16244969	19670615	1	20120315	7
35824556	20120412	16326028	19540415	1	20120412	7
35853077	20120201	16107786	19511115	2	20120201	8
35872630	20121018	16893774	19590701	1	20121018	7
35994184	20120224	16180882	19570607	1	20120224	8
36333150	20120219	16164558	19530612	1	20120219	8
36355574	20120301	16196354	19460408	1	20120301	8
36405604	20111130	15930064	19770712	2	20111130	7
3653399020130905		17883454	19500228	2	20130905	8
36545376	20121019	16897072	19550310	1	20121019	10
36689846	20120527	16454792	19421230	1	20120527	8
36776268	20111125	15918928	20000607	1	20111125	8
36805659	20121009	16865572	19600529	2	20121009	7
36846545	20120110	16056166	20000922	1	20120110	8
36927261	20111105	15862003	19670610	2	20111105	7
36984733	20121024	16909108	20010926	2	20121024	8
37013095	20111114	15886380	19970605	1	20111114	8
37016345	20120810	16688178	19621104	1	20120810	7
37225062	20120331	16285047	19580610	1	20120331	7
37265875	20111002	15756624	19440821	2	20111002	8
37287380	20110721	15553116	20001222	1	20110721	8
37355207	20120201	16107850	19660311	2	20120201	8
3737450820130826		17850897	19691012	1	20130826	7
37506302	20110927	15745013	19660715	1	20110927	8
37623362	20111027	15833552	19511125	1	20111027	7
37668034	20110510	15343881	19591202	1	20110510	7
37708080	20110820	15640786	19830430	2	20110820	7
37792066	20111103	15856224	19801028	2	20111103	10
3780038720130403		17398950	19630206	1	20130403	7
37834029	20110611	15434124	19910130	2	20110611	10
37857775	20110606	15415214	19750708	2	20110606	8
3787347520130525		17559896	19400409	1	20130525	8
37887517	20111212	15969973	19420624	1	20111212	8

37943547	20120924	16817432	19551107	1	20120924	8
37950633	20120813	16695028	19460525	2	20120813	9
37982573	20110722	15556113	19810902	2	20110722	8
37991541	20111220	15994586	19571107	1	20111220	7
38008489	20111223	16002175	19681205	1	20111223	8
38044916	20120629	16554944	19590305	2	20120629	7
38077519	20110624	15472243	19691014	2	20110624	8
38116846	20120227	16184868	19771208	1	20120227	10
38118842	20111202	15939787	19550919	2	20111202	8
38124913	20120831	16743656	19610501	2	20120831	9
38125132	20110727	15567808	19510222	1	20110727	10
38191810	20120425	16361053	19461102	1	20120425	8
38210843	20110905	15683510	19580914	1	20110905	10
38211631	20110824	15650743	19401123	2	20110824	8
38219806	20111128	15925302	19800327	1	20111128	7
38220405	20120618	16521146	19560521	1	20120618	8
38238629	20120130	16098376	19541113	1	20120130	7
38243651	20120229	16190256	19790218	1	20120229	8
38252630	20111113	15883298	19680502	1	20111113	7
38258070	20120126	16090368	19620721	2	20120126	10
38288903	20111002	15756560	19490301	2	20111002	7
38320897	20121013	16877772	19420214	1	20121013	10
38325303	20120602	16474152	19590502	2	20120602	8
38345072	20121009	16867507	19720217	1	20121009	8
38402152	20111117	15898132	19591030	1	20111117	9
38416272	20111217	15986640	19760715	1	20111217	10
38422707	20120423	16355396	19790206	1	20120423	8
3845666520130227		17284943	19520913	2	20130227	7
3845817220130220		17264486	19450217	1	20130220	7
38520091	20120426	16364053	19341111	1	20120426	8
38520273	20120105	16041347	19690101	1	20120105	7
38521356	20111026	15830587	19881209	1	20111026	8
3852451520130920		17931744	19370116	2	20130920	7
38562457	20120326	16271822	19760224	1	20120326	8
38564237	20120713	16603604	19750727	1	20120713	8
38567054	20120209	16137230	20090613	1	20120209	9
38568819	20111121	15907095	19970324	2	20111121	7
38574855	20120301	16196312	19670625	2	20120301	8
3860324620131212		18203206	19681224	1	20131212	7
38619806	20120703	16568370	19510712	1	20120703	7
38623493	20120108	16047174	19490613	1	20120108	8
3862504620121216		17077518	19350312	1	20121216	7
3862592220131022		18032784	19850413	1	20131022	7
38626323	20120823	16724777	19640120	1	20120823	9
3863717120131227		18243389	19351230	1	20131227	9
38642772	20120807	16675405	19740905	2	20120807	7
38654012	20120705	16577834	19500830	2	20120705	10
38656052	20120713	16604418	19401120	1	20120713	7
38660912	20121015	16882410	19800718	2	20121015	7
38663604	20120717	16614763	19440225	1	20120717	10
3869001620130225		17276557	19530910	2	20130225	8
3870092420130422		17456972	19951104	1	20130422	8
38703934	20120502	16384106	19370105	1	20120502	7
38721061	20120211	16142963	19880707	1	20120211	8
38763483	20120531	16466544	19940708	2	20120531	10

38780175	20120321	16260398	19481122	2	20120321	7
38782808	20130329	17382063	19730313	1	20130329	8
38783210	20120213	16147016	19590110	1	20120213	10
38804056	20130529	17570247	19650203	1	20130529	9
38815780	20120624	16538033	19460302	1	20120624	7
38819771	20130801	17772592	20001222	1	20130801	7
38850123	20120519	16436415	19620517	1	20120519	9
38875911	20120427	16367695	19690615	2	20120427	7
38877735	20120829	16739115	19741128	1	20120829	8
38880385	20120729	16645574	19470419	1	20120729	8
38892852	20120618	16524643	19620211	1	20120618	9
38908100	20120619	16528344	19801122	2	20120619	8
38914839	20130807	17795993	19480812	1	20130807	7
38933992	20121027	16916796	19510826	1	20121027	7
38940635	20120603	16474412	19950803	1	20120603	8
38942755	20121214	17072593	19490922	1	20121214	8
38953898	20120722	16626757	19640824	2	20120722	8
38956240	20130113	17159703	19740330	2	20130113	9
38972291	20130503	17493742	19520420	1	20130503	8
39012196	20121107	16952517	19850713	2	20121107	8
39046225	20120823	16724516	19980508	2	20120823	10
39051871	20121121	16995932	19630120	1	20121121	10
39067793	20130616	17625641	19691112	1	20130616	7
39098378	20121206	17041943	20070101	2	20121206	8
39111085	20121008	16863061	19470228	1	20121008	10
39126755	20130625	17652795	19491113	1	20130625	8
39139963	20120729	16645523	19661202	1	20120729	8
39149321	20130119	17180086	19601230	1	20130119	8
39180911	20130820	17836913	19590617	1	20130820	8
39183965	20121104	16938714	20010529	2	20121104	8
39201451	20120914	16791101	19740109	2	20120914	7
39202250	20130301	17292087	19540614	2	20130301	7
39216665	20121112	16967840	19660608	1	20121112	9
39287166	20130205	17229607	19490613	2	20130205	7
39297579	20130113	17159828	19591010	1	20130113	8
39306359	20121217	17081621	19751208	2	20121217	8
39310560	20121118	16985899	20020808	1	20121118	8
39341532	20131017	18020300	19560226	1	20131017	9
39376299	20130610	17608858	19480101	2	20130610	7
39385132	20130823	17845110	20031128	2	20130823	9
39511814	20131219	18221743	19661207	2	20131219	8
39517232	20130613	17619477	19680108	1	20130613	8
39559847	20130317	17345758	19590219	1	20130317	7
39585847	20131119	18125801	19570723	2	20131119	8
39612152	20130913	17914463	19950201	1	20130913	8
39645788	20130804	17780998	19520109	2	20130804	7
39645960	20130821	17837747	19401218	1	20130821	8
39649520	20131125	18139633	19390427	1	20131125	8
39667271	20130616	17625280	19560120	1	20130616	8
39699337	20130630	17665859	19621231	1	20130630	7
39724328	20130805	17785278	19651105	2	20130805	7
39737070	20130708	17696778	19951129	2	20130708	9
39774602	20130707	17693900	20050726	2	20130707	7
39905378	20130706	17693189	19880611	1	20130706	9
39910582	20130719	17735960	19601006	2	20130719	7

3993171020131023		18037188	19710906	1	20131023	8
3999783620131024		18040882	19621020	1	20131024	9
4004653520131228		18245308	19700425	2	20131228	8
4007533020130814		17818769	19641005	1	20130814	7
4015739920130921		17932690	19580927	2	20130921	7
4017801620131120		18129455	19850301	2	20131120	8
4018822520131111		18099288	19780909	2	20131111	8
4019691620131219		18223031	19521220	1	20131219	7
4025172720131006		17982252	19520913	1	20131006	7
1298653	20120520	16436935	19501124	2	20120520	7
262435320130517		17538837	19300222	2	20130517	7
3283087	20120701	16556923	19420905	2	20120701	10
3891698	20120517	16430555	19250115	1	20120517	8
7060846	20120828	16735622	19541215	2	20120828	8
7366134	20111103	15855762	19500102	2	20111103	8
7717773	20111218	15987284	19451201	2	20111218	7
8666497	20121014	16878389	19410116	1	20121014	9
878735720131030		18054135	19221123	1	20131030	7
8896024	20120404	16298618	19790911	1	20120404	8
9073147	20121122	17001273	19500514	1	20121122	8
9790141	20120531	16466089	19320913	2	20120531	7
993076120130910		17901847	19500220	1	20130910	9
11298729	20111230	16020735	19570702	2	20111230	10
11776253	20120828	16736310	19370813	2	20120828	8
12370160	20120209	16136963	19420126	2	20120209	7
1248146820131104		18072662	19531020	2	20131104	7
1264027620130506		17500910	19460310	2	20130506	9
1295578120131017		18020323	19480305	2	20131017	8
13183023	20120926	16824733	19290205	1	20120926	10
15679206	20121204	17036704	19521015	1	20121204	7
1659614820130822		17840947	19630303	1	20130822	8
16902424	20120510	16411119	19540915	2	20120510	8
17244143	20120209	16137148	19400101	2	20120209	8
17489508	20111201	15936443	19581112	1	20111201	7
1751880820121130		17021913	19621025	1	20121130	9
1773561420130618		17633781	19650717	2	20130618	8
18103816	20120716	16610774	19360219	2	20120716	10
1833641920131102		18066290	19561115	1	20131102	7
1872257120131126		18145406	19760323	2	20131126	7
1922833220130422		17456637	19510806	1	20130422	8
1985847020130626		17657879	19620910	1	20130626	8
21774958	20120724	16634100	19551025	2	20120724	8
2198088120130916		17921923	19580801	1	20130916	7
2198958220131104		18069182	19490310	1	20131104	7
22655169	20120323	16266785	19641221	2	20120323	10
22701184	20120307	16219225	19511122	1	20120307	7
22914450	20121115	16979767	19470502	2	20121115	10
23005529	20120816	16706626	19321211	1	20120816	10
23367819	20120213	16147711	19580201	2	20120213	10
24139524	20110725	15561022	19690811	2	20110725	8
24203938	20111118	15901067	19370224	2	20111118	9
2481767220130412		17429317	19490404	2	20130412	9
25214646	20120318	16250728	19440727	1	20120318	8
2548971820130812		17811192	19370805	2	20130812	8
26062748	20120425	16361133	19680517	1	20120425	8

26063229	20120417	16340630	19560820	1	20120417	7
26123682	20121127	17012256	19520823	2	20121127	10
26213467	20130910	17902567	19570503	2	20130910	7
27487634	20110830	15664315	19311019	2	20110830	7
27614897	20120403	16297083	19530301	1	20120403	7
27640239	20120322	16263790	19350325	1	20120322	7
28214655	20120130	16097994	19530622	2	20120130	7
28778576	20120915	16793896	19380803	1	20120915	8
28885974	20121128	17015807	19571124	1	20121128	10
29019849	20111225	16005535	19470405	2	20111225	7
29897754	20120316	16248460	19541023	2	20120316	8
29928754	20111221	15998052	19691030	2	20111221	10
30709081	20120928	16829991	19481102	2	20120928	8
31183978	20130811	17806870	19381006	2	20130811	7
31750046	20110618	15455422	19660908	2	20110618	8
32159265	20130429	17475230	19380808	1	20130429	7
32506288	20120131	16101492	19470310	1	20120131	9
32525614	20130724	17748830	19580204	1	20130724	8
32600365	20120923	16814576	19500414	1	20120923	7
32630641	20120129	16094740	19660120	1	20120129	10
32916419	20120218	16163816	19540607	2	20120218	10
33129101	20130115	17168067	19531208	2	20130115	8
33132002	20130422	17455824	19540926	2	20130422	8
33391536	20131007	17987470	19601215	1	20131007	8
33780766	20130227	17285669	19580308	1	20130227	7
33975358	20130522	17551854	19770214	2	20130522	7
34130524	20120713	16604493	19480208	1	20120713	7
34199649	20110829	15661444	20010528	2	20110829	10
34246418	20120503	16388890	19520306	2	20120503	10
34278847	20110913	15705913	19600120	2	20110913	9
34413315	20110922	15733066	19550102	2	20110922	9
34543356	20131014	18006799	19670420	1	20131014	8
34611126	20120529	16461033	19600730	1	20120529	7
34803333	20111027	15832282	19600119	2	20111027	7
34913661	20120528	16457909	19550925	2	20120528	8
34998953	20130619	17638106	19670830	2	20130619	7
35017062	20111021	15818434	19421023	2	20111021	10
35055391	20120514	16421620	19491229	2	20120514	10
35356071	20131126	18143563	19500910	1	20131126	7
35520060	20110815	15626116	19500402	2	20110815	8
35539754	20130802	17776695	19660512	2	20130802	7
35745790	20111221	15997952	19610118	2	20111221	8
35767498	20120416	16337001	19670615	1	20120416	7
35797967	20120617	16521071	19540208	2	20120617	7
35824556	20120505	16394522	19540415	1	20120505	7
35843404	20120311	16229679	19650606	1	20120311	8
35853077	20120320	16256860	19511115	2	20120320	10
35861724	20130509	17513700	19450304	1	20130509	7
36116913	20111006	15775784	20020712	2	20111006	9
36333150	20120322	16262380	19530612	1	20120322	7
36420812	20130602	17578710	19550102	2	20130602	7
36430203	20111213	15974390	19611004	1	20111213	9
36545376	20130722	17742063	19550310	1	20130722	8
36615200	20130218	17255488	19770509	2	20130218	9
36624449	20120128	16093379	19630502	1	20120128	8

36771456	20120217	16162011	19480726	2	20120217	10
36793665	20111210	15964451	19351206	2	20111210	8
36795398	20120928	16829362	19640327	1	20120928	8
36874176	20120229	16190441	19431006	1	20120229	10
36927261	20111127	15921811	19670610	2	20111127	10
36994420	20110708	15514607	19551201	1	20110708	8
37016345	20120905	16761927	19621104	1	20120905	7
3715978720131016		18016109	19580719	1	20131016	8
37389336	20120816	16706200	19461111	2	20120816	8
37395689	20121102	16936123	19600531	2	20121102	10
37437653	20111010	15782934	19590106	2	20111010	8
37559630	20111127	15921855	19591206	1	20111127	8
37693064	20111120	15903558	19871215	2	20111120	7
37708080	20111002	15756655	19830430	2	20111002	7
3776276820130215		17248098	19560815	1	20130215	7
37792066	20111111	15879798	19801028	2	20111111	10
37800398	20121017	16890351	19570222	1	20121017	9
37838123	20111031	15840530	19540812	1	20111031	10
37845480	20120111	16060011	19570426	1	20120111	7
37881100	20120829	16739200	19721002	1	20120829	8
37887517	20120218	16163907	19420624	1	20120218	10
3797342520130721		17738432	19520902	1	20130721	7
37979741	20120203	16117159	19390828	1	20120203	8
38077713	20110817	15632112	19580518	2	20110817	9
38078783	20120715	16606845	19750411	1	20120715	7
38086703	20111009	15782490	19820418	2	20111009	9
3811469120121217		17081450	19401127	1	20121217	8
38129496	20111110	15878052	19710419	2	20111110	10
3813667320130527		17561932	19540628	1	20130527	7
38169265	20121124	17006008	19410924	1	20121124	7
38196177	20120412	16328226	19490517	1	20120412	10
38210843	20111004	15766445	19580914	1	20111004	10
38218881	20121001	16837542	19940317	1	20121001	7
38220405	20120712	16597294	19560521	1	20120712	8
38238629	20120323	16266780	19541113	1	20120323	8
38252630	20111210	15966003	19680502	1	20111210	7
38258070	20120322	16263843	19620721	2	20120322	8
3826401620131006		17981938	19340625	2	20131006	8
3827110220130529		17570090	19620610	1	20130529	8
3831523020130707		17693594	19530731	2	20130707	8
38318091	20121109	16961153	19730605	2	20121109	8
38360188	20121217	17080756	19590101	1	20121217	7
38369481	20120701	16556933	19630722	1	20120701	8
38375643	20120209	16136630	19730224	1	20120209	10
38401693	20111210	15966093	19680606	2	20111210	8
3840375720131029		18052492	19611224	1	20131029	7
38416272	20111226	16009302	19760715	1	20111226	10
38425922	20120207	16129187	20040112	1	20120207	10
38486907	20120103	16032602	19950929	2	20120103	7
3848723920130424		17462973	19641208	1	20130424	8
38520091	20120504	16390229	19341111	1	20120504	7
38520273	20120120	16086836	19690101	1	20120120	10
3852228020131005		17981775	19680616	2	20131005	7
3852451520131227		18244059	19370116	2	20131227	9
38524695	20120627	16548323	19661216	1	20120627	7

38533776	20120201	16106784	19761113	2	20120201	7
38546360	20130521	17547420	19481018	1	20130521	7
38564237	20120911	16781412	19750727	1	20120911	8
38575654	20120212	16143389	19530509	1	20120212	9
38602050	20121205	17038121	19691228	2	20121205	9
38617639	20120130	16098285	19820320	1	20120130	10
38625046	20130218	17254022	19350312	1	20130218	8
38626323	20120921	16811094	19640120	1	20120921	8
38642772	20120825	16729342	19740905	2	20120825	7
38700924	20130527	17563765	19951104	1	20130527	9
38731758	20121002	16840908	19631006	2	20121002	10
38783210	20120220	16167605	19590110	1	20120220	9
38809835	20130225	17278292	19410521	2	20130225	7
38835868	20121217	17081539	19551106	1	20121217	8
38839882	20130801	17771624	19600115	1	20130801	8
38889995	20121018	16893212	19520421	1	20121018	8
38892852	20120808	16679798	19620211	1	20120808	10
38945050	20130923	17936801	19590416	2	20130923	7
38968308	20130421	17453367	19700904	2	20130421	9
39015139	20130805	17785536	19580302	1	20130805	8
39077004	20121103	16937959	19640902	1	20121103	10
39111085	20121030	16922327	19470228	1	20121030	9
39126982	20130225	17276809	19571227	1	20130225	7
39175547	20130108	17143122	19680606	1	20130108	8
39178693	20121127	17012087	19550415	1	20121127	7
39216665	20121130	17021044	19660608	1	20121130	10
39282230	20130131	17212355	19810426	1	20130131	7
39287166	20130211	17242913	19490613	2	20130211	7
39468581	20130531	17575736	19900329	1	20130531	7
39517232	20130815	17823583	19680108	1	20130815	8
39571750	20130401	17390304	19670306	1	20130401	9
39665764	20130812	17809498	19610120	2	20130812	7
39737070	20131014	18006359	19951129	2	20131014	7
39746786	20130829	17860282	19490107	2	20130829	7
39845788	20131007	17987482	19841112	1	20131007	8
39905378	20130817	17828799	19880611	1	20130817	9
39931710	20131211	18199230	19710906	1	20131211	8
39983056	20131015	18011887	19620614	1	20131015	8
40015541	20130927	17952754	19660820	2	20130927	7
40028351	20130827	17853482	19710809	1	20130827	8
40074826	20131107	18088567	19520910	1	20131107	8
40143359	20130921	17933520	19680802	2	20130921	9
40218937	20131026	18045524	19430207	1	20131026	8
1299747	20120926	16824545	19580525	1	20120926	10
2972454	20130710	17706964	19400126	2	20130710	8
3921537	20131015	18010921	19690801	2	20131015	8
6753237	20130801	17772688	19300922	1	20130801	8
7291261	20130122	17188207	19310801	1	20130122	8
7966383	20130215	17248724	19500423	1	20130215	7
8154356	20130331	17383669	19510327	1	20130331	7
8297787	20110605	15414537	19371111	2	20110605	10
8348821	20120302	16199239	19351211	1	20120302	7
9073147	20121217	17081652	19500514	1	20121217	9
9679112	20130102	17123341	19500116	2	20130102	8
9790141	20120618	16524511	19320913	2	20120618	8

11032545	20121121	16996735	19351207	2	20121121	10
11298729	20120409	16315671	19570702	2	20120409	8
12366517	20130429	17475188	19581208	1	20130429	9
12481468	20131119	18125959	19531020	2	20131119	7
13174179	20130626	17655544	19530112	2	20130626	9
13183023	20130215	17248562	19290205	1	20130215	8
13643680	20120621	16534074	19240520	1	20120621	7
13808565	20111212	15970320	19340827	2	20111212	8
13944053	20120306	16211723	19570816	1	20120306	8
13989816	20120828	16734819	19310202	1	20120828	8
14870145	20120729	16645215	19380705	2	20120729	7
16021128	20120801	16655354	19490824	2	20120801	7
16084530	20130820	17836981	19600224	1	20130820	9
18748599	20120503	16388769	19510403	1	20120503	10
19602790	20120827	16732622	19880622	1	20120827	8
19858470	20130710	17706726	19620910	1	20130710	9
20663267	20120901	16746448	19290430	1	20120901	8
20737706	20111122	15910102	19630522	2	20111122	7
21261234	20120503	16388572	19280707	2	20120503	7
21326501	20131116	18117746	19530315	1	20131116	9
21513020	20130712	17714189	19381101	1	20130712	8
21519437	20120320	16257352	19350219	1	20120320	8
21912572	20130108	17145255	19520128	1	20130108	8
22261423	20120729	16645374	19850628	2	20120729	9
22747724	20130228	17288305	19920529	2	20130228	8
23272711	20131127	18147144	19670820	2	20131127	8
24139524	20110812	15619692	19690811	2	20110812	10
24817672	20130618	17633605	19490404	2	20130618	8
24906103	20120813	16695040	19631101	1	20120813	7
26130370	20130510	17515900	19280816	2	20130510	8
26721697	20120709	16587563	19421210	2	20120709	9
27115395	20130831	17864585	19400428	2	20130831	7
27614897	20120506	16394805	19530301	1	20120506	9
27835354	20130514	17528091	19710112	2	20130514	9
28014779	20130328	17379174	19280608	2	20130328	7
28214655	20120326	16268966	19530622	2	20120326	8
28277534	20130126	17199785	19710519	1	20130126	9
28342383	20131122	18135978	19410622	1	20131122	8
28491432	20120807	16675080	19660409	2	20120807	7
28676917	20120327	16275279	19390408	2	20120327	7
29019849	20120302	16199143	19470405	2	20120302	8
29385646	20130717	17726714	19450215	2	20130717	8
29526690	20120402	16291280	19610922	2	20120402	8
29762945	20120524	16449119	19610125	2	20120524	9
29897754	20120503	16388563	19541023	2	20120503	10
30104166	20120421	16351256	19490217	2	20120421	8
30709081	20121016	16886246	19481102	2	20121016	7
31750046	20110711	15522985	19660908	2	20110711	8
31761816	20130317	17345702	19271023	1	20130317	7
31975323	20121115	16978763	20030417	2	20121115	8
31999607	20120720	16621877	19690426	2	20120720	9
32160217	20131102	18066258	19390120	2	20131102	8
32973141	20130501	17482476	19710629	1	20130501	7
33490389	20120514	16421586	19590320	2	20120514	7
33538464	20130619	17638145	19690425	2	20130619	9

3370685320130312		17331535	19560901	1	20130312	7
3371820620131222		18228227	19540925	2	20131222	7
3376753220130120		17180302	19571022	1	20130120	7
3378076620130331		17383705	19580308	1	20130331	8
3406295820130419		17450324	19390815	1	20130419	8
34199649	20110925	15738248	20010528	2	20110925	7
34339441	20111123	15913960	19470102	1	20111123	7
34377861	20120814	16699016	19490706	1	20120814	8
34438136	20120430	16373022	19481015	1	20120430	8
3494174720131224		18235172	19680311	1	20131224	7
3499895320130801		17772941	19670830	2	20130801	8
35017062	20111122	15910592	19421023	2	20111122	10
3502640520130505		17496337	20070712	1	20130505	9
35729896	20120105	16041287	19600705	2	20120105	7
35767498	20120508	16404172	19670615	1	20120508	8
35824556	20120522	16443187	19540415	1	20120522	7
35853077	20120510	16411530	19511115	2	20120510	8
35876983	20120206	16124716	19521101	2	20120206	8
3592640020130117		17175220	19681008	2	20130117	8
3607224920130408		17411428	19650903	2	20130408	8
36256618	20120417	16339670	19410628	2	20120417	7
36456834	20120326	16271338	19470401	2	20120326	7
36793665	20120204	16119337	19351206	2	20120204	10
36795398	20121107	16950633	19640327	1	20121107	7
36825475	20111128	15925319	19561031	1	20111128	7
36927261	20120413	16331069	19670610	2	20120413	8
3696344720130530		17573201	19490416	1	20130530	7
36994420	20110810	15612984	19551201	1	20110810	10
37155207	20110623	15469589	19580413	2	20110623	8
3715978720131223		18230693	19580719	1	20131223	8
37225062	20120519	16436578	19580610	1	20120519	7
37236058	20121001	16837281	19640825	1	20121001	7
37272096	20111204	15943263	19591113	1	20111204	10
37301181	20111005	15771179	19400430	1	20111005	7
37328282	20120625	16542037	19910901	1	20120625	7
37355207	20120509	16406965	19660311	2	20120509	7
37371394	20111006	15775818	19810707	2	20111006	7
37378180	20111224	16005407	19430307	1	20111224	10
37395689	20121224	17100173	19600531	2	20121224	9
37437653	20111023	15820520	19590106	2	20111023	9
37567876	20120821	16719218	19960817	1	20120821	9
37668034	20110718	15542935	19591202	1	20110718	7
37801131	20121121	16997689	19600215	2	20121121	8
37858789	20120618	16524746	20091028	2	20120618	9
37873420	20120929	16831164	19540103	2	20120929	9
37887517	20120315	16244226	19420624	1	20120315	9
37923334	20120327	16275234	19690205	2	20120327	8
3804758420130712		17714860	19510320	2	20130712	8
38125132	20110917	15718711	19510222	1	20110917	9
38137085	20120518	16435042	19560420	1	20120518	7
38211631	20110927	15744263	19401123	2	20110927	7
38217402	20120222	16172963	19480126	1	20120222	8
3822433820130304		17302387	19580410	2	20130304	8
38238629	20120417	16340647	19541113	1	20120417	8
3827110220130731		17765762	19620610	1	20130731	7

3835036620131228		18245224	20110118	1	20131228	8
38385227	20120720	16624036	19420212	1	20120720	7
3840375720131203		18167181	19611224	1	20131203	8
38416998	20120120	16086859	19630630	2	20120120	7
38419000	20121124	17006120	19530120	1	20121124	8
3842270720131010		17997129	19790206	1	20131010	9
38486907	20120130	16098230	19950929	2	20120130	7
38521356	20111225	16005986	19881209	1	20111225	9
3853030220130506		17501050	19581201	1	20130506	9
3857051320130718		17732643	19500926	1	20130718	9
38575654	20120328	16278276	19530509	1	20120328	10
3860205020130501		17484188	19691228	2	20130501	7
38605719	20120802	16657593	19691019	2	20120802	7
38626458	20120608	16497712	19740223	2	20120608	10
38637466	20120221	16171857	19690216	2	20120221	8
38656643	20120420	16349970	19650211	2	20120420	7
3866078520130922		17933681	19660601	1	20130922	8
3866091220130114		17163994	19800718	2	20130114	7
38663604	20120925	16821544	19440225	1	20120925	8
3866495820130314		17340038	19421218	1	20130314	7
38681264	20120326	16271846	19461229	1	20120326	8
3870092420130617		17629415	19951104	1	20130617	7
38754051	20120616	16520281	19530110	2	20120616	7
38814301	20120409	16315068	19771113	2	20120409	10
38866090	20120618	16524601	19590811	2	20120618	7
38877735	20120930	16831741	19741128	1	20120930	8
38892852	20121007	16858445	19620211	1	20121007	10
3891483920130822		17840655	19480812	1	20130822	7
38919801	20121110	16963036	19700429	1	20121110	8
38933992	20121220	17092620	19510826	1	20121220	7
3896830820130806		17789031	19700904	2	20130806	8
3901219620121224		17099684	19850713	2	20121224	9
3906147720130802		17778319	19511106	2	20130802	9
39103305	20120831	16742966	19641220	2	20120831	10
3915131020130726		17755804	19600906	2	20130726	9
3915556120130506		17501040	19571102	1	20130506	7
3917554720130219		17260156	19680606	1	20130219	8
3920978420131116		18117186	19400628	2	20131116	7
3928223020130222		17272008	19810426	1	20130222	8
3936828020130819		17833222	19480806	1	20130819	7
3940353720130306		17312602	19411007	1	20130306	7
3953207520130426		17469909	19550715	1	20130426	7
3988168020130830		17863291	19860325	2	20130830	7
3988184020130606		17598874	19400605	1	20130606	7
4001193620130915		17917720	19880207	2	20130915	7
4013105320130901		17865143	19550928	2	20130901	7
117888720131109		18094297	19670525	2	20131109	7
144566520130828		17858116	19510223	2	20130828	9
283275920130128		17203436	19250310	1	20130128	8
5912072	20120709	16588760	19560705	1	20120709	7
672331520130123		17190888	19500128	1	20130123	9
796638320130314		17339043	19500423	1	20130314	8
886770720130517		17537788	19460715	1	20130517	8
891453220130530		17573234	19470210	1	20130530	8
907314720130111		17157312	19500514	1	20130111	7

10568328	20120814	16697746	19430915	2	20120814	8
11032545	20130101	17117778	19351207	2	20130101	7
11298729	20120419	16345752	19570702	2	20120419	8
11324337	20111005	15771515	19431121	1	20111005	10
12366517	20130621	17644947	19581208	1	20130621	8
13808565	20111227	16010771	19340827	2	20111227	7
13846714	20120919	16805519	19551210	2	20120919	10
13944053	20120320	16256729	19570816	1	20120320	9
15258534	20130312	17332736	19530912	2	20130312	8
15679206	20130216	17250036	19521015	1	20130216	8
16636127	20120211	16140888	19310302	1	20120211	7
16669106	20120713	16604479	19260820	2	20120713	9
16803080	20131101	18063943	19521101	2	20131101	7
16902424	20120723	16630614	19540915	2	20120723	8
17104379	20110920	15726959	19720811	2	20110920	8
17237897	20131001	17963038	19351020	2	20131001	9
17617708	20120615	16517365	19250715	1	20120615	8
17745856	20121120	16993623	19300205	1	20121120	8
18748599	20120606	16489520	19510403	1	20120606	10
19943716	20131009	17996448	19460224	1	20131009	8
20361562	20120428	16369147	19480326	2	20120428	8
20737706	20111214	15977259	19630522	2	20111214	8
21774958	20121002	16840651	19551025	2	20121002	7
21960838	20120510	16412162	19370116	2	20120510	7
22023172	20130703	17682183	19371017	2	20130703	7
22514790	20111219	15990362	19410120	2	20111219	7
22914450	20130205	17230730	19470502	2	20130205	8
23092866	20130525	17559462	19471110	2	20130525	8
23156561	20130121	17184088	19620607	2	20130121	8
23367819	20120515	16424927	19580201	2	20120515	8
24203938	20111213	15970777	19370224	2	20111213	10
25157408	20120528	16458116	19361218	1	20120528	7
25586509	20131018	18023553	19630221	2	20131018	9
26063229	20120917	16797448	19560820	1	20120917	7
26213467	20131106	18083039	19570503	2	20131106	8
26721697	20120726	16640440	19421210	2	20120726	7
27614897	20120603	16474653	19530301	1	20120603	10
27835354	20130524	17557852	19710112	2	20130524	8
27871712	20130910	17901789	19420716	1	20130910	7
29073163	20130216	17250180	19520930	2	20130216	7
30160384	20120101	16022605	19701017	1	20120101	7
30587258	20120624	16538036	19621203	1	20120624	7
30709081	20130331	17384038	19481102	2	20130331	8
31236610	20110524	15381784	19370917	2	20110524	10
31363152	20110920	15726954	19751022	1	20110920	9
31548762	20120316	16248330	19641118	1	20120316	10
31750046	20110825	15654365	19660908	2	20110825	8
32401528	20111029	15837493	19280405	1	20111029	10
32402101	20120528	16458108	19371022	1	20120528	8
32506288	20120504	16392706	19470310	1	20120504	9
32818498	20120124	16089336	19651207	1	20120124	8
32973141	20130605	17594221	19710629	1	20130605	8
32980782	20120815	16702983	19581015	2	20120815	7
33118331	20110725	15560665	19420927	2	20110725	10
34128466	20120410	16317372	19760221	2	20120410	7

3430561820130615		17625045	19760513	1	20130615	8
3499895320130814		17818745	19670830	2	20130814	7
35086067	20111102	15851703	19371212	1	20111102	7
35510282	20120612	16508117	19690726	2	20120612	10
35729896	20120411	16324369	19600705	2	20120411	7
35797967	20120709	16588842	19540208	2	20120709	9
35843404	20120708	16584228	19650606	1	20120708	10
35982866	20121017	16889047	19681016	2	20121017	7
36170157	20110829	15661676	19550126	1	20110829	8
3641438720130617		17629496	19731128	2	20130617	7
36456834	20121114	16975926	19470401	2	20121114	8
3662444920131121		18131891	19630502	1	20131121	8
36825475	20111212	15970740	19561031	1	20111212	8
3696344720130605		17594086	19490416	1	20130605	7
36994420	20110921	15728901	19551201	1	20110921	8
37012854	20110811	15614593	19360507	1	20110811	10
37076703	20121130	17022180	19520515	2	20121130	8
37299835	20120228	16187599	19920514	1	20120228	7
3733209520130206		17233800	19430104	2	20130206	7
3736689520130129		17206473	19631021	2	20130129	9
3737450820131212		18203267	19691012	1	20131212	7
37378180	20111229	16016906	19430307	1	20111229	7
37437653	20111107	15866689	19590106	2	20111107	10
3756592720130407		17407394	19370308	1	20130407	8
37568368	20111222	16000982	19591025	2	20111222	7
37829713	20120514	16420615	19750101	1	20120514	8
37857775	20110919	15723265	19750708	2	20110919	10
37882818	20111101	15844776	19610712	1	20111101	8
37970584	20120215	16155581	19610115	2	20120215	7
38078783	20120911	16778994	19750411	1	20120911	9
38124980	20111018	15807045	20010707	1	20111018	8
38130255	20120902	16747096	19390508	1	20120902	7
38132900	20120710	16593051	19700803	1	20120710	7
38137085	20120628	16552128	19560420	1	20120628	7
38211631	20111108	15869817	19401123	2	20111108	10
38220405	20120828	16736316	19560521	1	20120828	8
38295497	20111101	15846407	19511220	1	20111101	7
38369481	20120808	16678081	19630722	1	20120808	10
38375643	20120309	16227018	19730224	1	20120309	7
3842270720131019		18025442	19790206	1	20131019	8
38524695	20120911	16781008	19661216	1	20120911	8
38562231	20121124	17006042	19500611	2	20121124	8
38630896	20120613	16511777	19480301	1	20120613	8
38642772	20120927	16824784	19740905	2	20120927	10
3866082120130220		17264505	19600901	1	20130220	9
3868758020121227		17110330	19450220	2	20121227	8
38698429	20121022	16902417	19580226	2	20121022	7
3870092420130709		17702873	19951104	1	20130709	8
3877364720130103		17128317	19691119	2	20130103	8
3878280820130830		17862879	19730313	1	20130830	8
3884857620130416		17440367	19500713	1	20130416	7
38877735	20121014	16878375	19741128	1	20121014	8
38892852	20121101	16931413	19620211	1	20121101	9
3893399220130106		17135507	19510826	1	20130106	8
3906147720130827		17854773	19511106	2	20130827	9

39097137	20121015	16882428	19960620	1	20121015	7
39193663	20130403	17399763	19520608	1	20130403	8
39231486	20130418	17447939	19611211	1	20130418	7
39279635	20130407	17407558	19680517	1	20130407	9
39292712	20130603	17584306	19661031	1	20130603	8
39300088	20130128	17202583	19581101	2	20130128	7
39310560	20130226	17281191	20020808	1	20130226	8
39331174	20130307	17317145	19700912	2	20130307	8
39370542	20130627	17660923	19680819	2	20130627	8
39526119	20131217	18216200	19741014	1	20131217	8
39588799	20130722	17741215	19580622	2	20130722	8
39645788	20131031	18057960	19520109	2	20131031	8
39746786	20131030	18053216	19490107	2	20131030	8
39881680	20130930	17958248	19860325	2	20130930	9
40011936	20131130	18156101	19880207	2	20131130	9
40131053	20130930	17957654	19550928	2	20130930	7
40141751	20131201	18156803	19570805	1	20131201	7
822300	20121115	16979885	19491010	2	20121115	8
2516134	20121106	16948484	19580107	1	20121106	10
2979717	20130330	17383429	19440218	1	20130330	8
7389404	20131202	18162735	19240815	2	20131202	8
7695525	20120128	16094202	19510818	1	20120128	8
9073147	20130226	17281526	19500514	1	20130226	9
11163114	20130903	17875613	19570815	1	20130903	9
12366517	20130701	17669191	19581208	1	20130701	9
12876947	20130829	17860099	19530628	2	20130829	9
14823155	20120725	16637532	19630729	2	20120725	9
15906840	20130629	17665391	19530107	2	20130629	7
16472154	20120416	16336808	19270304	2	20120416	10
16746682	20120111	16060058	19611028	2	20120111	7
17104379	20111026	15830556	19720811	2	20111026	10
17617708	20120818	16711175	19250715	1	20120818	7
17827268	20130320	17356714	19450717	2	20130320	7
19566406	20120808	16678520	19430316	1	20120808	7
19943716	20131130	18156021	19460224	1	20131130	7
20577291	20120813	16695013	19550520	1	20120813	9
22914450	20130221	17266883	19470502	2	20130221	8
23092866	20130615	17624991	19471110	2	20130615	8
23156561	20130225	17277825	19620607	2	20130225	7
24203938	20120102	16025167	19370224	2	20120102	8
25045323	20131030	18054220	19640928	2	20131030	8
26157426	20111201	15936558	19790509	2	20111201	8
26213467	20131220	18226510	19570503	2	20131220	8
26721697	20120907	16770365	19421210	2	20120907	8
27640239	20120528	16456613	19350325	1	20120528	7
28214655	20120803	16660096	19530622	2	20120803	7
28386983	20120619	16526838	19470105	2	20120619	7
29385646	20130817	17828720	19450215	2	20130817	7
30160384	20120213	16146264	19701017	1	20120213	10
30313292	20120106	16044914	19620108	2	20120106	7
30587258	20120808	16678833	19621203	1	20120808	7
30759263	20130130	17209560	19770826	1	20130130	9
31548762	20120413	16331537	19641118	1	20120413	10
31809106	20130415	17436116	19500820	2	20130415	7
32506288	20120802	16657575	19470310	1	20120802	7

3257943420130722		17741839	19281026	1	20130722	7
3297314120130707		17693771	19710629	1	20130707	9
32980782	20121102	16936062	19581015	2	20121102	10
33118331	20110921	15730450	19420927	2	20110921	8
3312910120130406		17406782	19531208	2	20130406	7
3376753220130306		17311665	19571022	1	20130306	7
34091139	20120217	16161334	19331130	1	20120217	10
34223944	20120305	16209498	19291012	1	20120305	10
34278847	20120124	16089095	19600120	2	20120124	10
34438136	20120623	16537585	19481015	1	20120623	7
34803333	20120610	16500255	19600119	2	20120610	10
35351112	20110618	15455269	19500415	2	20110618	10
35687162	20120603	16474844	19700809	2	20120603	10
35729896	20121220	17091570	19600705	2	20121220	7
35876983	20120320	16256825	19521101	2	20120320	10
35982866	20121118	16986085	19681016	2	20121118	8
3607224920130916		17921918	19650903	2	20130916	7
36151038	20120926	16821858	19820531	2	20120926	9
3639627720131105		18077661	19350731	1	20131105	8
3641438720130805		17785750	19731128	2	20130805	7
36612949	20110510	15343300	19660806	2	20110510	8
3661520020130521		17548462	19770509	2	20130521	7
3674655120130606		17597724	19531014	1	20130606	8
36791738	20121102	16936090	19650105	2	20121102	7
3680227420130403		17397529	19600301	1	20130403	7
37124008	20120203	16115644	19500518	2	20120203	7
37274718	20120131	16101378	20091219	1	20120131	8
37299835	20120402	16290696	19920514	1	20120402	8
37334784	20120220	16168296	19560117	2	20120220	8
37397221	20110914	15710563	19440220	1	20110914	8
37559630	20120227	16182971	19591206	1	20120227	8
37676156	20121007	16858440	19500506	2	20121007	8
37713216	20120504	16391998	19620615	1	20120504	10
3771529020130928		17954259	19560126	1	20130928	9
3785747920131028		18049346	20061003	1	20131028	9
3800210720130215		17247716	19590427	1	20130215	8
38128379	20120623	16537382	19410822	1	20120623	10
38217402	20120408	16311133	19480126	1	20120408	7
38218881	20121214	17075069	19940317	1	20121214	7
38220405	20120922	16813985	19560521	1	20120922	8
38231015	20121130	17022114	19391111	1	20121130	7
38425922	20120410	16319332	20040112	1	20120410	9
3845212920130122		17186967	19500103	2	20130122	7
38627224	20121212	17067288	19771002	1	20121212	8
38630896	20120802	16657743	19480301	1	20120802	7
38642772	20121108	16956842	19740905	2	20121108	7
38658274	20120708	16584381	19661214	1	20120708	7
3868125320130709		17702051	19710109	2	20130709	7
3870092420130814		17819593	19951104	1	20130814	7
38721061	20120623	16537642	19880707	1	20120623	10
3878280820130918		17929044	19730313	1	20130918	7
38785556	20120821	16717909	19590302	2	20120821	10
38814301	20120703	16567131	19771113	2	20120703	10
3881754820130728		17757439	19701101	2	20130728	8
3881868720130405		17403695	19581025	1	20130405	7

3887773520121122		17000486	19741128	1	20121122	8
3891483920130917		17923019	19480812	1	20130917	8
3893399220130226		17282019	19510826	1	20130226	9
3894663320130219		17258876	19560520	2	20130219	7
38974651	20120827	16733159	19530710	1	20120827	10
3898677520130217		17250756	19740519	1	20130217	7
39016109	20120921	16812286	19661010	2	20120921	7
3906147720131002		17969931	19511106	2	20131002	9
3907354620130430		17477695	19610120	1	20130430	8
3907595120130213		17244124	19390316	1	20130213	9
3910111620130722		17741752	19491129	1	20130722	8
3912249120131207		18184778	19671112	2	20131207	7
3917869320130121		17184385	19550415	1	20130121	8
3929271220130619		17637268	19661031	1	20130619	8
3951952320130715		17720155	19501112	1	20130715	8
3957418020130825		17847376	19611109	1	20130825	8
3968735920130917		17925949	19351129	1	20130917	7
3973291720131020		18025772	19800203	1	20131020	7
3996318320130722		17738553	19500923	1	20130722	7
82230020130505		17496102	19491010	2	20130505	8
2010711	20120812	16690594	19461008	2	20120812	9
3783742	20121111	16963604	19420522	2	20121111	7
4398576	20111230	16020321	19521001	2	20111230	7
5104858	20111229	16018463	19430711	2	20111229	7
6471563	20111111	15881166	19460801	1	20111111	7
14538659	20120916	16794357	19281227	1	20120916	8
16376800	20120521	16440666	19770111	1	20120521	8
1659614820131225		18237911	19630303	1	20131225	8
1686467820131126		18145449	19460602	2	20131126	7
16927610	20120511	16414468	19660713	2	20120511	8
20368074	20121008	16862205	19590322	2	20121008	8
2058131120130815		17823347	19630819	1	20130815	8
2309286620130721		17738228	19471110	2	20130721	8
23101635	20120803	16662292	19510825	2	20120803	8
2315656120130307		17317770	19620607	2	20130307	8
24654748	20121101	16927073	19940414	1	20121101	7
2490610320130121		17183753	19631101	1	20130121	8
25129468	20120504	16391737	19521105	1	20120504	8
26721697	20121018	16892968	19421210	2	20121018	7
2693411220131213		18206516	19430726	1	20131213	8
28181295	20120202	16112622	19460211	2	20120202	8
28214655	20121008	16861478	19530622	2	20121008	10
28377119	20120523	16447108	19320210	2	20120523	7
29019849	20120614	16515303	19470405	2	20120614	7
2962567020130711		17711764	19600220	1	20130711	8
2974721520130110		17152647	19530718	1	20130110	7
3041347920131225		18238229	19510501	2	20131225	7
31363152	20120110	16055602	19751022	1	20120110	8
31430418	20120511	16415494	19461125	1	20120511	8
32506288	20120903	16752670	19470310	1	20120903	7
32854516	20121127	17012235	19530309	1	20121127	8
32980782	20121115	16979057	19581015	2	20121115	8
33118331	20111117	15894927	19420927	2	20111117	10
34043588	20120815	16703045	19520817	2	20120815	7
34199649	20111129	15928262	20010528	2	20111129	8

34264341	20120109	16051877	19510502	1	20120109	9
34803333	20120626	16544056	19600119	2	20120626	9
35143498	20120403	16297029	19340707	1	20120403	7
35386528	20120806	16670478	19630127	1	20120806	10
35729896	20130420	17452905	19600705	2	20130420	8
35797967	20120808	16679790	19540208	2	20120808	7
36068005	20121210	17057838	20030123	2	20121210	10
36072249	20131022	18033168	19650903	2	20131022	9
36094663	20120705	16577952	19501012	1	20120705	8
36151038	20121217	17078327	19820531	2	20121217	7
36170157	20111004	15766949	19550126	1	20111004	7
36414387	20130826	17851053	19731128	2	20130826	7
36612949	20110516	15360244	19660806	2	20110516	8
36615200	20130701	17671780	19770509	2	20130701	9
36659957	20120815	16703065	19511202	1	20120815	8
36757456	20120610	16500280	19560310	2	20120610	10
36927261	20120728	16645003	19670610	2	20120728	9
37225062	20120809	16684525	19580610	1	20120809	7
37324917	20120401	16285588	19510613	2	20120401	10
37500291	20120531	16466344	19650420	1	20120531	8
37572819	20130524	17558129	19710702	2	20130524	8
37792066	20120218	16162441	19801028	2	20120218	10
37834029	20111129	15927586	19910130	2	20111129	7
37858789	20120823	16725118	20091028	2	20120823	9
37873475	20131005	17981676	19400409	1	20131005	7
37909425	20120326	16268933	19620627	2	20120326	7
38018610	20111101	15846834	19850504	1	20111101	7
38187596	20120920	16808839	19470428	2	20120920	8
38211631	20120229	16188921	19401123	2	20120229	8
38293559	20120818	16711302	19541120	2	20120818	10
38486907	20120415	16333747	19950929	2	20120415	8
38555930	20121122	16999924	19610830	1	20121122	7
38567054	20120922	16814053	20090613	1	20120922	9
38658274	20120926	16823933	19661214	1	20120926	7
38683657	20130922	17933874	19570826	2	20130922	7
38721061	20120815	16702936	19880707	1	20120815	8
38751085	20121106	16948480	19540701	1	20121106	7
38789605	20120715	16606817	19561031	1	20120715	7
38801115	20130402	17395990	19580906	2	20130402	7
38811039	20121204	17036461	19471101	1	20121204	10
38814301	20120712	16599833	19771113	2	20120712	10
38877735	20130125	17198043	19741128	1	20130125	8
38882632	20130914	17917157	19570225	1	20130914	9
38940635	20121105	16942915	19950803	1	20121105	7
38974651	20120914	16791601	19530710	1	20120914	8
39022190	20130704	17687116	19660311	2	20130704	7
39149321	20130411	17425732	19601230	1	20130411	8
39165269	20130623	17647215	19940908	1	20130623	9
39178693	20130204	17225965	19550415	1	20130204	7
39407379	20130418	17447655	19551127	2	20130418	8
39532075	20130609	17605456	19550715	1	20130609	8
39570246	20130804	17781105	19361118	2	20130804	7
39647591	20130924	17942143	19961219	1	20130924	8
39653720	20131223	18230595	19600224	1	20131223	7
39729754	20131013	18003410	19520915	1	20131013	8

39732917	20131108	18091807	19800203	1	20131108	7
1445665	20131206	18180772	19510223	2	20131206	7
2215294	20111115	15891082	19300820	1	20111115	7
2516134	20121226	17106645	19580107	1	20121226	7
7519893	20121228	17113457	19540813	1	20121228	8
9961584	20130117	17175085	19560308	1	20130117	7
11515483	20120417	16340563	19510304	2	20120417	8
13019506	20120930	16831743	19580324	1	20120930	8
14823155	20121106	16947698	19630729	2	20121106	7
16376800	20120626	16545606	19770111	1	20120626	7
17104379	20120306	16214587	19720811	2	20120306	10
20577291	20121024	16909096	19550520	1	20121024	10
21011814	20120306	16214491	19400921	1	20120306	8
21333200	20120214	16152030	19550826	2	20120214	9
21544456	20130102	17122465	19500317	1	20130102	8
23156561	20130401	17390243	19620607	2	20130401	8
23874320	20111231	16022023	19530817	1	20111231	8
24654748	20121127	17011728	19940414	1	20121127	9
26741833	20120723	16630708	19651126	2	20120723	7
27172925	20120529	16461066	19651215	2	20120529	7
28989788	20120415	16333670	19800702	1	20120415	10
30759263	20130329	17382117	19770826	1	20130329	8
31236610	20110917	15718513	19370917	2	20110917	7
32811931	20130325	17367874	19321217	2	20130325	8
33526180	20130311	17326528	19551205	1	20130311	7
33633817	20130916	17920870	19491012	2	20130916	7
33676390	20131129	18154875	19651228	1	20131129	7
33767532	20130417	17442206	19571022	1	20130417	7
34189952	20121224	17099749	19421125	1	20121224	7
34400232	20130409	17416398	19280726	1	20130409	9
34438136	20120710	16592278	19481015	1	20120710	7
34444694	20120530	16463702	19611019	1	20120530	10
35086067	20111124	15917011	19371212	1	20111124	7
35687162	20120726	16640192	19700809	2	20120726	9
35946022	20120426	16364099	19481112	2	20120426	7
36612949	20110610	15432416	19660806	2	20110610	9
36615200	20130810	17806210	19770509	2	20130810	8
36659957	20121127	17013199	19511202	1	20121127	10
37076703	20130322	17363824	19520515	2	20130322	9
37161403	20130716	17723338	19710108	1	20130716	7
37225062	20120929	16831319	19580610	1	20120929	9
37366522	20120413	16330798	19480805	2	20120413	7
37456001	20120917	16798423	19461130	1	20120917	10
37500291	20120622	16535857	19650420	1	20120622	8
37694614	20120825	16729275	19571212	2	20120825	8
37720539	20120420	16349593	19500110	2	20120420	7
37792066	20120528	16458175	19801028	2	20120528	8
37857775	20120109	16051252	19750708	2	20120109	8
37909425	20120331	16284538	19620627	2	20120331	7
37922853	20120529	16460613	19570722	1	20120529	8
37970584	20120510	16411980	19610115	2	20120510	7
38002107	20130302	17296407	19590427	1	20130302	9
38018610	20120127	16091705	19850504	1	20120127	7
38078783	20121120	16993576	19750411	1	20121120	8
38118842	20120521	16440704	19550919	2	20120521	9

38211631	20120413	16331153	19401123	2	20120413	8
3821888120130216		17249628	19940317	1	20130216	8
38567054	20121126	17010105	20090613	1	20121126	8
3864199520130129		17206660	19751021	2	20130129	8
3864277220130109		17149605	19740905	2	20130109	9
3865605220130825		17847612	19401120	1	20130825	7
3866082120130515		17531844	19600901	1	20130515	8
3869842920130107		17139624	19580226	2	20130107	8
38721061	20120827	16733067	19880707	1	20120827	10
38789605	20120805	16664984	19561031	1	20120805	9
3880961920130814		17819604	19760906	1	20130814	7
38851433	20120905	16762338	19630707	1	20120905	8
3887773520130215		17248681	19741128	1	20130215	8
3888263220131020		18025674	19570225	1	20131020	9
3889285220130914		17915316	19620211	1	20130914	8
3891483920131118		18120605	19480812	1	20131118	8
3900437020130119		17179204	19620310	1	20130119	7
39034736	20121003	16846353	19500118	1	20121003	7
3903735920130327		17376254	19830905	2	20130327	7
3906147720131129		18154920	19511106	2	20131129	8
3907354620131003		17973145	19610120	1	20131003	7
3914932120130613		17617958	19601230	1	20130613	8
3917869320130226		17278383	19550415	1	20130226	8
3926548020131001		17964573	19500502	1	20131001	7
3972169220130930		17958254	19980807	1	20130930	8
3991321820131026		18045577	19641130	2	20131026	8
3998999620131003		17975285	19580324	1	20131003	7
82230020130728		17757781	19491010	2	20130728	8
144566520131215		18208822	19510223	2	20131215	9
2010711	20120917	16796033	19461008	2	20120917	10
220398720130919		17929919	19700730	1	20130919	9
642764120130820		17837001	19610110	1	20130820	9
745243920130709		17702500	19760602	1	20130709	8
751989320130111		17157337	19540813	1	20130111	7
967911220130923		17937582	19500116	2	20130923	7
996158420130219		17256958	19560308	1	20130219	7
12413444	20120706	16581036	19311115	2	20120706	8
15371003	20120131	16099804	19350225	2	20120131	8
1815842420130130		17209515	19631226	2	20130130	9
1833431120130402		17396080	19531112	1	20130402	9
2111119320131119		18124601	19481226	1	20131119	8
21430375	20120930	16831505	19351208	2	20120930	10
2154445620130207		17239162	19500317	1	20130207	8
2176400120131027		18046100	19600125	1	20131027	7
2315656120130417		17443375	19620607	2	20130417	7
2354314820130617		17629579	19531201	2	20130617	8
27859821	20120304	16204190	19590226	2	20120304	8
30313292	20120607	16493515	19620108	2	20120607	7
3058725820130412		17429372	19621203	1	20130412	7
3092699920130712		17714955	19690216	2	20130712	8
3179200420130215		17248143	19701101	2	20130215	8
31894970	20120309	16225878	19641124	2	20120309	7
3353846420130916		17921935	19690425	2	20130916	7
3376753220130522		17550397	19571022	1	20130522	9
34199649	20120131	16101587	20010528	2	20120131	7

34488396	20120921	16811668	19640224	1	20120921	7
35946022	20121121	16996840	19481112	2	20121121	9
3606800520130103		17127652	20030123	2	20130103	7
36170157	20111107	15866485	19550126	1	20111107	10
36612949	20110616	15449219	19660806	2	20110616	8
3661520020131011		18000524	19770509	2	20131011	9
36757456	20120626	16545333	19560310	2	20120626	7
3693199420121222		17097129	19680302	1	20121222	8
3695276820131125		18140781	19560730	2	20131125	9
3727313520130228		17288146	19451119	1	20130228	9
37299835	20121031	16924094	19920514	1	20121031	7
37694614	20121021	16899036	19571212	2	20121021	7
37745883	20120322	16263566	19490404	2	20120322	8
37792066	20120624	16537976	19801028	2	20120624	8
37929707	20121117	16985470	19440401	1	20121117	7
38118842	20120719	16621822	19550919	2	20120719	7
38195152	20121002	16840687	19780814	1	20121002	10
38205139	20120702	16562902	19410801	1	20120702	7
3821084320131216		18212671	19580914	1	20131216	9
38211631	20120601	16471053	19401123	2	20120601	7
3825742020130718		17732260	19600916	1	20130718	8
3829938620131226		18239588	19630519	1	20131226	8
3841376220131114		18112421	19500320	1	20131114	8
3849750620130812		17806962	19510801	1	20130812	8
38576293	20120502	16380457	19940410	1	20120502	7
3858965020130716		17724519	19920902	1	20130716	7
3862722420130727		17757273	19771002	1	20130727	8
3864277220130223		17273768	19740905	2	20130223	7
38656552	20120706	16581932	19640101	1	20120706	8
3866082120130603		17584418	19600901	1	20130603	8
38693731	20120723	16630029	19901206	1	20120723	8
3869842920130228		17287102	19580226	2	20130228	8
3878280820131127		18148034	19730313	1	20131127	9
3886609020131126		18144427	19590811	2	20131126	8
3887773520130311		17328514	19741128	1	20130311	9
3891483920131206		18182790	19480812	1	20131206	7
3910179620130501		17484156	19501126	1	20130501	9
3917869320130309		17323776	19550415	1	20130309	7
3920277220130810		17805483	19761006	1	20130810	8
3945128020131125		18139875	19610915	2	20131125	7
3953585820130730		17762694	19670627	2	20130730	7
3991321820131031		18058785	19641130	2	20131031	8
220398720131017		18020256	19700730	1	20131017	9
1110871720130917		17924481	19550816	2	20130917	8
13019506	20121028	16917298	19580324	1	20121028	8
13302095	20120109	16050192	19400602	2	20120109	8
1403153920130302		17296169	19591024	2	20130302	7
20368074	20121206	17042344	19590322	2	20121206	7
2176400120131208		18185196	19600125	1	20131208	8
23874320	20120229	16189726	19530817	1	20120229	7
26581248	20120902	16746936	19970529	2	20120902	10
28989788	20121013	16877711	19800702	1	20121013	8
29856960	20120521	16440664	19520102	2	20120521	9
3092699920130723		17743197	19690216	2	20130723	8
3131905820130527		17564037	19790916	1	20130527	8

31363152	20120920	16809307	19751022	1	20120920	8
33576613	20120113	16067417	19671121	1	20120113	10
34488396	20121009	16864365	19640224	1	20121009	7
35982866	20130411	17425701	19681016	2	20130411	9
36068005	20130123	17191352	20030123	2	20130123	8
36105370	20121007	16858466	19650719	2	20121007	7
36170157	20111207	15954204	19550126	1	20111207	8
36615200	20131216	18212126	19770509	2	20131216	9
36693922	20130127	17199925	19580111	2	20130127	8
36731450	20121010	16867757	19740304	2	20121010	8
36791738	20130125	17197868	19650105	2	20130125	7
36973587	20120409	16314222	19570221	1	20120409	9
37299835	20121112	16966677	19920514	1	20121112	8
37494410	20130804	17781040	19780206	1	20130804	7
37943990	20120325	16268812	19590220	2	20120325	7
38006289	20130228	17288274	19500102	1	20130228	8
38130255	20130222	17271793	19390508	1	20130222	8

nax
